# Stable Isotope Dynamic Labeling of Secretomes (SIDLS) Identifies Authentic Secretory Proteins Released by Cancer and Stromal Cells*[Fn FN2]

**DOI:** 10.1074/mcp.TIR117.000516

**Published:** 2018-06-18

**Authors:** Dean E. Hammond, J. Dinesh Kumar, Lorna Raymond, Deborah M. Simpson, Robert J. Beynon, Graham J. Dockray, Andrea Varro

**Affiliations:** From the ‡Department of Cellular and Molecular Physiology, Institute of Translational Medicine, University of Liverpool, Crown St, Liverpool, UK;; §Centre for Proteome Research, Institute of Integrative Biology, University of Liverpool, Crown St, Liverpool, UK

**Keywords:** Secretome, SILAC, Cancer Biology, Exocytosis, Cell secretion, Dynamic labelling, Short term labelling

## Abstract

Analysis of secretomes critically underpins the capacity to understand the mechanisms determining interactions between cells and between cells and their environment. In the context of cancer cell micro-environments, the relevant interactions are recognized to be an important determinant of tumor progression. Global proteomic analyses of secretomes are often performed at a single time point and frequently identify both classical secreted proteins (possessing an N-terminal signal sequence), as well as many intracellular proteins, the release of which is of uncertain biological significance. Here, we describe a mass spectrometry-based method for stable isotope dynamic labeling of secretomes (SIDLS) that, by dynamic SILAC, discriminates the secretion kinetics of classical secretory proteins and intracellular proteins released from cancer and stromal cells in culture. SIDLS is a robust classifier of the different cellular origins of proteins within the secretome and should be broadly applicable to nonproliferating cells and cells grown in short term culture.

Protein secretion critically supports a diverse range of cellular functions including cell-cell and cell-matrix interactions, as well as specialized functions such as hormone or digestive enzyme release. The constitutive secretion of proteins is a property of all cells, whereas regulated secretion (*i.e.* dependent on release of preformed stores after increased intracellular Ca^2+^) occurs in specialized cells including neurons, endocrine and exocrine cells. It is now appreciated that an understanding of secretomes (the totality of secreted proteins) is of crucial importance in health and disease (1–4). For example, the secretomes of cancer and stromal cells contribute strongly to the cellular microenvironment that determines tumor progression (5). Thus, secretome studies have proven attractive both because they may provide insight into mechanisms of disease and because they facilitate the discovery of biomarkers that can be used for diagnosis, staging and monitoring of therapy.

Despite considerable progress in developing methods for secretome profiling (6–8) there remain problematical issues in interpretation of the data. Such studies frequently identify “classical” secreted proteins defined by an N-terminal signal sequence, but they also identify many intracellular proteins, the apparent secretion of which is often of uncertain significance and not readily discriminated from tissue leakage/cell death (9). Interpretation is further compounded by the fact that many studies are performed at a single time point, such that kinetic differences in the release of different components of the secretome are obscured. The classification of secretome proteins by gene ontology (GO)^1^ terms or predictions from computational tools/algorithms such as SignalP (10) or SecretomeP (11) can be used to segregate classically secreted proteins from intracellular proteins. However, experimental approaches that support this classification would be of obvious advantage. For example, a triple-labeling, single time point approach was adopted by Kristensen and colleagues (12), in which they pointed out that the extent of labeling could be used to discriminate newly synthesized secretome proteins and those that were mobilized from pre-existing stores. Here, we extend this thinking by describing a mass spectrometry (MS)-based strategy using stable isotope dynamic labeling of secretomes (SIDLS) that discriminates between classical secretory proteins and intracellular proteins within the secretome of cultured cells. The method differs from traditional SILAC, in which proteins are labeled for a fixed period to ensure all are fully labeled. Further, it differs from the single time point pulsed SILAC approach (12) through dynamic labeling, in which the progressive incorporation of label into proteins is monitored over time. We demonstrate that a time dependence of labeling is of considerable value in the study of cell secretomes. A kinetic approach exploits the different labeling kinetics of classical secretory proteins that exhibit rapid incorporation of label compared with the much slower labeling of the bulk of intracellular proteins, even though some of the latter are present in the secretome. By monitoring the rate of incorporation of labeled amino acids into newly synthesized proteins as they appear in the media, we can differentiate those proteins that have been destined for secretion from those with low rates of labeling or low turnover relative to the growth rate of the cells, a feature of intracellular proteins.

## EXPERIMENTAL PROCEDURES

### 

#### 

##### Cell Culture

Human primary cancer-associated myofibroblasts (CAMs) were derived from resected human esophageal squamous cancer tissue, obtained from patients as described previously (13). Esophageal squamous cell cancer cells, OE21, were purchased from American Type Culture Collection (Manassas, VA). All cells were maintained at 37 °C, in 5% v/v CO_2_, and cultured in DMEM, supplemented with 10% v/v FBS as previously described (14).

##### Stable Isotopic Dynamic Labeling, Mass Spectrometry and Protein Identification

**Fig. 1.**
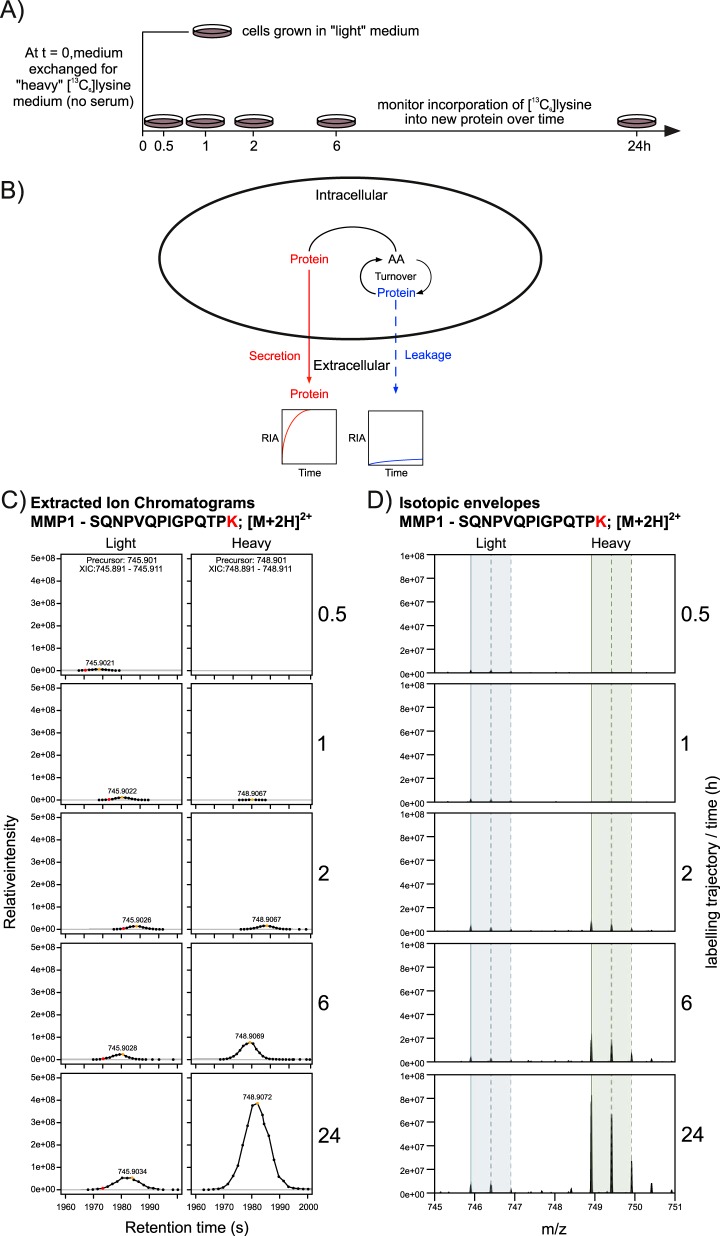
**Concept, design and analytical structure of the study.** *A*, Dishes of cells (OE21 or CAMs) were initially seeded and established in standard growth medium (DMEM) before this was exchanged for SILAC DMEM containing heavy stable isotope-labeled lysine ((^13^C_6_)lysine). Cell-conditioned SILAC DMEM was then collected at the indicated times (1 dish per time point) for up to 24 h. Protein secreted from the cells was concentrated using StrataClean and digested into peptides for subsequent analysis by LC-MSMS. *B*, Schematic diagram showing how measuring changes in the abundance of secreted protein(s) and their extent of labeling with heavy stable isotopic amino acids over time will resolve proteins that are physiologically secreted from those that leak from the cell. The rate of incorporation of (^13^C_6_)lysine into nascent proteins within the secretome was determined from the LC-MSMS data; *C*, shows representative extracted ion currents (XICs) and *D*, isotopic envelopes for the peptide SQNPVQPIGPQTPK from matrix metalloproteinase 1 (MMP1). Measuring the rate of incorporation of heavy isotope (expressed as the relative isotope abundance, or RIA), with time (RIA_t_), allows secretion dynamics to be determine for every detected protein secreted.

Cells (1 × 10^6^) were seeded in complete medium (DMEM) in five T75 flasks giving 80–90% confluency, per flask. The following day, the cell-conditioned medium on each flask was changed to fresh 37 °C heavy-labeled ((^13^C_6_)-labeled l-lysine) DMEM (10 ml volume per dish, serum-free). At the following time intervals - 30 min, 1 h, 2 h, 6 h, and 24 h - all 10 ml of now heavy-labeled cell-conditioned DMEM from each flask was collected for subsequent secretome profiling (Fig. 1*A*), as follows. Each medium/secretome preparation was centrifuged at 800 × *g* for 7 min to remove debris and the protein component within each was concentrated by mixing, with agitation, with 25 μl StrataClean resin (Agilent Technologies Ltd., Wokingham, UK). The resin beads were washed twice in 25 mm ammonium bicarbonate (ambic). Each secretome-loaded StrataClean suspension was re-suspended in 80 μl of 25 mm ambic and 5 μl of 1% (w/v) RapiGest (Waters, Hertfordshire, UK) in 25 mm ambic, prior to on-bead proteolytic digestion with trypsin (MS grade Trypsin Gold, Promega). The samples were heated at 80 °C for 10 min after which proteins were reduced, by the addition of 5 μl of 60 mm DTT at 60 °C for 10 min, before being cooled prior to addition of 5 μl of 180 mm iodoacetamide and incubation at RT for 30 min in the dark. Trypsin (1 μg) was added and the samples incubated at 37 °C overnight on a rotary mixer. Peptide digests were subsequently acidified by the addition of 1 μl of trifluoroacetic acid (TFA) and incubated at 37 °C for 45 min. Following centrifugation at 17,000 × *g* for 30 min, 10 μl of each clarified supernatant (peptide mixture) was prepared for nano LC-MS/MS. Peptide digests (2 μl) from each sample were loaded onto a trap column (Acclaim PepMap 100, 2 cm × 75 μm inner diameter, C18, 3 μm, 100 Å) at 5 μl/min with an aqueous solution containing 0.1% (v/v) TFA and 2% (v/v) acetonitrile. After 3 min, the trap column was set in-line with an analytical column (Easy-Spray PepMap® RSLC 50 cm × 75 μm inner diameter, C18, 2 μm, 100 Å) (Dionex, Sunnyvale, CA). Peptides were loaded in 0.1% (v/v) formic acid and eluted with a linear gradient of 3.8 - 40% buffer B (HPLC grade acetonitrile 80% (v/v) with 0.1% (v/v) formic acid) over 95 min at 300 nl/min, followed by a washing step (5 min at 99% solvent B) and an equilibration step (15 min at 3.8% solvent). All peptide separations were carried out using an Ultimate 3000 nano system (Dionex). The column was operated at a constant temperature of 40 °C and the LC system was coupled to a Q-Exactive mass spectrometer (Thermo Fisher), as described previously (15). The Q-Exactive was operated in data-dependent mode with survey scans acquired at a resolution of 70,000 at *m*/*z* 200. Up to the 10 most abundant peptides of charge state between 2+ and 4+ were selected for fragmentation by higher energy collisional dissociation with an isolation window of 2.0 Th and normalized collision energy of 30. The maximum ion injection times for the survey scan and the MS/MS scans were 250 and 100 ms, respectively, and the ion target value was set to 1E6 for survey scans and 1E5 for the MS/MS scans. Repetitive sequencing of peptides was minimized through dynamic exclusion of the sequenced peptides for 20 s.

Acquired MS data were searched and analyzed using Andromeda (16) and MaxQuant 1.5.8.3 (17) against a reviewed human UniProt protein database (date: 03/09/2016 containing 20,203 entries), using the default settings; briefly: the minimum required peptide length was seven amino acids long, trypsin/P was specified as the proteolytic enzyme and a single missed cleavage was allowed. Cysteine carbamidomethylation was set as a fixed modification and methionine oxidation was allowed as a variable modification. The initial precursor and fragment ion maximum mass deviations were set to 20 ppm and 0.5 Da, respectively. Peptide and protein false discovery rates (FDRs) were set to 1%, the “requant” function activated and “match between runs” enabled with the default parameters. In supplemental material (supplemental Fig. S5), we include copies of relevant figures in the main text (Figs. 3*A*/3*B*, 6*A*/6*B*, and 8*A*/8*B*) where the requant function was disabled, to demonstrate that although the total number of proteins is lower, the results and conclusions of this study are unchanged.

##### Quantification and Kinetic Analysis of Secretion

To analyze the rate of incorporation of heavy stable isotope-labeled amino acids into nascent proteins within secreted proteins, the MaxQuant peptide-level “evidence.txt” output file was analyzed in detail. Initially, peptides from known contaminant proteins as well as those generated by proteolytic mis-cleavage events (thus potentially carrying >1 labeling site) were omitted. Although only lysine-terminated peptides have the potential to carry a dynamic stable isotope label for kinetic measurements, identification of secretome proteins was based on both arginine- and lysine-terminated tryptic peptide matches. It would, of course, be possible in the future to use both labeled lysine and arginine to increase the number of kinetically informative peptides but the principles of the method we describe here would not change as a consequence. Peptide mass spectral “evidence data” for secretome proteins were then split into two lists, according to cell-line (OE21 or CAM).

For each peptide passing a 1% FDR threshold in the Andromeda search, the relative isotope abundance (RIA) was calculated at each time point if present in the MS data. RIA is expressed as abundance of heavy, labeled peptide (H), divided by the abundance of all (heavy + light) peptide (RIA_t_ = H/H+L). We applied a set of stringent criteria to produce high quality data-sets for each cell line analyzed. To model the secretome labeling trajectory, the RIA data for at least three time-points were used. We focused on peptides that had been identified and quantified, allowing RIA calculation, at more than one time point in the labeling trajectory, so that we were effectively tracking their RIA behavior over time. Moreover, because the protein content of the secretome increases with time, we only analyzed peptide mass spectral data that included RIA data at 6 h and 24 h post exchange of culture medium. A small number of peptides were rejected from further analysis if they implied labeling profiles that could not be biologically possible in this experimental system, specifically, where the calculated RIA at 30 min or 60 min after medium exchange was greater than that after 6 h or 24 h - these are likely artifacts. Because proteins at *t* = 0 are completely unlabeled and for fully labeled proteins, RIA = 1, we fitted a simplified version of the general first order equation:

RIAt=(1-exp⁡(-k.t))

which generates the optimal fitted curve for a first order rise to plateau labeling (*k*) from an initial value of 0 to a final value of 1.0. Fitting was achieved using the nls() function in R.

To assess changes in the abundance of proteins identified from the 1% FDR Andromeda search in our secretomes, we summed the mass spectral peptide intensity reported by MaxQuant for labeled (heavy) and unlabeled (light) features to obtain a quantification value. These intensity values represent the summed eXtracted Ion Current (XIC) of all isotopic clusters associated with the identified peptide sequence. If more than one peptide was identified and quantified per protein, we calculated the mean abundance of labeled (heavy) and unlabeled (light) peptide species. This was used to monitor changes in abundance of each individual protein in the secretome with time which, for a physiologically secreted protein, should increase. To calculate a measure of the flux of each protein from intracellular to extracellular pools/compartments, we first measured the abundance (P) secreted over an 18 h period by subtracting the amount secreted after 6 h from that after 24 h. We then multiplied this with the first-order rate constant (*k*) at which newly synthesized protein acquired heavy isotopic label, to give flux (flux = *k.* (P)). In order to obtain protein-level kinetic data, RIA data for peptides belonging to the same protein were grouped together and fitted using the nls() function in R. Our high-quality data-sets for both cell lines were then cross-annotated with the output from SignalP (as described in Functional analysis, below), to explore the relationship(s) between labeling kinetics and predicted sub-cellular localization. All mathematical modeling and data visualizations used R (v3.5.0) and ggplot2 (v2.0.1). Extraction and visualization of mass spectral isotopic patterns and XIC data were carried out using the “RforProteomics” package (1.15.0) (18). Abundance and kinetic plots are provided for every protein in supplemental material.

##### Functional Analysis

All protein hits from the 1% FDR Andromeda search were subsequently used for subcellular localization and GO enrichment analysis. The FASTA amino acid sequence for each protein identified in OE21 or CAM secretomes was extracted from the UniProt database and submitted to SignalP v4.1 (http://www.cbs.dtu.dk/services/SignalP/), to identify classically secreted proteins wherein a threshold SignalP d-score >0.5 defines classical secretion, as described previously (10).

UniProt accession numbers for all proteins in each secretome collected over the labeling trajectory were loaded into the R/Bioconductor package “clusterProfiler” (version 3.8.0 (19)) to allow GO over-representation analyses. We used the “enrichGO” function together with “compareCluster” to track changes in the functional enrichment profile with time, based on a hypergeometric distribution using a background list of all proteins in the *H. sapiens* annotation database. To remove redundant GO terms the “simplify” function was applied using the “Wang” measure of semantic similarity (20) (similarity cut-off of 0.5), reporting only terms with the lowest FDR-adjusted *p* values. A similar approach was taken to obtain GO functional profiles of our secretomes based on labeling kinetics.

##### Western Blot Analysis

Secretomes were probed by Western blotting for selected classically secreted proteins, namely MMP1 (antibody BAF901, R&D Systems, Oxfordshire, UK), MMP3 (antibody AF913, R&D Systems), TGFB/TGFβig-h3 (antibody AF2935, R&D Systems) and SCG2 (antibody ab96589, Abcam, Cambridge, UK). In some experiments, the cells were pre-incubated for 30 min with 10 μg.ml^−1^ brefeldin A (BFA; eBioscience, Ltd., Hatfield, UK), or 10 μg.ml^−1^ cycloheximide (Sigma, Dorset, UK) and/or 1 μm ionomycin (Sigma). Proteins were resolved by SDS-PAGE and processed for Western blotting as described previously (13).

## RESULTS

### 

#### 

##### Experimental Strategy

Secretomes, particulary in the early periods of incubation, are low abundance and we concentrated proteins by adsorption onto StrataClean, a silica-based bead preparation with a high affinity for protein. This captures all secretome proteins, and tryptic digests can be conducted directly on the beads—SDS-PAGE of bead eluate after digestion confirms completeness of digestion (results not shown). Peptides recovered from the on-bead digests were then used directly for LC-MS/MS. To establish the linearity of the StrataClean bead capture, we completed control experiments in which fresh “virgin” (v) culture media was mixed in different proportions (specifically, 0:100, 20:80, 50:50, 80:20 and 100:0) with media that had been cell-conditioned (cc) for 24 h with CAM cells (supplemental Fig. S1, supplemental Table S1). StrataClean was used exactly as described under Experimental Procedures. The label free abundance of recovered proteins exhibited excellent linearity with protein load, confirming the quantitative performance of the protein capture method (supplemental Fig. S1, supplemental Table S1). Further, the assessment of the degree of labeling of protein captured by StrataClean is internally controlled and thus independent of the quantity of protein.

##### Kinetic Parameters for Secretome Proteins: Abundance and Flux

Each protein in a secretome has two kinetic parameters of relevance. The first is the change in abundance as a function of time. A protein that is actively secreted should accumulate in the medium, unless there is an opposing removal process that takes the protein back into the cell or which elicits extracellular degradation (a possibility, given the number of endopeptidases that are secreted from cells). Thus, abundance is not enough to define secretome kinetics. The second necessary parameter is flux, or the rate at which the protein flows from the intracellular pool to the extracellular space. Measurement of flux can also discriminate between classically secreted proteins and those that are released from the cell through necrotic or apoptotic changes, provided a labeling method is used to discriminate these pools. If a cell is supplied with labeled precursors, such as amino acids, the newly synthesized and labeled *intracellular* proteins will enter and equilibrate with the unlabeled pre-existing pool. Thus, although newly synthesized proteins are fully labeled, they are diluted by a large, pre-existing pool of unlabeled protein, and thus the RIA is low. Subsequent leakage from the cell would reflect loss of this minimally-labeled mixture. If a small proportion of this pool is then released from the cell, the fraction of protein molecules that are labeled will be low. By contrast, proteins that are *classically secreted by the constitutive pathway* do not have a large intracellular reservoir to dilute the labeling of newly synthesized molecules, so all labeled proteins exiting the endoplasmic reticulum/Golgi are immediately secreted from the cell and will exhibit rapid acquisition of complete labeling in the medium. A third class of proteins, those of the *regulated secretory pathway* which is a feature of neural, exocrine, and endocrine cells, can have a large, stored intracellular pool in secretory vesicles, and thus, newly synthesized protein should enter this pool and may exhibit relatively slow labeling. We reasoned that these different kinetic behaviors could be used to discriminate between classically secreted proteins and those derived from intracellular protein leakage.

Tensioned against protein turnover is the change in protein abundance. Secretome protein pools that expand would be expected to exhibit a rapid increase in label enrichment, consistent with a small intracellular pool and physiological secretion. By contrast, continued leakage of an intracellular protein, *e.g.* through cell damage, will reflect the extent of labeling of the intracellular pool, and unless this is an intrinsically high turnover protein, the degree of labeling will be low. To develop this logic further, a secreted protein pool that is static (not increasing) but which has a high degree of labeling must be subject to rapid removal from the extracellular pool (Fig. 1*B*). It follows that two measurements, changes in secretome protein abundance and the kinetic profile of labeling, could resolve proteins that are physiologically secreted from those that leak from the cell.

##### Rapid Labeling and Secretion in Both Cancer and Stromal Cells of a Classically Secreted Protein but Not an Intracellular Protein

**Fig. 2.**
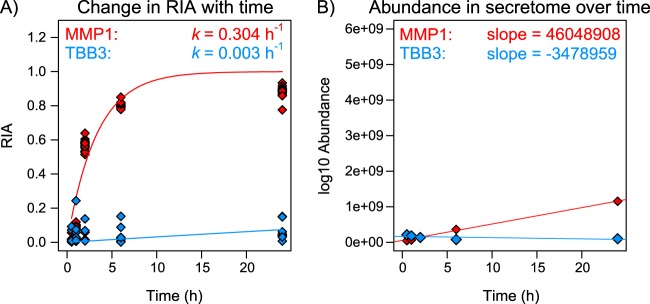
**Representative dynamic behavior of a classically secreted protein (MMP1) and an intracellular protein (TBB3).** *A*, Secreted proteins acquire heavy label quickly because there is no intracellular pool to dilute/delay acquisition of label. This is exemplified by the rapid rise toward RIA of 1, as shown by MMP1 (red line). *B*, The abundance of a secreted protein should increase continuously with time, as also demonstrated by MMP1 (red line). Opposing behaviors can be seen for the intracellular protein TBB3 (*A*, *B*, blue lines).

In this study, a cancer (OE21) cell and a stromal cell (CAM) were labeled over 24 h with a stable isotope-labeled amino acid in the medium and the size of the secreted pool was assessed by summing the abundance of labeled (heavy, H) and unlabeled (light, L) peptide mass spectral features over time (0.5 to 24 h; Fig. 1*C*, 1*D*). Concurrently, the extent of labeling was assessed by monitoring changes in the relative isotope abundance (RIA, expressed as H/(H+L)) of these features, with time. Both cell types were monitored over time for incorporation of (^13^C_6_)lysine, and for protein abundance. To illustrate the concept: the peptide SQNPVQPIGPQTP***K*** from an established physiologically secreted protein, matrix metalloproteinase 1 (MMP1), could be detected in unlabeled form 30 min after incubating CAMs in (^13^C_6_)lysine-containing culture medium but, at this time, no labeled peptide was present (Fig. 1*C*, 1*D*). The unlabeled material defines MMP1 that was pre-synthesized prior to medium exchange and equilibration of the extracellular labeled amino acid with the intracellular tRNA pool. Over time, there was a progressive increase in labeled MMP1, highlighted by the shift in the relative peak heights of either XICs (Fig. 1*C*) or peptide precursor ion isotopic envelopes (Fig. 1*D*), as well as in change in RIA with time (RIA_t_, Fig. 2*A*, red line). However, intracellular proteins, of which tubulin beta 3 (TBB3) is a representative example, incorporated virtually no label during the same period (Fig. 2*A*, blue line). Moreover, a comparison of the quantified abundance (summing both labeled and unlabeled peptides to obtain the total pool) revealed that although the output of MMP1 increased steadily over the period of the experiment, the abundance of TBB3 remained relatively static or even declined (Fig. 2*B*).

##### Secretome Constituents Meeting the Criteria for Kinetic Analysis

We established a dataset of secretome proteins upon which a rigorous analysis of secretory kinetics might be made. After removing peptide spectrum matches (PSMs) from known contaminant proteins (included in the MaxQuant installation by default), we had a dataset with 92,480 PSMs: 48,933 in OE21 secretome samples corresponding to 2109 unique proteins and 43,547 in CAM samples corresponding to 1815 unique proteins. Approximately 9% of total PSMs (8,274) were generated by proteolytic mis-cleavage events and although those containing lysine residues could also report on labeling extent, they were excluded in this analysis. Arginine-terminating PSMs accounted for ∼40% of the total (OE21, 19,243; CAM, 17,566) and were used for protein identification purposes but not for measurement of label incorporation. The remaining 55,671 PSMs (OE21, 29,690; CAM, 25,981) were lysine-terminated with a single instance of this amino acid; these mapped to 1751 (OE21) and 1484 (CAM) unique proteins. Further application of the stringent filtering criteria described in the experimental procedures section yielded 910 individual proteins from OE21 cells (RIA_t_ data generated from 13,585 PSMs) and 549 proteins from CAMs (RIA_t_ data generated from 11,313 PSMs). Pre- and postfiltered RIA_t_ data sets can be found in supplemental Table S2.

##### Classically Secreted Proteins Exhibit Distinct Secretory Kinetics

**Fig. 3.**
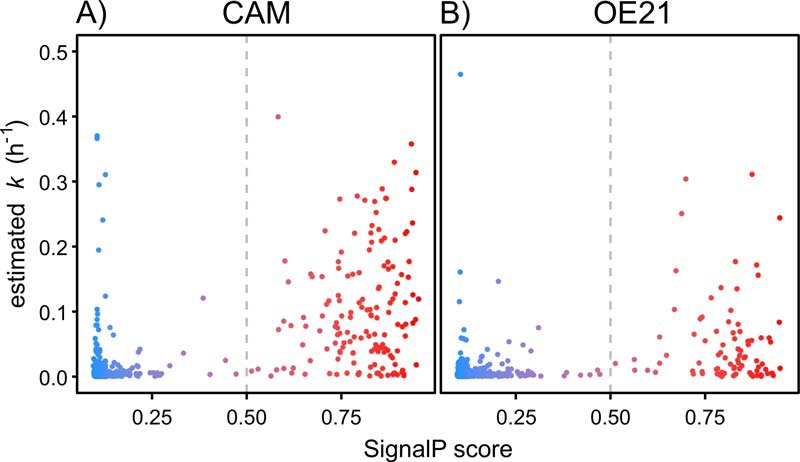
**Rates of label exchange for individual proteins discriminated based on SignalP score.** The first order rate constant (*k*) of incorporation of (^13^C_6_)lysine into nascent proteins within the CAM (*A*) or OE21 (*B*) secretome was plotted as a function of the SignalP score. Symbols are color-coded based on SignalP score, from 0 = dark blue, through to the maximum score of 1 = red. SIDLS-determined predictions of secretion on the basis of rate (*k*) of light-to-heavy label incorporation closely match those predicted by SignalP, with the vast majority of proteins showing high rates of label exchange (>0.05) also having high SignalP scores, typically >0.5. An equivalent plot with the MaxQuant requant function disabled is provided in supplemental material.

For each protein in the reduced and filtered data set, the labeling vector was measured as a first order rate constant (*k*) defining the rise to plateau labeling and, if appropriate, exchange into a pre-existing, unlabeled intracellular pool. Moreover, the quantified abundance (summing both labeled and unlabeled peptides to obtain the total pool) was used to determine the rate of pool expansion. These two parameters were then used to discriminate classically secreted proteins from intracellular proteins that were externalized by leakage. Complete labeling trajectory and abundance curves for individual proteins from both CAMs and OE21 cells are provided in the supplemental material (supplemental Fig. S2 (CAM) and supplemental Fig. S3 (OE21); red plots—labeling kinetics; blue plots—abundance). Many proteins were rapidly labeled and after a short period the RIA_t_ reached unity. By contrast, other proteins were barely labeled in the same time frame. Further exploration of the data indicated that well known classically secreted proteins (*e.g.* MMP1, MMP2, MMP3, TGFβig-h3) incorporated (^13^C_6_)lysine rapidly whereas known intracellular proteins (*e.g.* BAG3, COF1, SYSC, TBB5) were labeled minimally. In addition, the abundance in the cell medium of classically secreted proteins increased whereas that of intracellular proteins remained either constant or declined (for abundance data-sets for both CAM and OE21 cells, see the blue plots in supplemental Fig. S2 and supplemental Fig. S3, respectively). These observations therefore support the hypothesis that the rate of labeling of a protein (*k*) is a reliable indicator of physiological secretion, *i.e.* proteins destined for secretion by virtue of an N-terminal signal sequence and with no large intracellular pool to dampen the incorporation of label. High *k* values are associated with those proteins that were operationally defined as secreted, whether based on direct knowledge or through bioinformatically-derived (SignalP) prediction (Fig. 3*A* and 3*B*, see also supplemental Fig. S5*A* and S5*B* with requant disabled). The SignalP d-cutoff score is a combined value from both signal-peptide and cleavage site prediction networks. The default thresholds resulting in a positive prediction of a signal peptide are 0.5 or 0.45 for eukaryotic proteins with or without a transmembrane domain, respectively (10). Based on our biology-driven SIDLS approach, these thresholds appear to be accurate (see gray dashed lines in Fig. 3*A*, CAMs; and 3*B*, OE21 cells).

##### Matching Secretory Kinetics and SignalP Scores

**Fig. 4.**
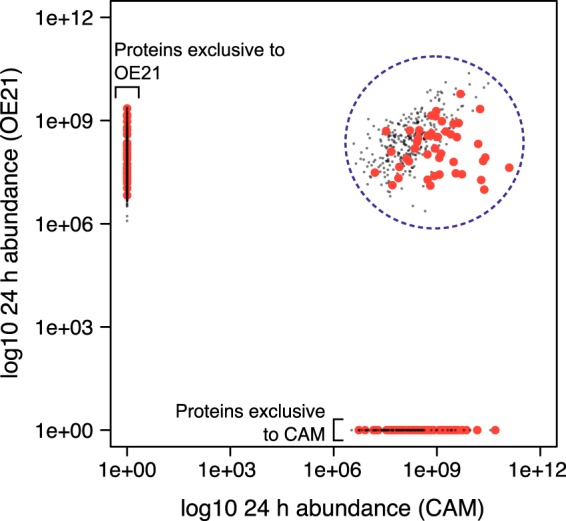
**Classification of proteins on the basis of secretome behavior.** Monitoring the abundance of each protein in the 24 h secretomes of the two cell types (OE21 and CAM) allows the identification of six different protein populations; classically secreted (SignalP score >0.5, large red points) or not (SignalP score <0.5, small gray points), and either common to the two cells (blue dashed circled) or exclusive to OE21 or to CAMs, as highlighted on the plot.

**Fig. 5.**
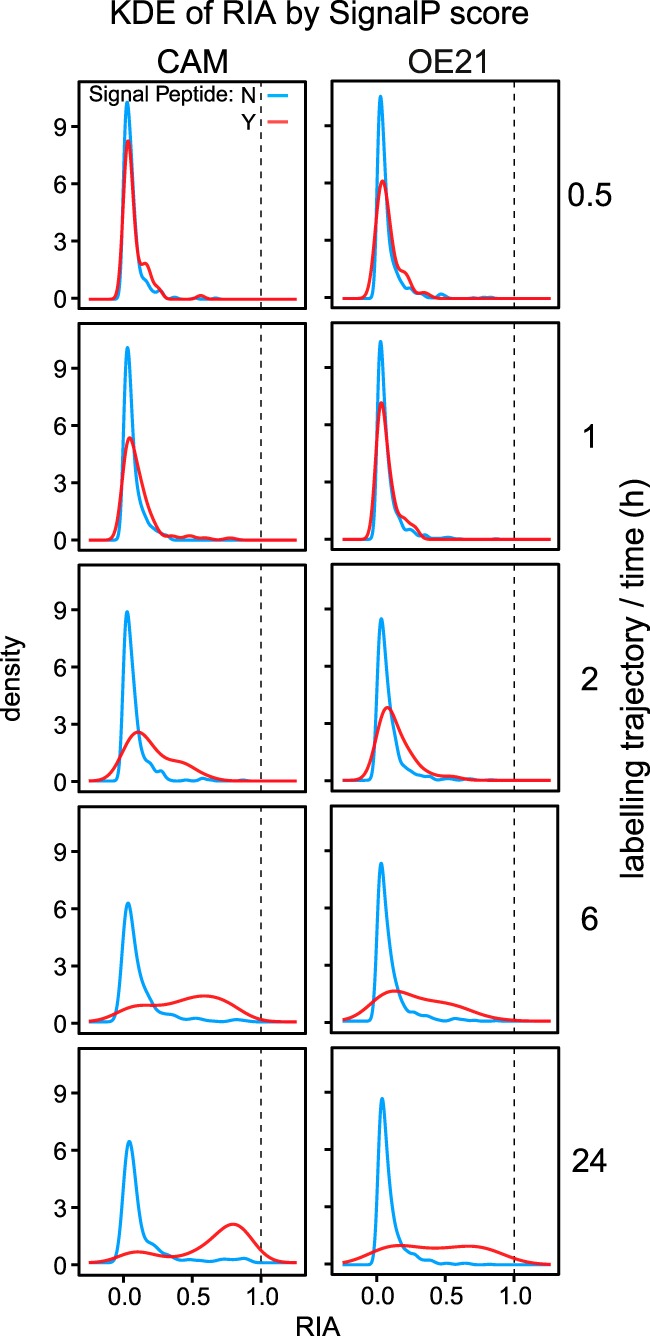
**Time-dependent changes in peptide relative isotope abundance (RIA).** For all high quality (^13^C_6_)lysine-labeled peptides at the indicated times, the RIA, expressed as the ratio H/(H+L) was calculated and plotted as a distribution curve using kernel density estimation. For proteins that do not contain a signal peptide (SignalP <0.5), peptides remain almost entirely unlabeled over the total trajectory of the study (blue lines). Peptides from secreted proteins (SignalP >0.5), however, demonstrate a clear transition from largely unlabeled to extensively labeled as a consequence of protein turnover/label exchange. This is especially true in the stromal CAM cell line (left hand panels), but is also evident although less pronounced in cancer (OE21) cells. The dotted lines define the maximum possible RIA that the system can attain.

**Fig. 6.**
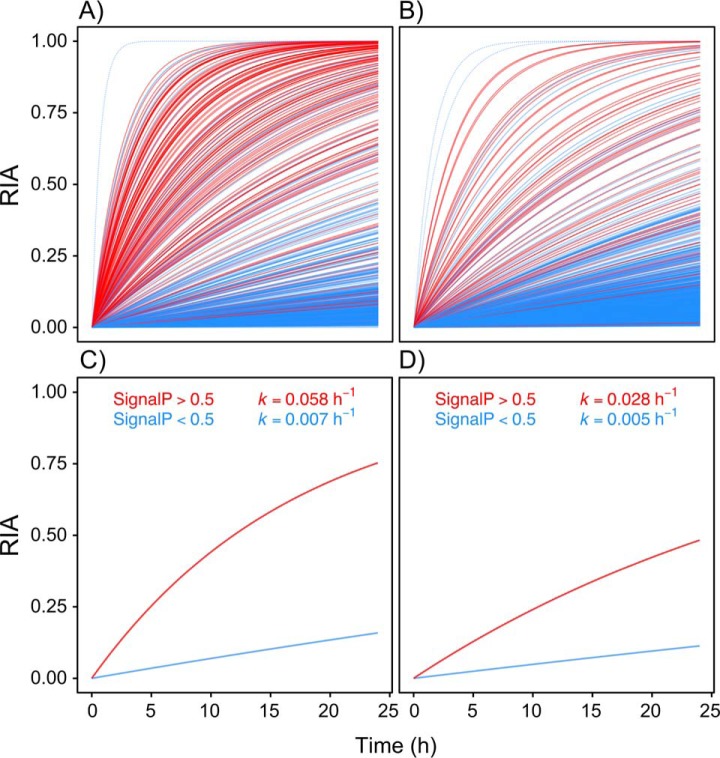
**Rates of label exchange for individual proteins and complete secretomes, discriminated based on SignalP scores.** First-order rate constants at which newly synthesized proteins acquire heavy isotopic label, for every protein in the CAM (*A*) and OE21 (*B*) secretomes. Physiologically-secreted proteins (SignalP >0.5; red lines) clearly acquire new (^13^C_6_)lysine at a higher rate compared with intracellular proteins that merely “leak” from the cell (SignalP <0.5; blue lines). Some proteins with low SignalP scores appear to be readily secreted or have very high turnover (blue dashed lines); however, manual inspection of the raw MS data for these proteins revealed them to be artifacts (see main text for explanation). Relating “global” secretome kinetics to protein classification on the basis of a computational prediction of the presence or not of a signal peptide (SignalP score), reveals an impressive discrimination in CAMs (*C*) and, to a lesser extent, in cancer cells (Panel *D*), thus raising questions about the secretome behavior of cells *in vivo* in the tumor microenvironment. An equivalent plot with the MaxQuant requant function disabled is provided in supplemental material.

To relate secretome kinetics to a protein classification we divided the secretome data into proteins with a SignalP score of >0.5 (classical secretory proteins) compared with those without (<0.5). From the secretomes of the two cell types it was readily possible to identify six different protein populations, namely classically secreted or not, those that were common to the two cells or those exclusive to OE21 or CAMs (Fig. 4). After 6 h of labeling, the RIA of CAM secretome proteins with SignalP >0.5 was clearly distinguishable from those with <0.5. This was also evident although less pronounced in OE21 cells at 24 h (Fig. 5). Rates of label exchange differed for individual proteins (Fig. 6*A*, CAMs; and Fig. 6*B*, OE21 cells) but the discrimination based on SignalP score was nonetheless impressive. There are proteins, however, that appear to acquire label very quickly, but which are not classically secreted proteins (*i.e.* with SignalP scores <0.5; for *e.g.* see dashed lines in Fig. 6*A* and 6*B*, see also supplemental Fig. S5*C* and S5*D* with requant disabled). These initially raised concerns to us but their RIA_t_ data are suspicious in that they show an instant rise to plateau without any subsequent increase over time. Detailed inspection of the peptide-level chromatography and mass spectrometry data for these proteins revealed them to be erroneous measurements derived from mis-assignment of heavy/light peptide features by MaxQuant. In fact, in all these cases, there was no tandem MS evidence of *both* light and heavy features, and we believe these are anomalous data-points. The association between SignalP >0.5 and *k* >0.02/h (combined *k* for SignalP >0.5 in OE21, Fig. 6*B*, red line) was highly significant for both CAMs and OE21 cells (Fisher exact text, *p* < 0.001 in both cases). Combined fits of all proteins, in each cell type, sub-classified based on SignalP score (> or <0.5), indicated a >10-fold difference in the rate of label exchange (*k*) in CAMs (Fig. 6*C*), and an ∼5-fold difference in OE21 cells (Fig. 6*D*).

A cut-off of *k* ≥ 0.1/h for the kinetics of a classically secreted protein was initially selected based on an empirical assessment of the data. Indeed, in support of this, inspection of Fig. 6*C* (red line) shows the predicted *k* for all proteins with SignalP >0.5 in CAMs was quite close to this cut off, *i.e.* 0.058/h. However, the selection of the cut-off in *k* to differentiate classically secreted proteins may vary for different cell types depending on their biology. For example, there were lower rates of label exchange in the cancer cell line (OE21; Fig. 6*D*). Alternative approaches to defining the cut-off may prove useful in comparative studies between cell types (21). Taking a combined fit value of each secretome sub-classified by SignalP > or <0.5, which is valid also based on its relationship to *k* (Fig. 3), identified a distinct group of 108/549 (CAMs) and 57/910 (OE21) proteins that are “true” physiologically secreted proteins.

##### Validation of the Kinetics of Classically Secreted Proteins

**Fig. 7.**
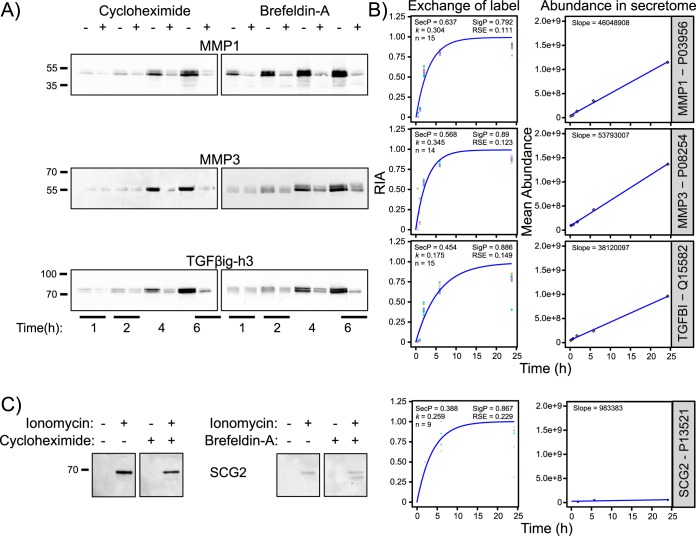
**Validation of the kinetics of classically secreted proteins by Western blotting.** *A*, The kinetic behavior of three representative classically secreted proteins, MMP1, MMP3 and TGFBI/TGFβig-h3, following pharmacological blockade of secretion in CAMs, were validated using a standard immunoblotting approach. When translation was inhibited with cycloheximide, the accumulation in the medium of MMP1 and TGFβig-h3 was already reduced at 1 h. For these two proteins (and MMP2), secretion was clearly inhibited at 4 h. Similarly, upon perturbation of secretion using brefeldin A (BFA), the appearance of MMP1 was suppressed at 1 h and of all three proteins from 2 h. *B*, The kinetics of secretion of MMP1, MMP3 and TGFβig-h3 determined by SIDLS clearly match those determined by the orthogonal approach of Western blotting. *C*, Evidence that SIDLS can discern proteins of the regulated secretory pathway. Western blotting confirmation that secretion of secretogranin-2 (SCG2), an established marker of a minor, endocrine-like secretory phenotype known to be present in stromal myofibroblasts (CAMs), was stimulated by a short (30 min) stimulation with ionomycin. This is consistent with calcium-evoked exocytosis and the response was resistant to cycloheximide (left) and BFA (right), supporting the idea that this represents release from intracellular storage vesicles.

To determine whether the SIDLS kinetic profiles were compatible with those derived by other methods, we used orthogonal analysis by Western blots to define the kinetic responses of three representative classically secreted proteins, MMP1, MMP3 and transforming growth factor-beta-induced protein ig-h3 (TGFBI/TGFβig-h3), following pharmacological treatments that arrest protein secretion. When translation was inhibited in CAMs with cycloheximide, the accumulation in the medium of MMP1 and TGFβig-h3 was already inhibited at 1 h and for these two as well as MMP2 was very clearly inhibited at 4 h (Fig. 7*A*, left). Similarly, using the ER-to-Golgi transport inhibitor, brefeldin A (BFA), to block progression along the secretory pathway, the appearance of MMP1 was suppressed at 1 h and for all three proteins at 2 h (Fig. 7*A*, right). The rate of appearance and abundance of all three proteins in the secretome measured using immunoblotting closely mirrors that determined by SIDLS (Fig. 7*B*).

##### Application of SIDLS to Proteins Exhibiting Regulated Secretion

Constitutively secreted proteins predominate in the secretomes of the two cells studied here. However, stromal myofibroblasts including CAMs may exhibit a modest regulated secretory pathway (22) and in this context it is interesting that a marker of the pathway, secretogranin-2 (SCG2), was identified and exhibited rapid label incorporation (*k* = 0.274/h). Immunoblotting confirmed that secretion of SCG2 was stimulated by a short (30 min) stimulation with ionomycin, consistent with calcium-evoked exocytosis and the response was resistant to cycloheximide and BFA, consistent with release from storage vesicles (Fig. 7*C*). Thus, SIDLS can also be applicable to proteins of the regulated secretory pathway. The kinetics of labeling of these proteins in the medium may be slower than for constitutively secreted proteins although this may be offset to a substantial extent for those regulated secretory proteins that exhibit preferential secretion of newly synthesized material (23, 24).

##### Meta-analysis of Classically Secreted Proteins in the CAM Secretome

**Fig. 8.**
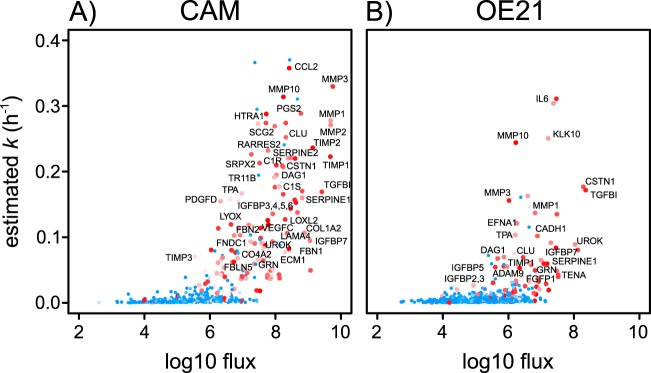
**Relationship between *k* and flux of protein from intracellular to extracellular pools.** A surrogate measurement of flux into the extracellular protein pool was determined by calculating, flux = *k.* (P), where *k* is first-order rate constant at which newly synthesized protein acquired heavy isotopic label and P is the amount of protein secreted over an 18 h period. Symbols are color-coded on the basis of SignalP classification (>0.5 = red; <0.5 = blue) with alpha transparency shading according to the score from SignalP predictions (from 0, high transparency - to 1, low transparency). *A*, CAMs; *B*, OE21 cells. Selected proteins of interest in tumor biology are highlighted. An equivalent plot with the MaxQuant requant function disabled is provided in supplemental material.

Classically secreted proteins included representatives of several important classes of secreted protein, notably extracellular matrix (ECM) proteins such as TGFB/TGFβig-h3, proteases (*e.g.* MMP1, MMP3, C1R), protease inhibitors (*e.g.* TIMP1, TIMP2, SERPINE1, SERPINE2), chemokines (*e.g.* RARRES2), cytokines (*e.g.* the growth factors, VEGFC and CTGF) and growth factor-associated proteins (*e.g.* IGFBP3, -4, -5, -6), some of which have already been characterized in previous studies of myofibroblast secretomes (14, 22, 25). When flux is plotted against estimated *k*, this subset is readily distinguishable (Fig. 8, selected examples are highlighted, see also supplemental Fig. S5*E* and S5*F* with requant disabled).

**Fig. 9.**
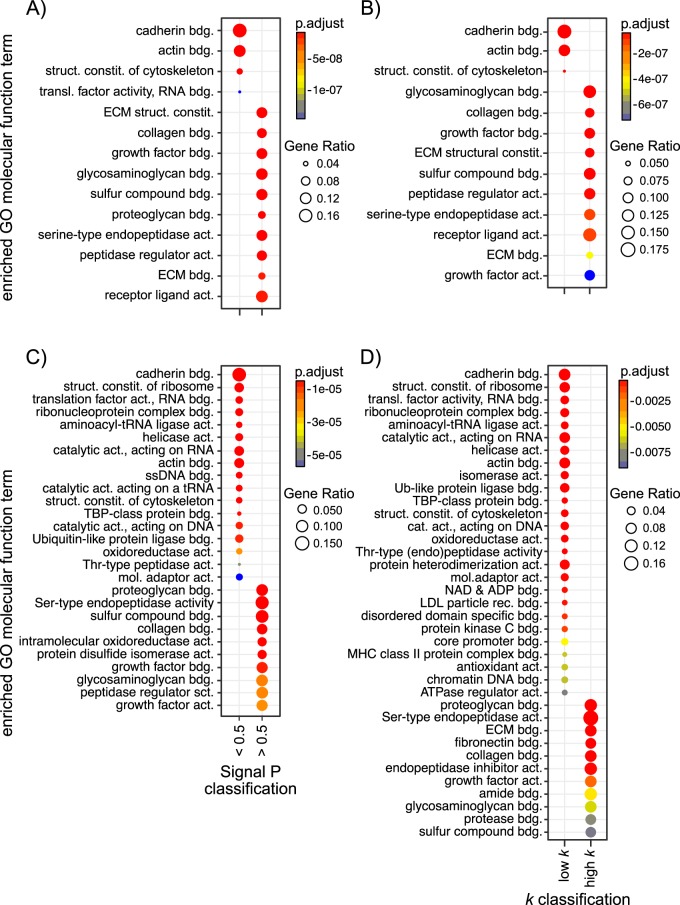
**Functional analysis of the secretome.** GO enrichment analysis was performed using the “compareCluster” function in the R/Bioconductor package “clusterProfiler”. All proteins identified in the CAM (Panel *A*, *B*) or OE21 (*C*, *D*) secretomes were loaded simultaneously in clusters based upon either their classification according to SignalP (d-score < or >0.5, *A*, *C*) or their classification according to SIDLS (“high *k*” *versus* “low *k*”, *B*, *D*) and statistically over-represented GOMF terms of each protein set determined (hypergeometric test pvalueCutoff, *p* = 0.000001 for CAM, *p* = 0.01 for OE21). To aid visualization and interpretation of the data, redundant GO terms were removed using the “simplify” function (as described under Experimental Procedures) and the most over-represented GOMF terms plotted as dotplots. GO terms linked with binding to multiple ECM components are over-represented in the secreted protein clusters (SigP >0.5 and “high *k*”). Non secreted proteins, however, showed an enrichment with terms associated primarily with translation and cell structure (SigP <0.5 and “low *k*”). Abbreviations: bdg. = binding; struct. = structural; constit. = constituent; act. = activity; transl. = translation; dep. = dependent; mol. = molecular.

Using a SignalP d-score < or >0.5, in isolation, as a classifier of secretion (Fig. 9*A* (CAM) and Fig. 9*C* (OE21)), GO over-representation analysis revealed molecular function (GOMF) terms linked with binding to multiple ECM components for secreted proteins (SigP >0.5). Nonsecreted proteins, however, showed an enrichment with terms associated primarily with translation and cell structure, consistent with the idea that these are simply “leaked” proteins that function in these intracellular processes. Using our data-driven, SIDLS-determined classification of the secretome, in combination with SignalP (see Fig. 6*C* and 6*D*) to separate the secretome into a “high *k*” group (*k* > combined fit for SignalP >0.5 proteins) and a “low *k*” group (*k* < combined fit for SignalP >0.5 proteins) reveals an almost identical pattern of GOMF terms (Fig. 9*B* (CAM) and 9*D* (OE21)), adding further confidence to our SIDLS-mediated approach to secretome classification.

## DISCUSSION

We describe a dynamic stable isotopic labeling- and mass spectrometry-based approach to characterize the physiological secretome of any cell that can be maintained in culture. Unlike traditional SILAC approaches, our stable isotope dynamic labeling of secretomes (SIDLS) method exploits the kinetics of exchange from light-to-heavy stable isotopic labeling that occurs with protein synthesis *de novo* over relatively short labeling trajectories, thus dispensing with the need for exogenously-added serum factors required in more long-term cell cultures. This is beneficial as serum often contains an abundance of factors that influence the behavior and physiology of the culture system. SIDLS can confidently discriminate the secretion kinetics of physiologically relevant classical secretory proteins from intracellular proteins that are released from cells either through damage during cell culture, apoptosis or “leakage.” It is therefore a powerful classifier of the different cellular origins of proteins within the secretome and should be broadly applicable, allowing secretome characterization of nonproliferating cells and cells only viable in short term culture. Embedding new knowledge of the rate of synthesis of the secretome constituents improves upon previously described approaches that rely on either the time-consuming labeling of cells to completion with heavy isotopic labels (traditional SILAC approaches), complex click chemistry approaches, or a combination of the two (12, 26–29).

Across two different cell lines we obtained global secretome identification of over 2000 proteins and, in parallel, definitively determine the dynamic secretome behavior of a large proportion of these proteins, helping define their true intracellular origin and physiological role (see supplemental Tables S2 and S3 for complete data sets). As expected, classical secreted proteins show a shift in RIA from 0 toward 1 over time (as exemplified by MMP1 in Fig. 2*A*, red line). This is especially true in the nontransformed cells (CAMs), taking the SignalP score as a classifier of secretion or not (Fig. 5*A*). Our dynamic labeling strategy, combined with assessment of total abundance in the secretome, allowed us to distinguish, with high confidence, secreted protein assignments from erroneous measurements borne out of chromatographic and MS errors (*e.g.* co-eluting peptide MS isotopic envelopes skewing RIA calculations), as logic dictates that the total abundance of any secreted protein in the secretome must increase with time. Several proteins that appear to be secreted readily, but which have no known extracellular function, fall into this bracket (*e.g.* RRP12 in OE21 cells; GRHL1 and AL9A1 in CAMs; CHMP3 in both OE21 cells and CAMs).

In our data set, some classically secreted proteins show near identical behavior across both cell-lines (see supplemental Fig. S4). Although small, this list of proteins identified in both CAM and OE21 secretomes shows that in general, a commonality exists in the secretome behavior of proteins between stromal and cancer cells that exist within the same microenvironment. But several proteins were removed from our data-sets during stringent filtering of the RIA_t_ data. Indeed, some other classical secreted proteins, for *e.g.* additional members of the MMP family, did not meet our stringent filtering criteria - for example, where tandem MS evidence existed for both light and heavy peptide features, but only at one time point in the labeling trajectory (all protein data is included in the raw data in supplemental Tables S2 and S3). More in-depth proteomic analyses, for *e.g.* adopting fractionation approaches of each secretome sample, would increase the number of proteins identified allowing improved cross-comparison(s) to be made. However, it must be noted that, in general, the relationship between the rate of labeling in OE21 cancer cells and rate of labeling in CAMs, for common proteins, is not a strong correlation. Much clearer is a generally lower rate of incorporation of label into newly synthesized protein in the cancer cells (OE21), indicative of defective protein synthesis and/or trafficking though the secretory system in cancer.

One of the main advantages of SIDLS is that it provides an orthogonal perspective to secretome dynamics. By tracking the appearance of label in secreted proteins, it is possible to build a profile of the speed and duration of response of individual proteins and resolve true secreted proteins from low level intracellular leakage. The marked consonance between proteins that would be labeled as secreted through a high predictive score of a signal peptide and rapid labeling gives a convincing confirmatory perspective on the secretome. For this analysis, we have been very stringent in the retention of proteins, and those for which abundances were too low for recovery by data-dependent acquisition approaches would be recovered by more targeted methods, such as selected reaction monitoring (30). It is not too bold to imagine that the use of different labeled precursors in a pulse-labeling strategy would provide new insights into the interaction of co-cultures of cells mediated by their secretomes. Thus, inclusion of the simple expedient of dynamic labeling of secretomes will greatly increase the confidence with which such secretomes are studied.

## DATA AVAILABILITY

The mass spectrometry proteomics data for the SIDLS dynamic labeling aspect of this study have been deposited to the ProteomeXchange Consortium via the PRIDE (31) partner repository with the data set identifier PXD007231. Equivalent data for the linearity of protein capture by StrataClean are deposited to ProteomeXchange too, with the identifier PXD009838. The output of the MaxQuant searches of the SIDLS dynamic labeling proteomics data are available using MS-Viewer (http://msviewer.ucsf.edu/prospector/cgi-bin/msform.cgi?form=msviewer), using the search key identifier: 88dyh3qzvc. All annotated spectra can be accessed here. That for the StrataClean linearity experiment is also available using MS-Viewer using the search key idenitifier: hjhr9jxzpk.

## Supplementary Material

supplemental material

## Footnotes

^1^ The abbreviations used are:

GO: gene ontology
SIDLS: stable isotope dynamic labeling of secretomes
CAMs: cancer-associated myofibroblasts.
