# Supplementary material for: Stable Isotope Dynamic Labeling of Secretomes (SIDLS) Identifies Authentic Secretory Proteins Released by Cancer and Stromal Cells
Source: Mol Cell Proteomics. 2018 Jun 18;17(9):1837–49. doi: 10.1074/mcp.TIR117.000516 (PMC6126392; doi:10.1074/mcp.TIR117.000516)
Supplement: supplemental material [file TIR117.000516_index.html]

Supplement to Stable isotope dynamic labeling of secretomes (SIDLS) identifies authentic secretory proteins released by cancer and stromal cells | Molecular & Cellular Proteomics

## Supplemental Data

- Supplemental Fig. 1 - Supplemental Data (to be published online) - "Stable isotope dynamic labeling of secretomes (SIDLS) identifies authentic secretory proteins released by cancer and stromal cells"
- Supplemental Fig. 2 - Supplemental Data (to be published online) - "Stable isotope dynamic labeling of secretomes (SIDLS) identifies authentic secretory proteins released by cancer and stromal cells"
- Supplemental Fig. 3 - Supplemental Data (to be published online) - "Stable isotope dynamic labeling of secretomes (SIDLS) identifies authentic secretory proteins released by cancer and stromal cells"
- Supplemental Fig. 4 - Supplemental Data (to be published online) - "Stable isotope dynamic labeling of secretomes (SIDLS) identifies authentic secretory proteins released by cancer and stromal cells"
- Supplemental Fig. 5 - Supplemental Data (to be published online) - "Stable isotope dynamic labeling of secretomes (SIDLS) identifies authentic secretory proteins released by cancer and stromal cells"
- Supplemental Table 1 - Supplemental Data (to be published online) - "Stable isotope dynamic labeling of secretomes (SIDLS) identifies authentic secretory proteins released by cancer and stromal cells"
- Supplemental Table 2 - Supplemental Data (to be published online) - "Stable isotope dynamic labeling of secretomes (SIDLS) identifies authentic secretory proteins released by cancer and stromal cells"
- Supplemental Table 3 - Supplemental Data (to be published online) - "Stable isotope dynamic labeling of secretomes (SIDLS) identifies authentic secretory proteins released by cancer and stromal cells"
- Summary of Supplemental Files - Document explaining the content of Supplemental Material
