# Supplementary material for: Stable Isotope Dynamic Labeling of Secretomes (SIDLS) Identifies Authentic Secretory Proteins Released by Cancer and Stromal Cells
Source: Mol Cell Proteomics. 2018 Jun 18;17(9):1837–49. doi: 10.1074/mcp.TIR117.000516 (PMC6126392; doi:10.1074/mcp.TIR117.000516)
Supplement: supplemental material [file 134290_3_supp_155567_pwwwgp.pdf]

Supplemental Figure 1. The StrataClean approach is a very efficient method of capturing protein from dilute proteomes. A serial dilution experiment was performed to confirm the linearity of capture of proteins from secretome samples using StrataClean. StrataClean was to capture secreted protein from mixtures of ‘cell-conditioned’ (cc) media and ‘virgin’ (v) media in the ratios 100:0, 80:20, 50:50, 20:80 and 0:100. Each was then proteolytically digested on-bead as described in the experimental procedures section, prior to LC-MSMS and analysis using MaxQuant. For 622 proteins across all samples (except 0:100), the total signal intensity was measured and linear regression performed to investigate the relationship between it and protein input (conditioned:virgin media ratio, denoted cc:v mix). The R2 of all 622 correlations are plotted as a distribution curve using kernel density estimation confirming the approach to be very efficient at capturing dilute proteomes (see subsequent pages for protein-by-protein linear regression plots).

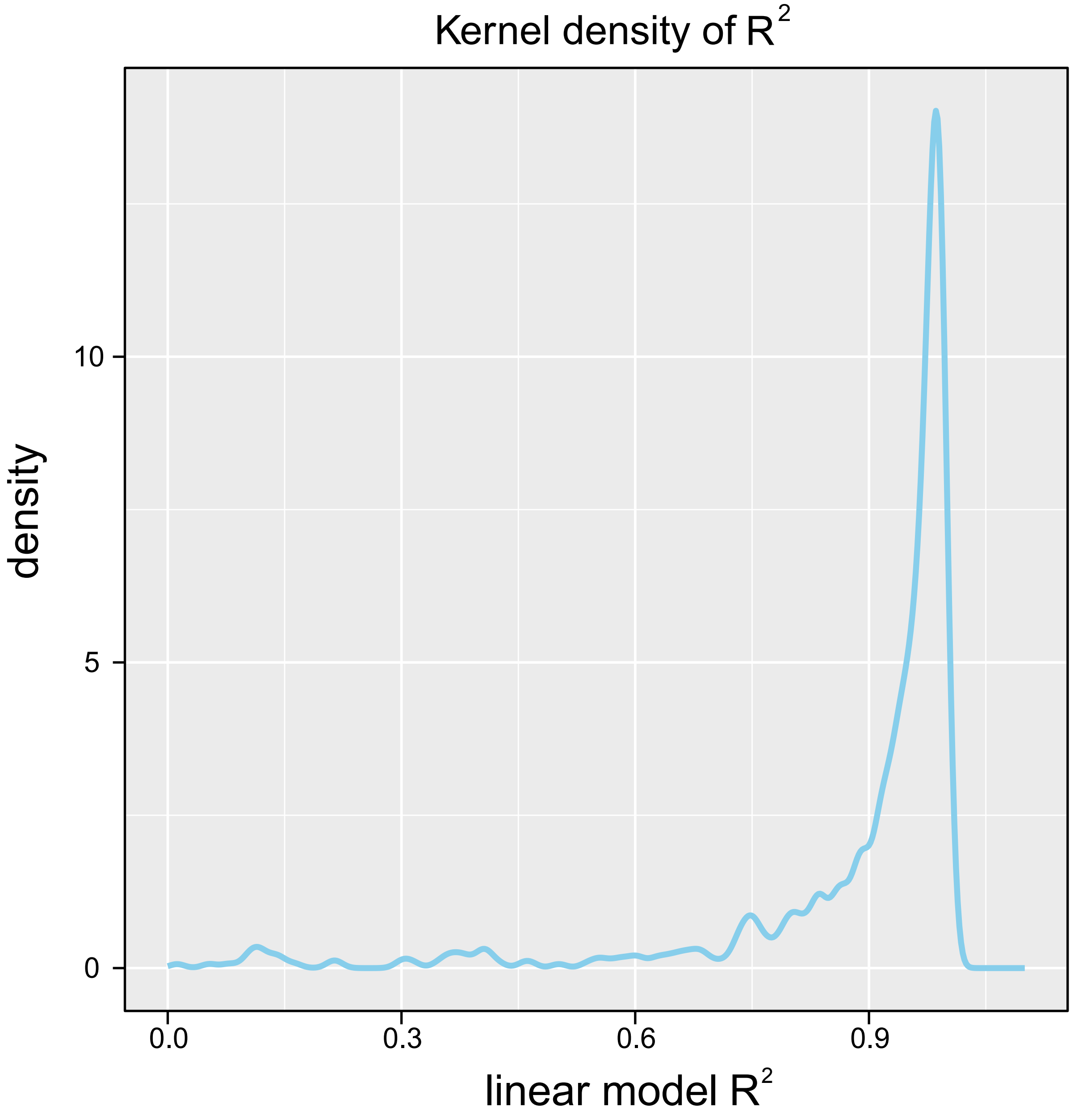

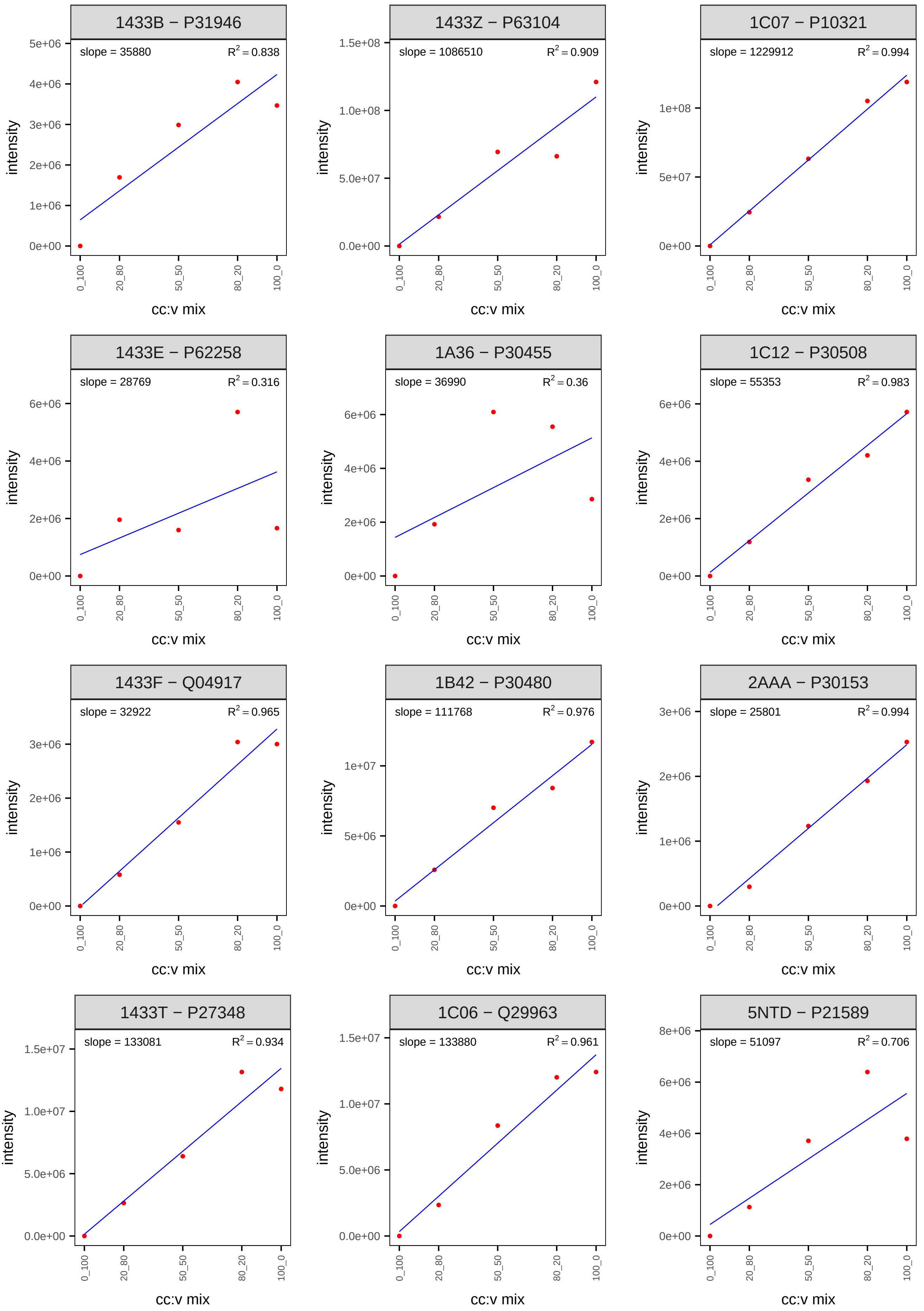
1433B − P31946
slope = 35880
R2 = 0.838
1433Z − P63104
slope = 1086510
R2 = 0.909
1C07 − P10321
slope = 1229912
R2 = 0.994
1433E − P62258
slope = 28769
R2 = 0.316
1A36 − P30455
slope = 36990
R2 = 0.36
1C12 − P30508
slope = 55353
R2 = 0.983
1433F − Q04917
slope = 32922
R2 = 0.965
1B42 − P30480
slope = 111768
R2 = 0.976
2AAA − P30153
slope = 25801
R2 = 0.994
1433T − P27348
slope = 133081
R2 = 0.934
1C06 − Q29963
slope = 133880
R2 = 0.961
5NTD − P21589
slope = 51097
R2 = 0.706
intensity
cc:v mix
0_100
20_80
50_50
80_20
100_0

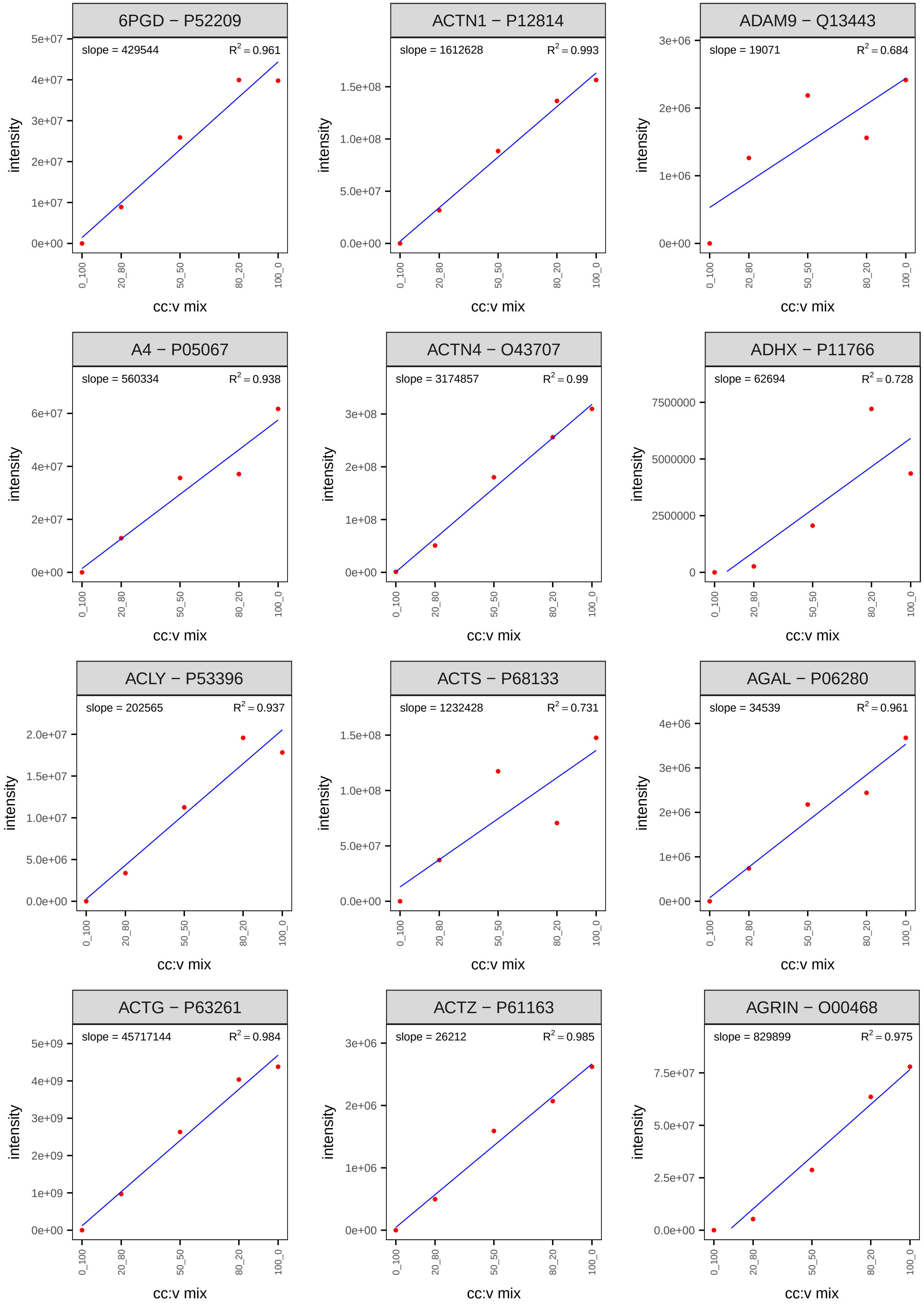
6PGD − P52209
slope = 429544
R2 = 0.961
ACTN1 − P12814
slope = 1612628
R2 = 0.993
ADAM9 − Q13443
slope = 19071
R2 = 0.684
A4 − P05067
slope = 560334
R2 = 0.938
ACTN4 − O43707
slope = 3174857
R2 = 0.99
ADHX − P11766
slope = 62694
R2 = 0.728
ACLY − P53396
slope = 202565
R2 = 0.937
ACTS − P68133
slope = 1232428
R2 = 0.731
AGAL − P06280
slope = 34539
R2 = 0.961
ACTG − P63261
slope = 45717144
R2 = 0.984
ACTZ − P61163
slope = 26212
R2 = 0.985
AGRIN − O00468
slope = 829899
R2 = 0.975
intensity
cc:v mix
0_100
20_80
50_50
80_20
100_0

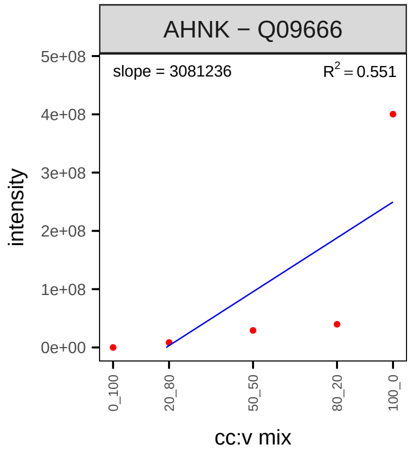
AHNK − Q09666
slope = 3081236
$R^2 = 0.551$
intensity
cc:v mix

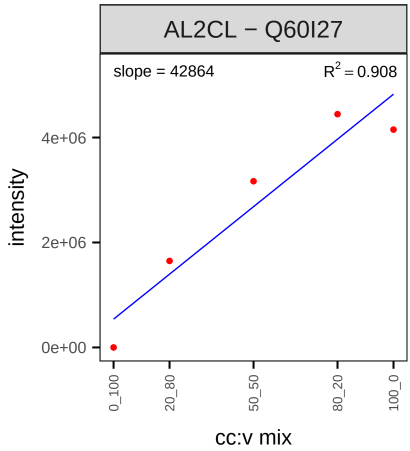
AL2CL − Q60I27
slope = 42864
$R^2 = 0.908$
intensity
cc:v mix

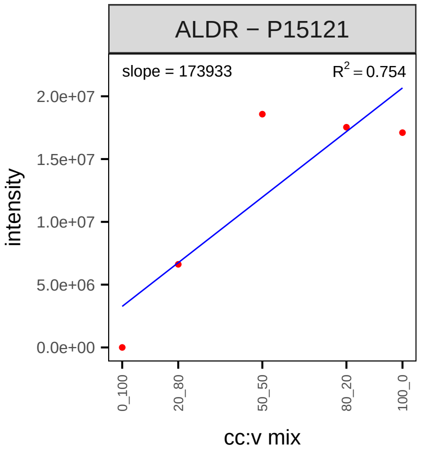
ALDR − P15121
slope = 173933
$R^2 = 0.754$
intensity
cc:v mix

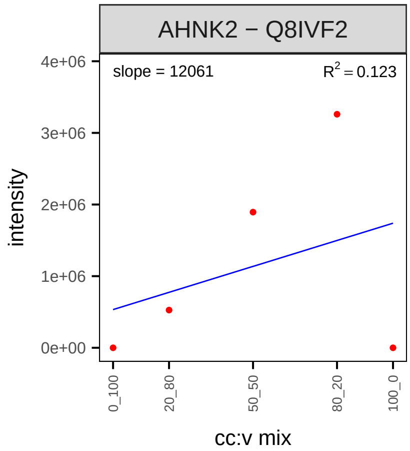
AHNK2 − Q8IVF2
slope = 12061
$R^2 = 0.123$
intensity
cc:v mix

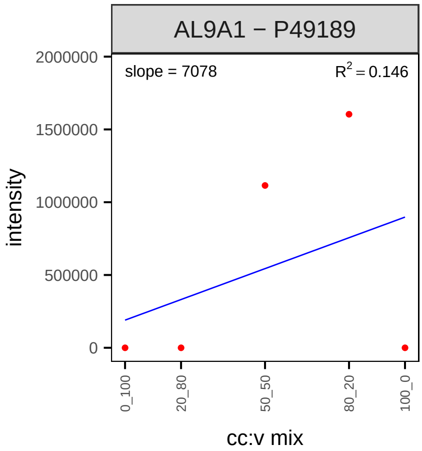
AL9A1 − P49189
slope = 7078
$R^2 = 0.146$
intensity
cc:v mix

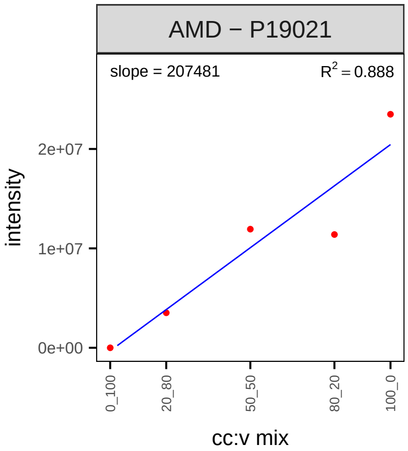
AMD − P19021
slope = 207481
$R^2 = 0.888$
intensity
cc:v mix

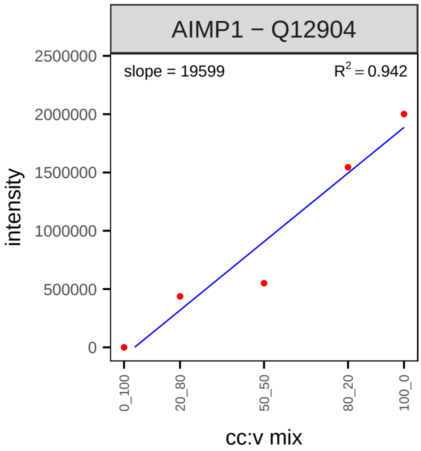
AIMP1 − Q12904
slope = 19599
$R^2 = 0.942$
intensity
cc:v mix

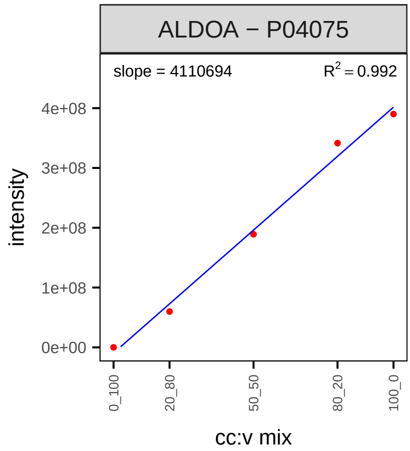
ALDOA − P04075
slope = 4110694
$R^2 = 0.992$
intensity
cc:v mix

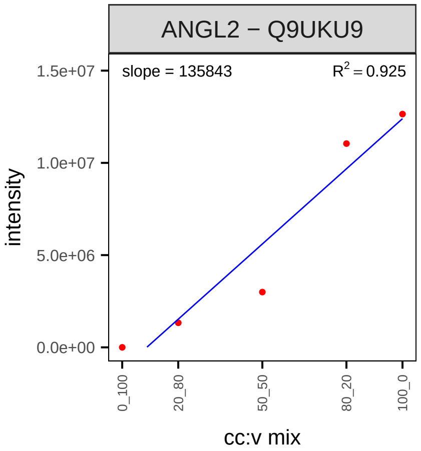
ANGL2 − Q9UKU9
slope = 135843
$R^2 = 0.925$
intensity
cc:v mix

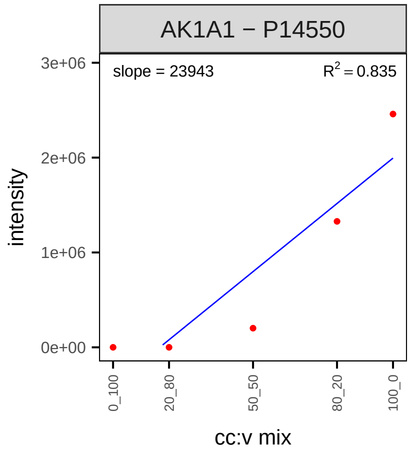
AK1A1 − P14550
slope = 23943
$R^2 = 0.835$
intensity
cc:v mix

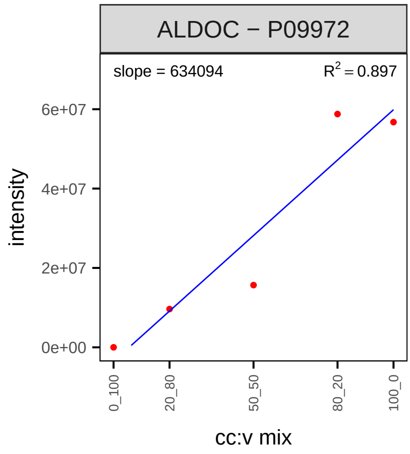
ALDOC − P09972
slope = 634094
$R^2 = 0.897$
intensity
cc:v mix

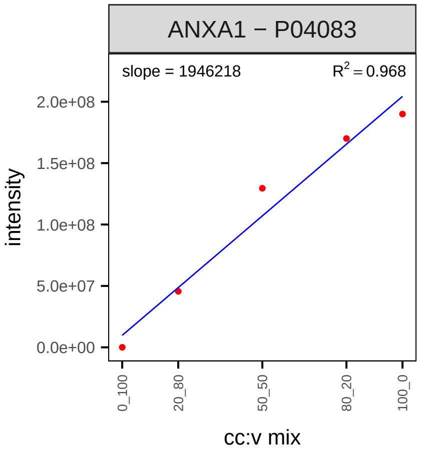
ANXA1 − P04083
slope = 1946218
$R^2 = 0.968$
intensity
cc:v mix

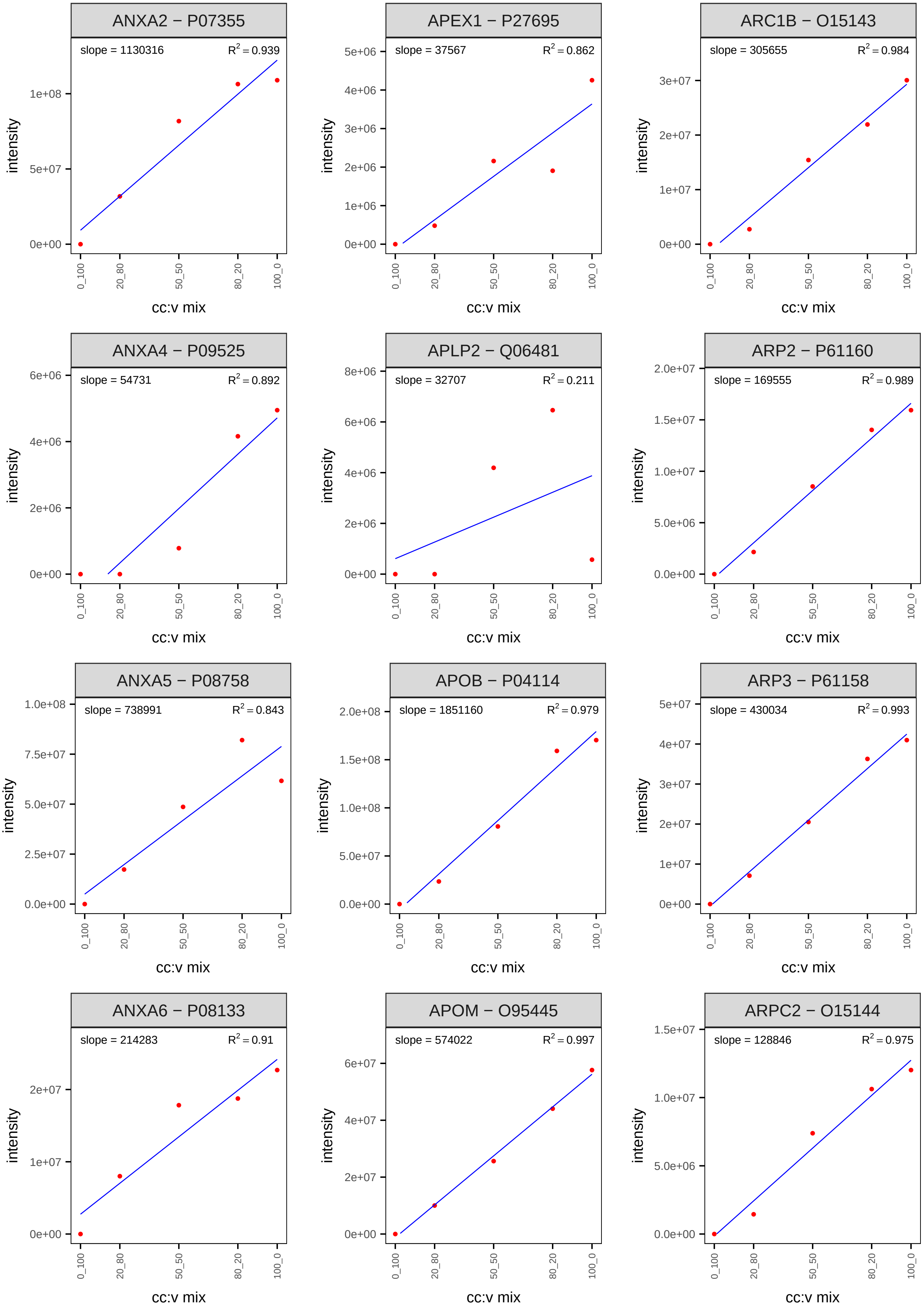
ANXA2 − P07355
slope = 1130316
$R^2 = 0.939$
APEX1 − P27695
slope = 37567
$R^2 = 0.862$
ARC1B − O15143
slope = 305655
$R^2 = 0.984$
ANXA4 − P09525
slope = 54731
$R^2 = 0.892$
APLP2 − Q06481
slope = 32707
$R^2 = 0.211$
ARP2 − P61160
slope = 169555
$R^2 = 0.989$
ANXA5 − P08758
slope = 738991
$R^2 = 0.843$
APOB − P04114
slope = 1851160
$R^2 = 0.979$
ARP3 − P61158
slope = 430034
$R^2 = 0.993$
ANXA6 − P08133
slope = 214283
$R^2 = 0.91$
APOM − O95445
slope = 574022
$R^2 = 0.997$
ARPC2 − O15144
slope = 128846
$R^2 = 0.975$
intensity
cc:v mix
0_100
20_80
50_50
80_20
100_0

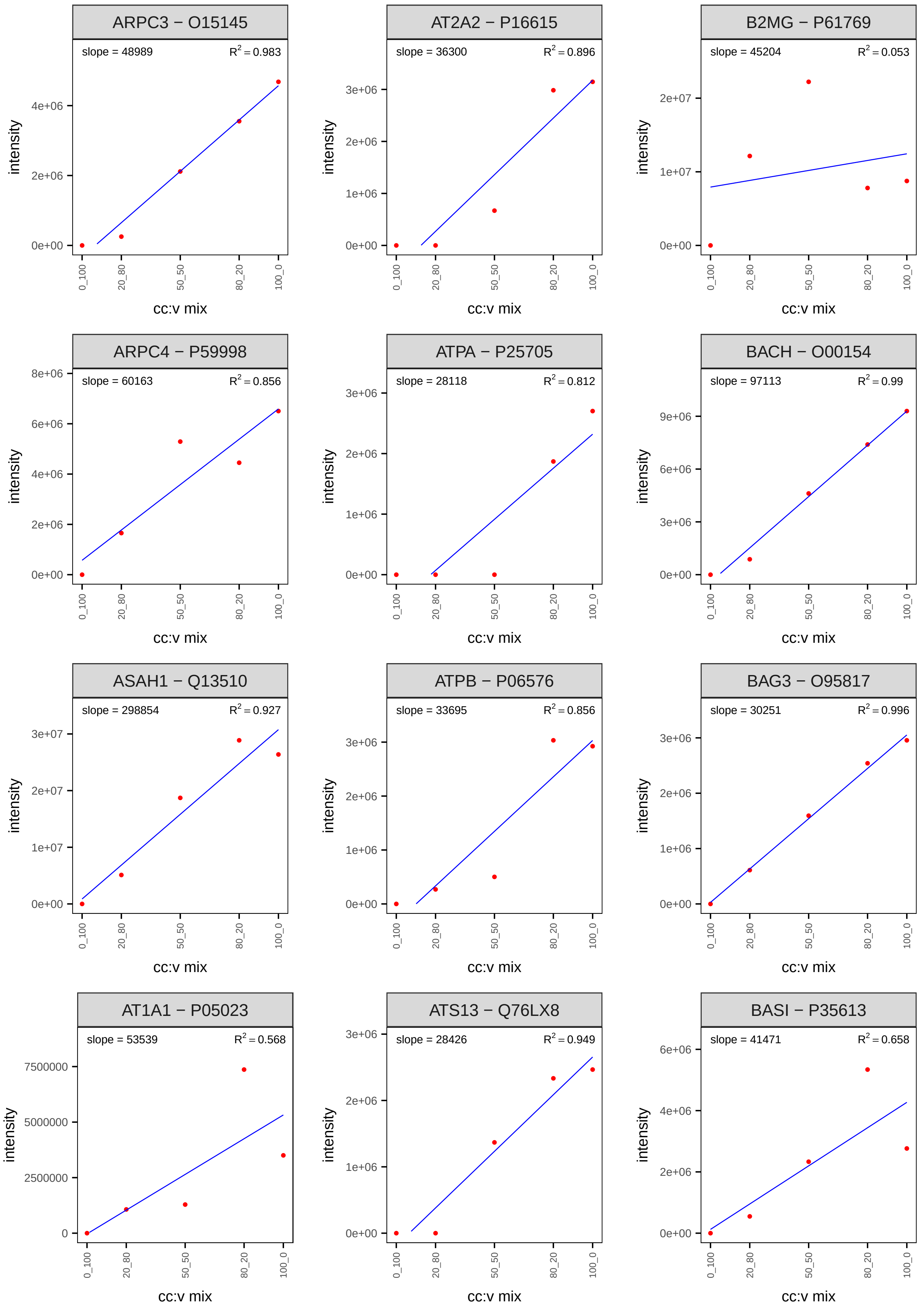
ARPC3 − O15145
slope = 48989
R² = 0.983
AT2A2 − P16615
slope = 36300
R² = 0.896
B2MG − P61769
slope = 45204
R² = 0.053
ARPC4 − P59998
slope = 60163
R² = 0.856
ATPA − P25705
slope = 28118
R² = 0.812
BACH − O00154
slope = 97113
R² = 0.99
ASAH1 − Q13510
slope = 298854
R² = 0.927
ATPB − P06576
slope = 33695
R² = 0.856
BAG3 − O95817
slope = 30251
R² = 0.996
AT1A1 − P05023
slope = 53539
R² = 0.568
ATS13 − Q76LX8
slope = 28426
R² = 0.949
BASI − P35613
slope = 41471
R² = 0.658
intensity
cc:v mix
0_100
20_80
50_50
80_20
100_0

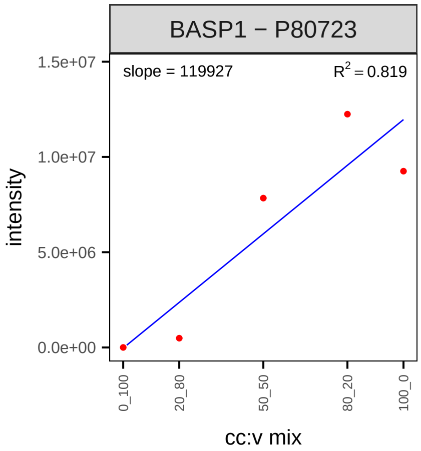
BASP1 − P80723
slope = 119927
$R^2 = 0.819$
intensity
1.5e+07
1.0e+07
5.0e+06
0.0e+00
0_100
20_80
50_50
80_20
100_0
cc:v mix

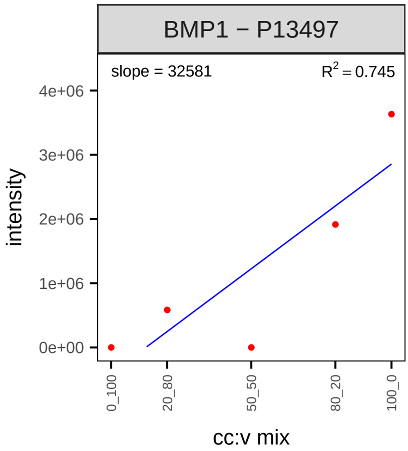
BMP1 − P13497
slope = 32581
$R^2 = 0.745$
intensity
4e+06
3e+06
2e+06
1e+06
0e+00
0_100
20_80
50_50
80_20
100_0
cc:v mix

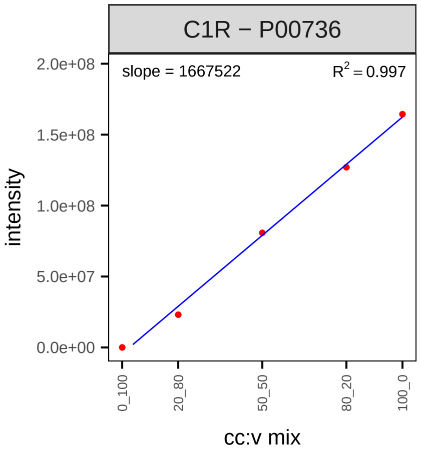
C1R − P00736
slope = 1667522
$R^2 = 0.997$
intensity
2.0e+08
1.5e+08
1.0e+08
5.0e+07
0.0e+00
0_100
20_80
50_50
80_20
100_0
cc:v mix

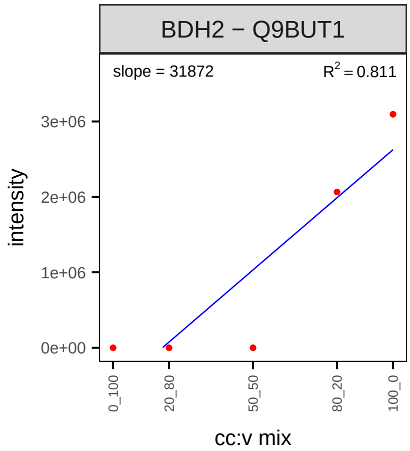
BDH2 − Q9BUT1
slope = 31872
$R^2 = 0.811$
intensity
3e+06
2e+06
1e+06
0e+00
0_100
20_80
50_50
80_20
100_0
cc:v mix

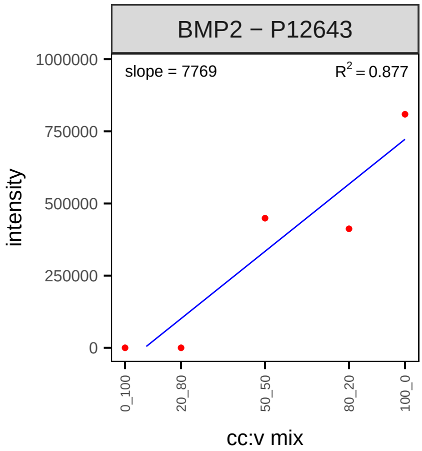
BMP2 − P12643
slope = 7769
$R^2 = 0.877$
intensity
1000000
750000
500000
250000
0
0_100
20_80
50_50
80_20
100_0
cc:v mix

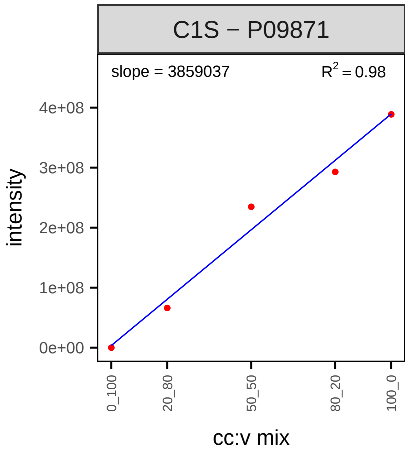
C1S − P09871
slope = 3859037
$R^2 = 0.98$
intensity
4e+08
3e+08
2e+08
1e+08
0e+00
0_100
20_80
50_50
80_20
100_0
cc:v mix

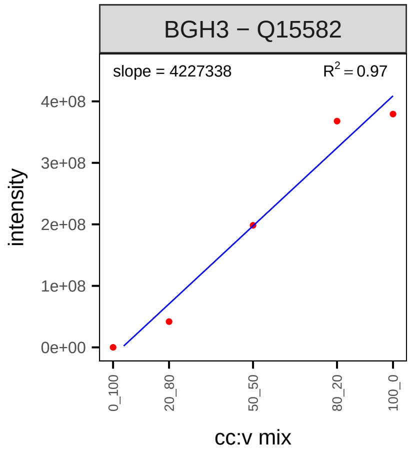
BGH3 − Q15582
slope = 4227338
$R^2 = 0.97$
intensity
4e+08
3e+08
2e+08
1e+08
0e+00
0_100
20_80
50_50
80_20
100_0
cc:v mix

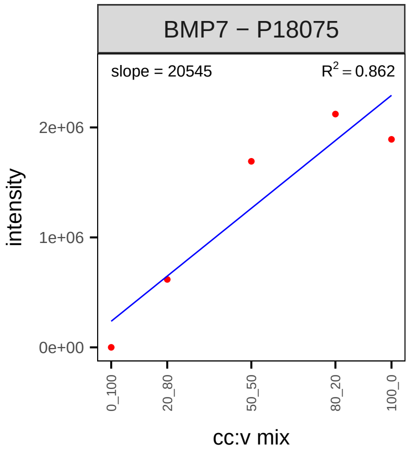
BMP7 − P18075
slope = 20545
$R^2 = 0.862$
intensity
2e+06
1e+06
0e+00
0_100
20_80
50_50
80_20
100_0
cc:v mix

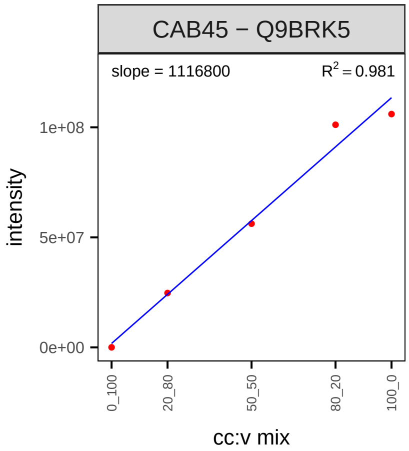
CAB45 − Q9BRK5
slope = 1116800
$R^2 = 0.981$
intensity
1e+08
5e+07
0e+00
0_100
20_80
50_50
80_20
100_0
cc:v mix

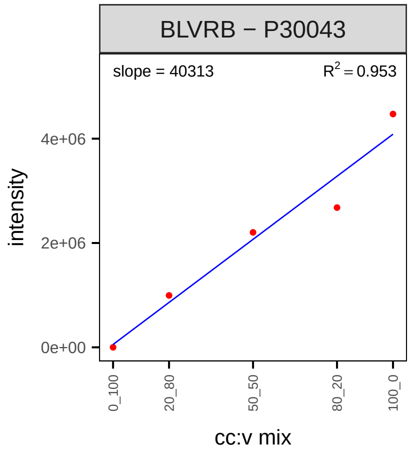
BLVRB − P30043
slope = 40313
$R^2 = 0.953$
intensity
4e+06
2e+06
0e+00
0_100
20_80
50_50
80_20
100_0
cc:v mix

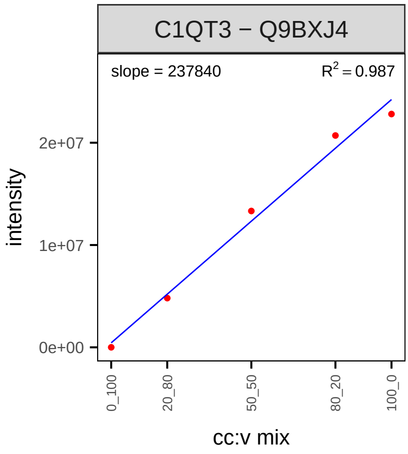
C1QT3 − Q9BXJ4
slope = 237840
$R^2 = 0.987$
intensity
2e+07
1e+07
0e+00
0_100
20_80
50_50
80_20
100_0
cc:v mix

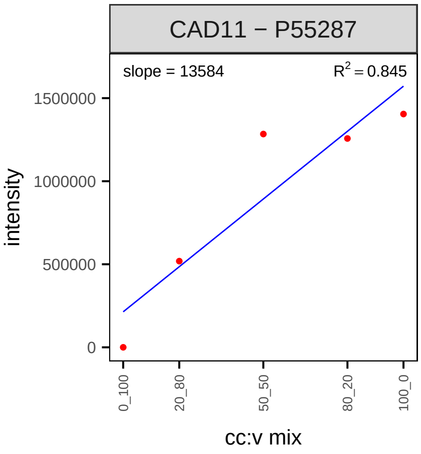
CAD11 − P55287
slope = 13584
$R^2 = 0.845$
intensity
1500000
1000000
500000
0
0_100
20_80
50_50
80_20
100_0
cc:v mix

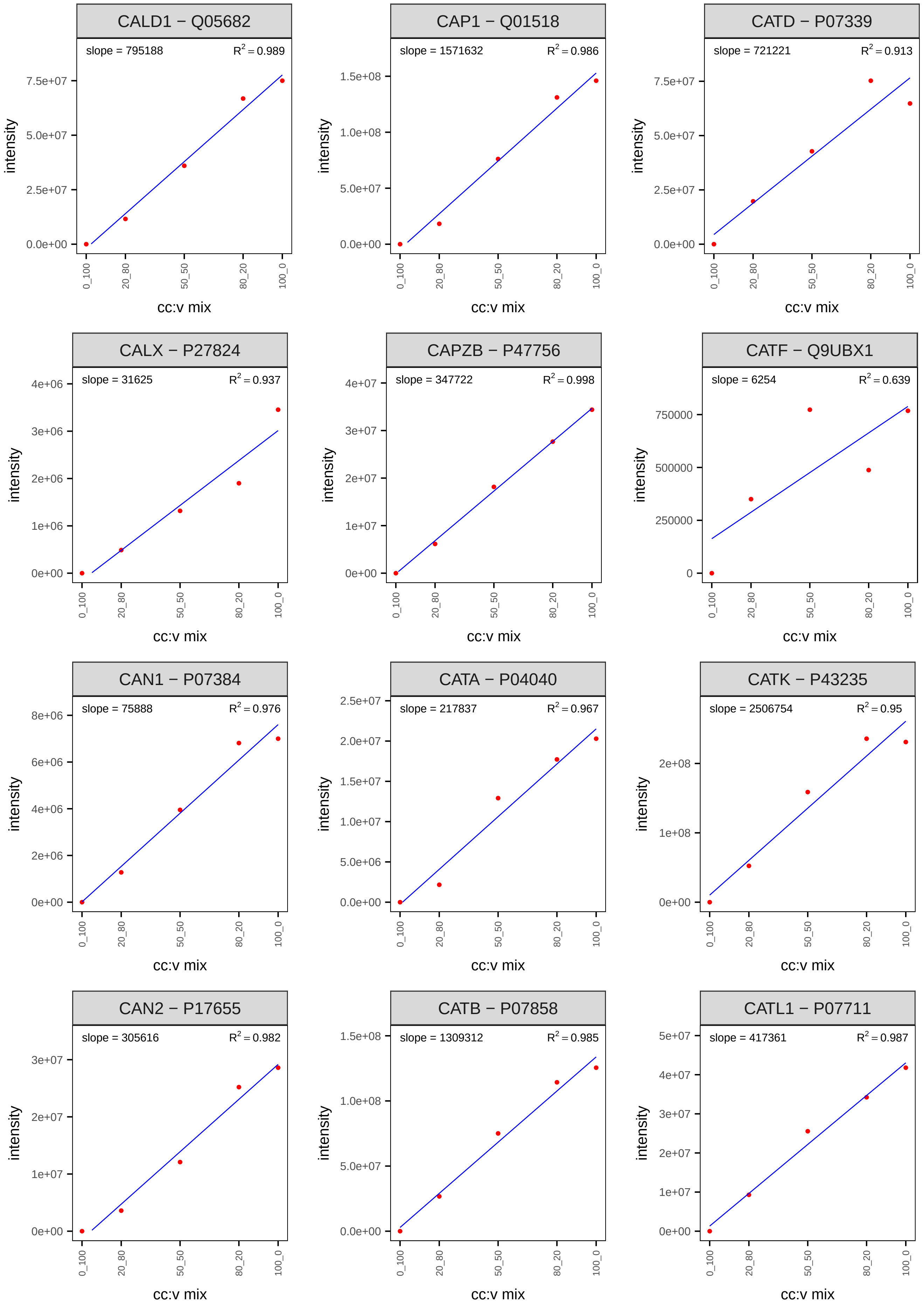
CALD1 − Q05682
slope = 795188
R2 = 0.989
CAP1 − Q01518
slope = 1571632
R2 = 0.986
CATD − P07339
slope = 721221
R2 = 0.913
CALX − P27824
slope = 31625
R2 = 0.937
CAPZB − P47756
slope = 347722
R2 = 0.998
CATF − Q9UBX1
slope = 6254
R2 = 0.639
CAN1 − P07384
slope = 75888
R2 = 0.976
CATA − P04040
slope = 217837
R2 = 0.967
CATK − P43235
slope = 2506754
R2 = 0.95
CAN2 − P17655
slope = 305616
R2 = 0.982
CATB − P07858
slope = 1309312
R2 = 0.985
CATL1 − P07711
slope = 417361
R2 = 0.987
intensity
cc:v mix
0_100
20_80
50_50
80_20
100_0

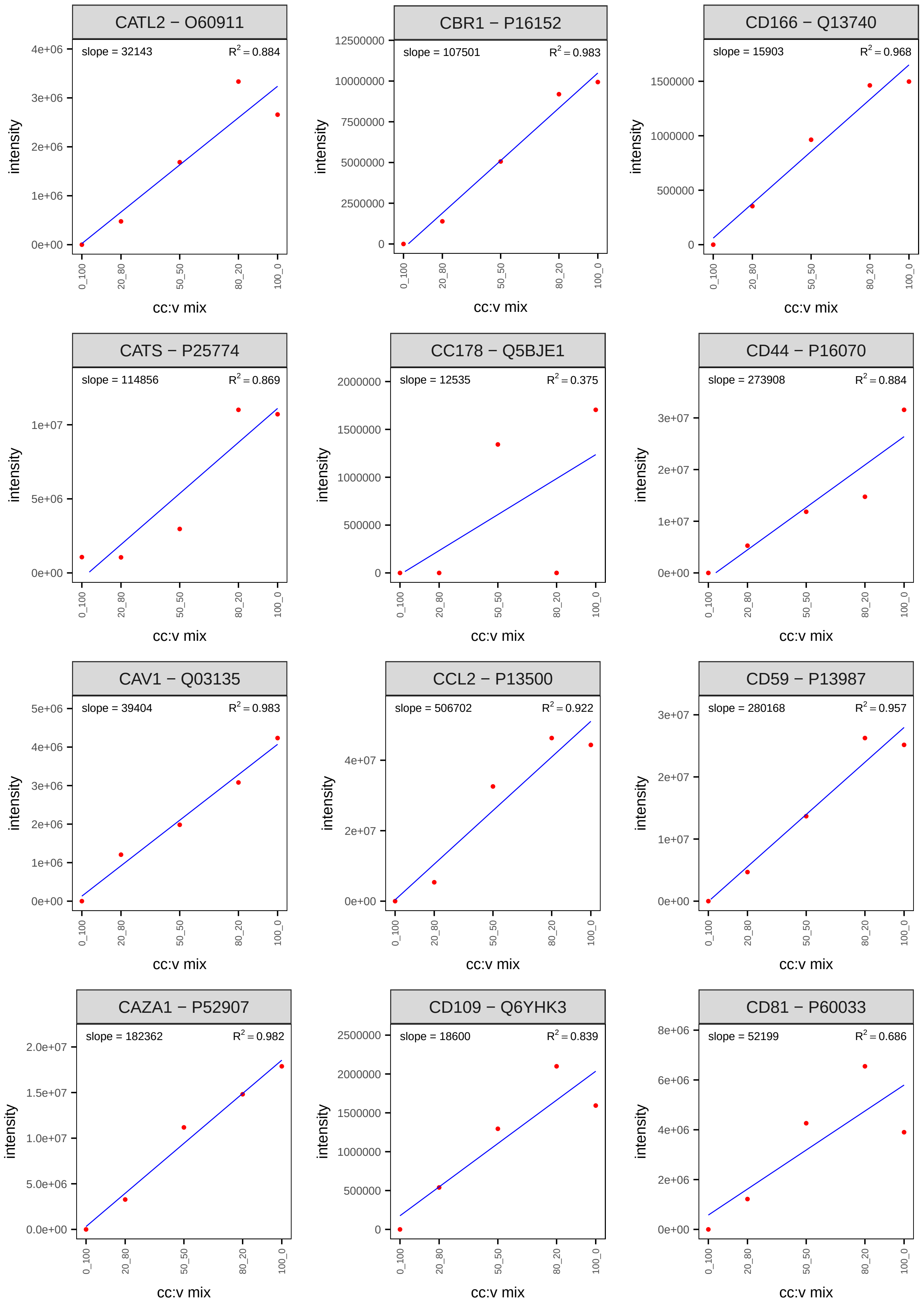
CATL2 − O60911
slope = 32143
R2 = 0.884
CBR1 − P16152
slope = 107501
R2 = 0.983
CD166 − Q13740
slope = 15903
R2 = 0.968
CATS − P25774
slope = 114856
R2 = 0.869
CC178 − Q5BJE1
slope = 12535
R2 = 0.375
CD44 − P16070
slope = 273908
R2 = 0.884
CAV1 − Q03135
slope = 39404
R2 = 0.983
CCL2 − P13500
slope = 506702
R2 = 0.922
CD59 − P13987
slope = 280168
R2 = 0.957
CAZA1 − P52907
slope = 182362
R2 = 0.982
CD109 − Q6YHK3
slope = 18600
R2 = 0.839
CD81 − P60033
slope = 52199
R2 = 0.686
intensity
cc:v mix
0_100
20_80
50_50
80_20
100_0

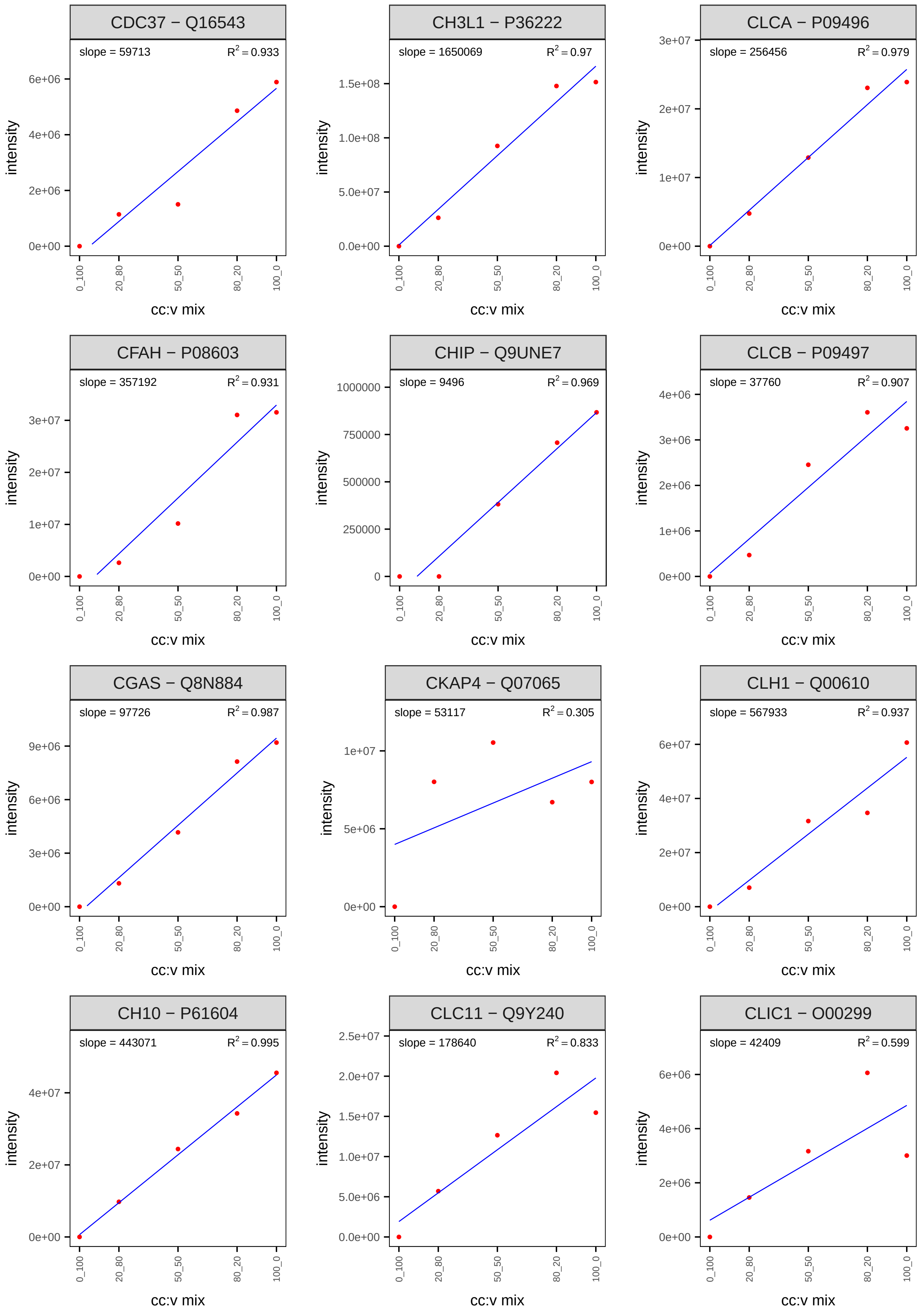
CDC37 − Q16543
slope = 59713
R2 = 0.933
CH3L1 − P36222
slope = 1650069
R2 = 0.97
CLCA − P09496
slope = 256456
R2 = 0.979
CFAH − P08603
slope = 357192
R2 = 0.931
CHIP − Q9UNE7
slope = 9496
R2 = 0.969
CLCB − P09497
slope = 37760
R2 = 0.907
CGAS − Q8N884
slope = 97726
R2 = 0.987
CKAP4 − Q07065
slope = 53117
R2 = 0.305
CLH1 − Q00610
slope = 567933
R2 = 0.937
CH10 − P61604
slope = 443071
R2 = 0.995
CLC11 − Q9Y240
slope = 178640
R2 = 0.833
CLIC1 − O00299
slope = 42409
R2 = 0.599
intensity
cc:v mix
0_100
20_80
50_50
80_20
100_0

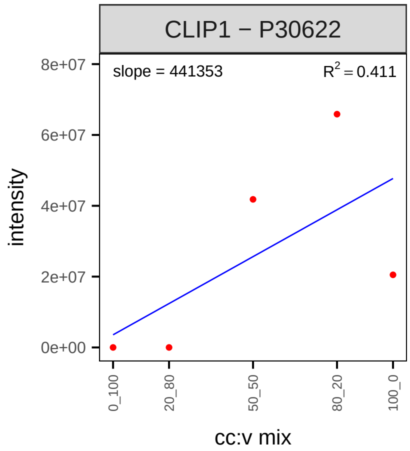
CLIP1 − P30622
slope = 441353
R2 = 0.411
8e+07
6e+07
4e+07
2e+07
0e+00
intensity
0_100
20_80
50_50
80_20
100_0
cc:v mix

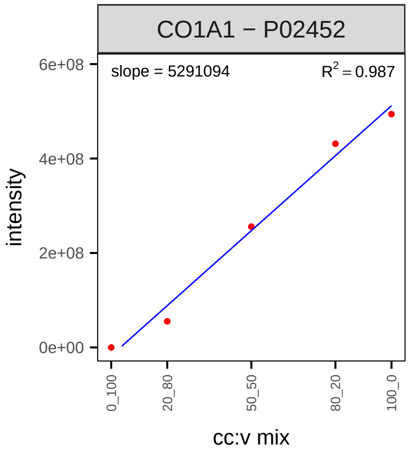
CO1A1 − P02452
slope = 5291094
R2 = 0.987
6e+08
4e+08
2e+08
0e+00
intensity
0_100
20_80
50_50
80_20
100_0
cc:v mix

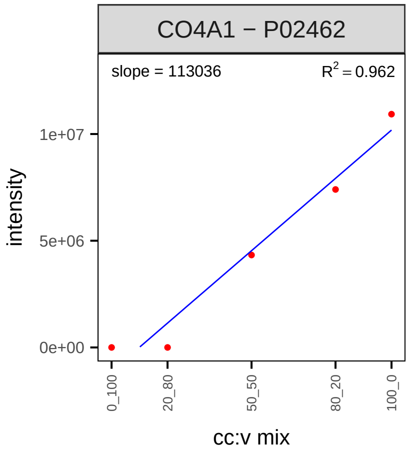
CO4A1 − P02462
slope = 113036
R2 = 0.962
1e+07
5e+06
0e+00
intensity
0_100
20_80
50_50
80_20
100_0
cc:v mix

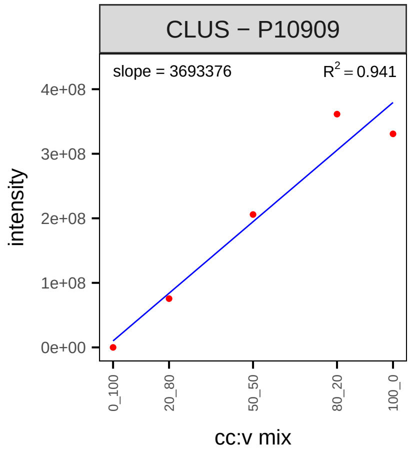
CLUS − P10909
slope = 3693376
R2 = 0.941
4e+08
3e+08
2e+08
1e+08
0e+00
intensity
0_100
20_80
50_50
80_20
100_0
cc:v mix

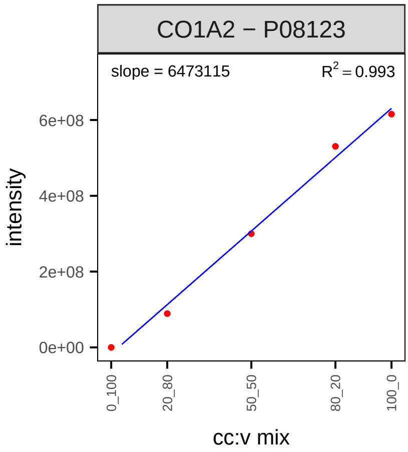
CO1A2 − P08123
slope = 6473115
R2 = 0.993
6e+08
4e+08
2e+08
0e+00
intensity
0_100
20_80
50_50
80_20
100_0
cc:v mix

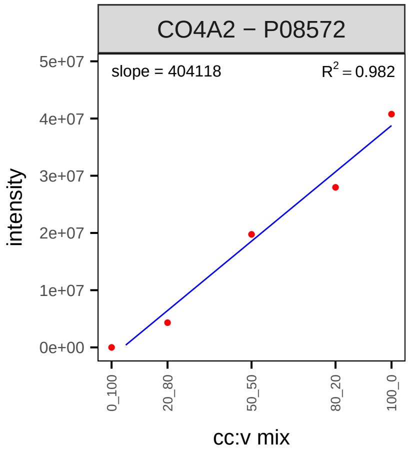
CO4A2 − P08572
slope = 404118
R2 = 0.982
5e+07
4e+07
3e+07
2e+07
1e+07
0e+00
intensity
0_100
20_80
50_50
80_20
100_0
cc:v mix

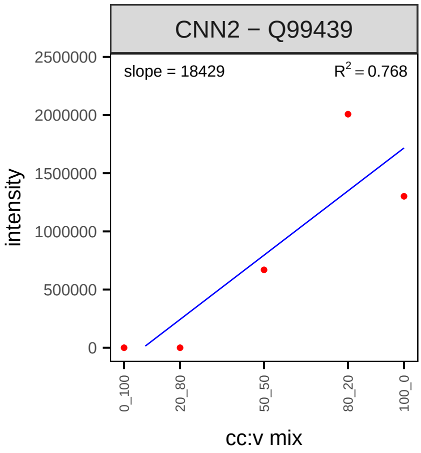
CNN2 − Q99439
slope = 18429
R2 = 0.768
2500000
2000000
1500000
1000000
500000
0
intensity
0_100
20_80
50_50
80_20
100_0
cc:v mix

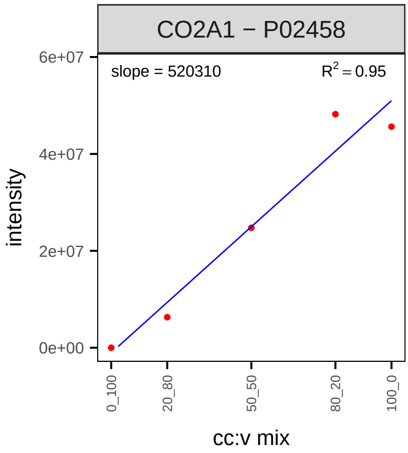
CO2A1 − P02458
slope = 520310
R2 = 0.95
6e+07
4e+07
2e+07
0e+00
intensity
0_100
20_80
50_50
80_20
100_0
cc:v mix

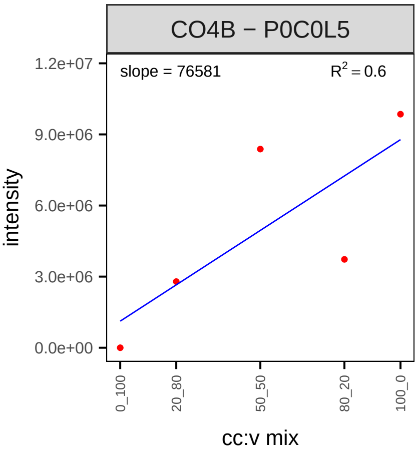
CO4B − P0C0L5
slope = 76581
R2 = 0.6
1.2e+07
9.0e+06
6.0e+06
3.0e+06
0.0e+00
intensity
0_100
20_80
50_50
80_20
100_0
cc:v mix

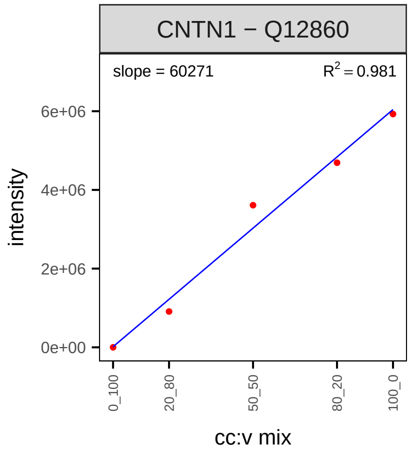
CNTN1 − Q12860
slope = 60271
R2 = 0.981
6e+06
4e+06
2e+06
0e+00
intensity
0_100
20_80
50_50
80_20
100_0
cc:v mix

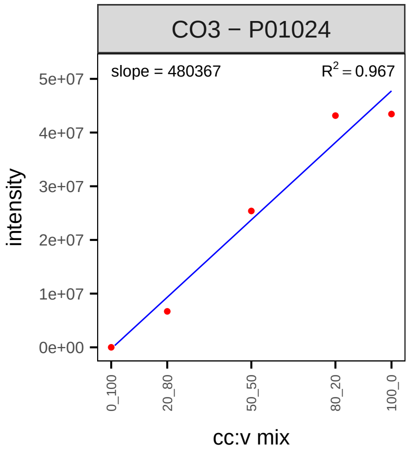
CO3 − P01024
slope = 480367
R2 = 0.967
5e+07
4e+07
3e+07
2e+07
1e+07
0e+00
intensity
0_100
20_80
50_50
80_20
100_0
cc:v mix

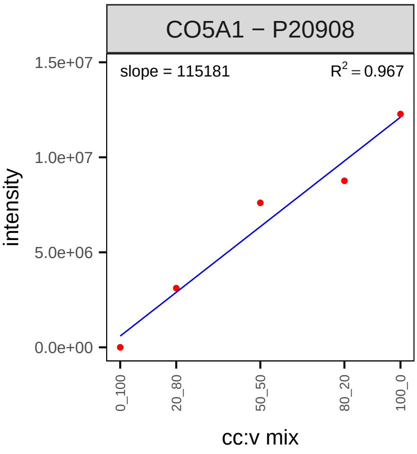
CO5A1 − P20908
slope = 115181
R2 = 0.967
1.5e+07
1.0e+07
5.0e+06
0.0e+00
intensity
0_100
20_80
50_50
80_20
100_0
cc:v mix

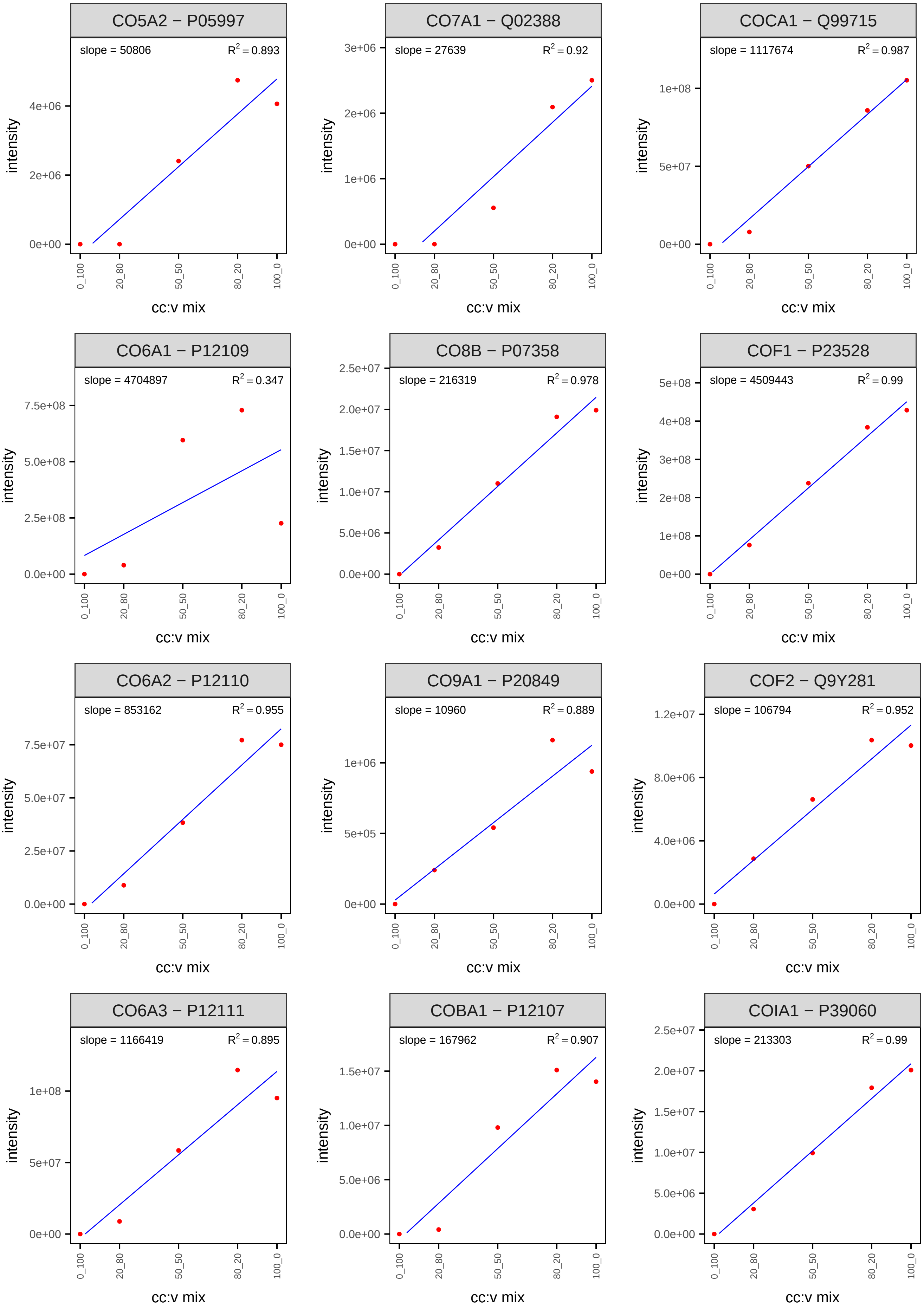
CO5A2 − P05997
slope = 50806
R^2 = 0.893
CO7A1 − Q02388
slope = 27639
R^2 = 0.92
COCA1 − Q99715
slope = 1117674
R^2 = 0.987
CO6A1 − P12109
slope = 4704897
R^2 = 0.347
CO8B − P07358
slope = 216319
R^2 = 0.978
COF1 − P23528
slope = 4509443
R^2 = 0.99
CO6A2 − P12110
slope = 853162
R^2 = 0.955
CO9A1 − P20849
slope = 10960
R^2 = 0.889
COF2 − Q9Y281
slope = 106794
R^2 = 0.952
CO6A3 − P12111
slope = 1166419
R^2 = 0.895
COBA1 − P12107
slope = 167962
R^2 = 0.907
COIA1 − P39060
slope = 213303
R^2 = 0.99
intensity
cc:v mix
0_100
20_80
50_50
80_20
100_0

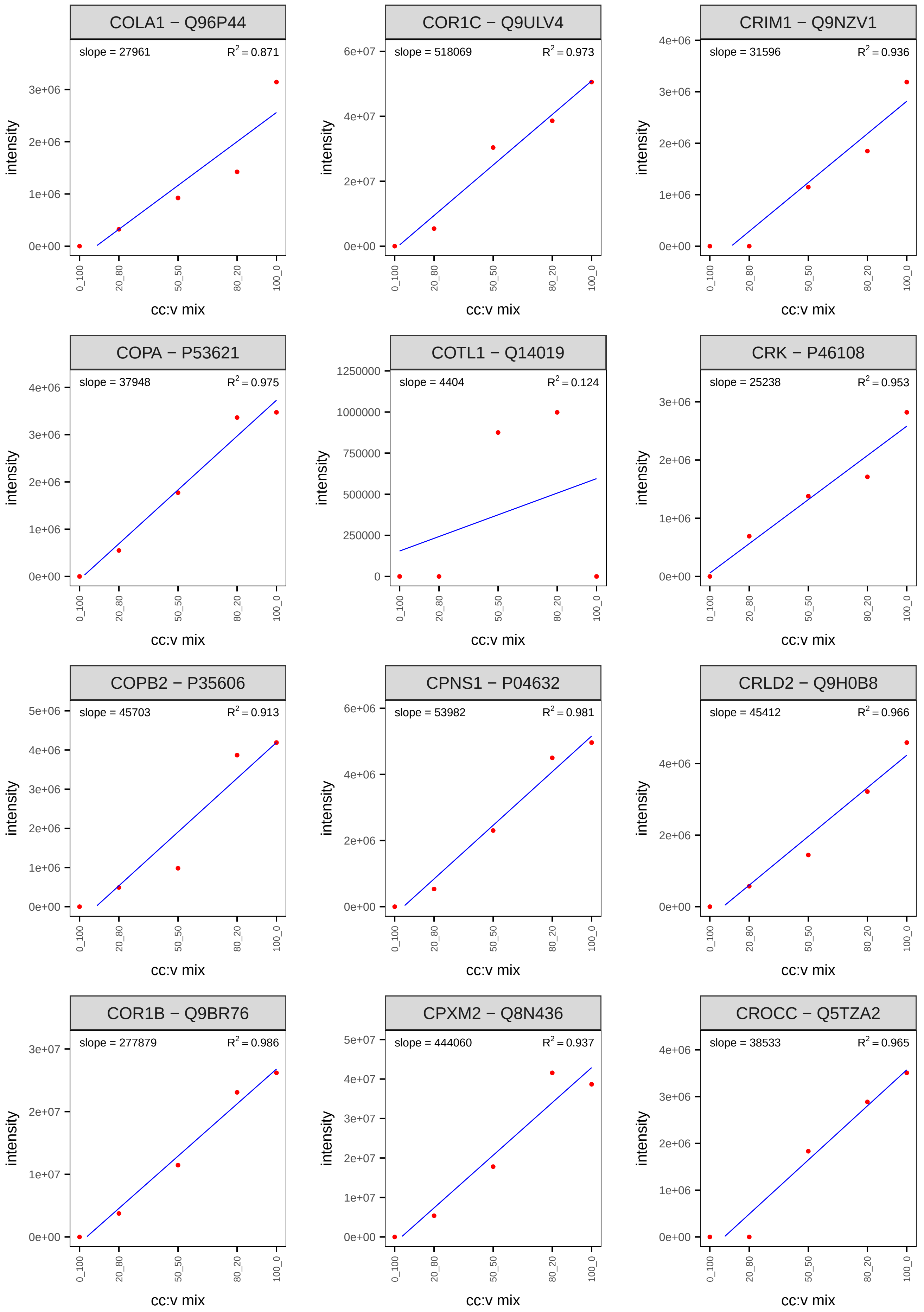
COLA1 − Q96P44
slope = 27961
$R^2 = 0.871$
COR1C − Q9ULV4
slope = 518069
$R^2 = 0.973$
CRIM1 − Q9NZV1
slope = 31596
$R^2 = 0.936$
COPA − P53621
slope = 37948
$R^2 = 0.975$
COTL1 − Q14019
slope = 4404
$R^2 = 0.124$
CRK − P46108
slope = 25238
$R^2 = 0.953$
COPB2 − P35606
slope = 45703
$R^2 = 0.913$
CPNS1 − P04632
slope = 53982
$R^2 = 0.981$
CRLD2 − Q9H0B8
slope = 45412
$R^2 = 0.966$
COR1B − Q9BR76
slope = 277879
$R^2 = 0.986$
CPXM2 − Q8N436
slope = 444060
$R^2 = 0.937$
CROCC − Q5TZA2
slope = 38533
$R^2 = 0.965$
intensity
cc:v mix
0_100
20_80
50_50
80_20
100_0

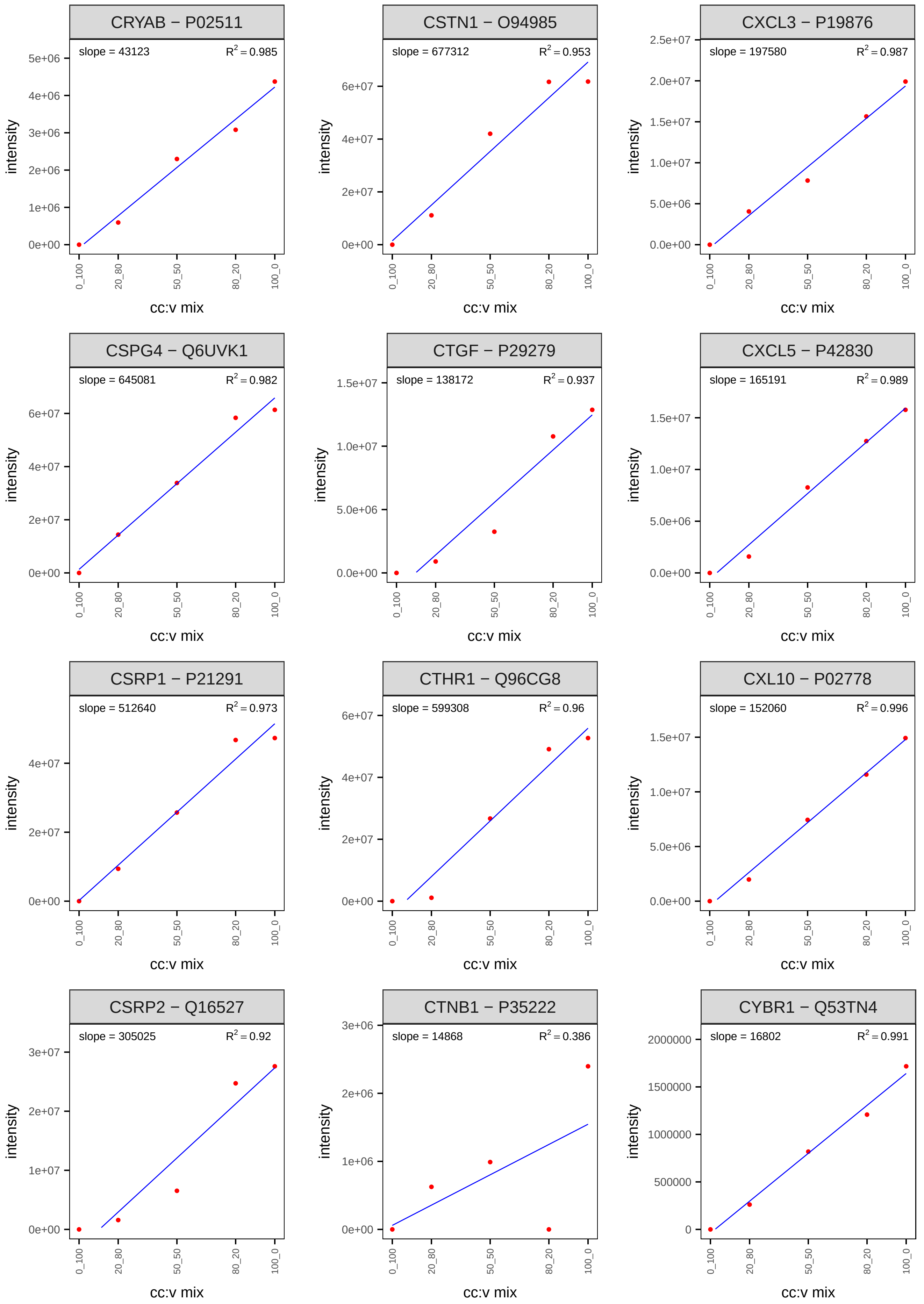
CRYAB − P02511
slope = 43123
R2 = 0.985
CSTN1 − O94985
slope = 677312
R2 = 0.953
CXCL3 − P19876
slope = 197580
R2 = 0.987
CSPG4 − Q6UVK1
slope = 645081
R2 = 0.982
CTGF − P29279
slope = 138172
R2 = 0.937
CXCL5 − P42830
slope = 165191
R2 = 0.989
CSRP1 − P21291
slope = 512640
R2 = 0.973
CTHR1 − Q96CG8
slope = 599308
R2 = 0.96
CXL10 − P02778
slope = 152060
R2 = 0.996
CSRP2 − Q16527
slope = 305025
R2 = 0.92
CTNB1 − P35222
slope = 14868
R2 = 0.386
CYBR1 − Q53TN4
slope = 16802
R2 = 0.991
intensity
cc:v mix
0_100
20_80
50_50
80_20
100_0

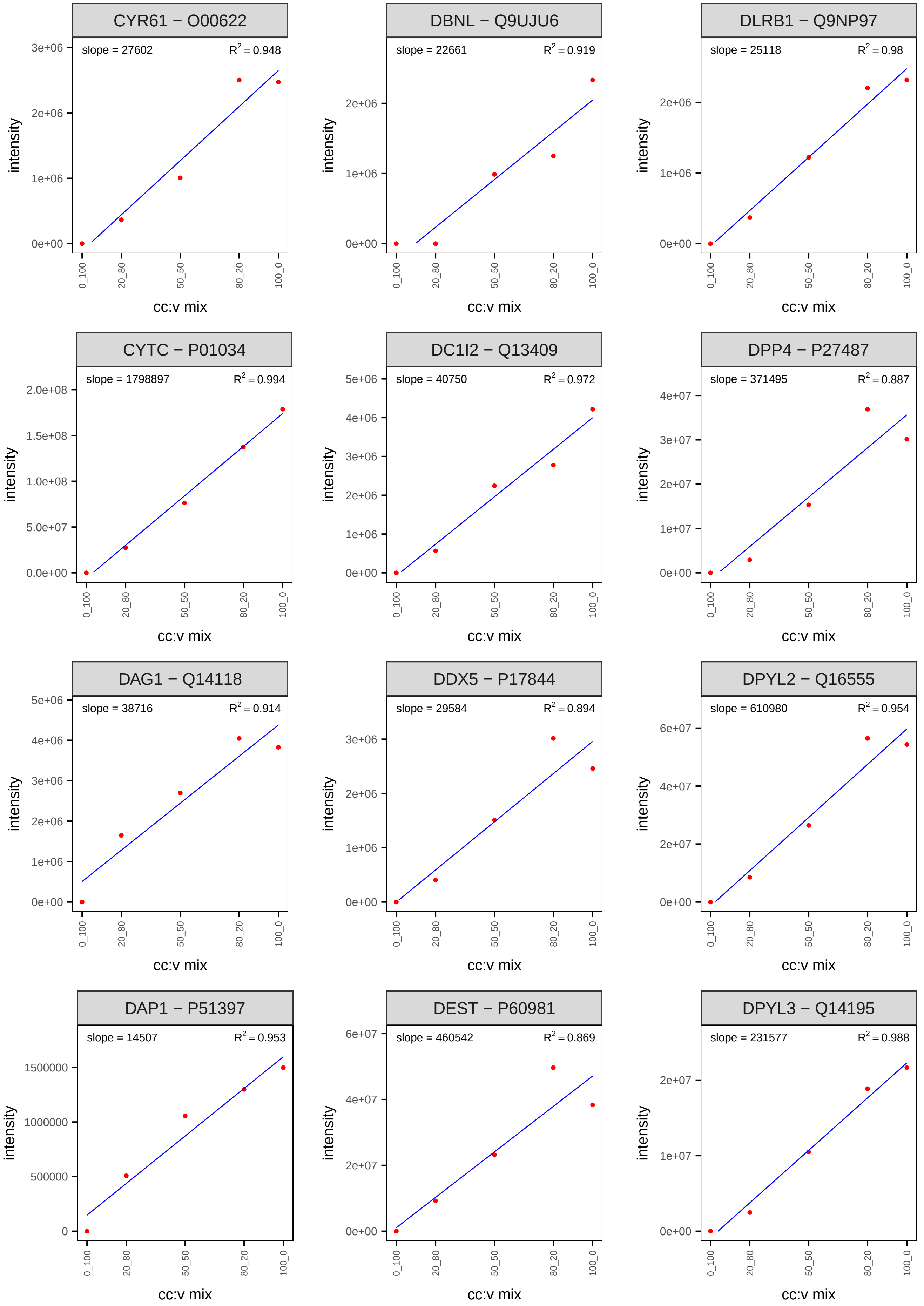
CYR61 − O00622
slope = 27602
R² = 0.948
DBNL − Q9UJU6
slope = 22661
R² = 0.919
DLRB1 − Q9NP97
slope = 25118
R² = 0.98
CYTC − P01034
slope = 1798897
R² = 0.994
DC1I2 − Q13409
slope = 40750
R² = 0.972
DPP4 − P27487
slope = 371495
R² = 0.887
DAG1 − Q14118
slope = 38716
R² = 0.914
DDX5 − P17844
slope = 29584
R² = 0.894
DPYL2 − Q16555
slope = 610980
R² = 0.954
DAP1 − P51397
slope = 14507
R² = 0.953
DEST − P60981
slope = 460542
R² = 0.869
DPYL3 − Q14195
slope = 231577
R² = 0.988
intensity
cc:v mix
0_100
20_80
50_50
80_20
100_0

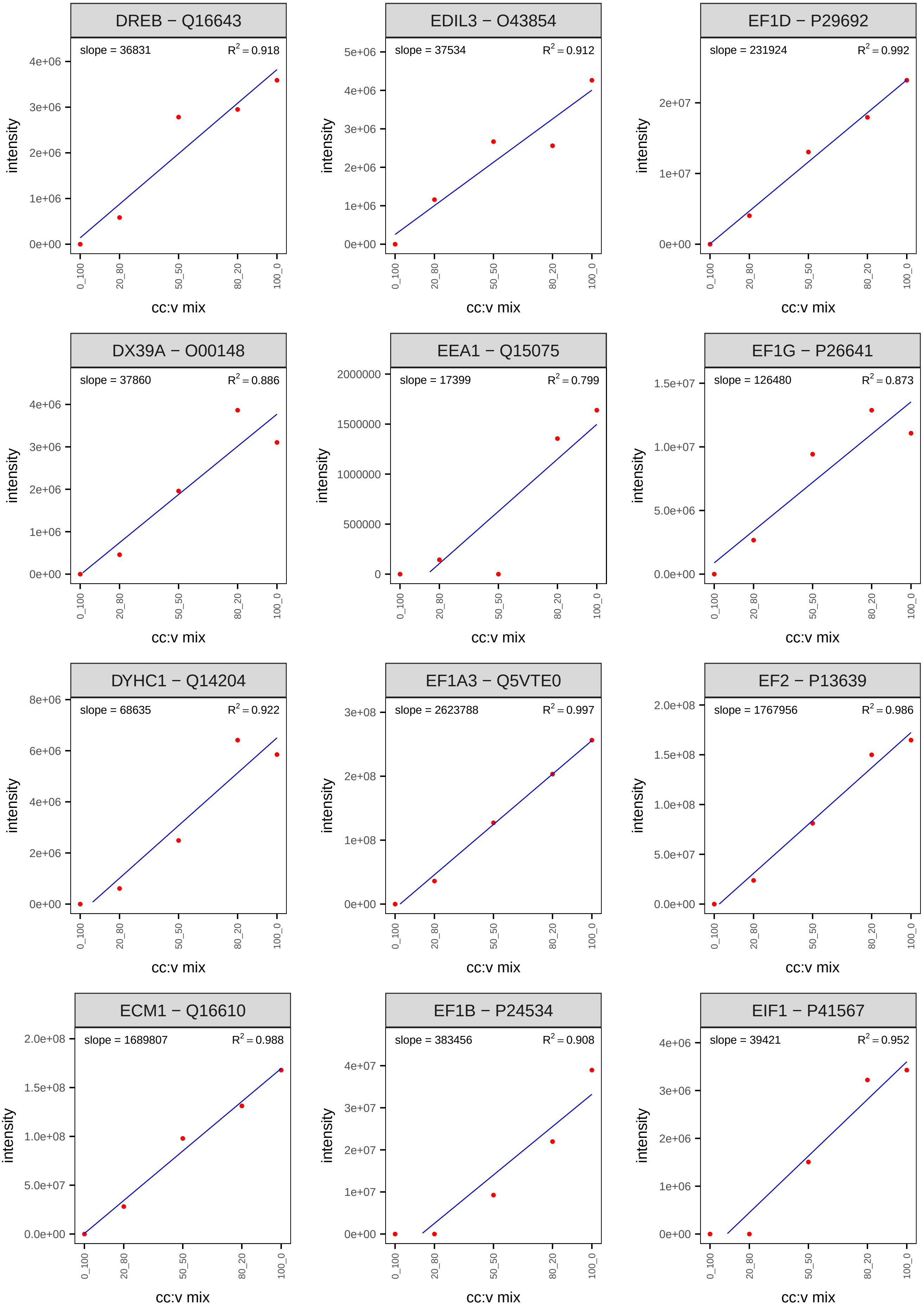
DREB − Q16643
slope = 36831
$R^2 = 0.918$
EDIL3 − O43854
slope = 37534
$R^2 = 0.912$
EF1D − P29692
slope = 231924
$R^2 = 0.992$
DX39A − O00148
slope = 37860
$R^2 = 0.886$
EEA1 − Q15075
slope = 17399
$R^2 = 0.799$
EF1G − P26641
slope = 126480
$R^2 = 0.873$
DYHC1 − Q14204
slope = 68635
$R^2 = 0.922$
EF1A3 − Q5VTE0
slope = 2623788
$R^2 = 0.997$
EF2 − P13639
slope = 1767956
$R^2 = 0.986$
ECM1 − Q16610
slope = 1689807
$R^2 = 0.988$
EF1B − P24534
slope = 383456
$R^2 = 0.908$
EIF1 − P41567
slope = 39421
$R^2 = 0.952$
intensity
cc:v mix
0_100
20_80
50_50
80_20
100_0

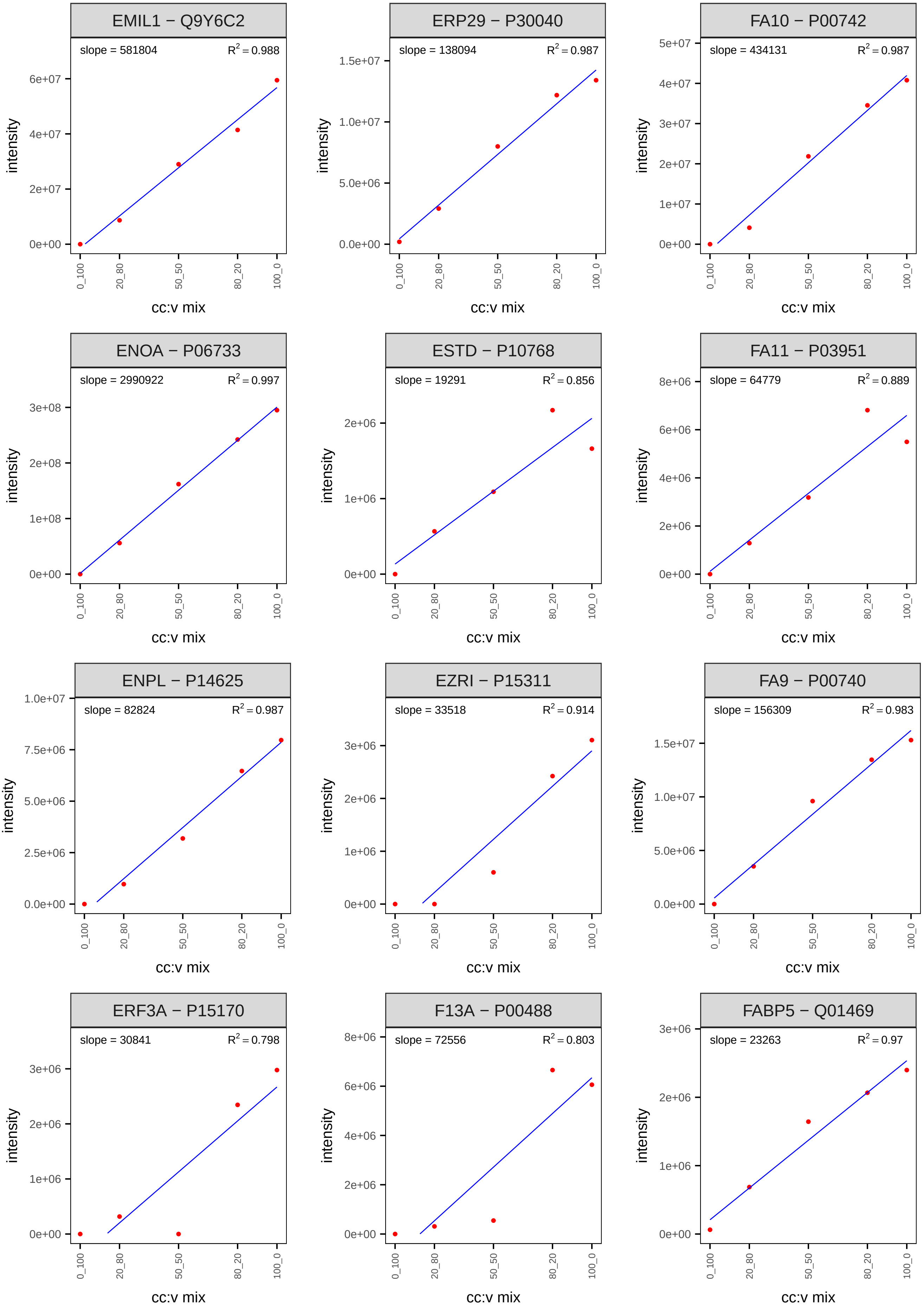
EMIL1 − Q9Y6C2
slope = 581804
R2 = 0.988
ERP29 − P30040
slope = 138094
R2 = 0.987
FA10 − P00742
slope = 434131
R2 = 0.987
ENOA − P06733
slope = 2990922
R2 = 0.997
ESTD − P10768
slope = 19291
R2 = 0.856
FA11 − P03951
slope = 64779
R2 = 0.889
ENPL − P14625
slope = 82824
R2 = 0.987
EZRI − P15311
slope = 33518
R2 = 0.914
FA9 − P00740
slope = 156309
R2 = 0.983
ERF3A − P15170
slope = 30841
R2 = 0.798
F13A − P00488
slope = 72556
R2 = 0.803
FABP5 − Q01469
slope = 23263
R2 = 0.97
intensity
cc:v mix
0_100
20_80
50_50
80_20
100_0

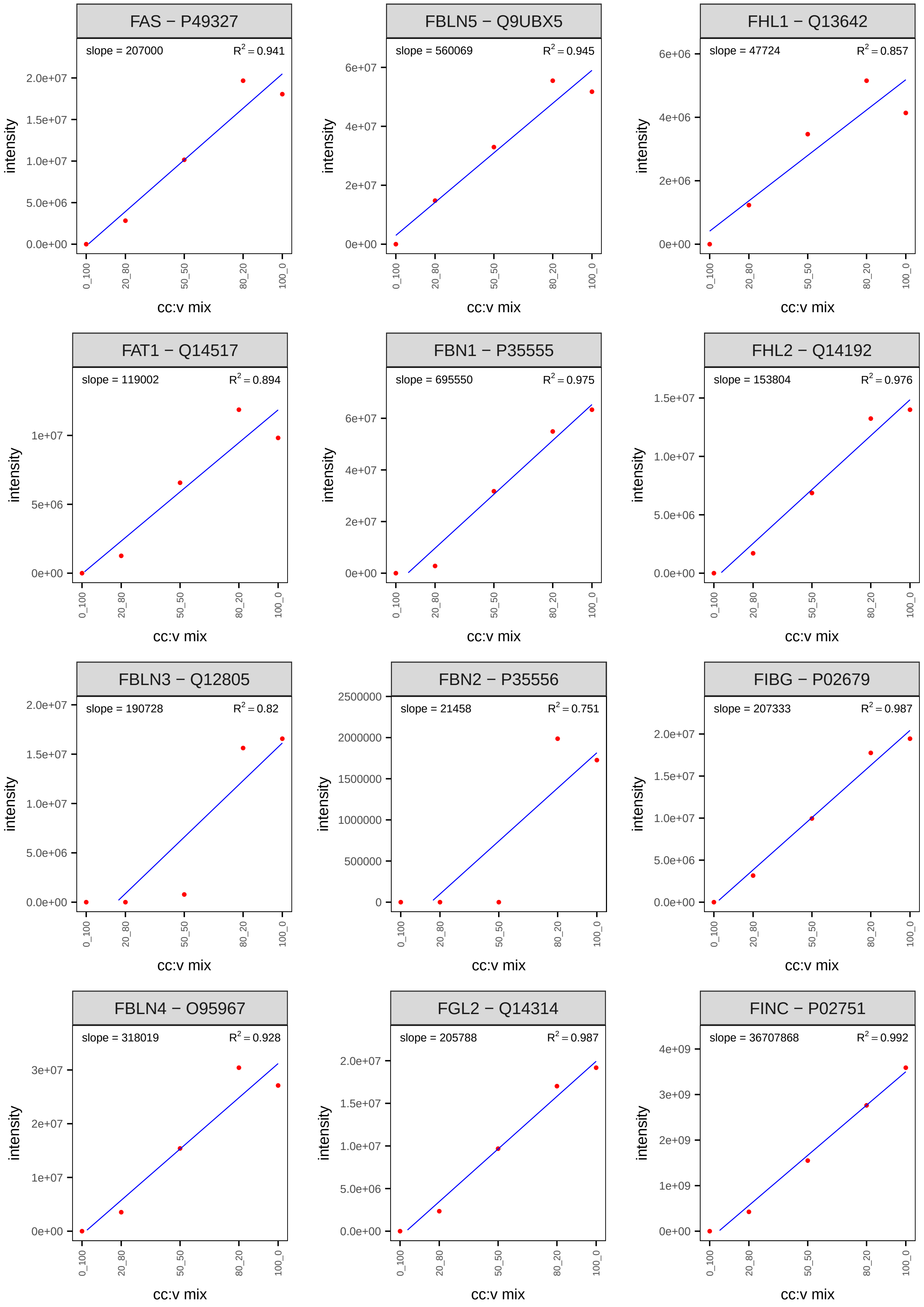
FAS − P49327
slope = 207000
R² = 0.941
FBLN5 − Q9UBX5
slope = 560069
R² = 0.945
FHL1 − Q13642
slope = 47724
R² = 0.857
FAT1 − Q14517
slope = 119002
R² = 0.894
FBN1 − P35555
slope = 695550
R² = 0.975
FHL2 − Q14192
slope = 153804
R² = 0.976
FBLN3 − Q12805
slope = 190728
R² = 0.82
FBN2 − P35556
slope = 21458
R² = 0.751
FIBG − P02679
slope = 207333
R² = 0.987
FBLN4 − O95967
slope = 318019
R² = 0.928
FGL2 − Q14314
slope = 205788
R² = 0.987
FINC − P02751
slope = 36707868
R² = 0.992
intensity
cc:v mix
0_100
20_80
50_50
80_20
100_0

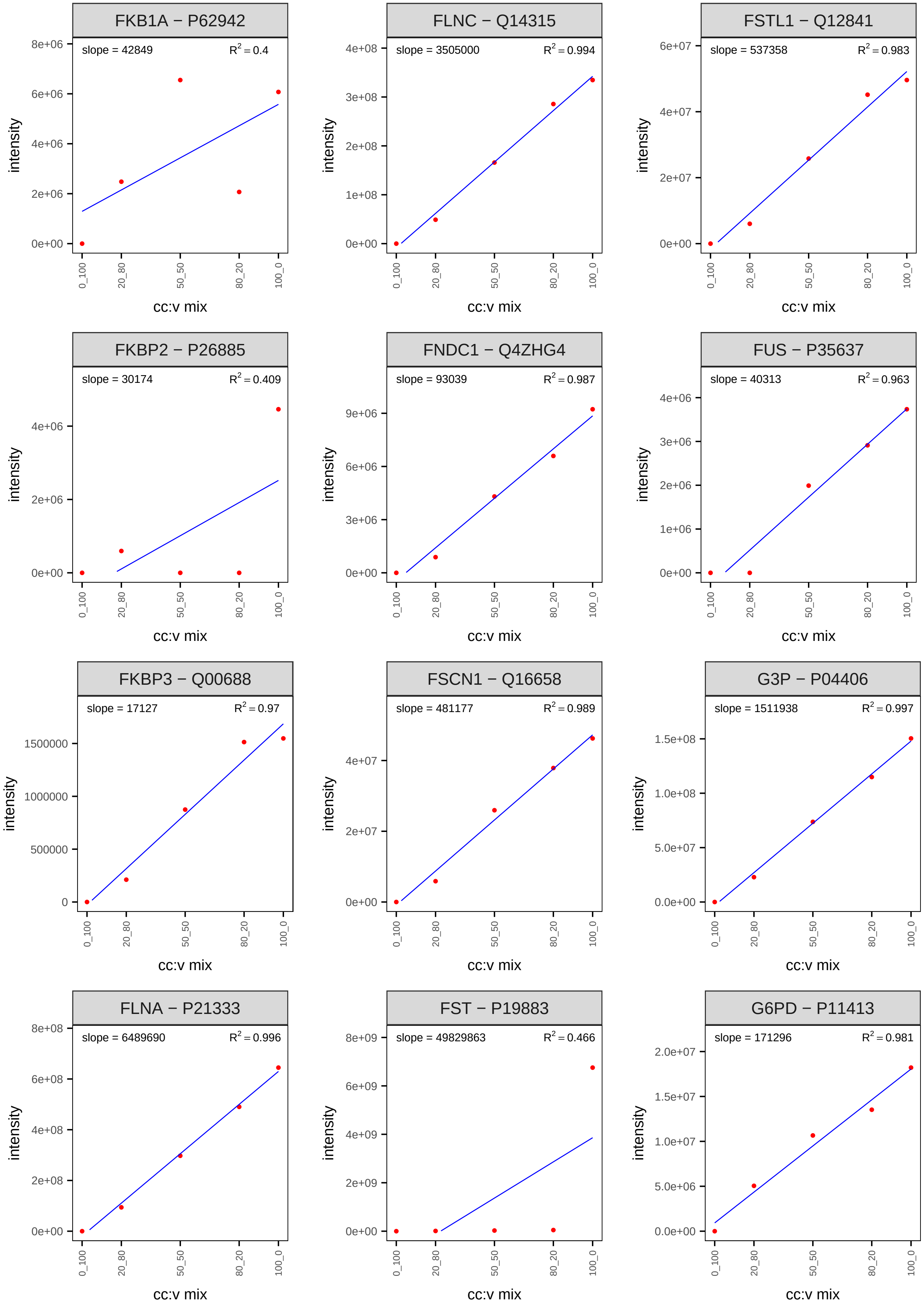
FKB1A − P62942
slope = 42849
R2 = 0.4
FLNC − Q14315
slope = 3505000
R2 = 0.994
FSTL1 − Q12841
slope = 537358
R2 = 0.983
FKBP2 − P26885
slope = 30174
R2 = 0.409
FNDC1 − Q4ZHG4
slope = 93039
R2 = 0.987
FUS − P35637
slope = 40313
R2 = 0.963
FKBP3 − Q00688
slope = 17127
R2 = 0.97
FSCN1 − Q16658
slope = 481177
R2 = 0.989
G3P − P04406
slope = 1511938
R2 = 0.997
FLNA − P21333
slope = 6489690
R2 = 0.996
FST − P19883
slope = 49829863
R2 = 0.466
G6PD − P11413
slope = 171296
R2 = 0.981
intensity
cc:v mix
0_100
20_80
50_50
80_20
100_0

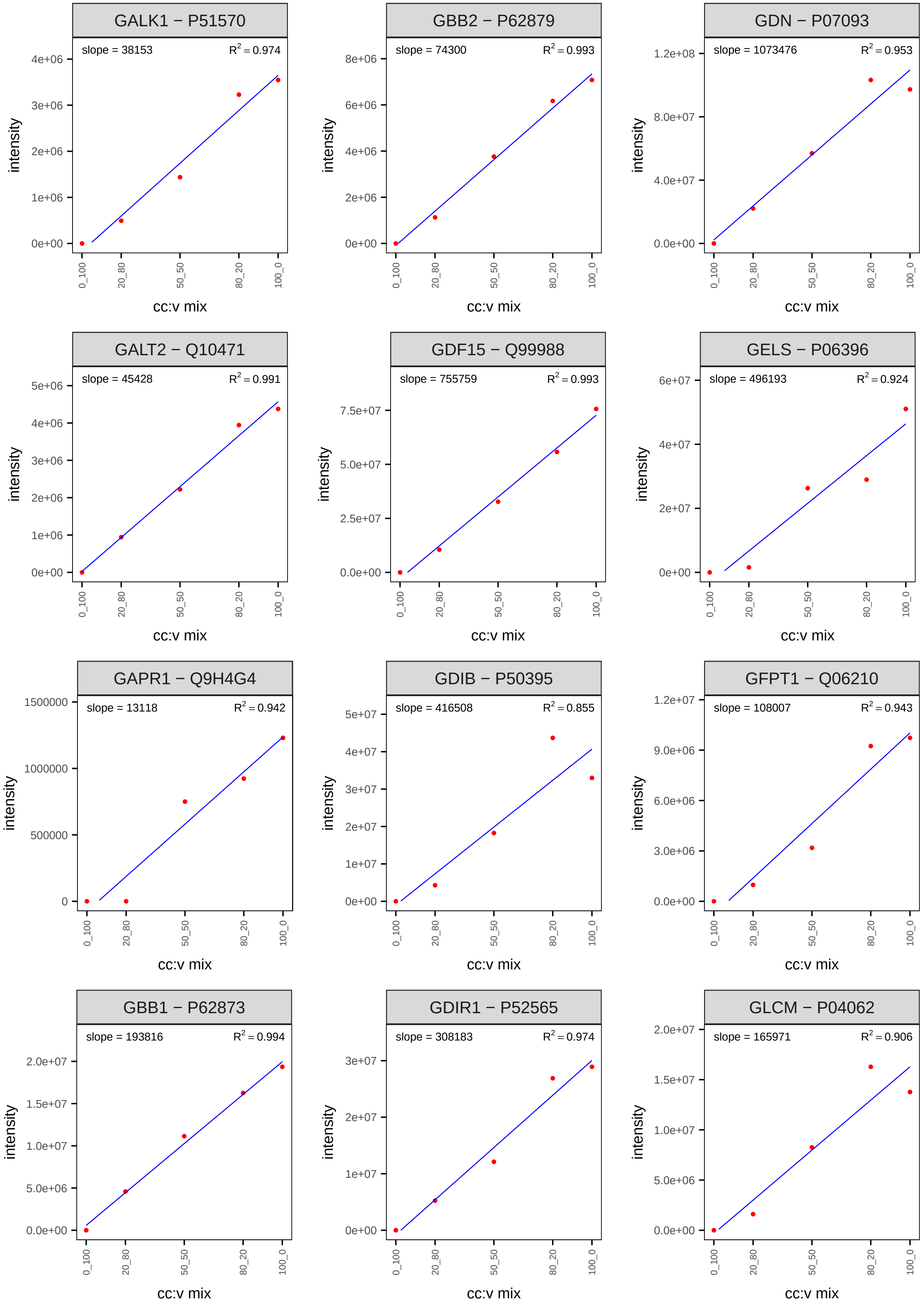
GALK1 − P51570
slope = 38153
$R^2 = 0.974$
GBB2 − P62879
slope = 74300
$R^2 = 0.993$
GDN − P07093
slope = 1073476
$R^2 = 0.953$
GALT2 − Q10471
slope = 45428
$R^2 = 0.991$
GDF15 − Q99988
slope = 755759
$R^2 = 0.993$
GELS − P06396
slope = 496193
$R^2 = 0.924$
GAPR1 − Q9H4G4
slope = 13118
$R^2 = 0.942$
GDIB − P50395
slope = 416508
$R^2 = 0.855$
GFPT1 − Q06210
slope = 108007
$R^2 = 0.943$
GBB1 − P62873
slope = 193816
$R^2 = 0.994$
GDIR1 − P52565
slope = 308183
$R^2 = 0.974$
GLCM − P04062
slope = 165971
$R^2 = 0.906$
intensity
cc:v mix
0_100
20_80
50_50
80_20
100_0

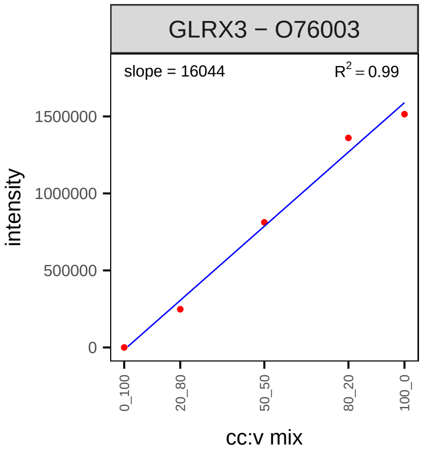
GLRX3 − O76003
slope = 16044
$R^2 = 0.99$
intensity
1500000
1000000
500000
0
0_100
20_80
50_50
80_20
100_0
cc:v mix

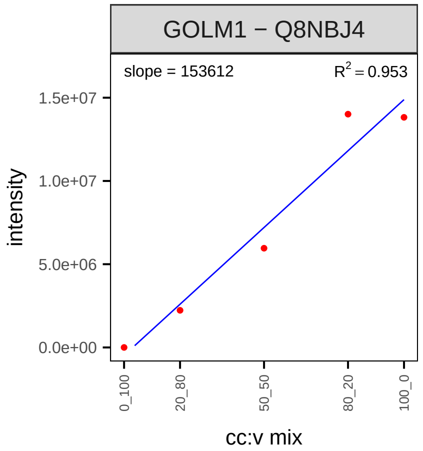
GOLM1 − Q8NBJ4
slope = 153612
$R^2 = 0.953$
intensity
1.5e+07
1.0e+07
5.0e+06
0.0e+00
0_100
20_80
50_50
80_20
100_0
cc:v mix

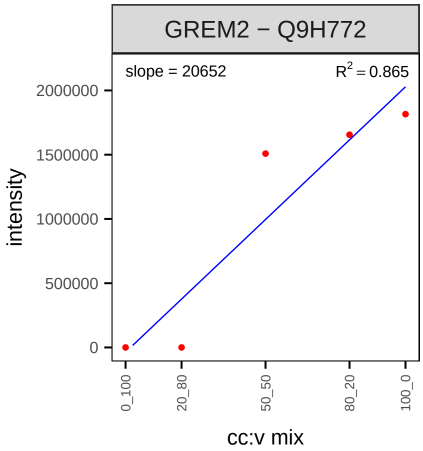
GREM2 − Q9H772
slope = 20652
$R^2 = 0.865$
intensity
2000000
1500000
1000000
500000
0
0_100
20_80
50_50
80_20
100_0
cc:v mix

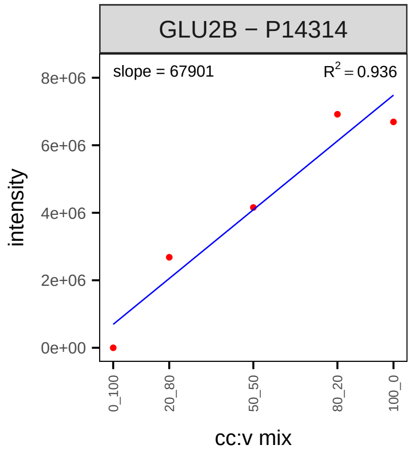
GLU2B − P14314
slope = 67901
$R^2 = 0.936$
intensity
8e+06
6e+06
4e+06
2e+06
0e+00
0_100
20_80
50_50
80_20
100_0
cc:v mix

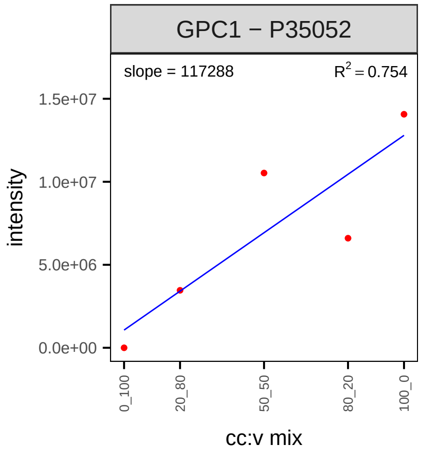
GPC1 − P35052
slope = 117288
$R^2 = 0.754$
intensity
1.5e+07
1.0e+07
5.0e+06
0.0e+00
0_100
20_80
50_50
80_20
100_0
cc:v mix

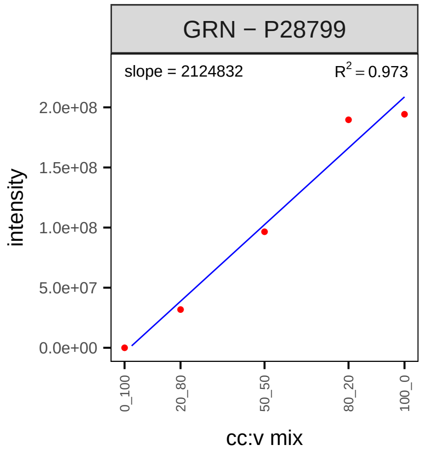
GRN − P28799
slope = 2124832
$R^2 = 0.973$
intensity
2.0e+08
1.5e+08
1.0e+08
5.0e+07
0.0e+00
0_100
20_80
50_50
80_20
100_0
cc:v mix

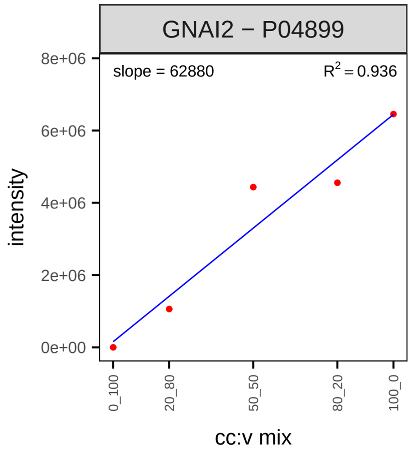
GNAI2 − P04899
slope = 62880
$R^2 = 0.936$
intensity
8e+06
6e+06
4e+06
2e+06
0e+00
0_100
20_80
50_50
80_20
100_0
cc:v mix

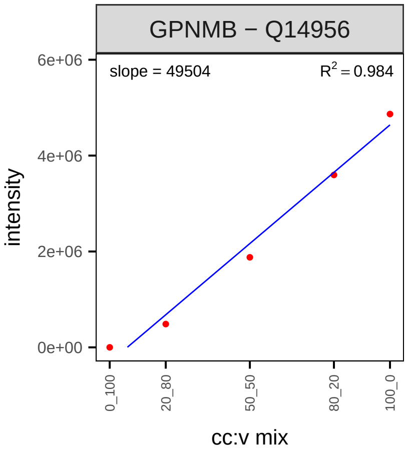
GPNMB − Q14956
slope = 49504
$R^2 = 0.984$
intensity
6e+06
4e+06
2e+06
0e+00
0_100
20_80
50_50
80_20
100_0
cc:v mix

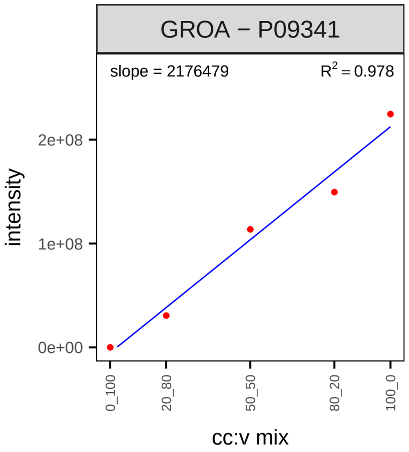
GROA − P09341
slope = 2176479
$R^2 = 0.978$
intensity
2e+08
1e+08
0e+00
0_100
20_80
50_50
80_20
100_0
cc:v mix

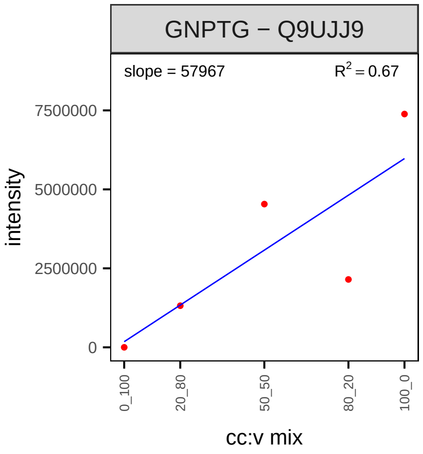
GNPTG − Q9UJJ9
slope = 57967
$R^2 = 0.67$
intensity
7500000
5000000
2500000
0
0_100
20_80
50_50
80_20
100_0
cc:v mix

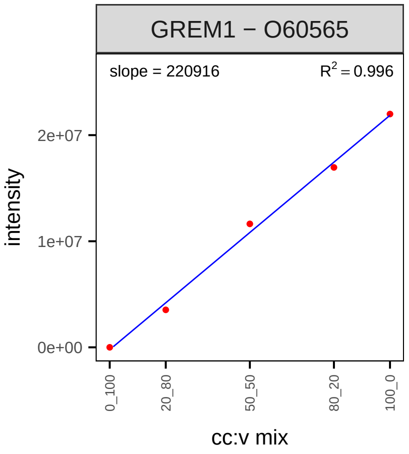
GREM1 − O60565
slope = 220916
$R^2 = 0.996$
intensity
2e+07
1e+07
0e+00
0_100
20_80
50_50
80_20
100_0
cc:v mix

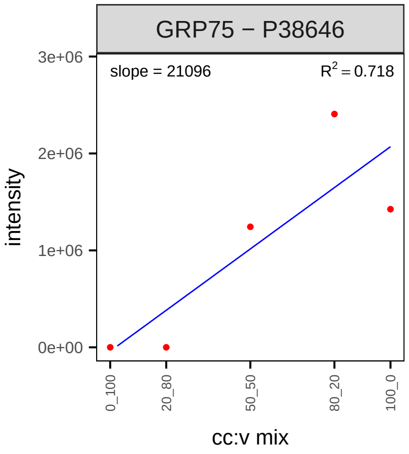
GRP75 − P38646
slope = 21096
$R^2 = 0.718$
intensity
3e+06
2e+06
1e+06
0e+00
0_100
20_80
50_50
80_20
100_0
cc:v mix

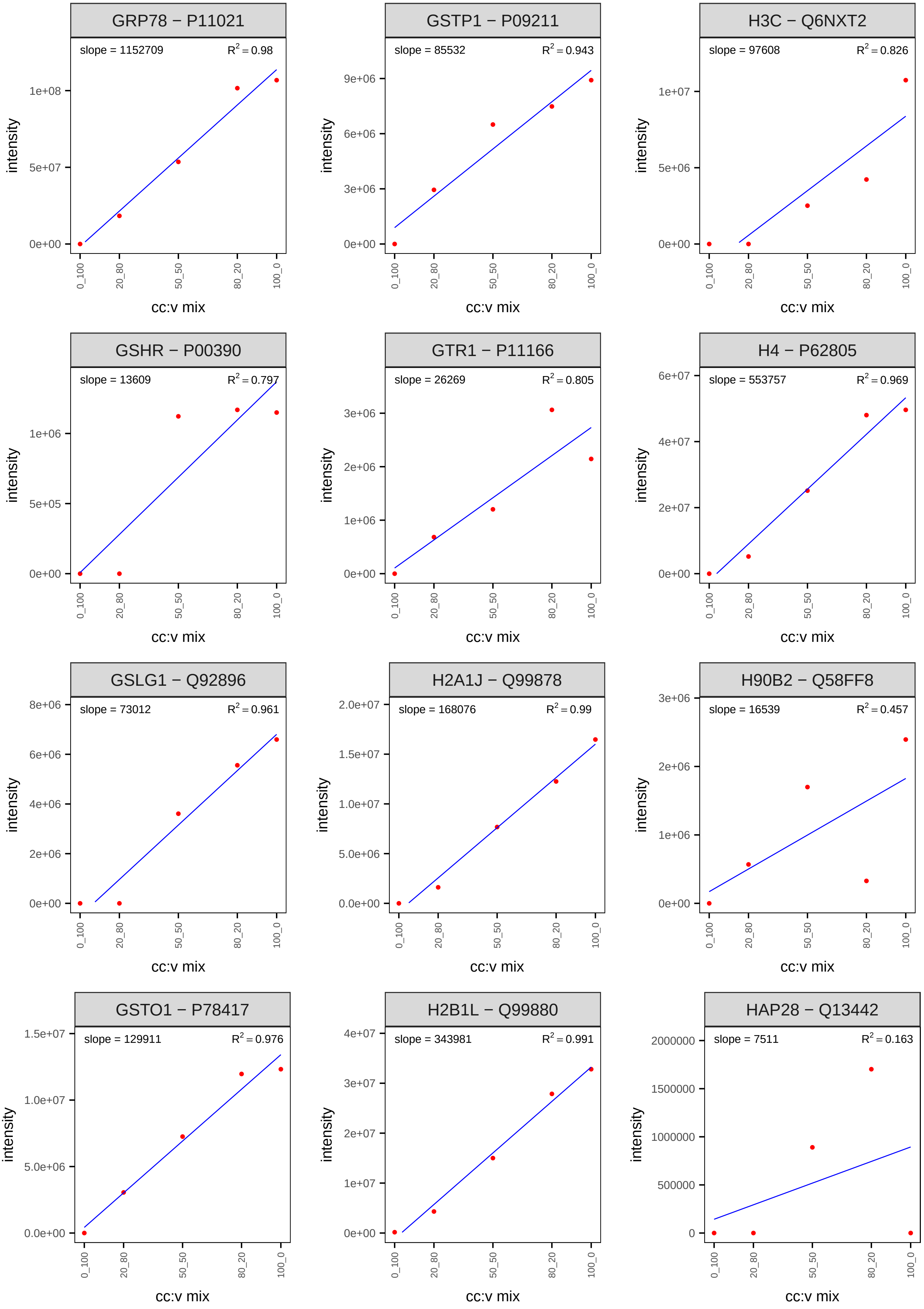
GRP78 − P11021
slope = 1152709
R² = 0.98
GSTP1 − P09211
slope = 85532
R² = 0.943
H3C − Q6NXT2
slope = 97608
R² = 0.826
GSHR − P00390
slope = 13609
R² = 0.797
GTR1 − P11166
slope = 26269
R² = 0.805
H4 − P62805
slope = 553757
R² = 0.969
GSLG1 − Q92896
slope = 73012
R² = 0.961
H2A1J − Q99878
slope = 168076
R² = 0.99
H90B2 − Q58FF8
slope = 16539
R² = 0.457
GSTO1 − P78417
slope = 129911
R² = 0.976
H2B1L − Q99880
slope = 343981
R² = 0.991
HAP28 − Q13442
slope = 7511
R² = 0.163
intensity
cc:v mix
0_100
20_80
50_50
80_20
100_0

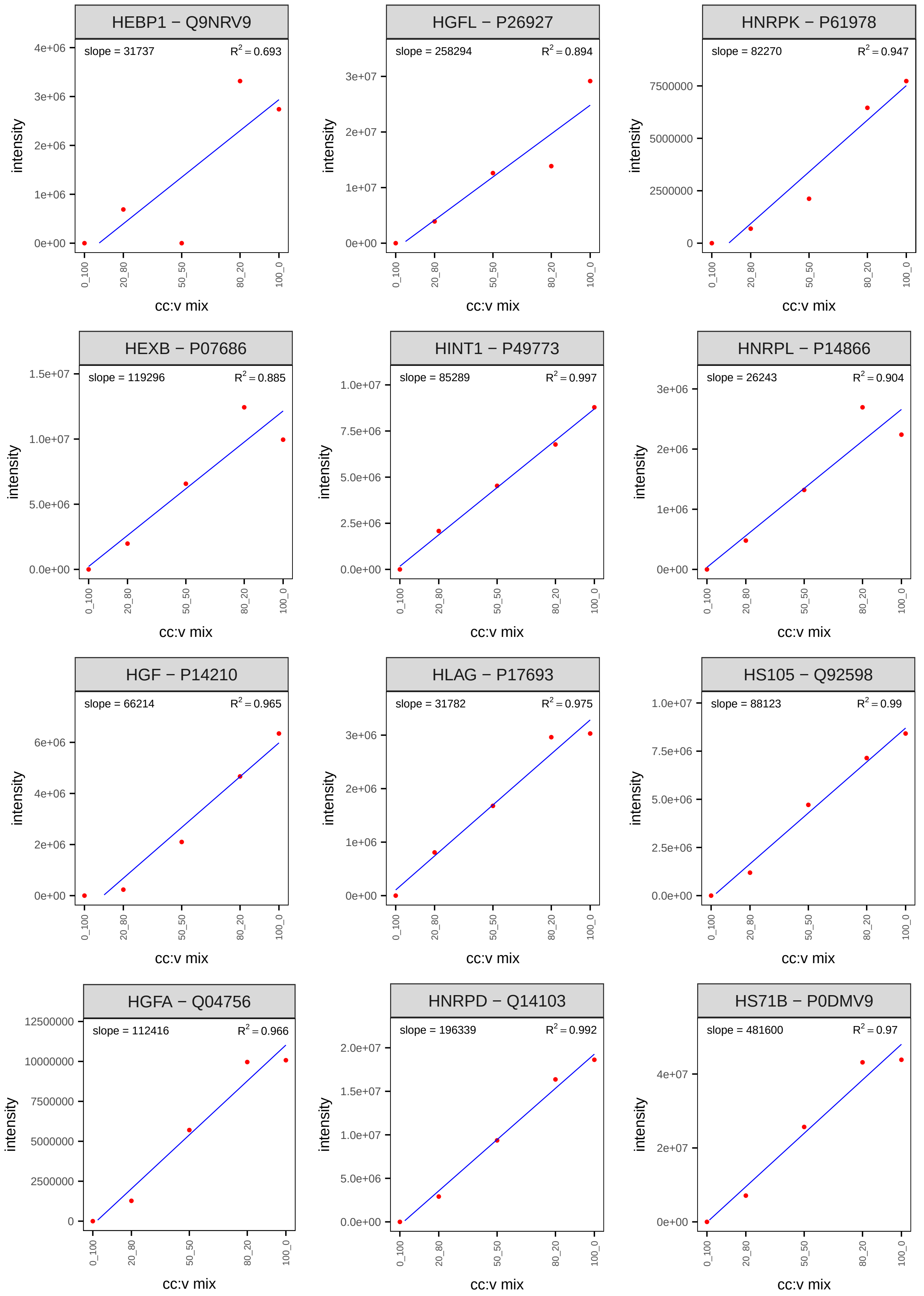
HEBP1 − Q9NRV9
slope = 31737
R2 = 0.693
HGFL − P26927
slope = 258294
R2 = 0.894
HNRPK − P61978
slope = 82270
R2 = 0.947
HEXB − P07686
slope = 119296
R2 = 0.885
HINT1 − P49773
slope = 85289
R2 = 0.997
HNRPL − P14866
slope = 26243
R2 = 0.904
HGF − P14210
slope = 66214
R2 = 0.965
HLAG − P17693
slope = 31782
R2 = 0.975
HS105 − Q92598
slope = 88123
R2 = 0.99
HGFA − Q04756
slope = 112416
R2 = 0.966
HNRPD − Q14103
slope = 196339
R2 = 0.992
HS71B − P0DMV9
slope = 481600
R2 = 0.97
intensity
cc:v mix
0_100
20_80
50_50
80_20
100_0

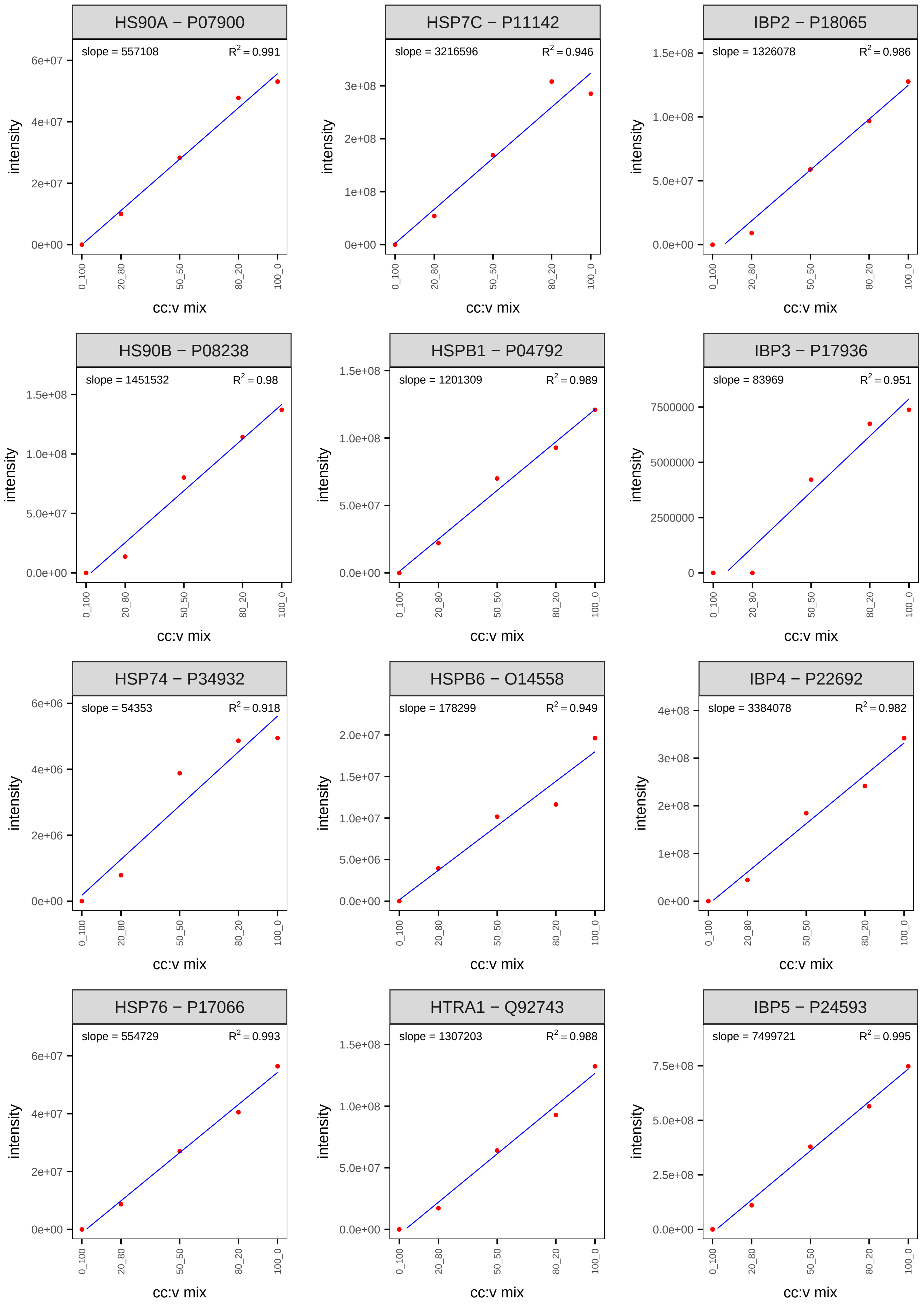
HS90A − P07900
slope = 557108
R2 = 0.991
HSP7C − P11142
slope = 3216596
R2 = 0.946
IBP2 − P18065
slope = 1326078
R2 = 0.986
HS90B − P08238
slope = 1451532
R2 = 0.98
HSPB1 − P04792
slope = 1201309
R2 = 0.989
IBP3 − P17936
slope = 83969
R2 = 0.951
HSP74 − P34932
slope = 54353
R2 = 0.918
HSPB6 − O14558
slope = 178299
R2 = 0.949
IBP4 − P22692
slope = 3384078
R2 = 0.982
HSP76 − P17066
slope = 554729
R2 = 0.993
HTRA1 − Q92743
slope = 1307203
R2 = 0.988
IBP5 − P24593
slope = 7499721
R2 = 0.995
intensity
cc:v mix
0_100
20_80
50_50
80_20
100_0

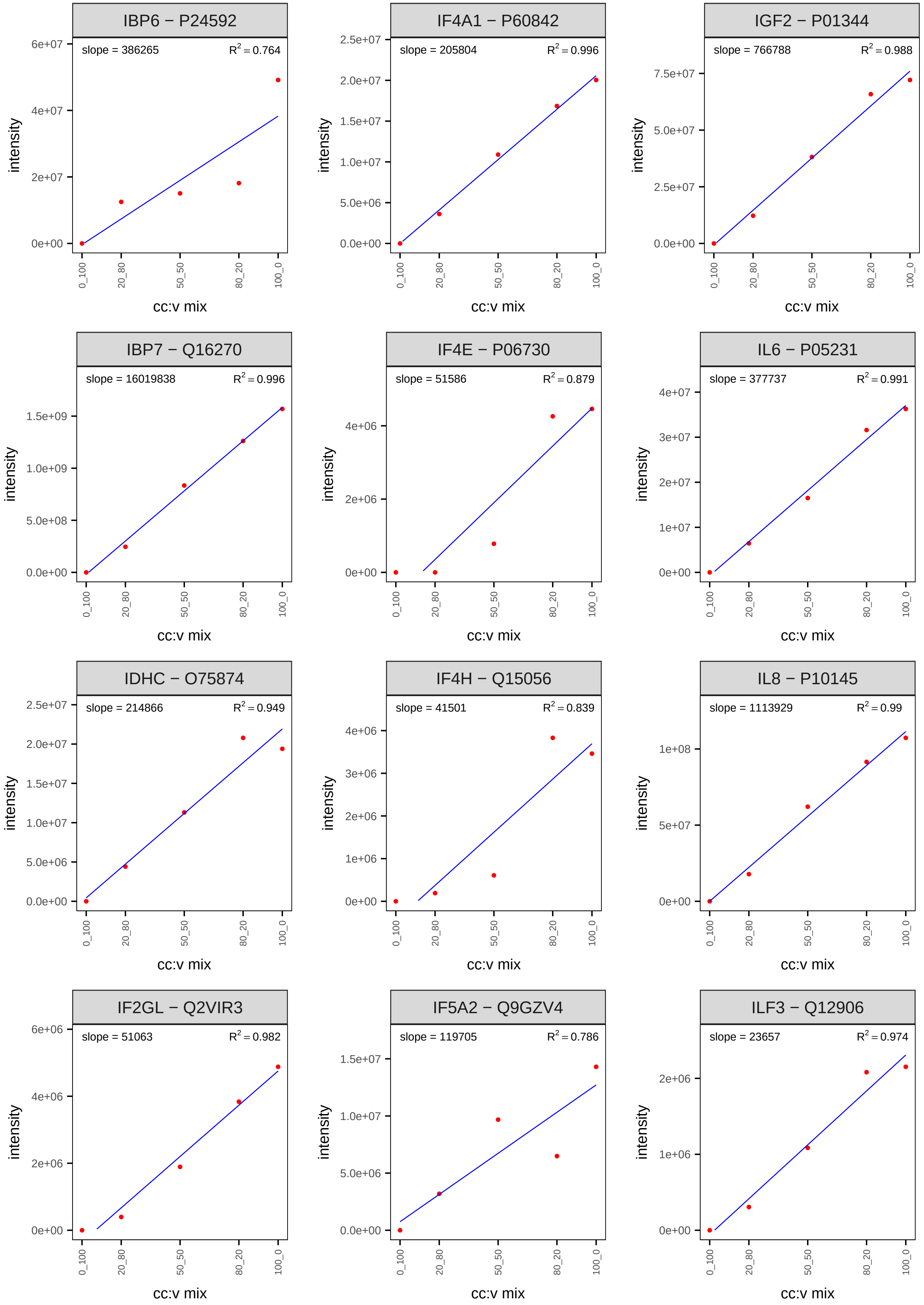
IBP6 − P24592
slope = 386265
R2 = 0.764
IF4A1 − P60842
slope = 205804
R2 = 0.996
IGF2 − P01344
slope = 766788
R2 = 0.988
IBP7 − Q16270
slope = 16019838
R2 = 0.996
IF4E − P06730
slope = 51586
R2 = 0.879
IL6 − P05231
slope = 377737
R2 = 0.991
IDHC − O75874
slope = 214866
R2 = 0.949
IF4H − Q15056
slope = 41501
R2 = 0.839
IL8 − P10145
slope = 1113929
R2 = 0.99
IF2GL − Q2VIR3
slope = 51063
R2 = 0.982
IF5A2 − Q9GZV4
slope = 119705
R2 = 0.786
ILF3 − Q12906
slope = 23657
R2 = 0.974
intensity
cc:v mix
0_100
20_80
50_50
80_20
100_0

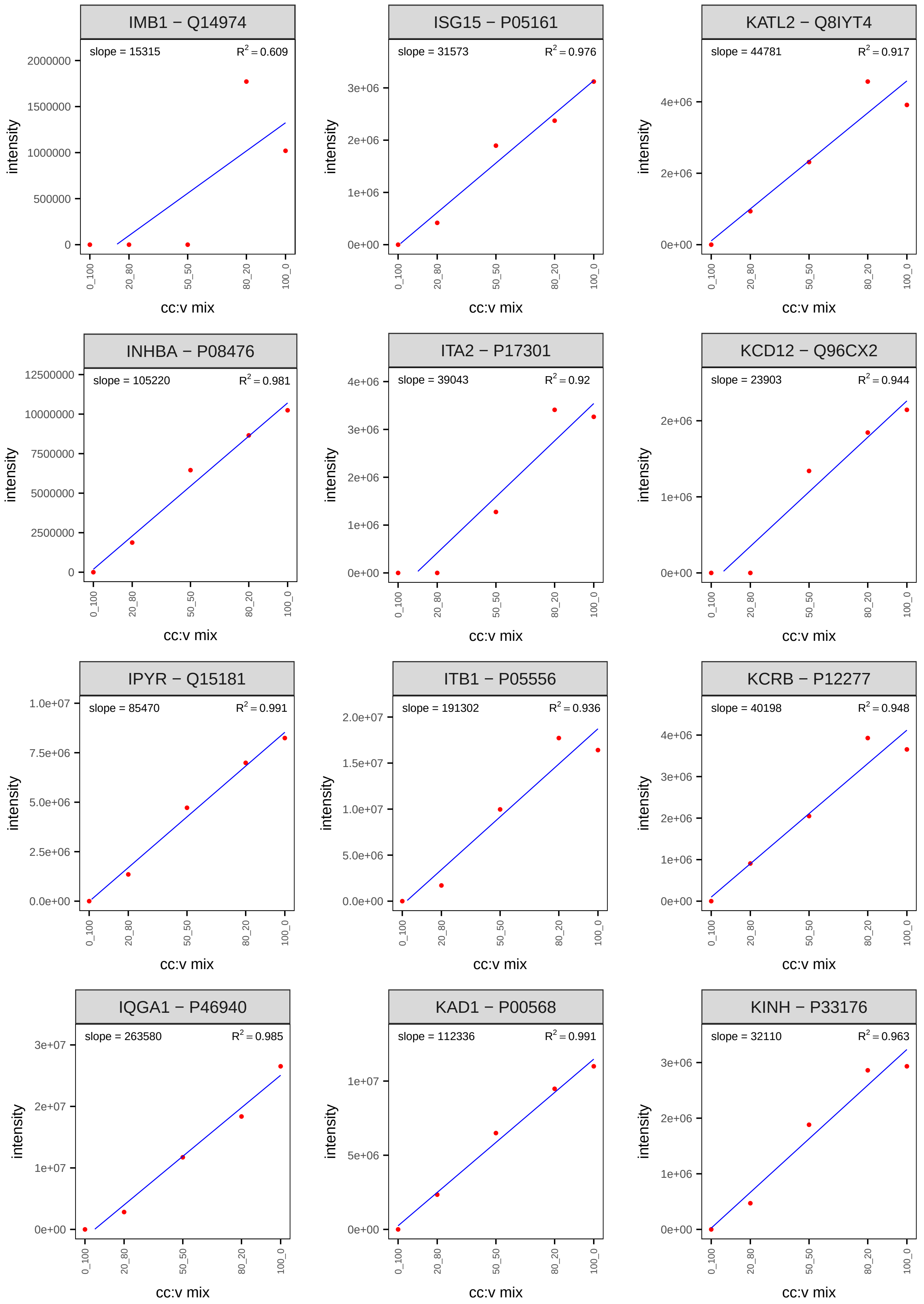
IMB1 − Q14974
slope = 15315
$R^2 = 0.609$
ISG15 − P05161
slope = 31573
$R^2 = 0.976$
KATL2 − Q8IYT4
slope = 44781
$R^2 = 0.917$
INHBA − P08476
slope = 105220
$R^2 = 0.981$
ITA2 − P17301
slope = 39043
$R^2 = 0.92$
KCD12 − Q96CX2
slope = 23903
$R^2 = 0.944$
IPYR − Q15181
slope = 85470
$R^2 = 0.991$
ITB1 − P05556
slope = 191302
$R^2 = 0.936$
KCRB − P12277
slope = 40198
$R^2 = 0.948$
IQGA1 − P46940
slope = 263580
$R^2 = 0.985$
KAD1 − P00568
slope = 112336
$R^2 = 0.991$
KINH − P33176
slope = 32110
$R^2 = 0.963$
intensity
cc:v mix
0_100
20_80
50_50
80_20
100_0

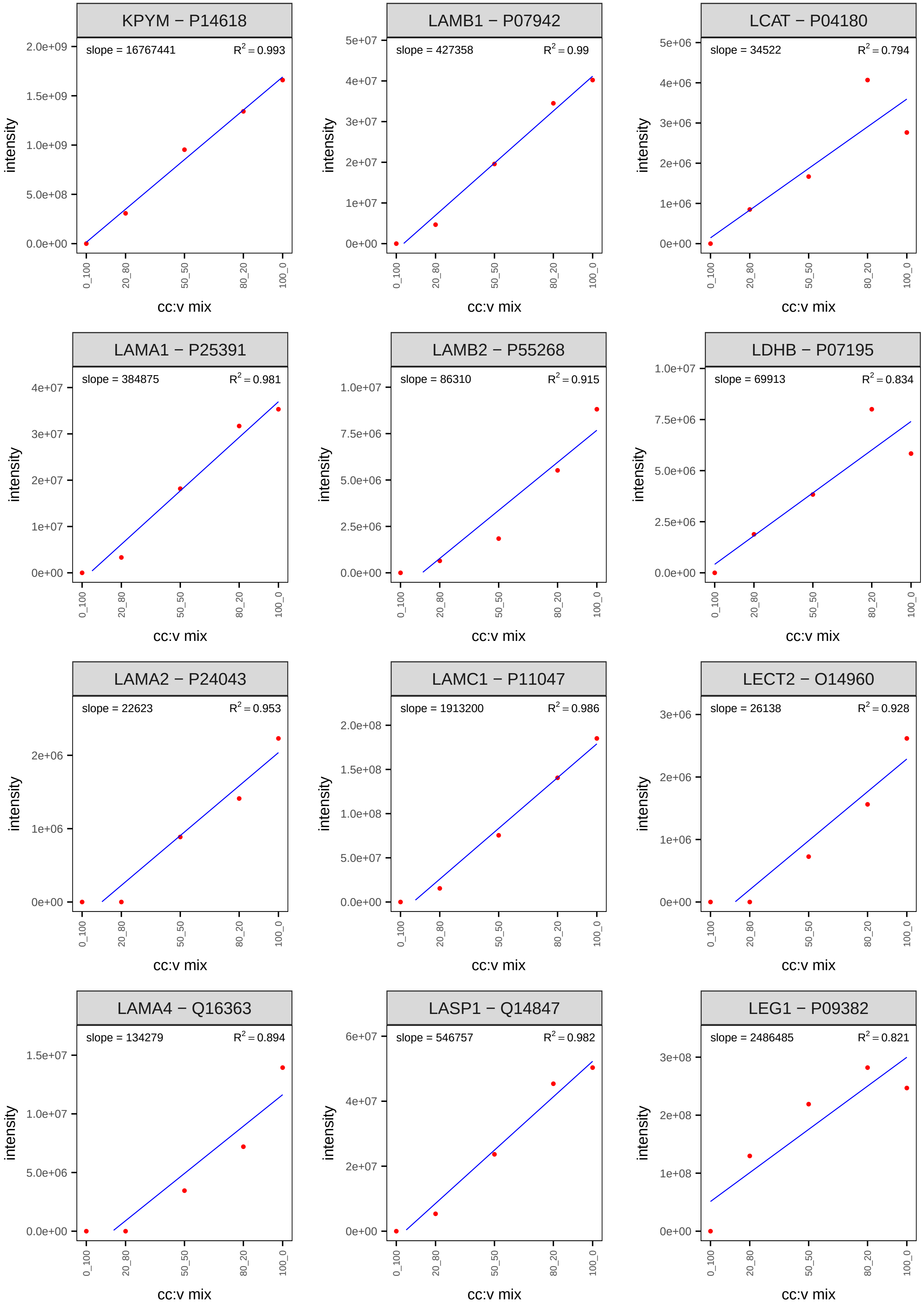
KPYM − P14618
slope = 16767441
$R^2 = 0.993$
LAMB1 − P07942
slope = 427358
$R^2 = 0.99$
LCAT − P04180
slope = 34522
$R^2 = 0.794$
LAMA1 − P25391
slope = 384875
$R^2 = 0.981$
LAMB2 − P55268
slope = 86310
$R^2 = 0.915$
LDHB − P07195
slope = 69913
$R^2 = 0.834$
LAMA2 − P24043
slope = 22623
$R^2 = 0.953$
LAMC1 − P11047
slope = 1913200
$R^2 = 0.986$
LECT2 − O14960
slope = 26138
$R^2 = 0.928$
LAMA4 − Q16363
slope = 134279
$R^2 = 0.894$
LASP1 − Q14847
slope = 546757
$R^2 = 0.982$
LEG1 − P09382
slope = 2486485
$R^2 = 0.821$
intensity
cc:v mix
0_100
20_80
50_50
80_20
100_0

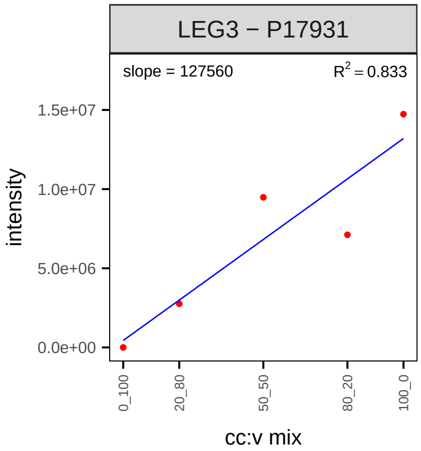
LEG3 − P17931
slope = 127560
$R^2 = 0.833$
intensity
1.5e+07
1.0e+07
5.0e+06
0.0e+00
0_100
20_80
50_50
80_20
100_0
cc:v mix

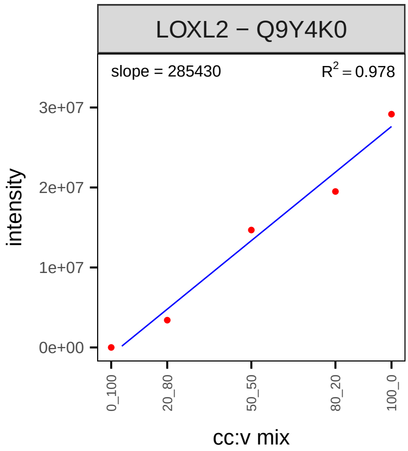
LOXL2 − Q9Y4K0
slope = 285430
$R^2 = 0.978$
intensity
3e+07
2e+07
1e+07
0e+00
0_100
20_80
50_50
80_20
100_0
cc:v mix

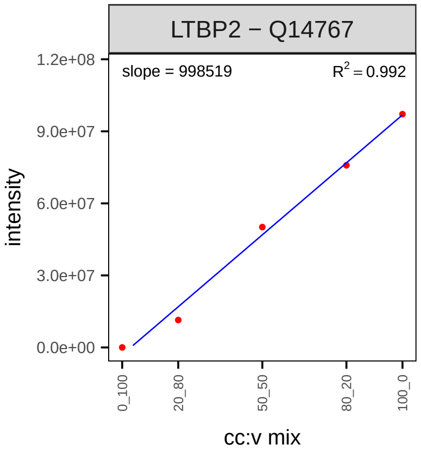
LTBP2 − Q14767
slope = 998519
$R^2 = 0.992$
intensity
1.2e+08
9.0e+07
6.0e+07
3.0e+07
0.0e+00
0_100
20_80
50_50
80_20
100_0
cc:v mix

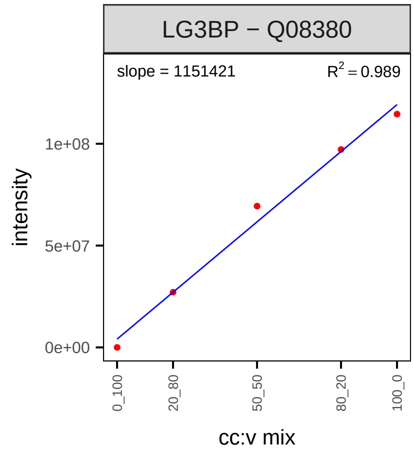
LG3BP − Q08380
slope = 1151421
$R^2 = 0.989$
intensity
1e+08
5e+07
0e+00
0_100
20_80
50_50
80_20
100_0
cc:v mix

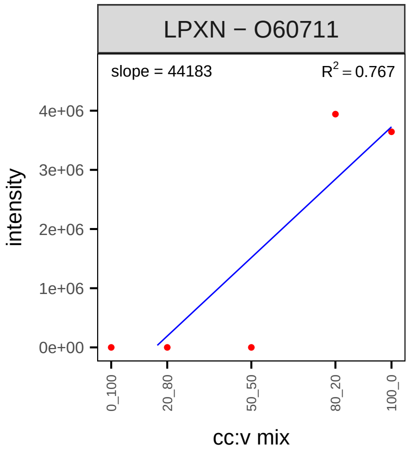
LPXN − O60711
slope = 44183
$R^2 = 0.767$
intensity
4e+06
3e+06
2e+06
1e+06
0e+00
0_100
20_80
50_50
80_20
100_0
cc:v mix

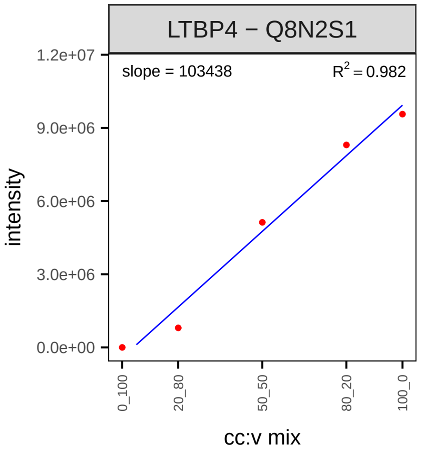
LTBP4 − Q8N2S1
slope = 103438
$R^2 = 0.982$
intensity
1.2e+07
9.0e+06
6.0e+06
3.0e+06
0.0e+00
0_100
20_80
50_50
80_20
100_0
cc:v mix

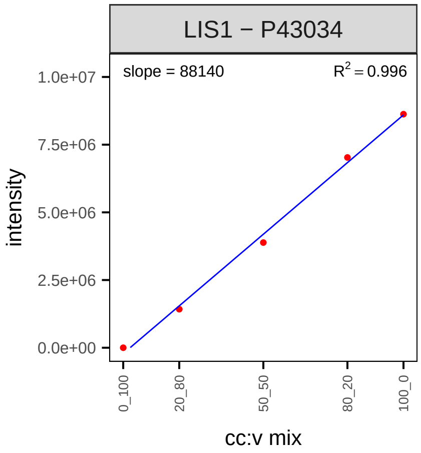
LIS1 − P43034
slope = 88140
$R^2 = 0.996$
intensity
1.0e+07
7.5e+06
5.0e+06
2.5e+06
0.0e+00
0_100
20_80
50_50
80_20
100_0
cc:v mix

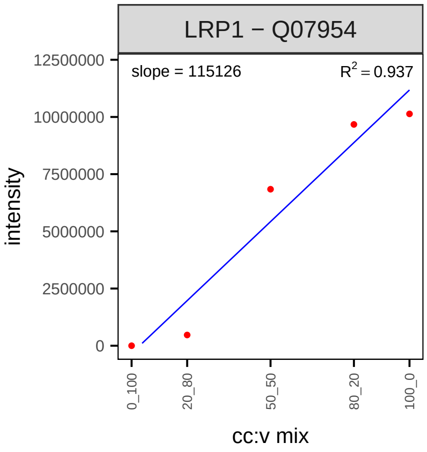
LRP1 − Q07954
slope = 115126
$R^2 = 0.937$
intensity
12500000
10000000
7500000
5000000
2500000
0
0_100
20_80
50_50
80_20
100_0
cc:v mix

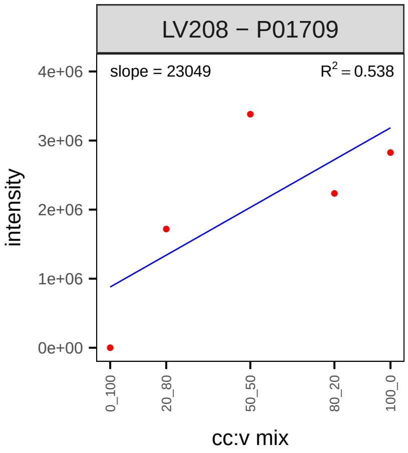
LV208 − P01709
slope = 23049
$R^2 = 0.538$
intensity
4e+06
3e+06
2e+06
1e+06
0e+00
0_100
20_80
50_50
80_20
100_0
cc:v mix

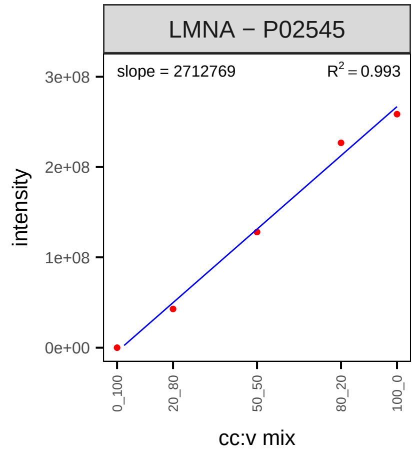
LMNA − P02545
slope = 2712769
$R^2 = 0.993$
intensity
3e+08
2e+08
1e+08
0e+00
0_100
20_80
50_50
80_20
100_0
cc:v mix

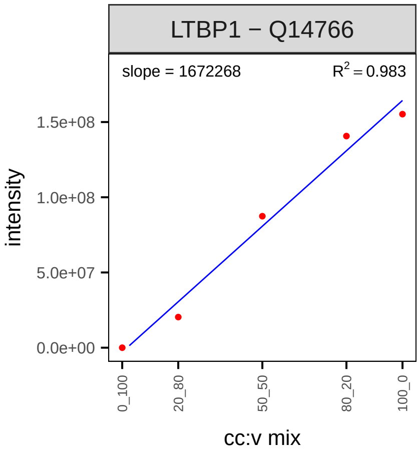
LTBP1 − Q14766
slope = 1672268
$R^2 = 0.983$
intensity
1.5e+08
1.0e+08
5.0e+07
0.0e+00
0_100
20_80
50_50
80_20
100_0
cc:v mix

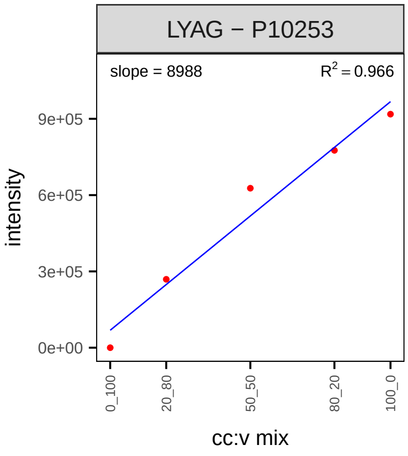
LYAG − P10253
slope = 8988
$R^2 = 0.966$
intensity
9e+05
6e+05
3e+05
0e+00
0_100
20_80
50_50
80_20
100_0
cc:v mix

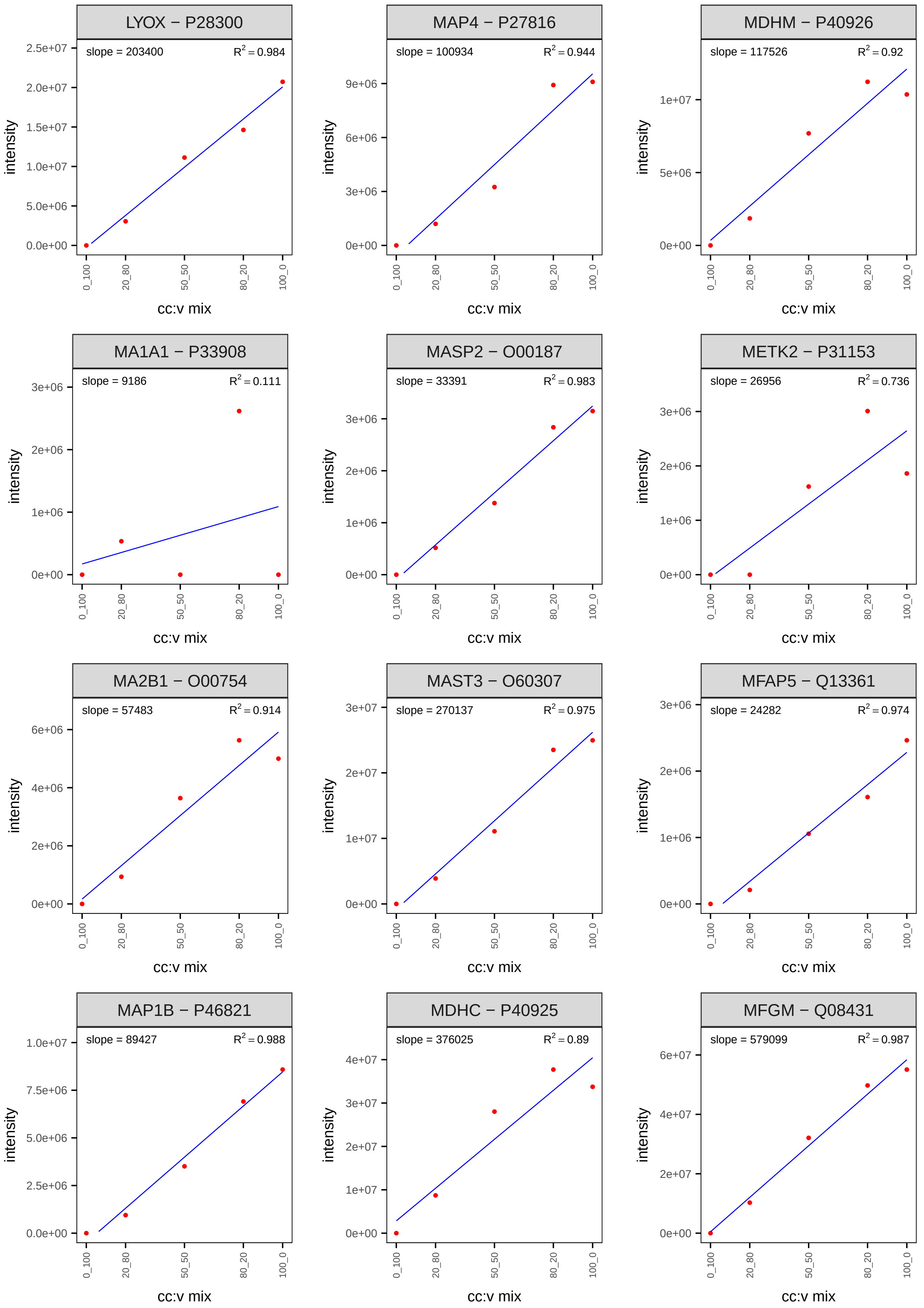
LYOX − P28300
slope = 203400
R^2 = 0.984
MAP4 − P27816
slope = 100934
R^2 = 0.944
MDHM − P40926
slope = 117526
R^2 = 0.92
MA1A1 − P33908
slope = 9186
R^2 = 0.111
MASP2 − O00187
slope = 33391
R^2 = 0.983
METK2 − P31153
slope = 26956
R^2 = 0.736
MA2B1 − O00754
slope = 57483
R^2 = 0.914
MAST3 − O60307
slope = 270137
R^2 = 0.975
MFAP5 − Q13361
slope = 24282
R^2 = 0.974
MAP1B − P46821
slope = 89427
R^2 = 0.988
MDHC − P40925
slope = 376025
R^2 = 0.89
MFGM − Q08431
slope = 579099
R^2 = 0.987
intensity
cc:v mix
0_100
20_80
50_50
80_20
100_0

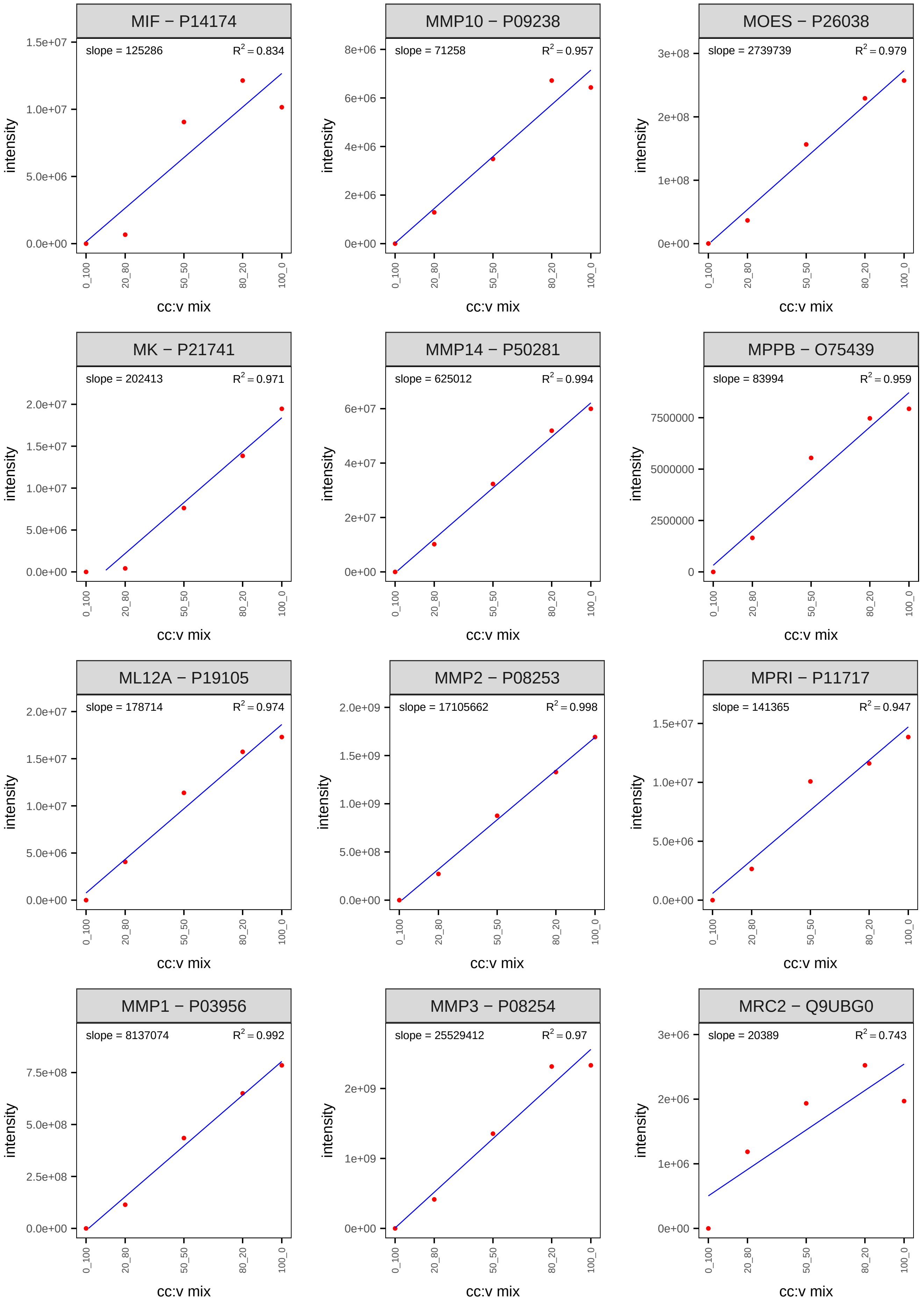
MIF − P14174
slope = 125286
$R^2 = 0.834$
MMP10 − P09238
slope = 71258
$R^2 = 0.957$
MOES − P26038
slope = 2739739
$R^2 = 0.979$
MK − P21741
slope = 202413
$R^2 = 0.971$
MMP14 − P50281
slope = 625012
$R^2 = 0.994$
MPPB − O75439
slope = 83994
$R^2 = 0.959$
ML12A − P19105
slope = 178714
$R^2 = 0.974$
MMP2 − P08253
slope = 17105662
$R^2 = 0.998$
MPRI − P11717
slope = 141365
$R^2 = 0.947$
MMP1 − P03956
slope = 8137074
$R^2 = 0.992$
MMP3 − P08254
slope = 25529412
$R^2 = 0.97$
MRC2 − Q9UBG0
slope = 20389
$R^2 = 0.743$
intensity
cc:v mix
0_100
20_80
50_50
80_20
100_0

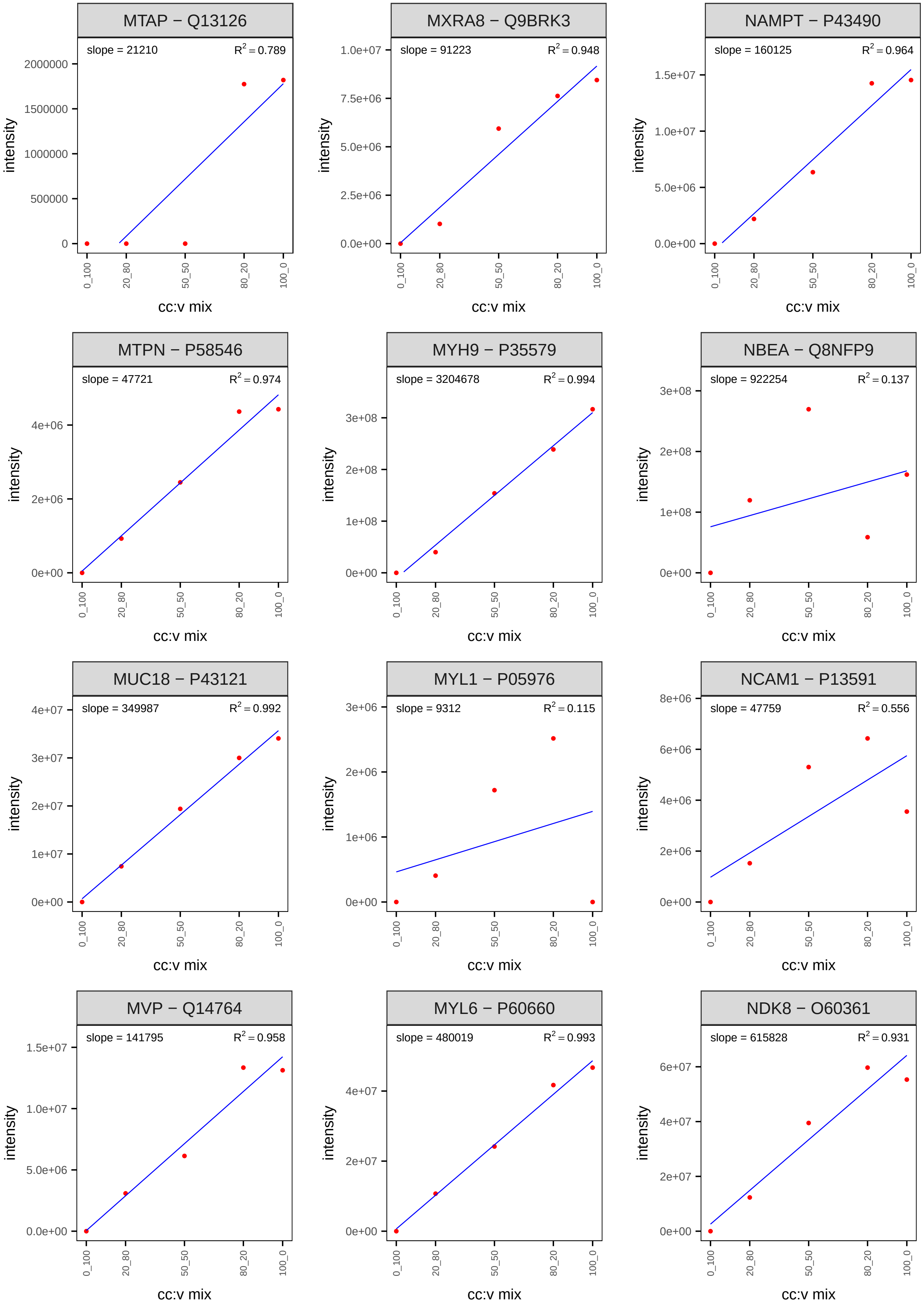
MTAP − Q13126
slope = 21210
$R^2 = 0.789$
MXRA8 − Q9BRK3
slope = 91223
$R^2 = 0.948$
NAMPT − P43490
slope = 160125
$R^2 = 0.964$
MTPN − P58546
slope = 47721
$R^2 = 0.974$
MYH9 − P35579
slope = 3204678
$R^2 = 0.994$
NBEA − Q8NFP9
slope = 922254
$R^2 = 0.137$
MUC18 − P43121
slope = 349987
$R^2 = 0.992$
MYL1 − P05976
slope = 9312
$R^2 = 0.115$
NCAM1 − P13591
slope = 47759
$R^2 = 0.556$
MVP − Q14764
slope = 141795
$R^2 = 0.958$
MYL6 − P60660
slope = 480019
$R^2 = 0.993$
NDK8 − O60361
slope = 615828
$R^2 = 0.931$
intensity
cc:v mix
0_100
20_80
50_50
80_20
100_0

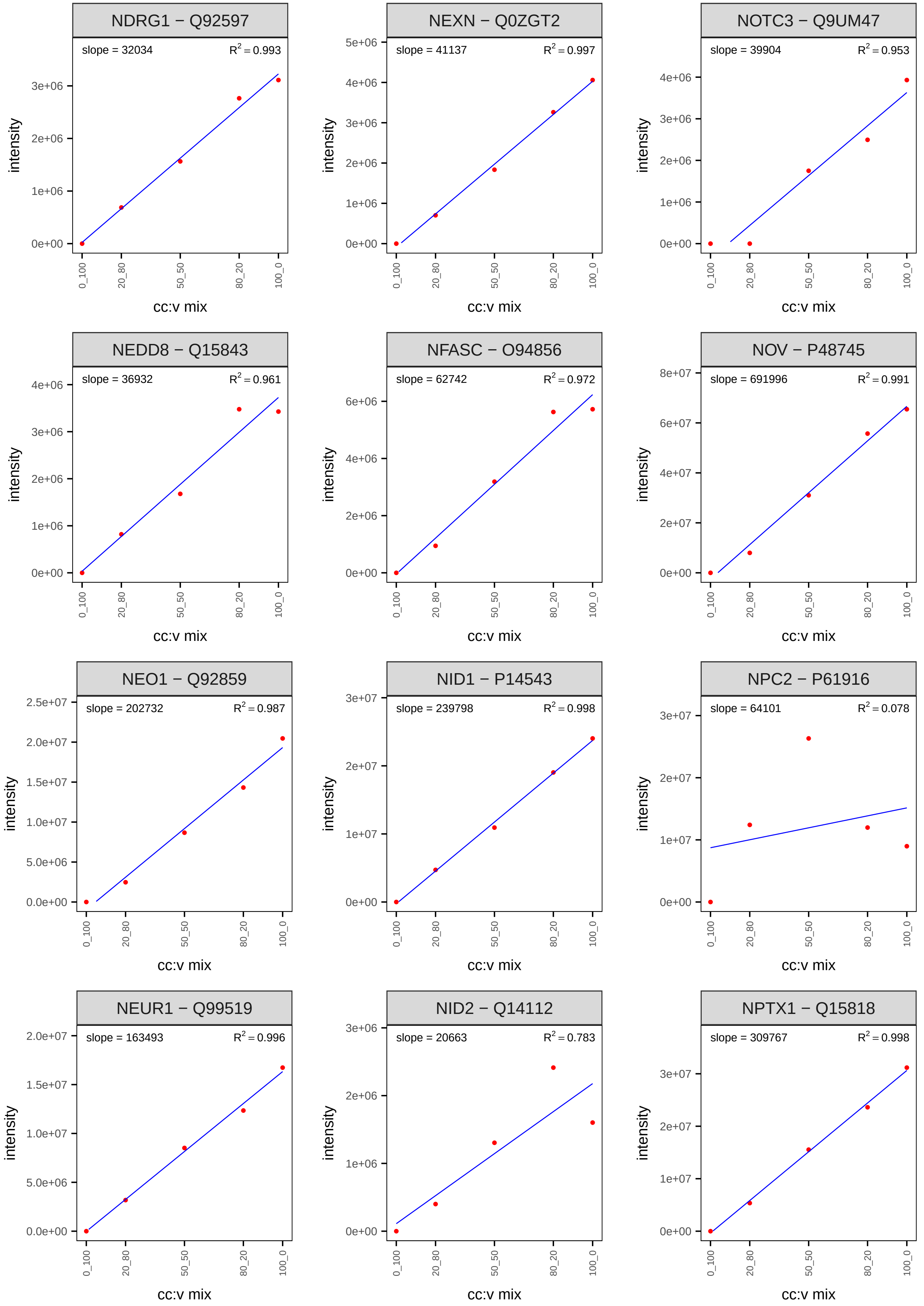
NDRG1 − Q92597
slope = 32034
R2 = 0.993
NEXN − Q0ZGT2
slope = 41137
R2 = 0.997
NOTC3 − Q9UM47
slope = 39904
R2 = 0.953
NEDD8 − Q15843
slope = 36932
R2 = 0.961
NFASC − O94856
slope = 62742
R2 = 0.972
NOV − P48745
slope = 691996
R2 = 0.991
NEO1 − Q92859
slope = 202732
R2 = 0.987
NID1 − P14543
slope = 239798
R2 = 0.998
NPC2 − P61916
slope = 64101
R2 = 0.078
NEUR1 − Q99519
slope = 163493
R2 = 0.996
NID2 − Q14112
slope = 20663
R2 = 0.783
NPTX1 − Q15818
slope = 309767
R2 = 0.998
intensity
cc:v mix
0_100
20_80
50_50
80_20
100_0

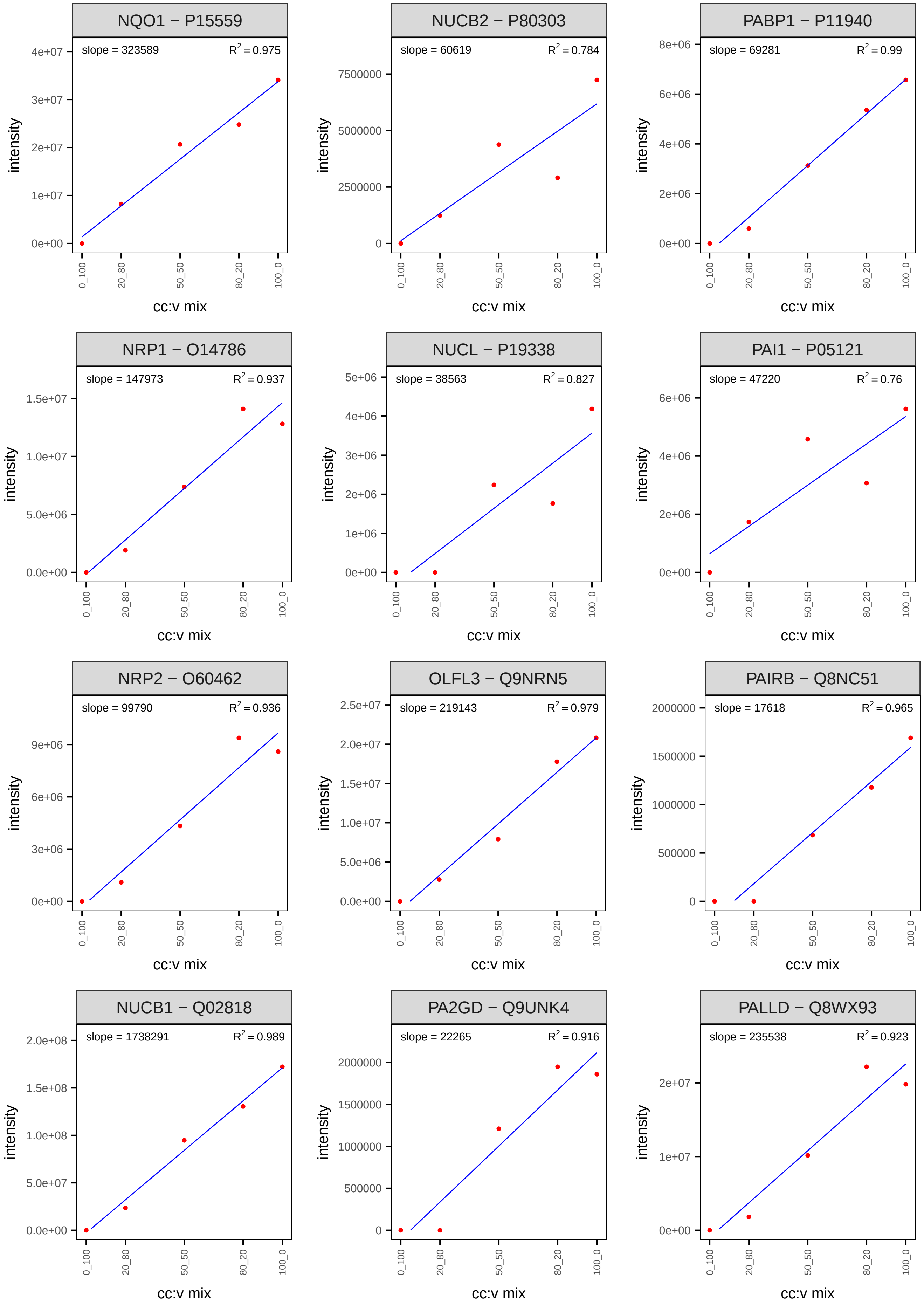
NQO1 − P15559
slope = 323589
R2 = 0.975
NUCB2 − P80303
slope = 60619
R2 = 0.784
PABP1 − P11940
slope = 69281
R2 = 0.99
NRP1 − O14786
slope = 147973
R2 = 0.937
NUCL − P19338
slope = 38563
R2 = 0.827
PAI1 − P05121
slope = 47220
R2 = 0.76
NRP2 − O60462
slope = 99790
R2 = 0.936
OLFL3 − Q9NRN5
slope = 219143
R2 = 0.979
PAIRB − Q8NC51
slope = 17618
R2 = 0.965
NUCB1 − Q02818
slope = 1738291
R2 = 0.989
PA2GD − Q9UNK4
slope = 22265
R2 = 0.916
PALLD − Q8WX93
slope = 235538
R2 = 0.923
intensity
cc:v mix
0_100
20_80
50_50
80_20
100_0

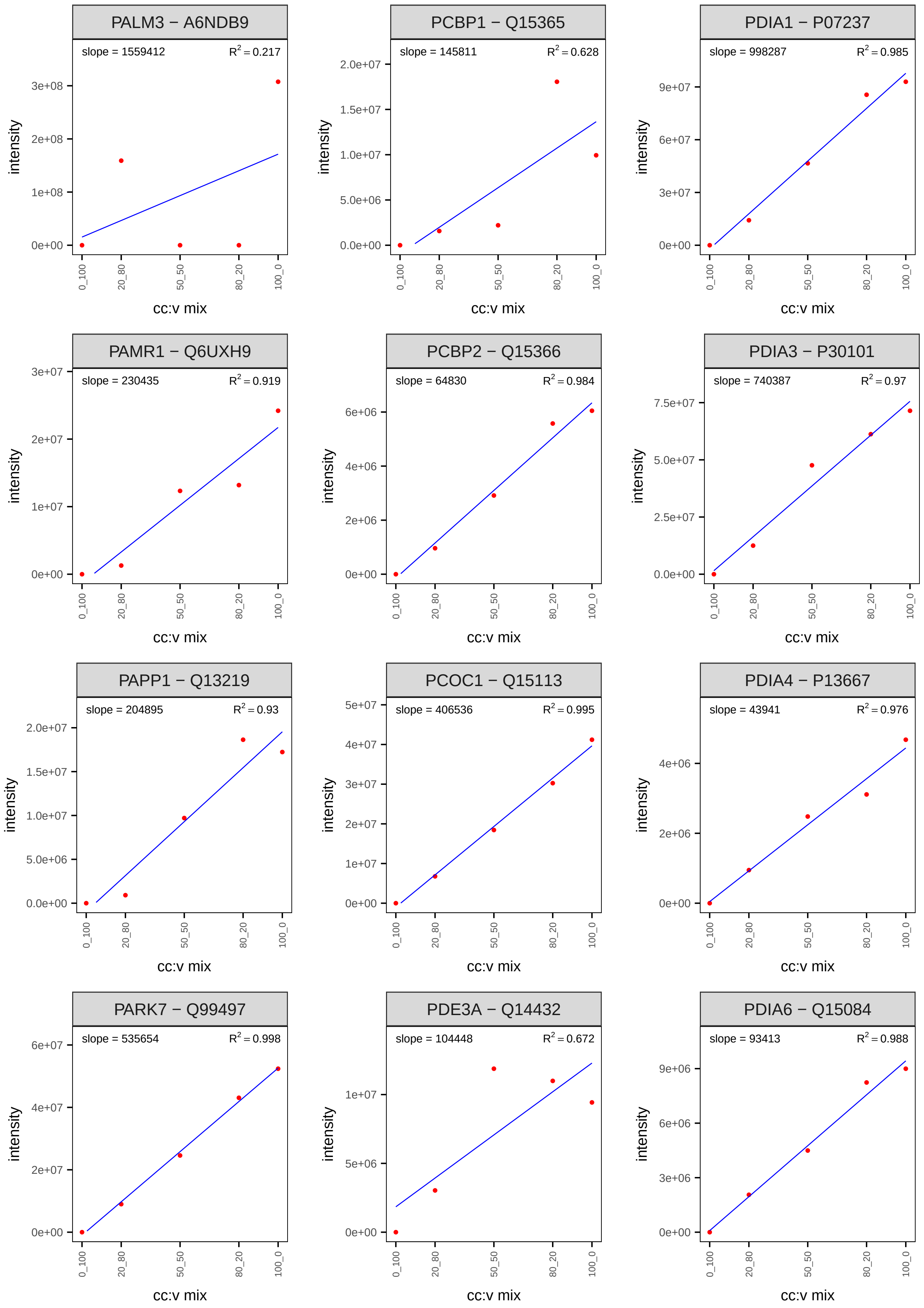
PALM3 − A6NDB9
slope = 1559412
R2 = 0.217
PCBP1 − Q15365
slope = 145811
R2 = 0.628
PDIA1 − P07237
slope = 998287
R2 = 0.985
PAMR1 − Q6UXH9
slope = 230435
R2 = 0.919
PCBP2 − Q15366
slope = 64830
R2 = 0.984
PDIA3 − P30101
slope = 740387
R2 = 0.97
PAPP1 − Q13219
slope = 204895
R2 = 0.93
PCOC1 − Q15113
slope = 406536
R2 = 0.995
PDIA4 − P13667
slope = 43941
R2 = 0.976
PARK7 − Q99497
slope = 535654
R2 = 0.998
PDE3A − Q14432
slope = 104448
R2 = 0.672
PDIA6 − Q15084
slope = 93413
R2 = 0.988
intensity
cc:v mix
0_100
20_80
50_50
80_20
100_0

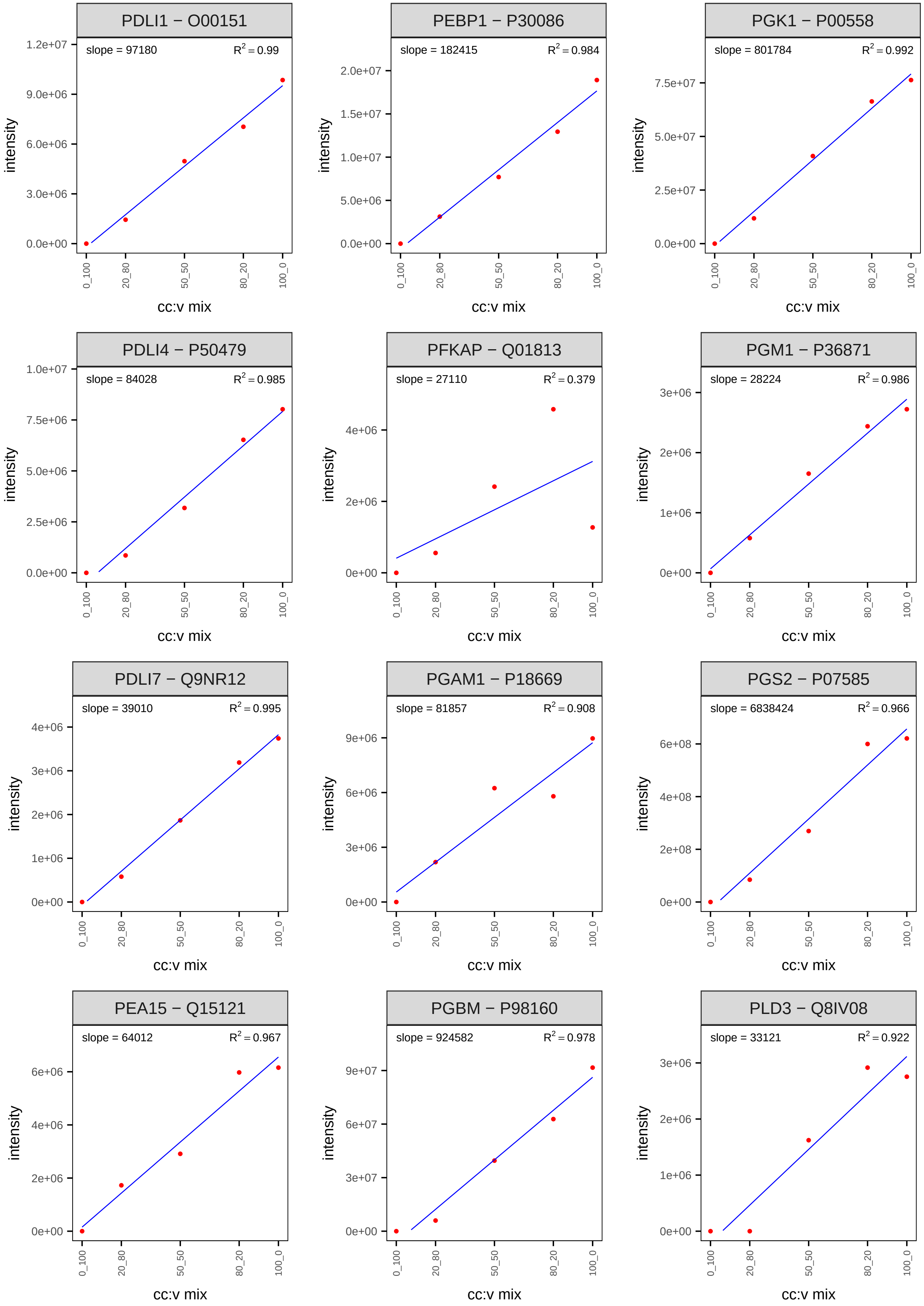
PDLI1 − O00151
slope = 97180
$R^2 = 0.99$
PEBP1 − P30086
slope = 182415
$R^2 = 0.984$
PGK1 − P00558
slope = 801784
$R^2 = 0.992$
PDLI4 − P50479
slope = 84028
$R^2 = 0.985$
PFKAP − Q01813
slope = 27110
$R^2 = 0.379$
PGM1 − P36871
slope = 28224
$R^2 = 0.986$
PDLI7 − Q9NR12
slope = 39010
$R^2 = 0.995$
PGAM1 − P18669
slope = 81857
$R^2 = 0.908$
PGS2 − P07585
slope = 6838424
$R^2 = 0.966$
PEA15 − Q15121
slope = 64012
$R^2 = 0.967$
PGBM − P98160
slope = 924582
$R^2 = 0.978$
PLD3 − Q8IV08
slope = 33121
$R^2 = 0.922$
intensity
cc:v mix
0_100
20_80
50_50
80_20
100_0

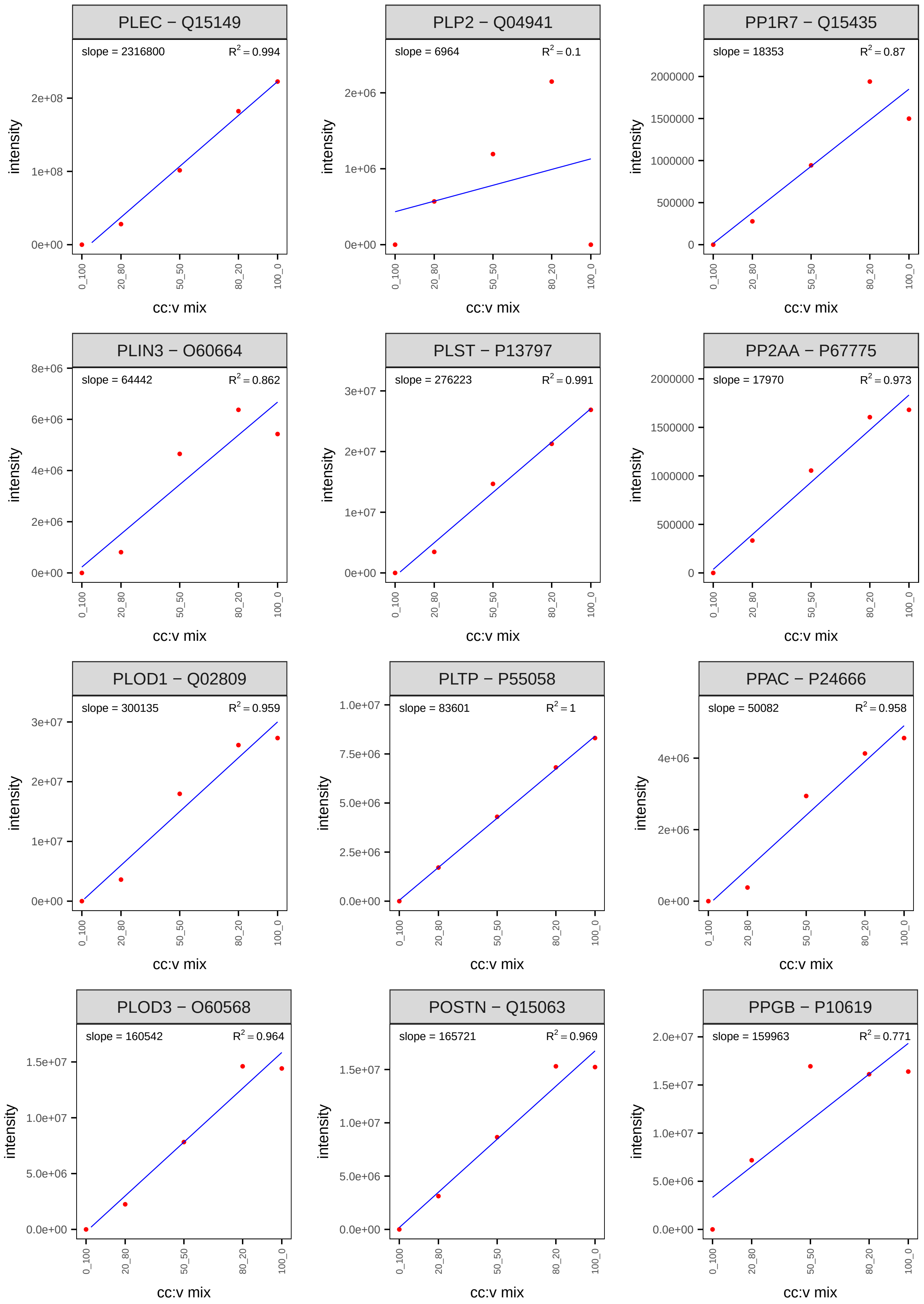
PLEC − Q15149
slope = 2316800
$R^2 = 0.994$
PLP2 − Q04941
slope = 6964
$R^2 = 0.1$
PP1R7 − Q15435
slope = 18353
$R^2 = 0.87$
PLIN3 − O60664
slope = 64442
$R^2 = 0.862$
PLST − P13797
slope = 276223
$R^2 = 0.991$
PP2AA − P67775
slope = 17970
$R^2 = 0.973$
PLOD1 − Q02809
slope = 300135
$R^2 = 0.959$
PLTP − P55058
slope = 83601
$R^2 = 1$
PPAC − P24666
slope = 50082
$R^2 = 0.958$
PLOD3 − O60568
slope = 160542
$R^2 = 0.964$
POSTN − Q15063
slope = 165721
$R^2 = 0.969$
PPGB − P10619
slope = 159963
$R^2 = 0.771$
intensity
cc:v mix
0_100
20_80
50_50
80_20
100_0

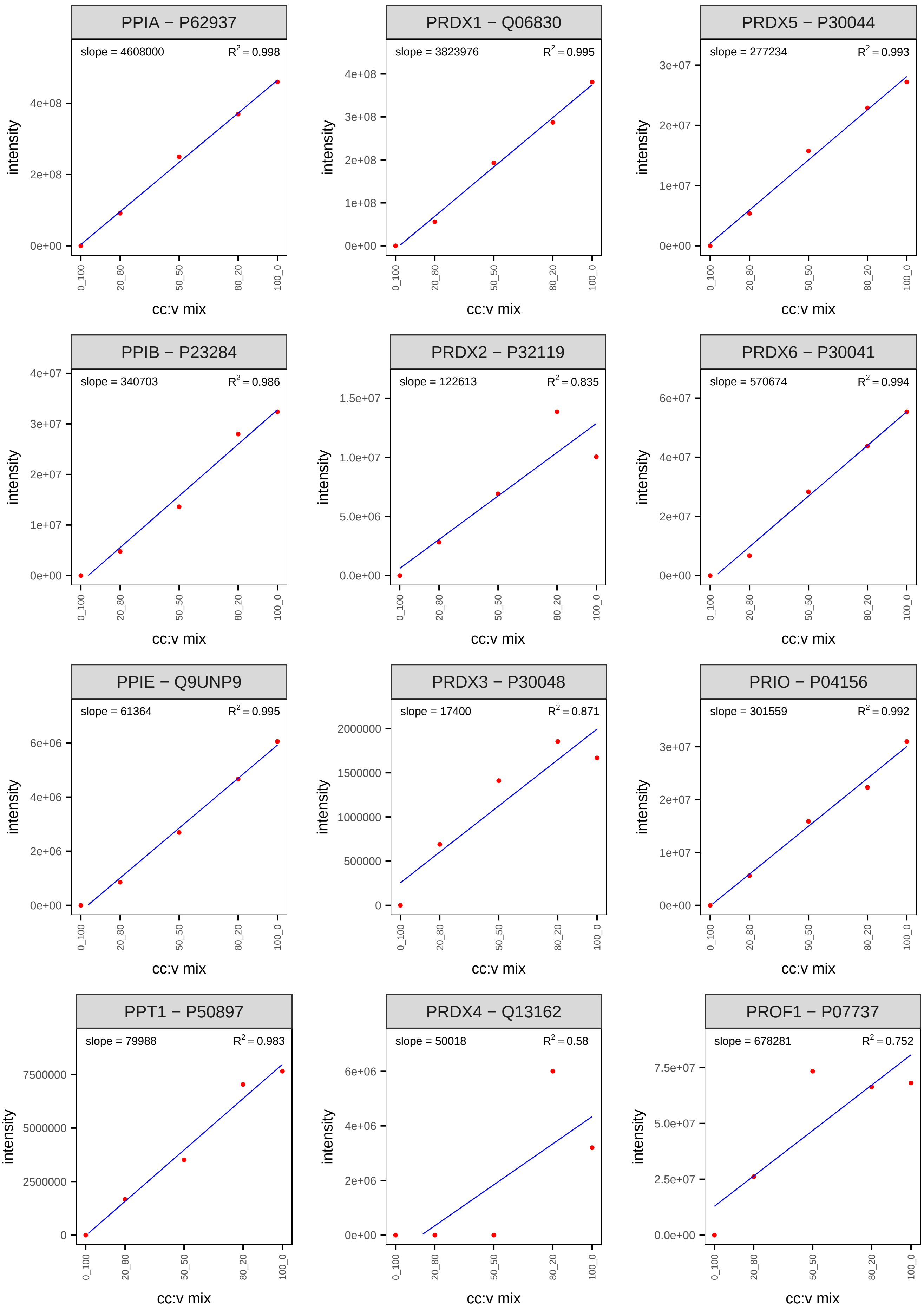
PPIA − P62937
slope = 4608000
R2 = 0.998
PRDX1 − Q06830
slope = 3823976
R2 = 0.995
PRDX5 − P30044
slope = 277234
R2 = 0.993
PPIB − P23284
slope = 340703
R2 = 0.986
PRDX2 − P32119
slope = 122613
R2 = 0.835
PRDX6 − P30041
slope = 570674
R2 = 0.994
PPIE − Q9UNP9
slope = 61364
R2 = 0.995
PRDX3 − P30048
slope = 17400
R2 = 0.871
PRIO − P04156
slope = 301559
R2 = 0.992
PPT1 − P50897
slope = 79988
R2 = 0.983
PRDX4 − Q13162
slope = 50018
R2 = 0.58
PROF1 − P07737
slope = 678281
R2 = 0.752
intensity
cc:v mix
0_100
20_80
50_50
80_20
100_0

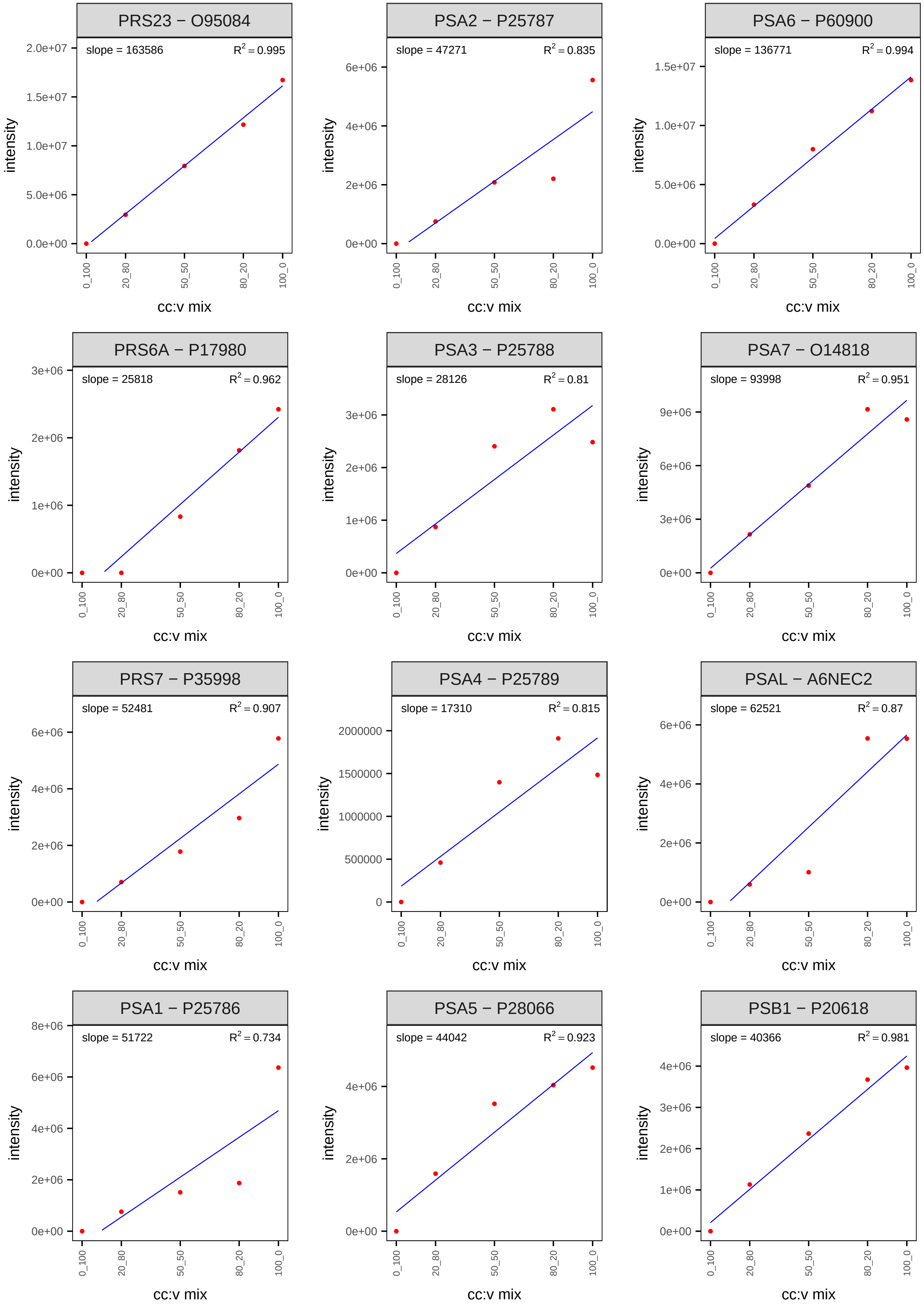
PRS23 − O95084
slope = 163586
R2 = 0.995
PSA2 − P25787
slope = 47271
R2 = 0.835
PSA6 − P60900
slope = 136771
R2 = 0.994
PRS6A − P17980
slope = 25818
R2 = 0.962
PSA3 − P25788
slope = 28126
R2 = 0.81
PSA7 − O14818
slope = 93998
R2 = 0.951
PRS7 − P35998
slope = 52481
R2 = 0.907
PSA4 − P25789
slope = 17310
R2 = 0.815
PSAL − A6NEC2
slope = 62521
R2 = 0.87
PSA1 − P25786
slope = 51722
R2 = 0.734
PSA5 − P28066
slope = 44042
R2 = 0.923
PSB1 − P20618
slope = 40366
R2 = 0.981
intensity
cc:v mix
0_100
20_80
50_50
80_20
100_0

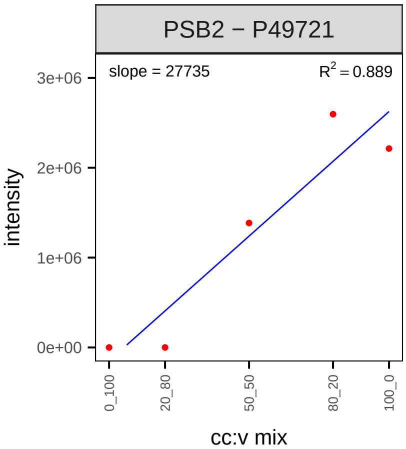
PSB2 − P49721
slope = 27735
$R^2 = 0.889$
intensity
3e+06
2e+06
1e+06
0e+00
0_100
20_80
50_50
80_20
100_0
cc:v mix

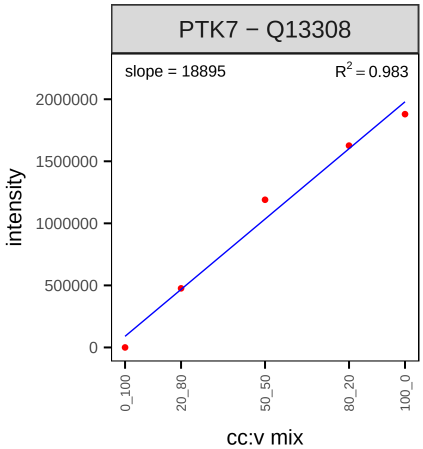
PTK7 − Q13308
slope = 18895
$R^2 = 0.983$
intensity
2000000
1500000
1000000
500000
0
0_100
20_80
50_50
80_20
100_0
cc:v mix

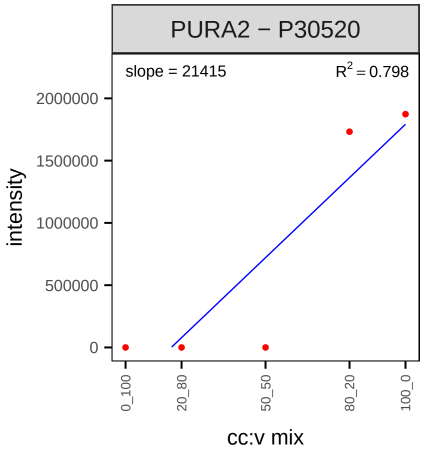
PURA2 − P30520
slope = 21415
$R^2 = 0.798$
intensity
2000000
1500000
1000000
500000
0
0_100
20_80
50_50
80_20
100_0
cc:v mix

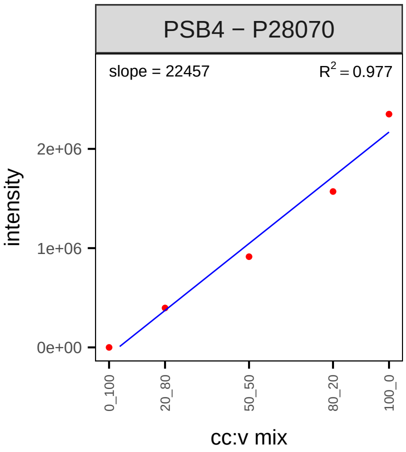
PSB4 − P28070
slope = 22457
$R^2 = 0.977$
intensity
2e+06
1e+06
0e+00
0_100
20_80
50_50
80_20
100_0
cc:v mix

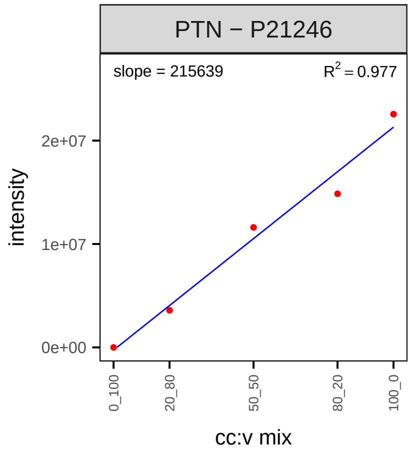
PTN − P21246
slope = 215639
$R^2 = 0.977$
intensity
2e+07
1e+07
0e+00
0_100
20_80
50_50
80_20
100_0
cc:v mix

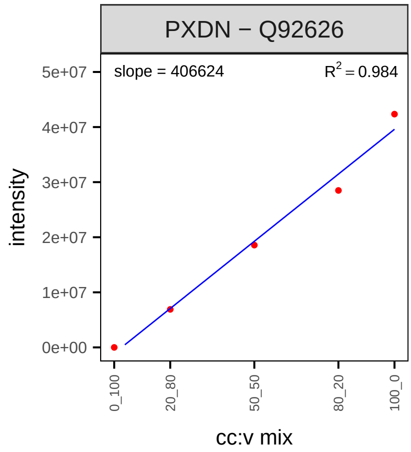
PXDN − Q92626
slope = 406624
$R^2 = 0.984$
intensity
5e+07
4e+07
3e+07
2e+07
1e+07
0e+00
0_100
20_80
50_50
80_20
100_0
cc:v mix

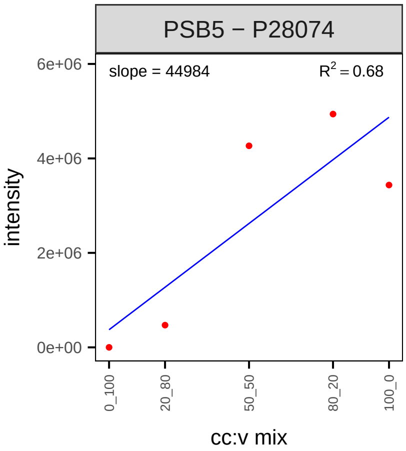
PSB5 − P28074
slope = 44984
$R^2 = 0.68$
intensity
6e+06
4e+06
2e+06
0e+00
0_100
20_80
50_50
80_20
100_0
cc:v mix

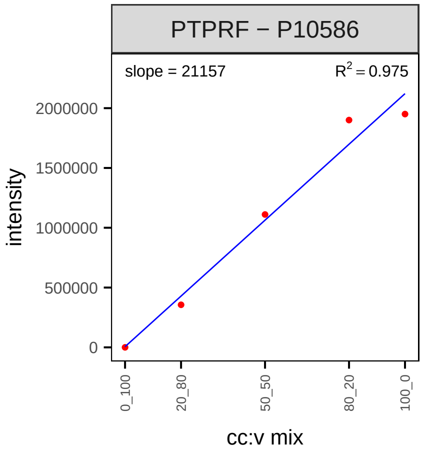
PTPRF − P10586
slope = 21157
$R^2 = 0.975$
intensity
2000000
1500000
1000000
500000
0
0_100
20_80
50_50
80_20
100_0
cc:v mix

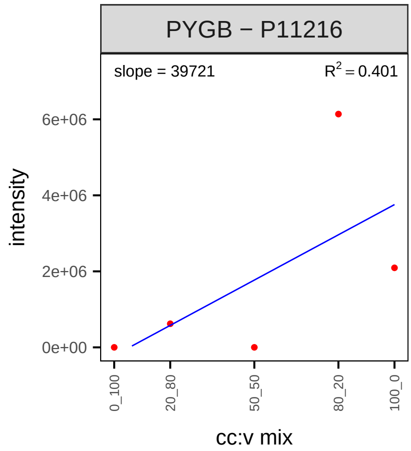
PYGB − P11216
slope = 39721
$R^2 = 0.401$
intensity
6e+06
4e+06
2e+06
0e+00
0_100
20_80
50_50
80_20
100_0
cc:v mix

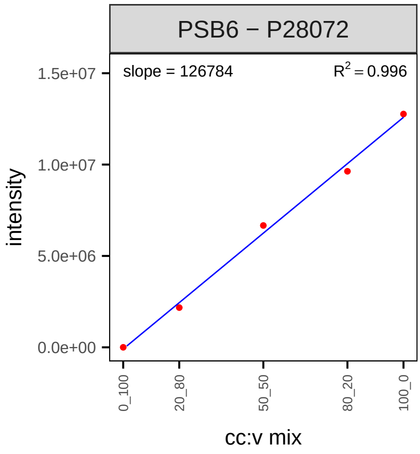
PSB6 − P28072
slope = 126784
$R^2 = 0.996$
intensity
1.5e+07
1.0e+07
5.0e+06
0.0e+00
0_100
20_80
50_50
80_20
100_0
cc:v mix

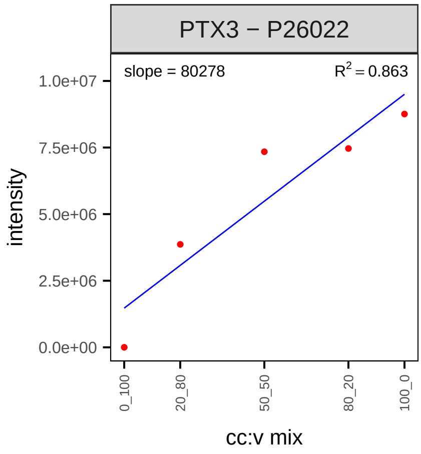
PTX3 − P26022
slope = 80278
$R^2 = 0.863$
intensity
1.0e+07
7.5e+06
5.0e+06
2.5e+06
0.0e+00
0_100
20_80
50_50
80_20
100_0
cc:v mix

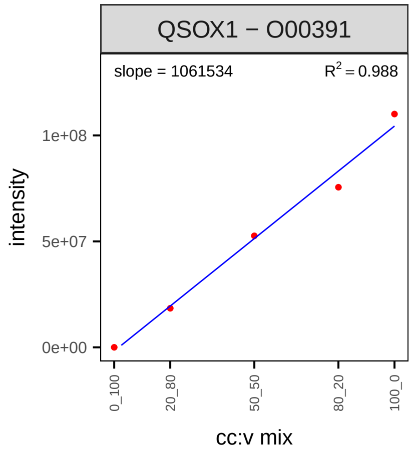
QSOX1 − O00391
slope = 1061534
$R^2 = 0.988$
intensity
1e+08
5e+07
0e+00
0_100
20_80
50_50
80_20
100_0
cc:v mix

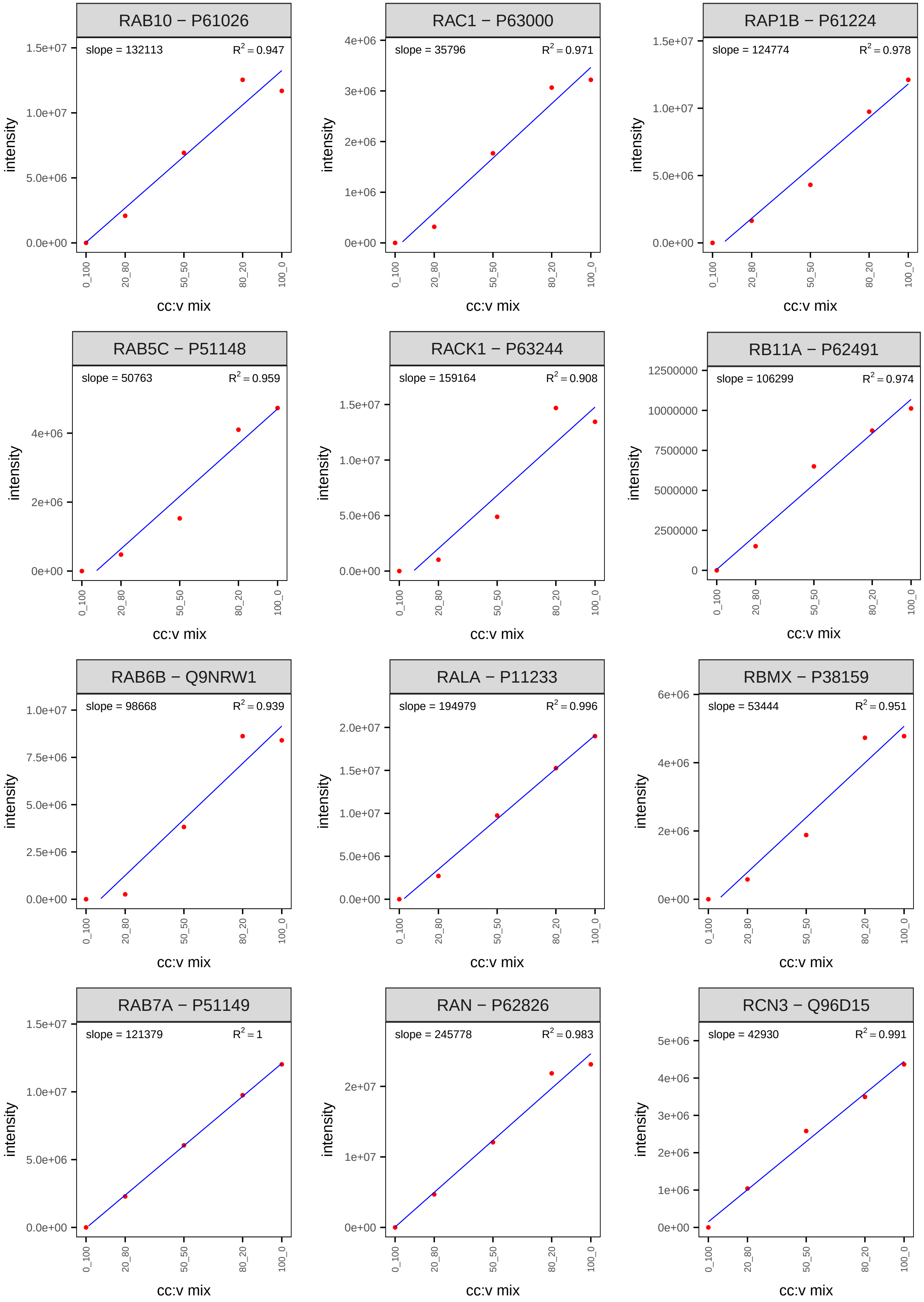
RAB10 − P61026
slope = 132113
R2 = 0.947
RAC1 − P63000
slope = 35796
R2 = 0.971
RAP1B − P61224
slope = 124774
R2 = 0.978
RAB5C − P51148
slope = 50763
R2 = 0.959
RACK1 − P63244
slope = 159164
R2 = 0.908
RB11A − P62491
slope = 106299
R2 = 0.974
RAB6B − Q9NRW1
slope = 98668
R2 = 0.939
RALA − P11233
slope = 194979
R2 = 0.996
RBMX − P38159
slope = 53444
R2 = 0.951
RAB7A − P51149
slope = 121379
R2 = 1
RAN − P62826
slope = 245778
R2 = 0.983
RCN3 − Q96D15
slope = 42930
R2 = 0.991
intensity
cc:v mix
0_100
20_80
50_50
80_20
100_0

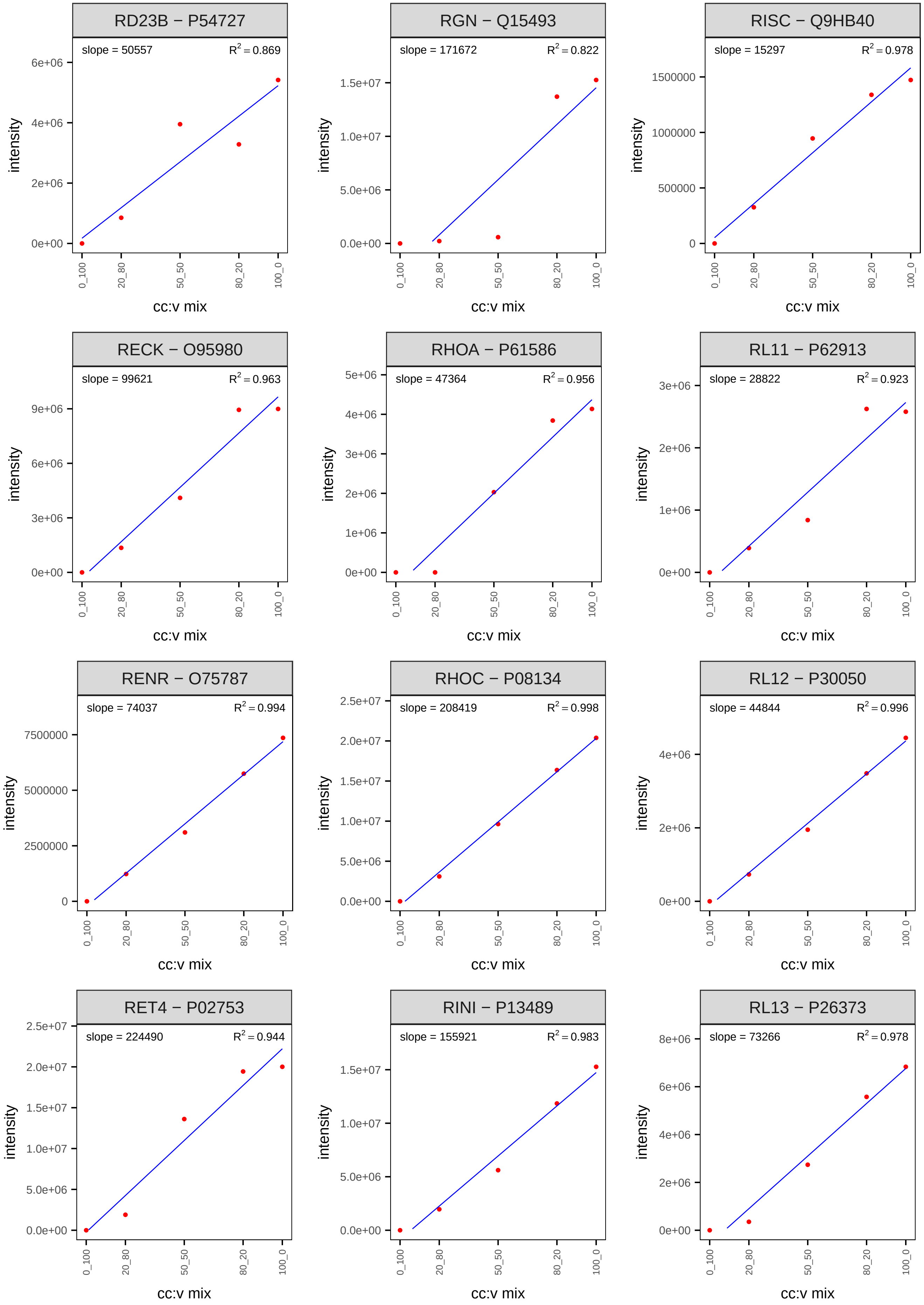
RD23B − P54727
slope = 50557
$R^2 = 0.869$
RGN − Q15493
slope = 171672
$R^2 = 0.822$
RISC − Q9HB40
slope = 15297
$R^2 = 0.978$
RECK − O95980
slope = 99621
$R^2 = 0.963$
RHOA − P61586
slope = 47364
$R^2 = 0.956$
RL11 − P62913
slope = 28822
$R^2 = 0.923$
RENR − O75787
slope = 74037
$R^2 = 0.994$
RHOC − P08134
slope = 208419
$R^2 = 0.998$
RL12 − P30050
slope = 44844
$R^2 = 0.996$
RET4 − P02753
slope = 224490
$R^2 = 0.944$
RINI − P13489
slope = 155921
$R^2 = 0.983$
RL13 − P26373
slope = 73266
$R^2 = 0.978$
intensity
cc:v mix
0_100
20_80
50_50
80_20
100_0

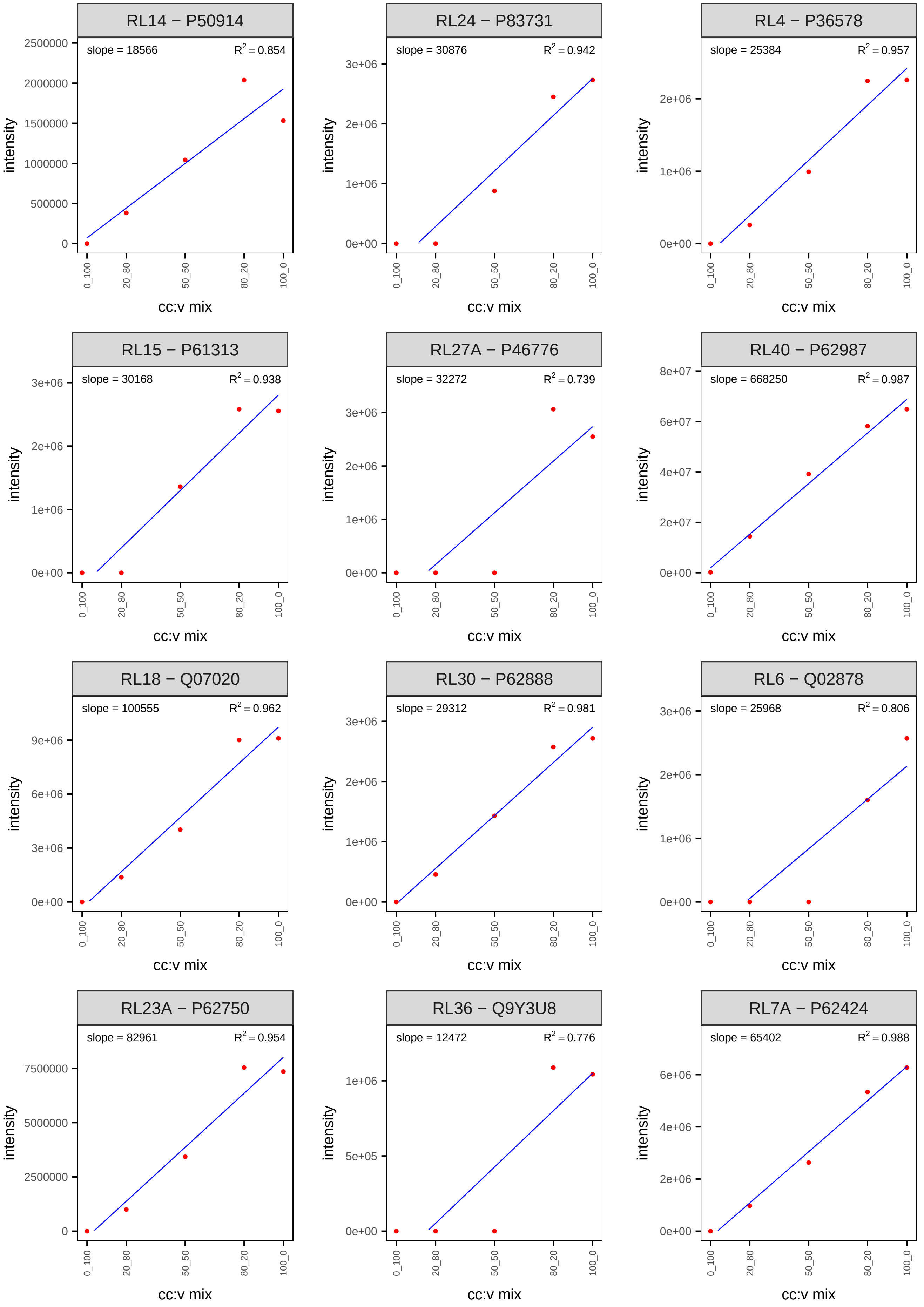
RL14 − P50914
slope = 18566
R^2 = 0.854
RL24 − P83731
slope = 30876
R^2 = 0.942
RL4 − P36578
slope = 25384
R^2 = 0.957
RL15 − P61313
slope = 30168
R^2 = 0.938
RL27A − P46776
slope = 32272
R^2 = 0.739
RL40 − P62987
slope = 668250
R^2 = 0.987
RL18 − Q07020
slope = 100555
R^2 = 0.962
RL30 − P62888
slope = 29312
R^2 = 0.981
RL6 − Q02878
slope = 25968
R^2 = 0.806
RL23A − P62750
slope = 82961
R^2 = 0.954
RL36 − Q9Y3U8
slope = 12472
R^2 = 0.776
RL7A − P62424
slope = 65402
R^2 = 0.988
intensity
cc:v mix
0_100
20_80
50_50
80_20
100_0

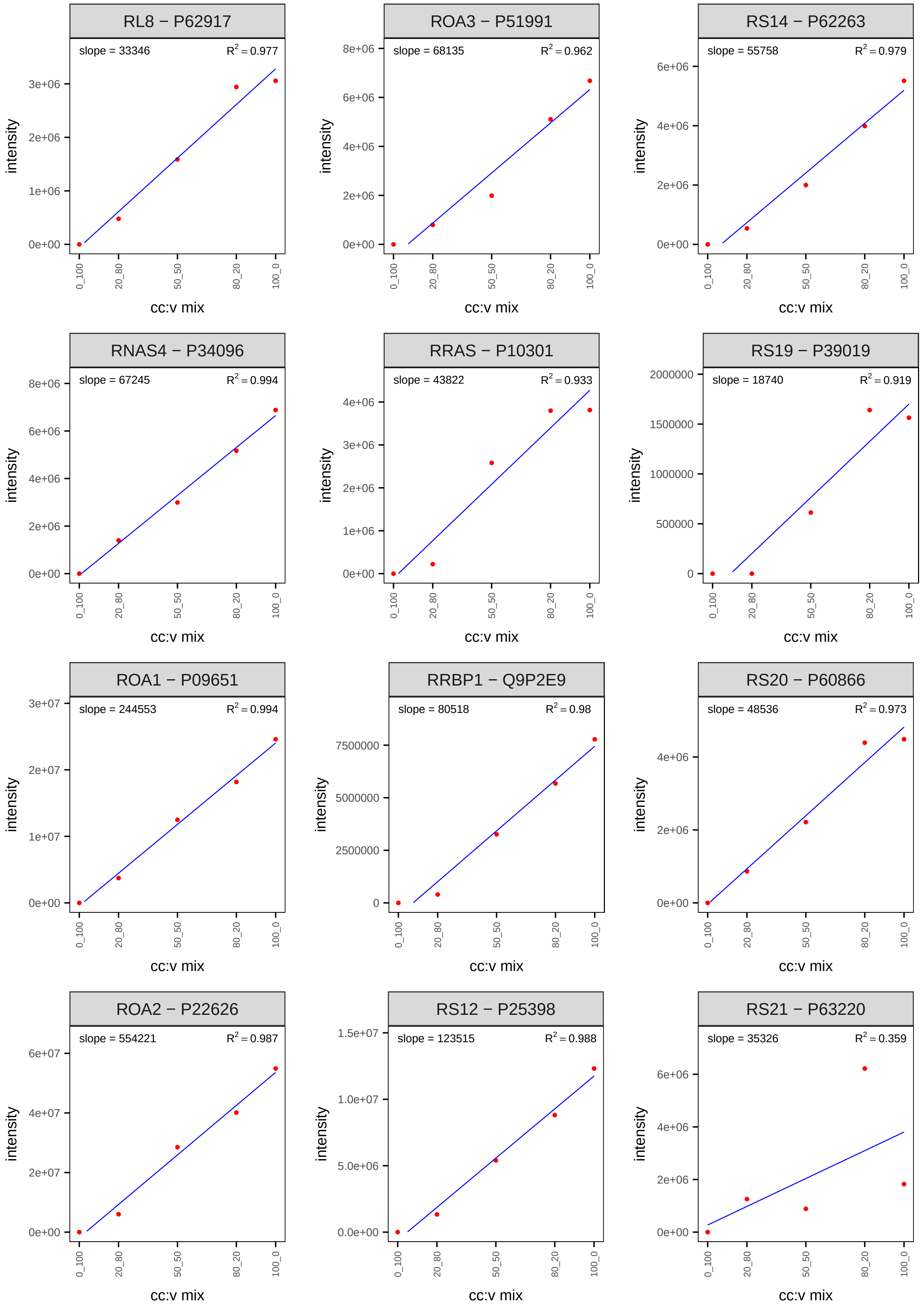
RL8 − P62917
slope = 33346
R2 = 0.977
ROA3 − P51991
slope = 68135
R2 = 0.962
RS14 − P62263
slope = 55758
R2 = 0.979
RNAS4 − P34096
slope = 67245
R2 = 0.994
RRAS − P10301
slope = 43822
R2 = 0.933
RS19 − P39019
slope = 18740
R2 = 0.919
ROA1 − P09651
slope = 244553
R2 = 0.994
RRBP1 − Q9P2E9
slope = 80518
R2 = 0.98
RS20 − P60866
slope = 48536
R2 = 0.973
ROA2 − P22626
slope = 554221
R2 = 0.987
RS12 − P25398
slope = 123515
R2 = 0.988
RS21 − P63220
slope = 35326
R2 = 0.359
intensity
cc:v mix
0_100
20_80
50_50
80_20
100_0

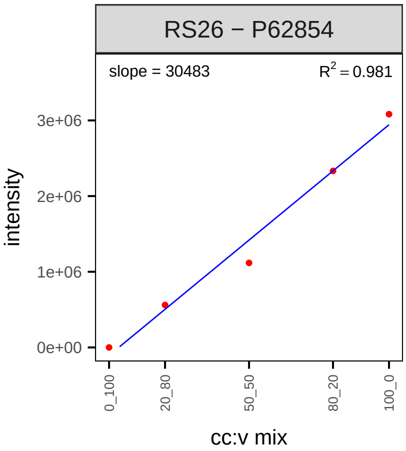
RS26 − P62854
slope = 30483
$R^2 = 0.981$
intensity
0e+00
1e+06
2e+06
3e+06
0_100
20_80
50_50
80_20
100_0
cc:v mix

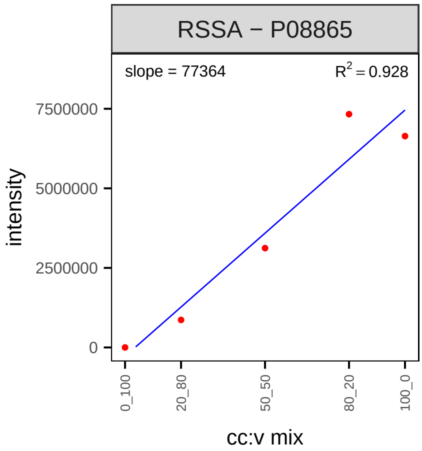
RSSA − P08865
slope = 77364
$R^2 = 0.928$
intensity
0
2500000
5000000
7500000
0_100
20_80
50_50
80_20
100_0
cc:v mix

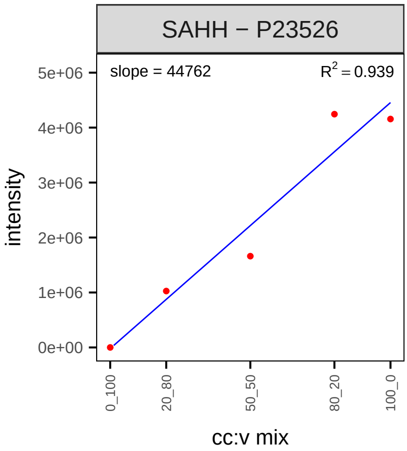
SAHH − P23526
slope = 44762
$R^2 = 0.939$
intensity
0e+00
1e+06
2e+06
3e+06
4e+06
5e+06
0_100
20_80
50_50
80_20
100_0
cc:v mix

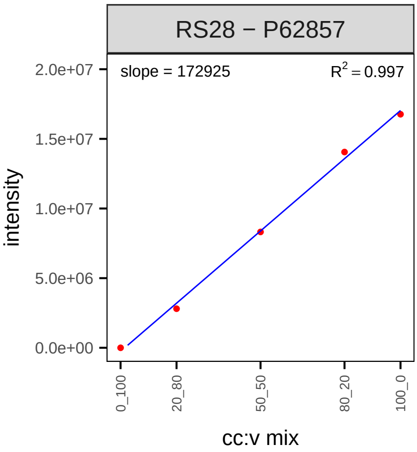
RS28 − P62857
slope = 172925
$R^2 = 0.997$
intensity
0.0e+00
5.0e+06
1.0e+07
1.5e+07
2.0e+07
0_100
20_80
50_50
80_20
100_0
cc:v mix

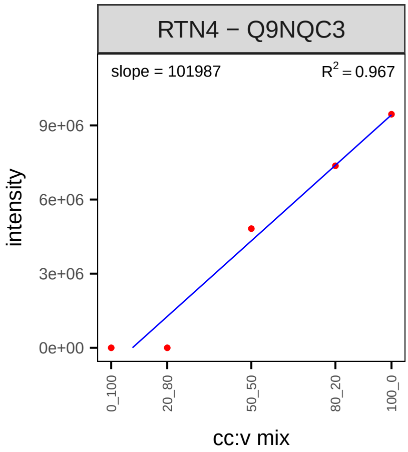
RTN4 − Q9NQC3
slope = 101987
$R^2 = 0.967$
intensity
0e+00
3e+06
6e+06
9e+06
0_100
20_80
50_50
80_20
100_0
cc:v mix

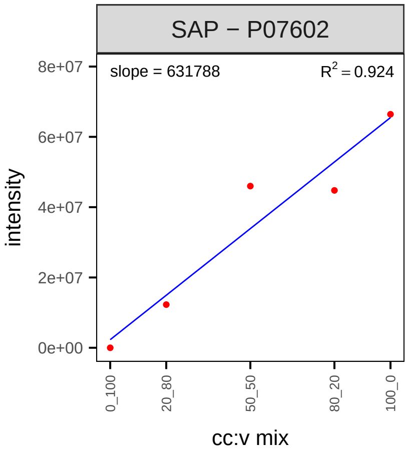
SAP − P07602
slope = 631788
$R^2 = 0.924$
intensity
0e+00
2e+07
4e+07
6e+07
8e+07
0_100
20_80
50_50
80_20
100_0
cc:v mix

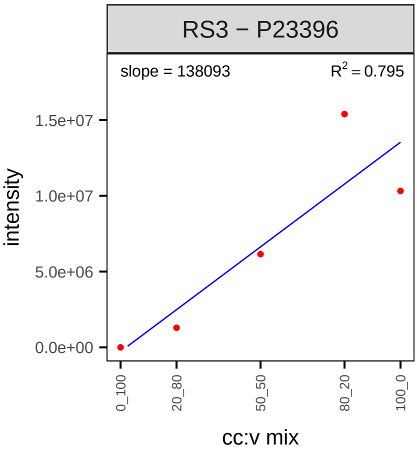
RS3 − P23396
slope = 138093
$R^2 = 0.795$
intensity
0.0e+00
5.0e+06
1.0e+07
1.5e+07
0_100
20_80
50_50
80_20
100_0
cc:v mix

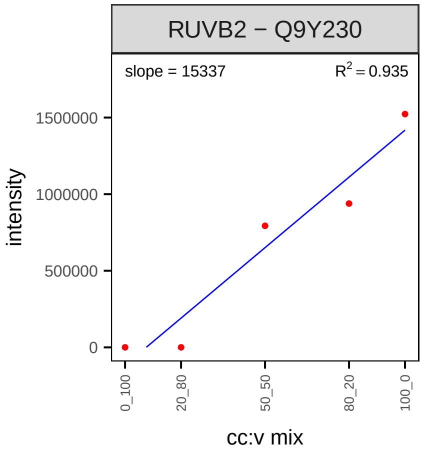
RUVB2 − Q9Y230
slope = 15337
$R^2 = 0.935$
intensity
0
500000
1000000
1500000
0_100
20_80
50_50
80_20
100_0
cc:v mix

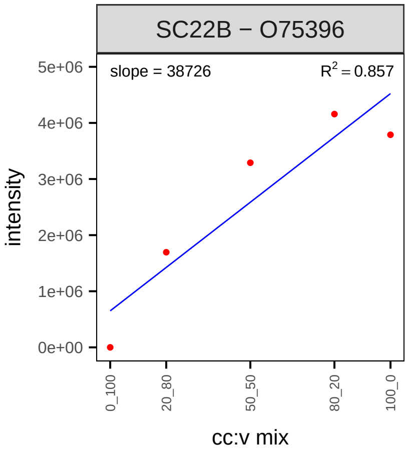
SC22B − O75396
slope = 38726
$R^2 = 0.857$
intensity
0e+00
1e+06
2e+06
3e+06
4e+06
5e+06
0_100
20_80
50_50
80_20
100_0
cc:v mix

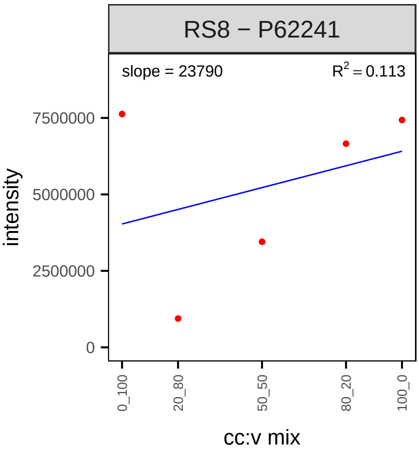
RS8 − P62241
slope = 23790
$R^2 = 0.113$
intensity
0
2500000
5000000
7500000
0_100
20_80
50_50
80_20
100_0
cc:v mix

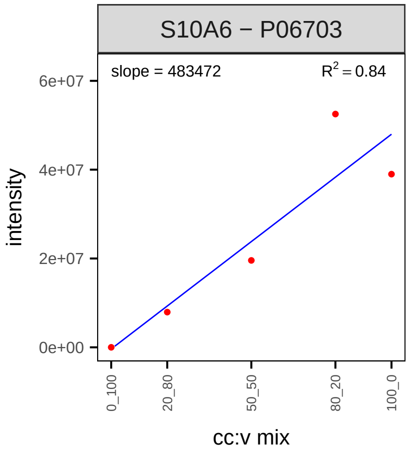
S10A6 − P06703
slope = 483472
$R^2 = 0.84$
intensity
0e+00
2e+07
4e+07
6e+07
0_100
20_80
50_50
80_20
100_0
cc:v mix

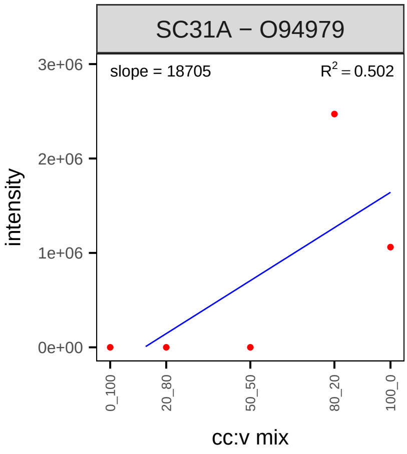
SC31A − O94979
slope = 18705
$R^2 = 0.502$
intensity
0e+00
1e+06
2e+06
3e+06
0_100
20_80
50_50
80_20
100_0
cc:v mix

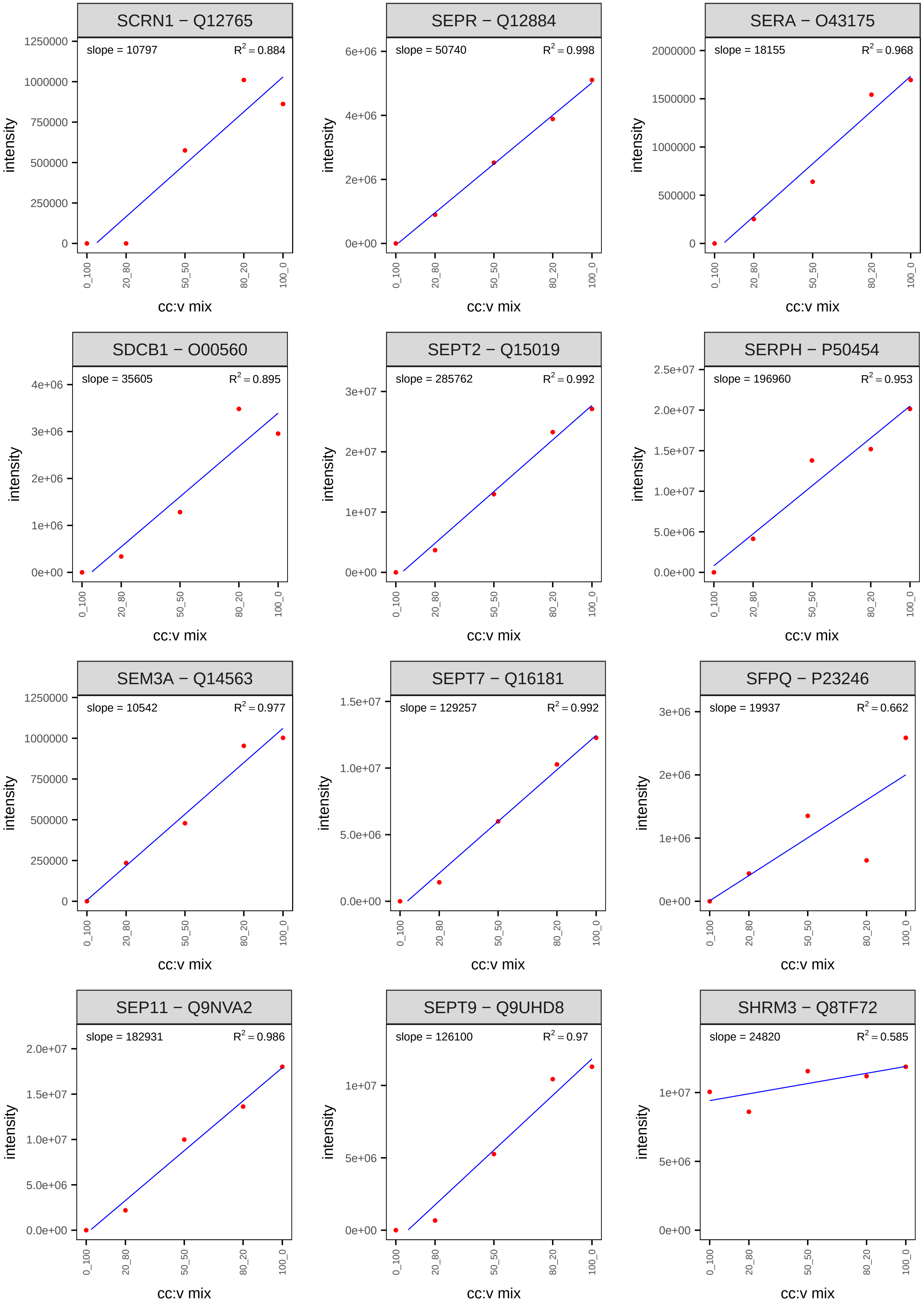

SCRN1 − Q12765
slope = 10797
R2 = 0.884
SEPR − Q12884
slope = 50740
R2 = 0.998
SERA − O43175
slope = 18155
R2 = 0.968
SDCB1 − O00560
slope = 35605
R2 = 0.895
SEPT2 − Q15019
slope = 285762
R2 = 0.992
SERPH − P50454
slope = 196960
R2 = 0.953
SEM3A − Q14563
slope = 10542
R2 = 0.977
SEPT7 − Q16181
slope = 129257
R2 = 0.992
SFPQ − P23246
slope = 19937
R2 = 0.662
SEP11 − Q9NVA2
slope = 182931
R2 = 0.986
SEPT9 − Q9UHD8
slope = 126100
R2 = 0.97
SHRM3 − Q8TF72
slope = 24820
R2 = 0.585
intensity
cc:v mix
0_100
20_80
50_50
80_20
100_0

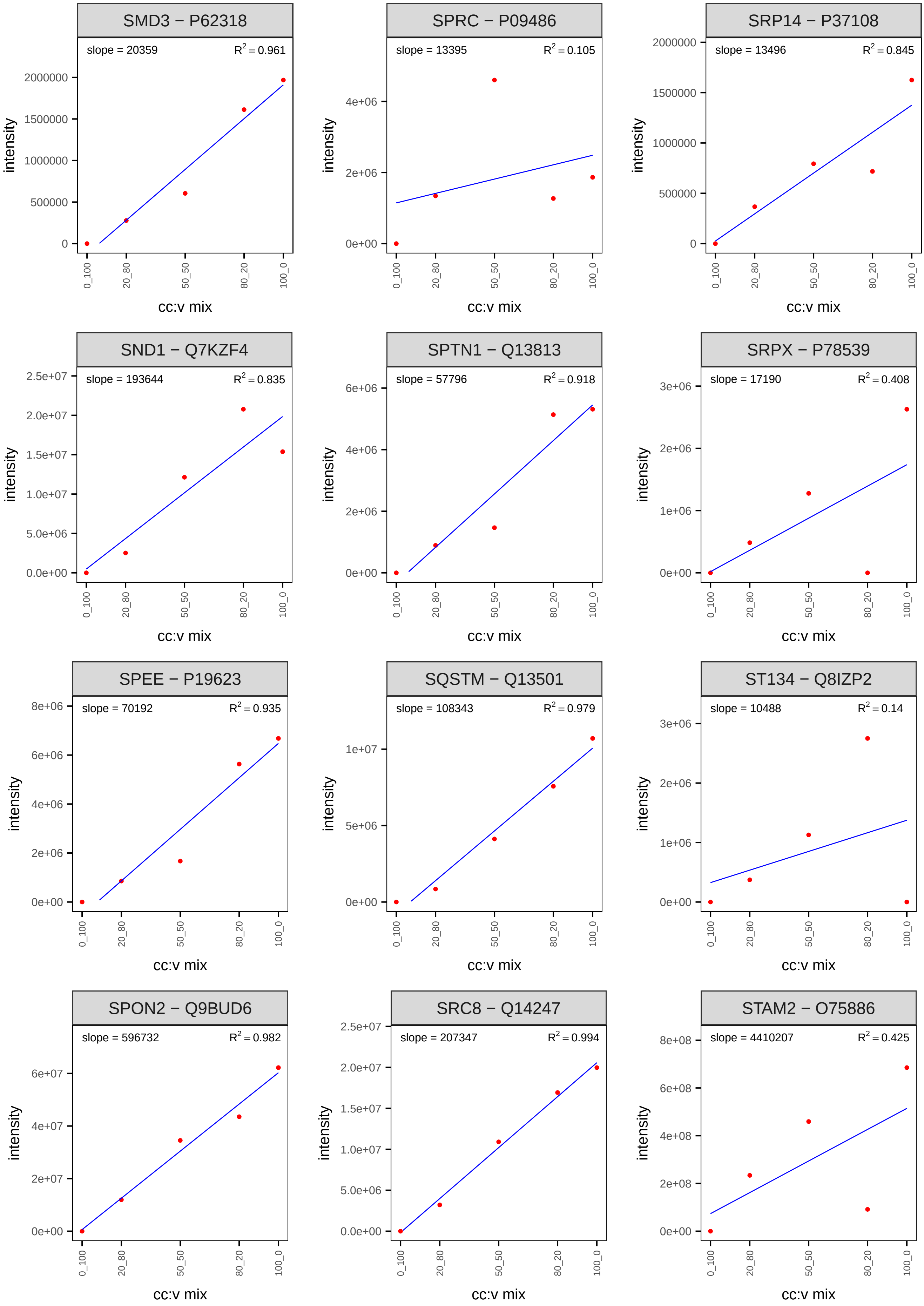

SMD3 − P62318
slope = 20359
R2 = 0.961
SPRC − P09486
slope = 13395
R2 = 0.105
SRP14 − P37108
slope = 13496
R2 = 0.845
SND1 − Q7KZF4
slope = 193644
R2 = 0.835
SPTN1 − Q13813
slope = 57796
R2 = 0.918
SRPX − P78539
slope = 17190
R2 = 0.408
SPEE − P19623
slope = 70192
R2 = 0.935
SQSTM − Q13501
slope = 108343
R2 = 0.979
ST134 − Q8IZP2
slope = 10488
R2 = 0.14
SPON2 − Q9BUD6
slope = 596732
R2 = 0.982
SRC8 − Q14247
slope = 207347
R2 = 0.994
STAM2 − O75886
slope = 4410207
R2 = 0.425
intensity
cc:v mix
0_100
20_80
50_50
80_20
100_0

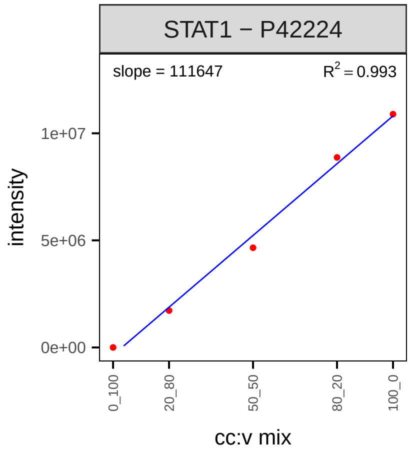
STAT1 − P42224
slope = 111647
$R^2 = 0.993$
intensity
cc:v mix

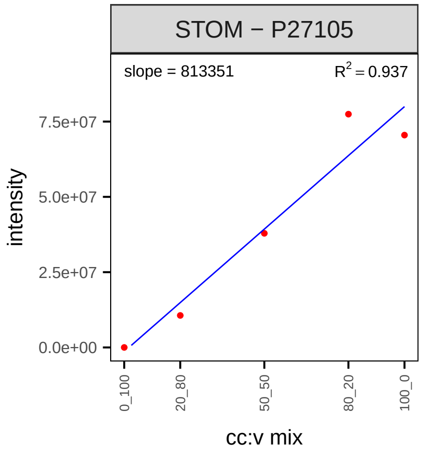
STOM − P27105
slope = 813351
$R^2 = 0.937$
intensity
cc:v mix

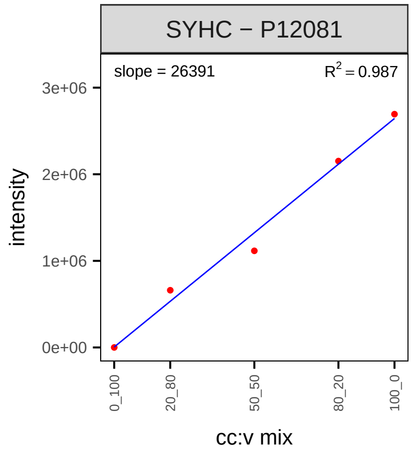
SYHC − P12081
slope = 26391
$R^2 = 0.987$
intensity
cc:v mix

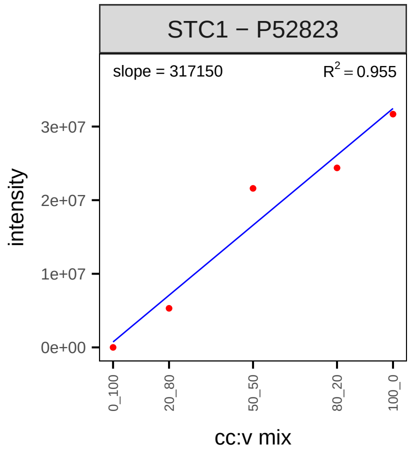
STC1 − P52823
slope = 317150
$R^2 = 0.955$
intensity
cc:v mix

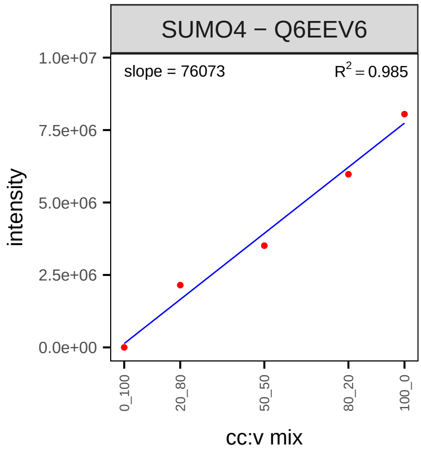
SUMO4 − Q6EEV6
slope = 76073
$R^2 = 0.985$
intensity
cc:v mix

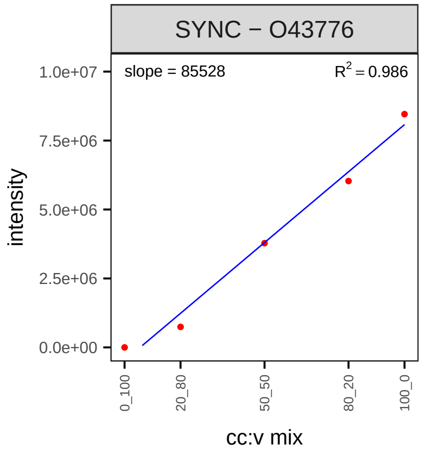
SYNC − O43776
slope = 85528
$R^2 = 0.986$
intensity
cc:v mix

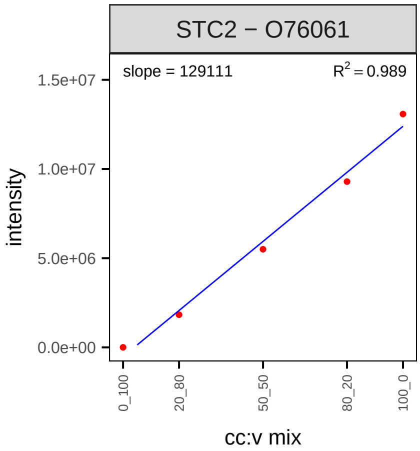
STC2 − O76061
slope = 129111
$R^2 = 0.989$
intensity
cc:v mix

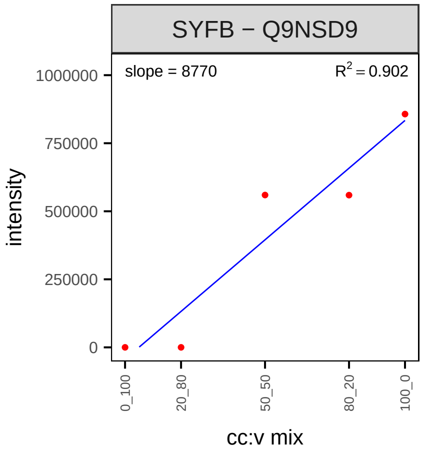
SYFB − Q9NSD9
slope = 8770
$R^2 = 0.902$
intensity
cc:v mix

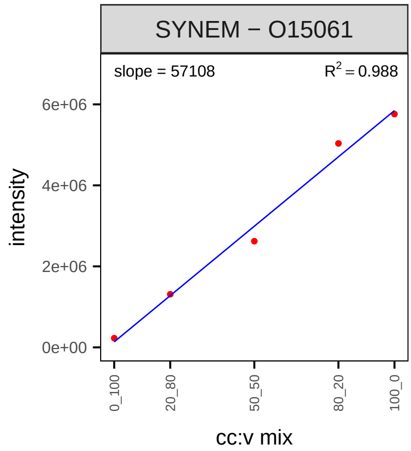
SYNEM − O15061
slope = 57108
$R^2 = 0.988$
intensity
cc:v mix

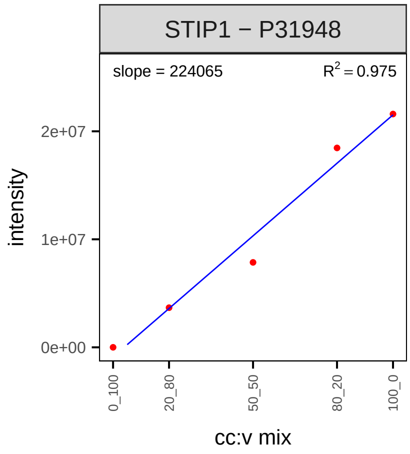
STIP1 − P31948
slope = 224065
$R^2 = 0.975$
intensity
cc:v mix

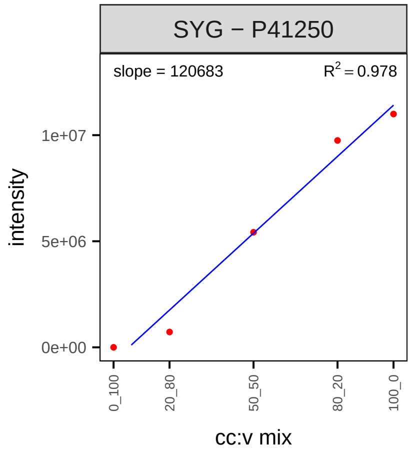
SYG − P41250
slope = 120683
$R^2 = 0.978$
intensity
cc:v mix

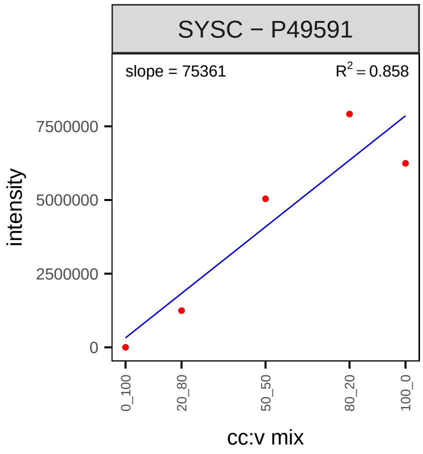
SYSC − P49591
slope = 75361
$R^2 = 0.858$
intensity
cc:v mix

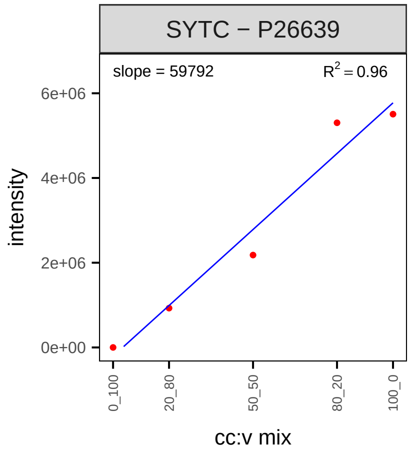
SYTC − P26639
slope = 59792
$R^2 = 0.96$
intensity
6e+06
4e+06
2e+06
0e+00
0_100
20_80
50_50
80_20
100_0
cc:v mix

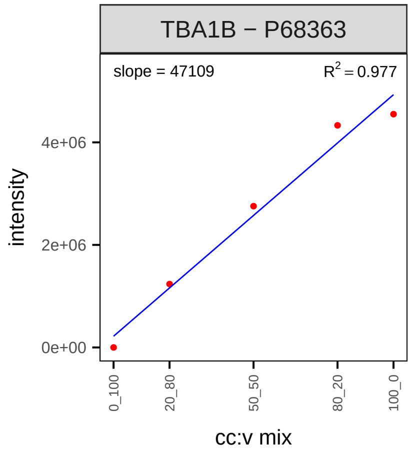
TBA1B − P68363
slope = 47109
$R^2 = 0.977$
intensity
4e+06
2e+06
0e+00
0_100
20_80
50_50
80_20
100_0
cc:v mix

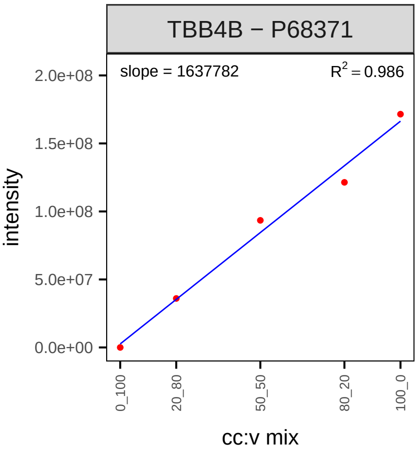
TBB4B − P68371
slope = 1637782
$R^2 = 0.986$
intensity
2.0e+08
1.5e+08
1.0e+08
5.0e+07
0.0e+00
0_100
20_80
50_50
80_20
100_0
cc:v mix

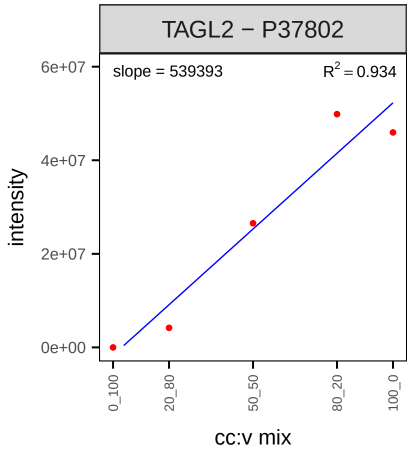
TAGL2 − P37802
slope = 539393
$R^2 = 0.934$
intensity
6e+07
4e+07
2e+07
0e+00
0_100
20_80
50_50
80_20
100_0
cc:v mix

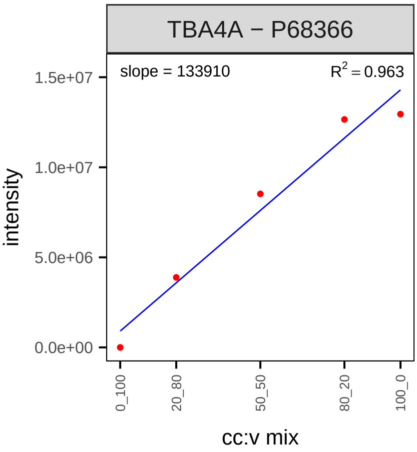
TBA4A − P68366
slope = 133910
$R^2 = 0.963$
intensity
1.5e+07
1.0e+07
5.0e+06
0.0e+00
0_100
20_80
50_50
80_20
100_0
cc:v mix

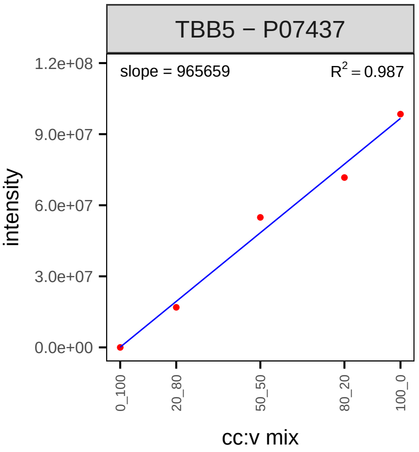
TBB5 − P07437
slope = 965659
$R^2 = 0.987$
intensity
1.2e+08
9.0e+07
6.0e+07
3.0e+07
0.0e+00
0_100
20_80
50_50
80_20
100_0
cc:v mix

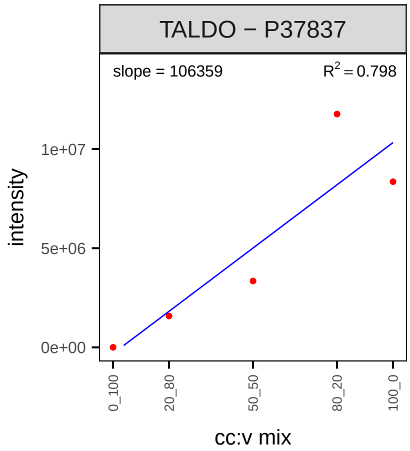
TALDO − P37837
slope = 106359
$R^2 = 0.798$
intensity
1e+07
5e+06
0e+00
0_100
20_80
50_50
80_20
100_0
cc:v mix

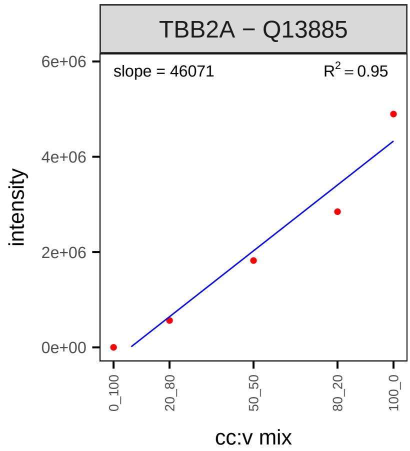
TBB2A − Q13885
slope = 46071
$R^2 = 0.95$
intensity
6e+06
4e+06
2e+06
0e+00
0_100
20_80
50_50
80_20
100_0
cc:v mix

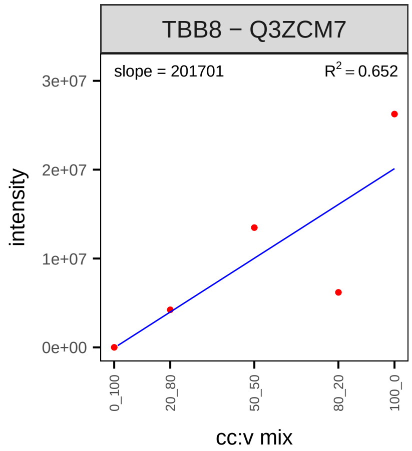
TBB8 − Q3ZCM7
slope = 201701
$R^2 = 0.652$
intensity
3e+07
2e+07
1e+07
0e+00
0_100
20_80
50_50
80_20
100_0
cc:v mix

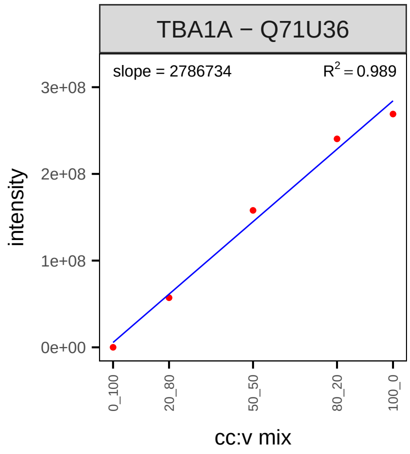
TBA1A − Q71U36
slope = 2786734
$R^2 = 0.989$
intensity
3e+08
2e+08
1e+08
0e+00
0_100
20_80
50_50
80_20
100_0
cc:v mix

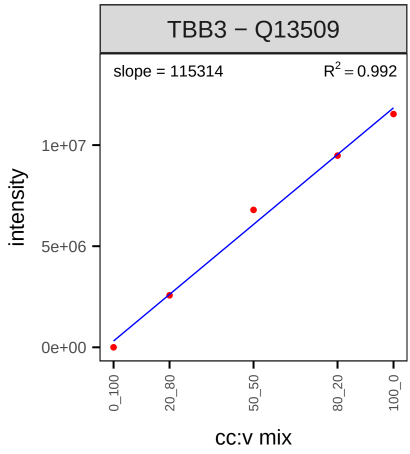
TBB3 − Q13509
slope = 115314
$R^2 = 0.992$
intensity
1e+07
5e+06
0e+00
0_100
20_80
50_50
80_20
100_0
cc:v mix

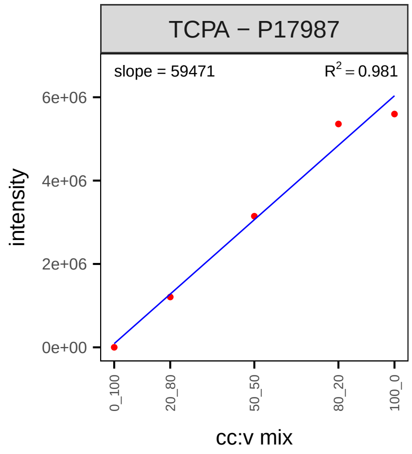
TCPA − P17987
slope = 59471
$R^2 = 0.981$
intensity
6e+06
4e+06
2e+06
0e+00
0_100
20_80
50_50
80_20
100_0
cc:v mix

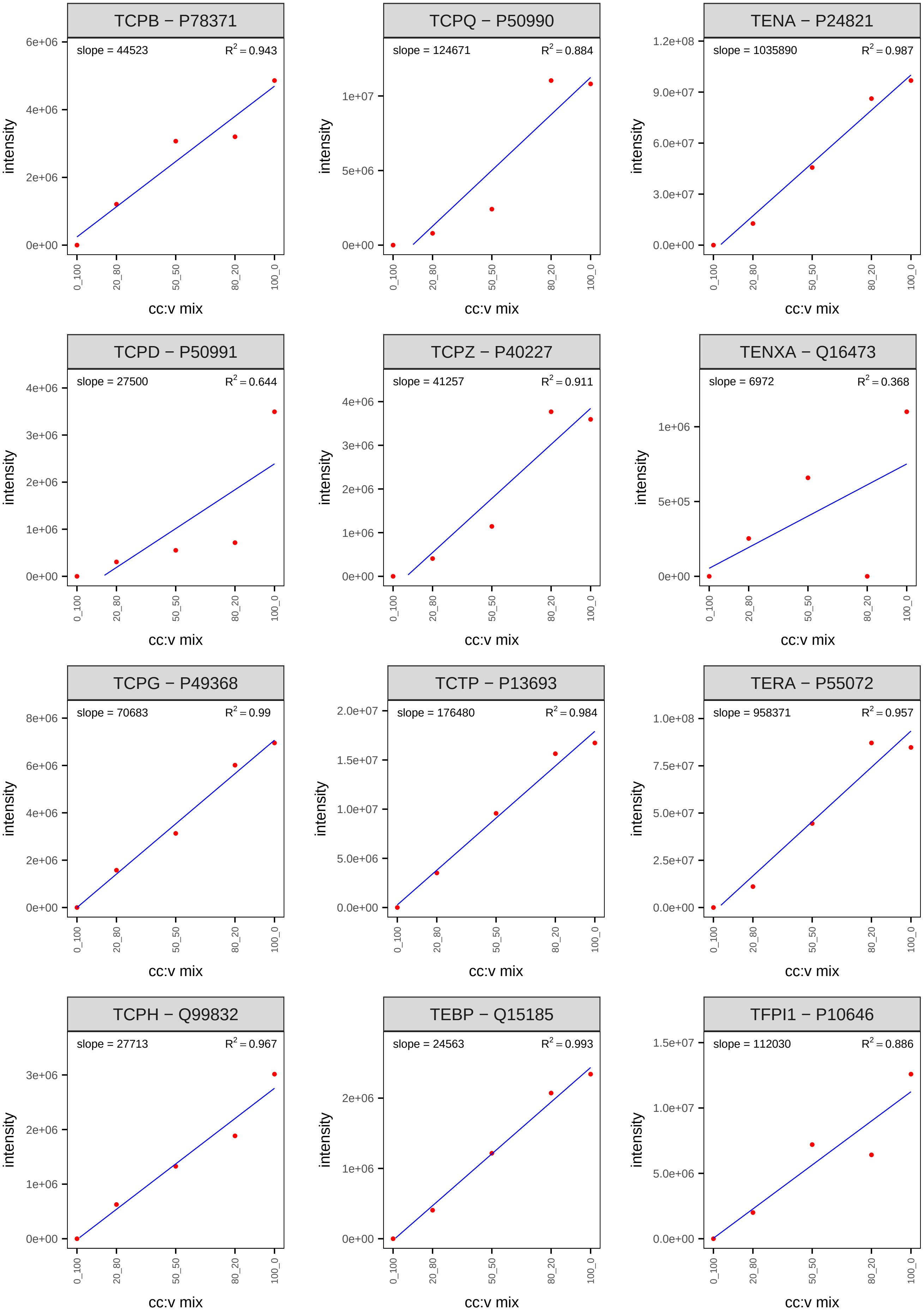
TCPB − P78371
slope = 44523
$R^2 = 0.943$
TCPQ − P50990
slope = 124671
$R^2 = 0.884$
TENA − P24821
slope = 1035890
$R^2 = 0.987$
TCPD − P50991
slope = 27500
$R^2 = 0.644$
TCPZ − P40227
slope = 41257
$R^2 = 0.911$
TENXA − Q16473
slope = 6972
$R^2 = 0.368$
TCPG − P49368
slope = 70683
$R^2 = 0.99$
TCTP − P13693
slope = 176480
$R^2 = 0.984$
TERA − P55072
slope = 958371
$R^2 = 0.957$
TCPH − Q99832
slope = 27713
$R^2 = 0.967$
TEBP − Q15185
slope = 24563
$R^2 = 0.993$
TFPI1 − P10646
slope = 112030
$R^2 = 0.886$
intensity
cc:v mix
0_100
20_80
50_50
80_20
100_0

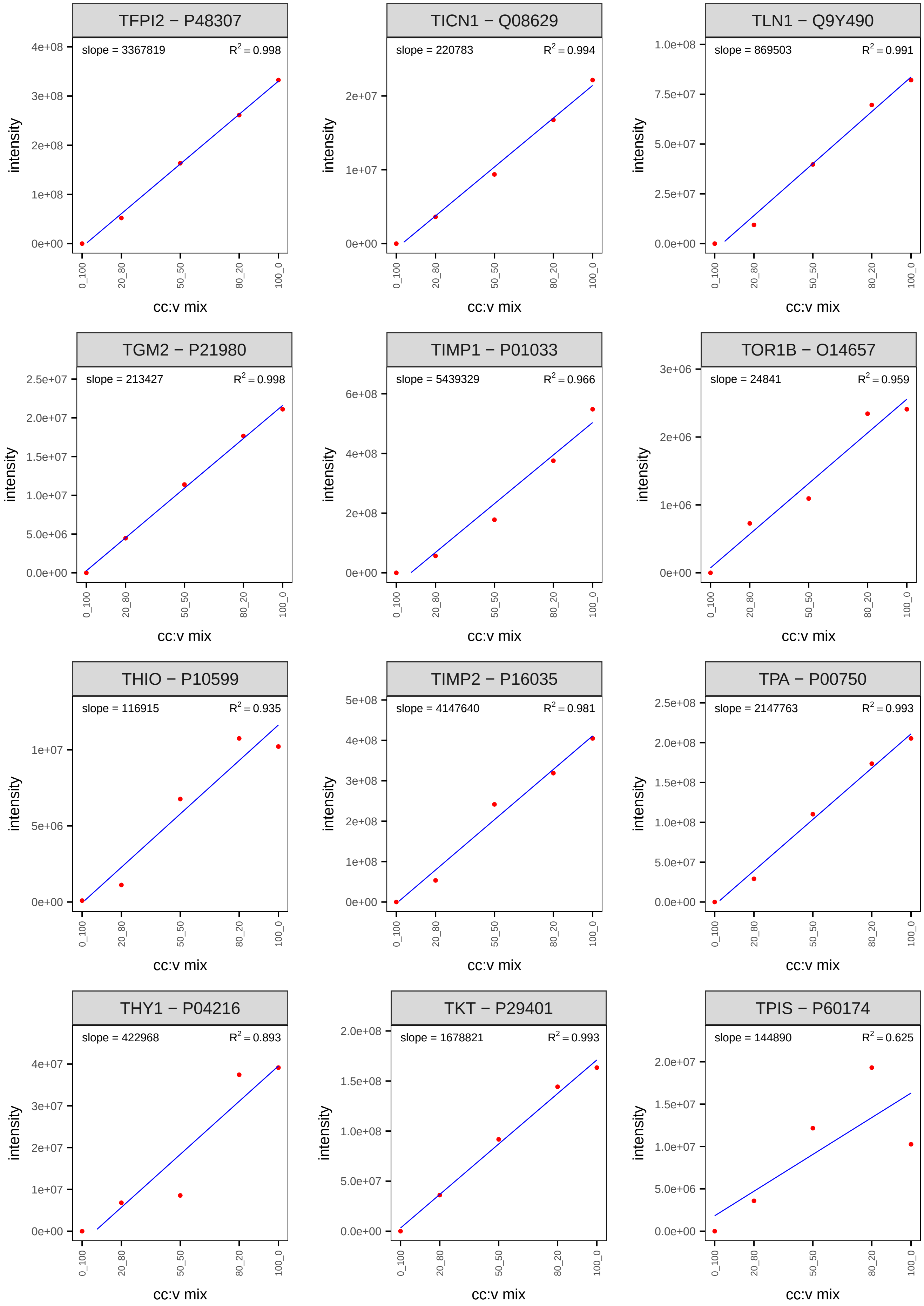
TFPI2 − P48307
slope = 3367819
R² = 0.998
TICN1 − Q08629
slope = 220783
R² = 0.994
TLN1 − Q9Y490
slope = 869503
R² = 0.991
TGM2 − P21980
slope = 213427
R² = 0.998
TIMP1 − P01033
slope = 5439329
R² = 0.966
TOR1B − O14657
slope = 24841
R² = 0.959
THIO − P10599
slope = 116915
R² = 0.935
TIMP2 − P16035
slope = 4147640
R² = 0.981
TPA − P00750
slope = 2147763
R² = 0.993
THY1 − P04216
slope = 422968
R² = 0.893
TKT − P29401
slope = 1678821
R² = 0.993
TPIS − P60174
slope = 144890
R² = 0.625
intensity
cc:v mix
0_100
20_80
50_50
80_20
100_0

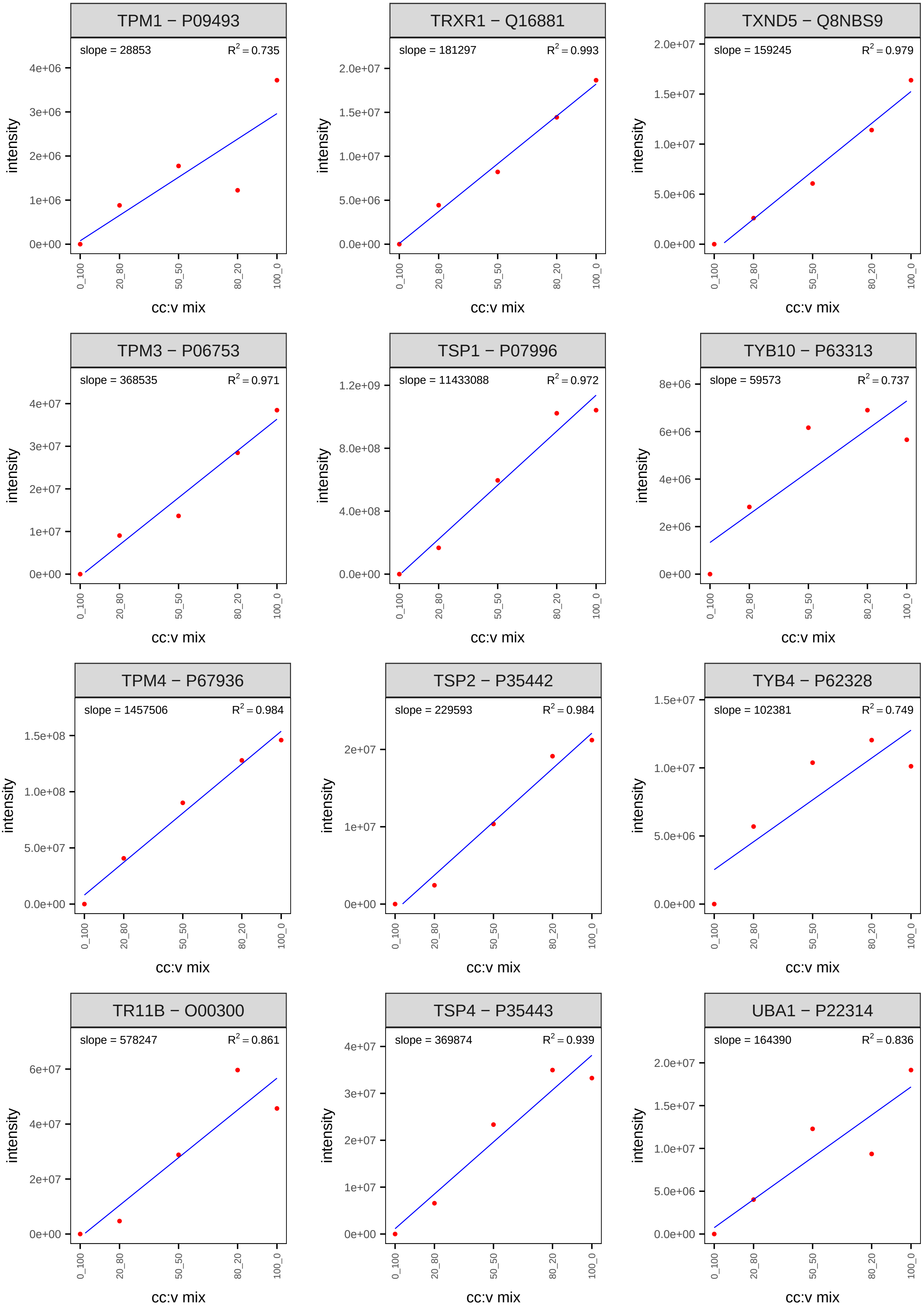
TPM1 − P09493
slope = 28853
R² = 0.735
TRXR1 − Q16881
slope = 181297
R² = 0.993
TXND5 − Q8NBS9
slope = 159245
R² = 0.979
TPM3 − P06753
slope = 368535
R² = 0.971
TSP1 − P07996
slope = 11433088
R² = 0.972
TYB10 − P63313
slope = 59573
R² = 0.737
TPM4 − P67936
slope = 1457506
R² = 0.984
TSP2 − P35442
slope = 229593
R² = 0.984
TYB4 − P62328
slope = 102381
R² = 0.749
TR11B − O00300
slope = 578247
R² = 0.861
TSP4 − P35443
slope = 369874
R² = 0.939
UBA1 − P22314
slope = 164390
R² = 0.836
intensity
cc:v mix
0_100
20_80
50_50
80_20
100_0

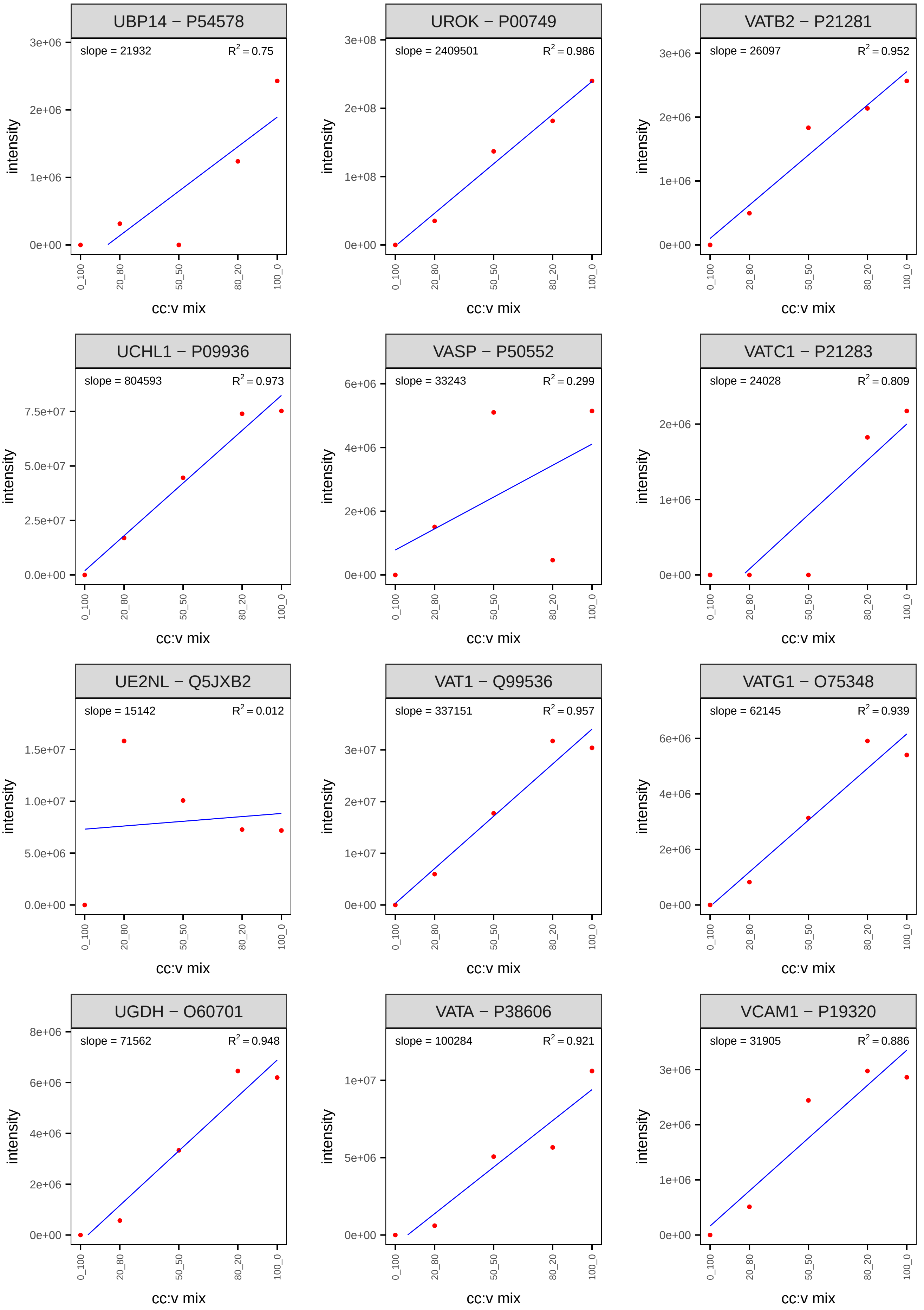
UBP14 − P54578
slope = 21932
$R^2 = 0.75$
UROK − P00749
slope = 2409501
$R^2 = 0.986$
VATB2 − P21281
slope = 26097
$R^2 = 0.952$
UCHL1 − P09936
slope = 804593
$R^2 = 0.973$
VASP − P50552
slope = 33243
$R^2 = 0.299$
VATC1 − P21283
slope = 24028
$R^2 = 0.809$
UE2NL − Q5JXB2
slope = 15142
$R^2 = 0.012$
VAT1 − Q99536
slope = 337151
$R^2 = 0.957$
VATG1 − O75348
slope = 62145
$R^2 = 0.939$
UGDH − O60701
slope = 71562
$R^2 = 0.948$
VATA − P38606
slope = 100284
$R^2 = 0.921$
VCAM1 − P19320
slope = 31905
$R^2 = 0.886$
intensity
cc:v mix
0_100
20_80
50_50
80_20
100_0

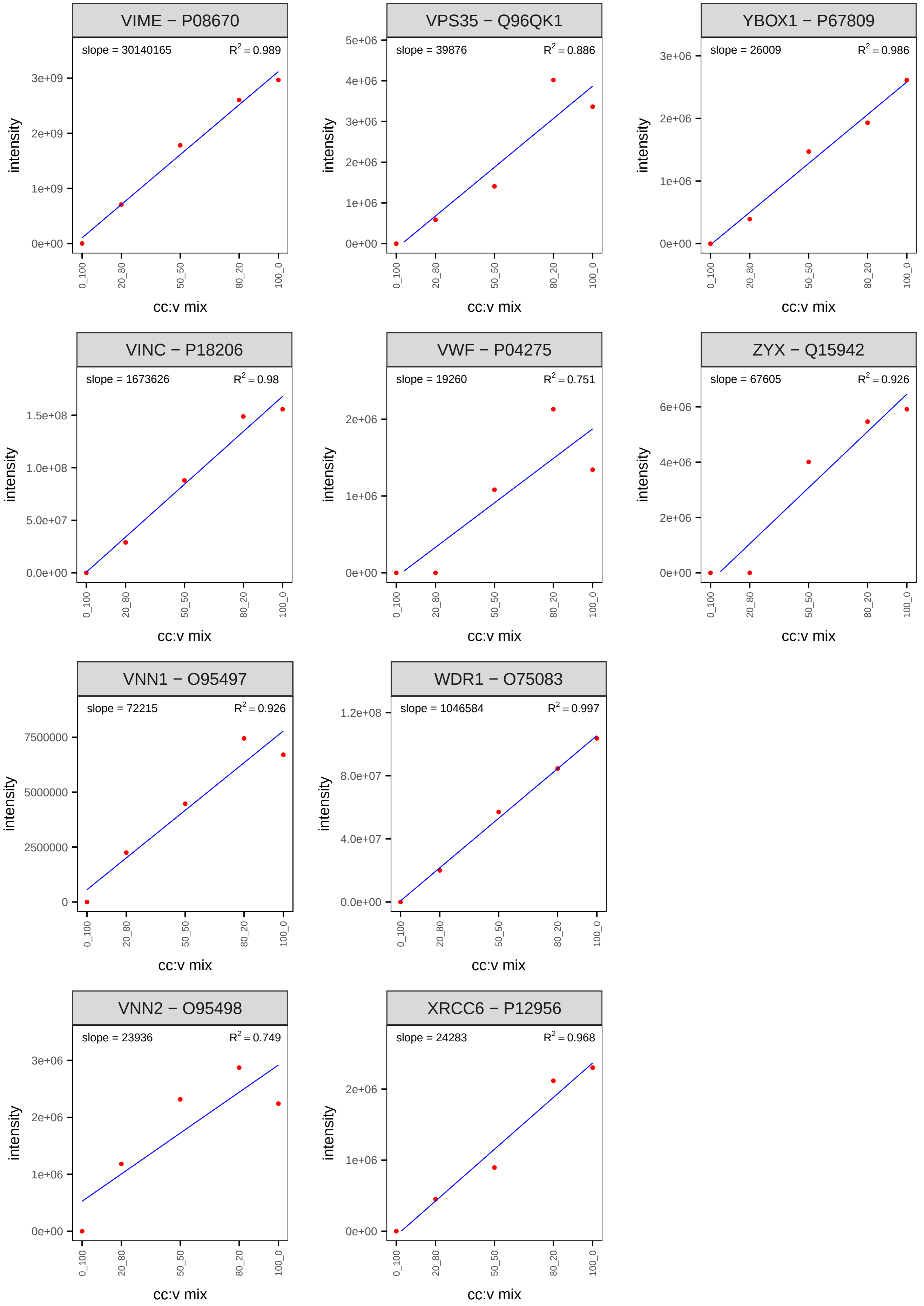
VIME − P08670
slope = 30140165
R^2 = 0.989
VPS35 − Q96QK1
slope = 39876
R^2 = 0.886
YBOX1 − P67809
slope = 26009
R^2 = 0.986
VINC − P18206
slope = 1673626
R^2 = 0.98
VWF − P04275
slope = 19260
R^2 = 0.751
ZYX − Q15942
slope = 67605
R^2 = 0.926
VNN1 − O95497
slope = 72215
R^2 = 0.926
WDR1 − O75083
slope = 1046584
R^2 = 0.997
VNN2 − O95498
slope = 23936
R^2 = 0.749
XRCC6 − P12956
slope = 24283
R^2 = 0.968
intensity
cc:v mix
0_100
20_80
50_50
80_20
100_0
