# Supplementary figures and images for: Stable Isotope Dynamic Labeling of Secretomes (SIDLS) Identifies Authentic Secretory Proteins Released by Cancer and Stromal Cells
Source: Mol Cell Proteomics. 2018 Jun 18;17(9):1837–49. doi: 10.1074/mcp.TIR117.000516 (PMC6126392; doi:10.1074/mcp.TIR117.000516)

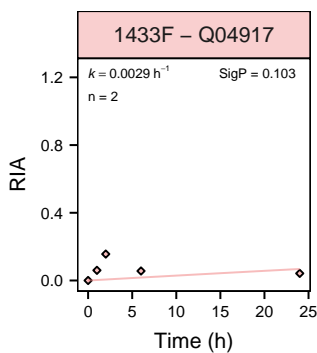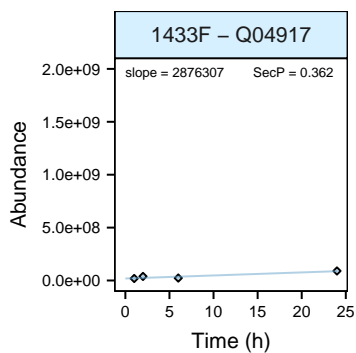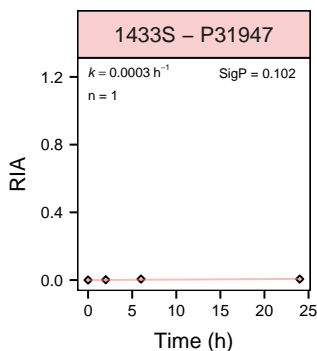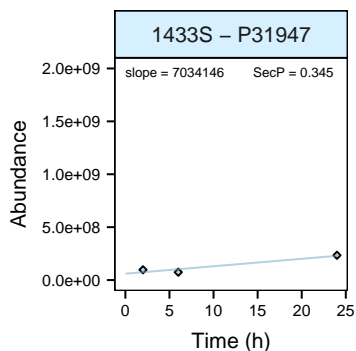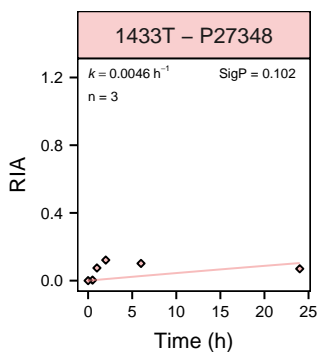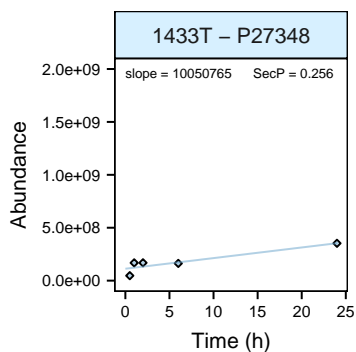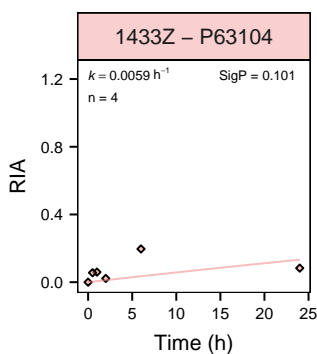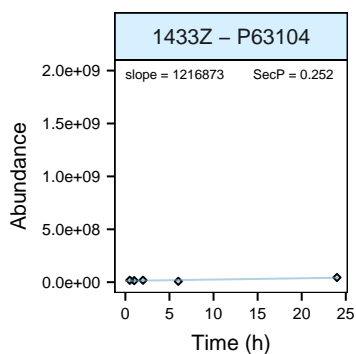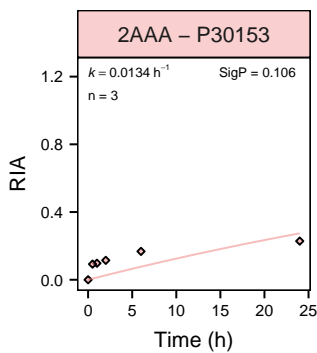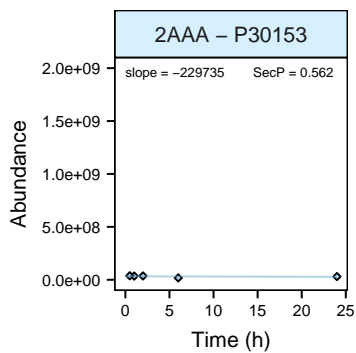

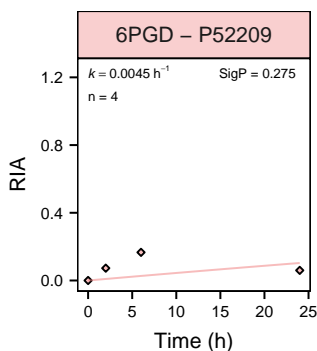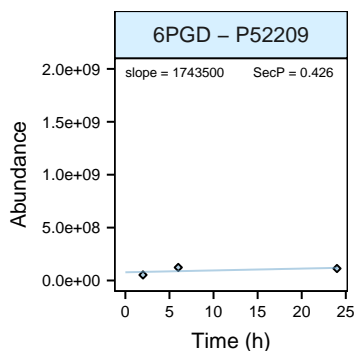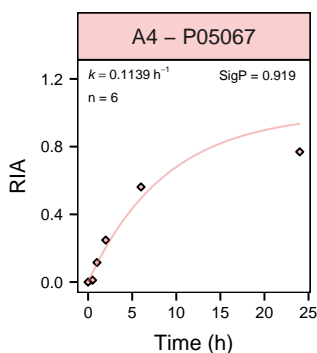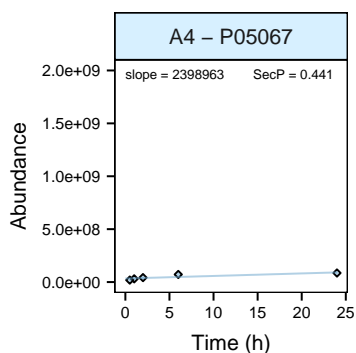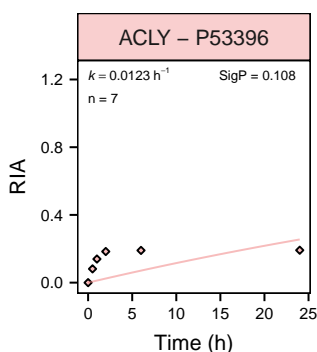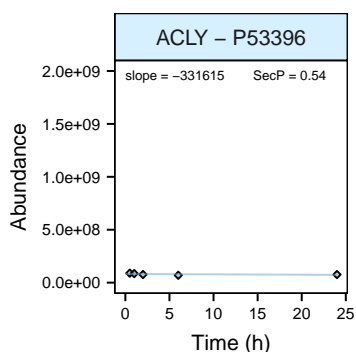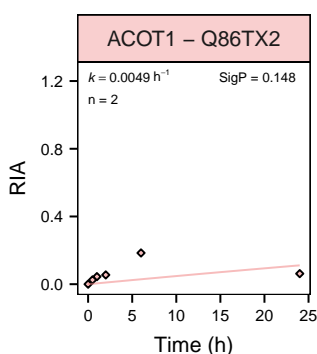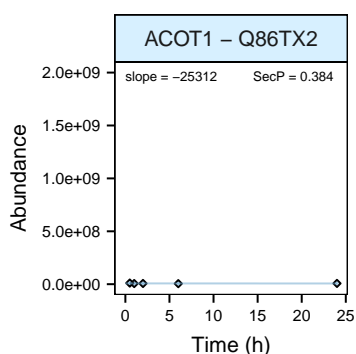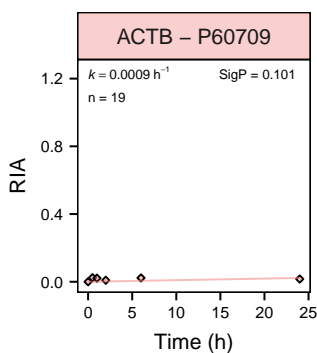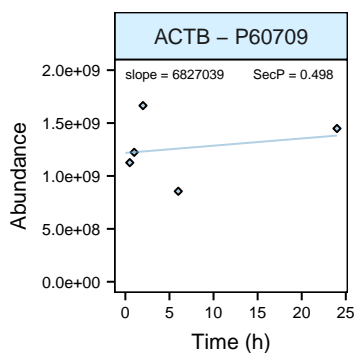

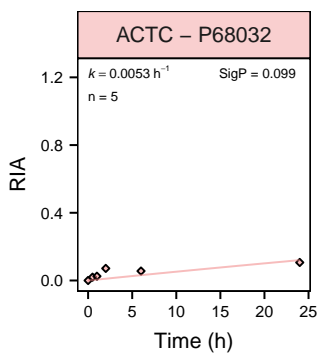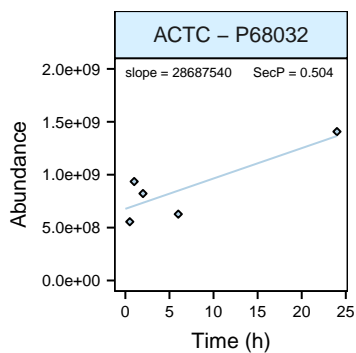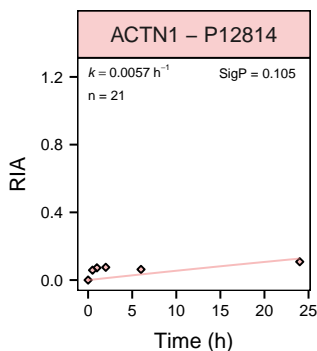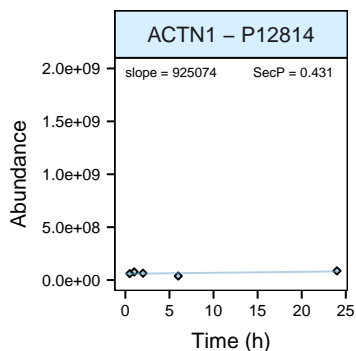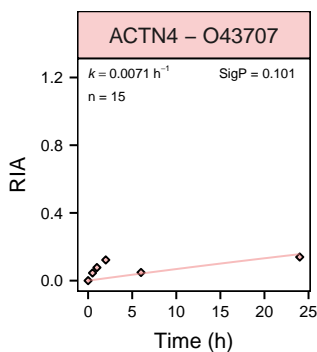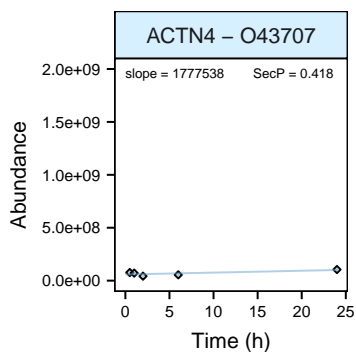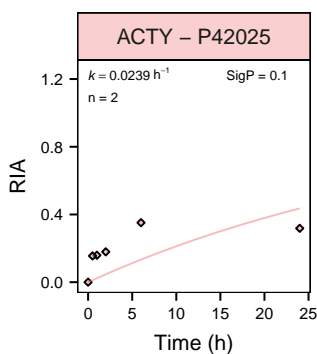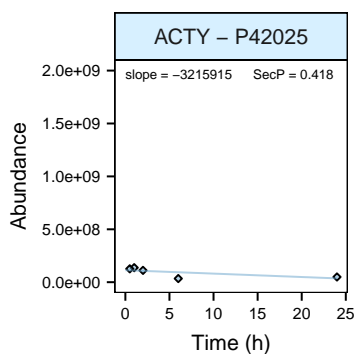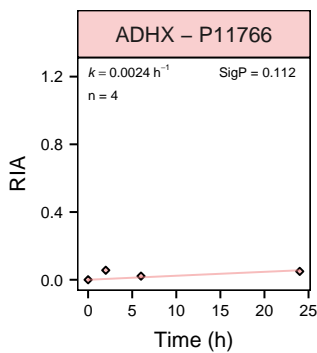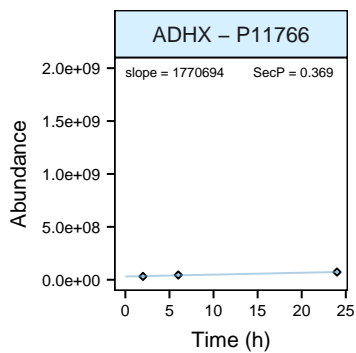

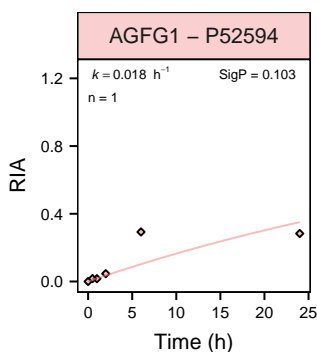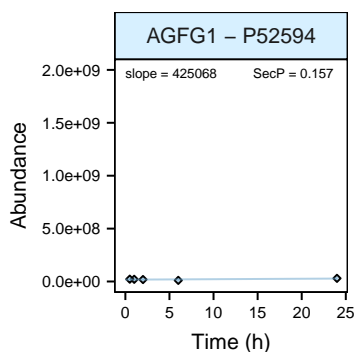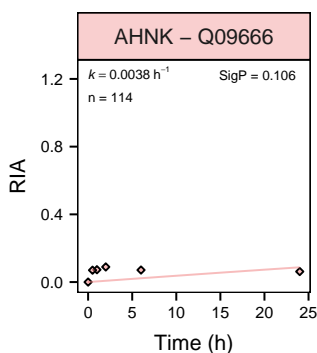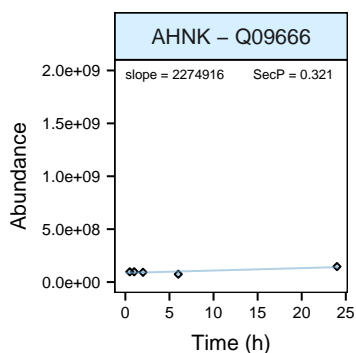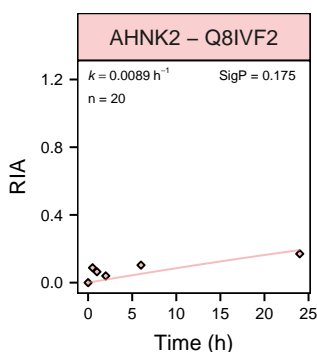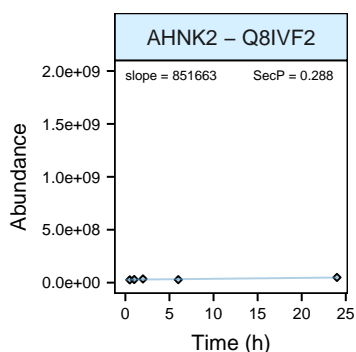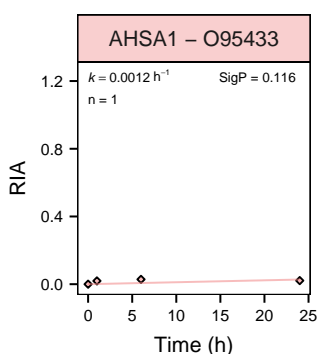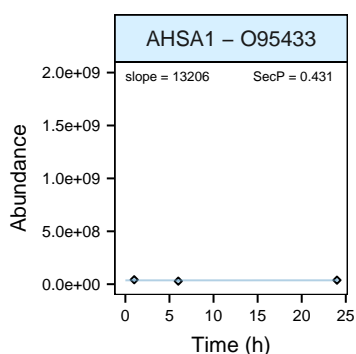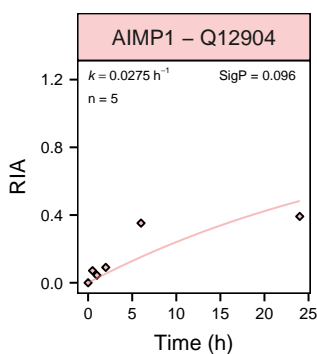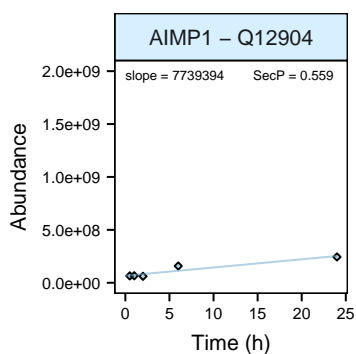

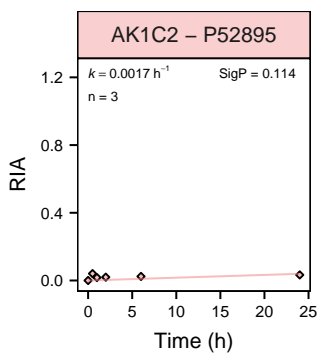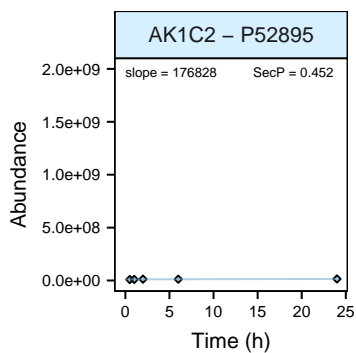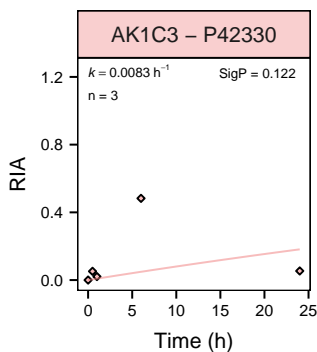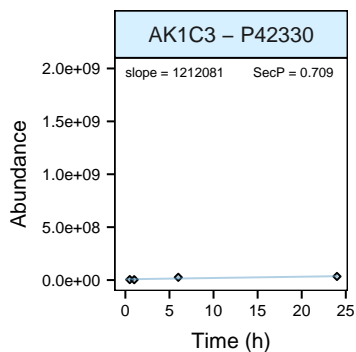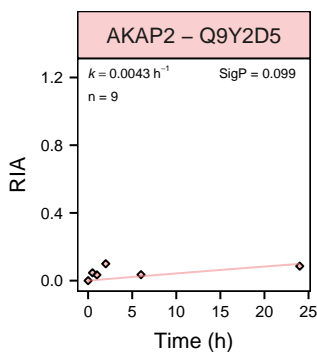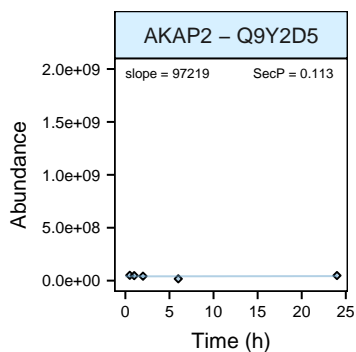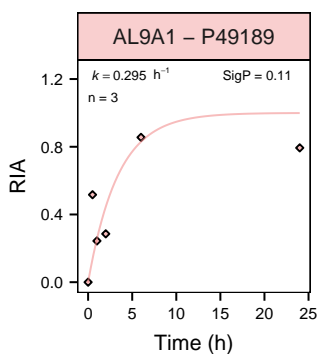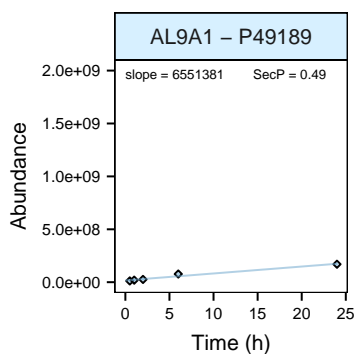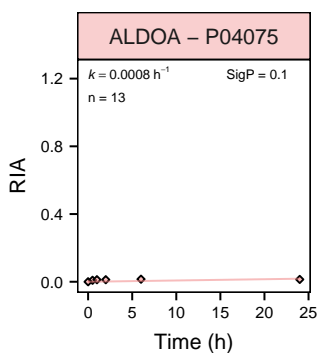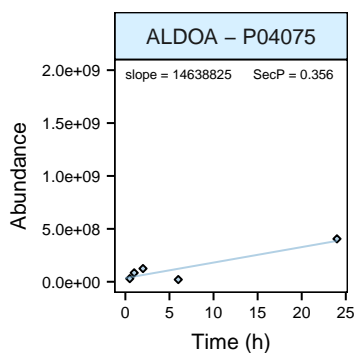

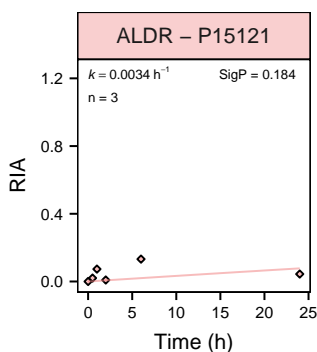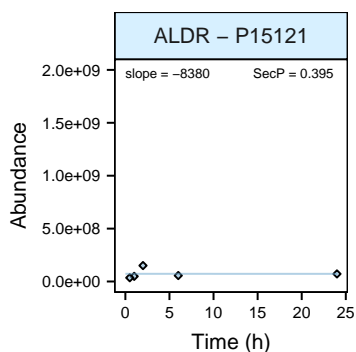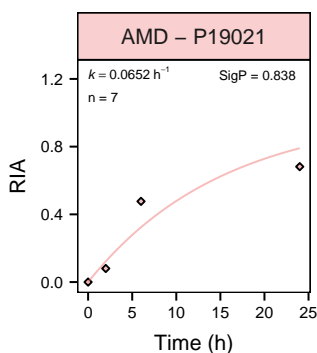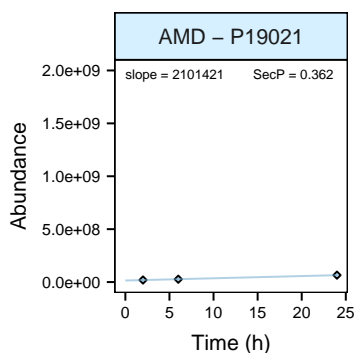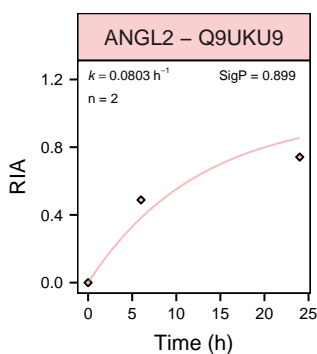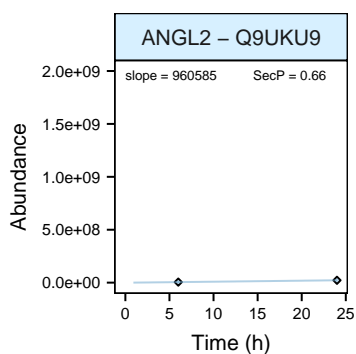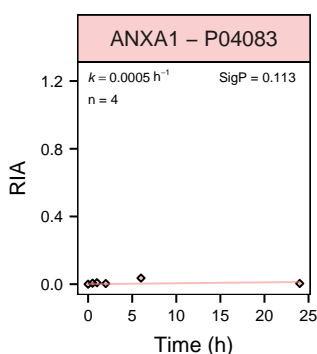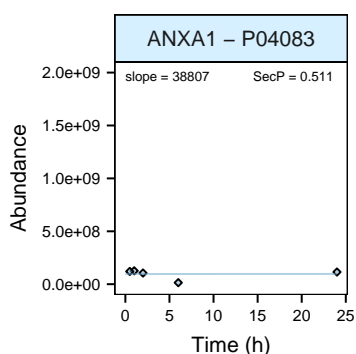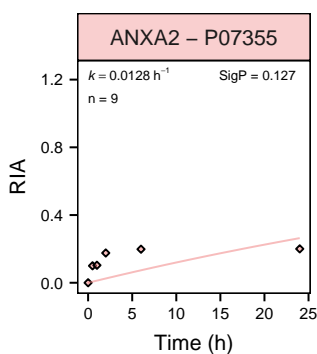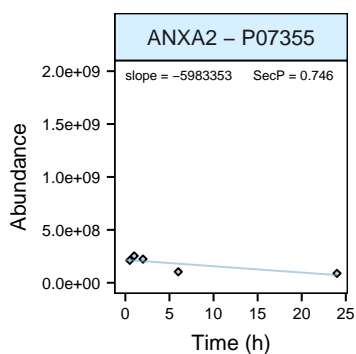

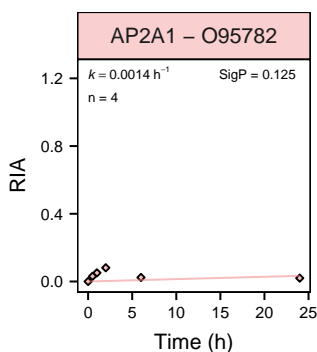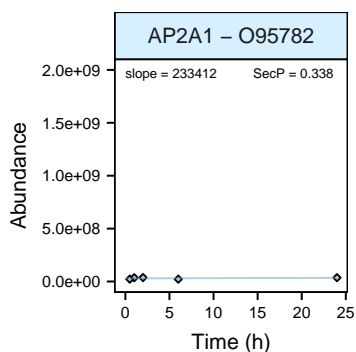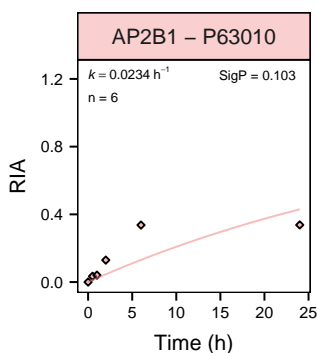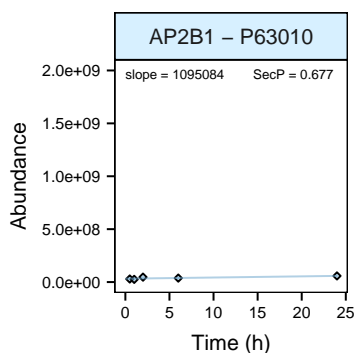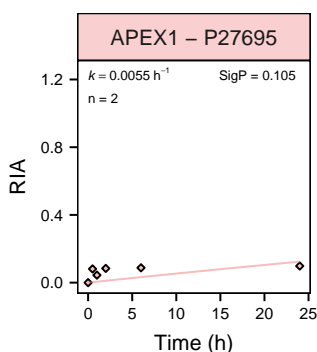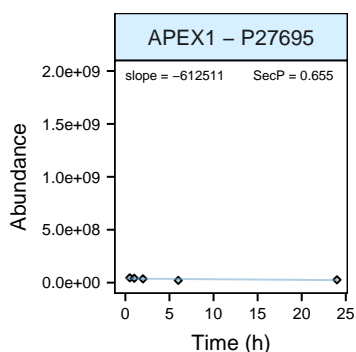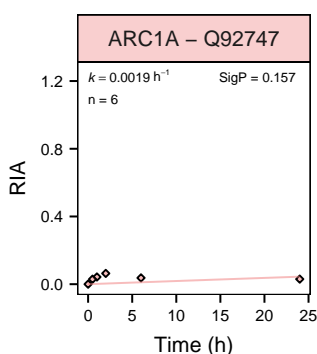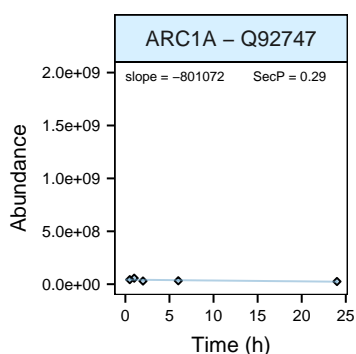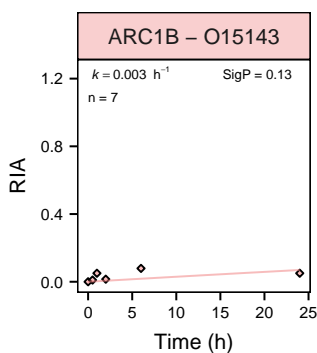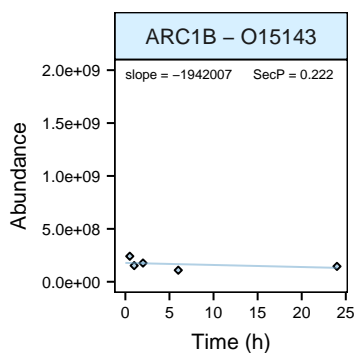

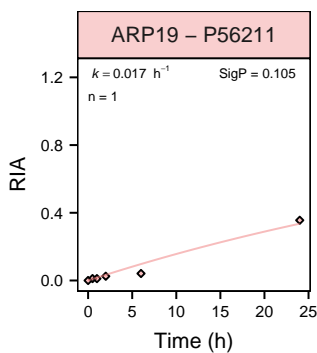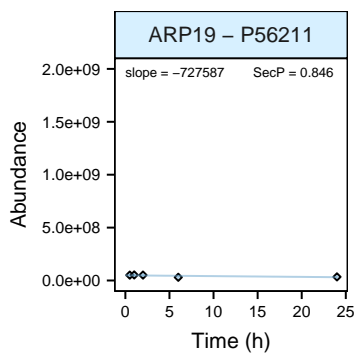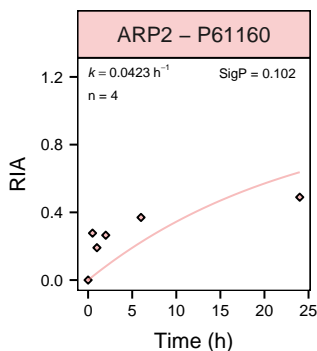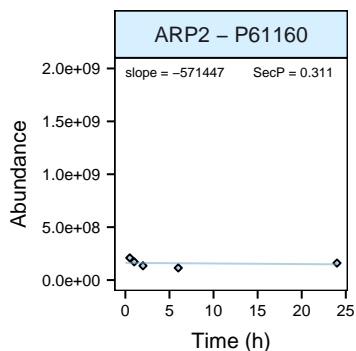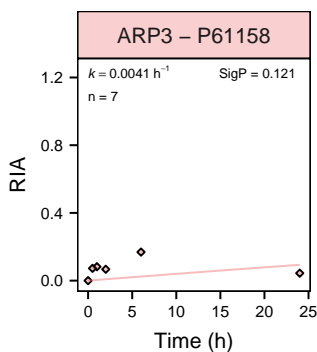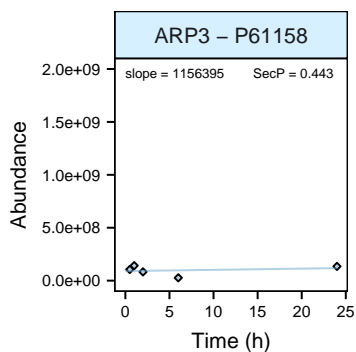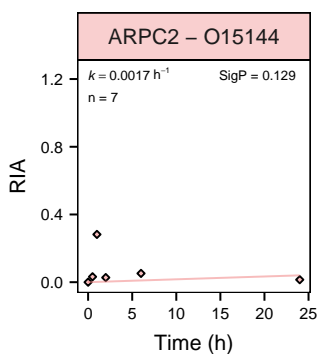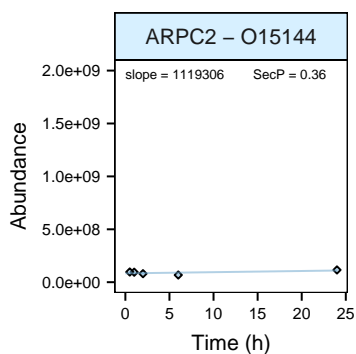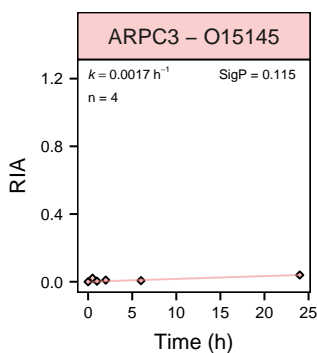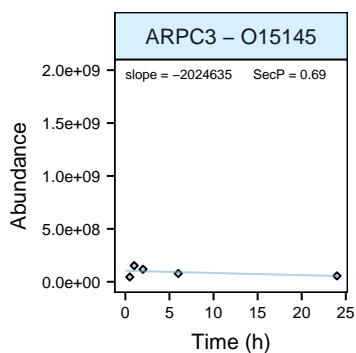

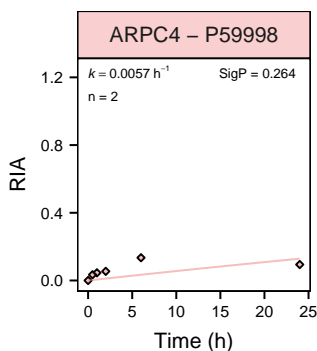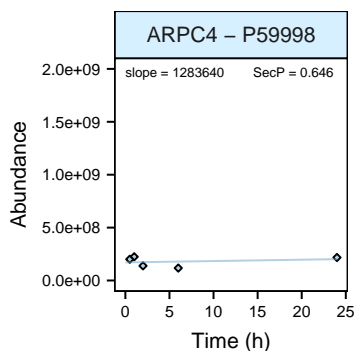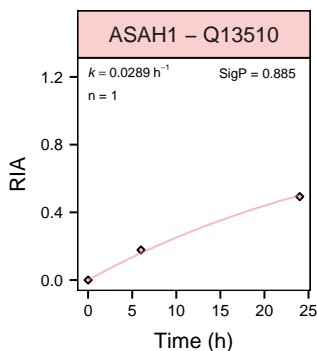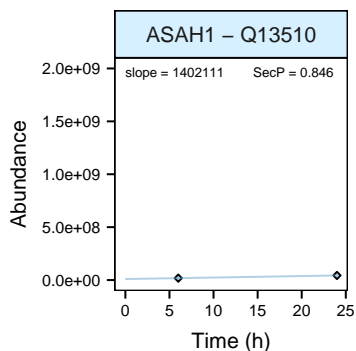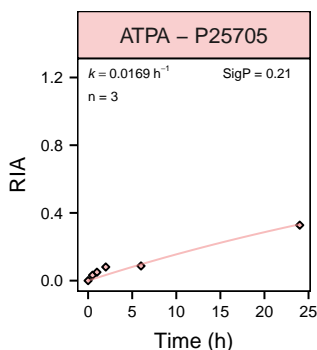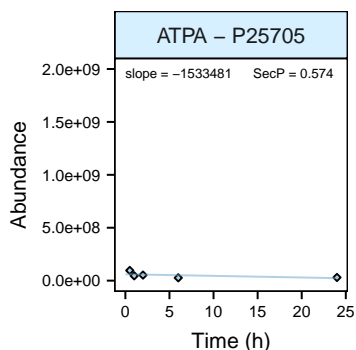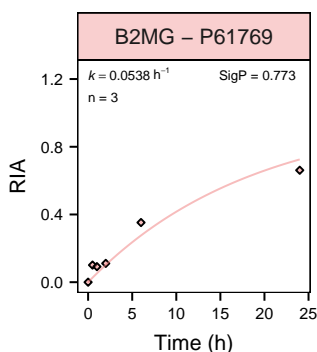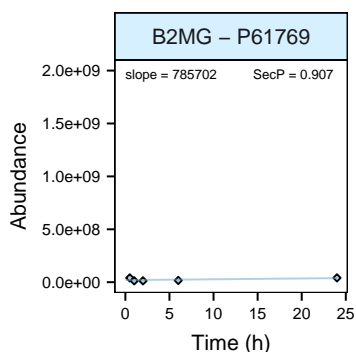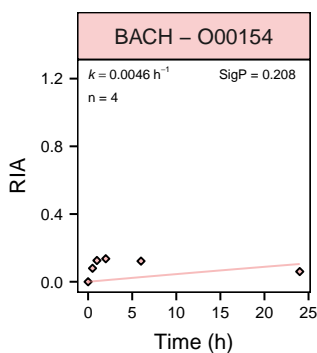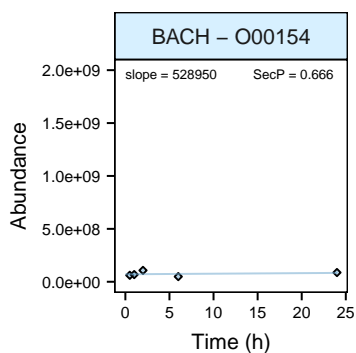

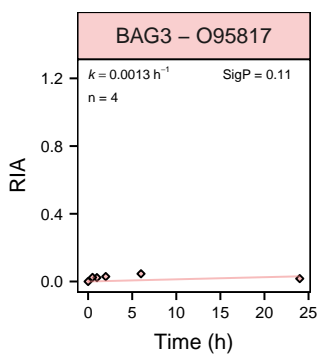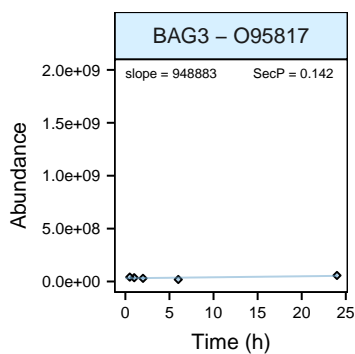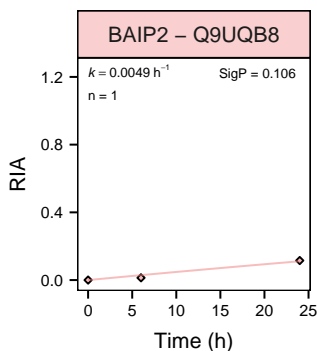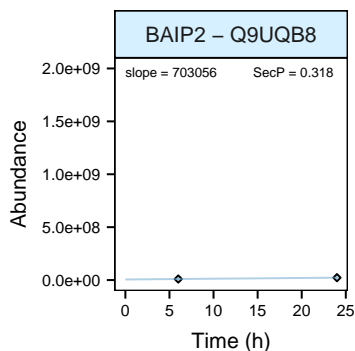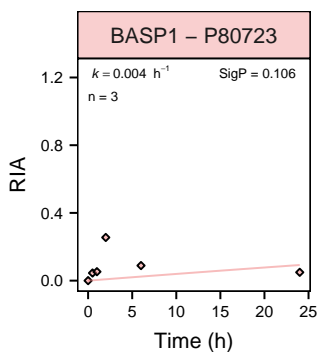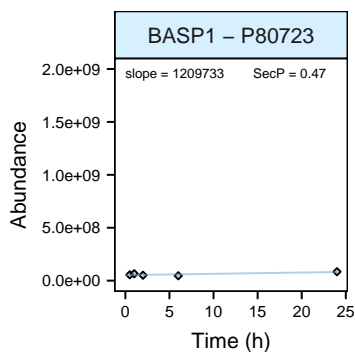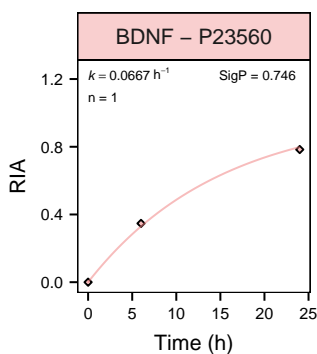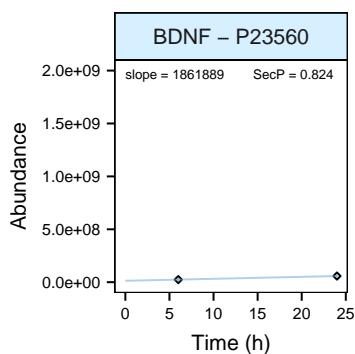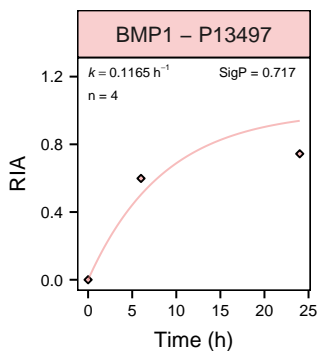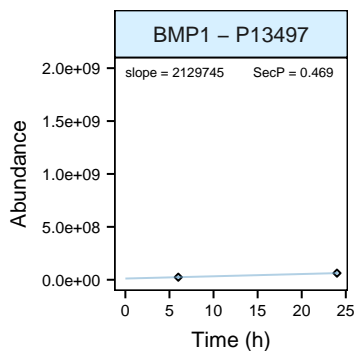

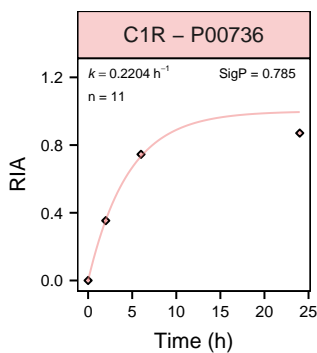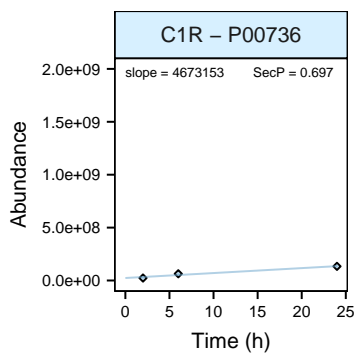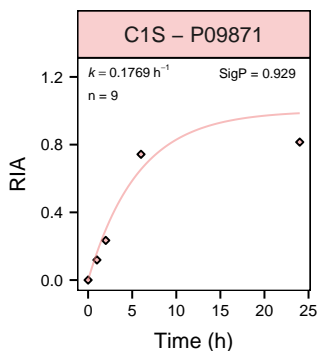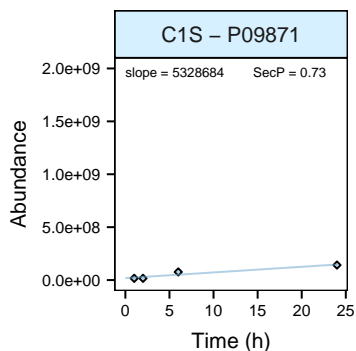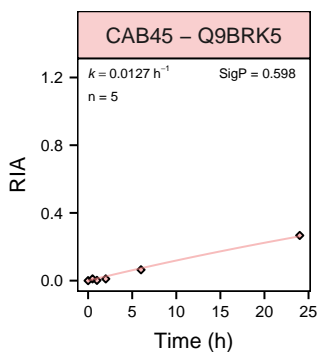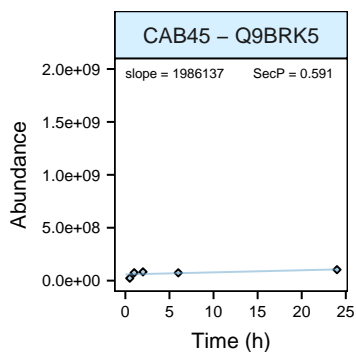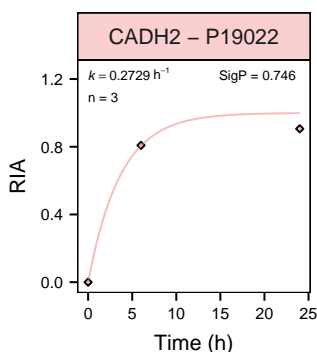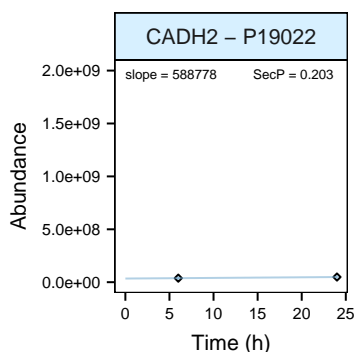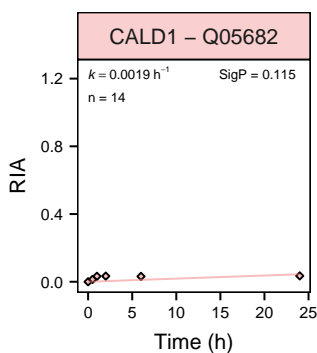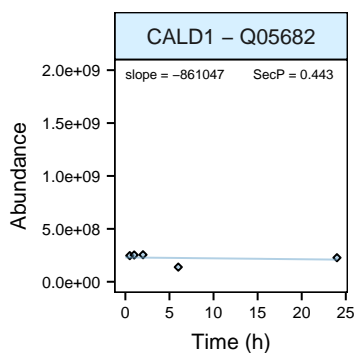

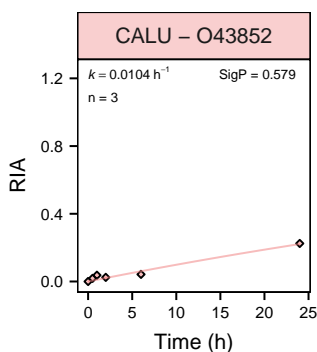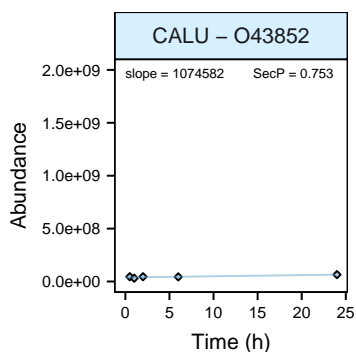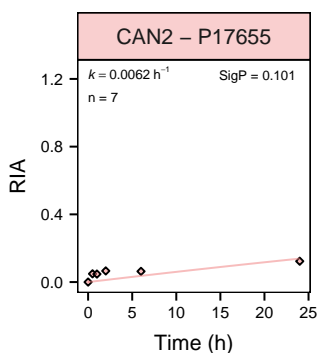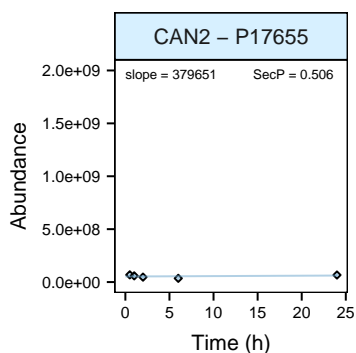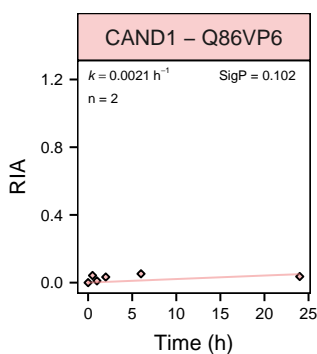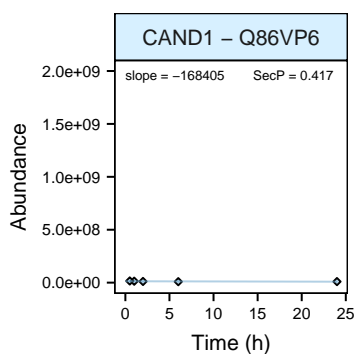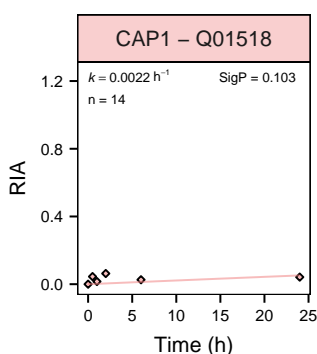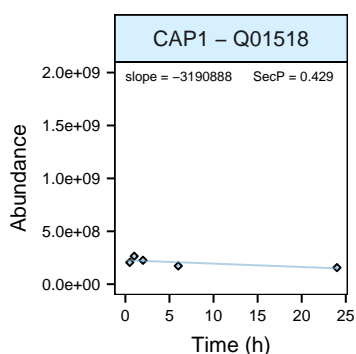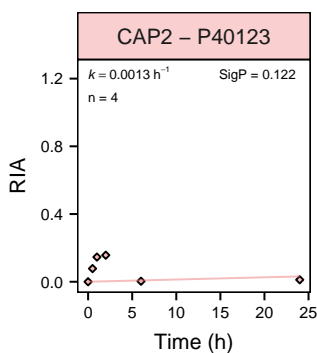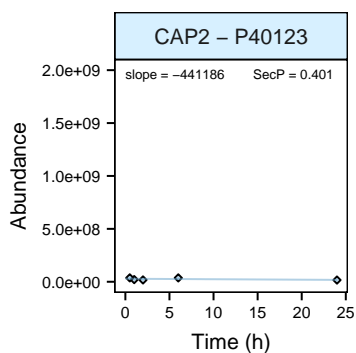

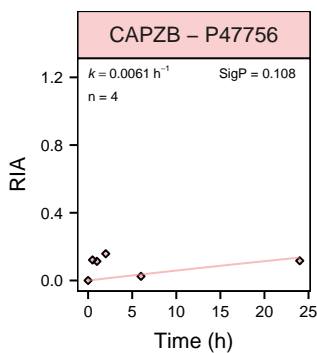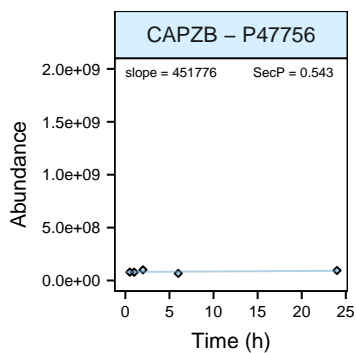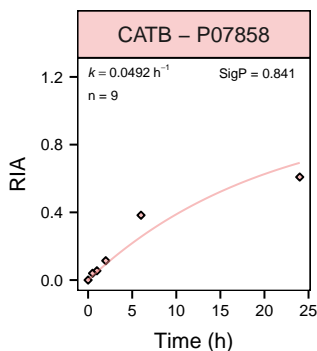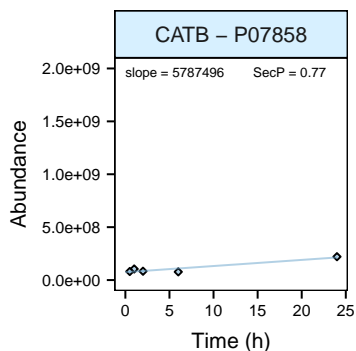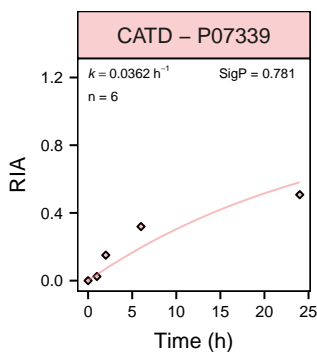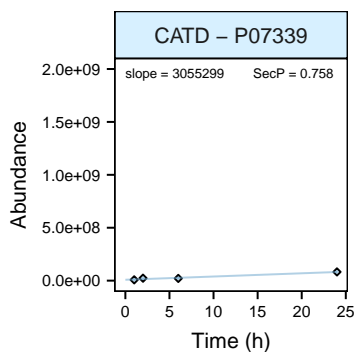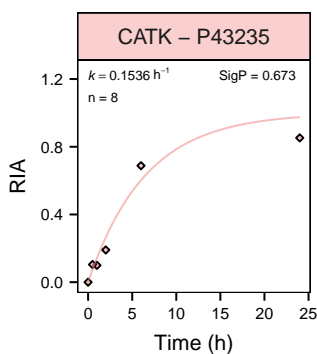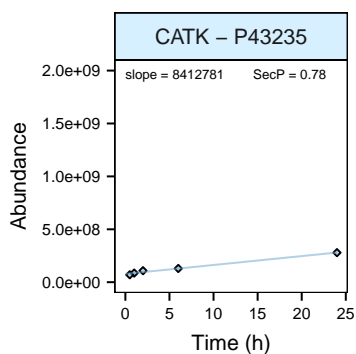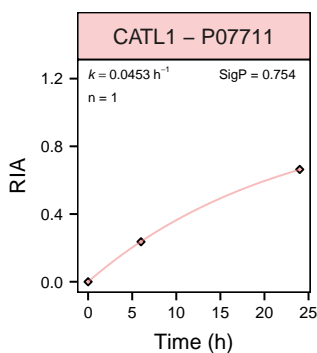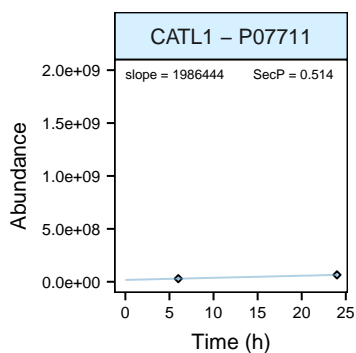

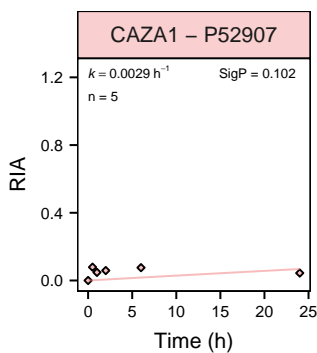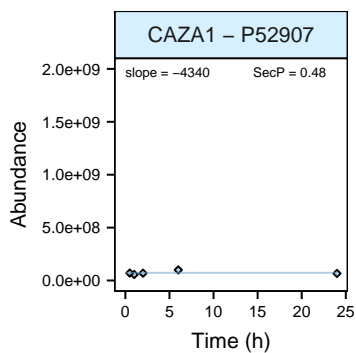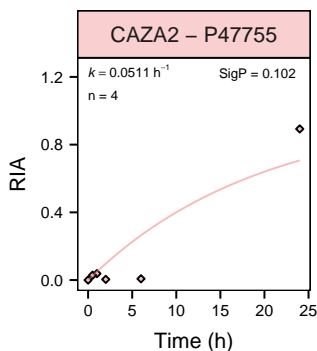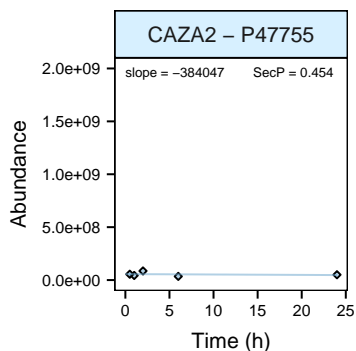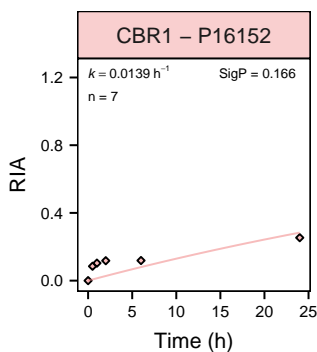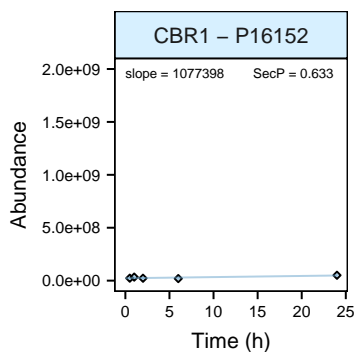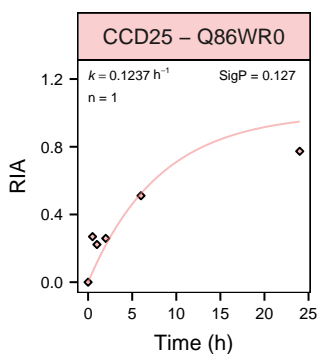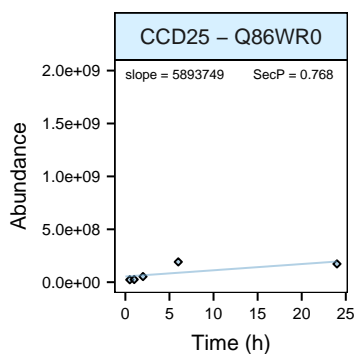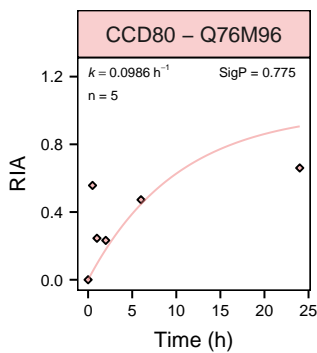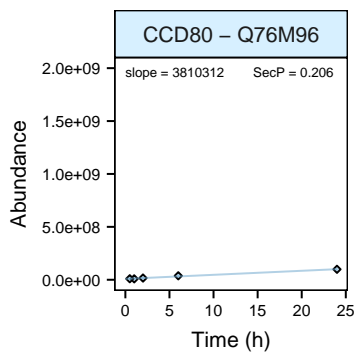

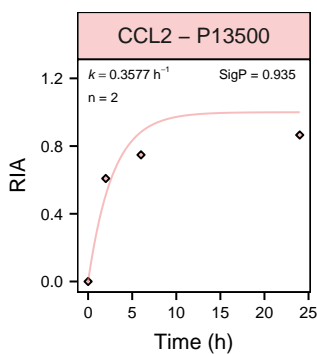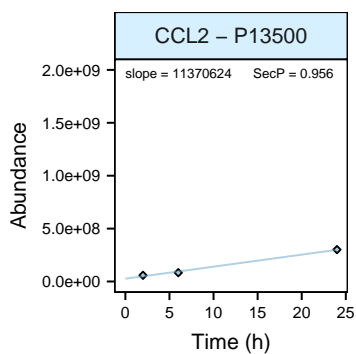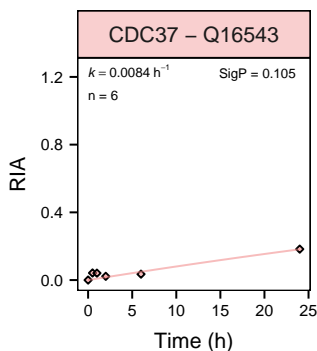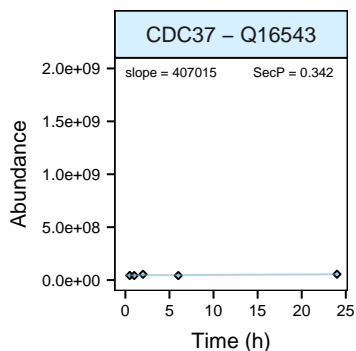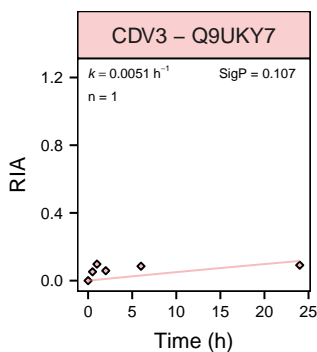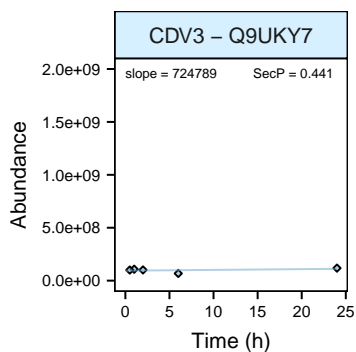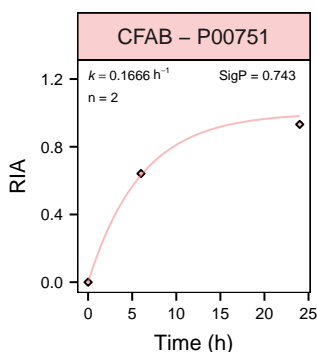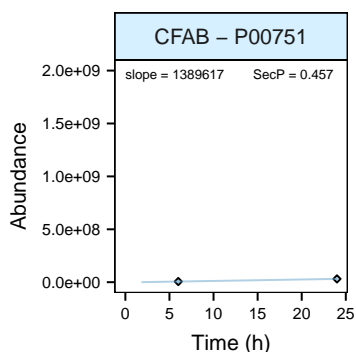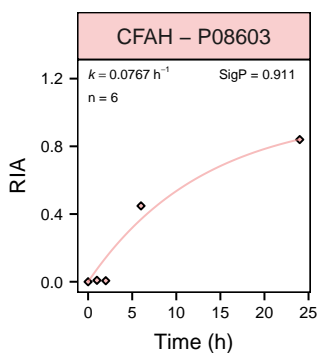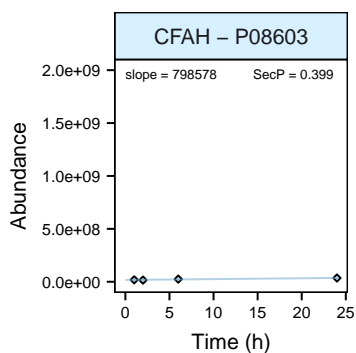

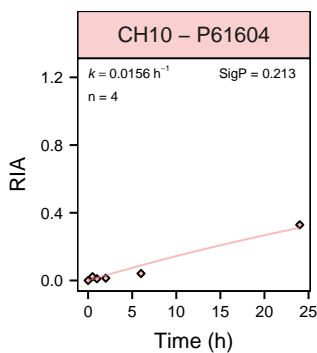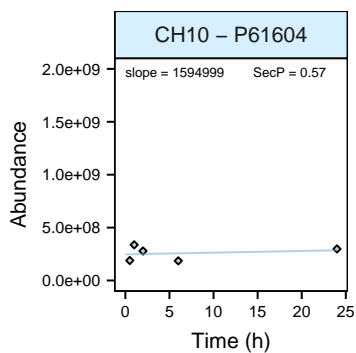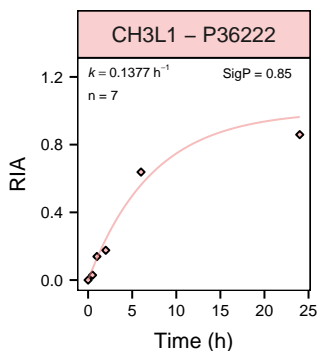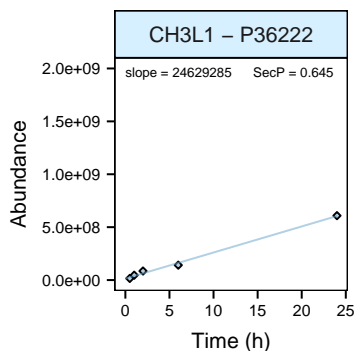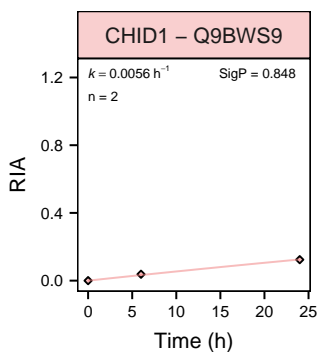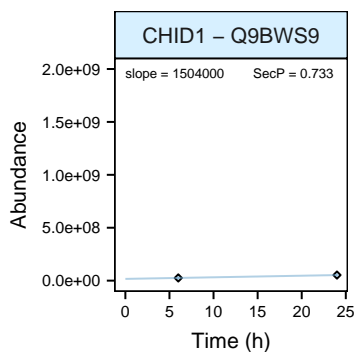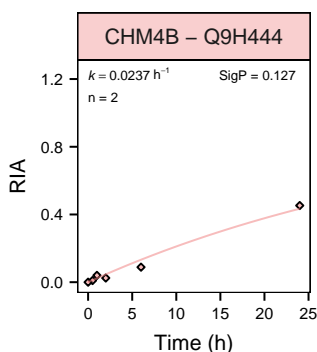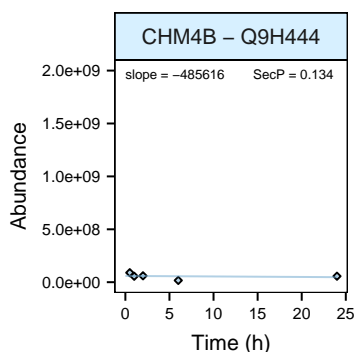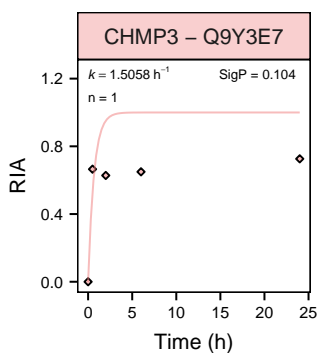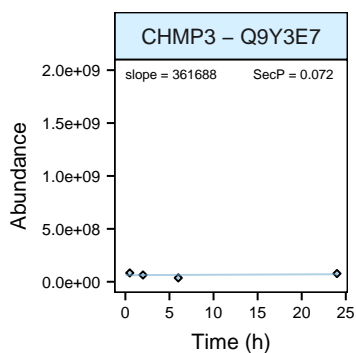

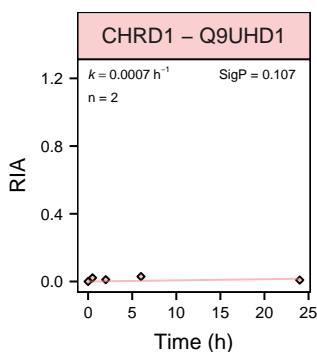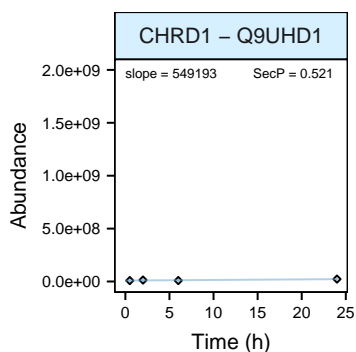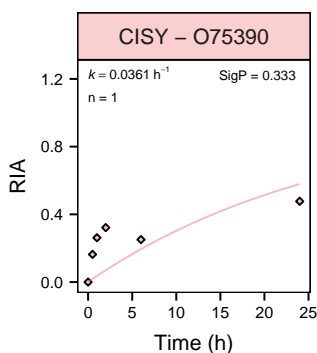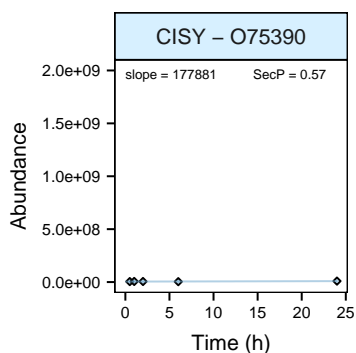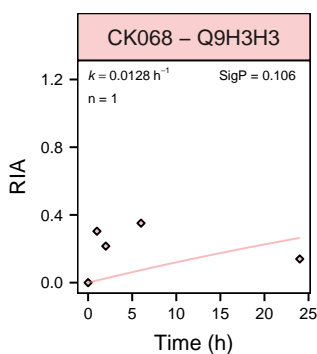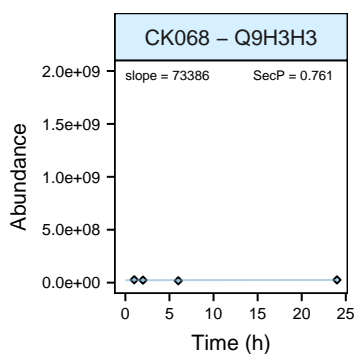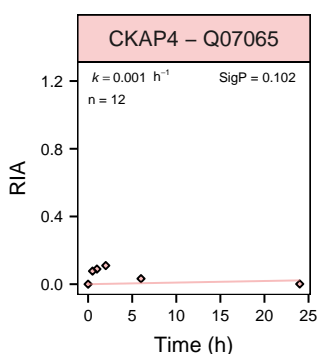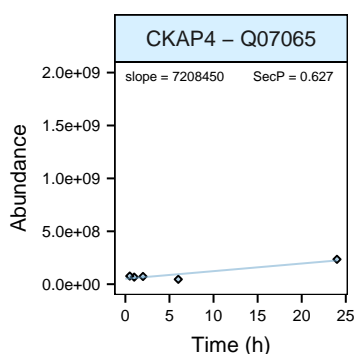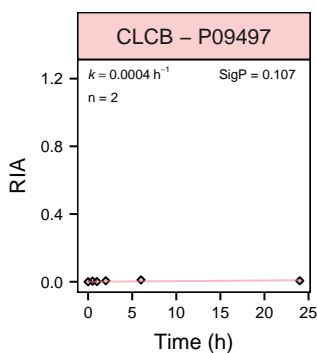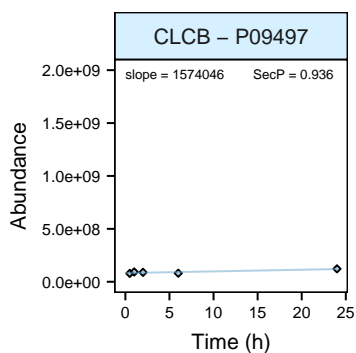

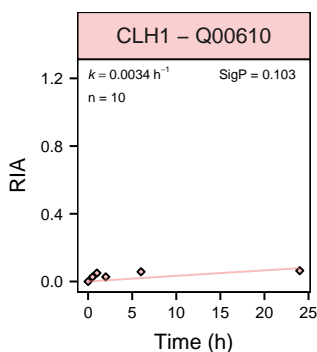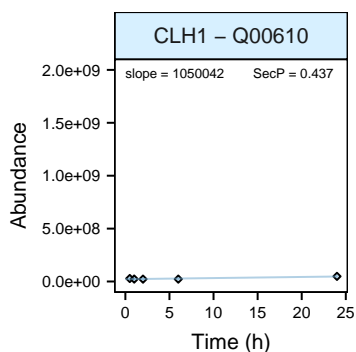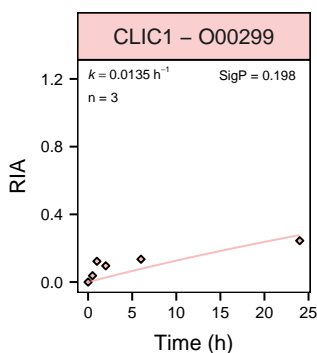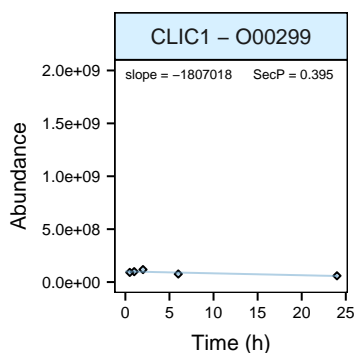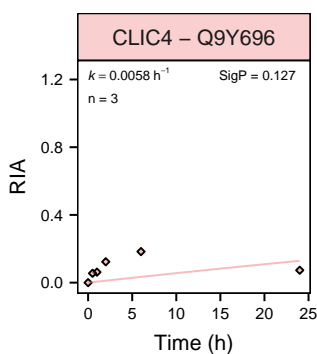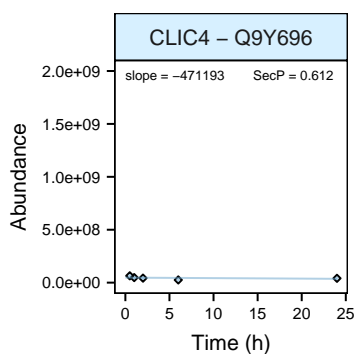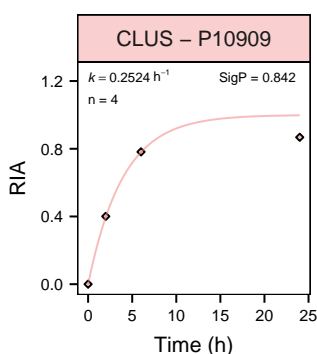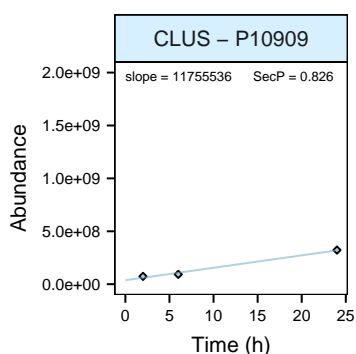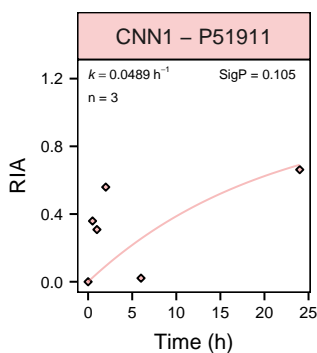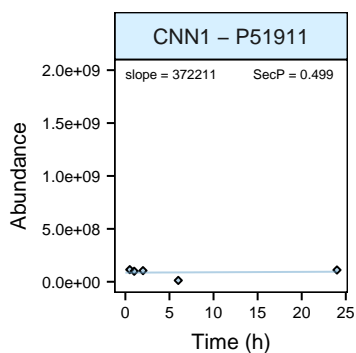

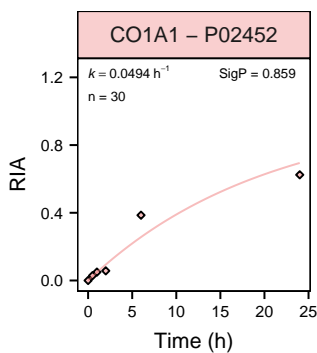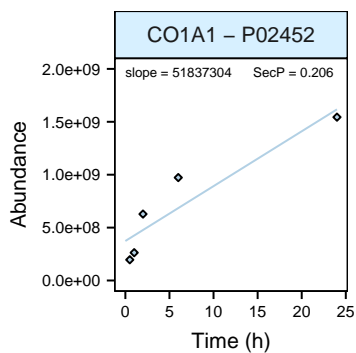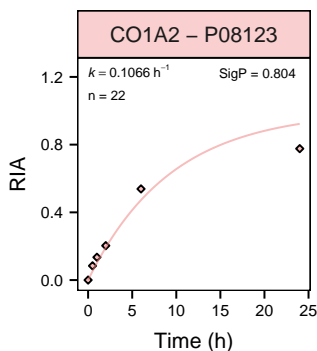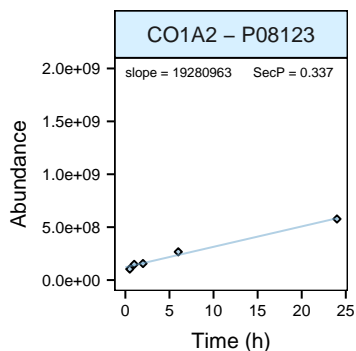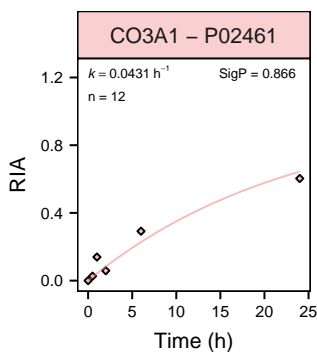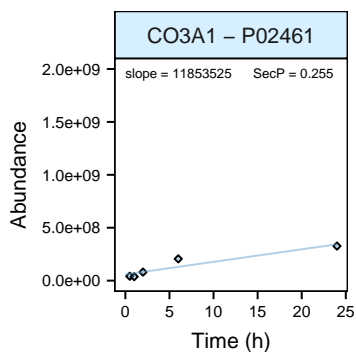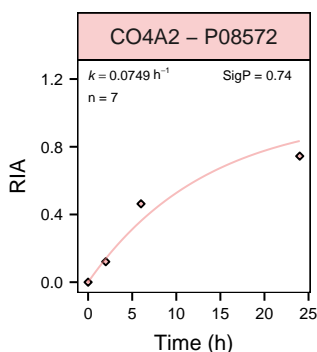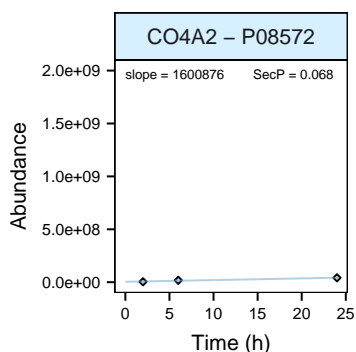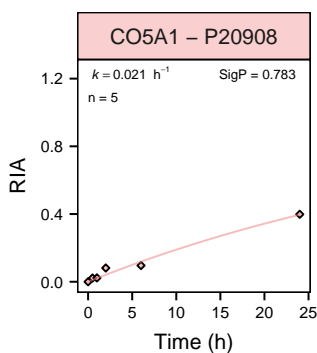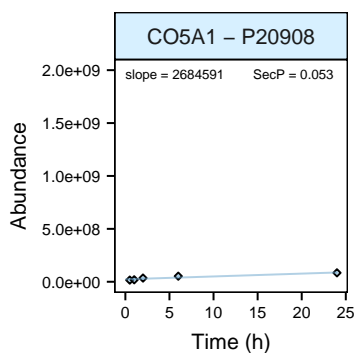

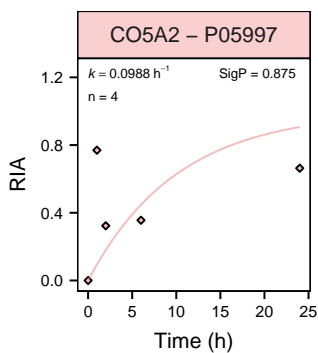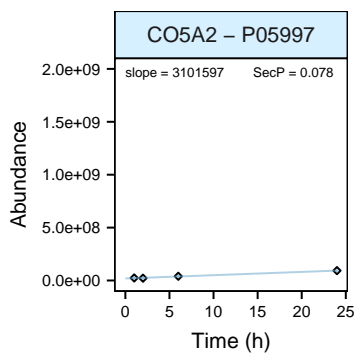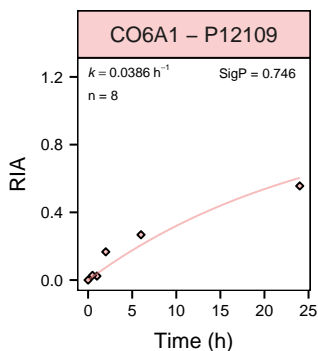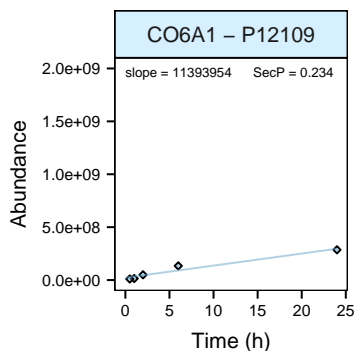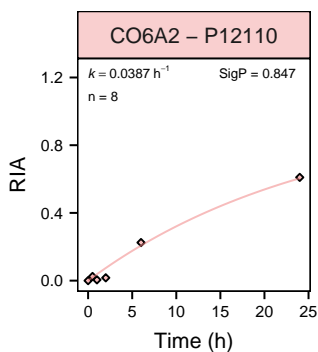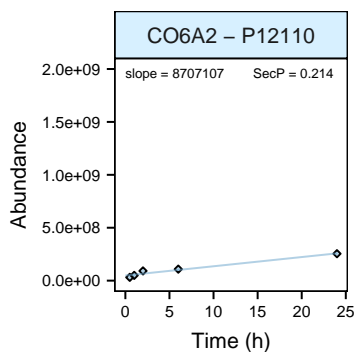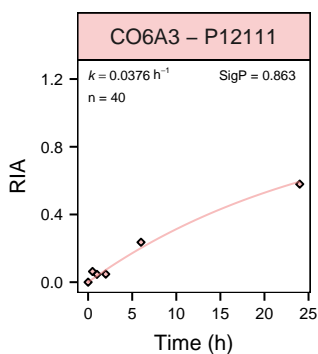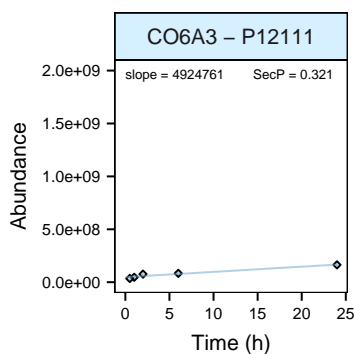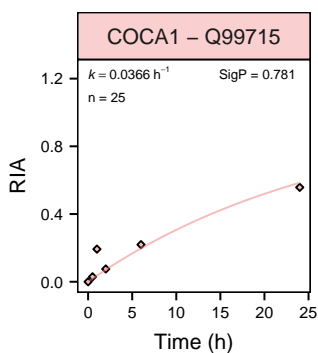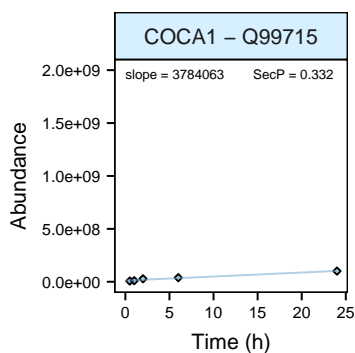

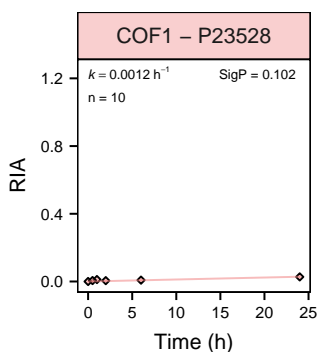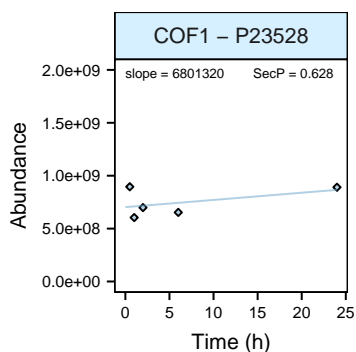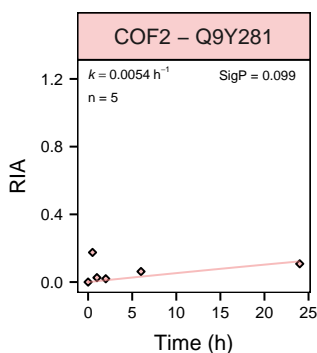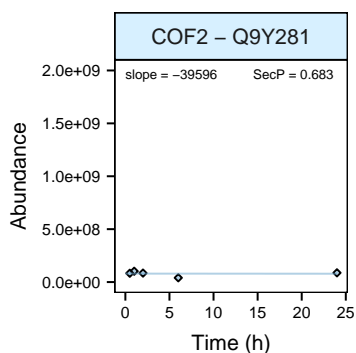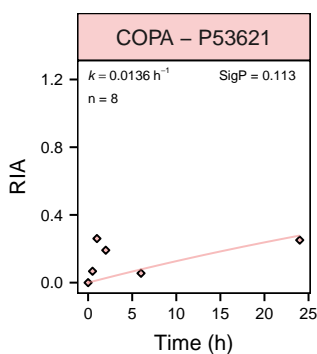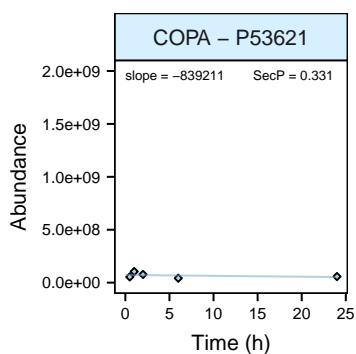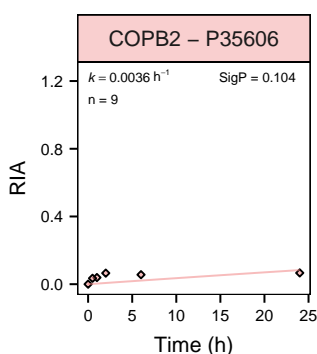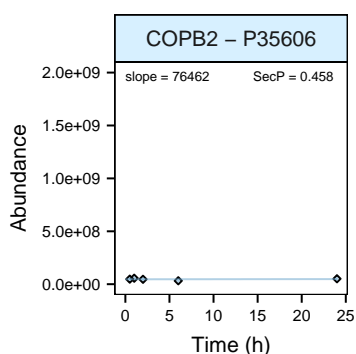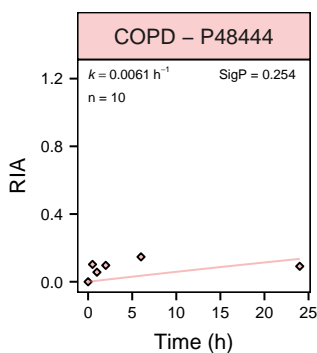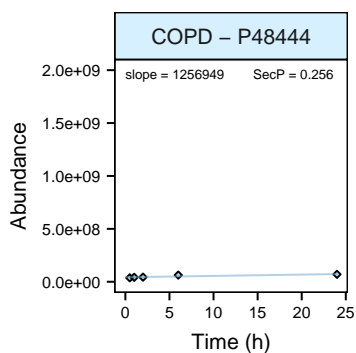

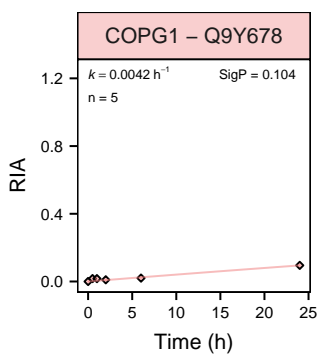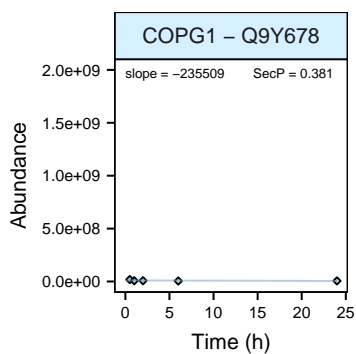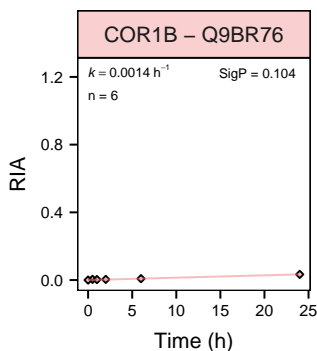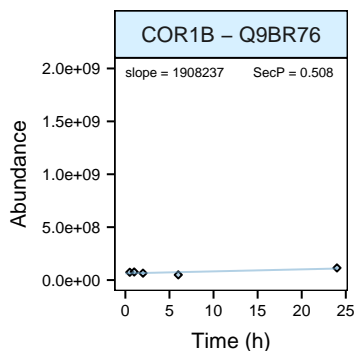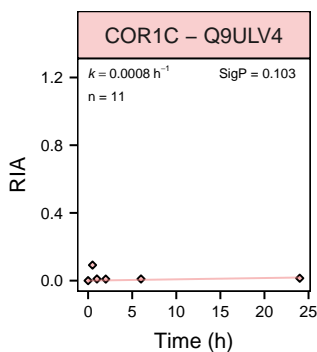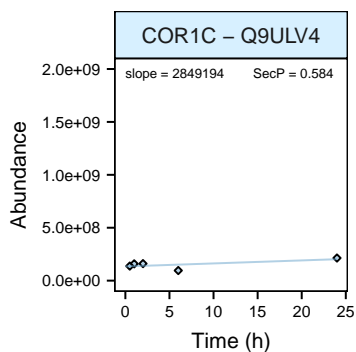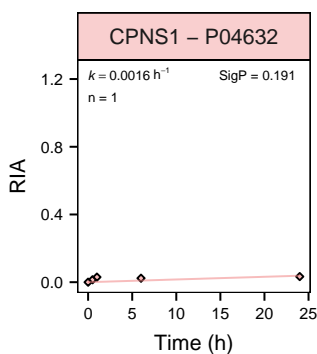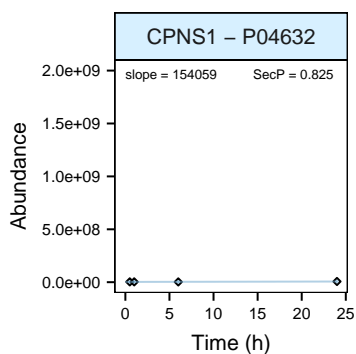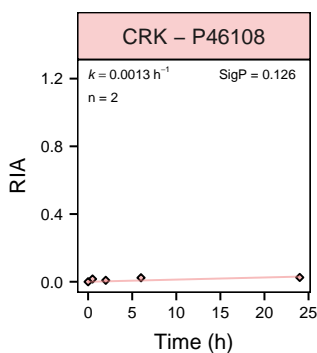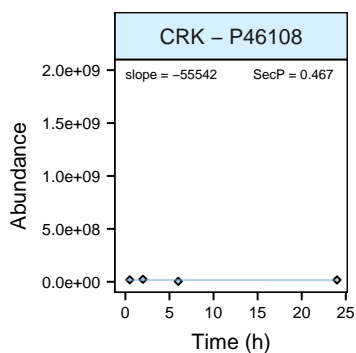

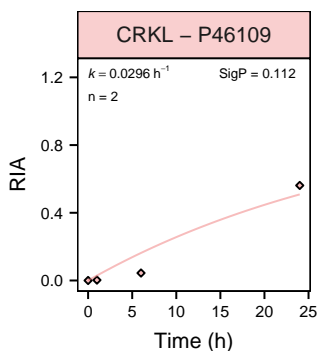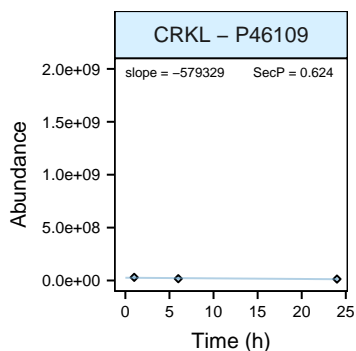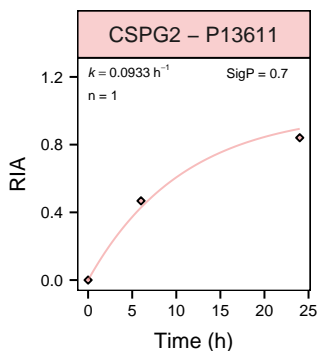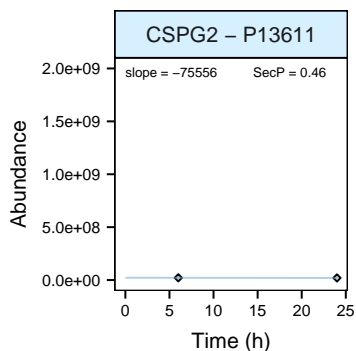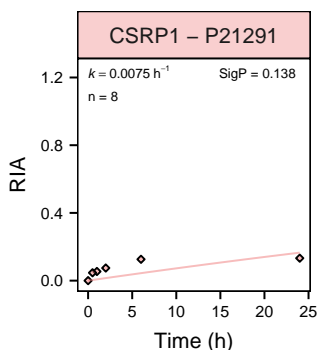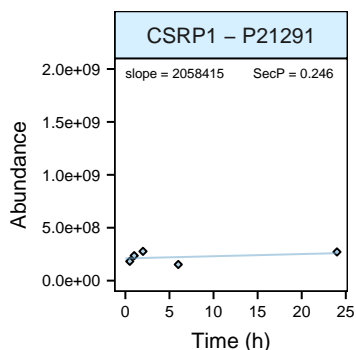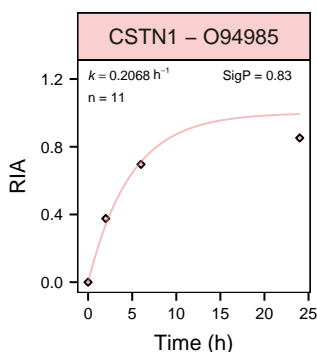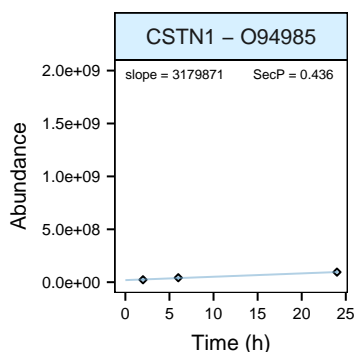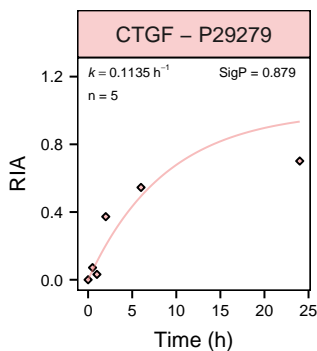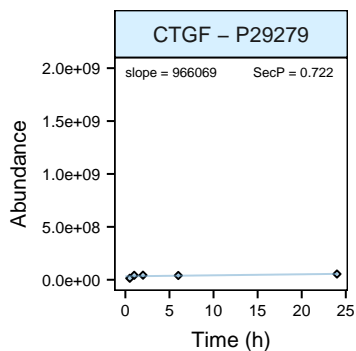

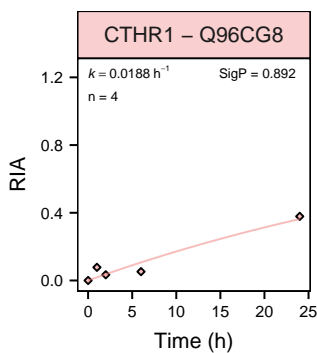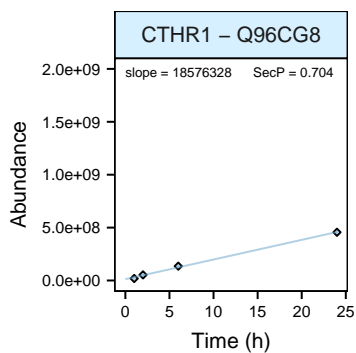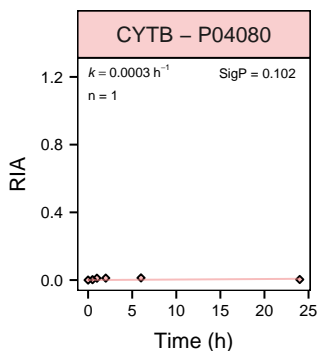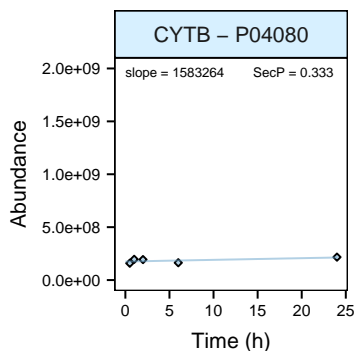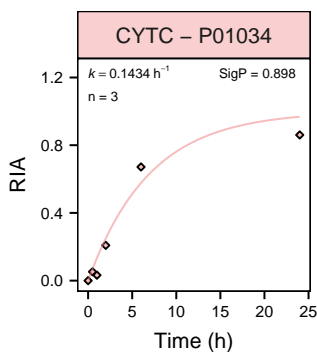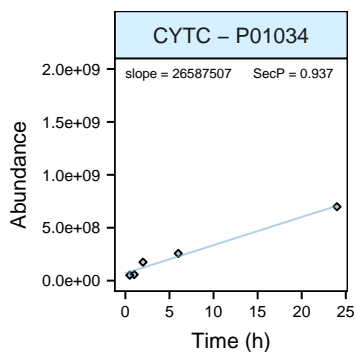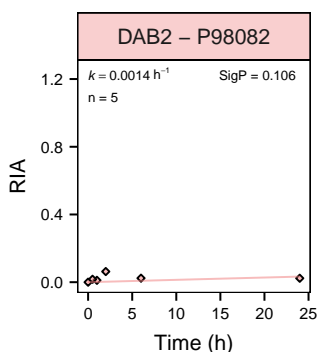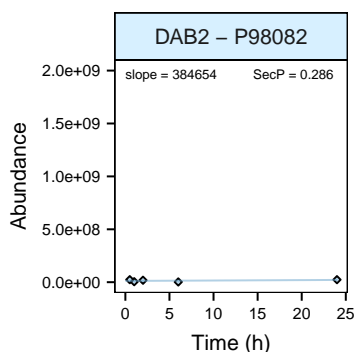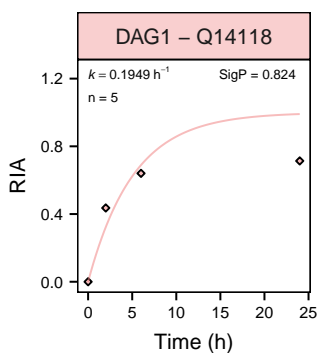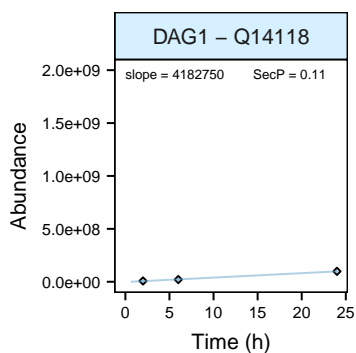

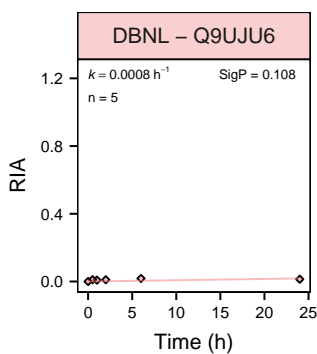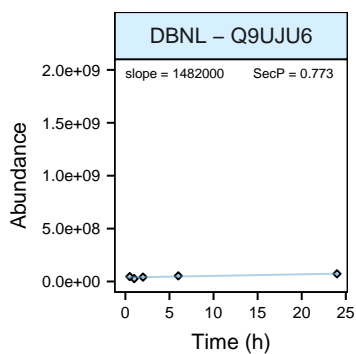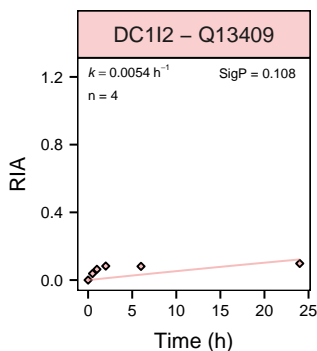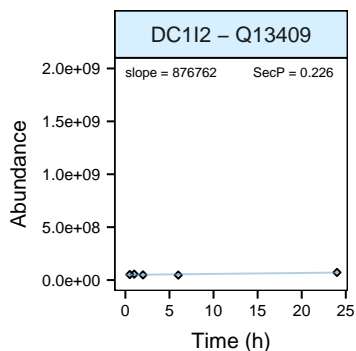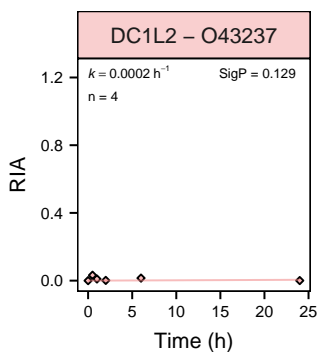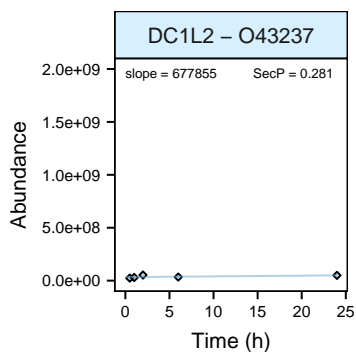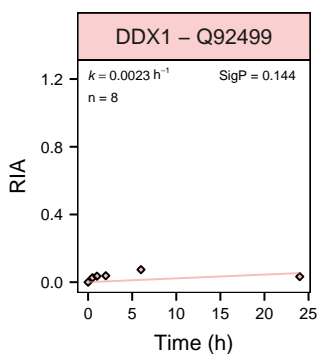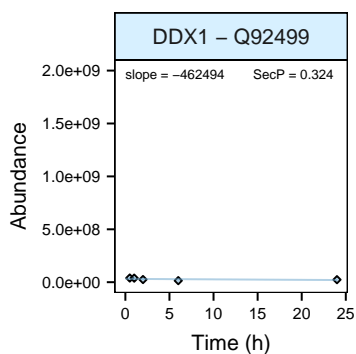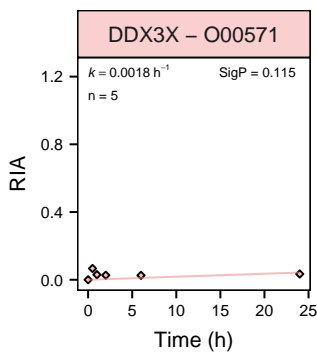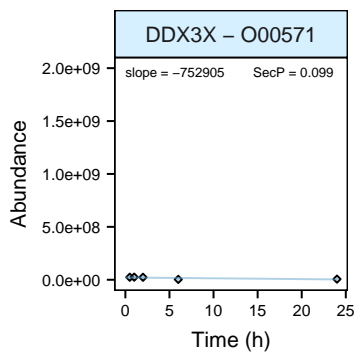

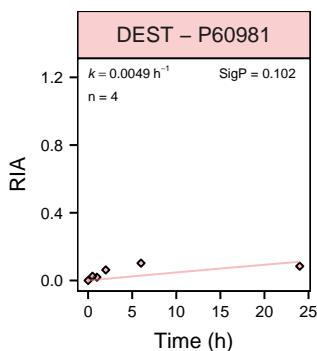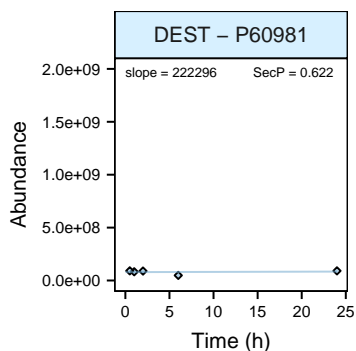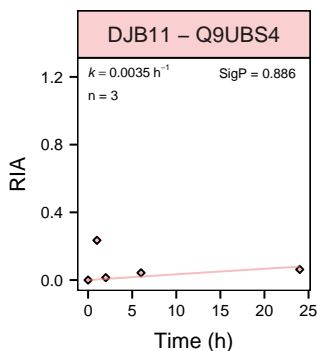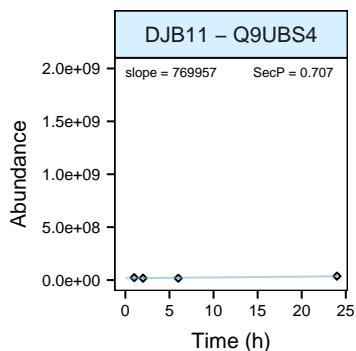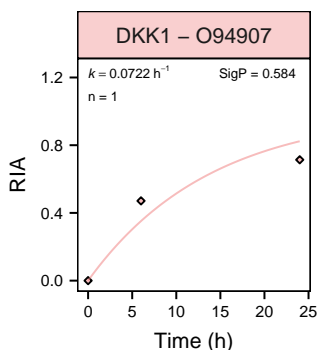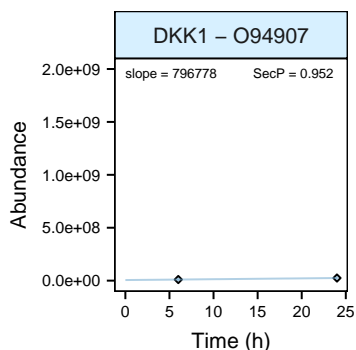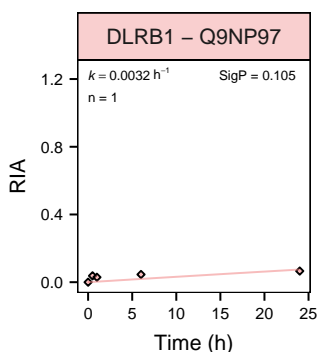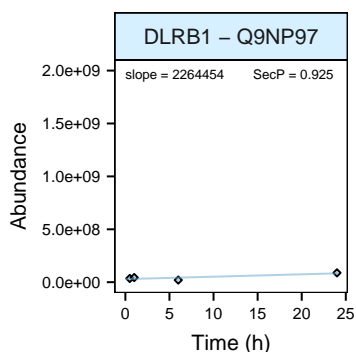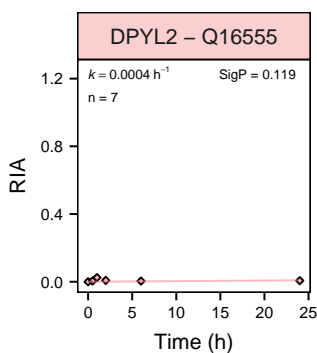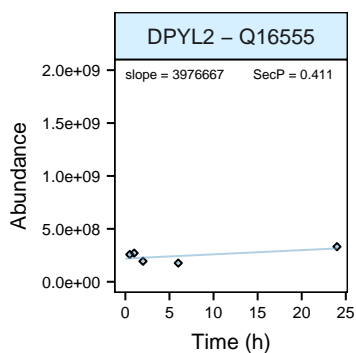

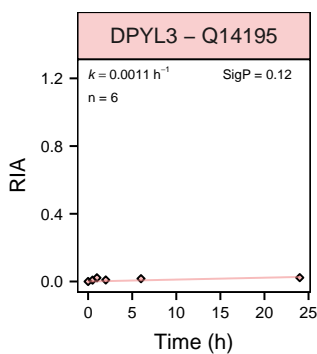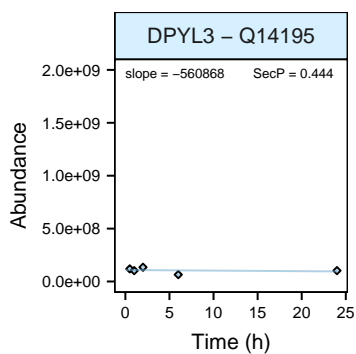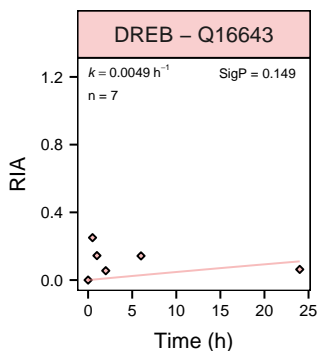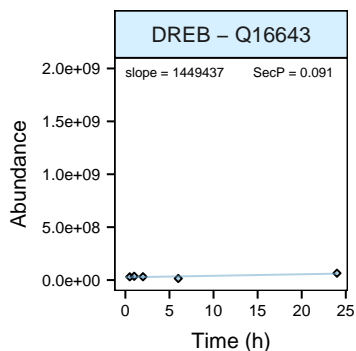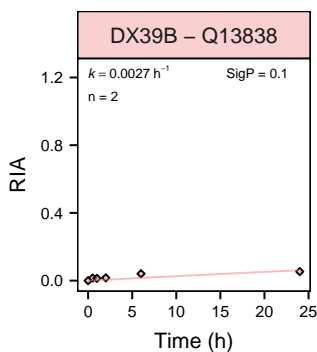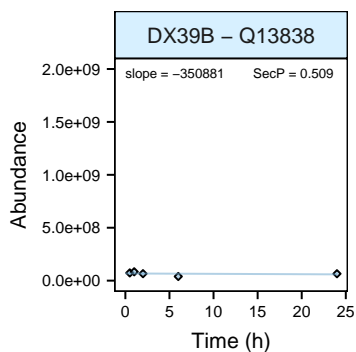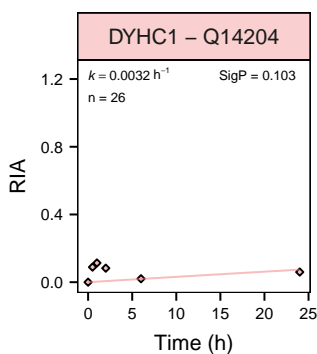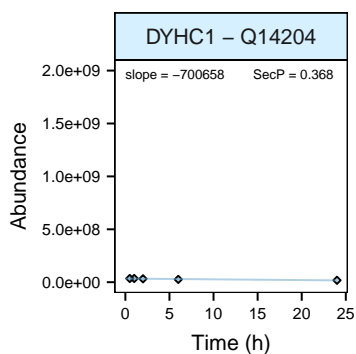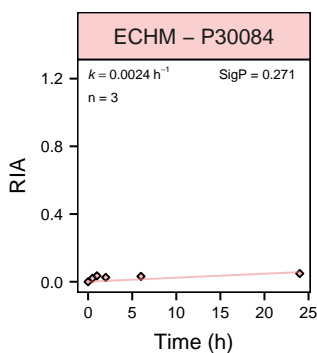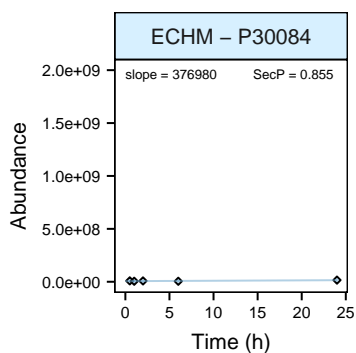

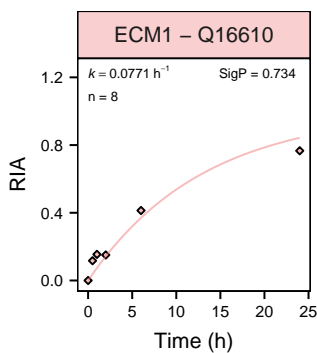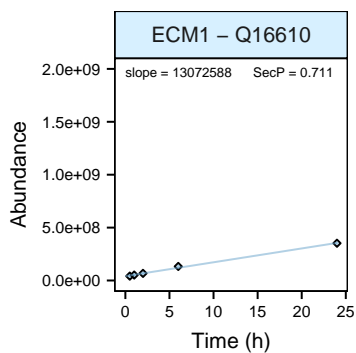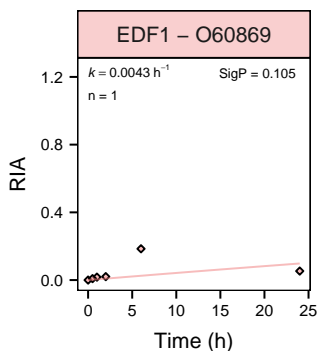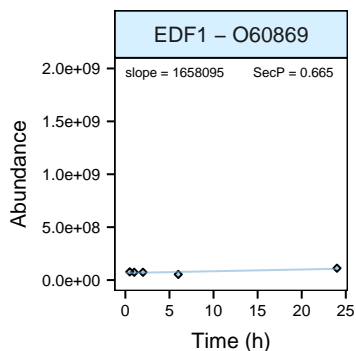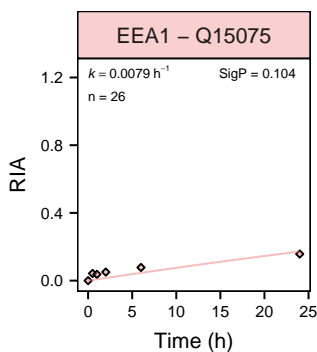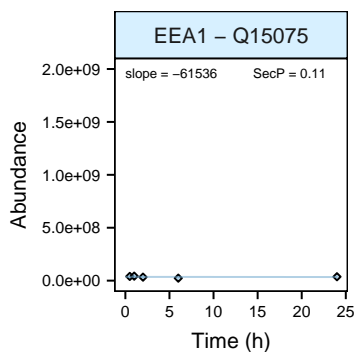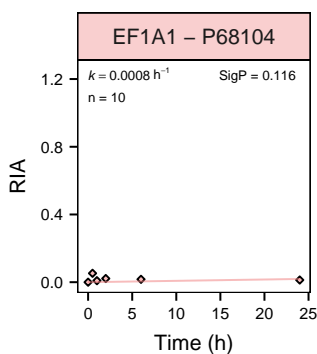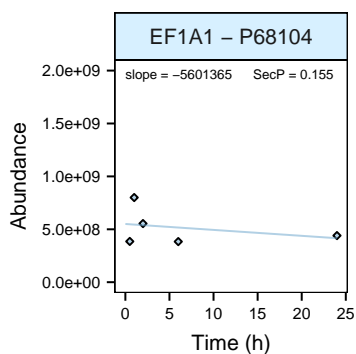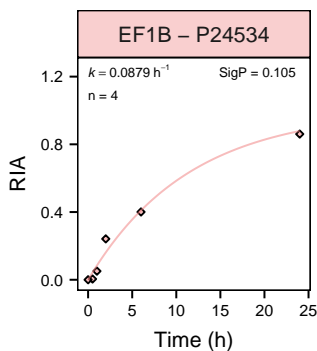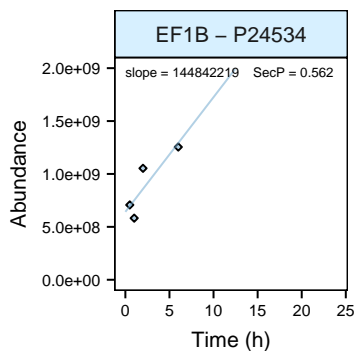

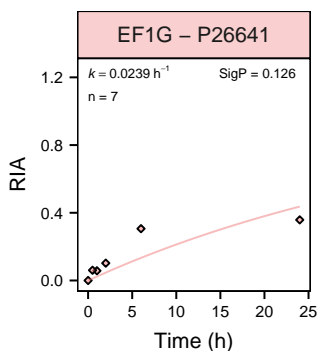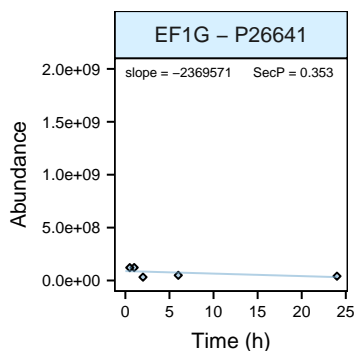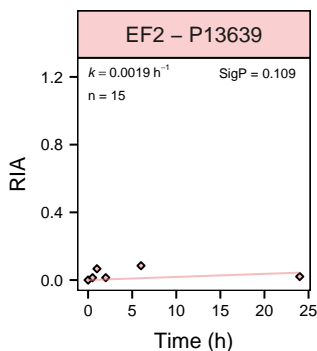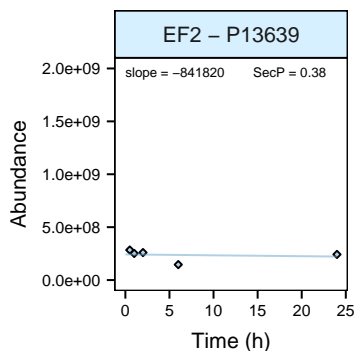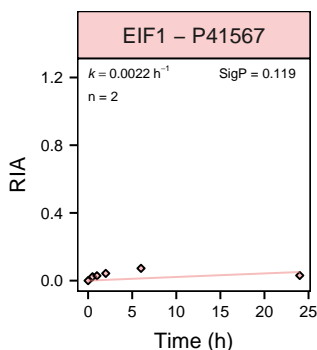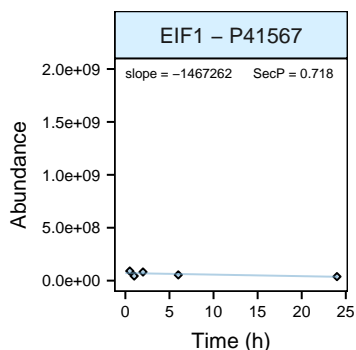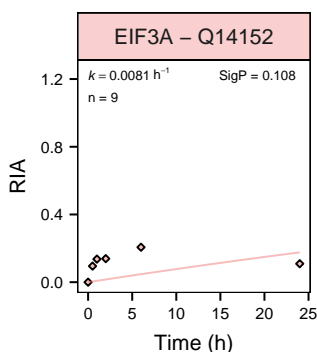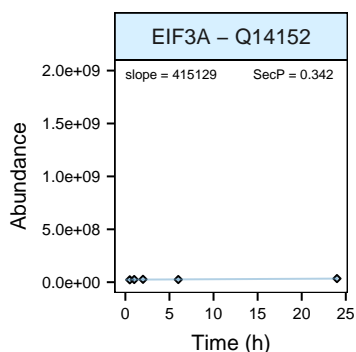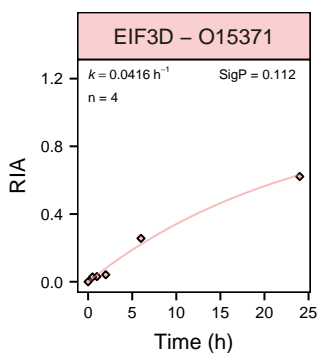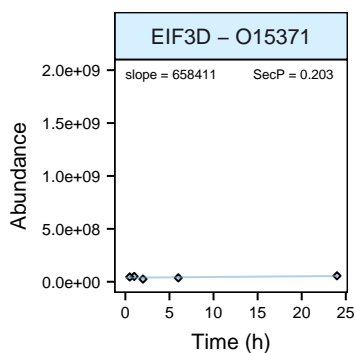

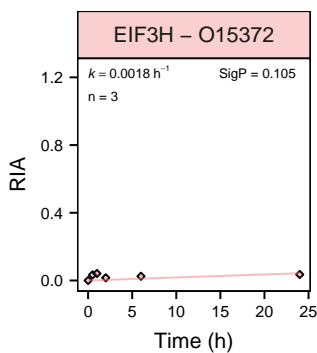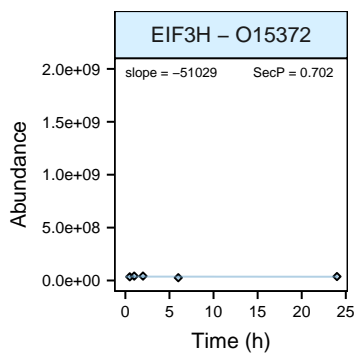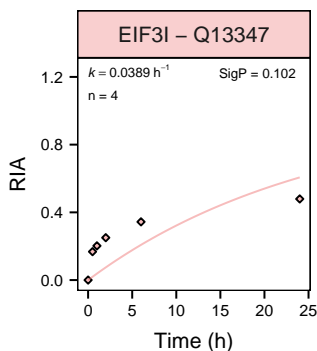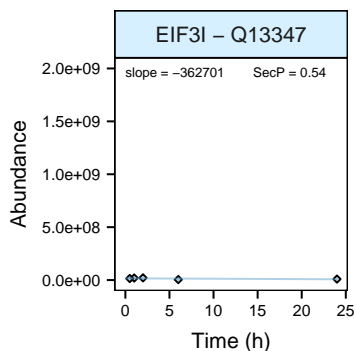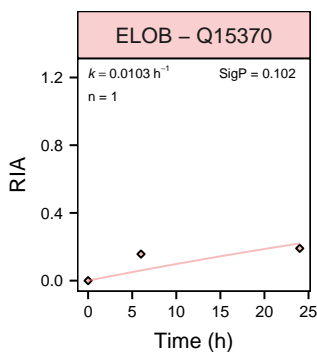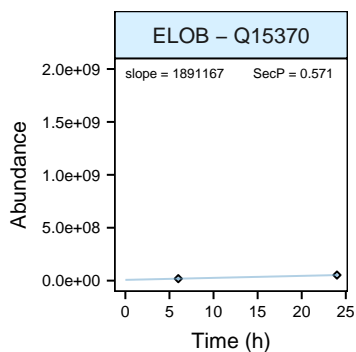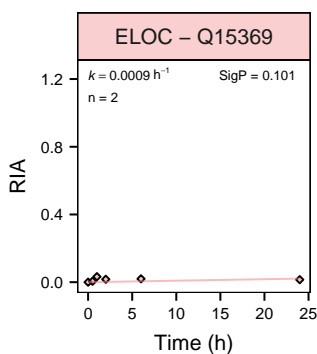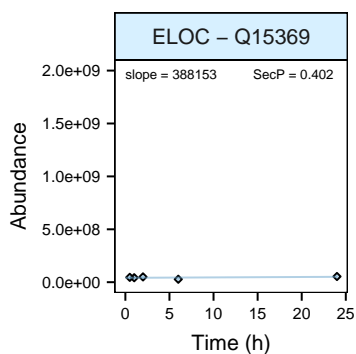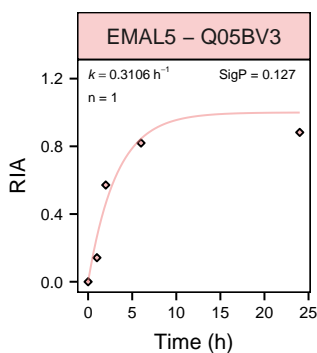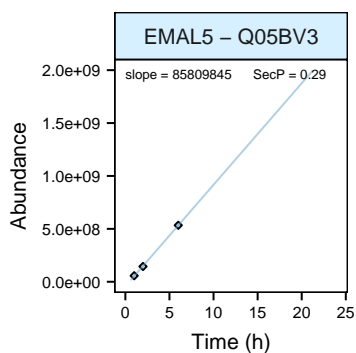

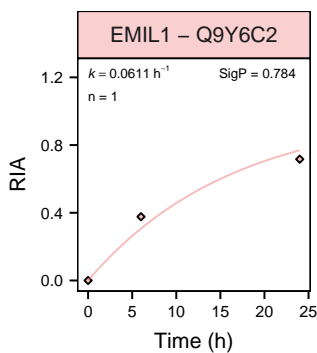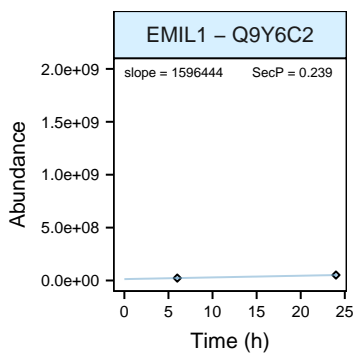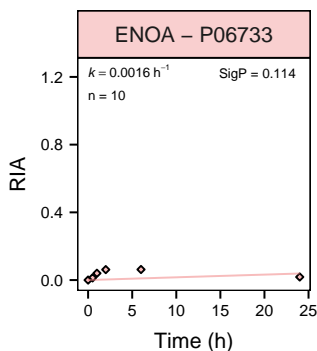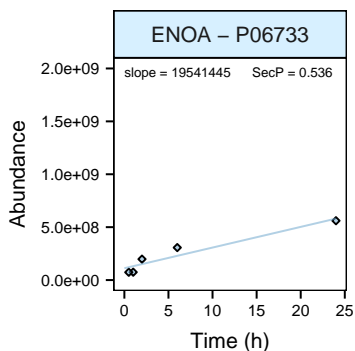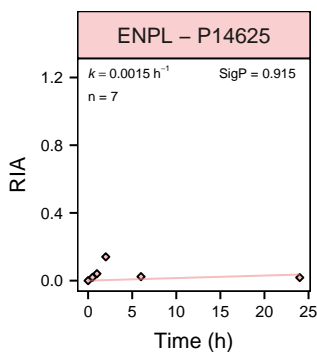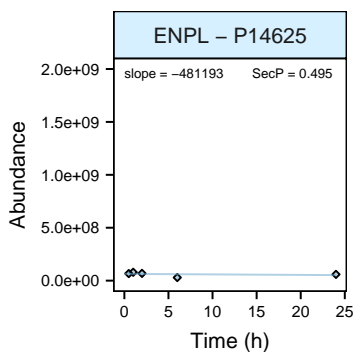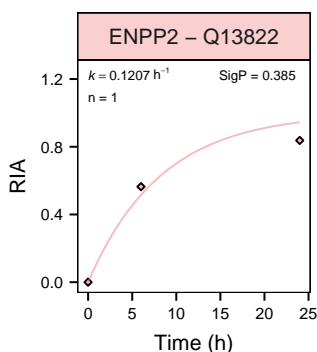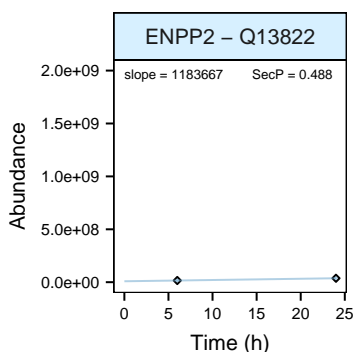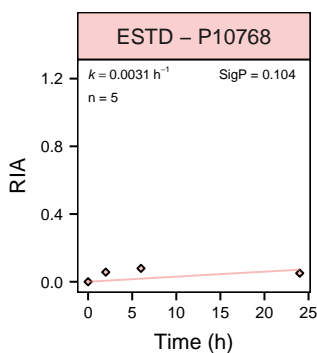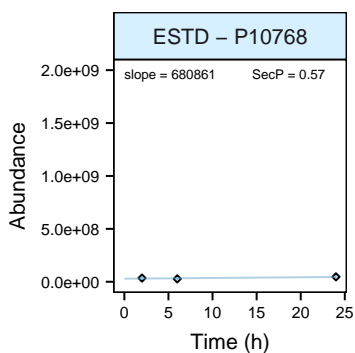

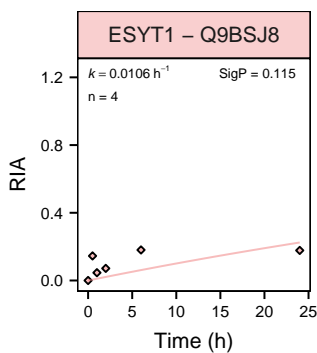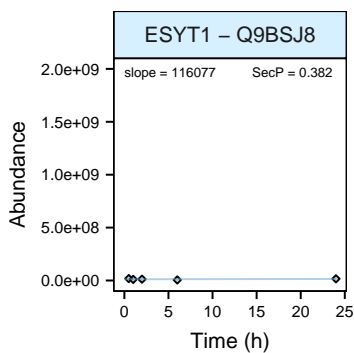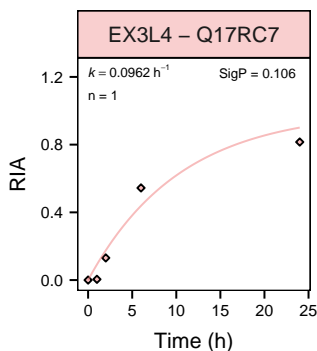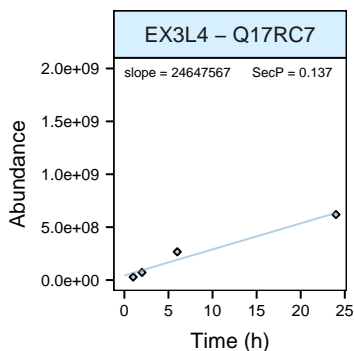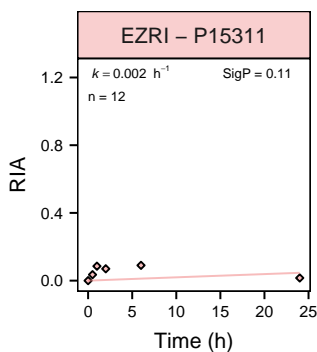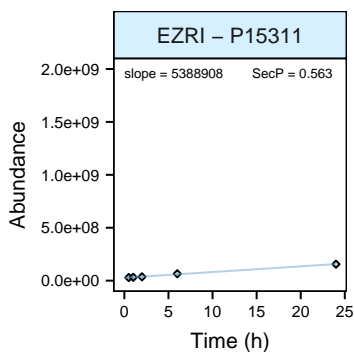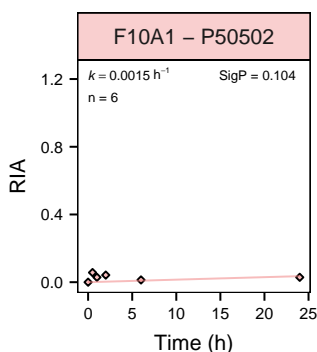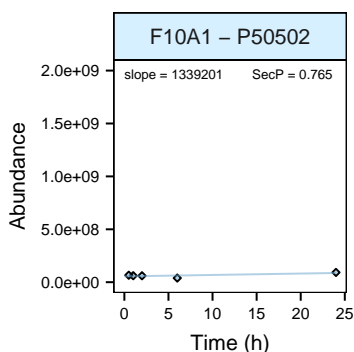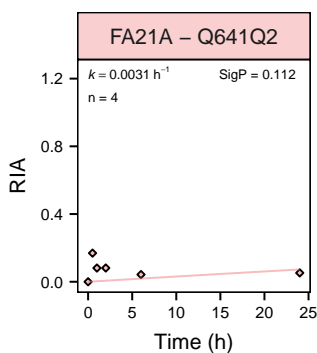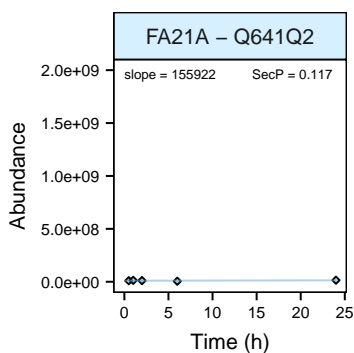

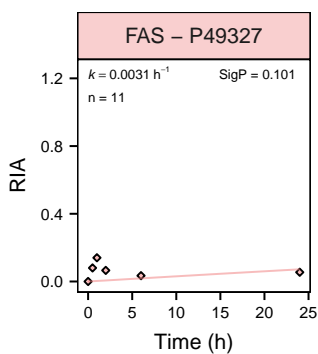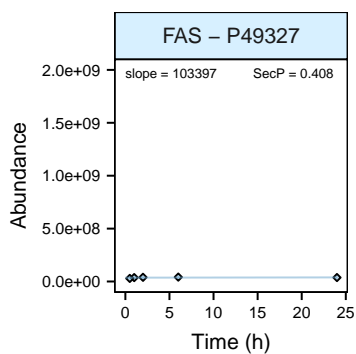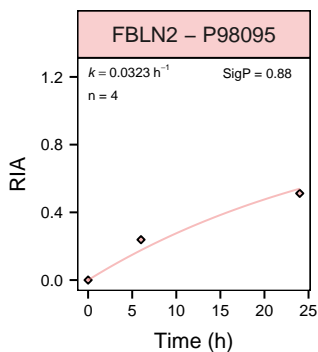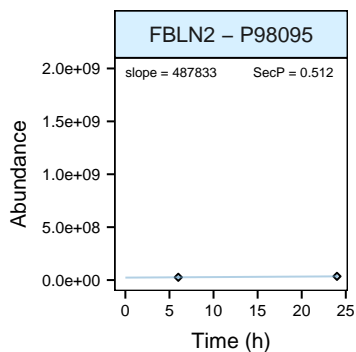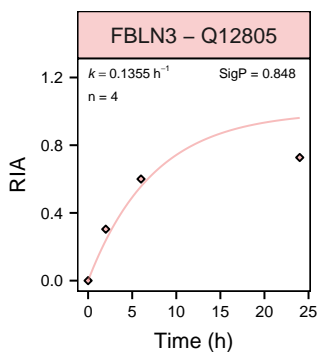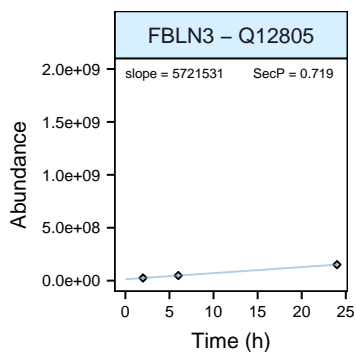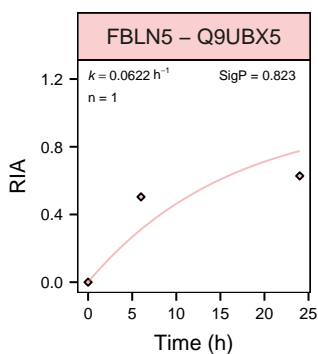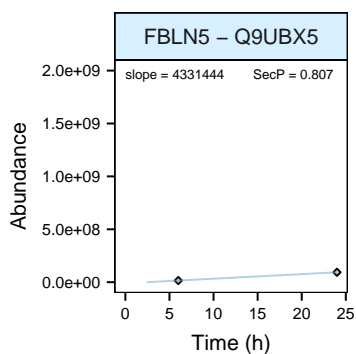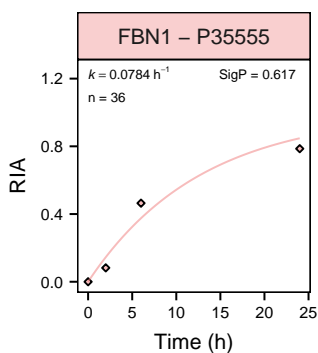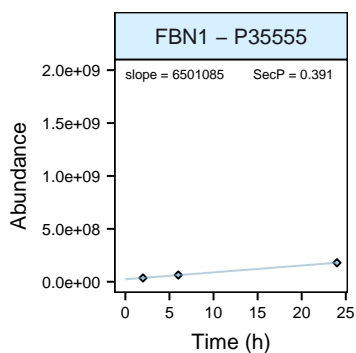

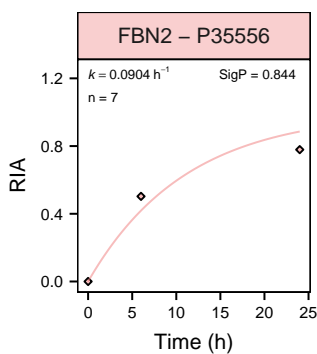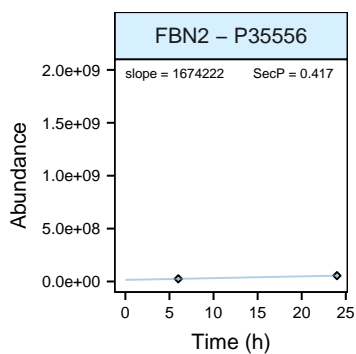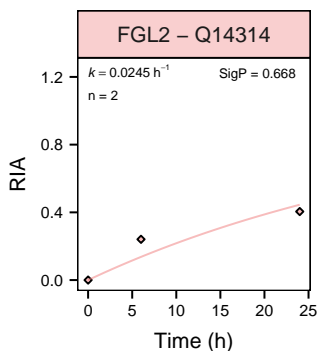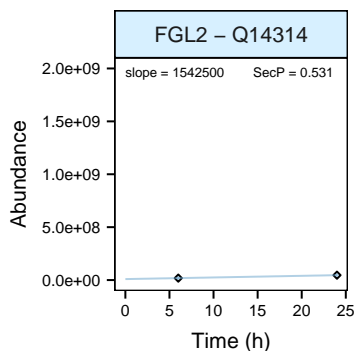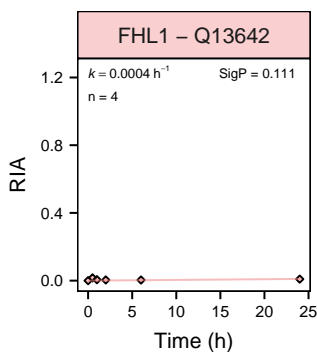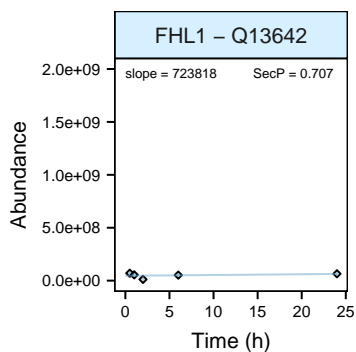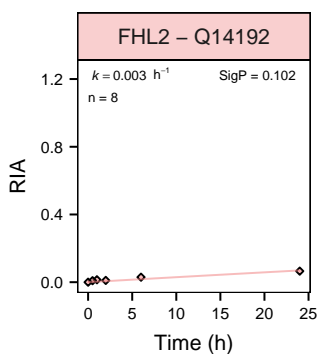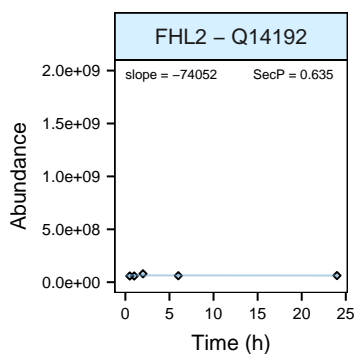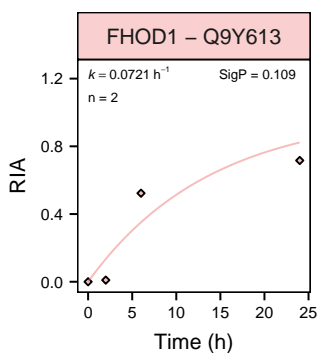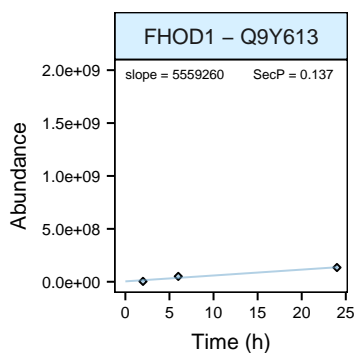

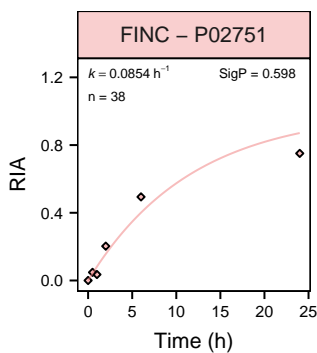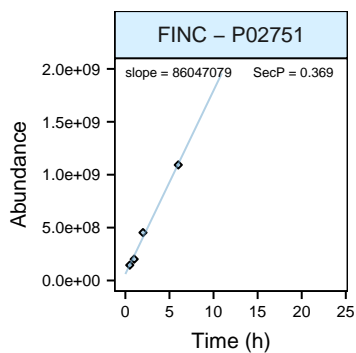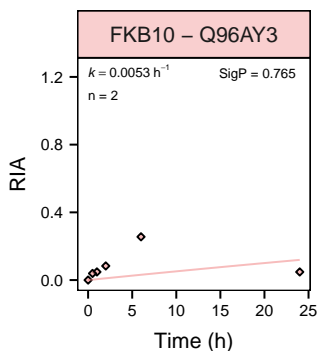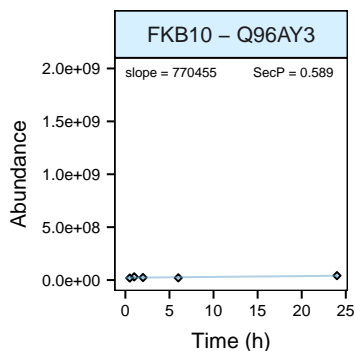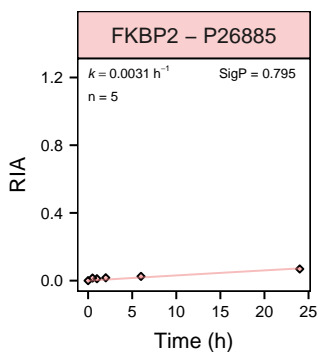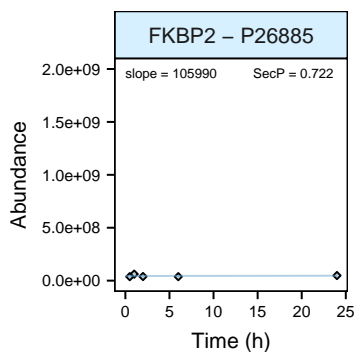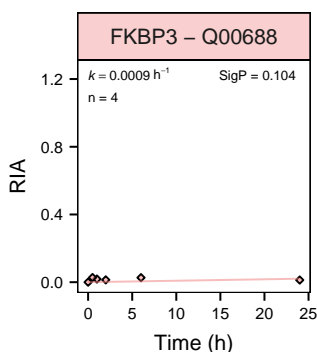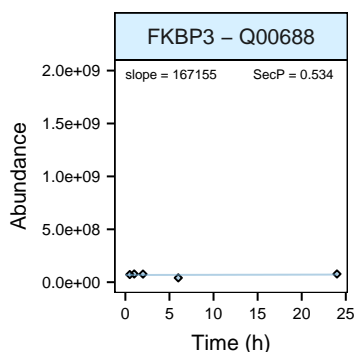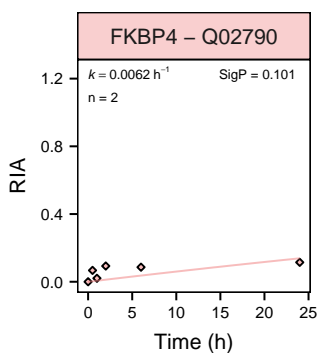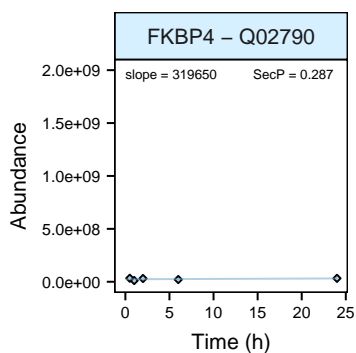

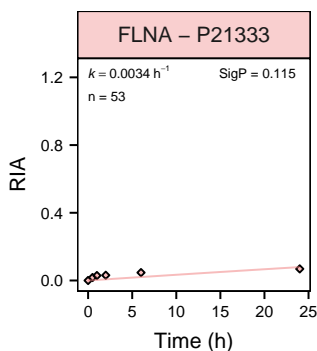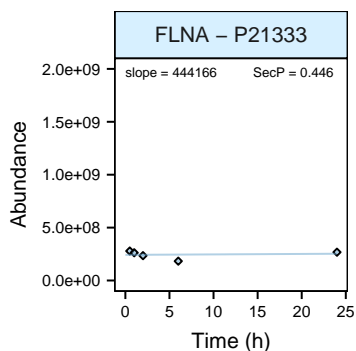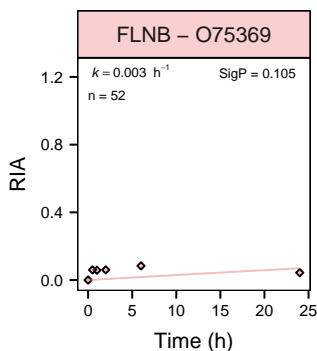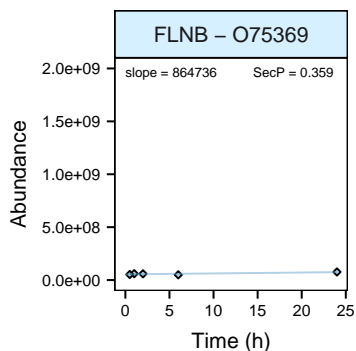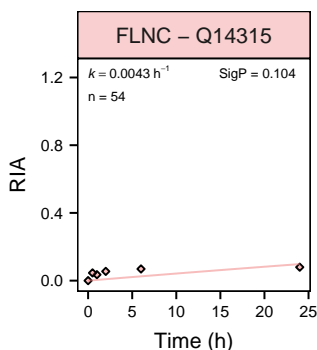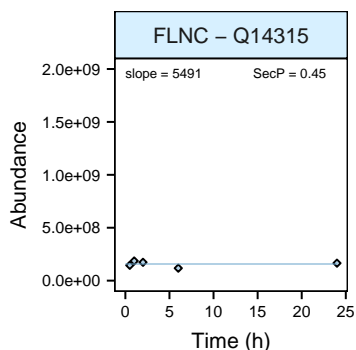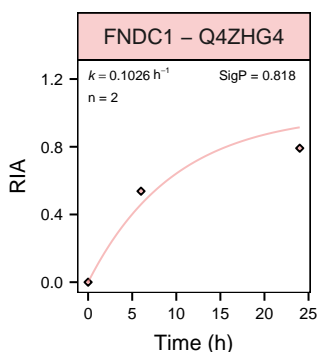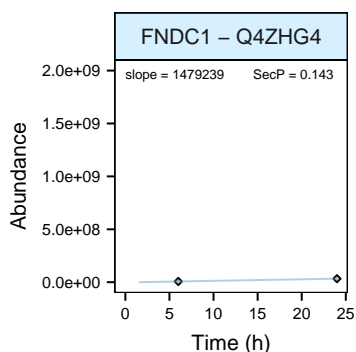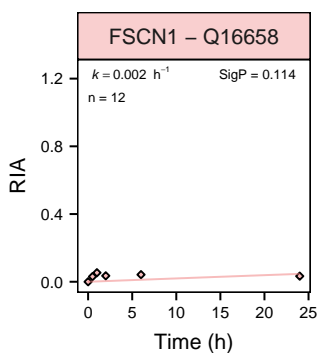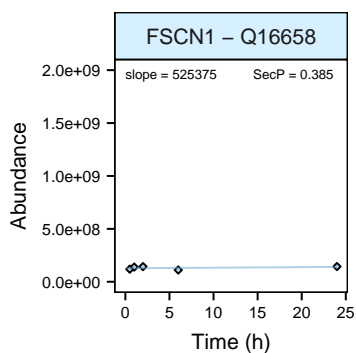

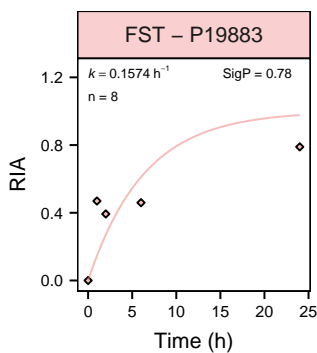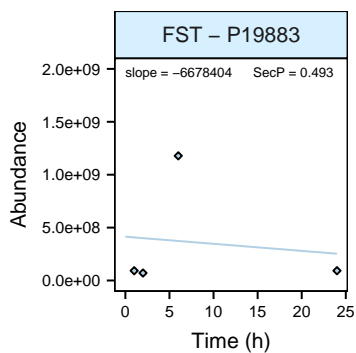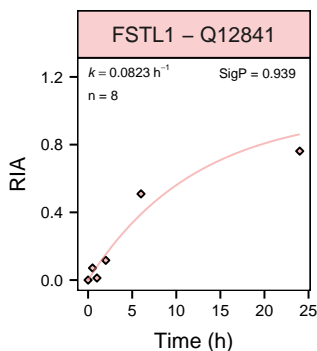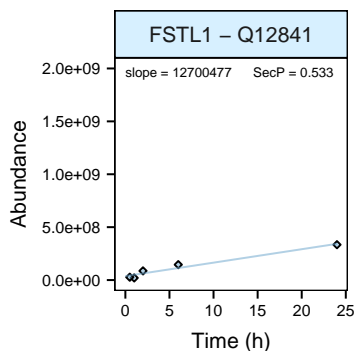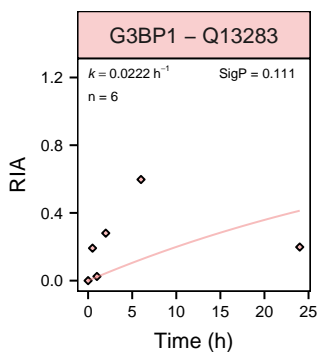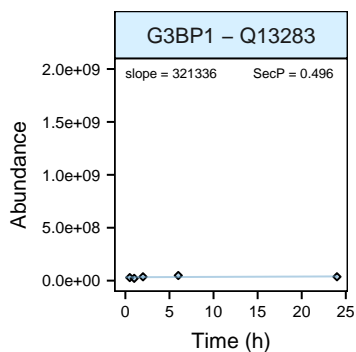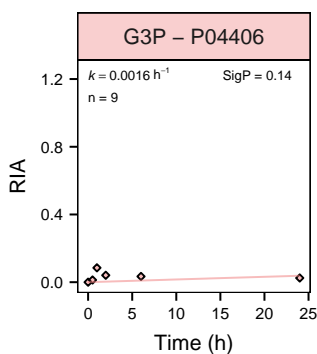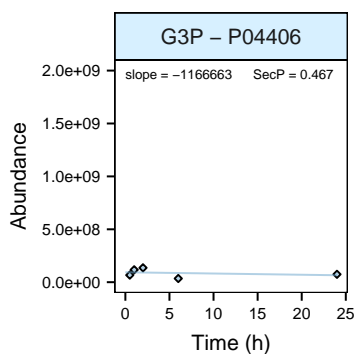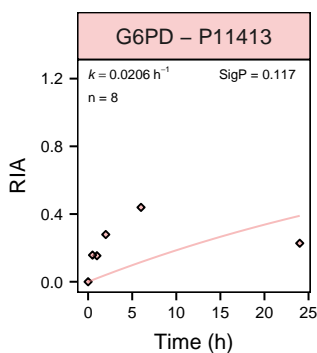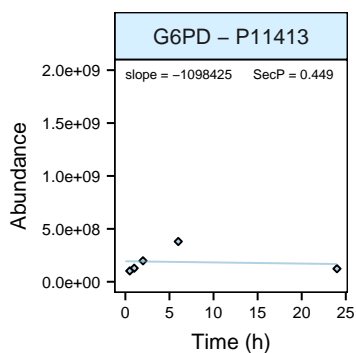

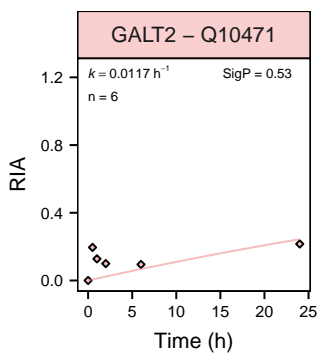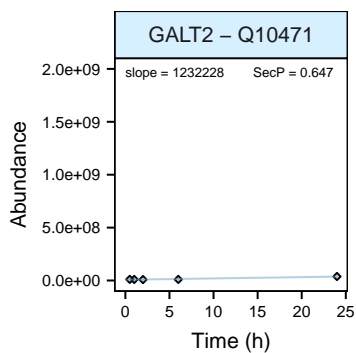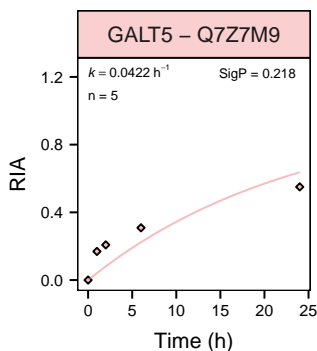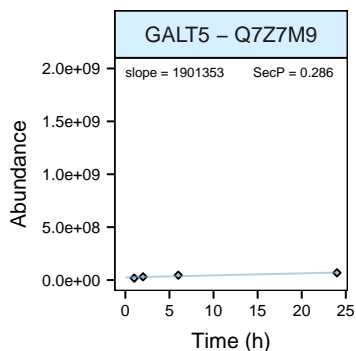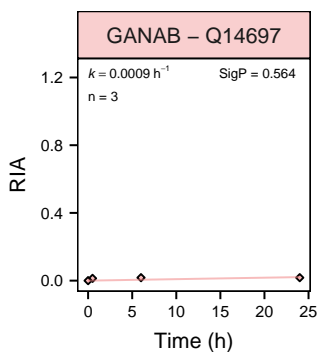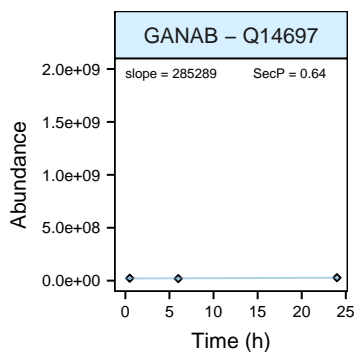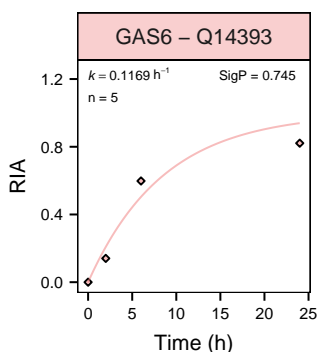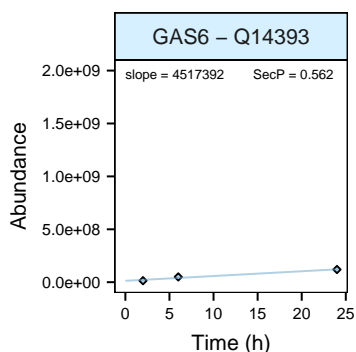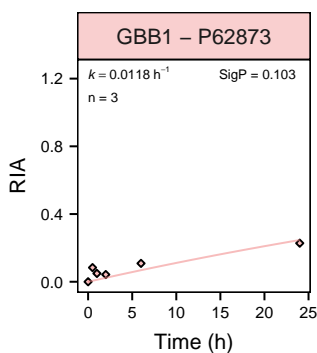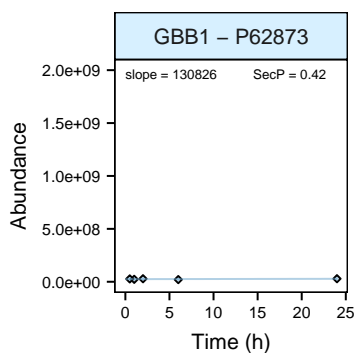

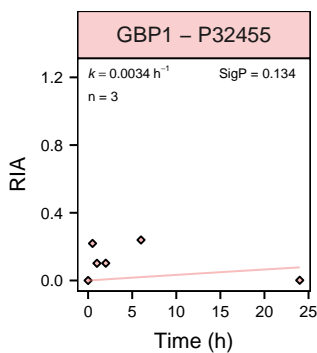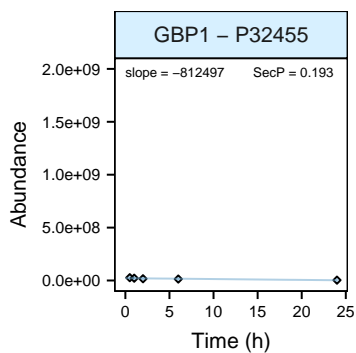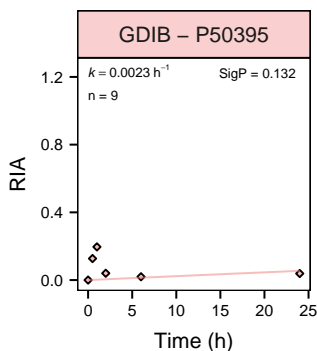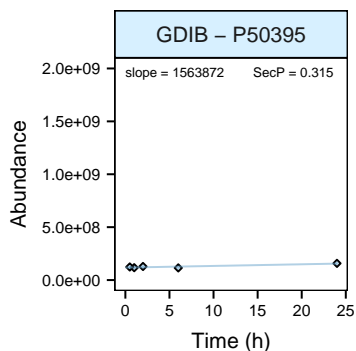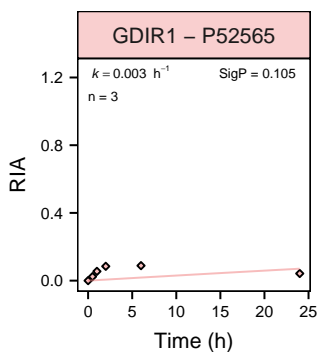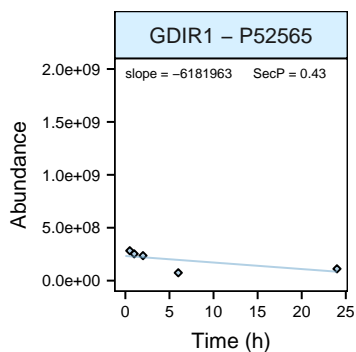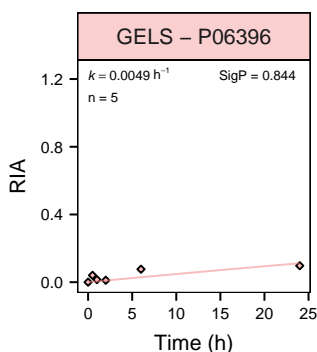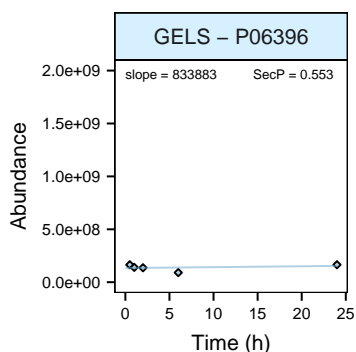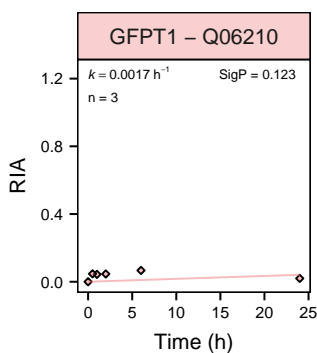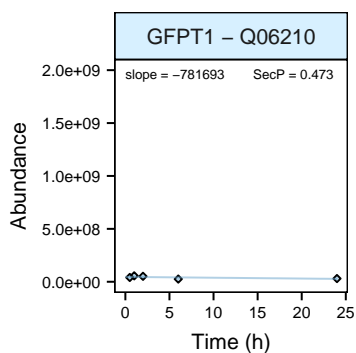

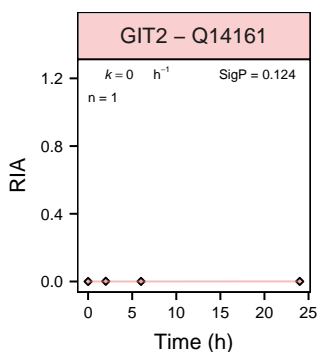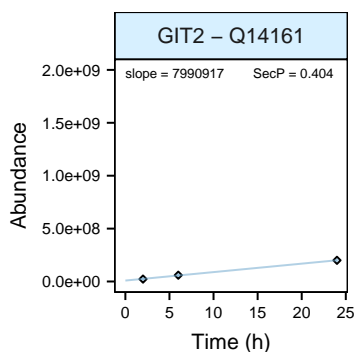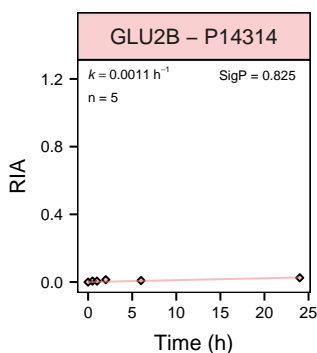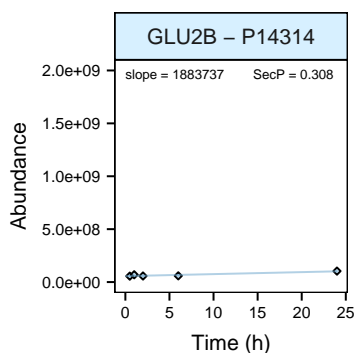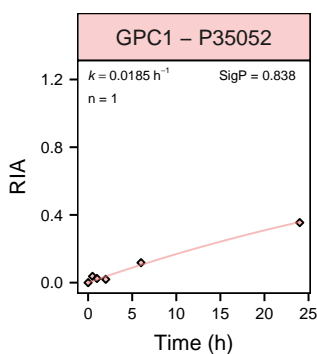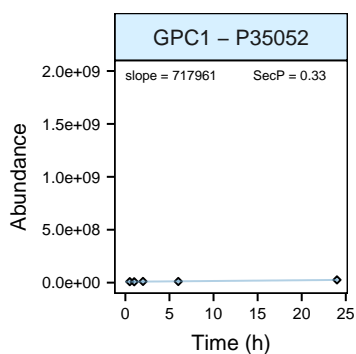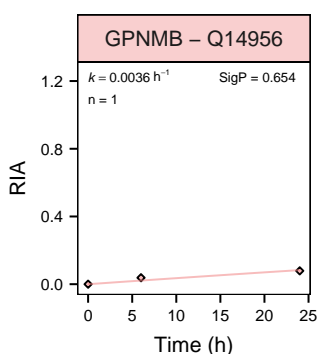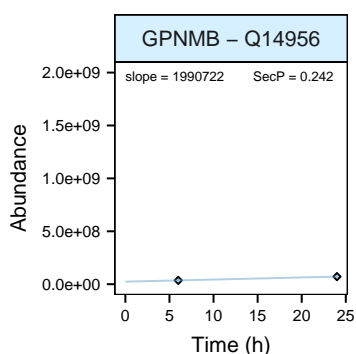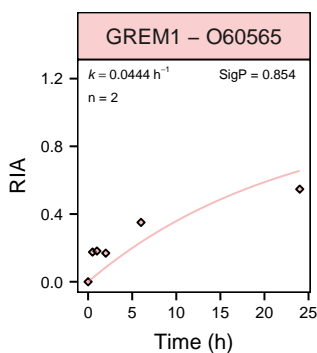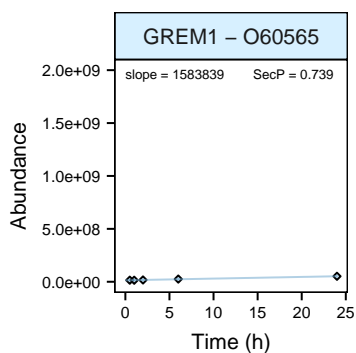

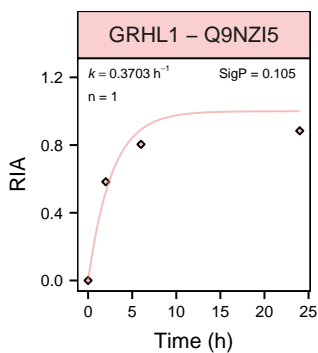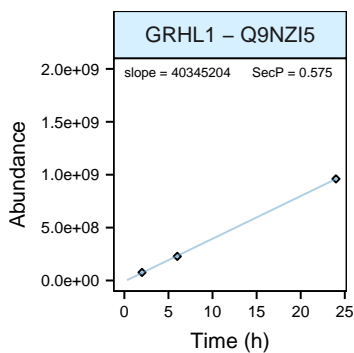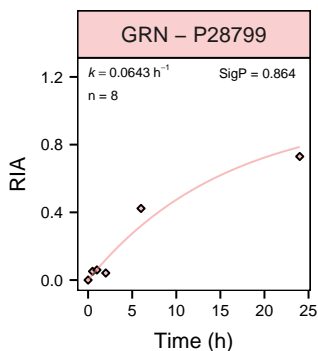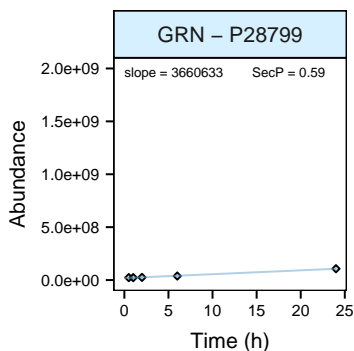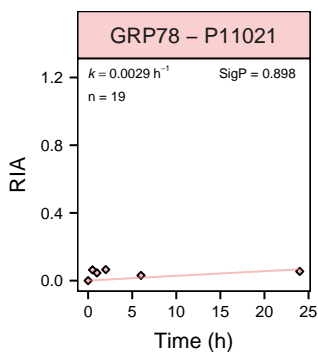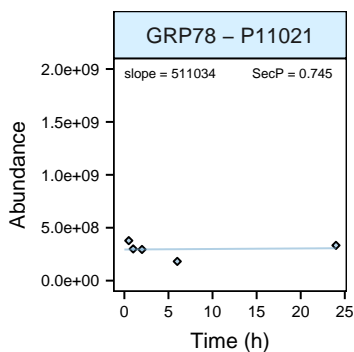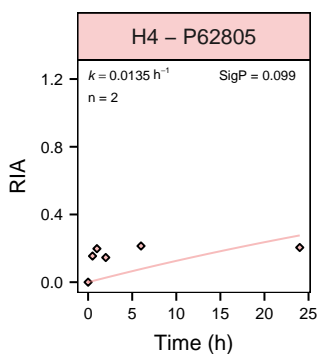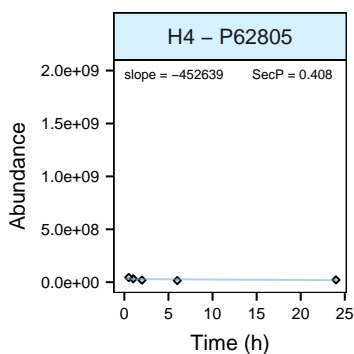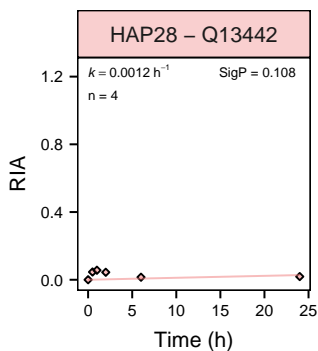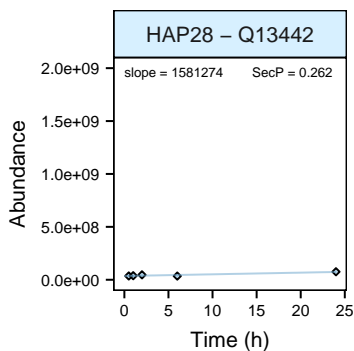

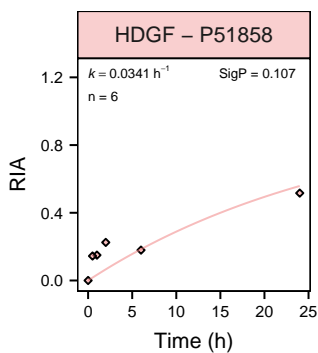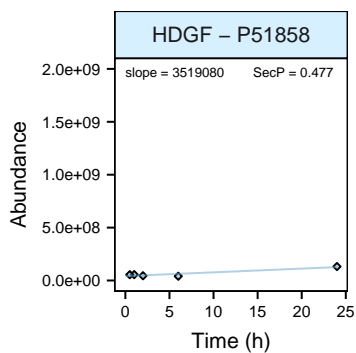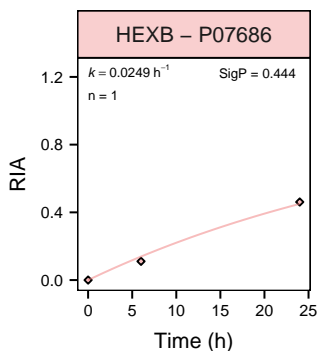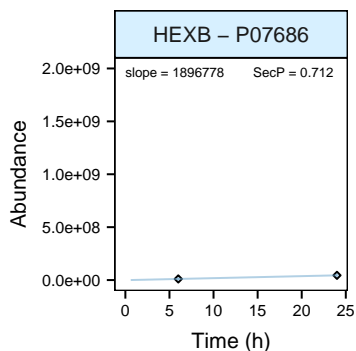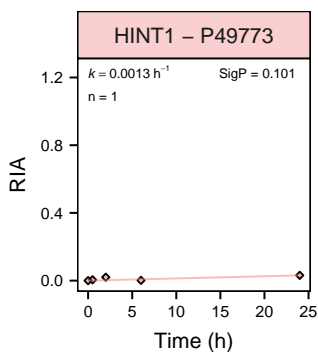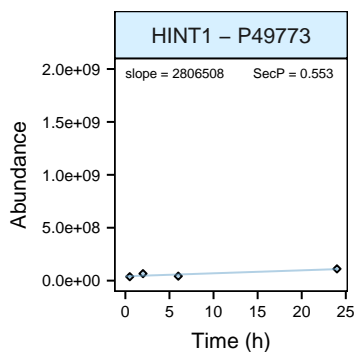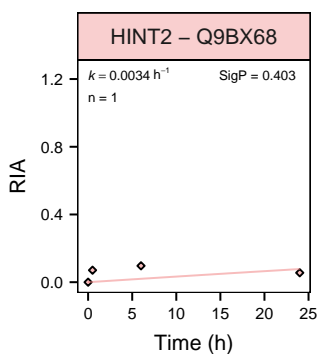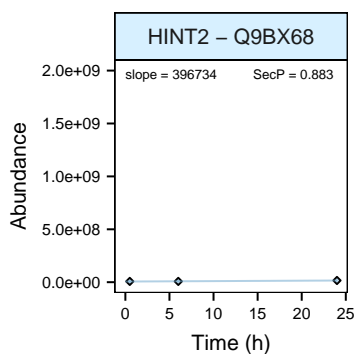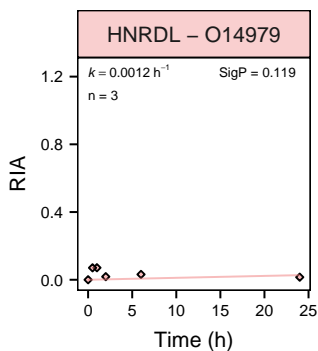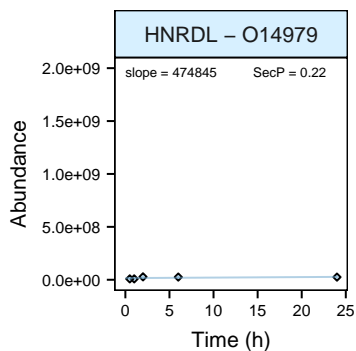

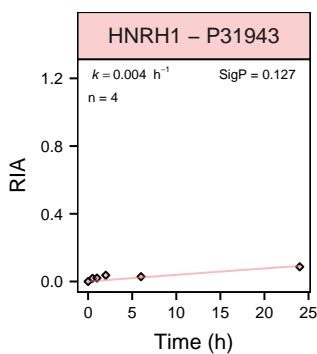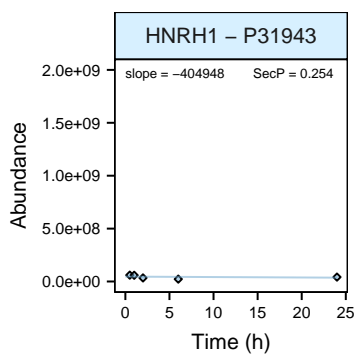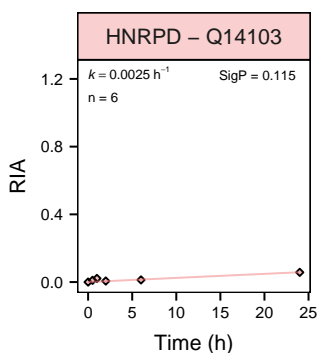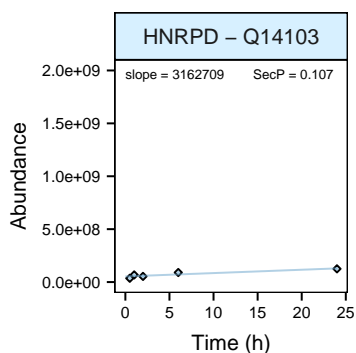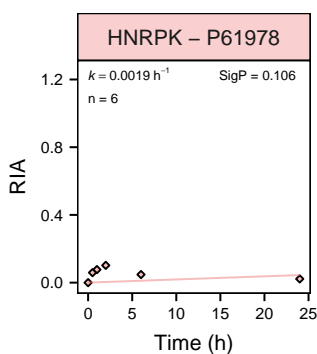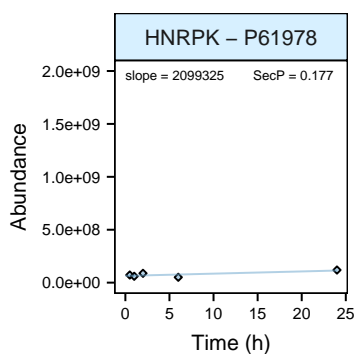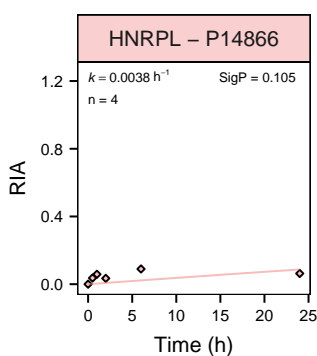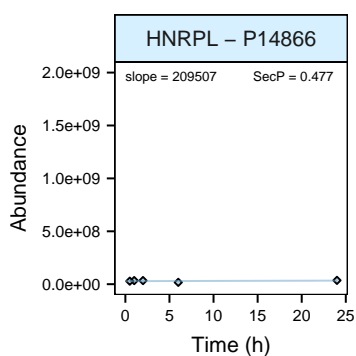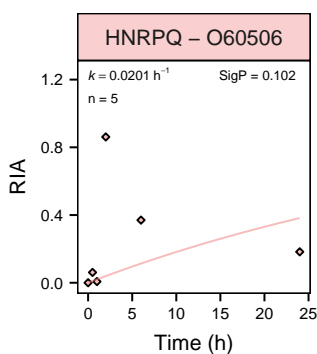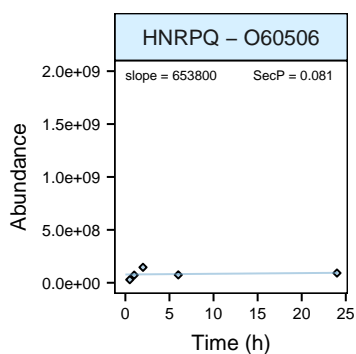

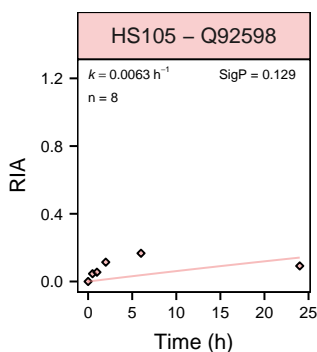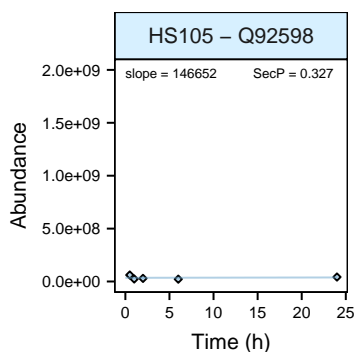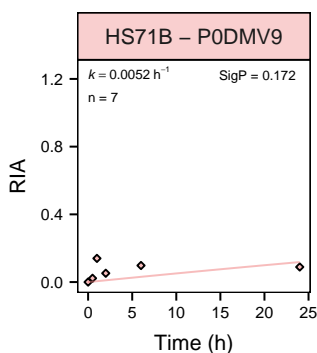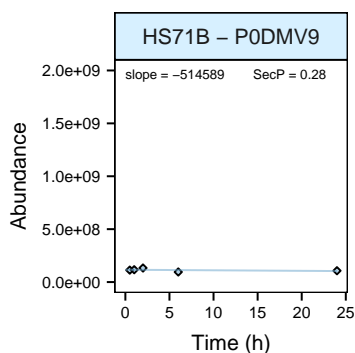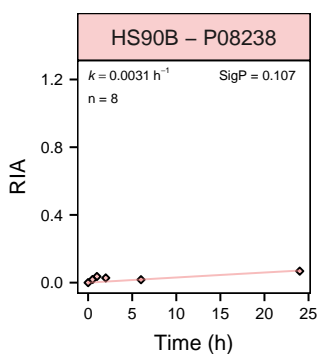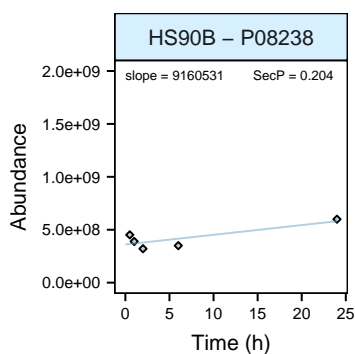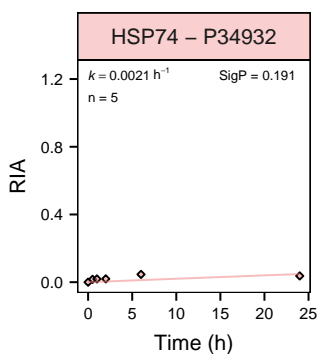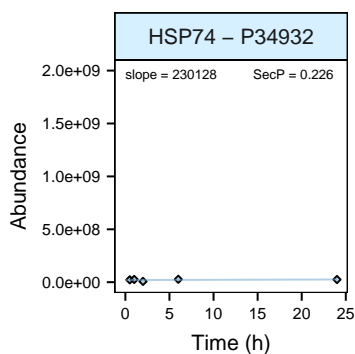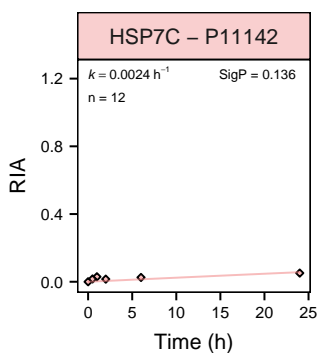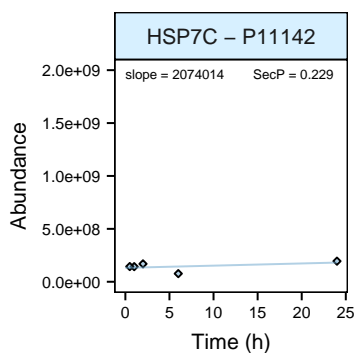

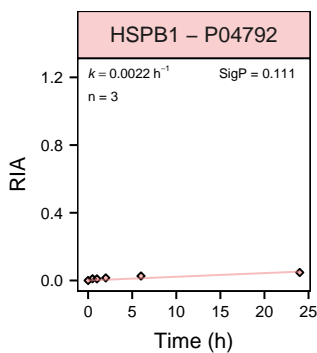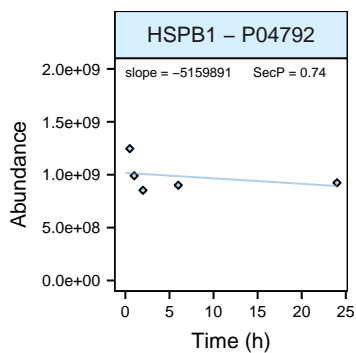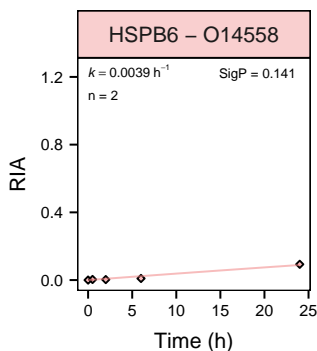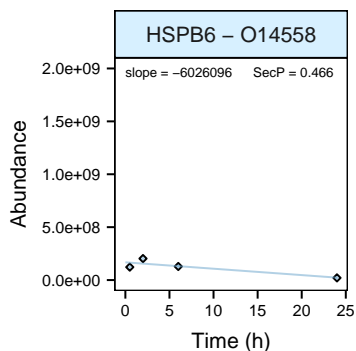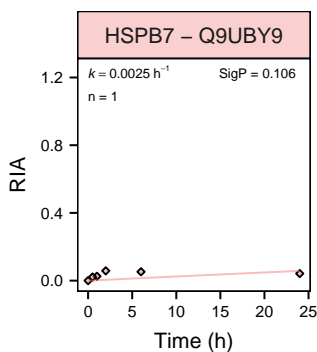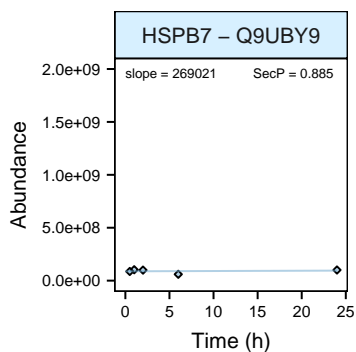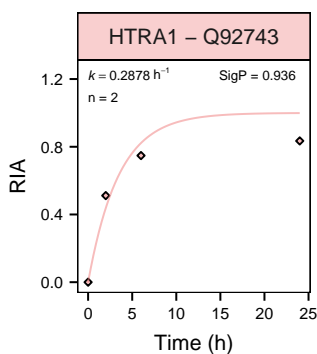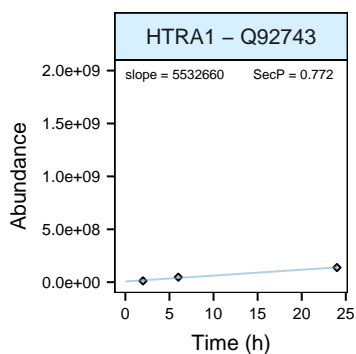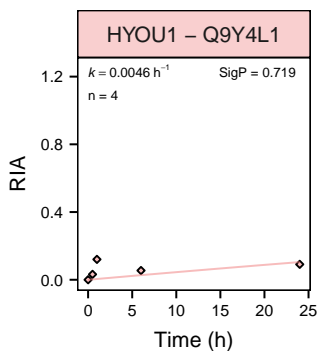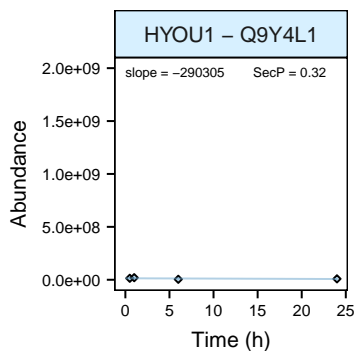

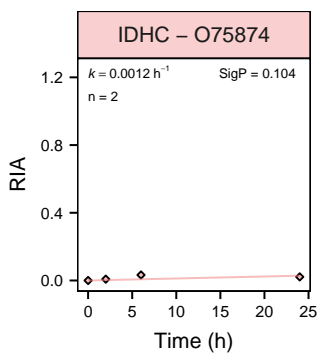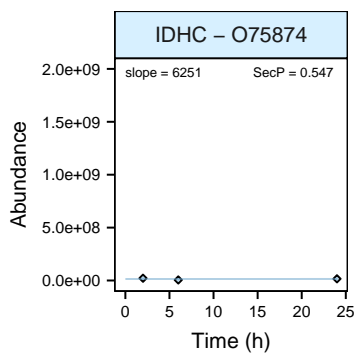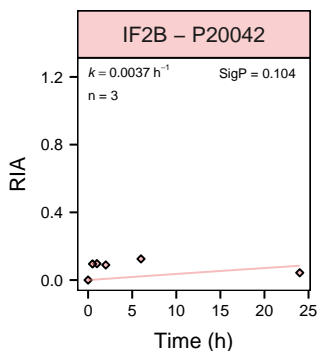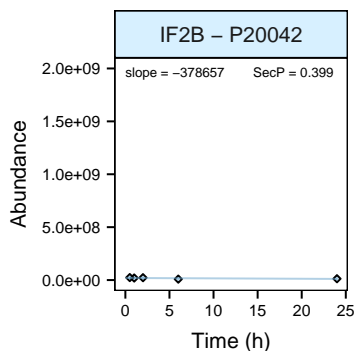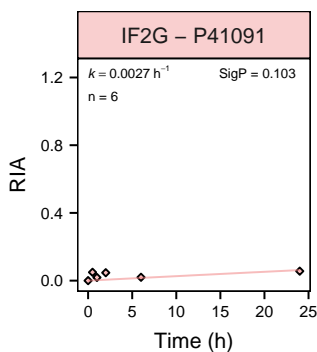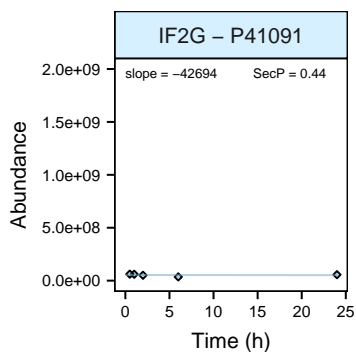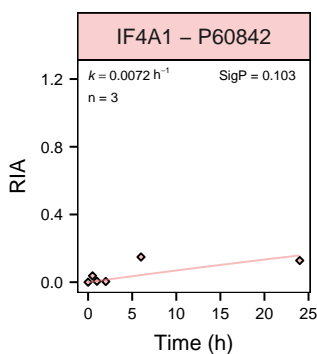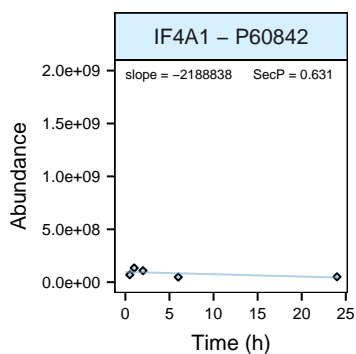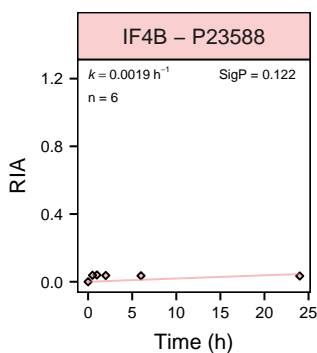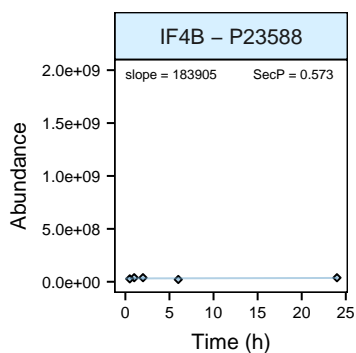

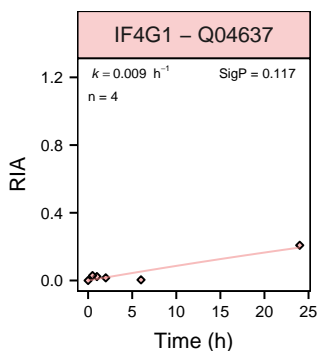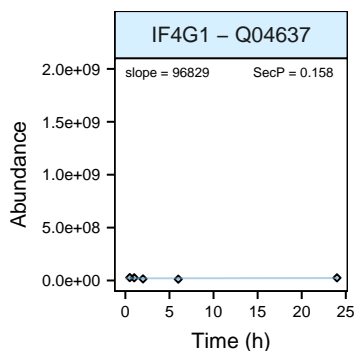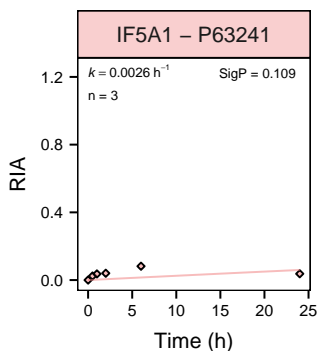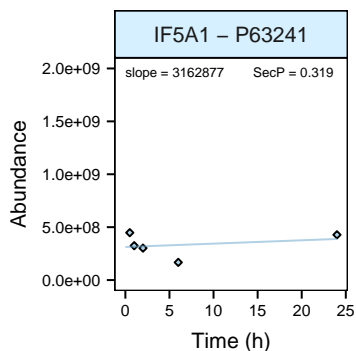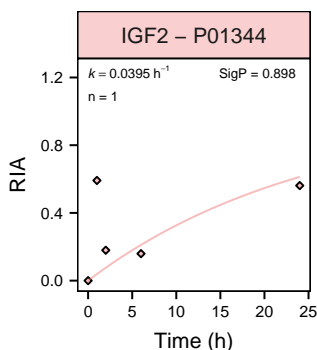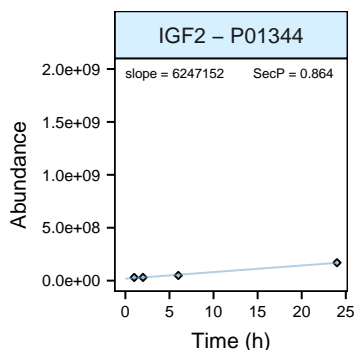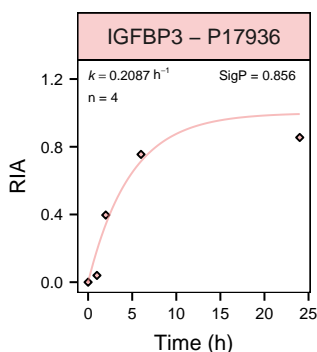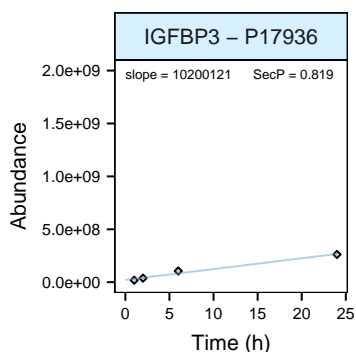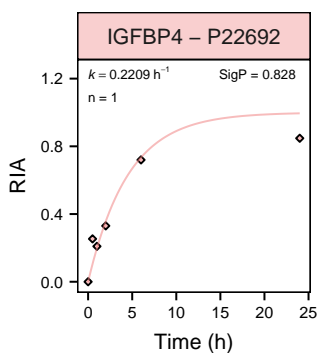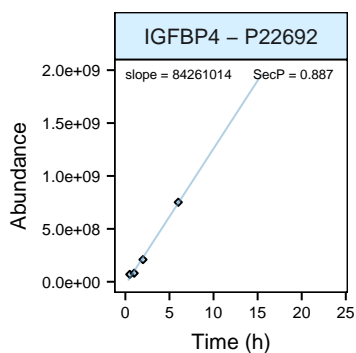

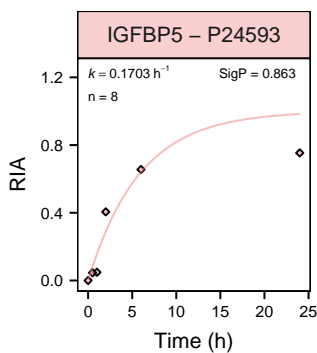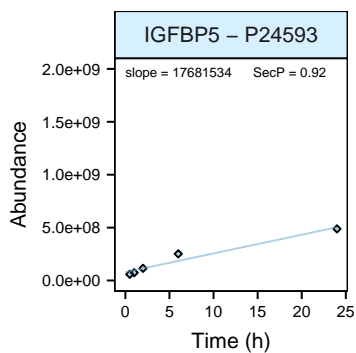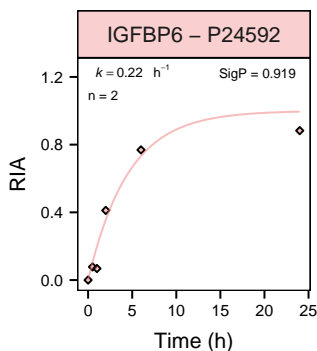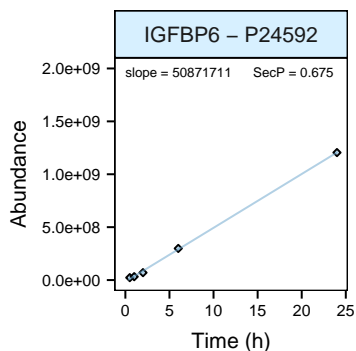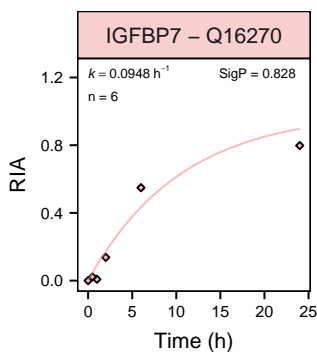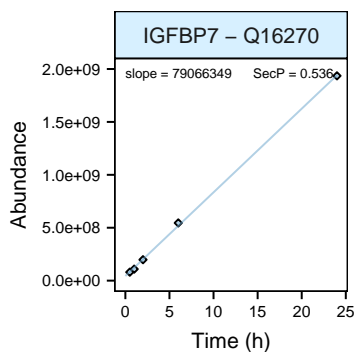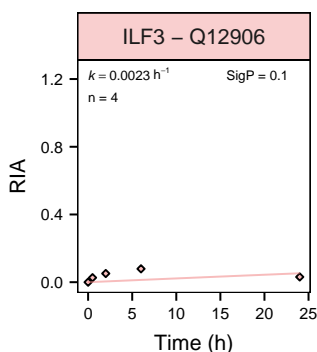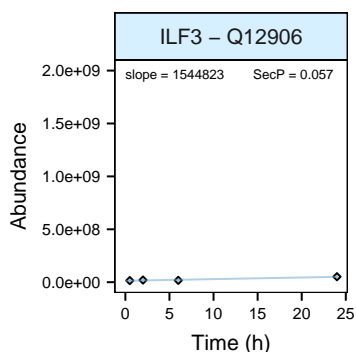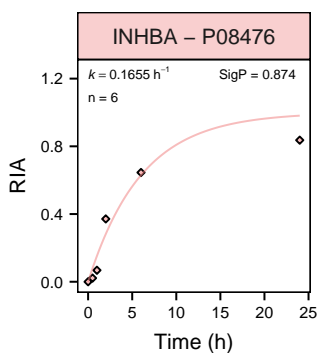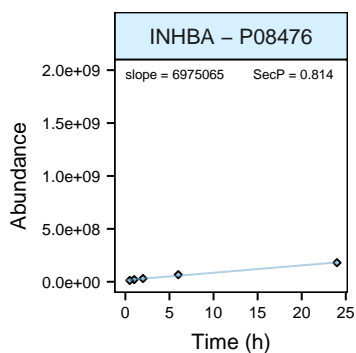

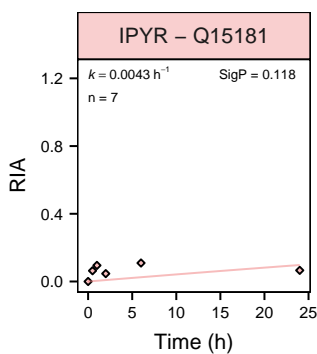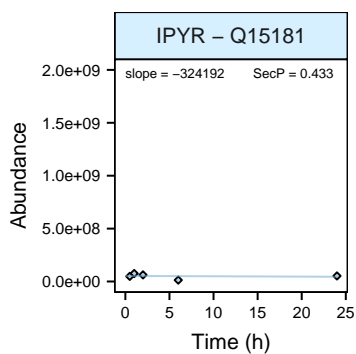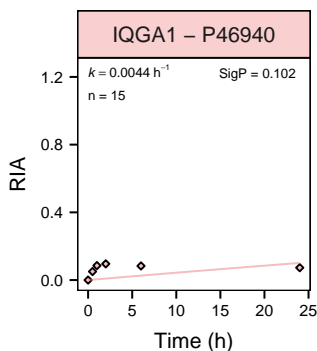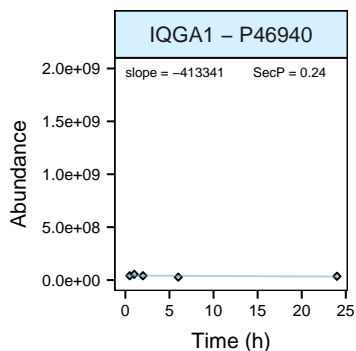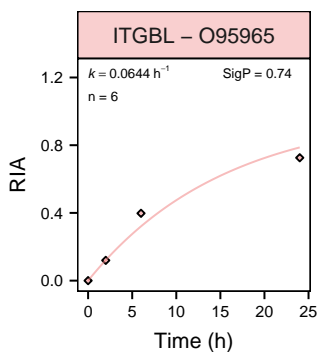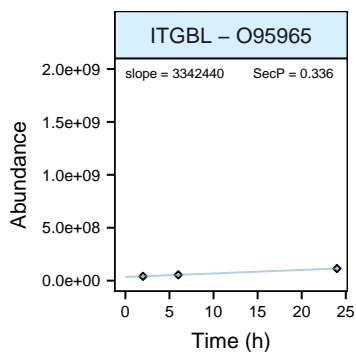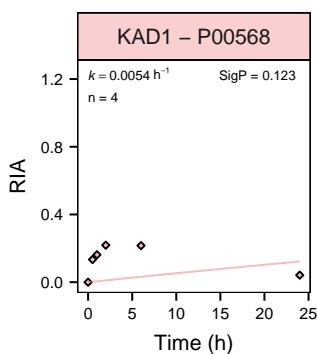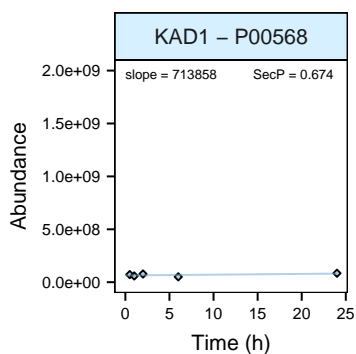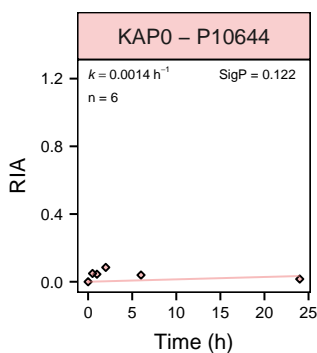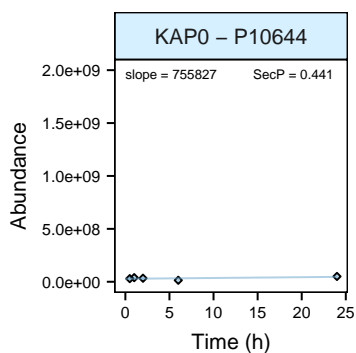

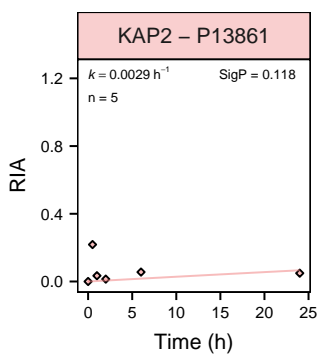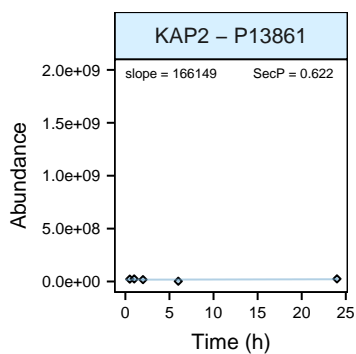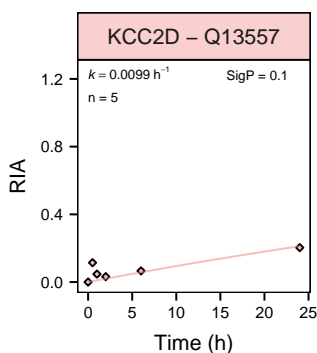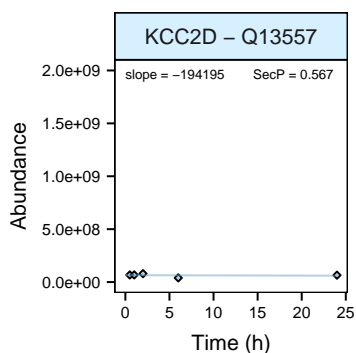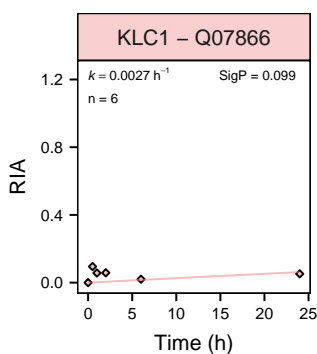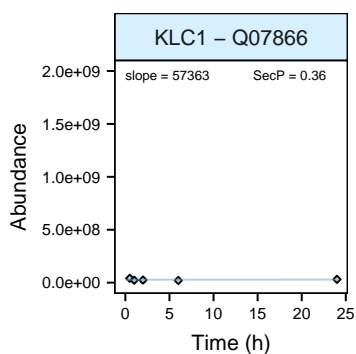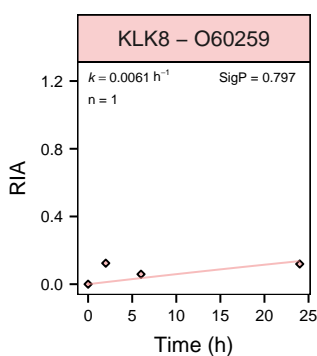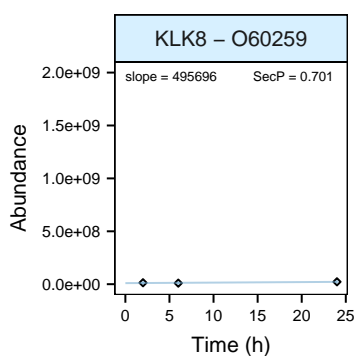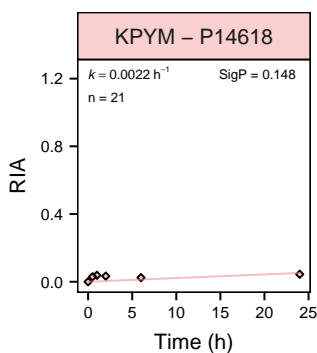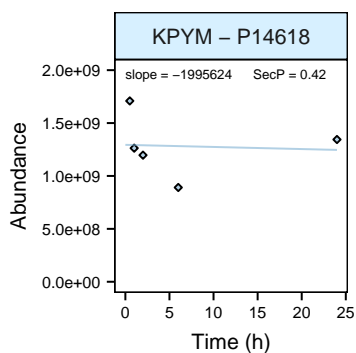

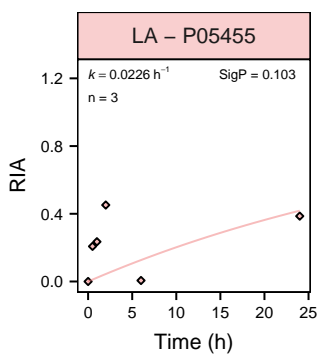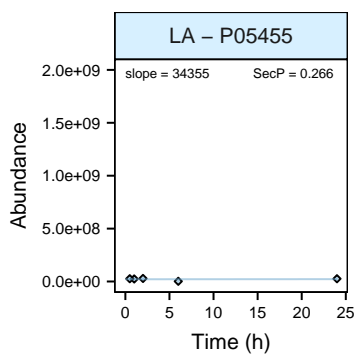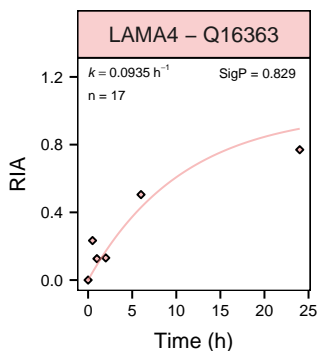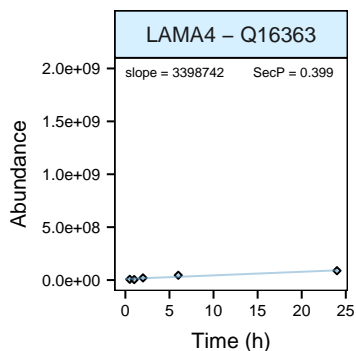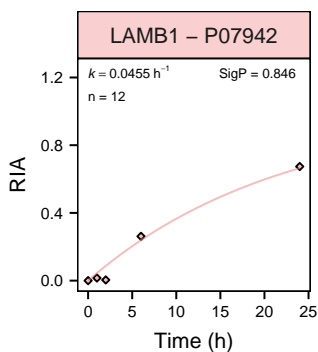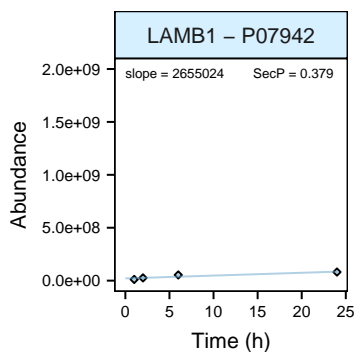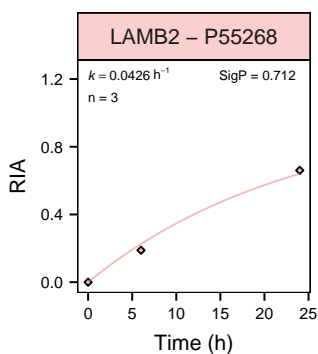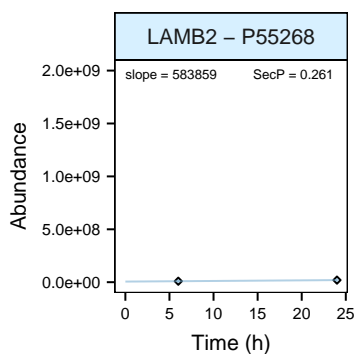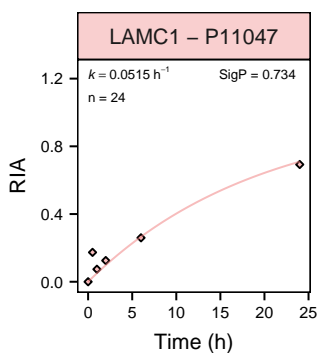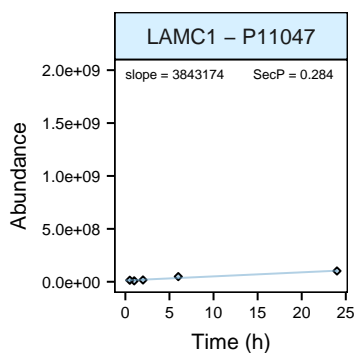

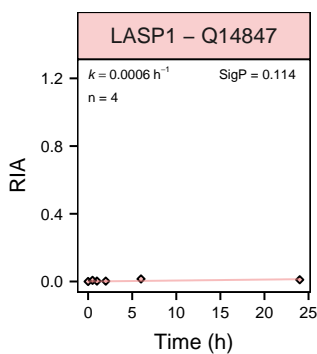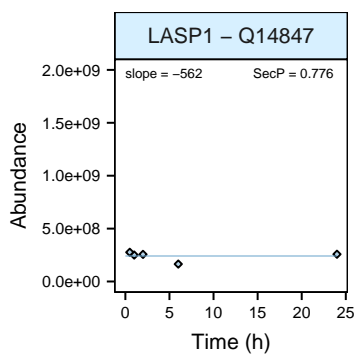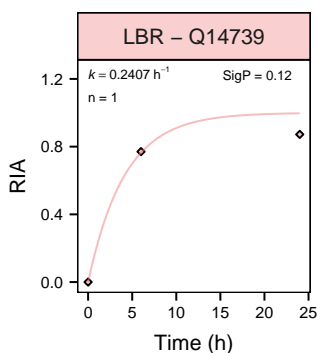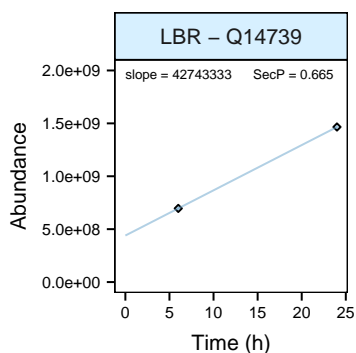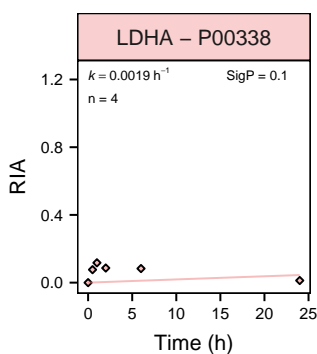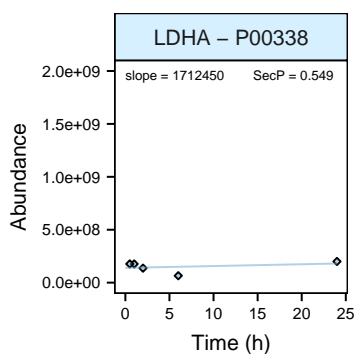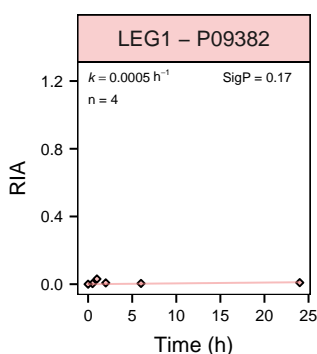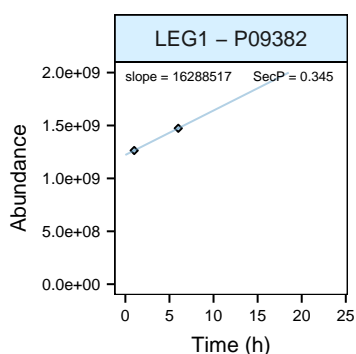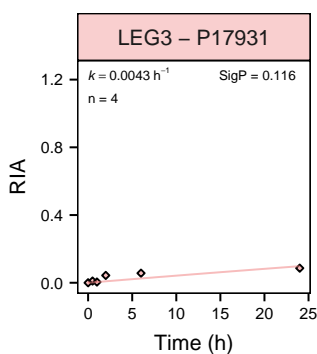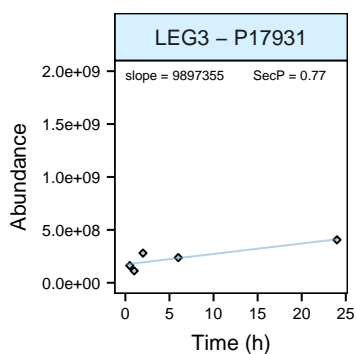

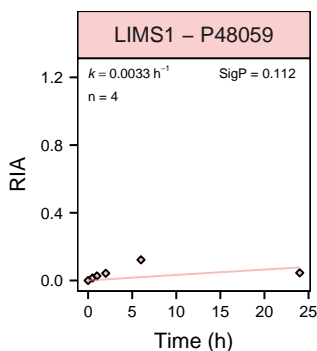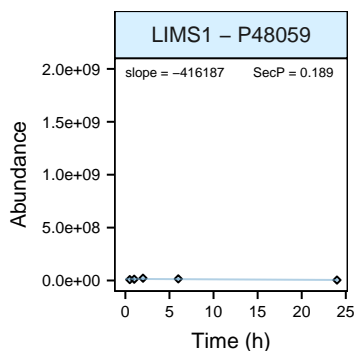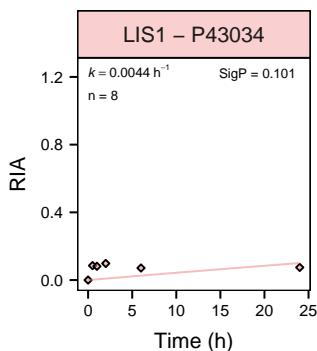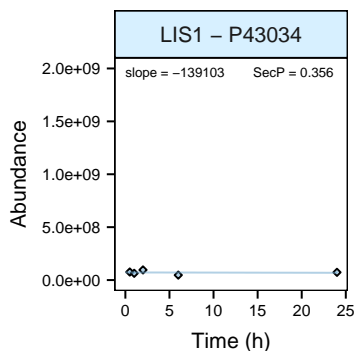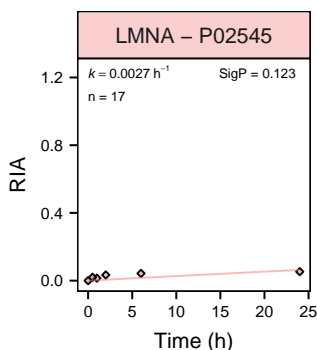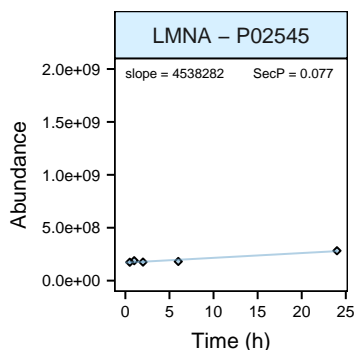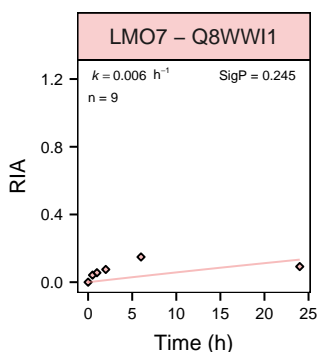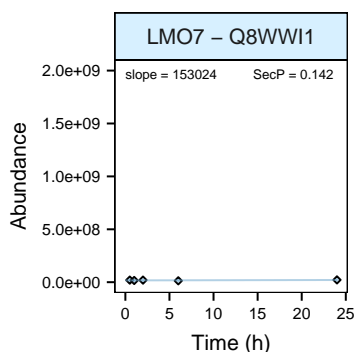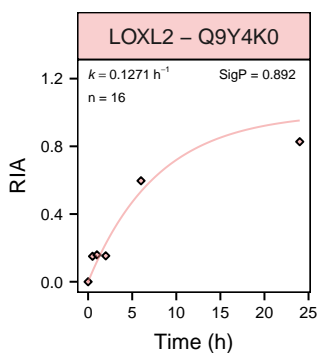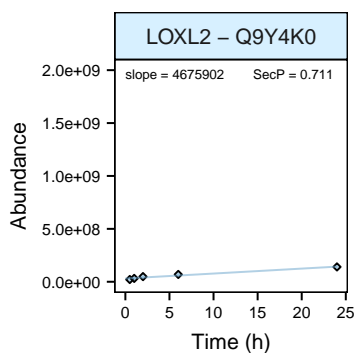

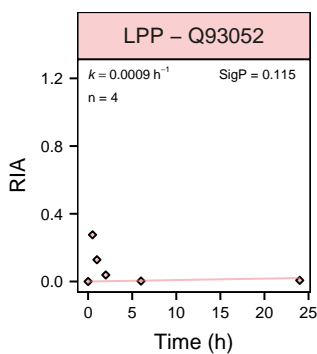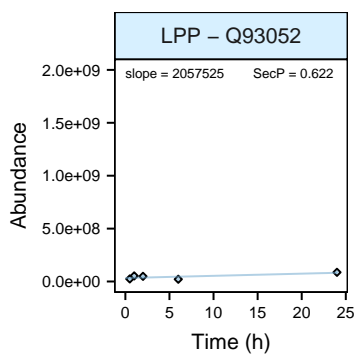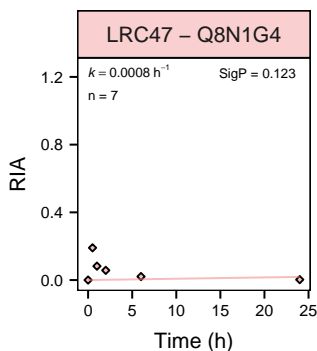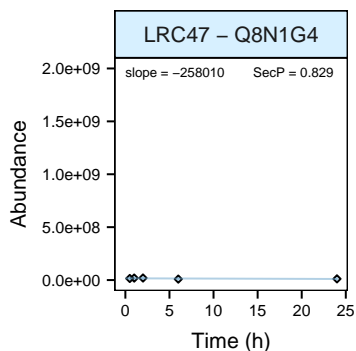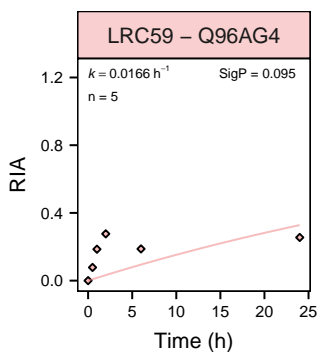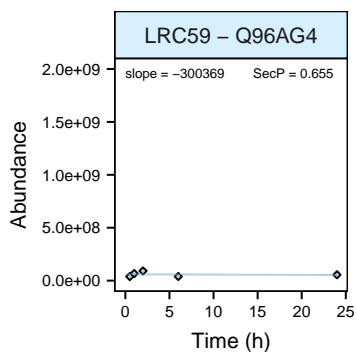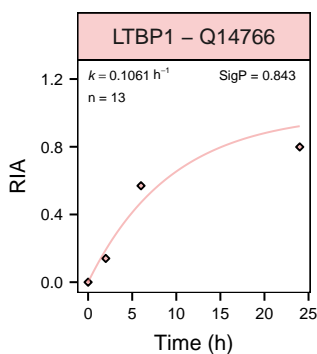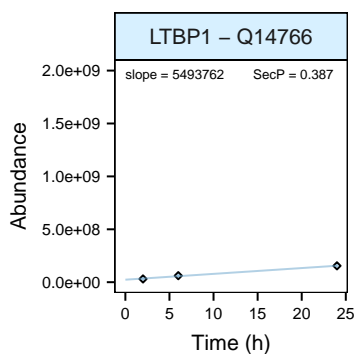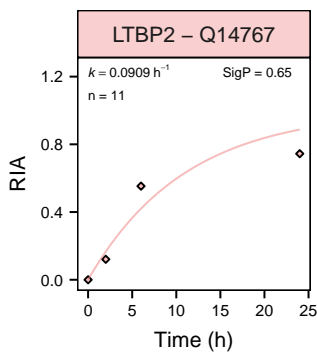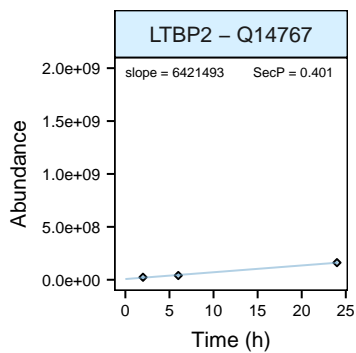

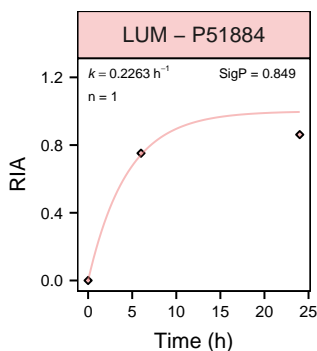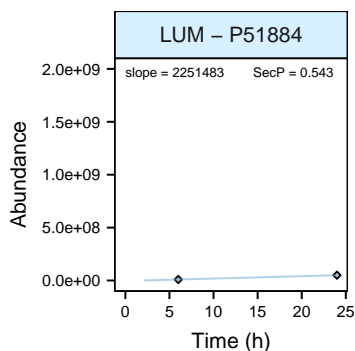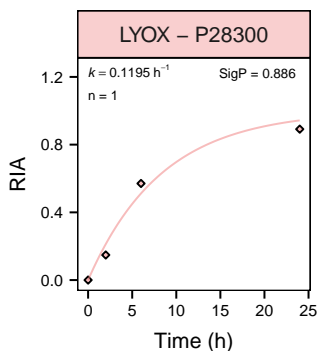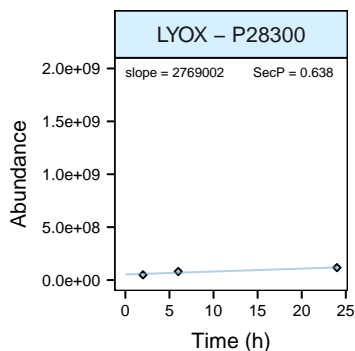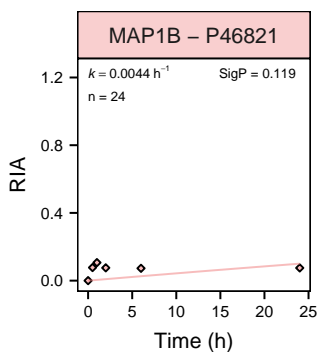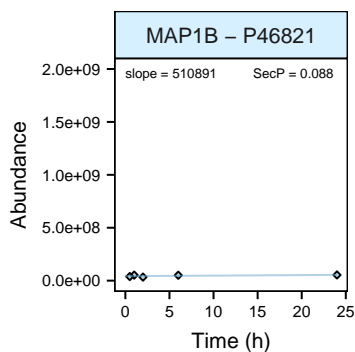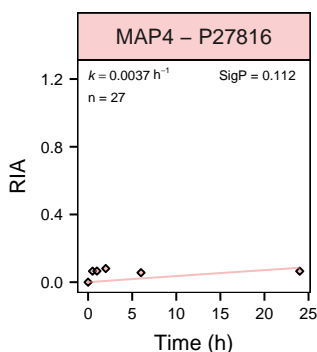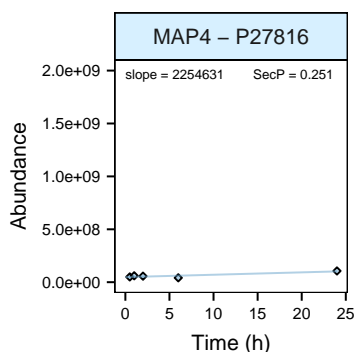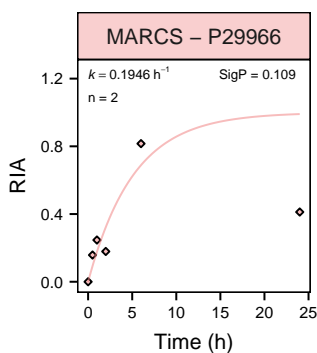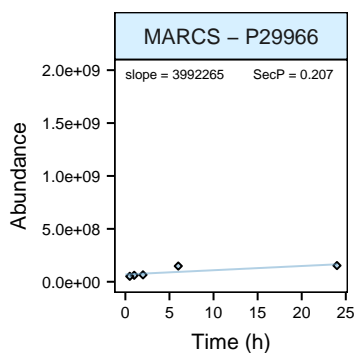

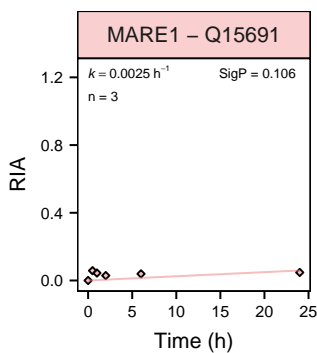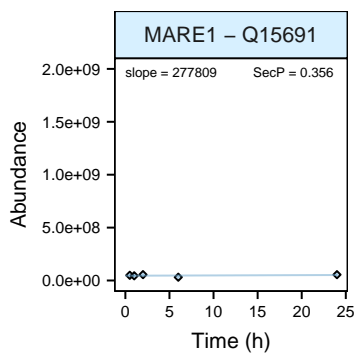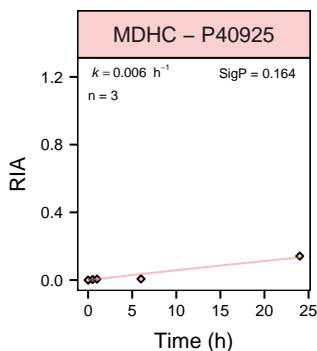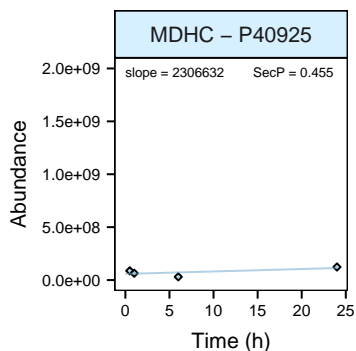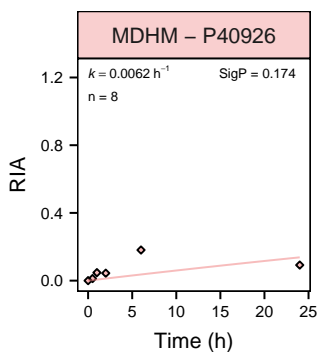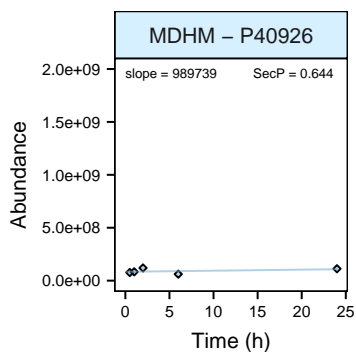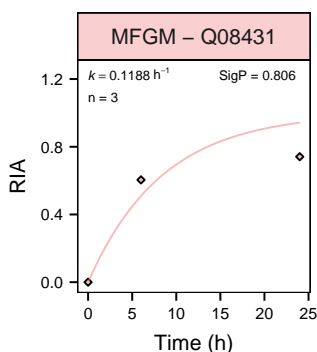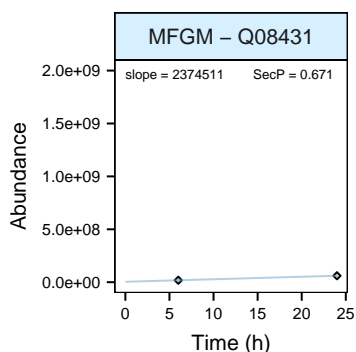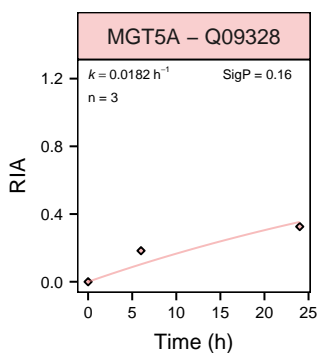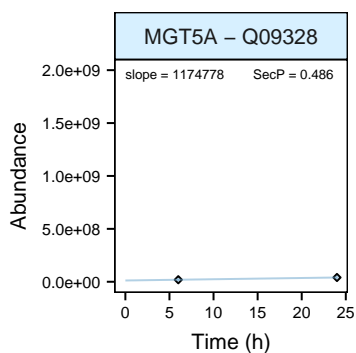

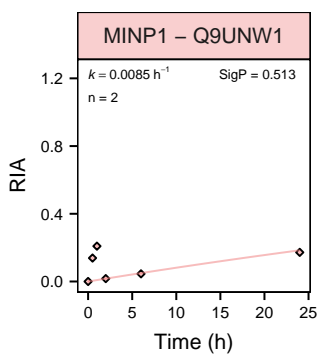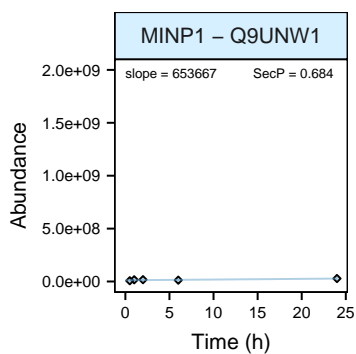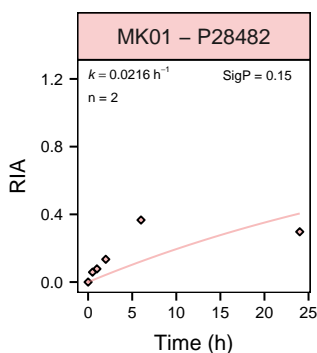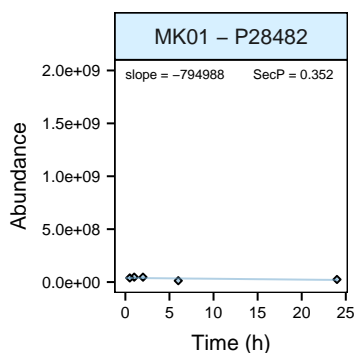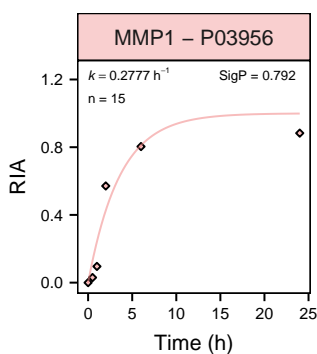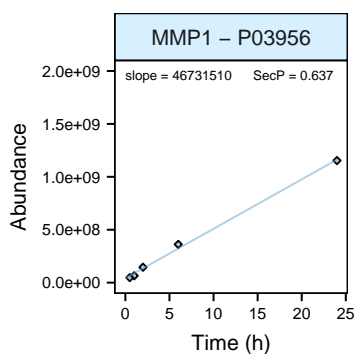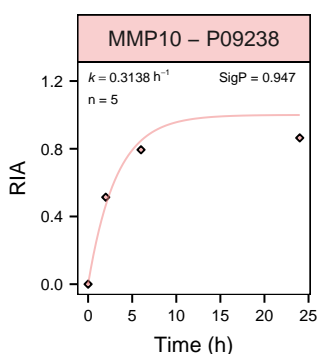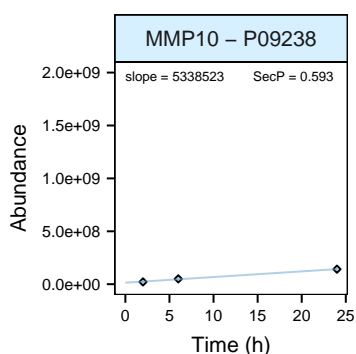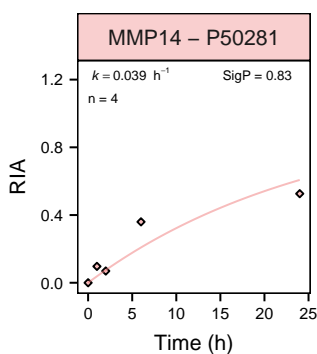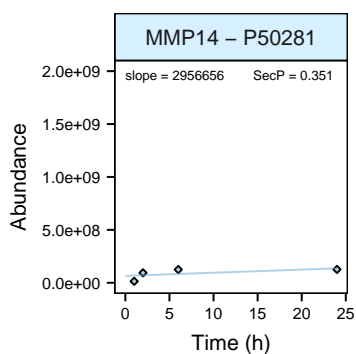

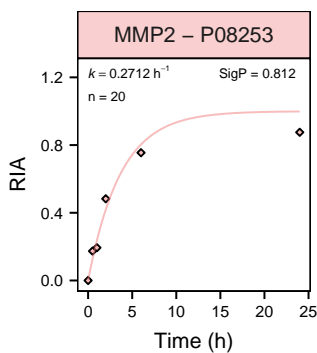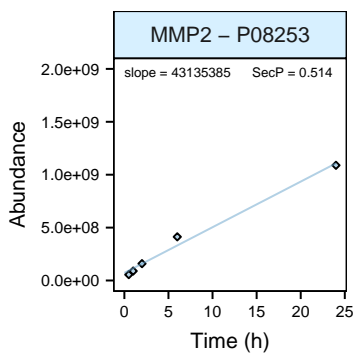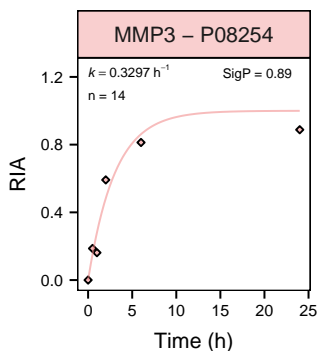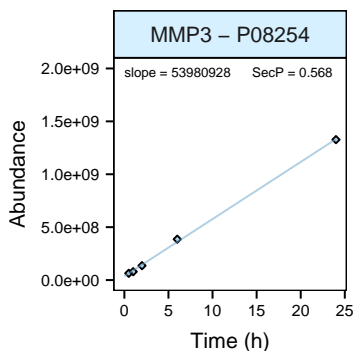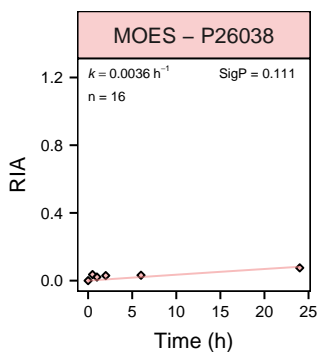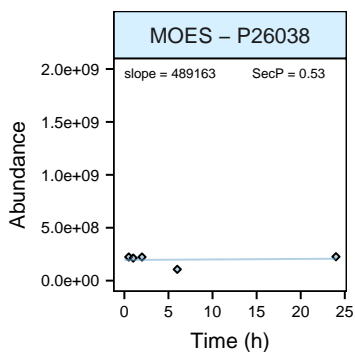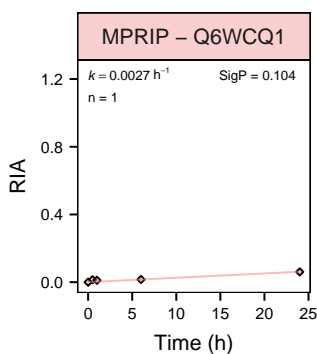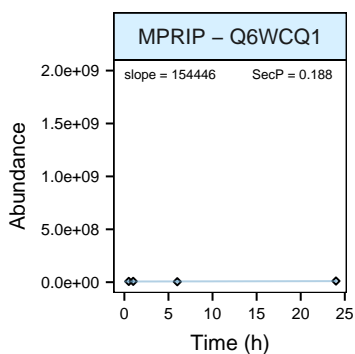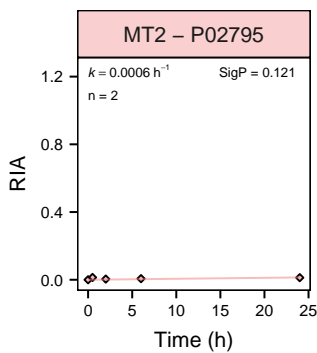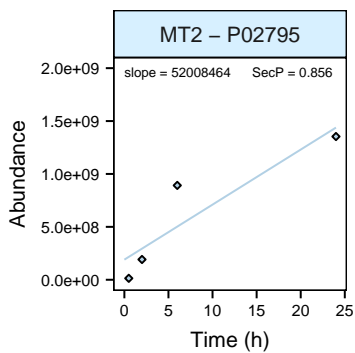

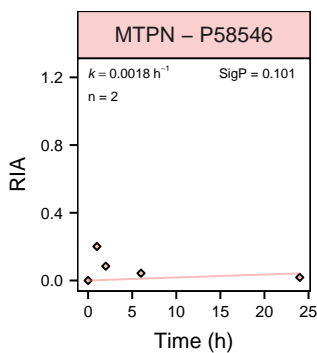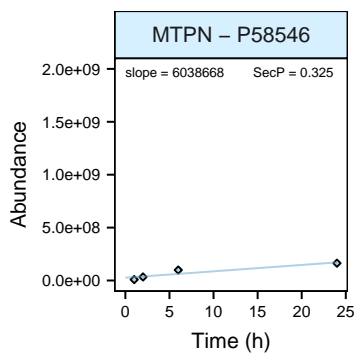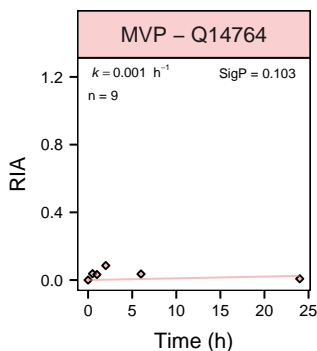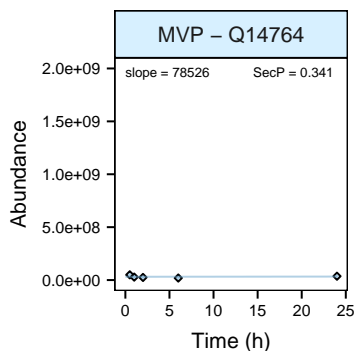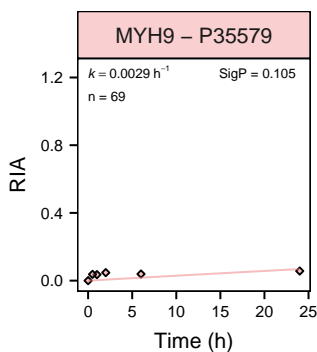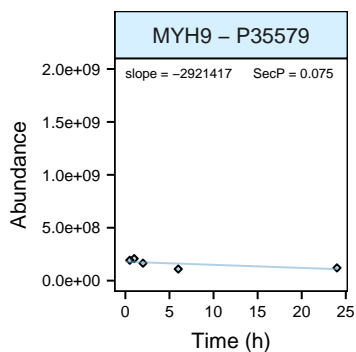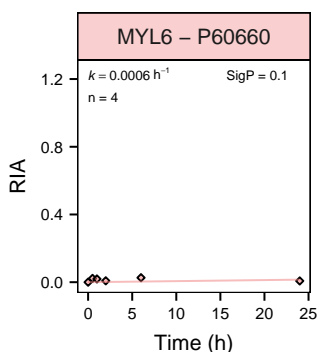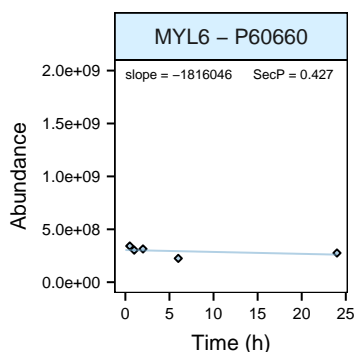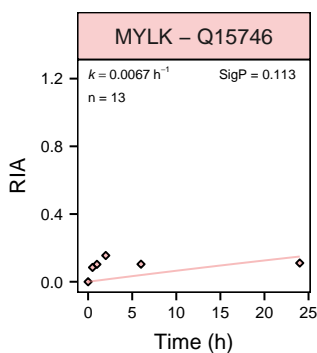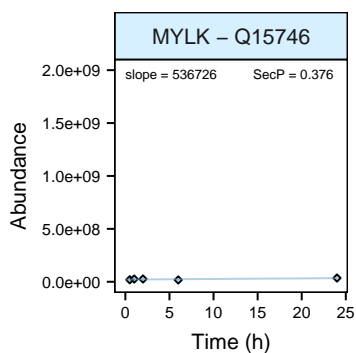

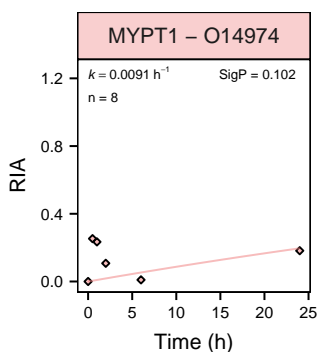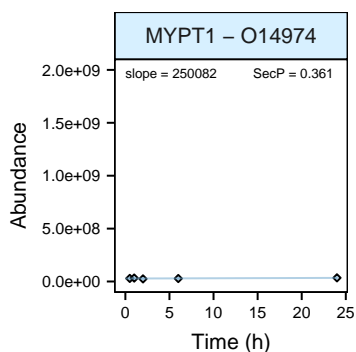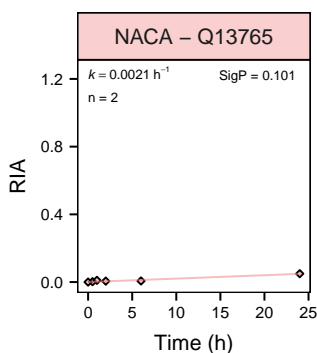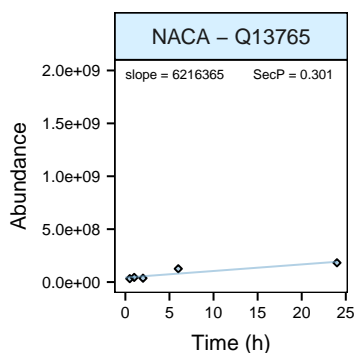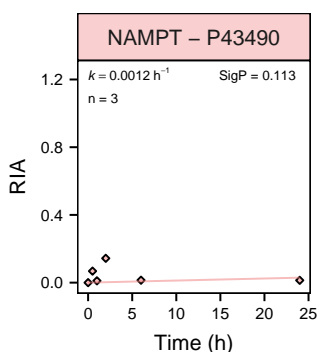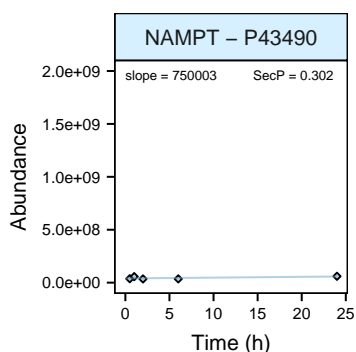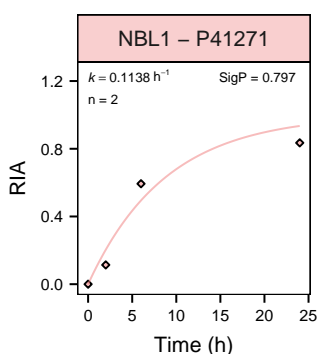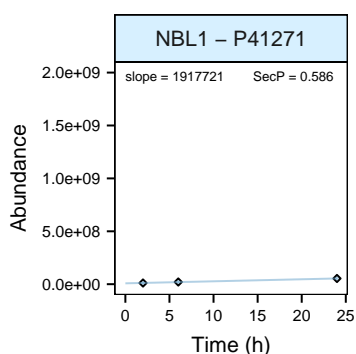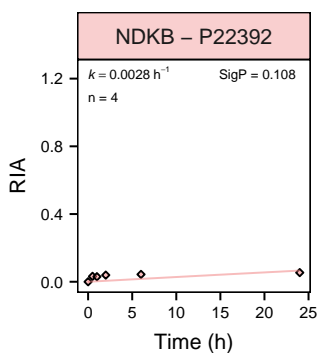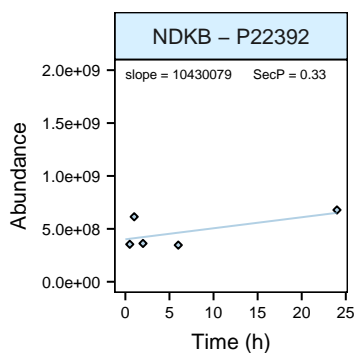

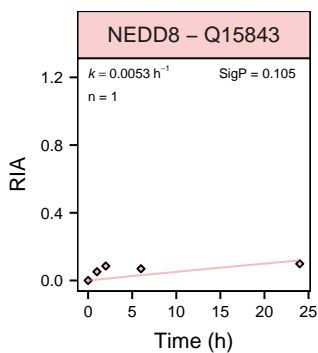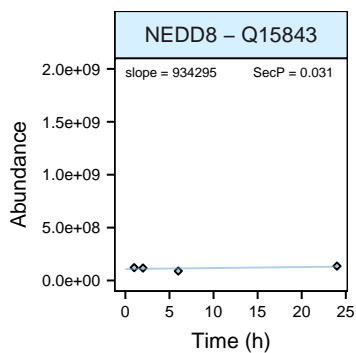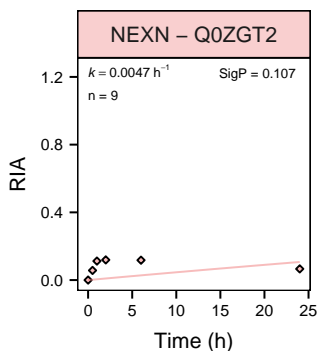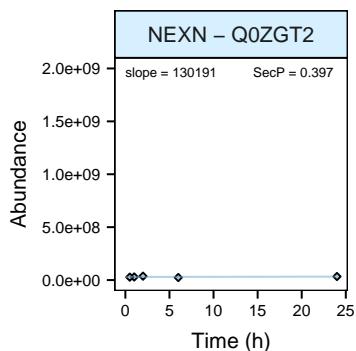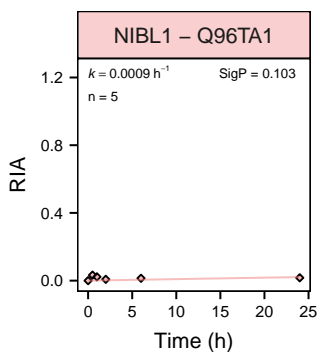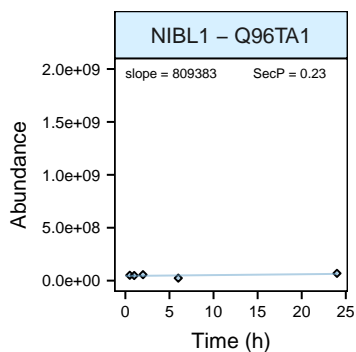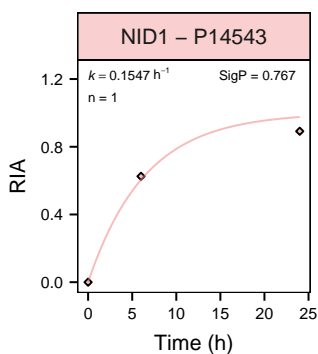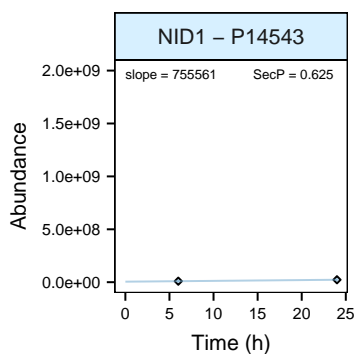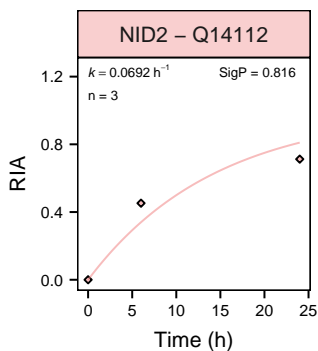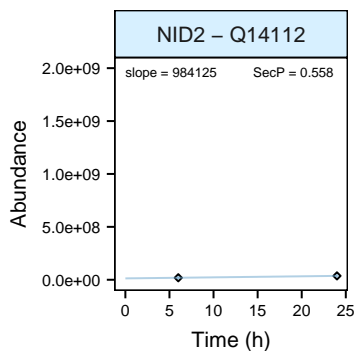

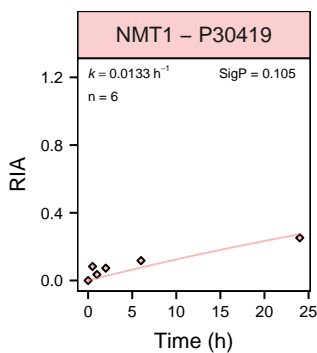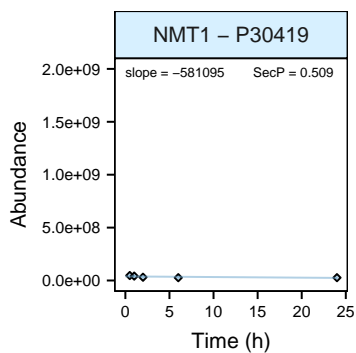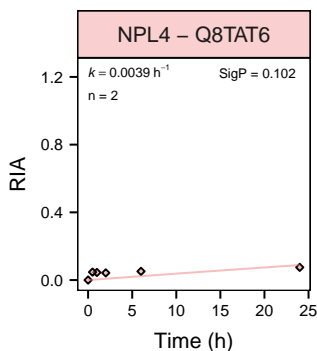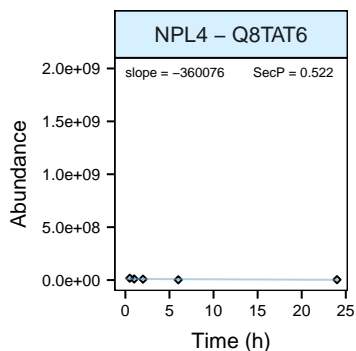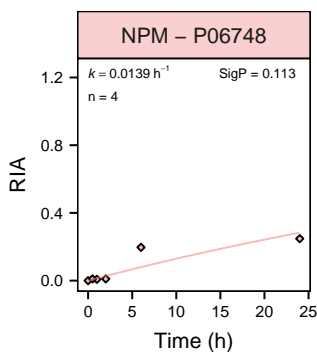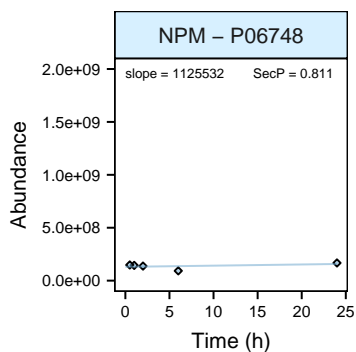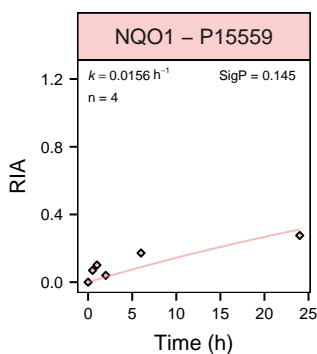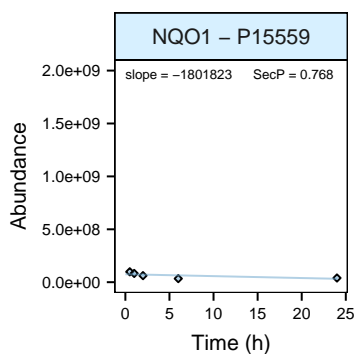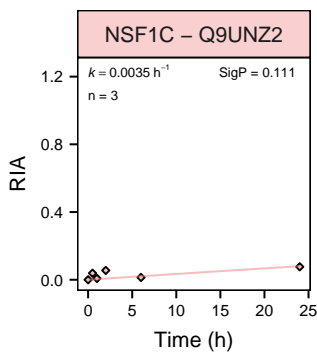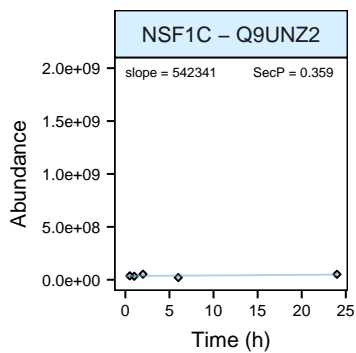

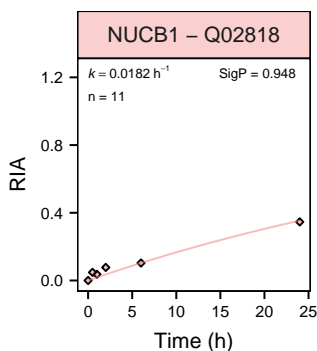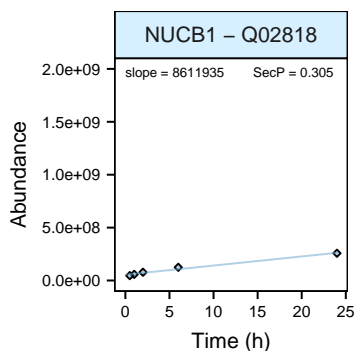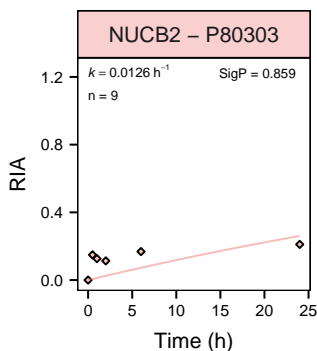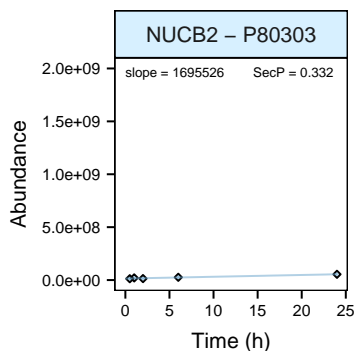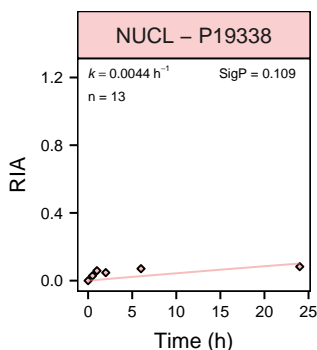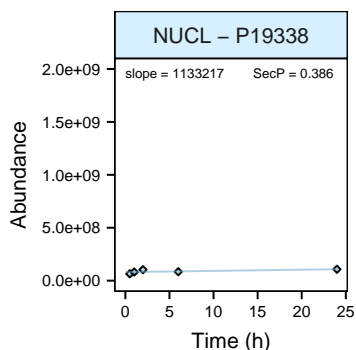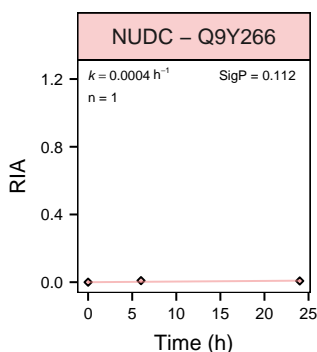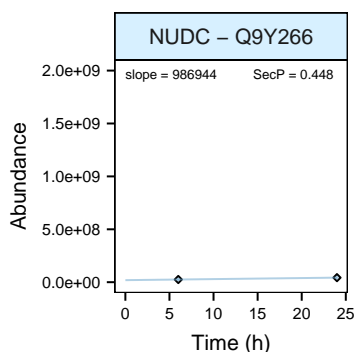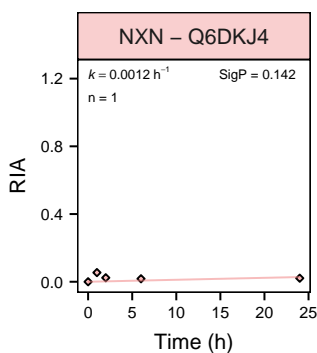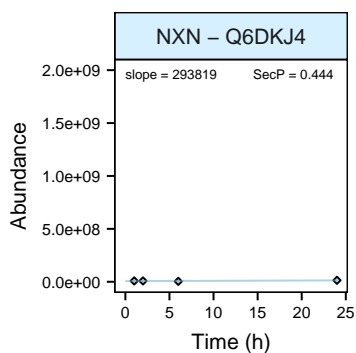

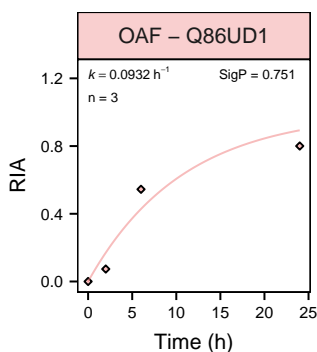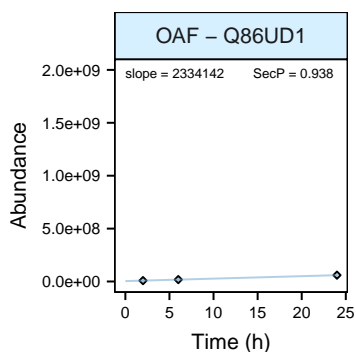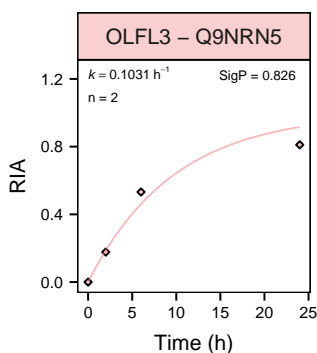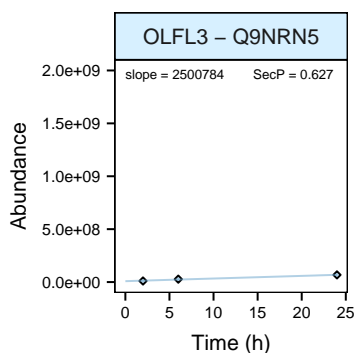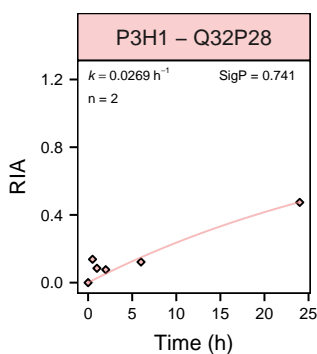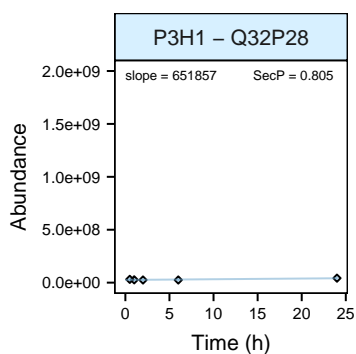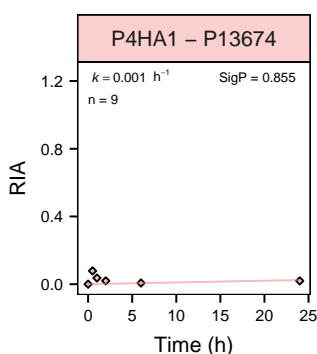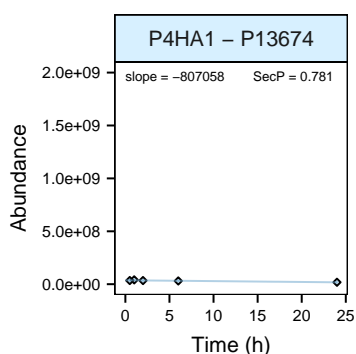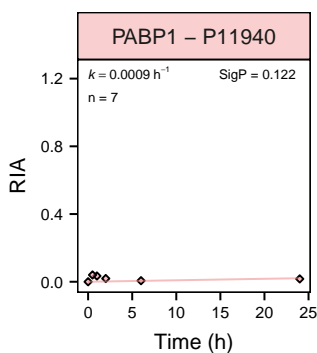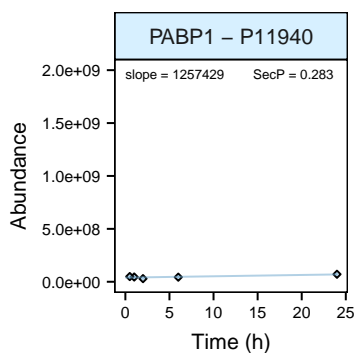

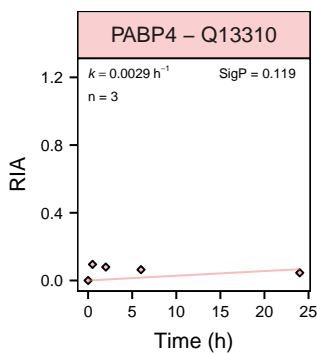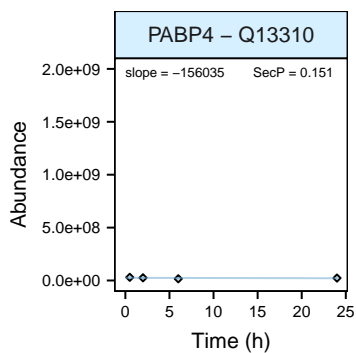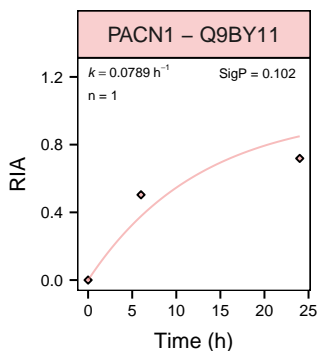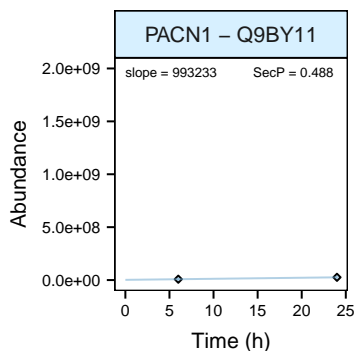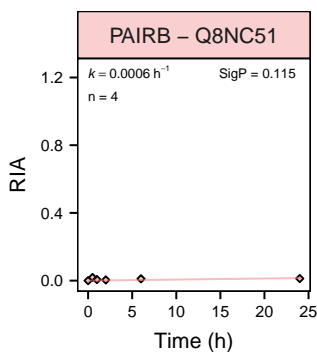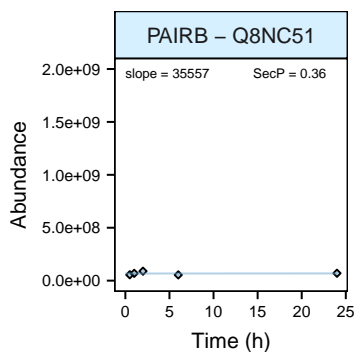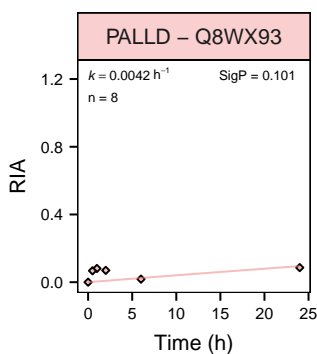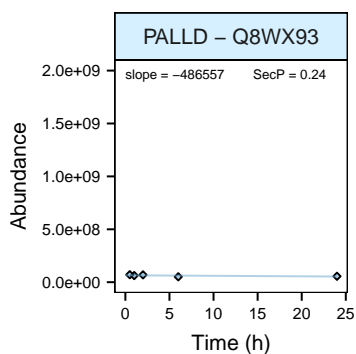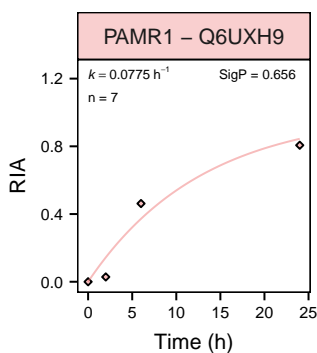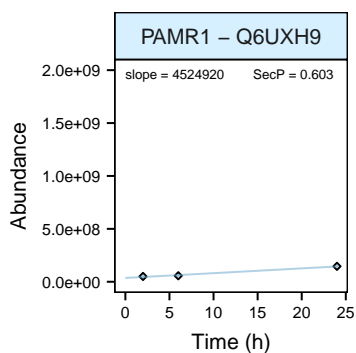

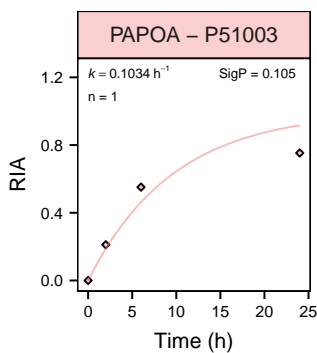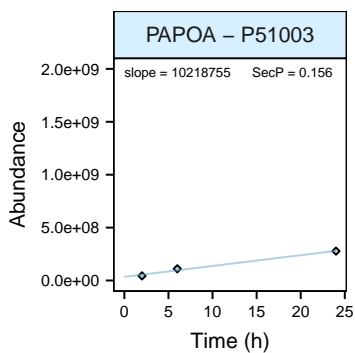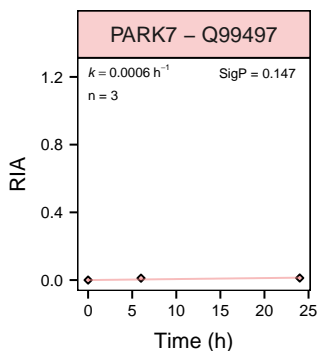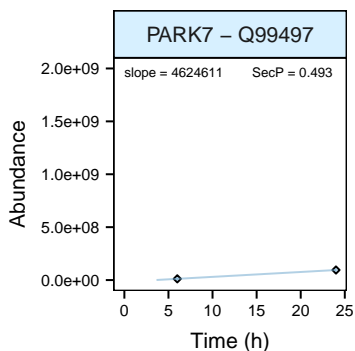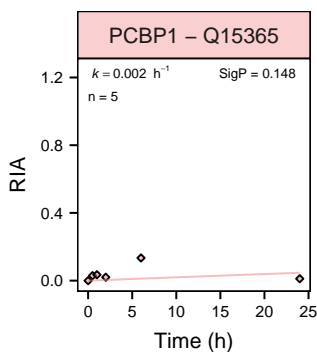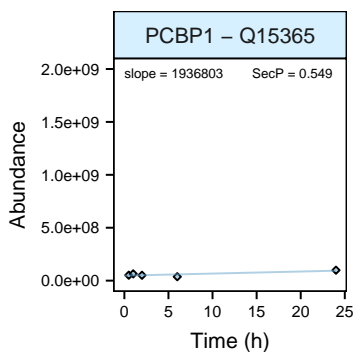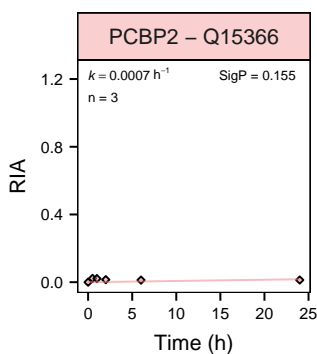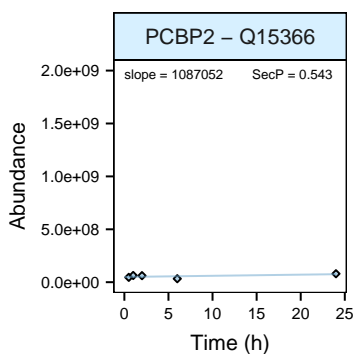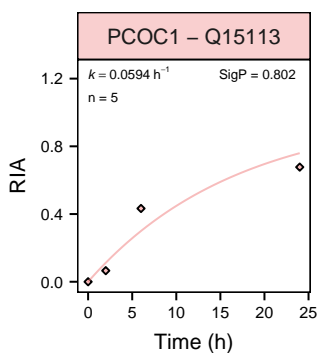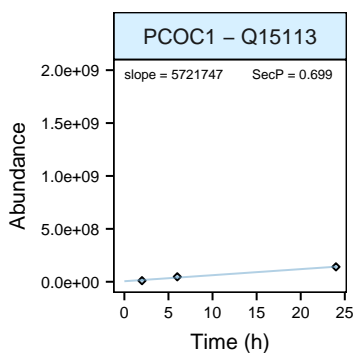

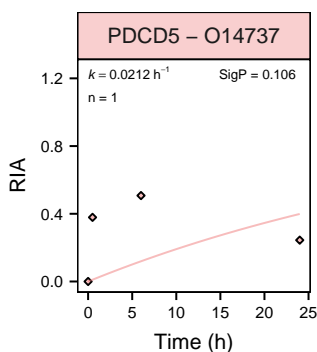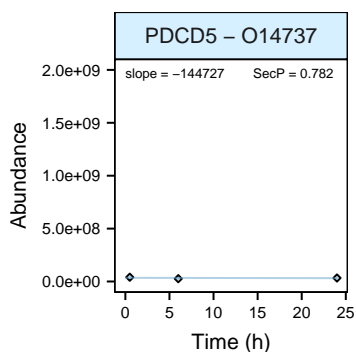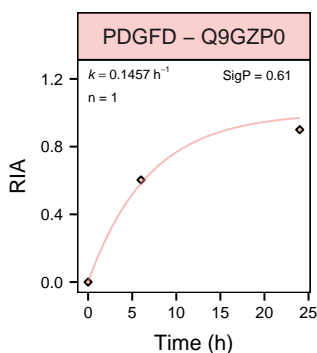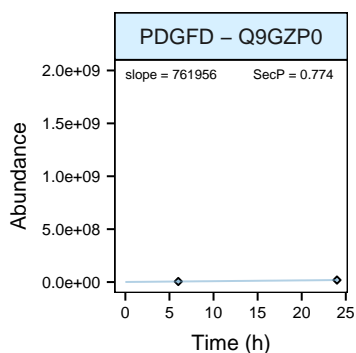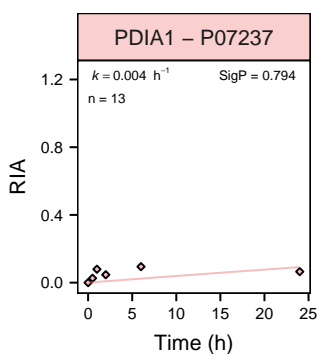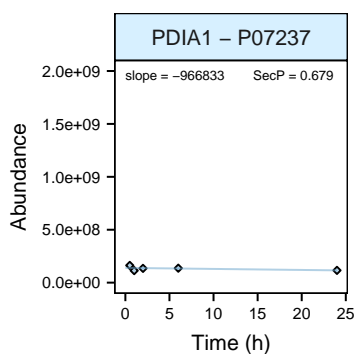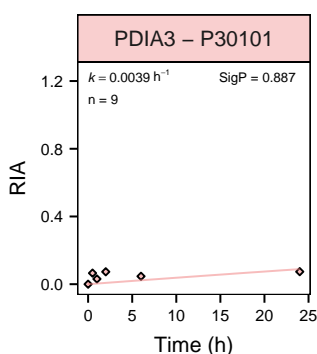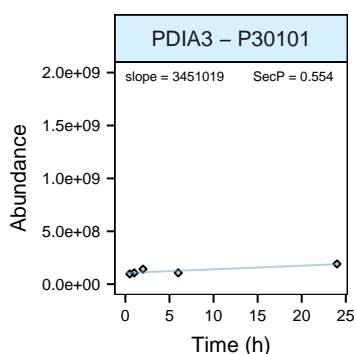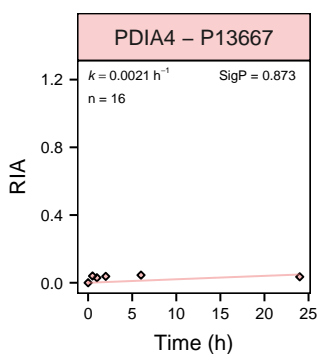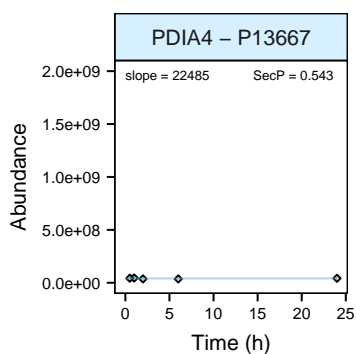

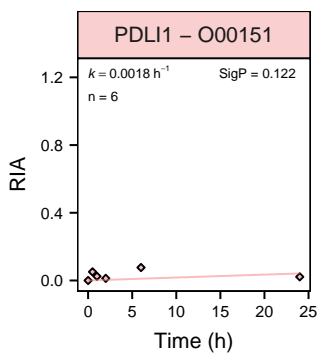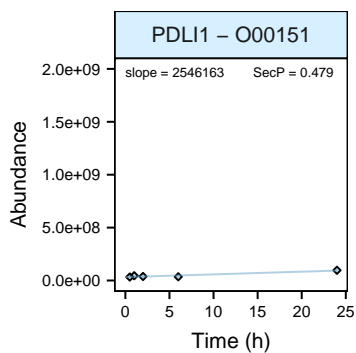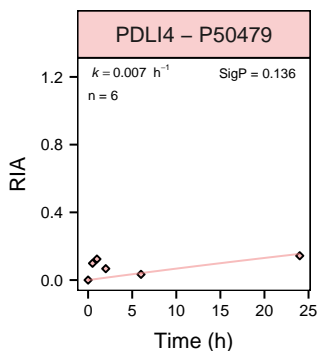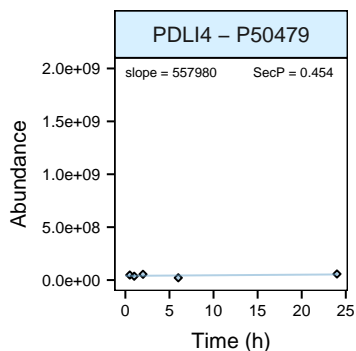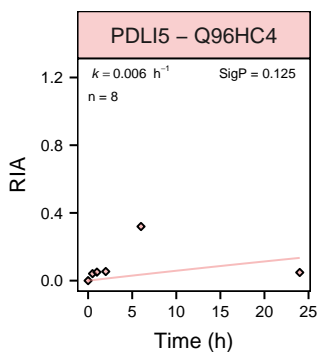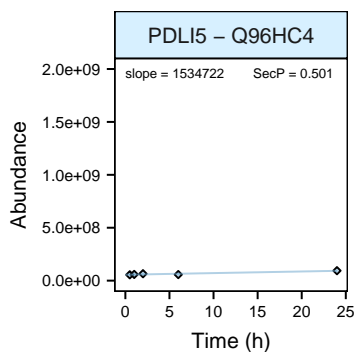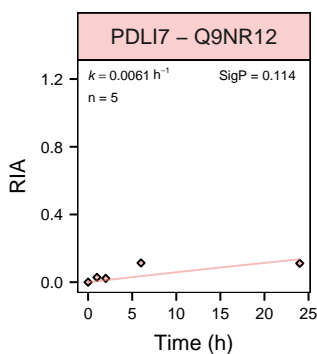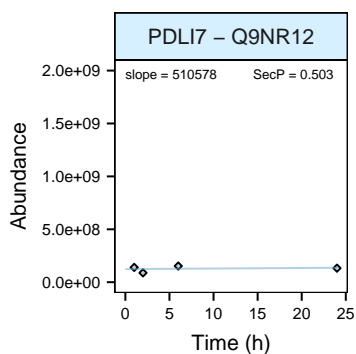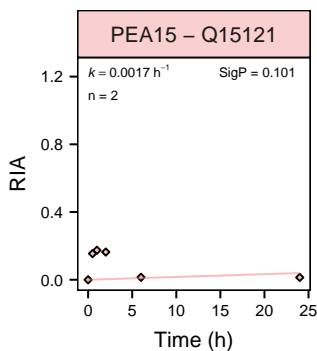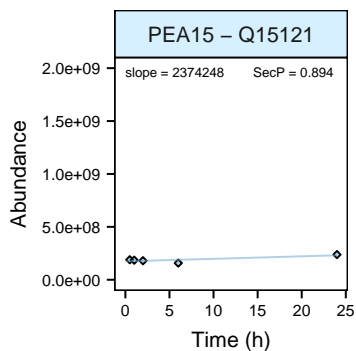

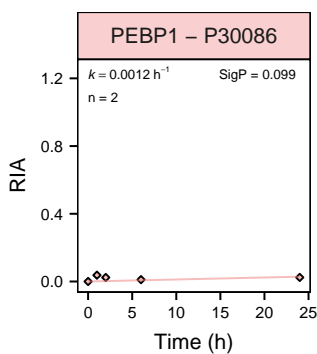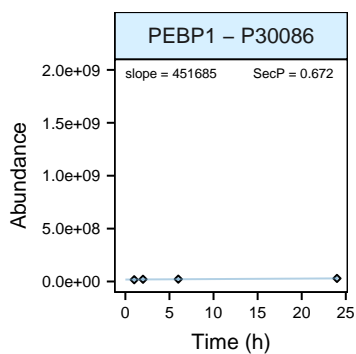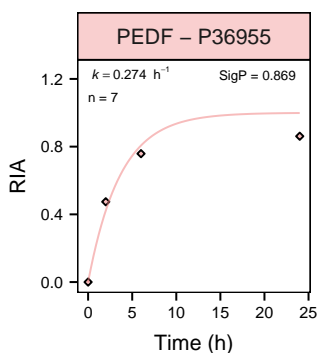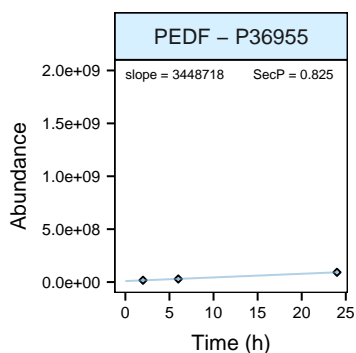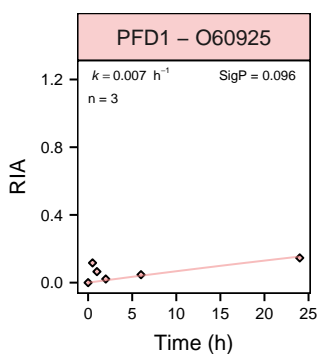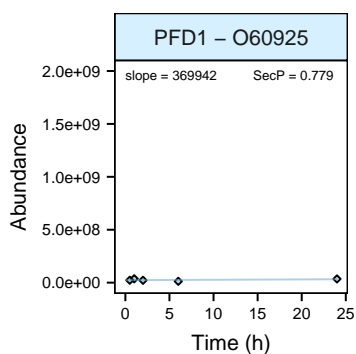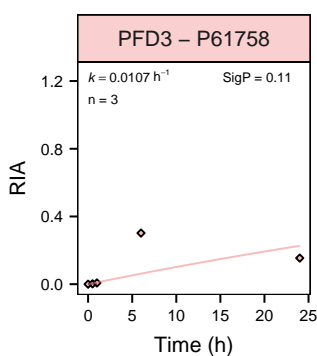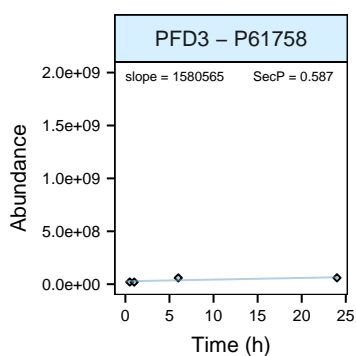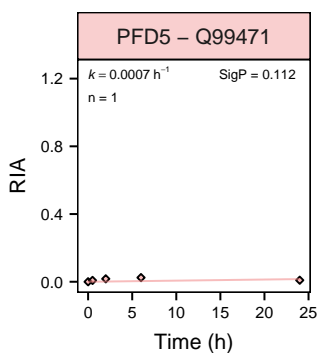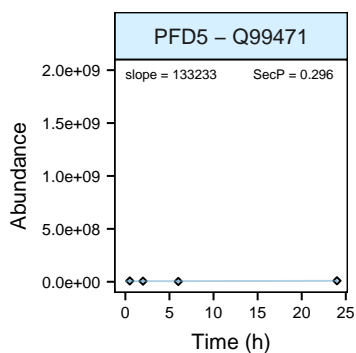

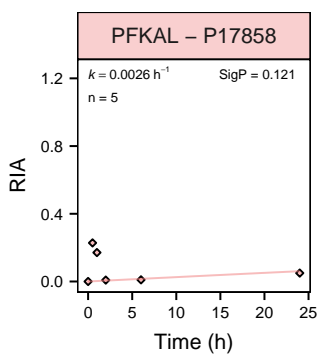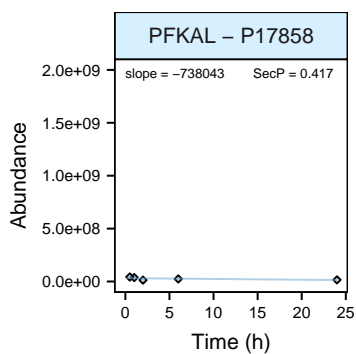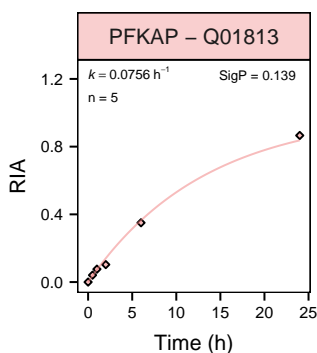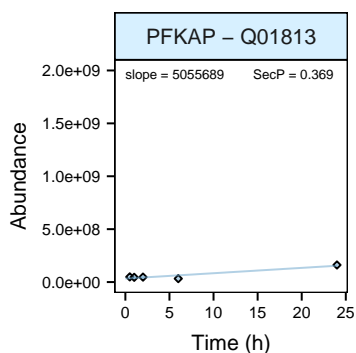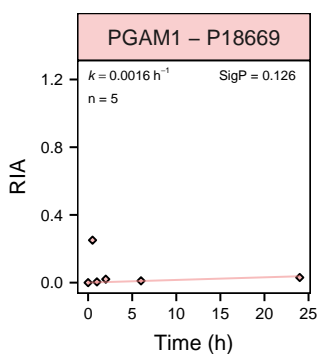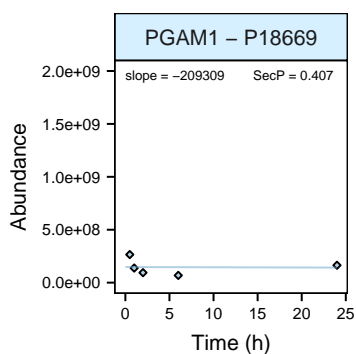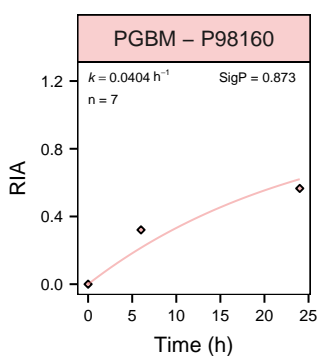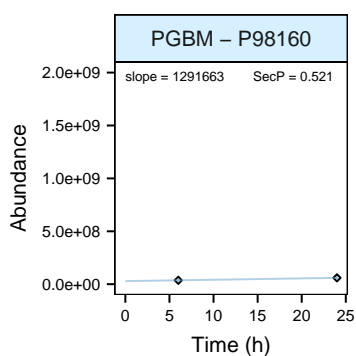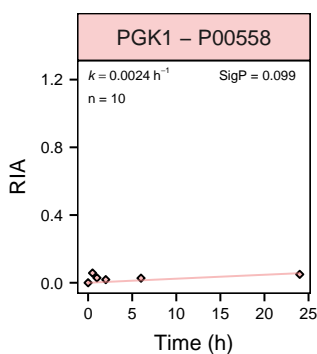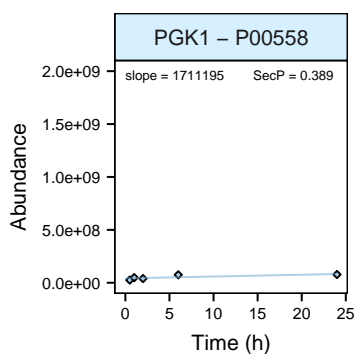

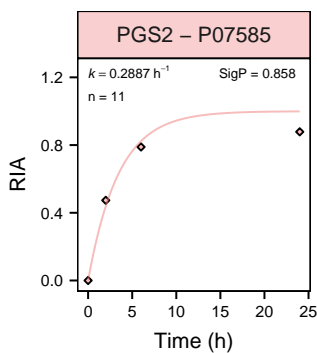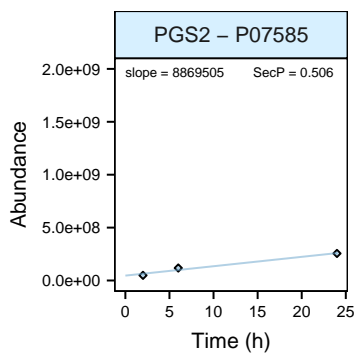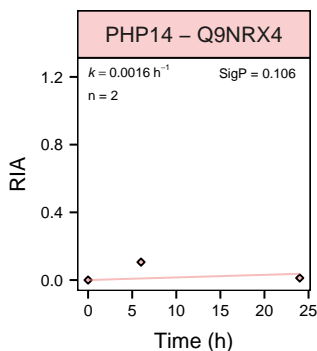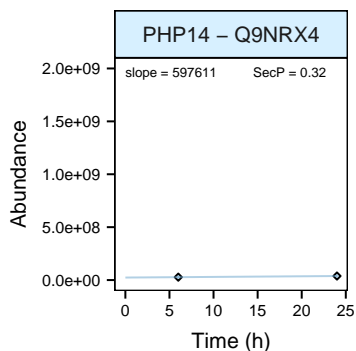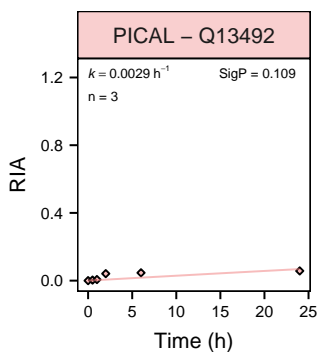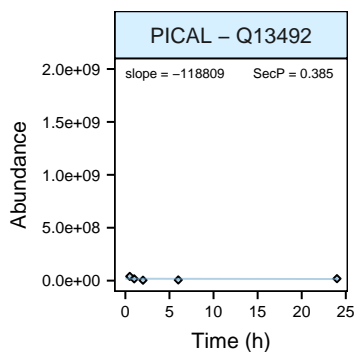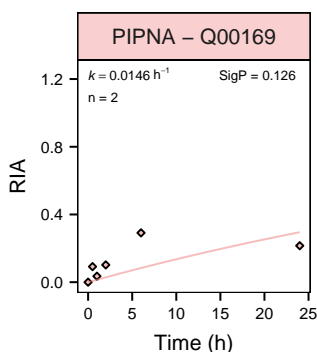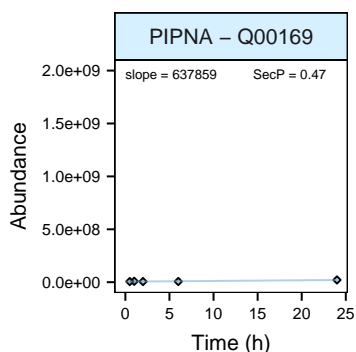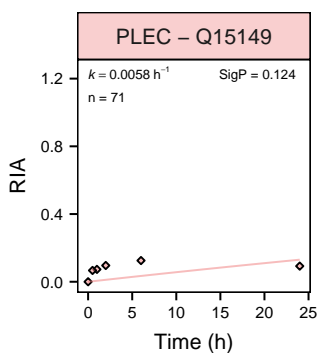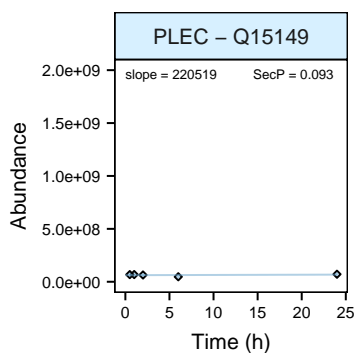

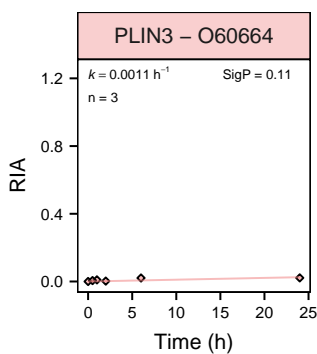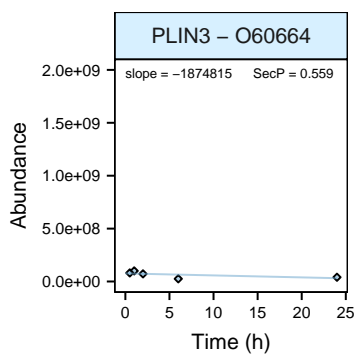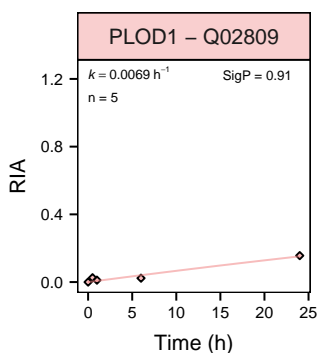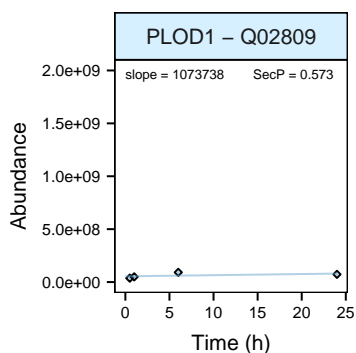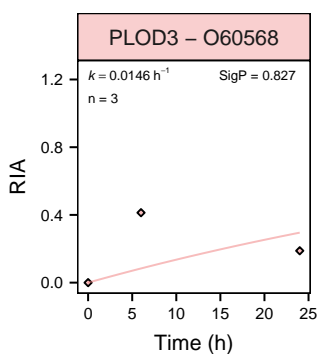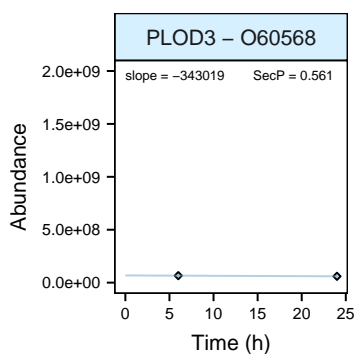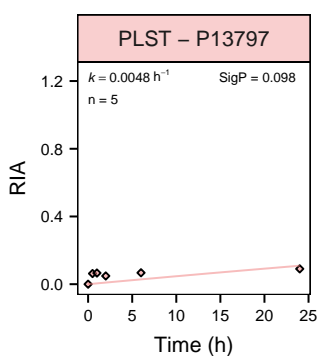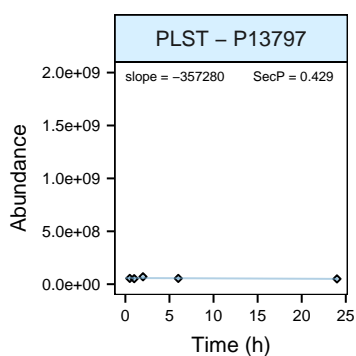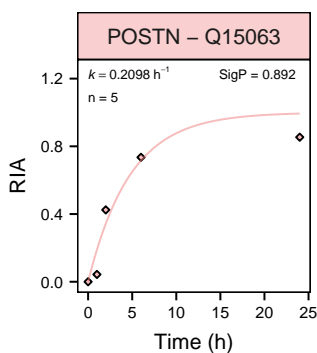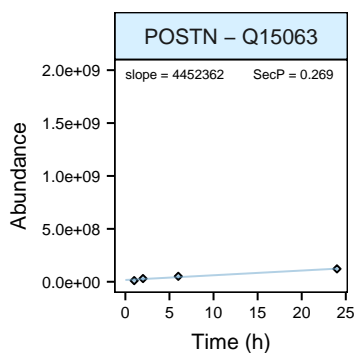

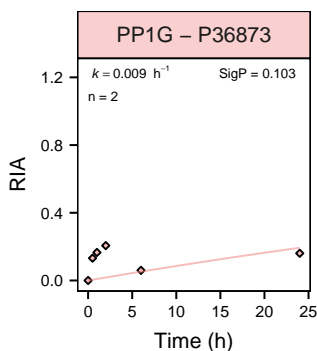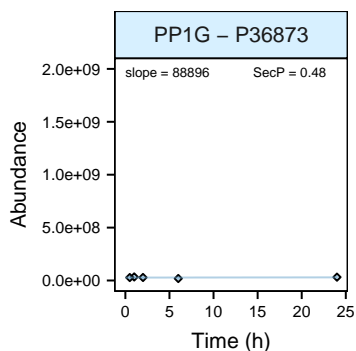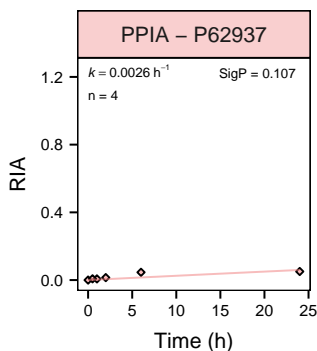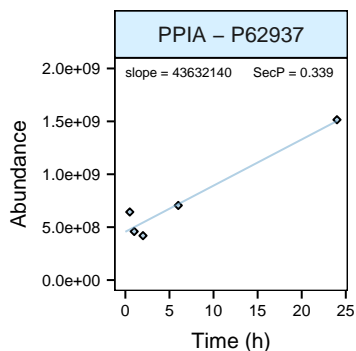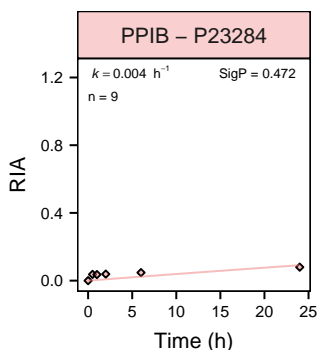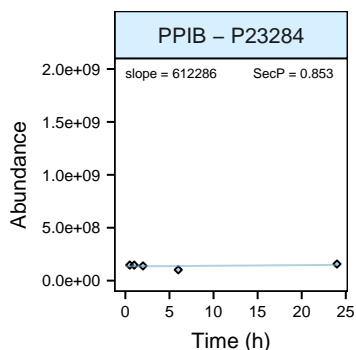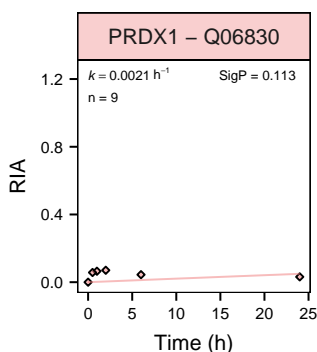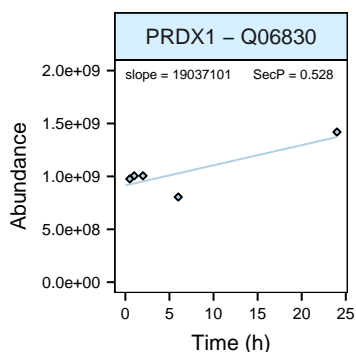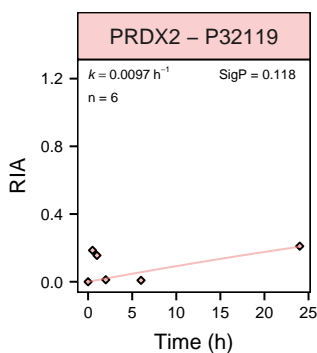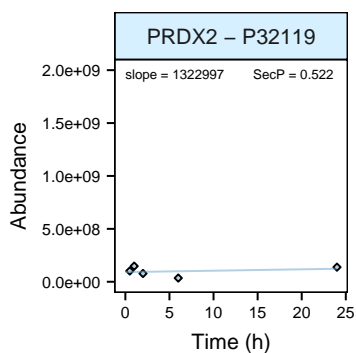

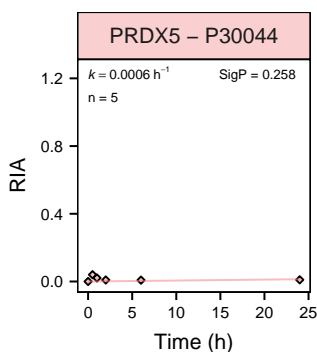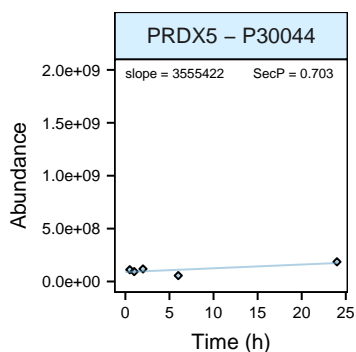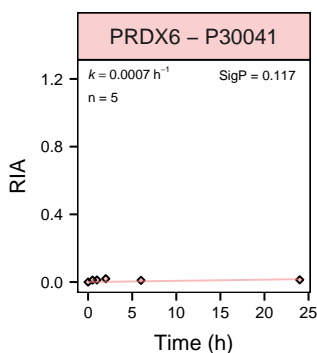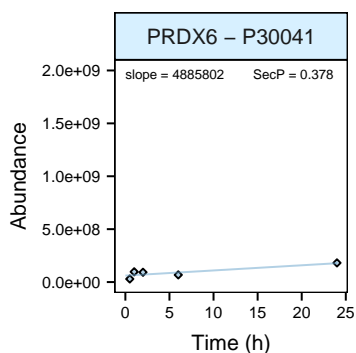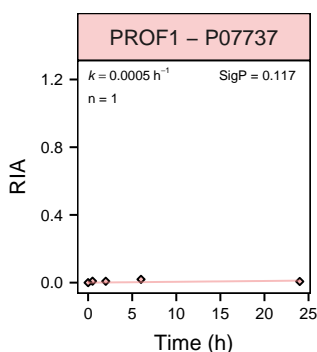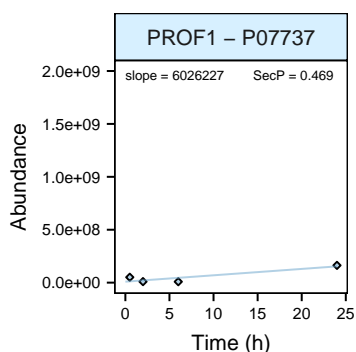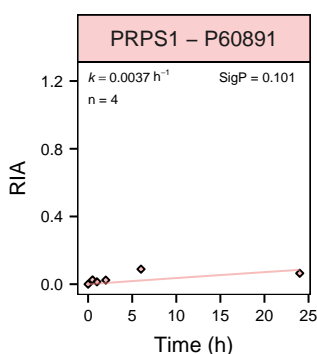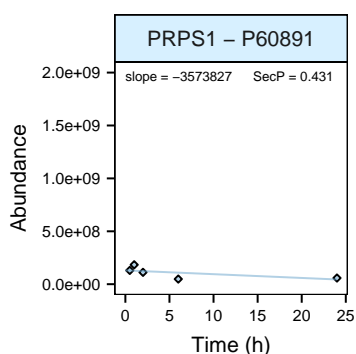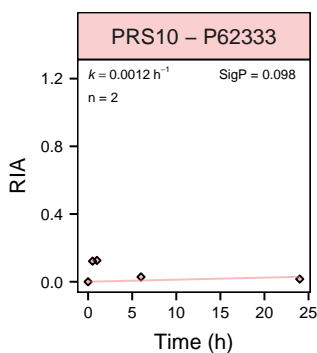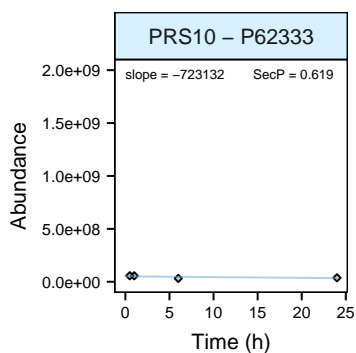

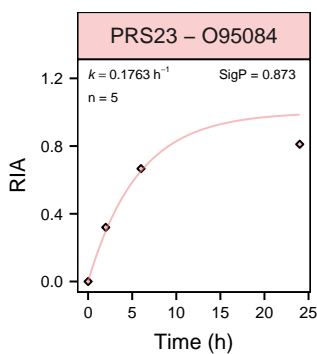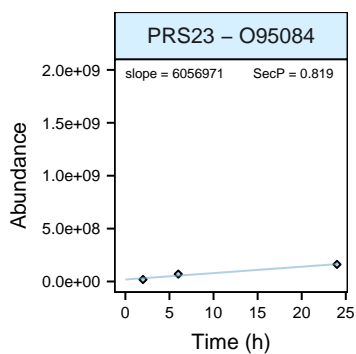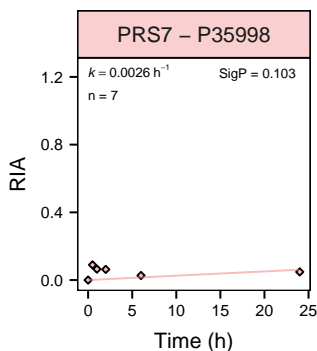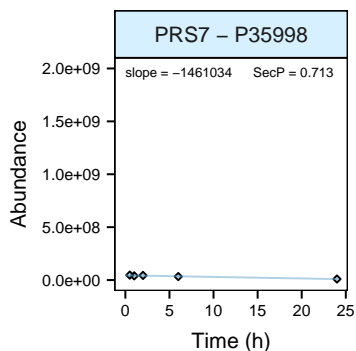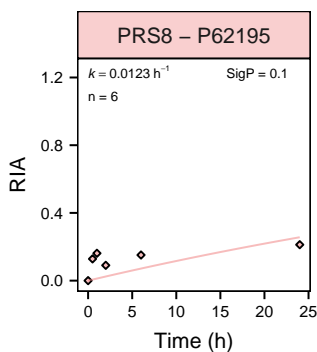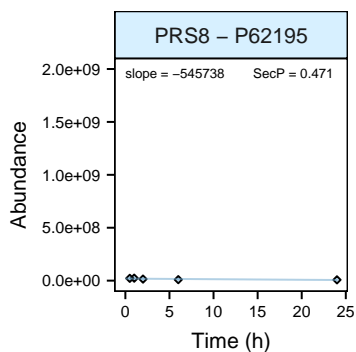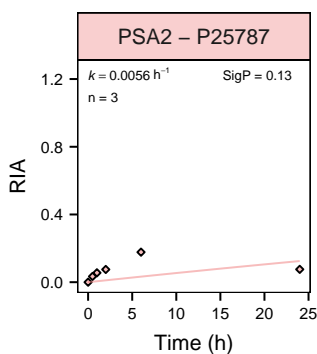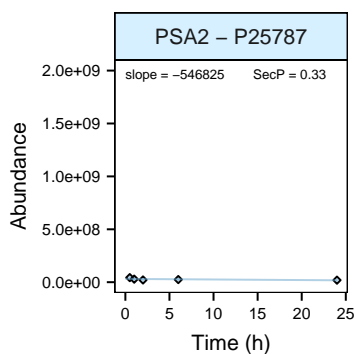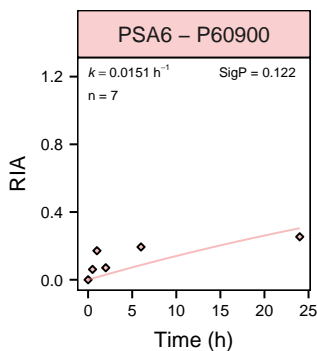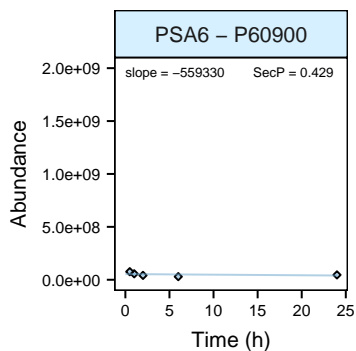

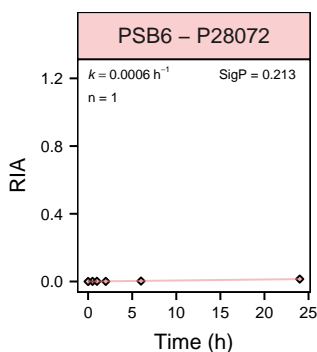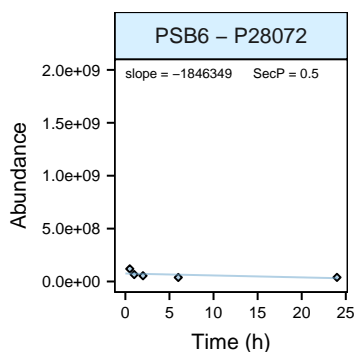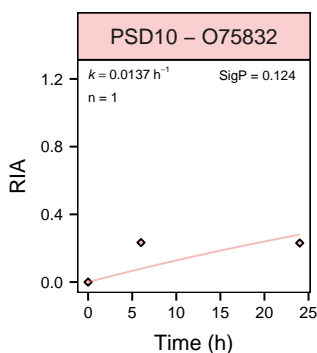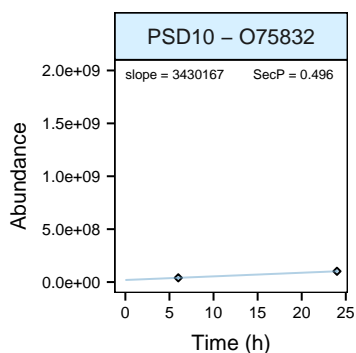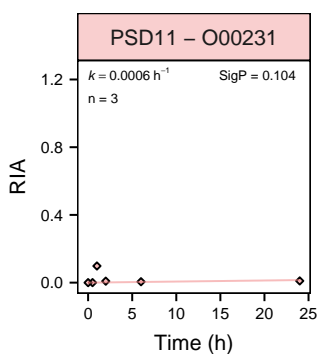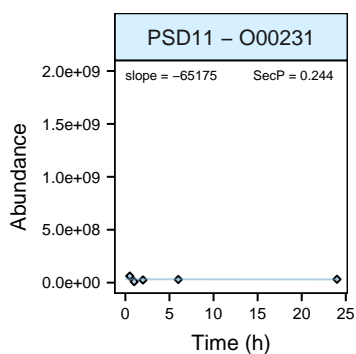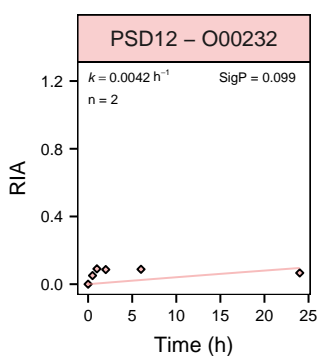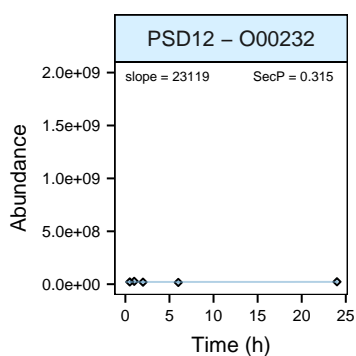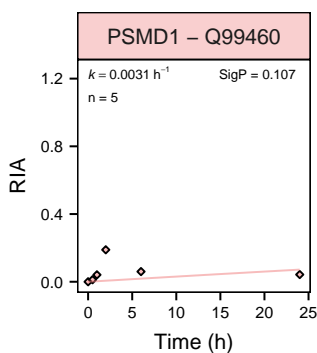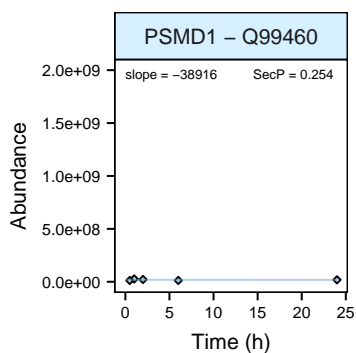

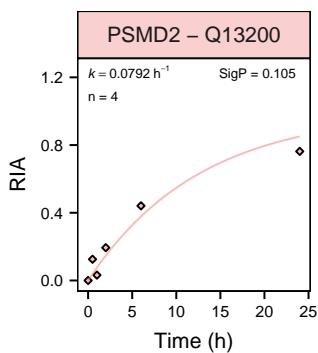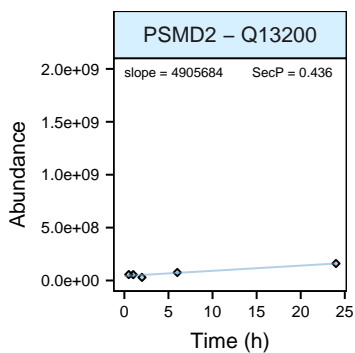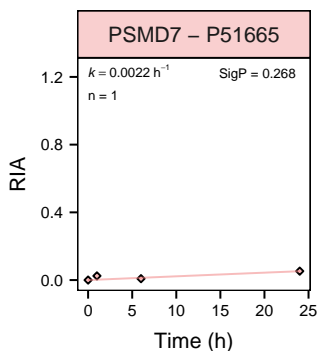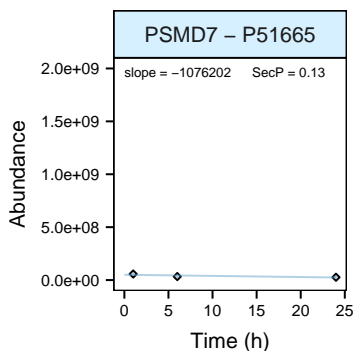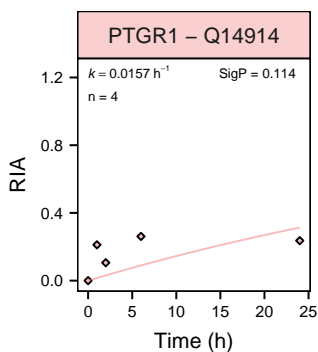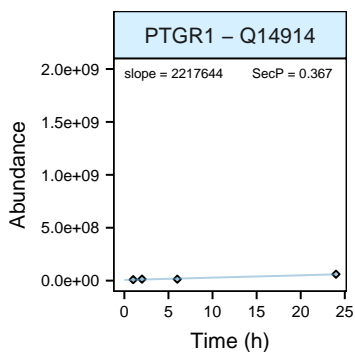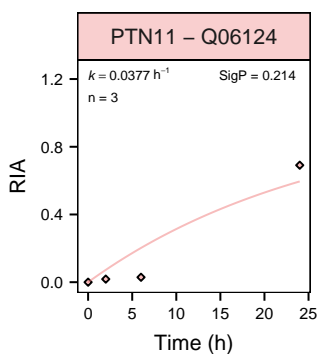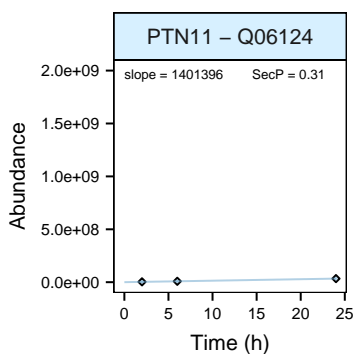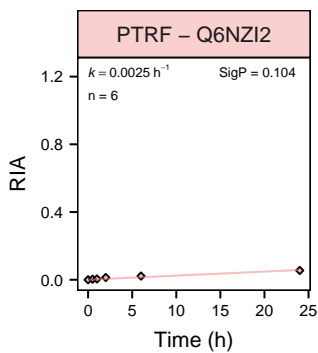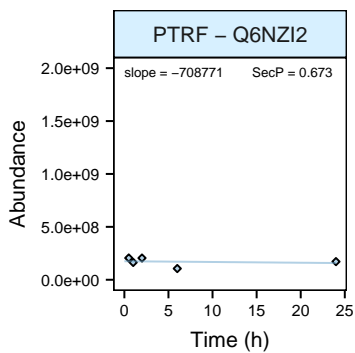

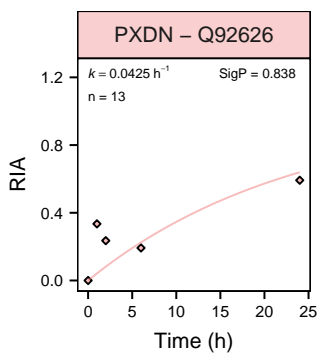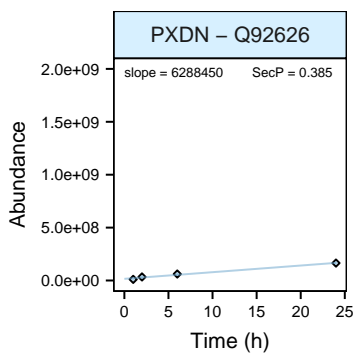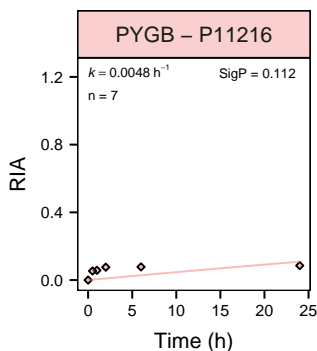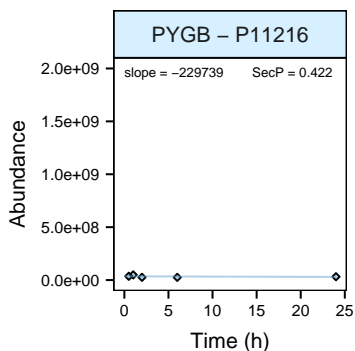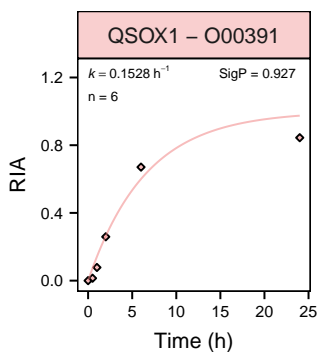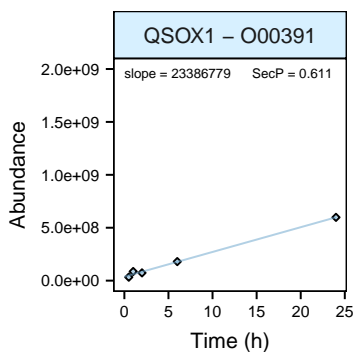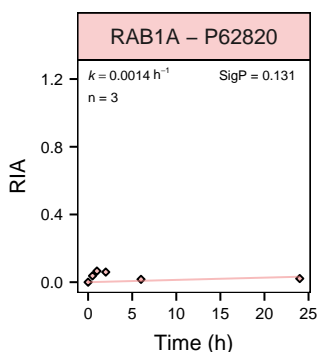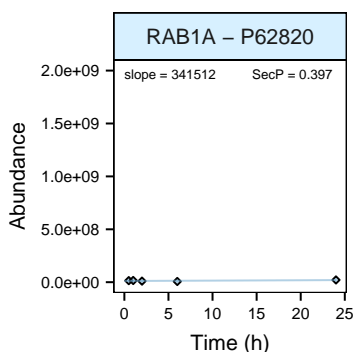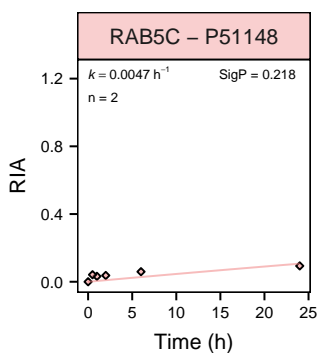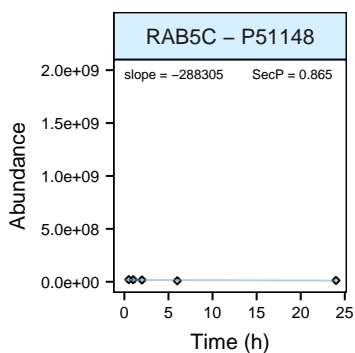

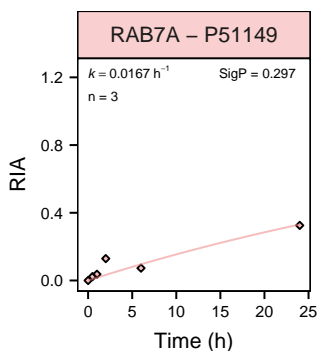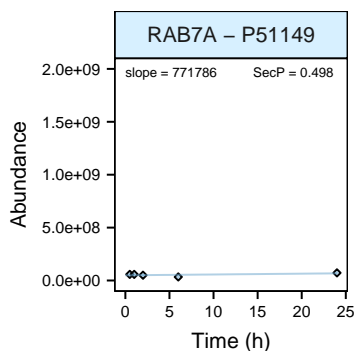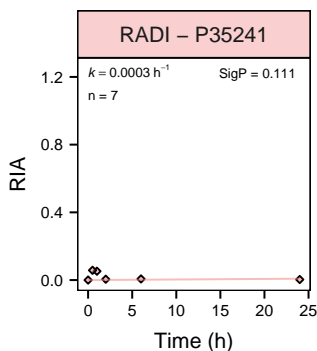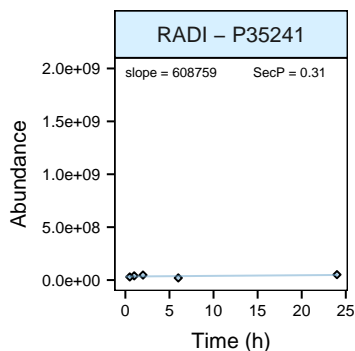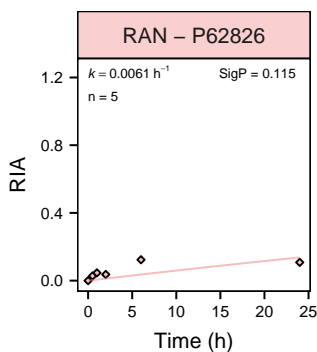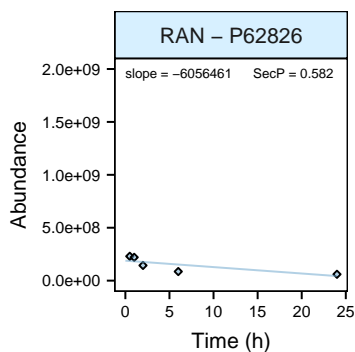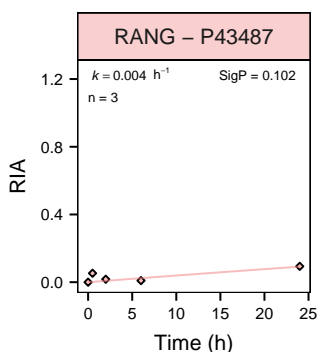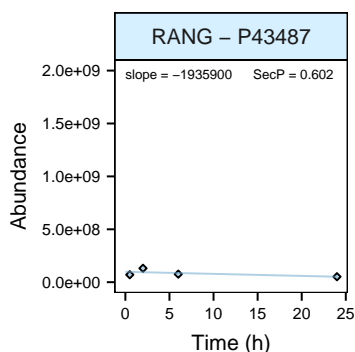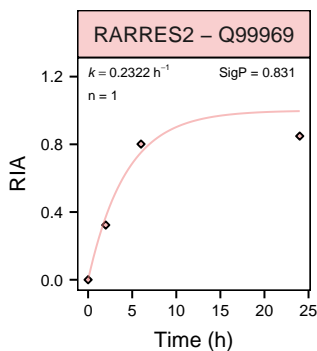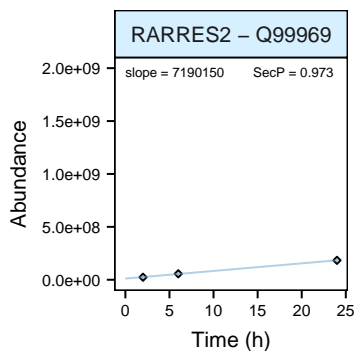

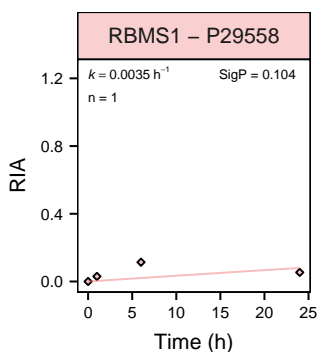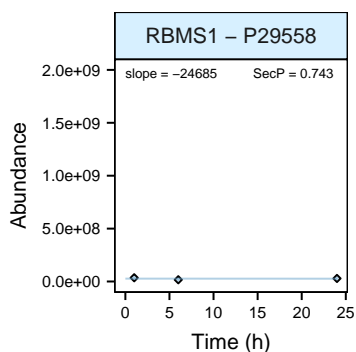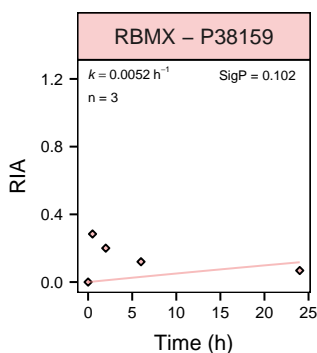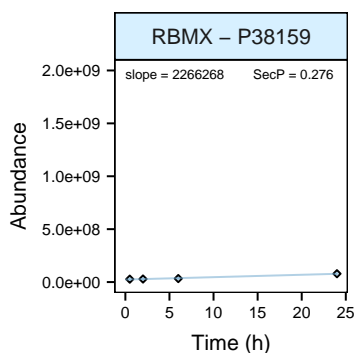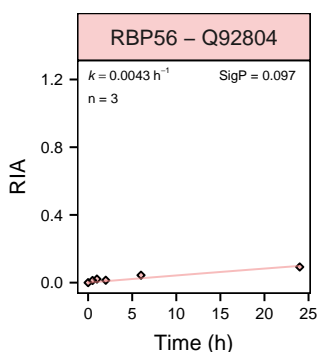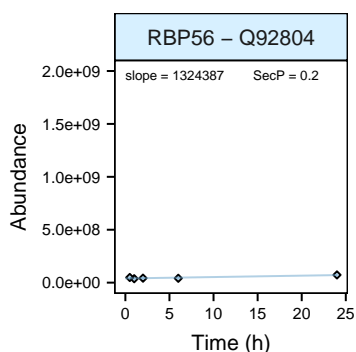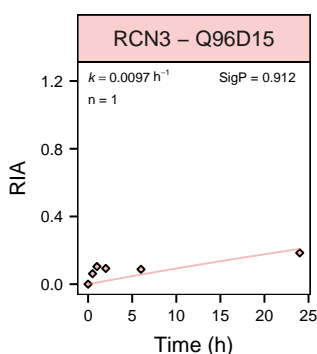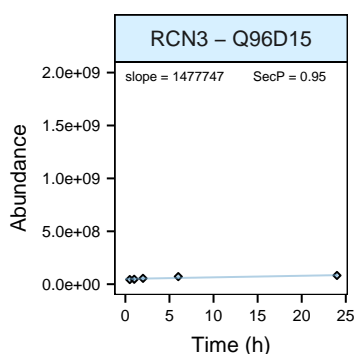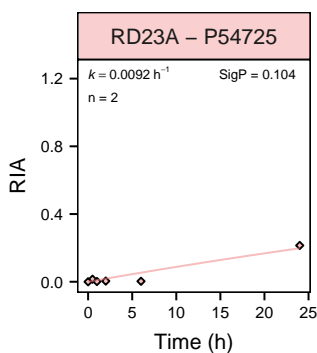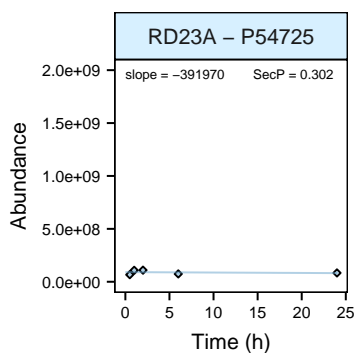

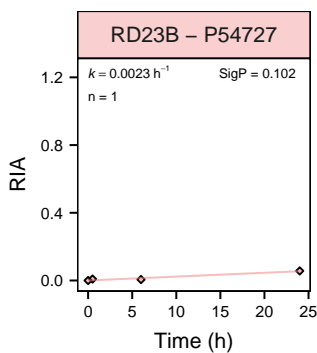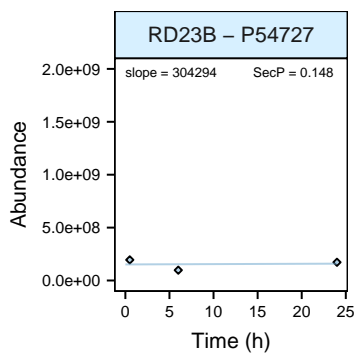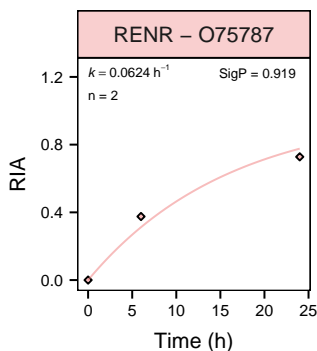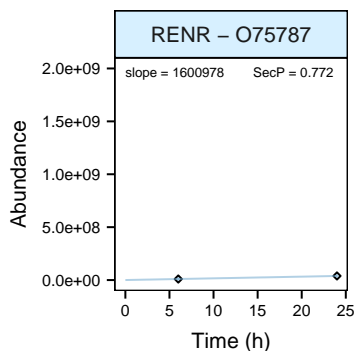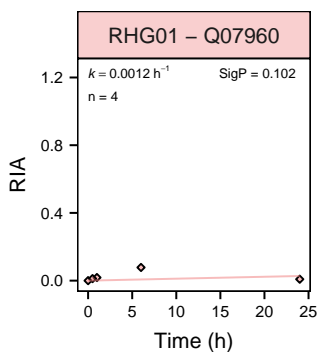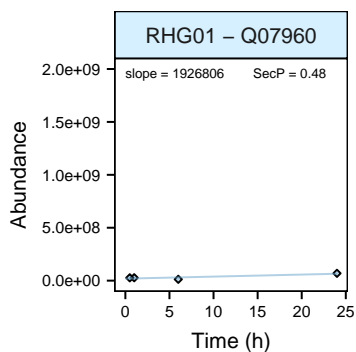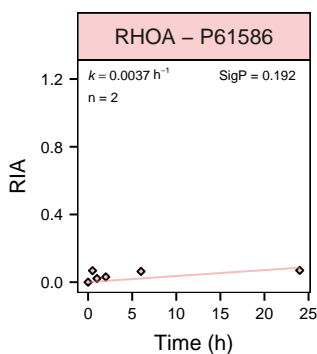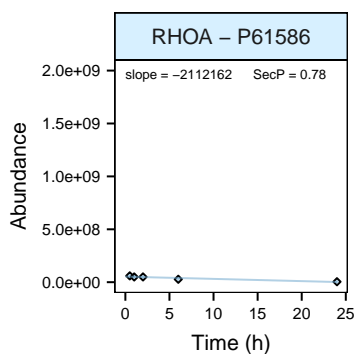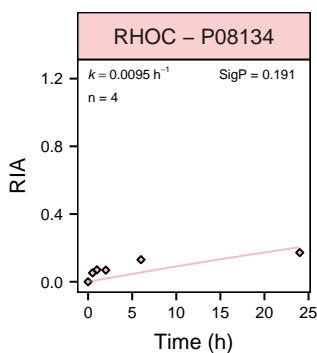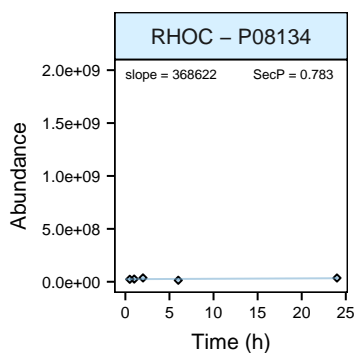

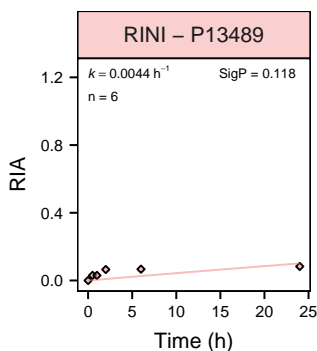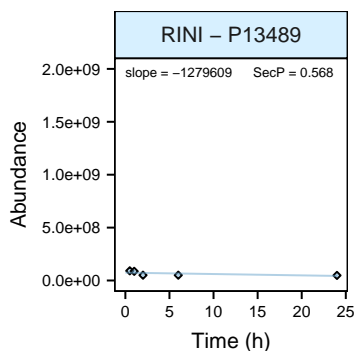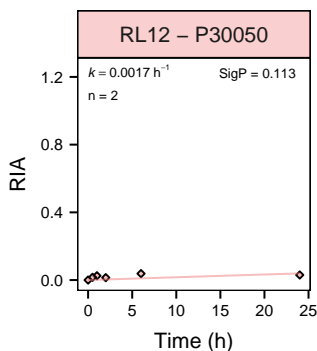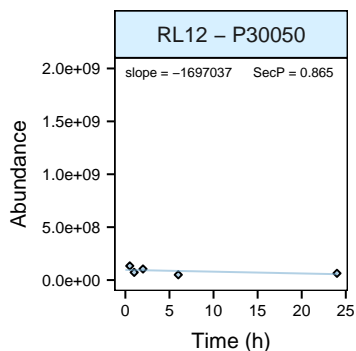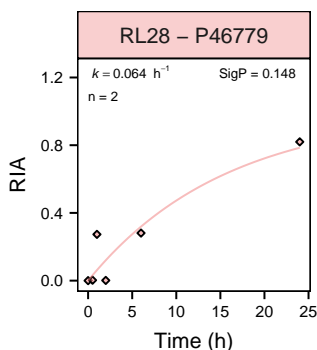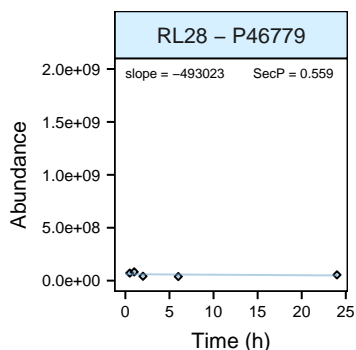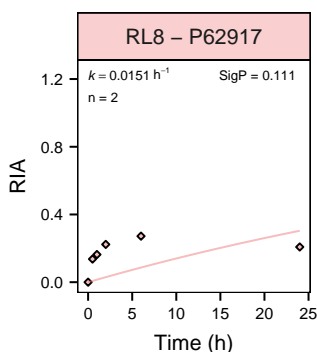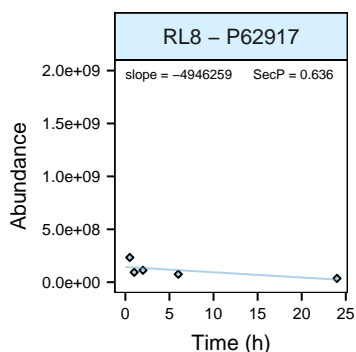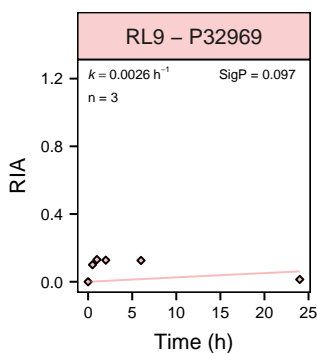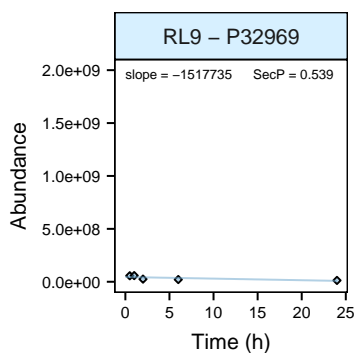

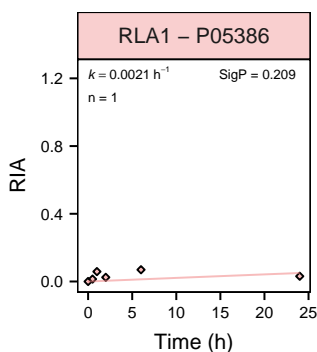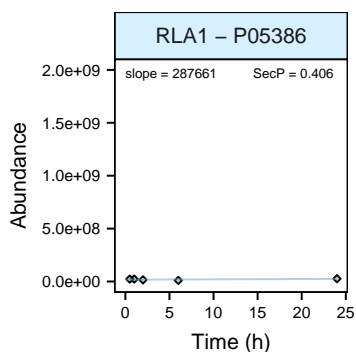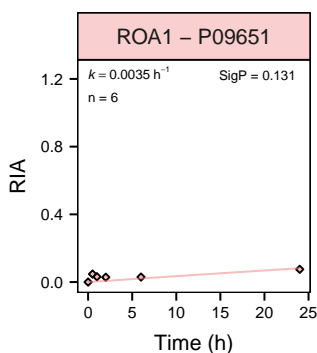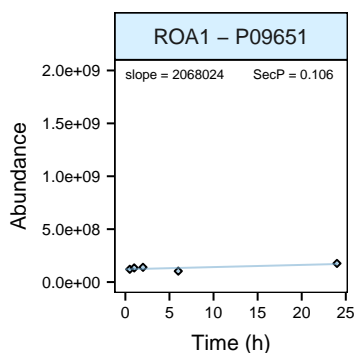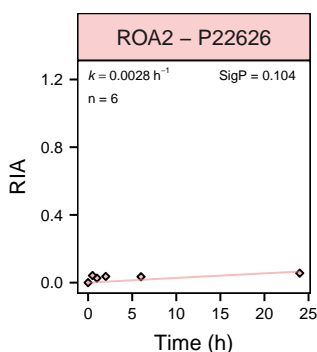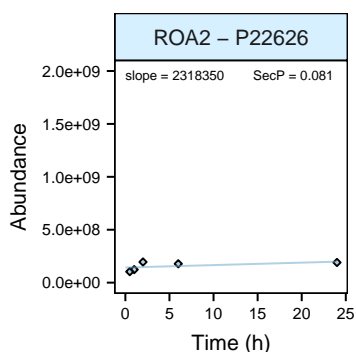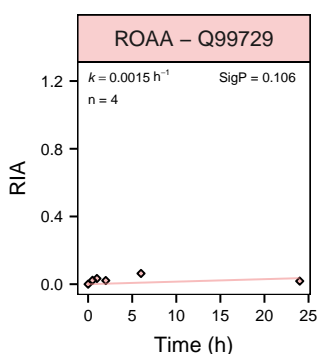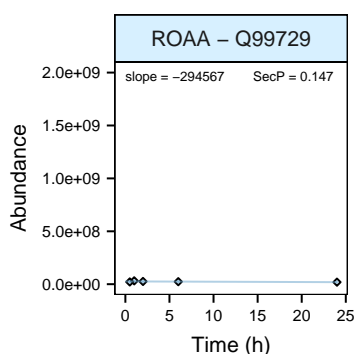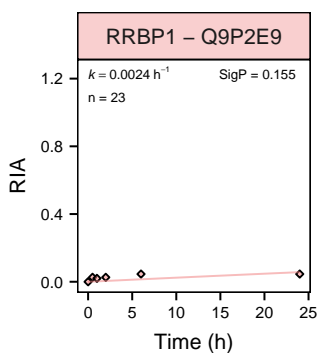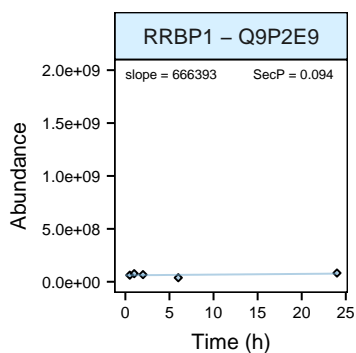

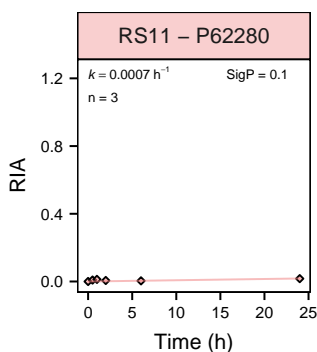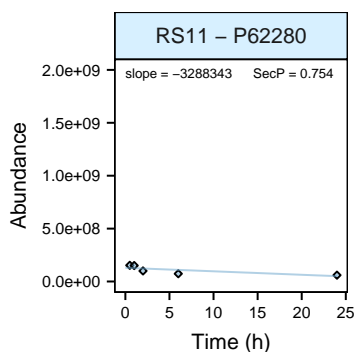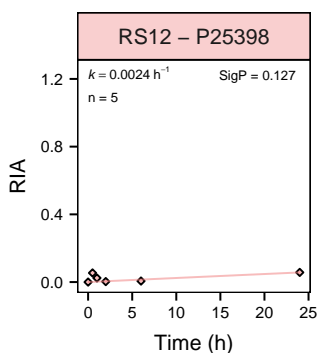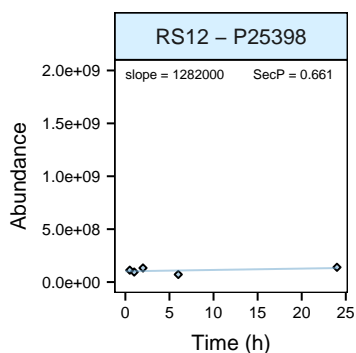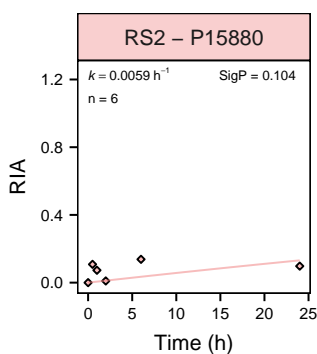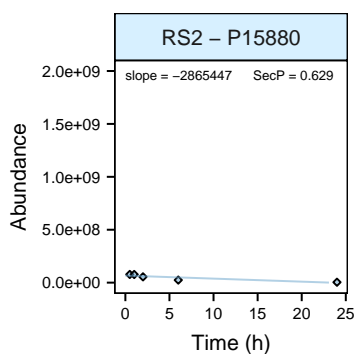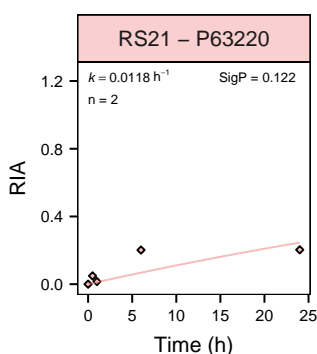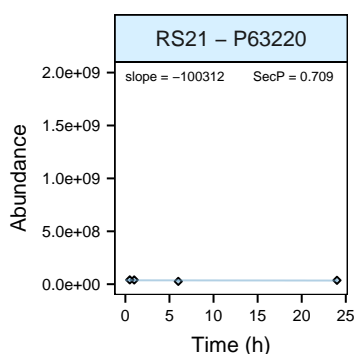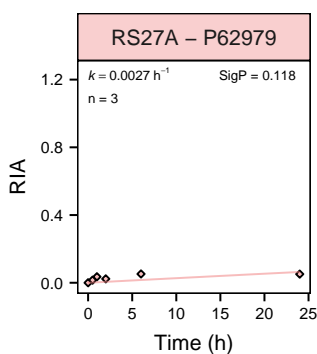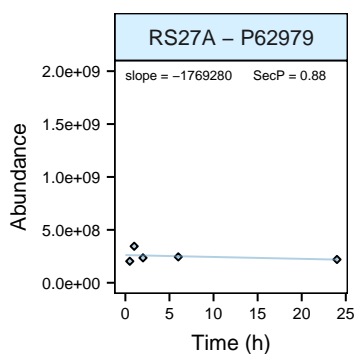

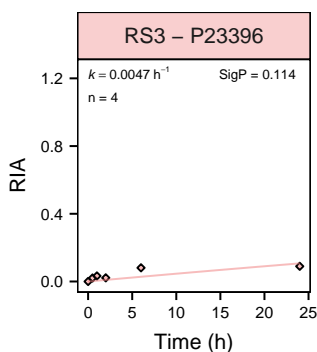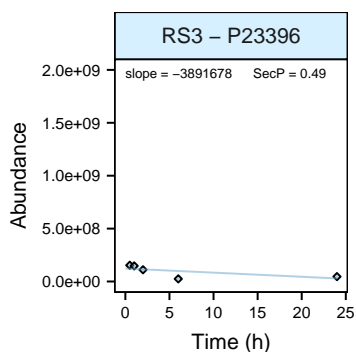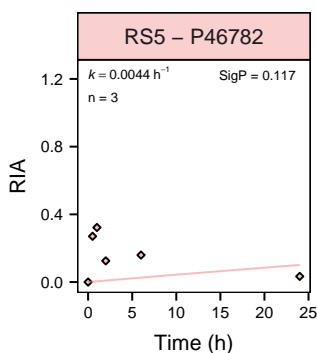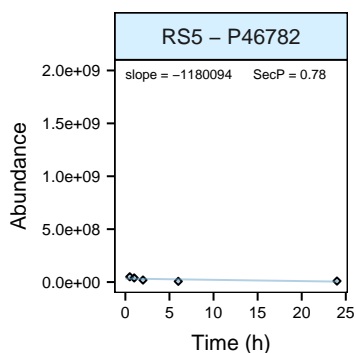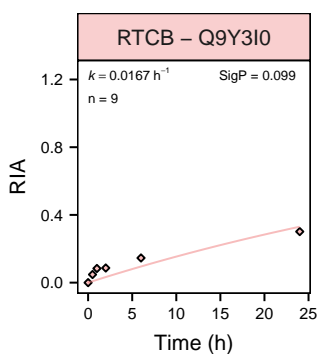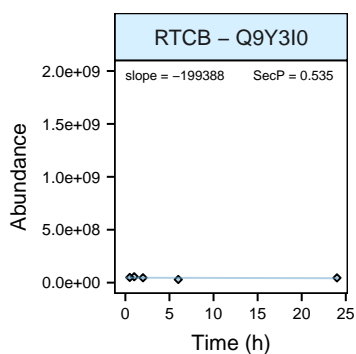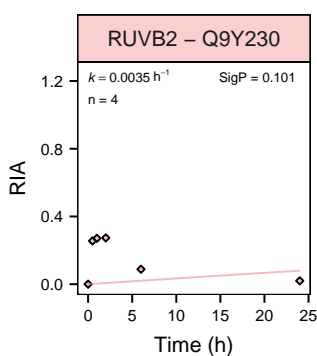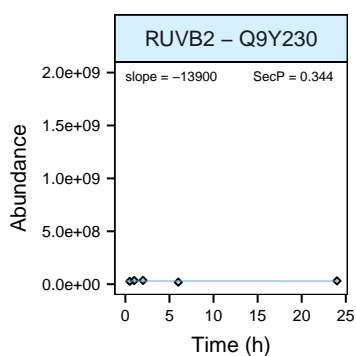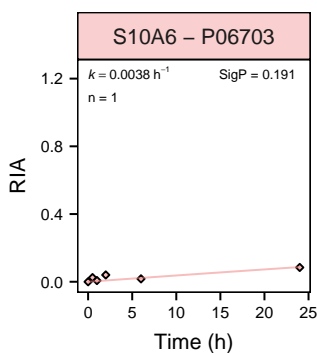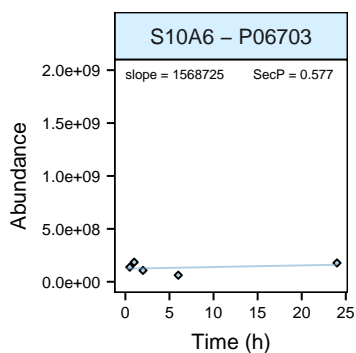

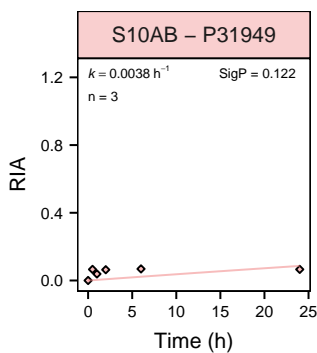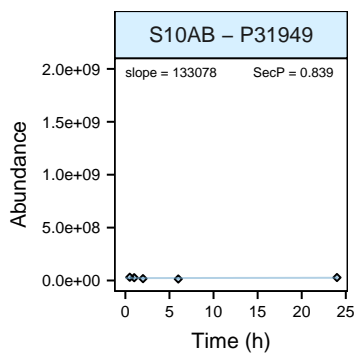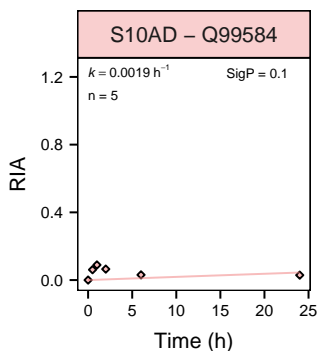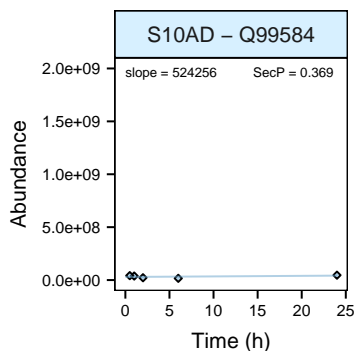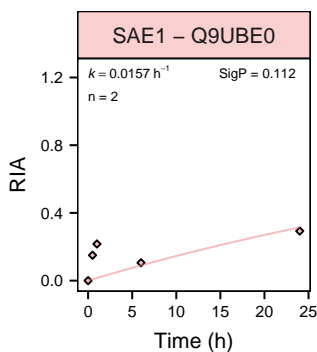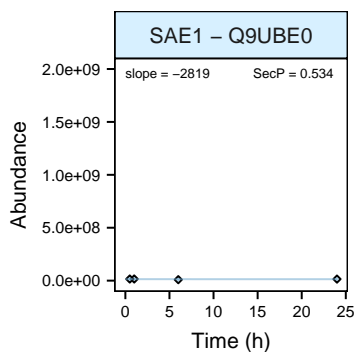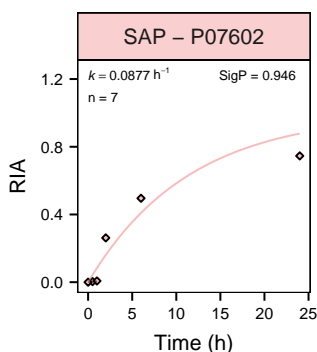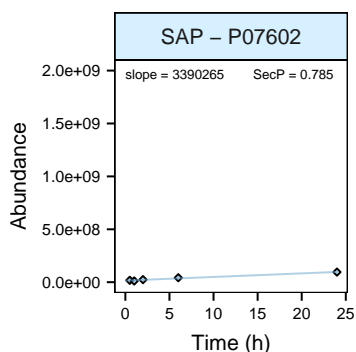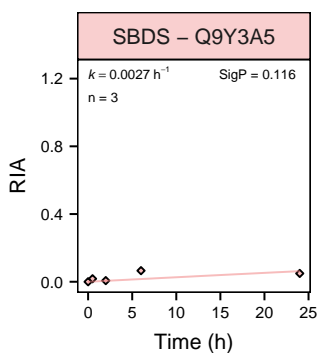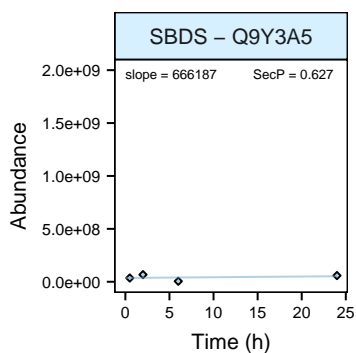

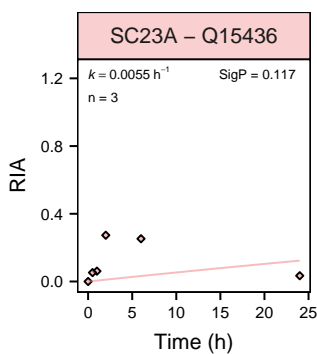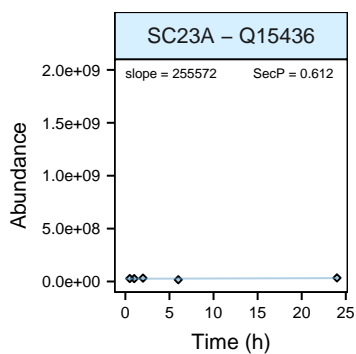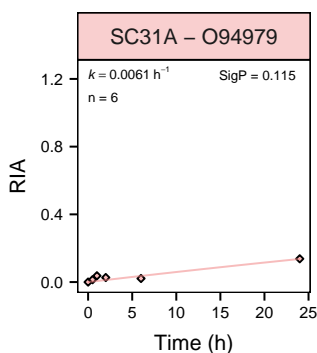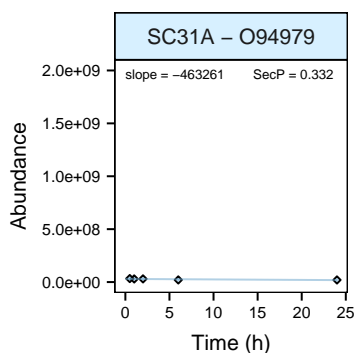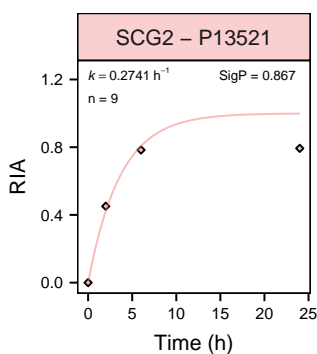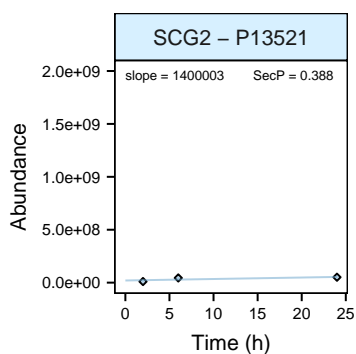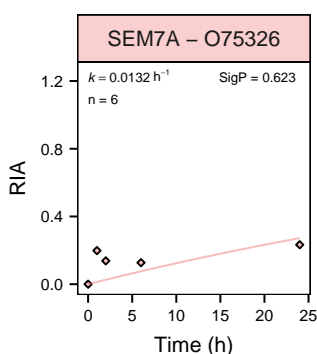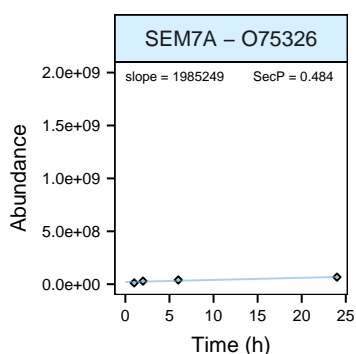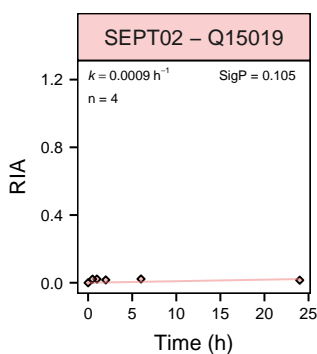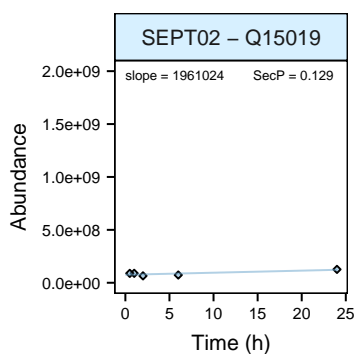

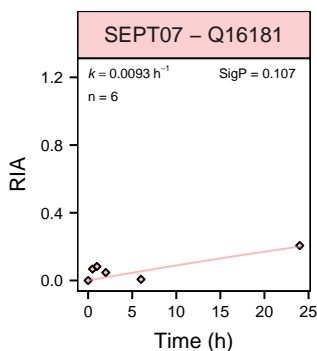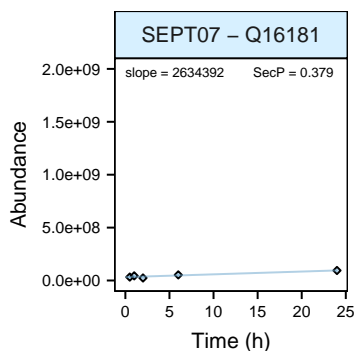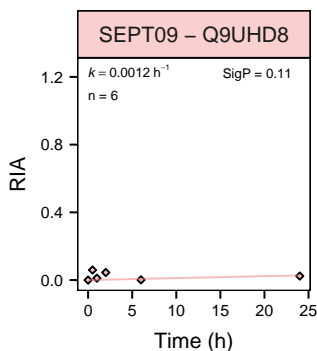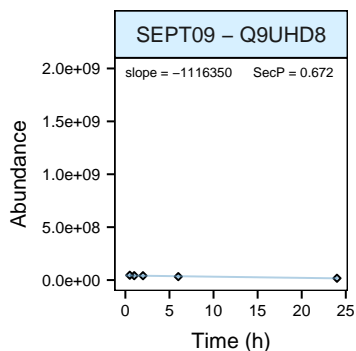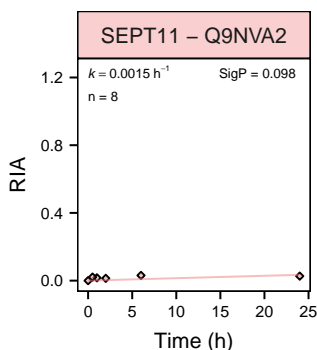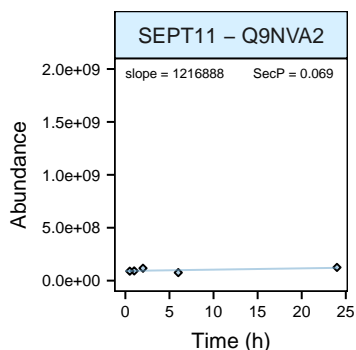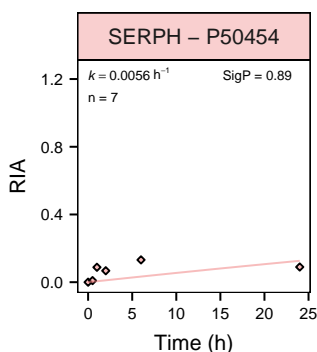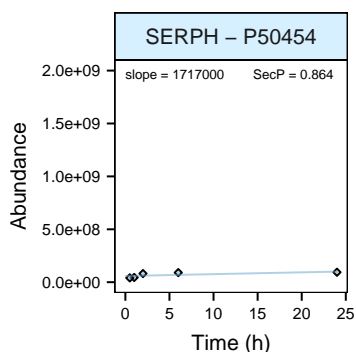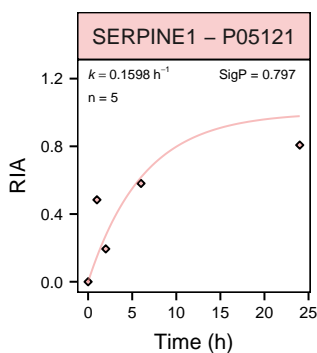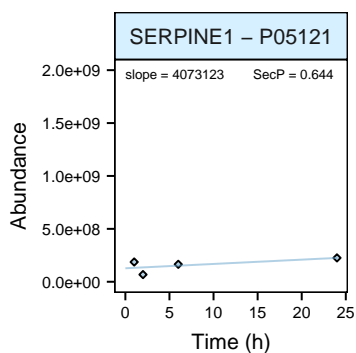

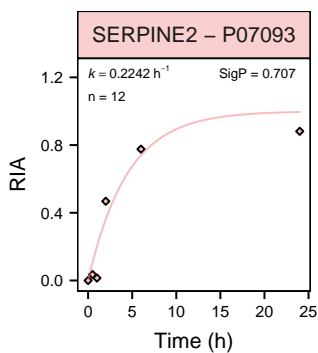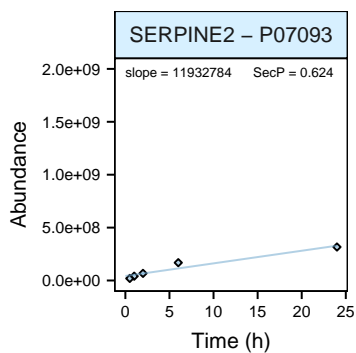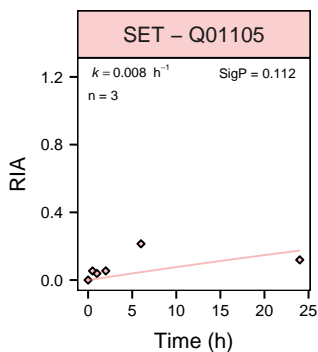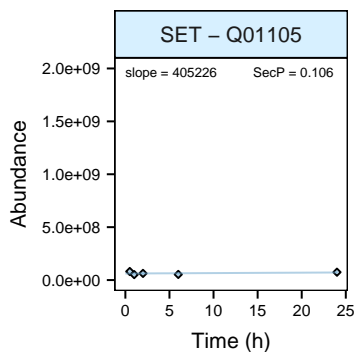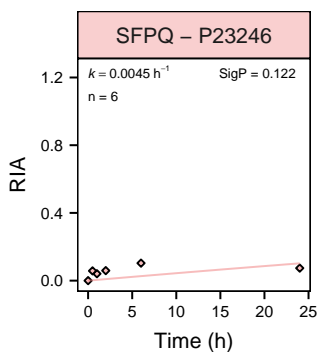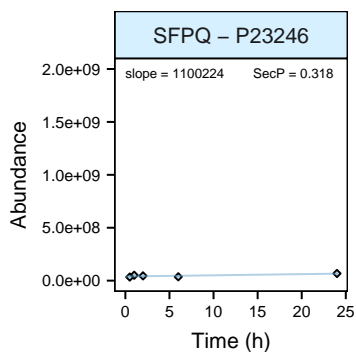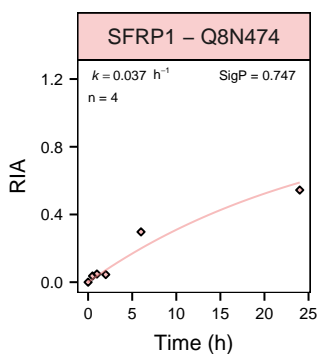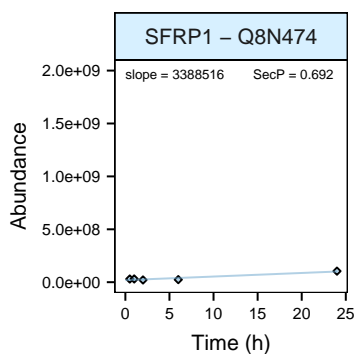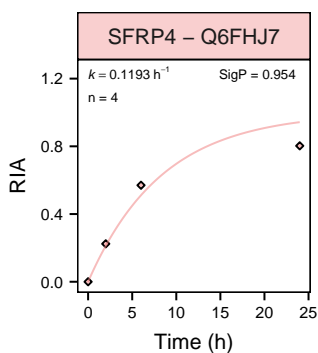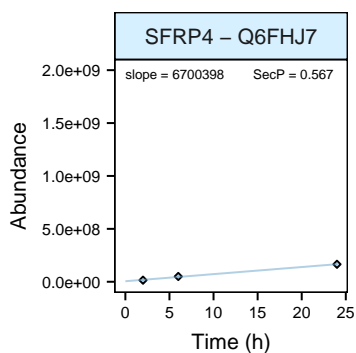

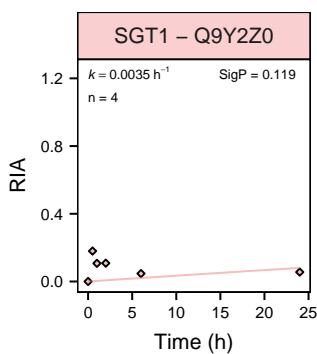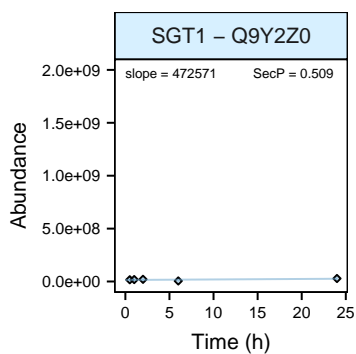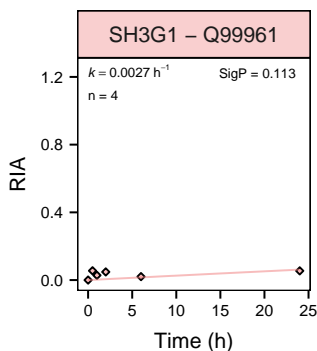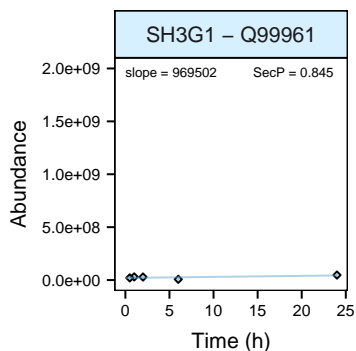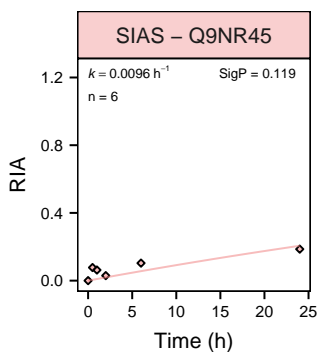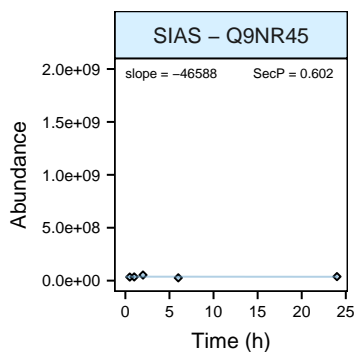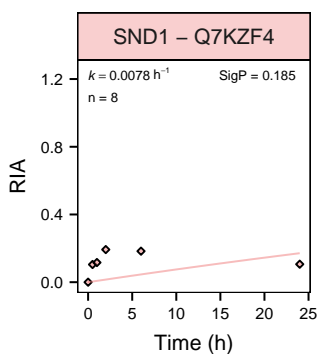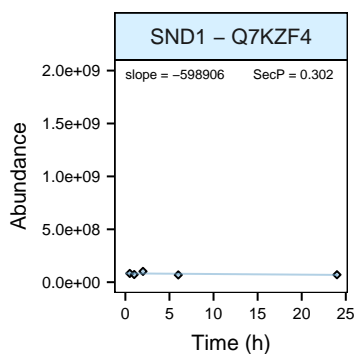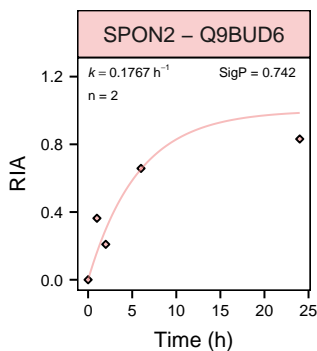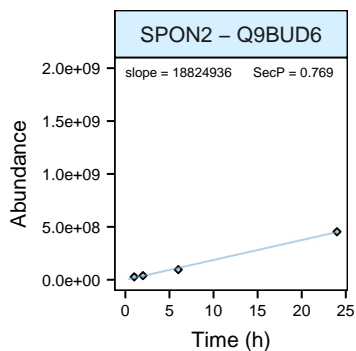

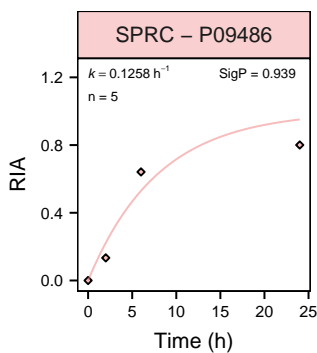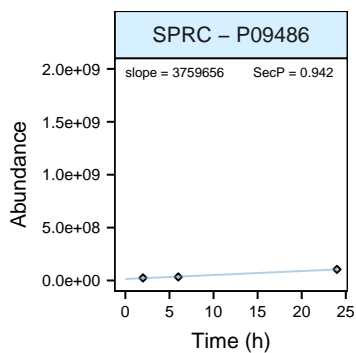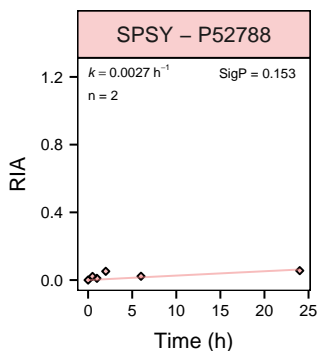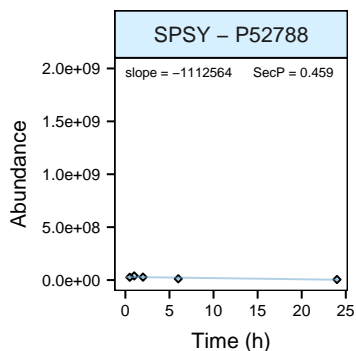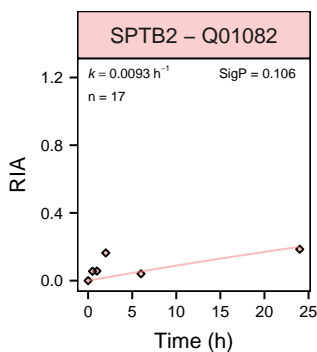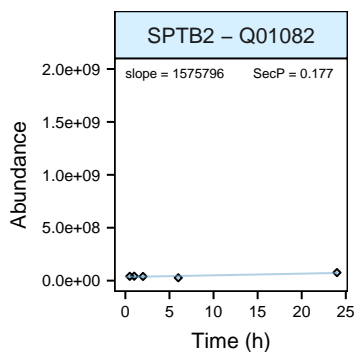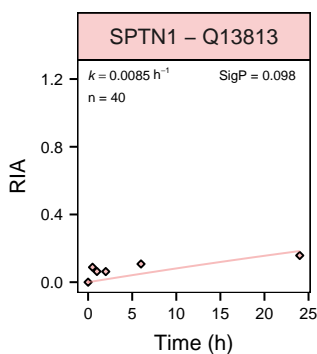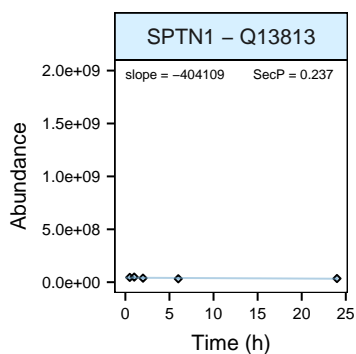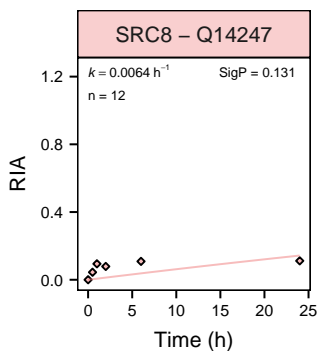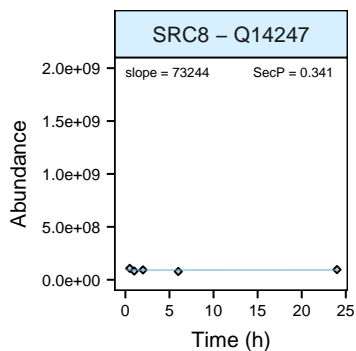

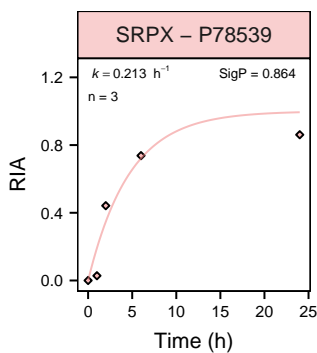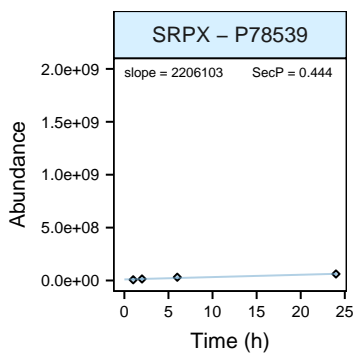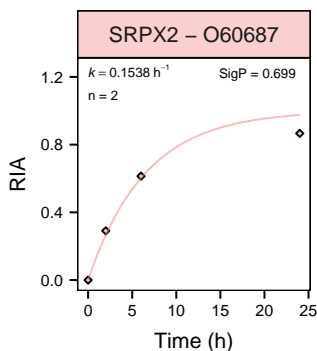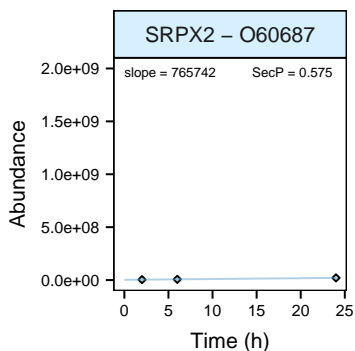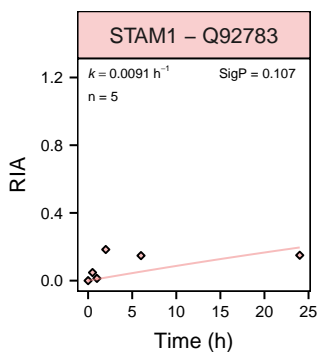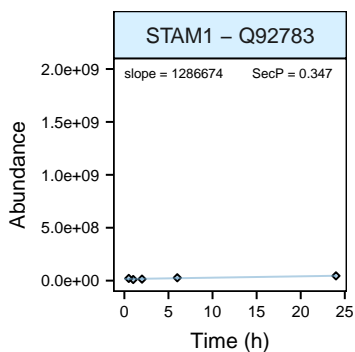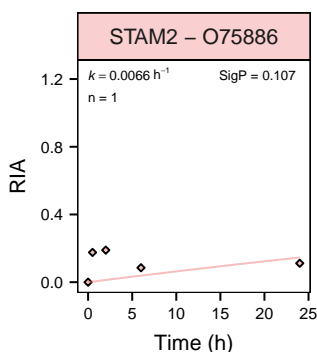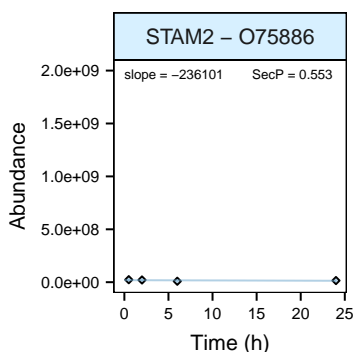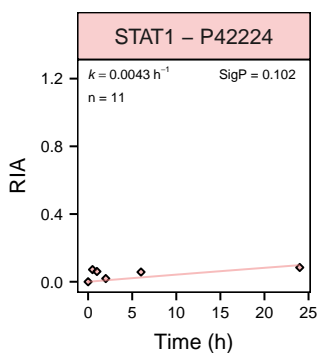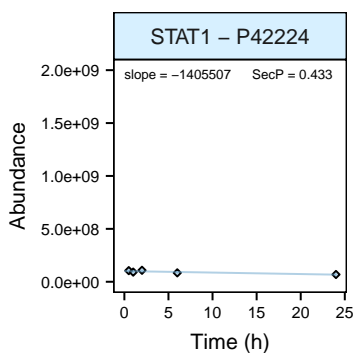

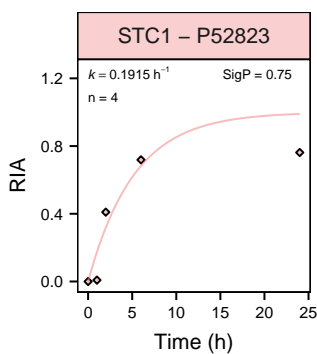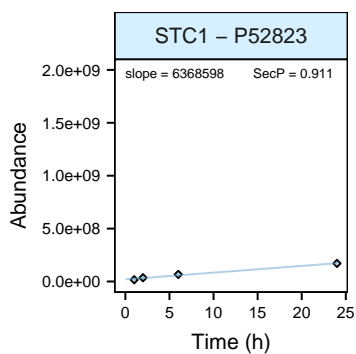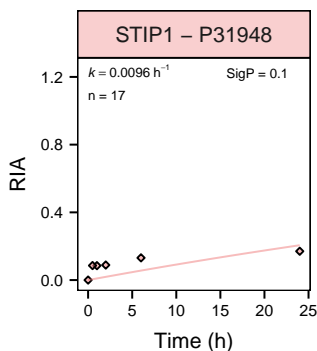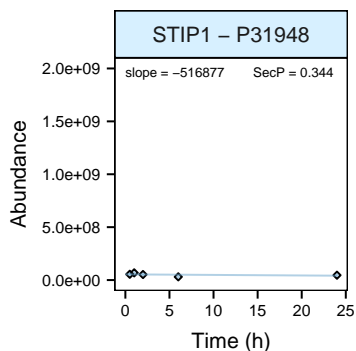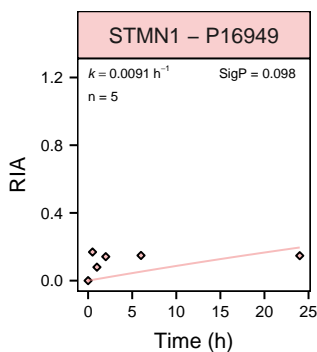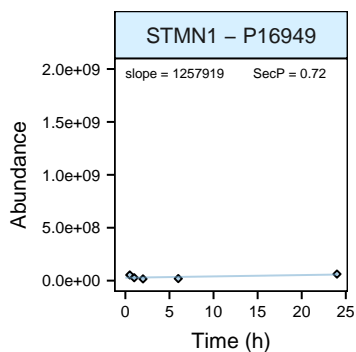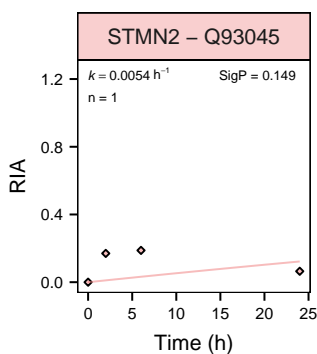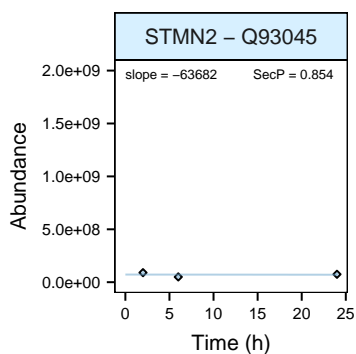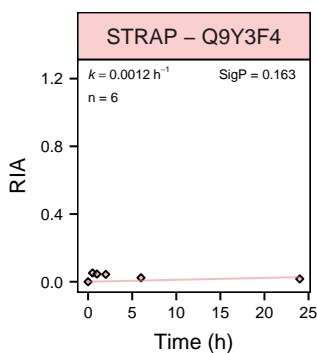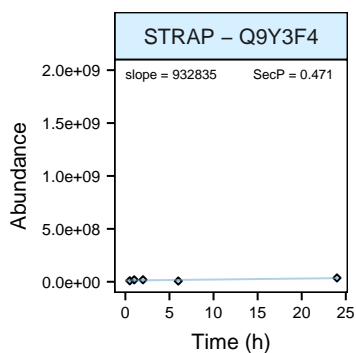

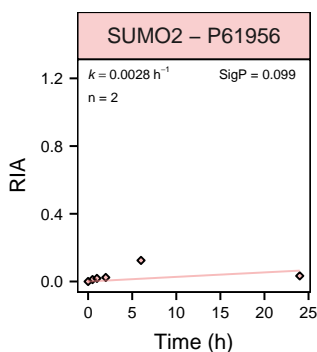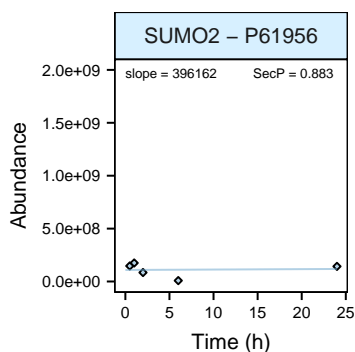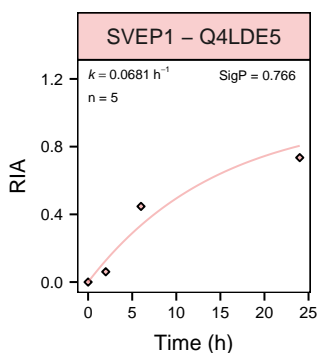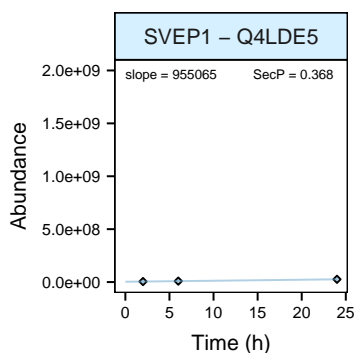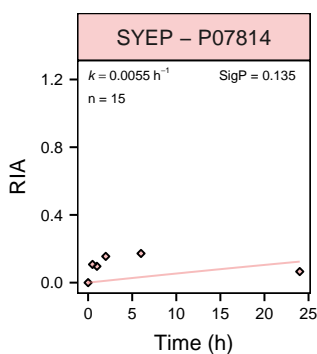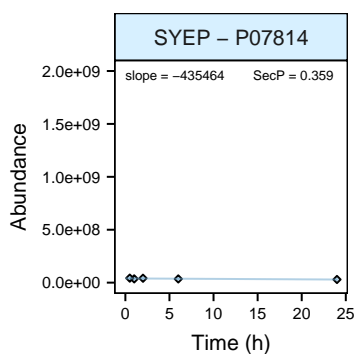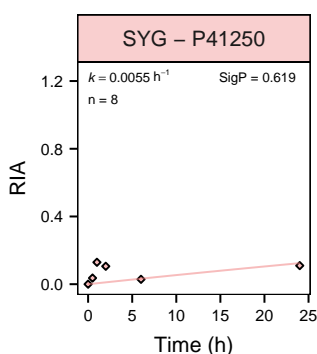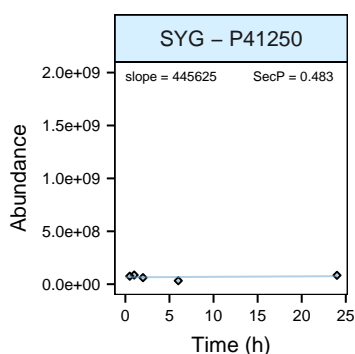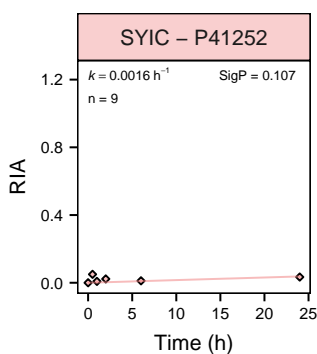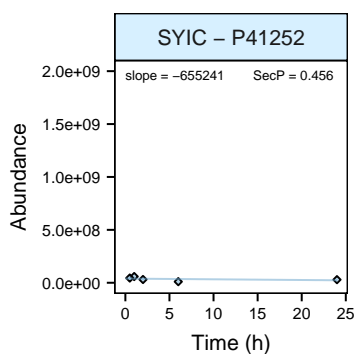

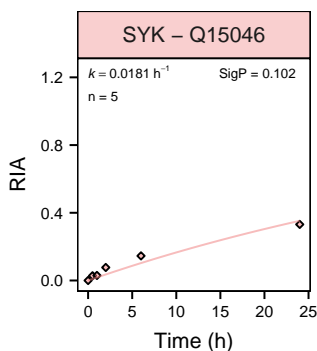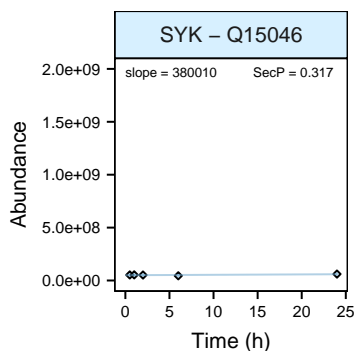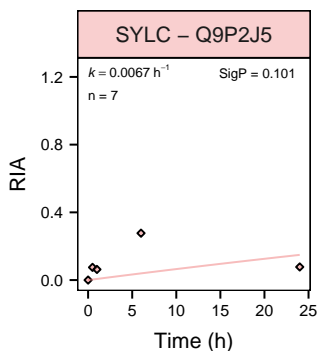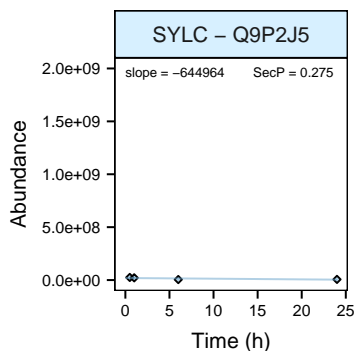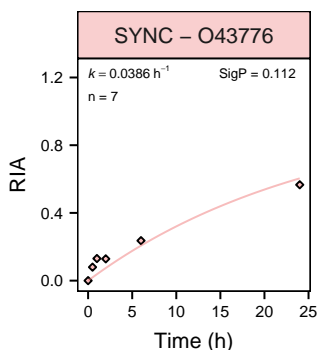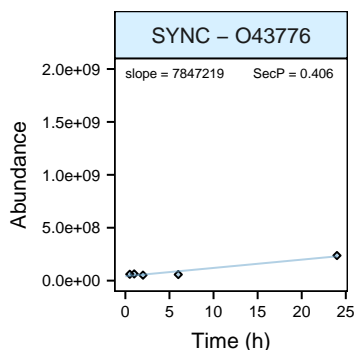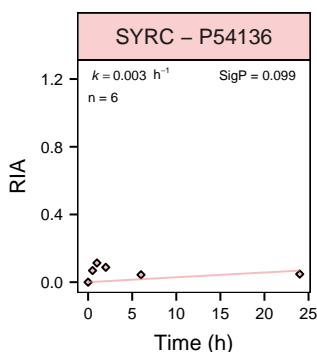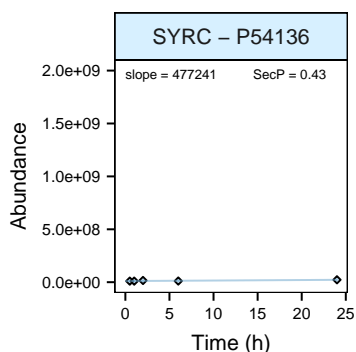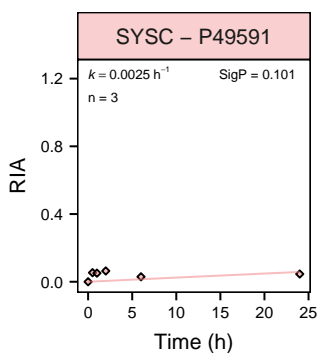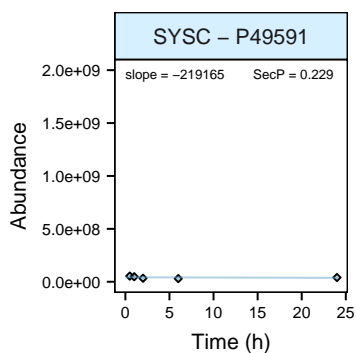

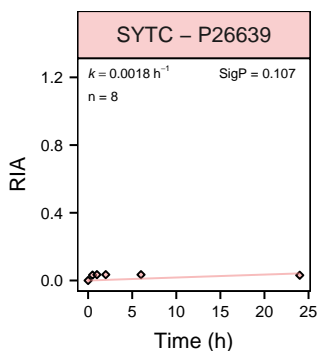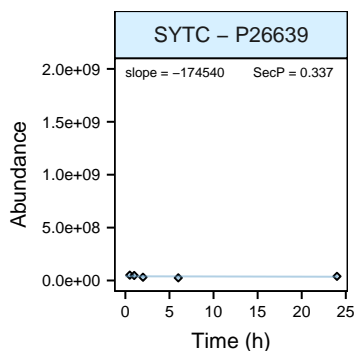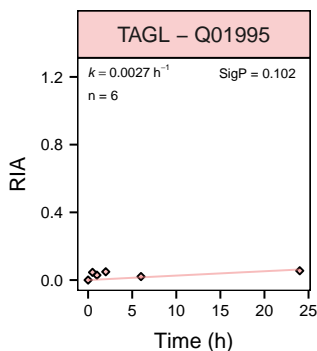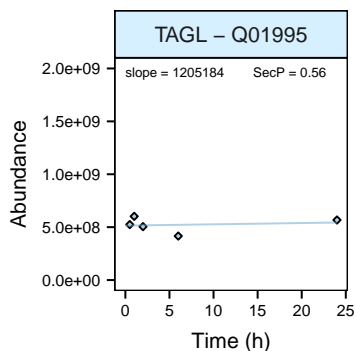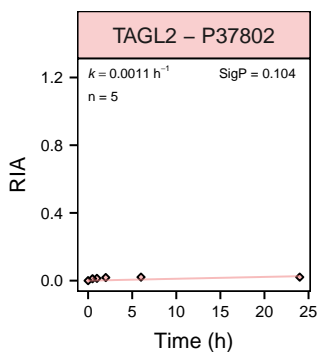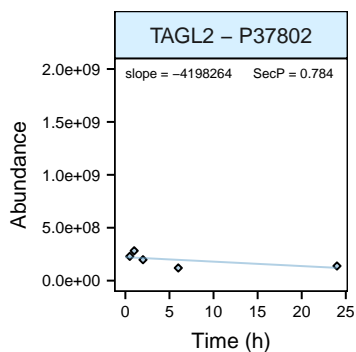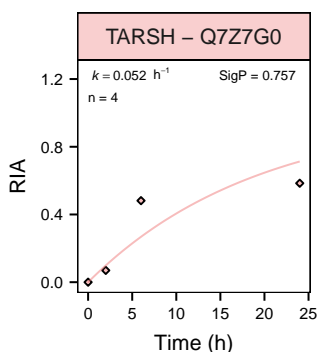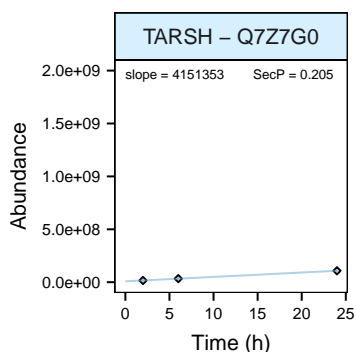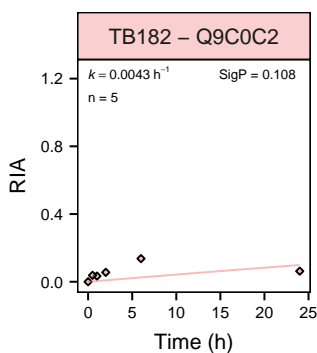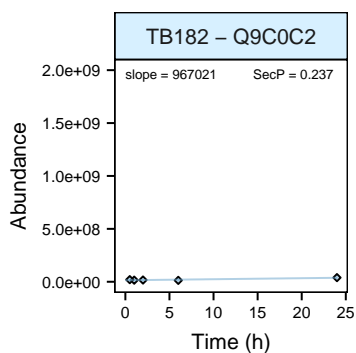

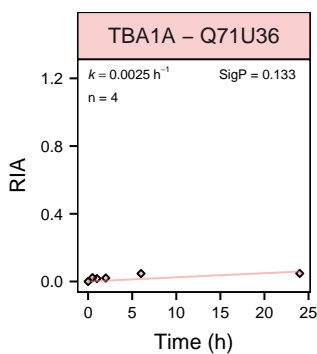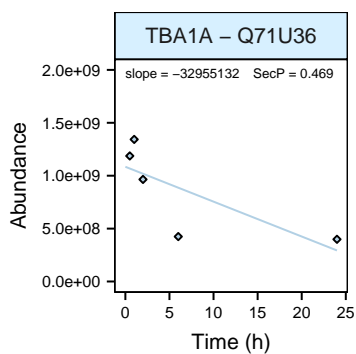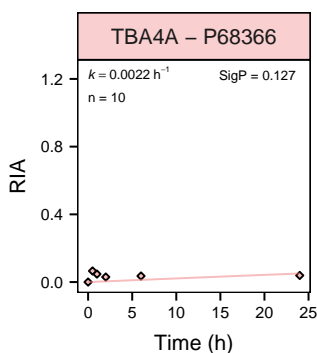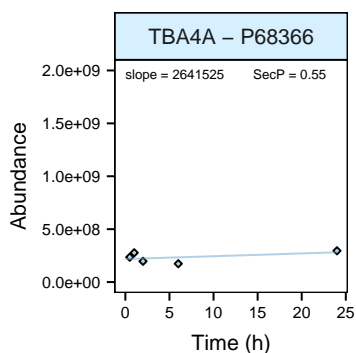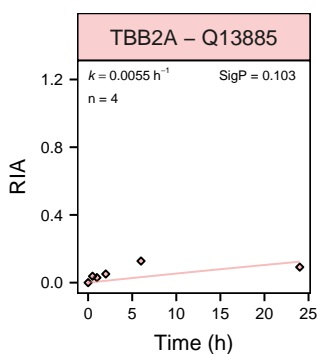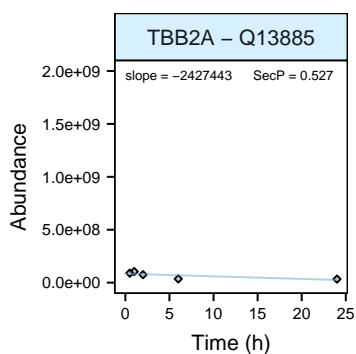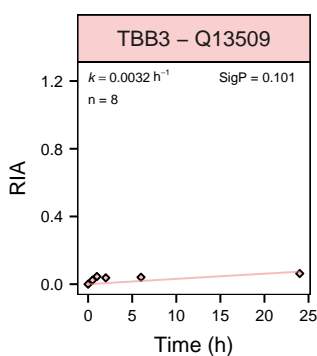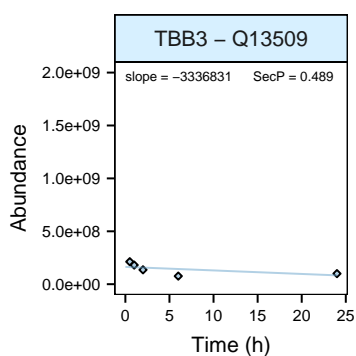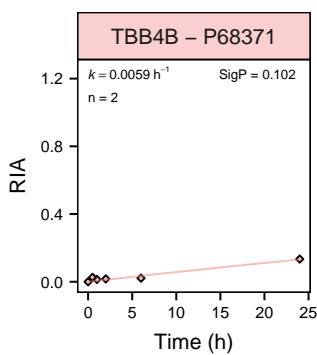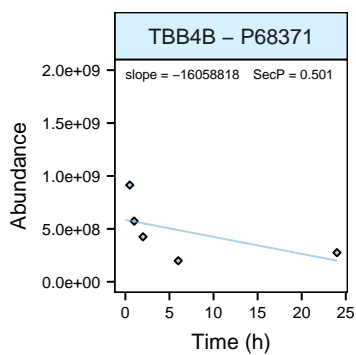

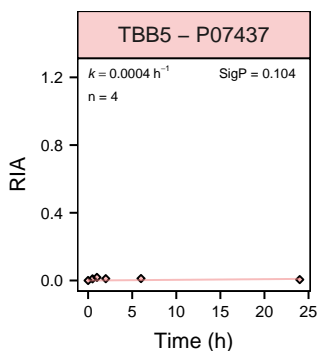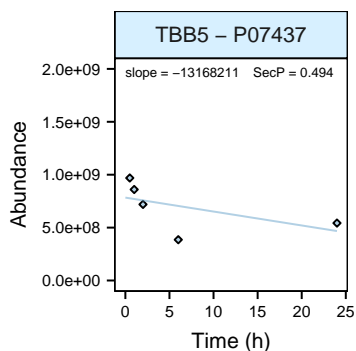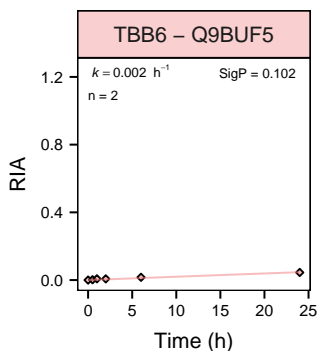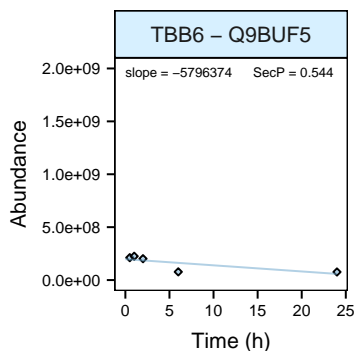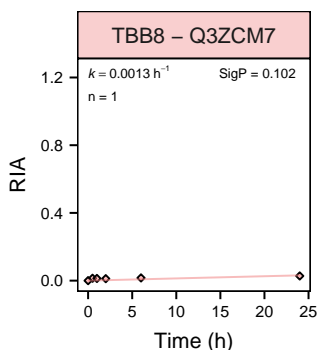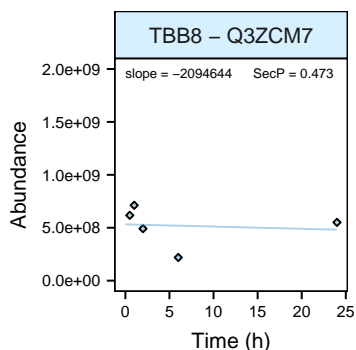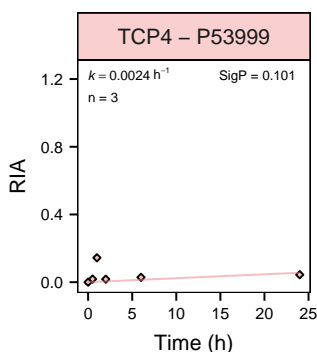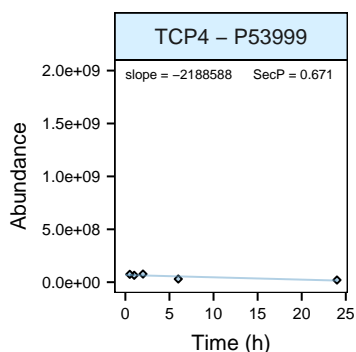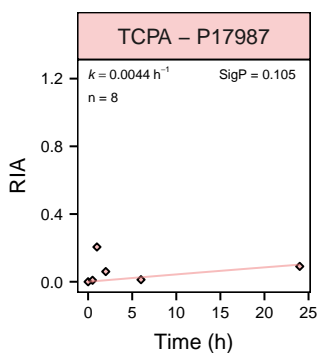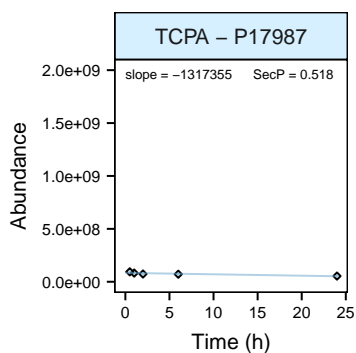

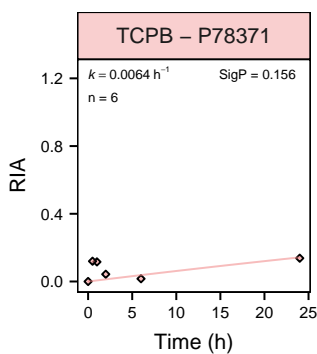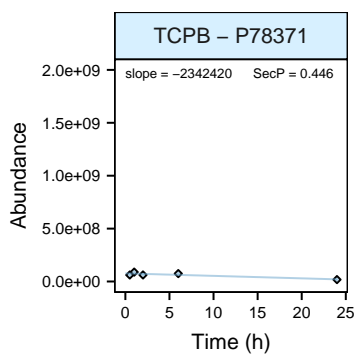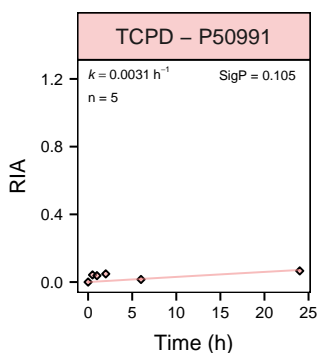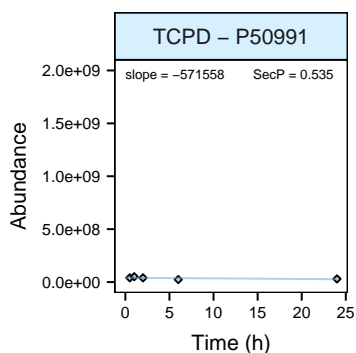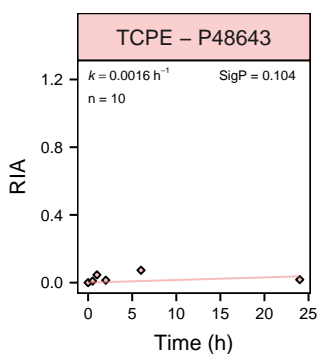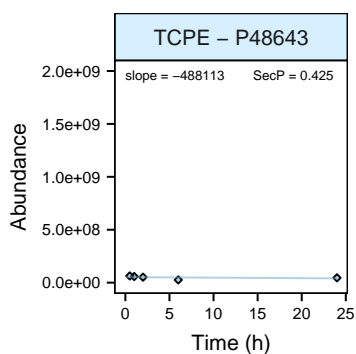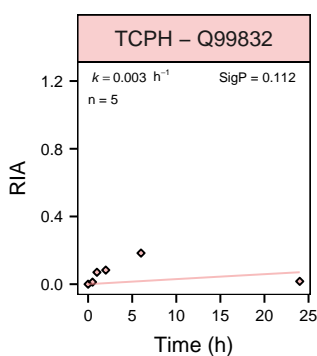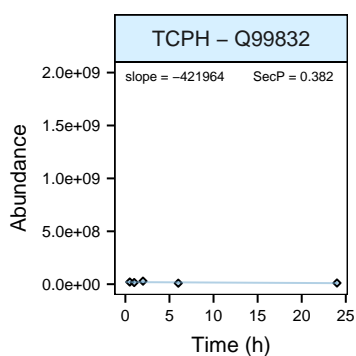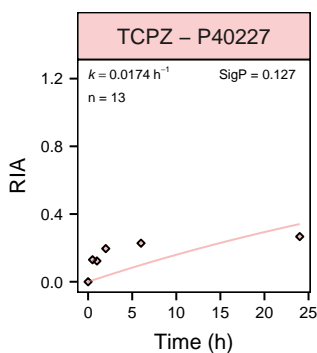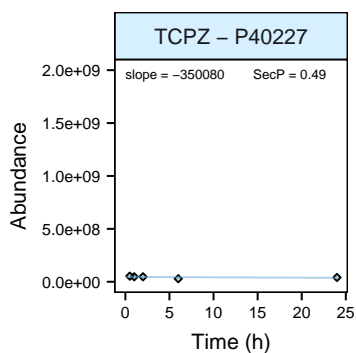

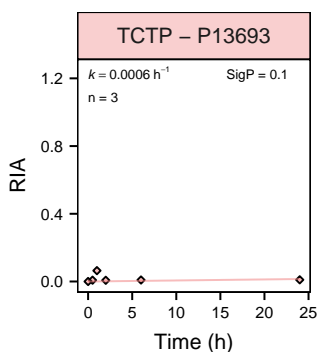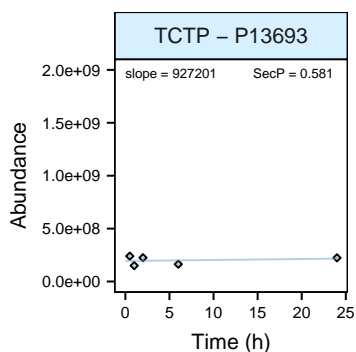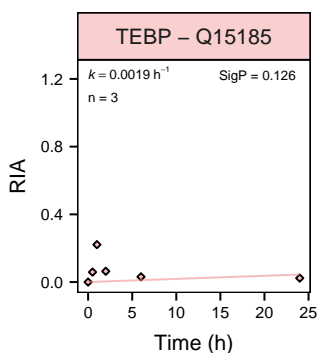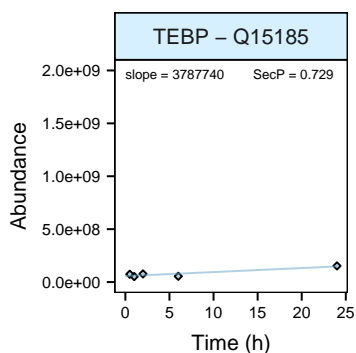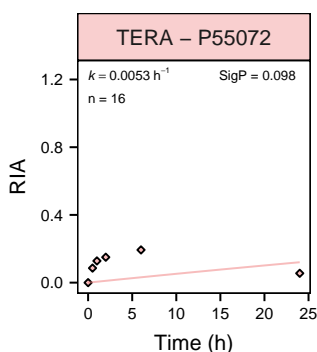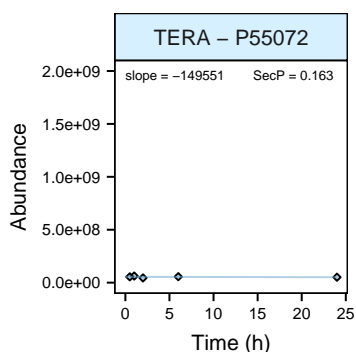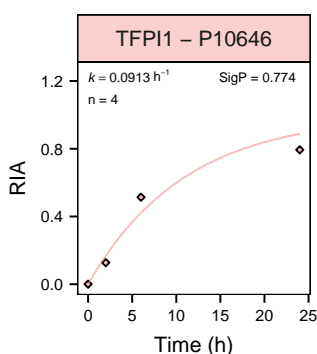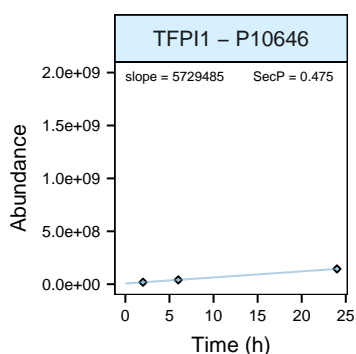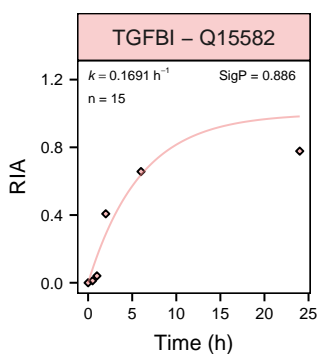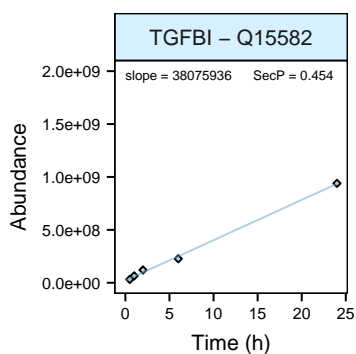

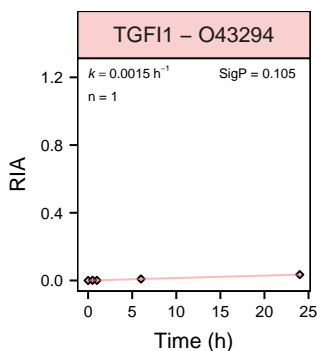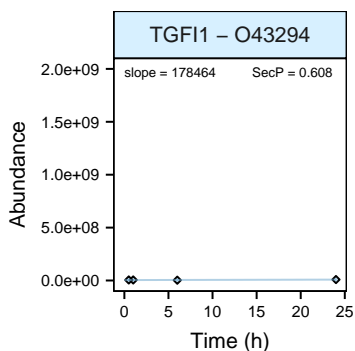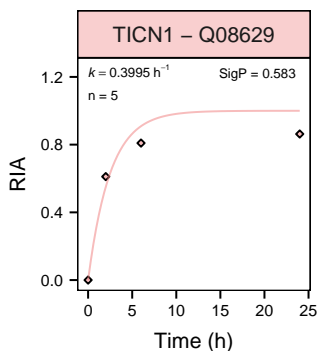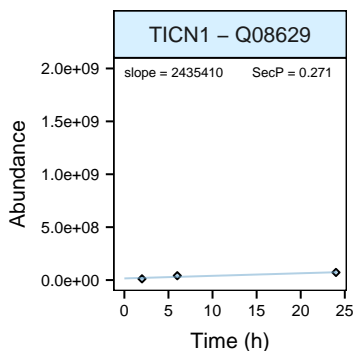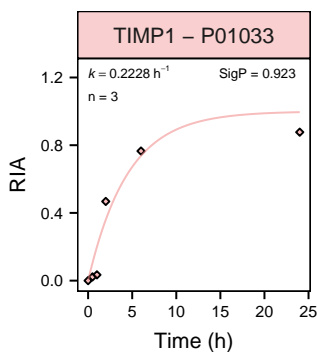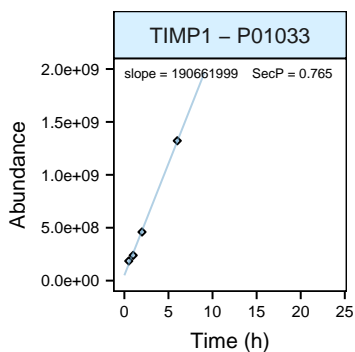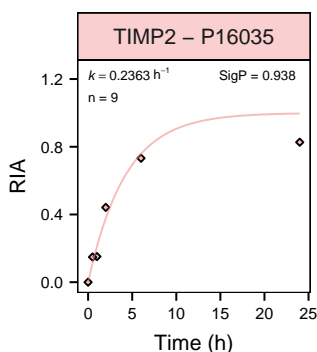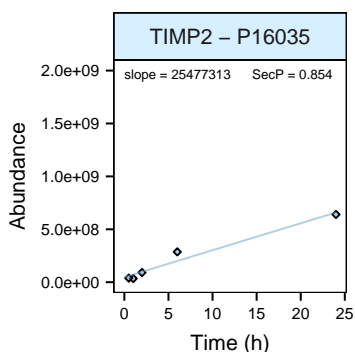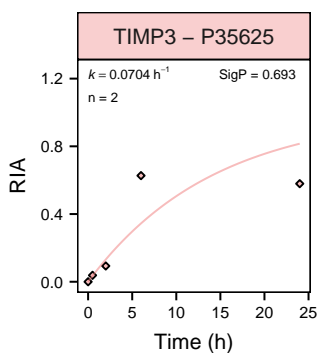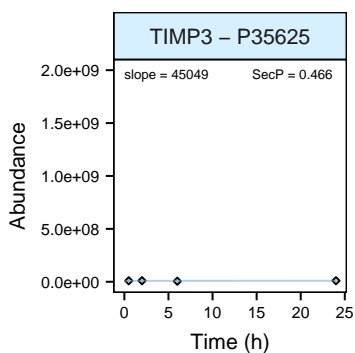

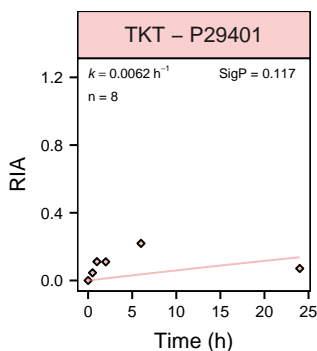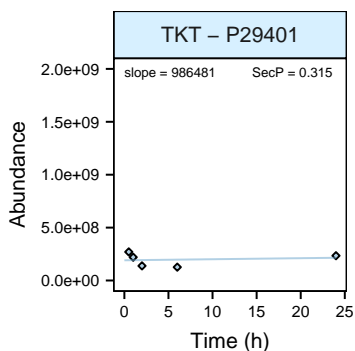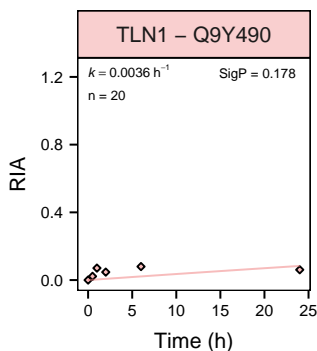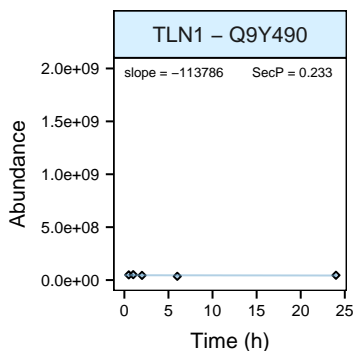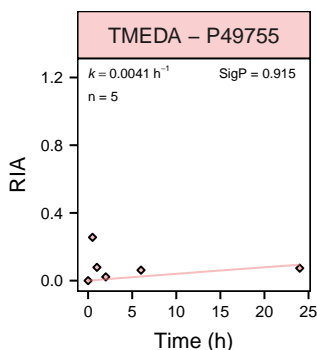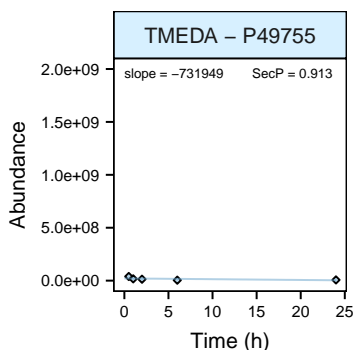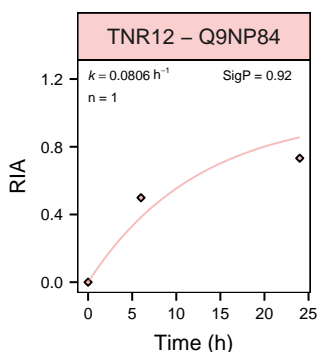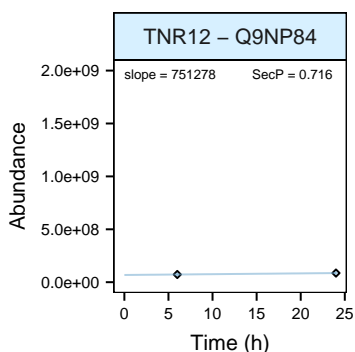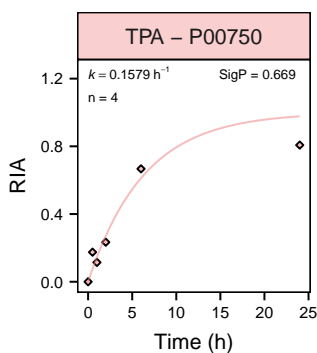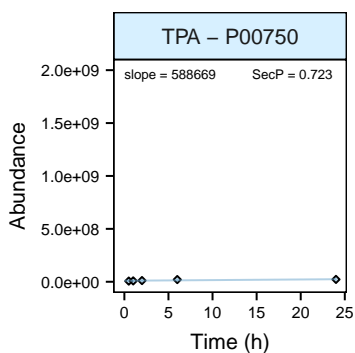

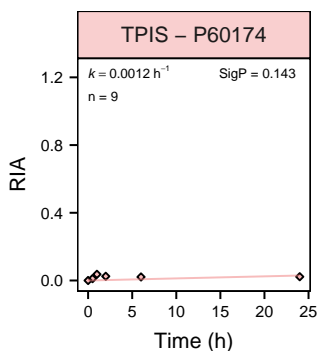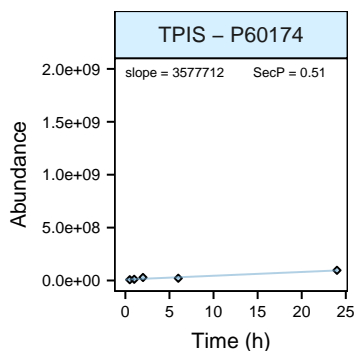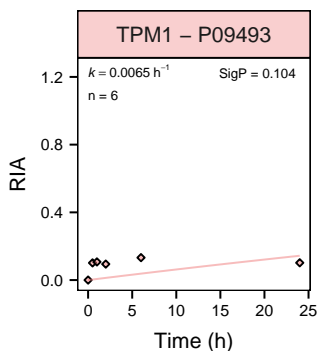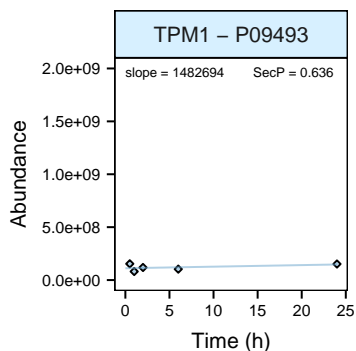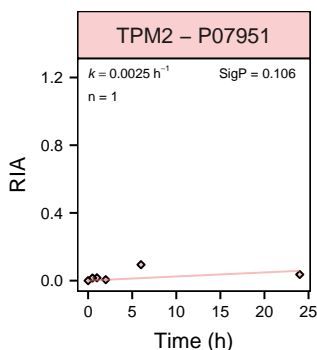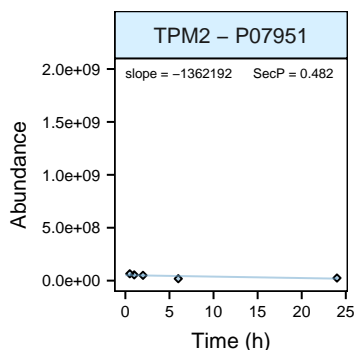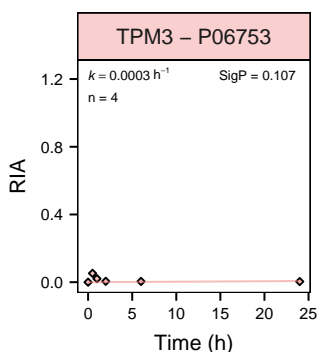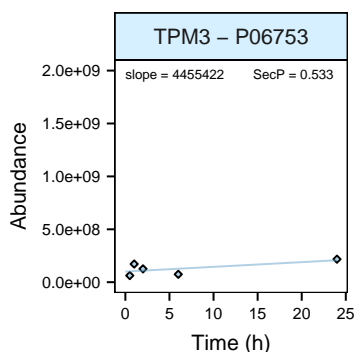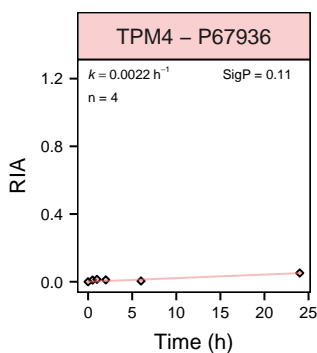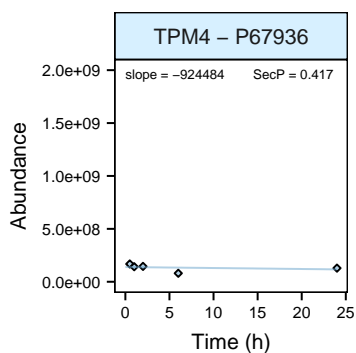

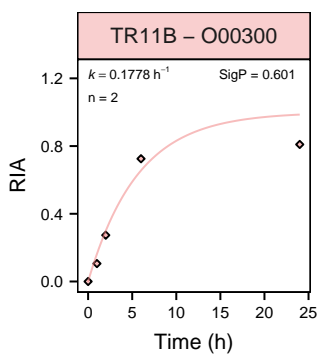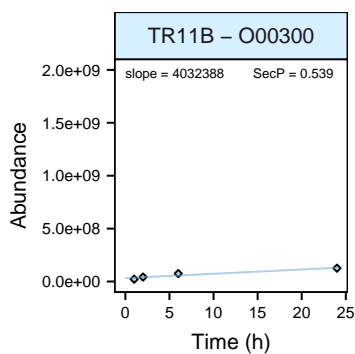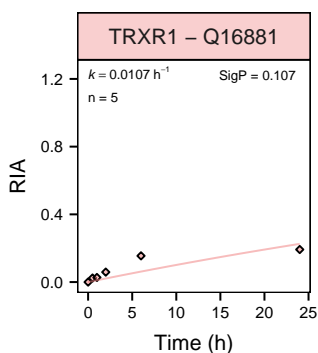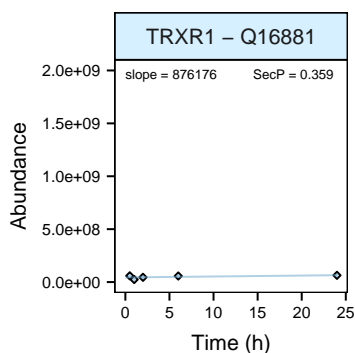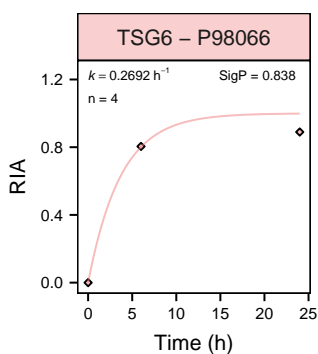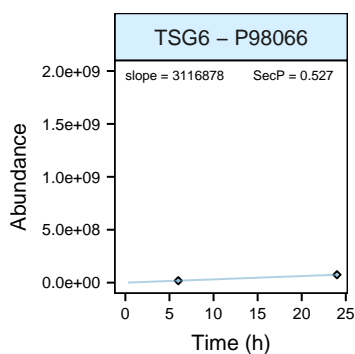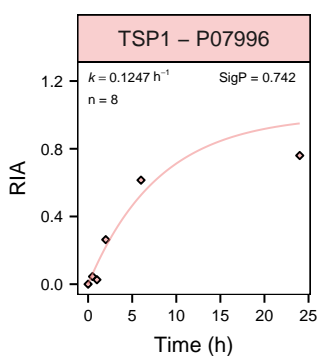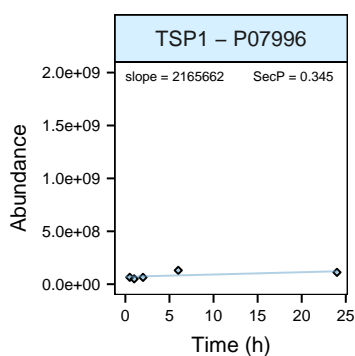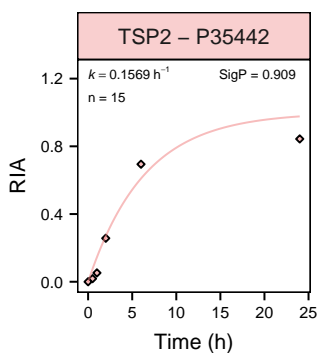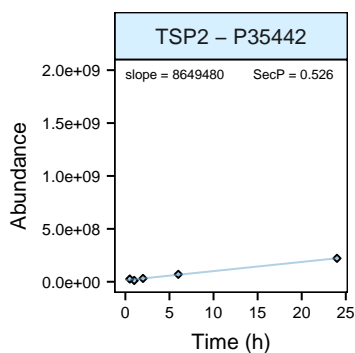

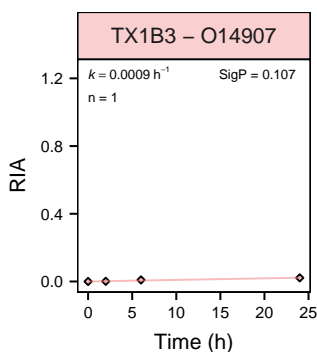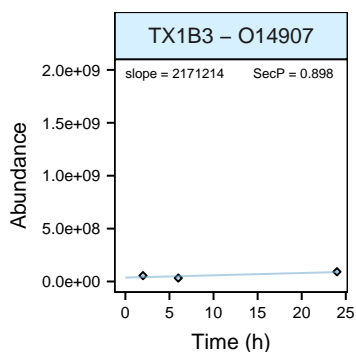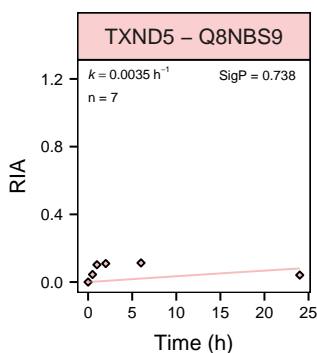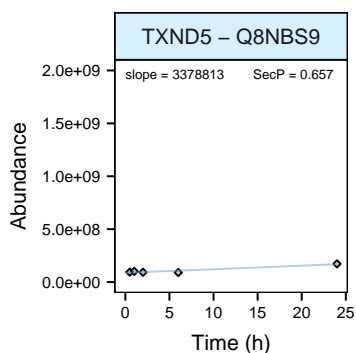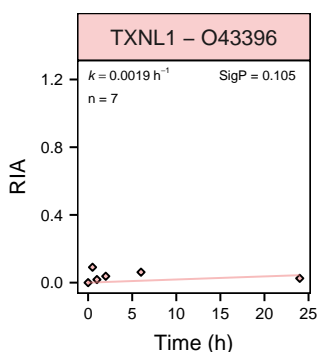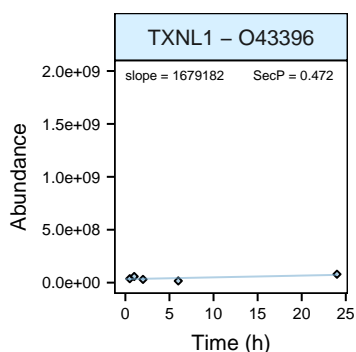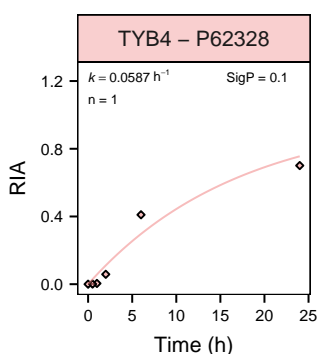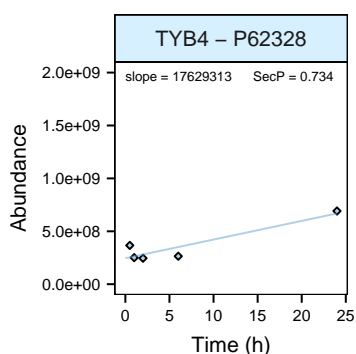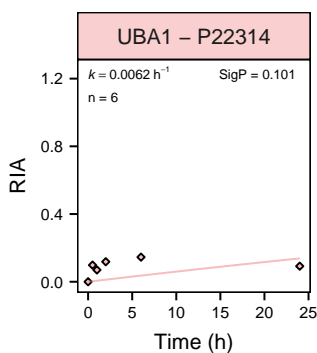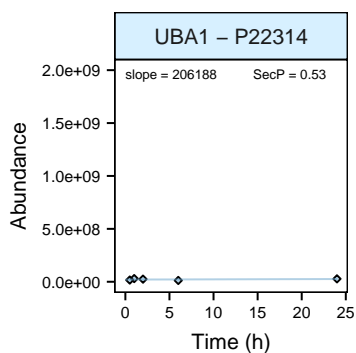

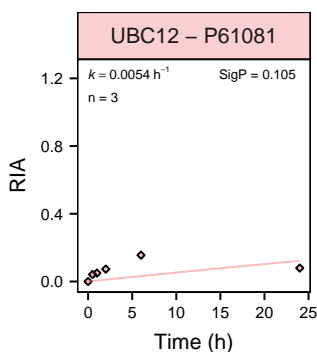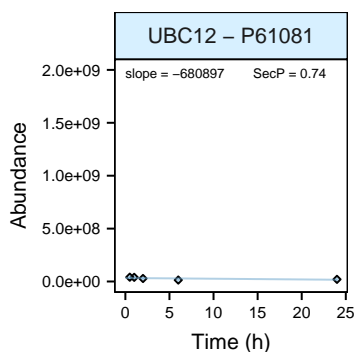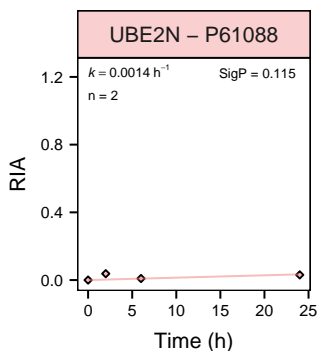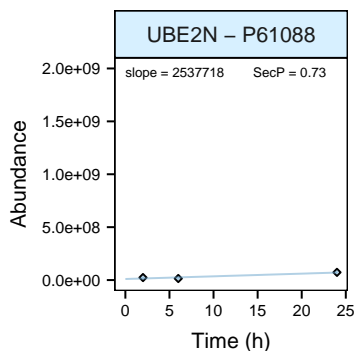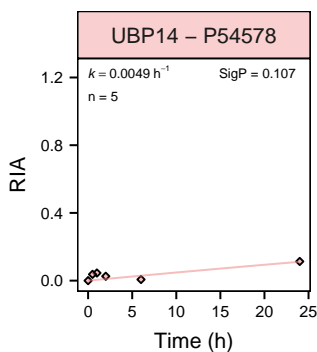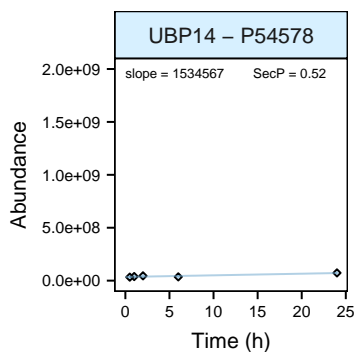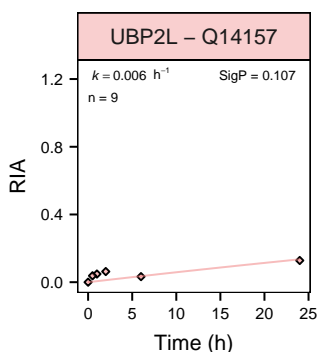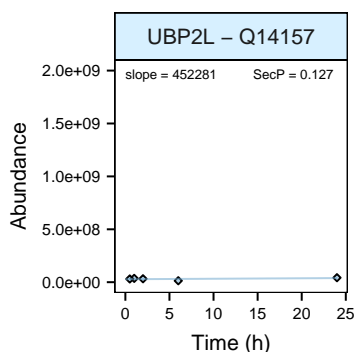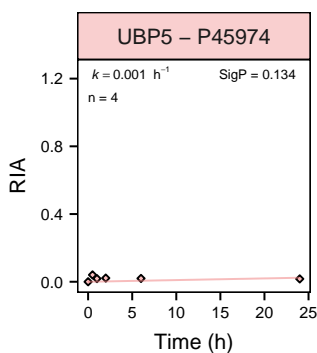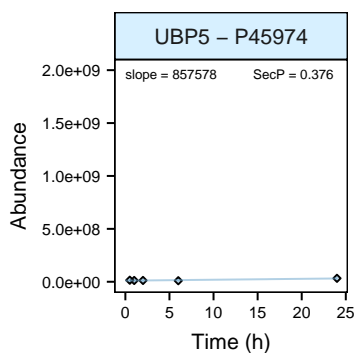

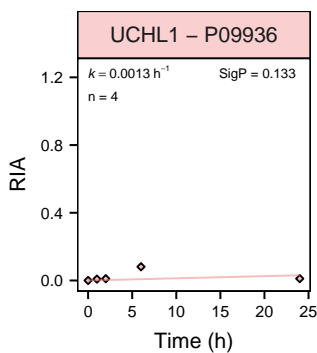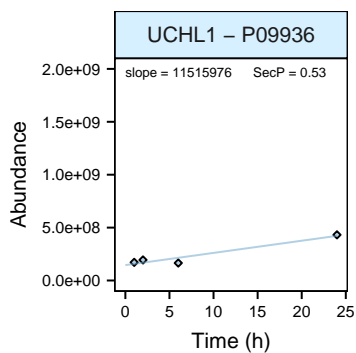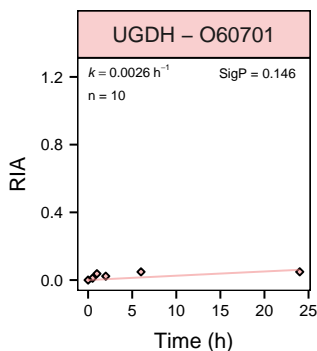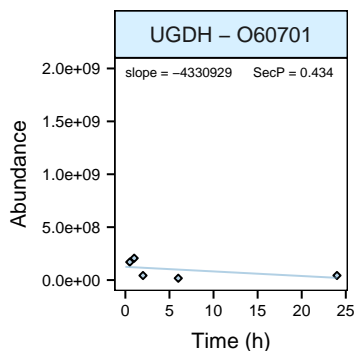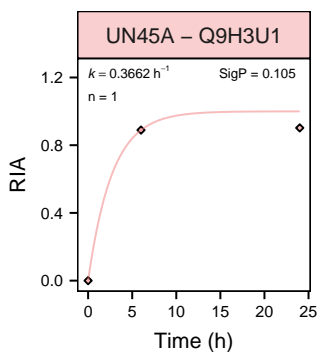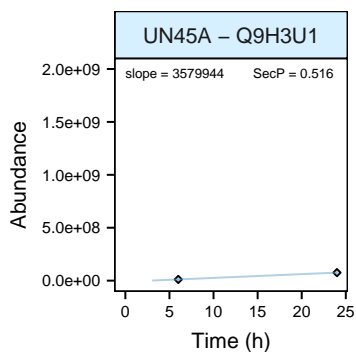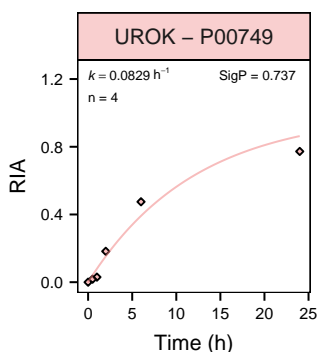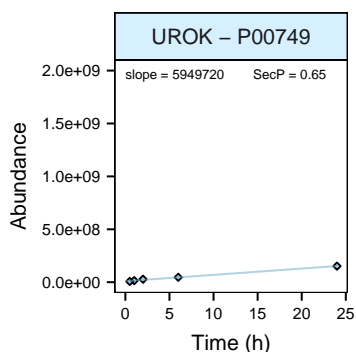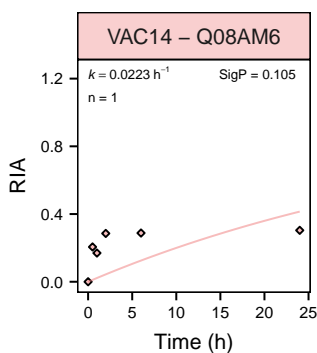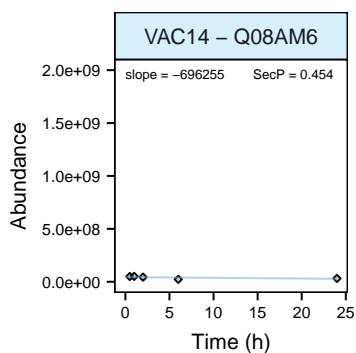

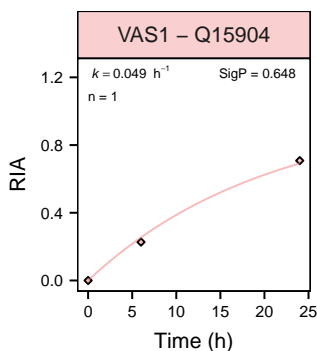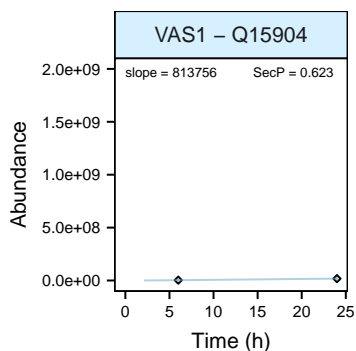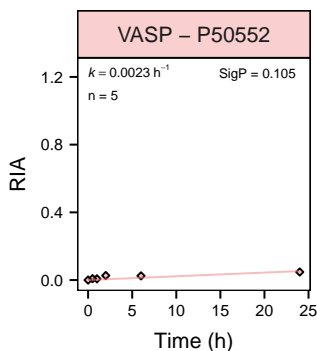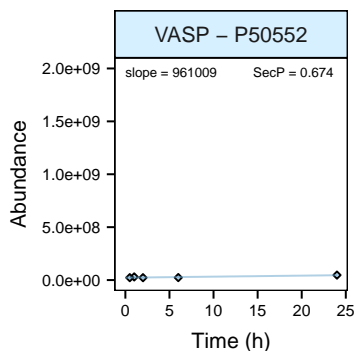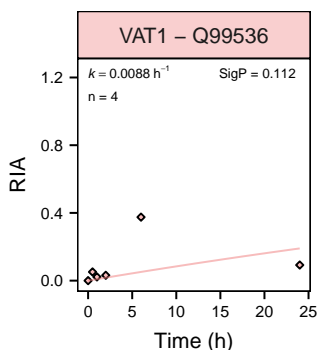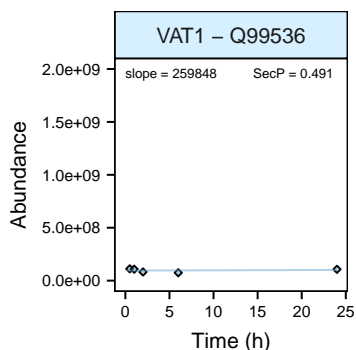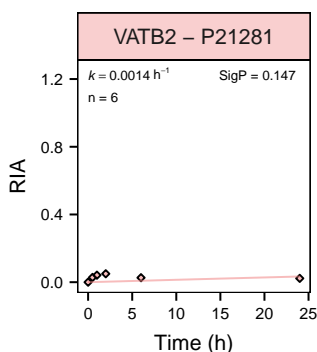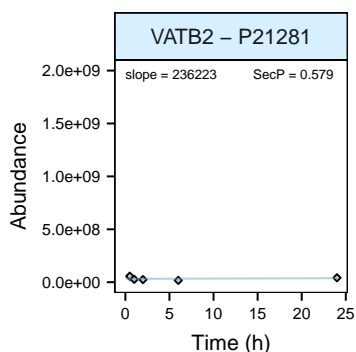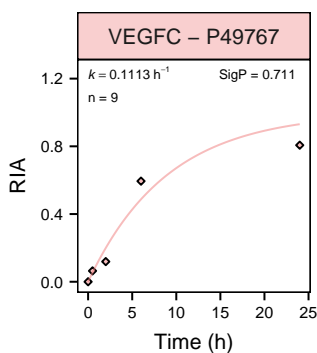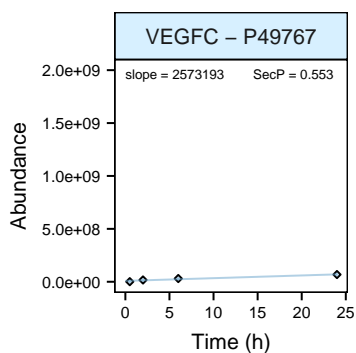

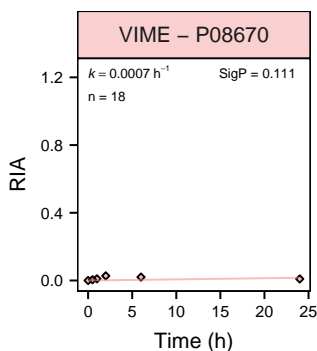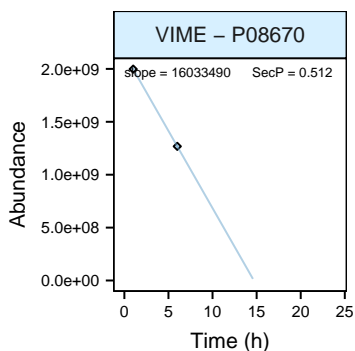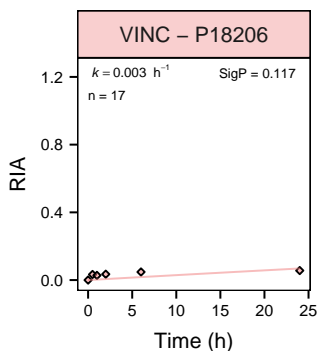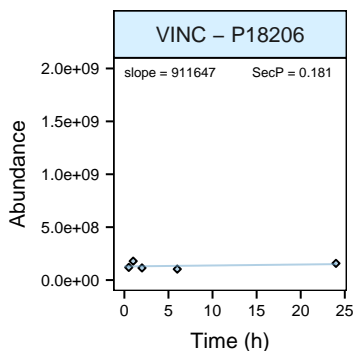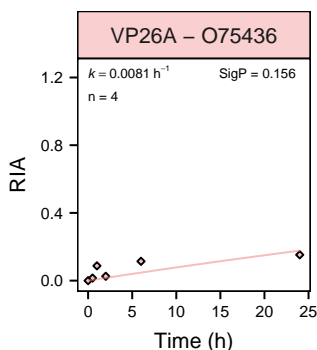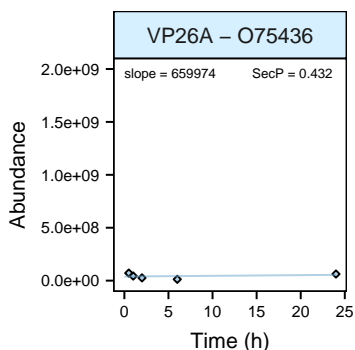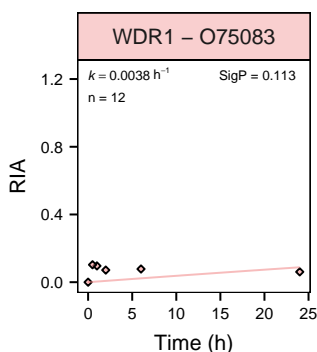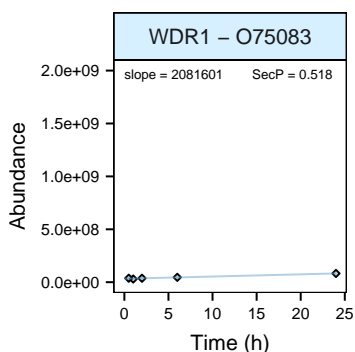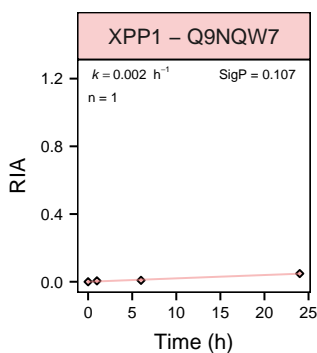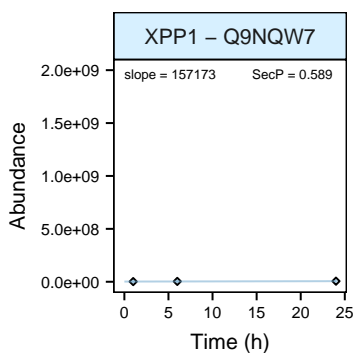

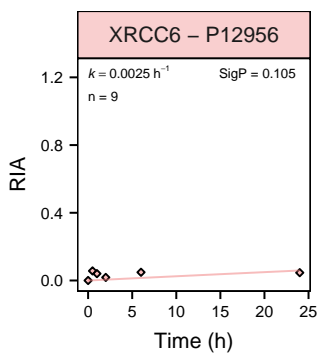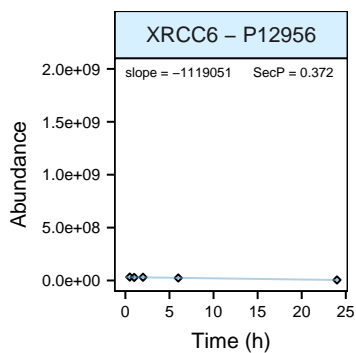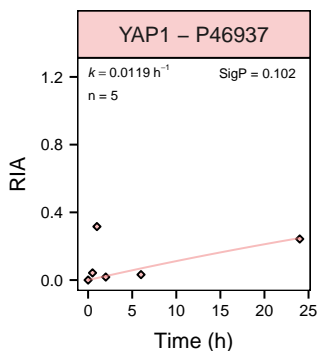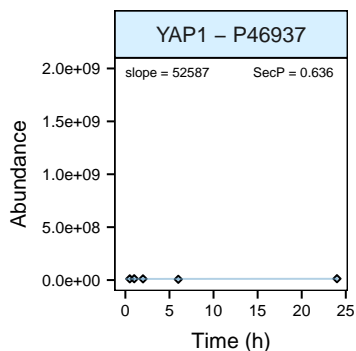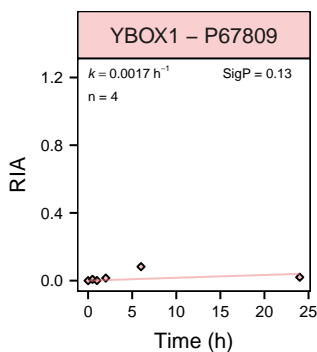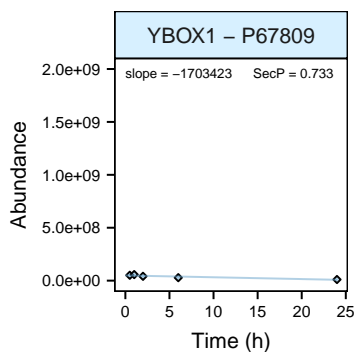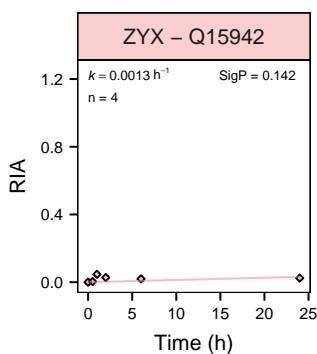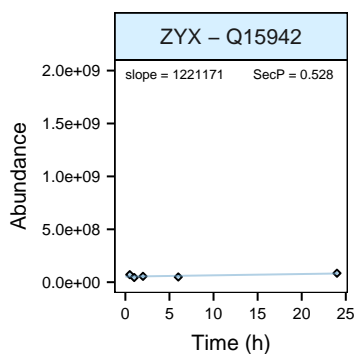

Supplement: supplemental material [file 134290_3_supp_155571_p888gq.pdf]

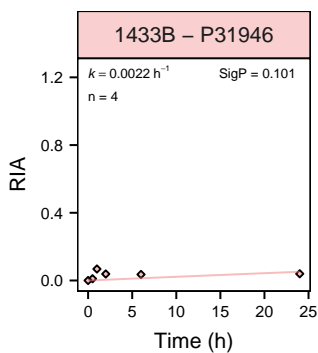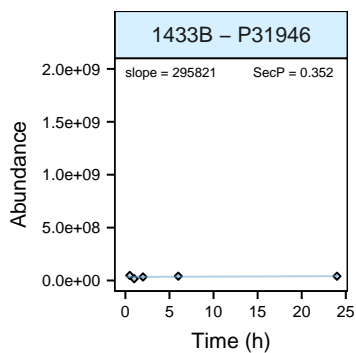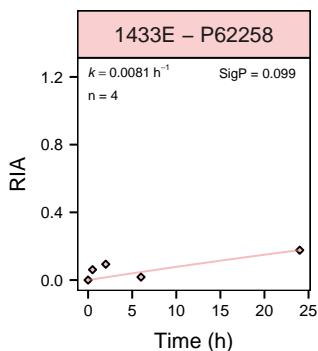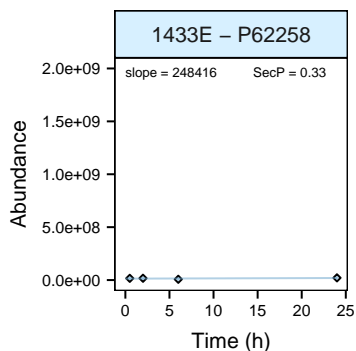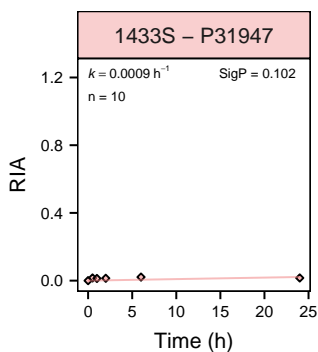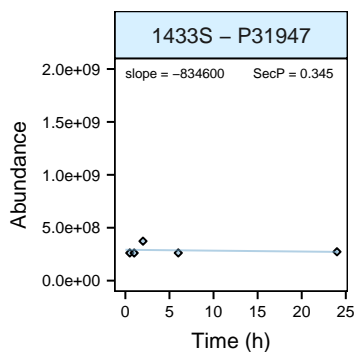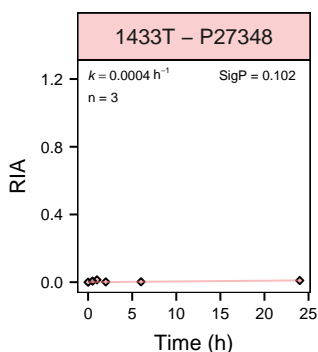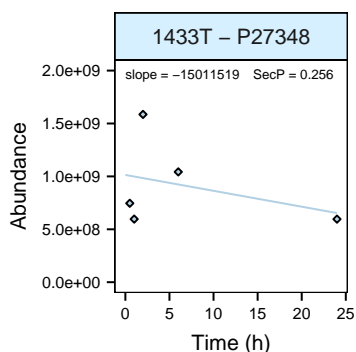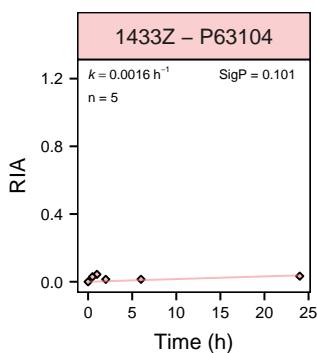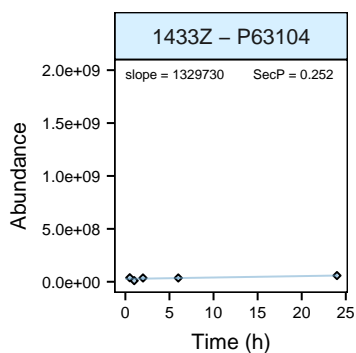

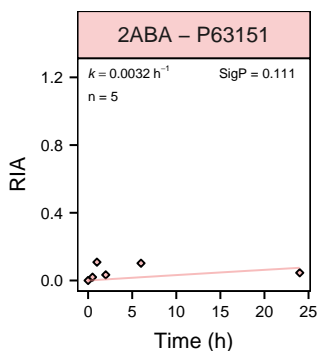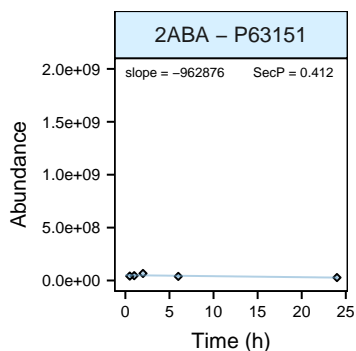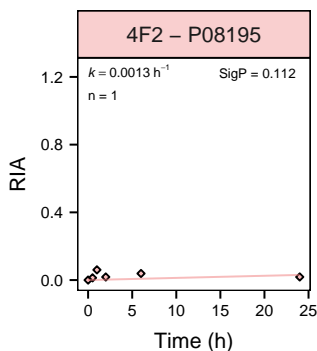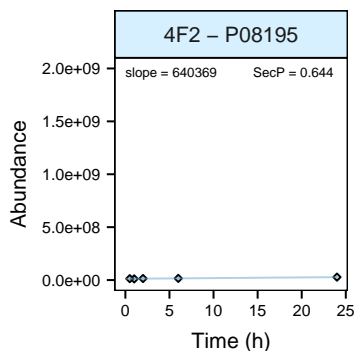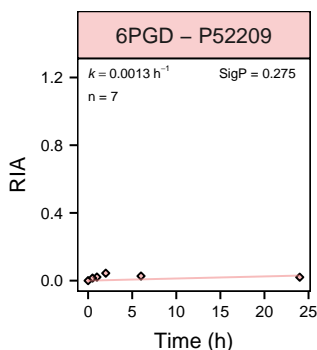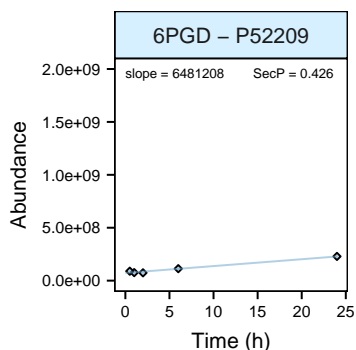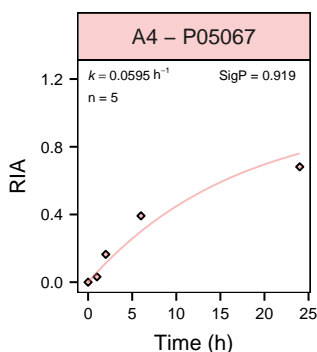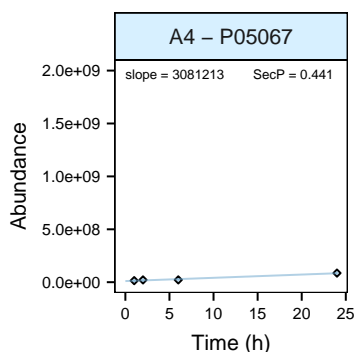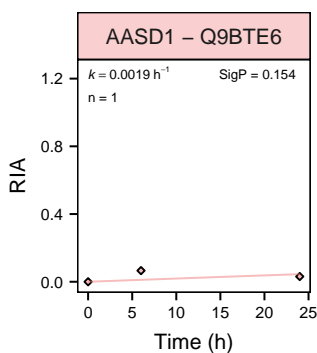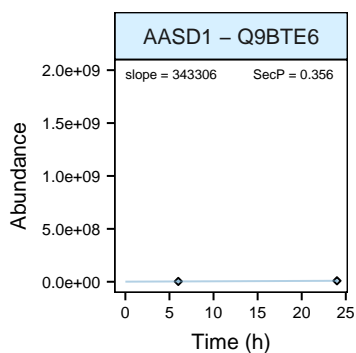

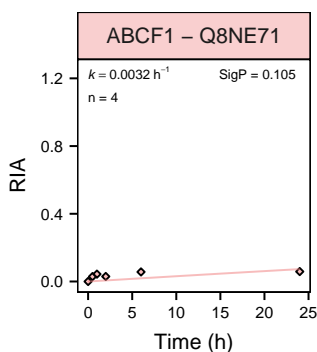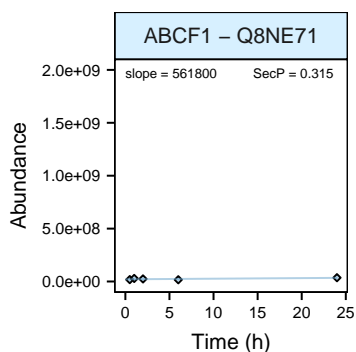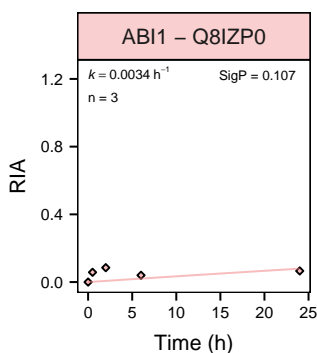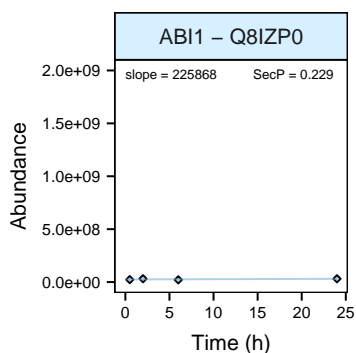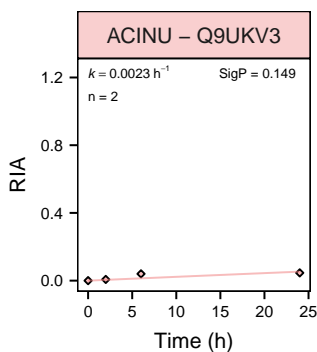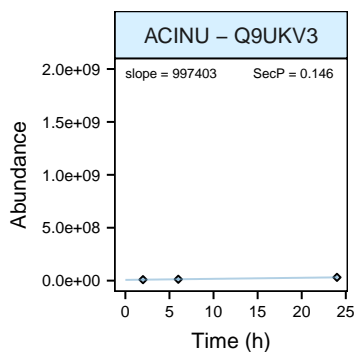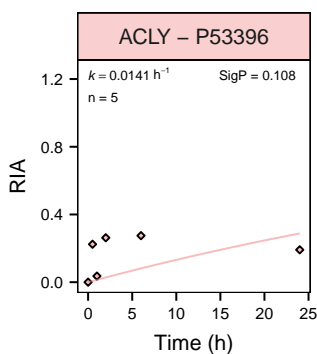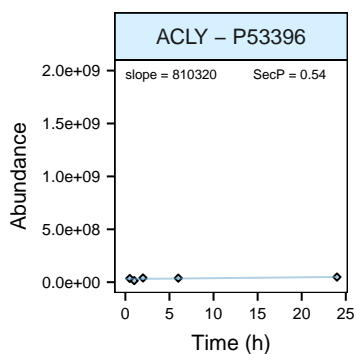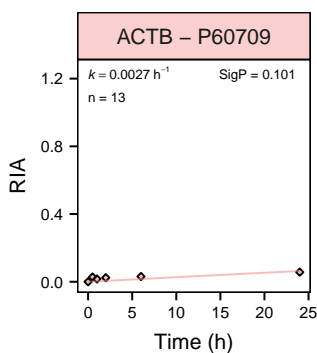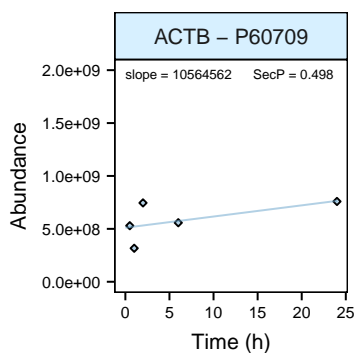

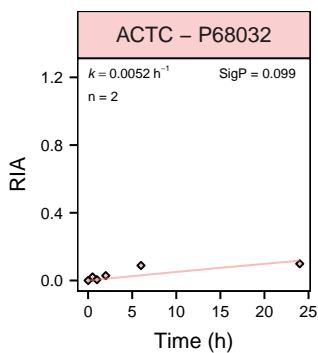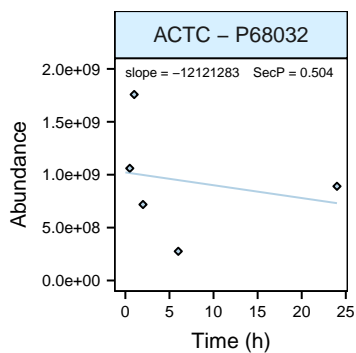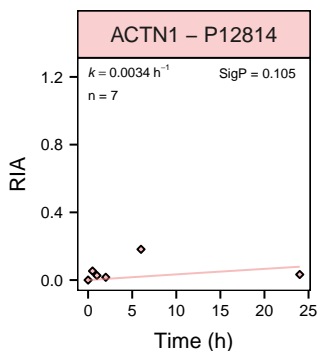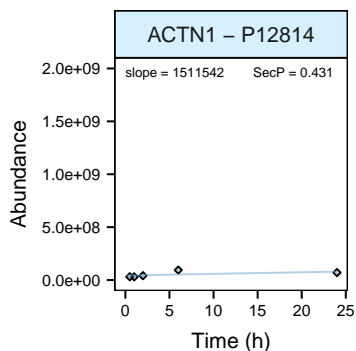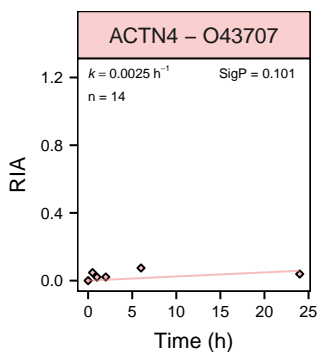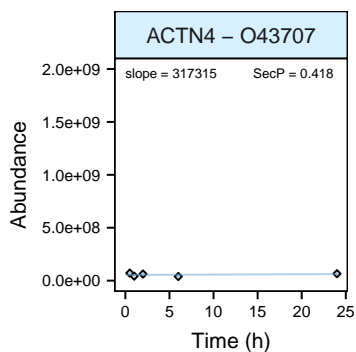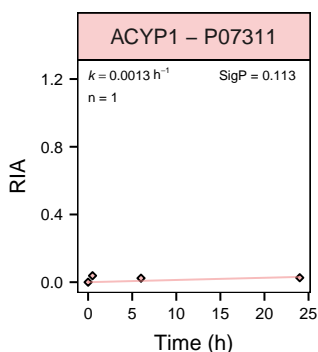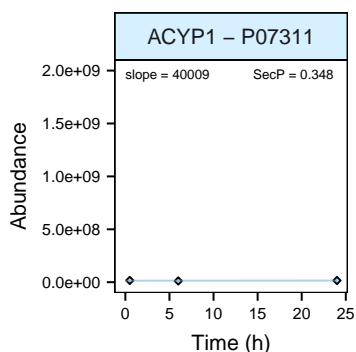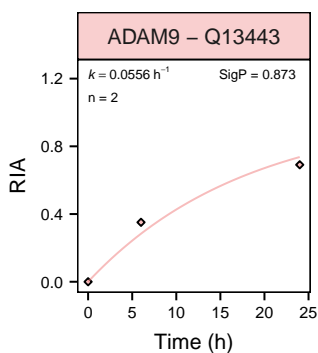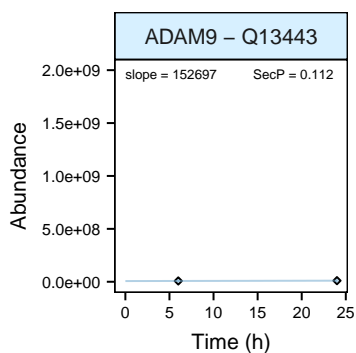

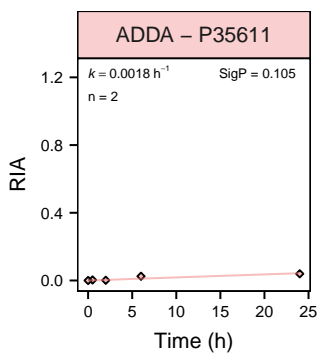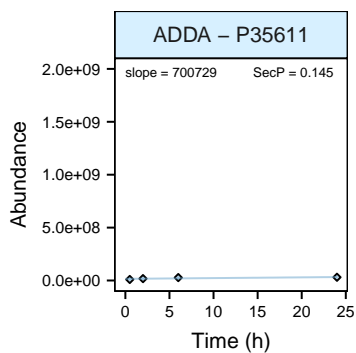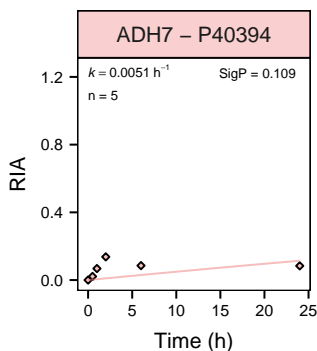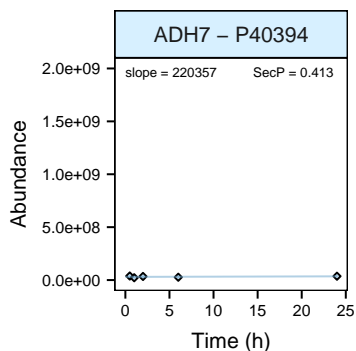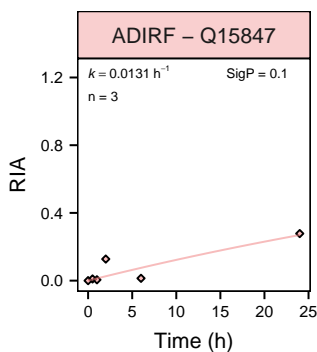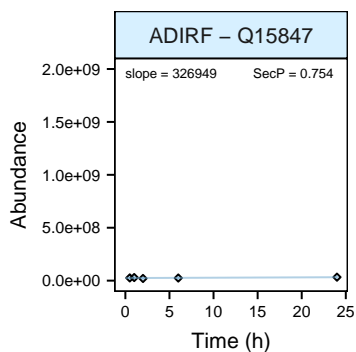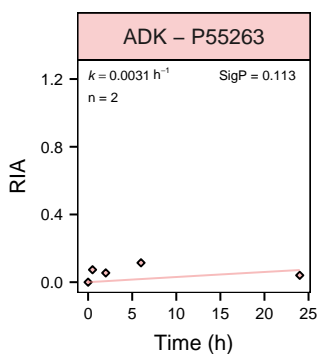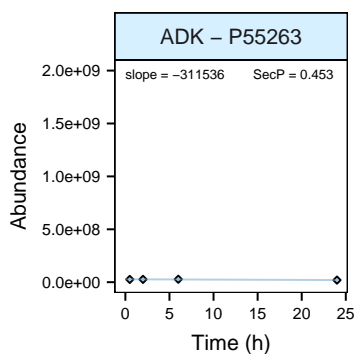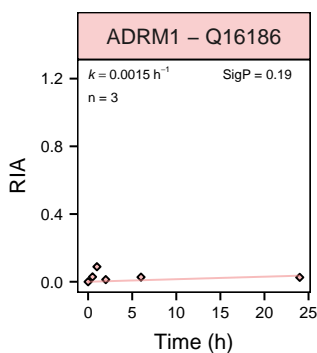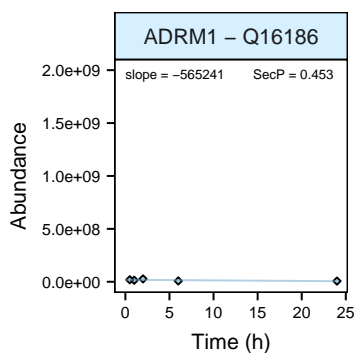

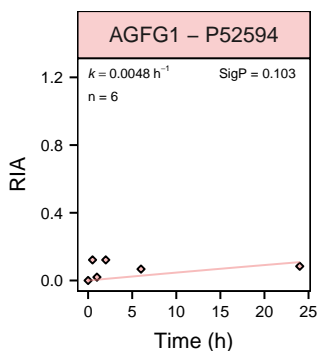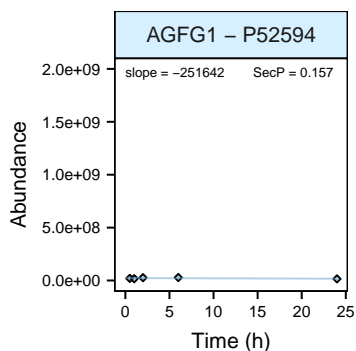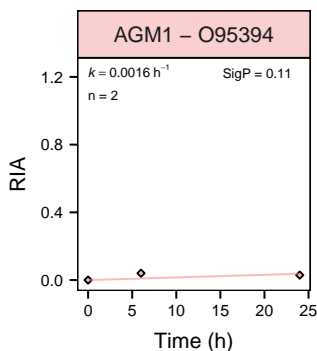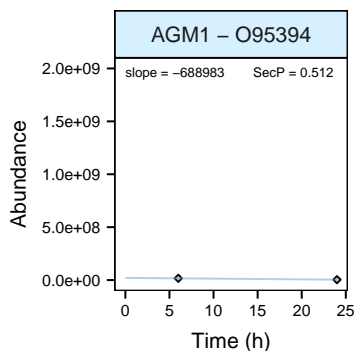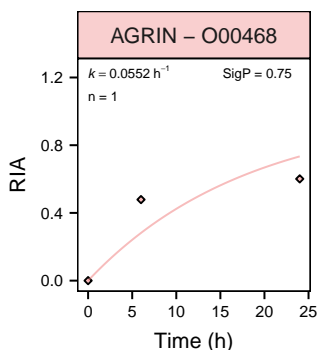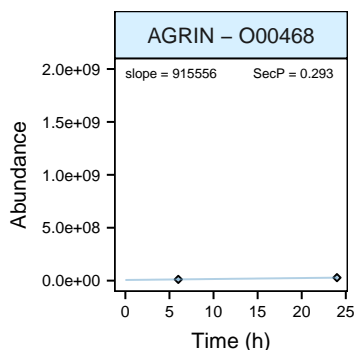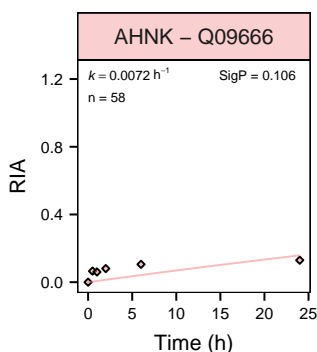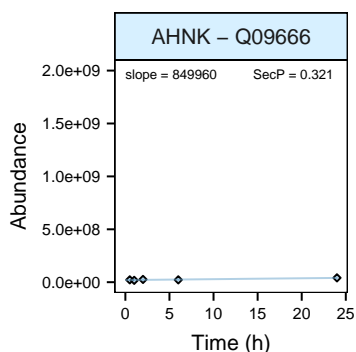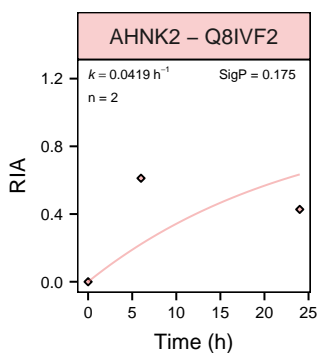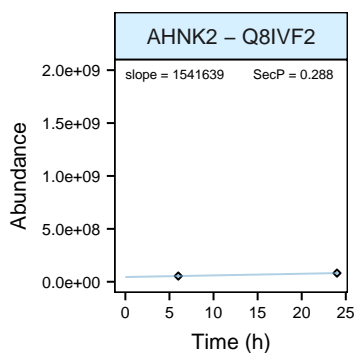

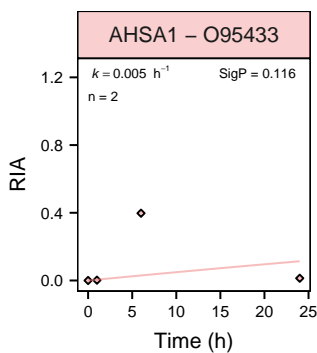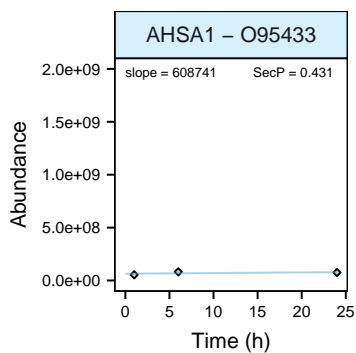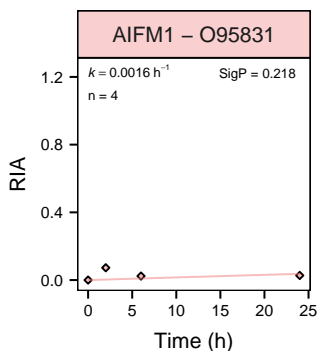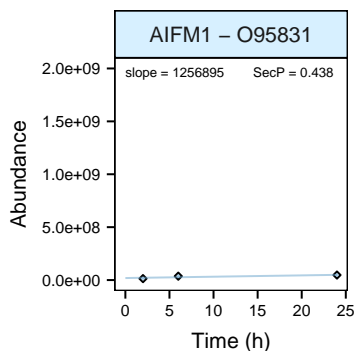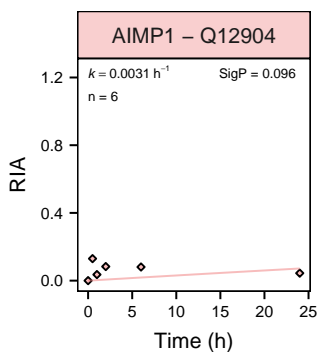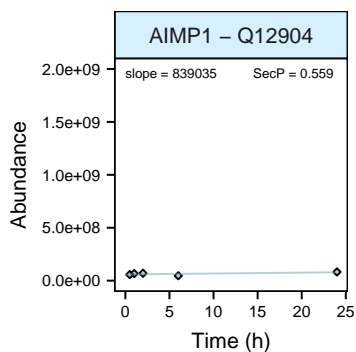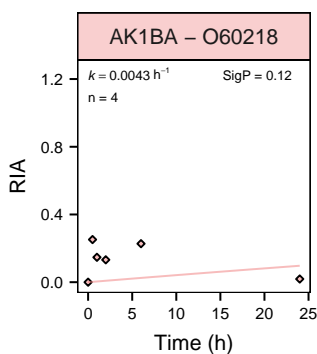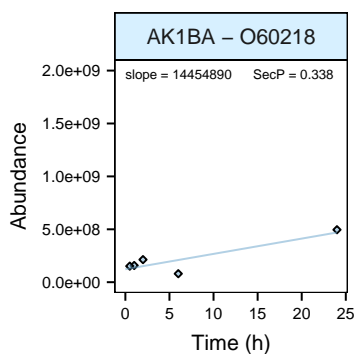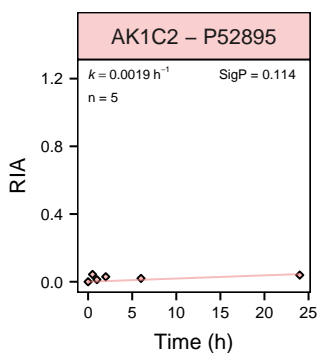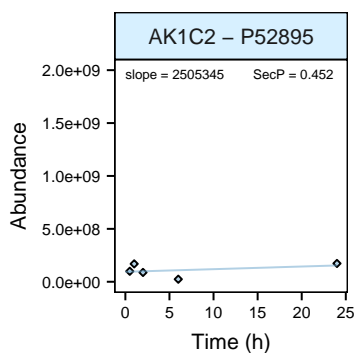

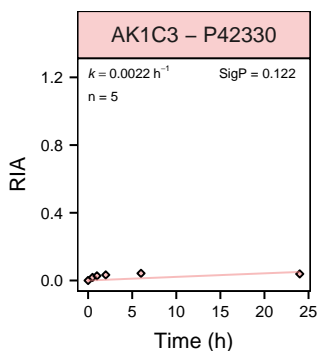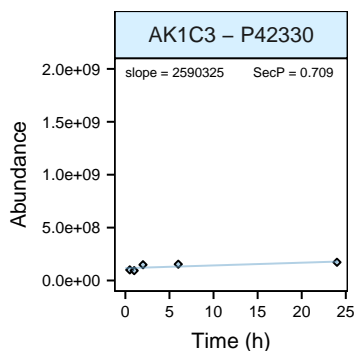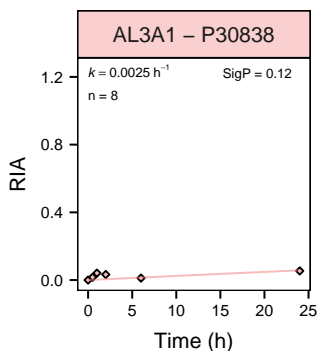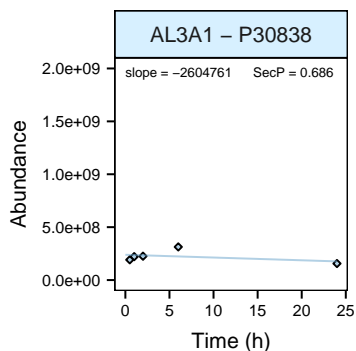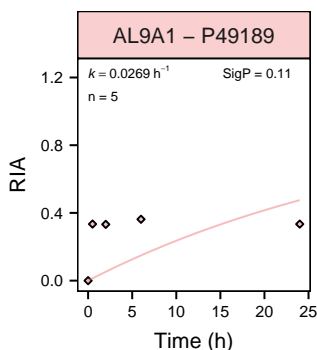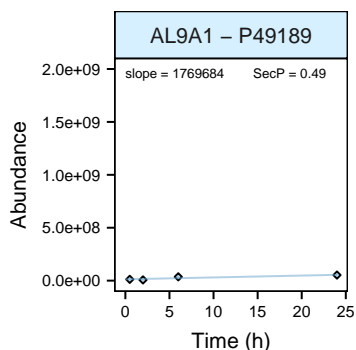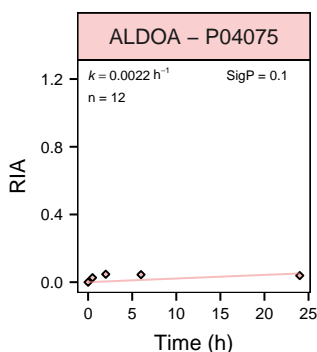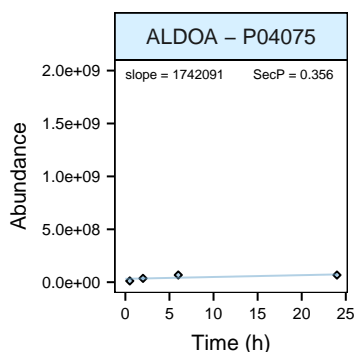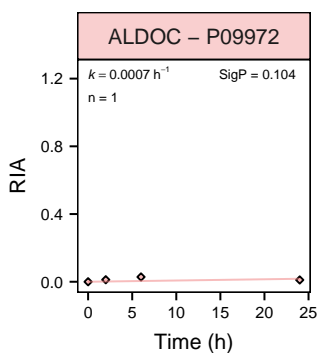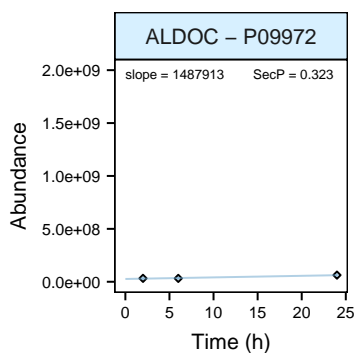

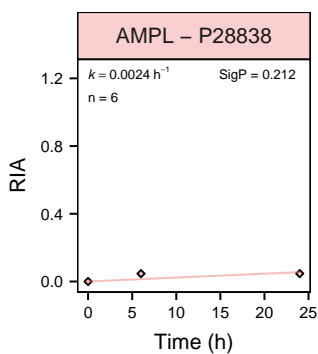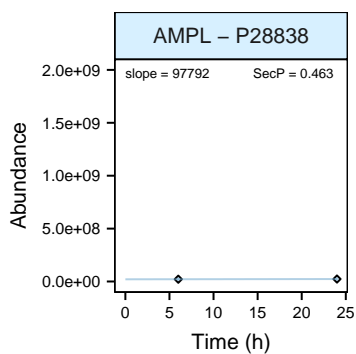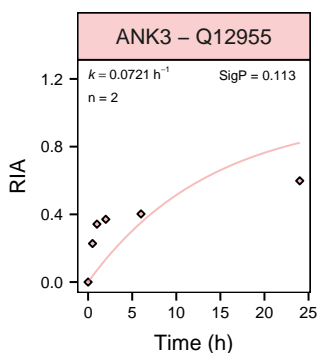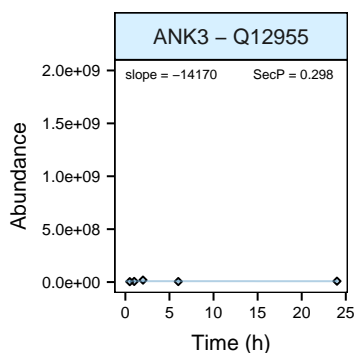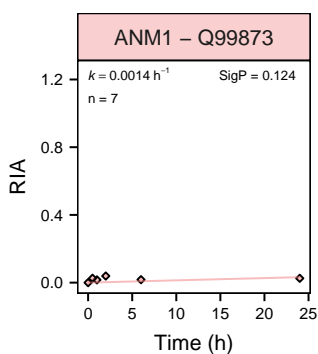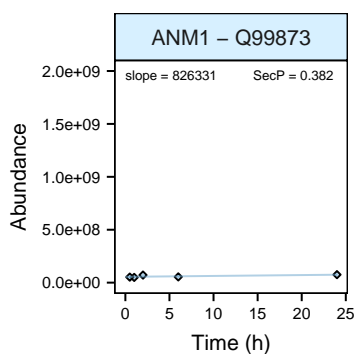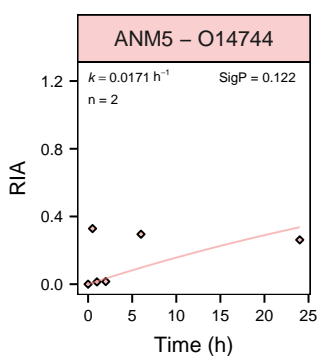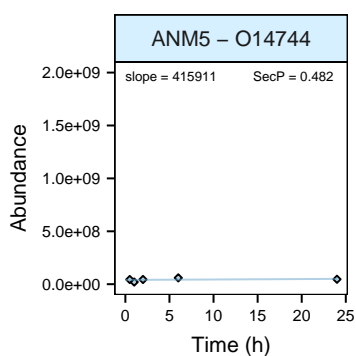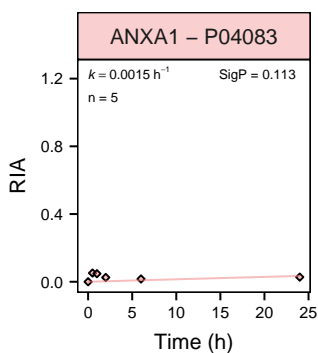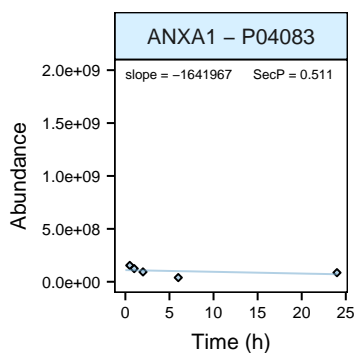

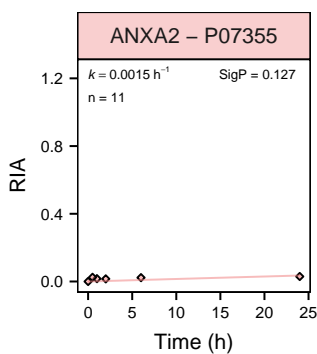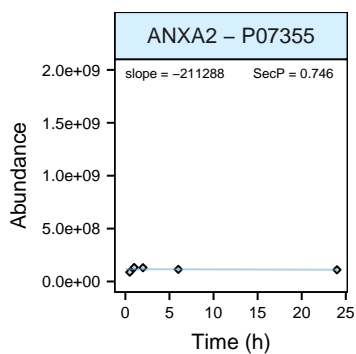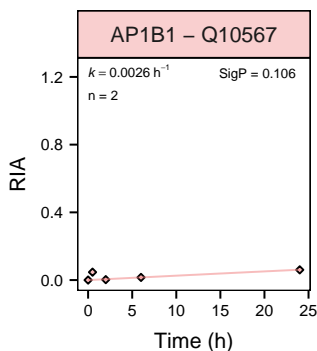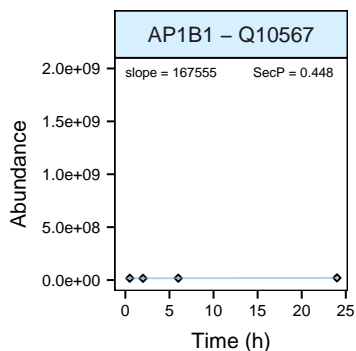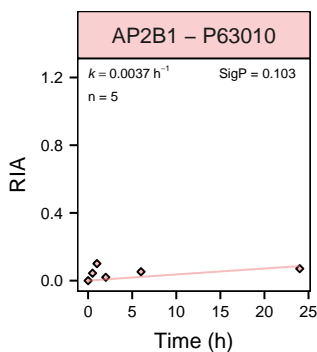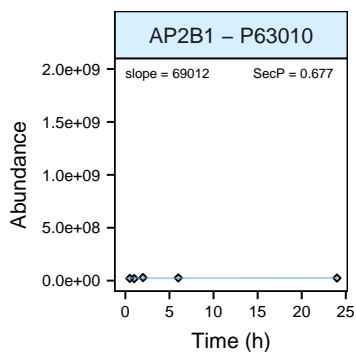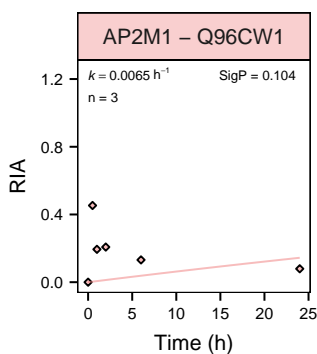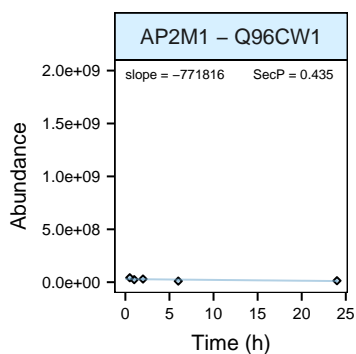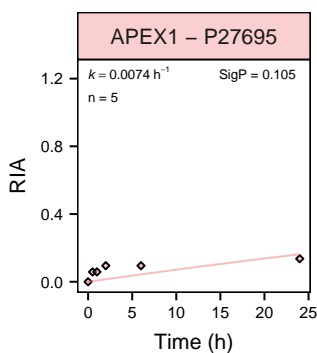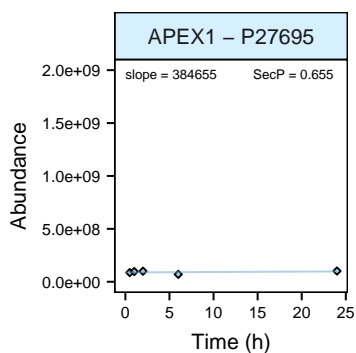

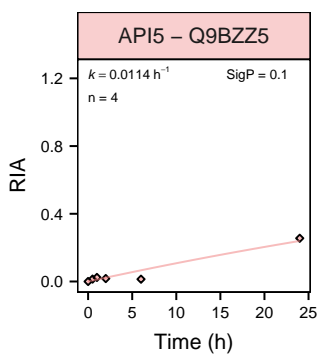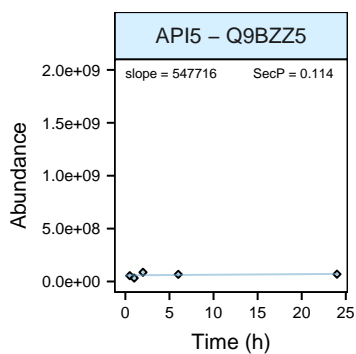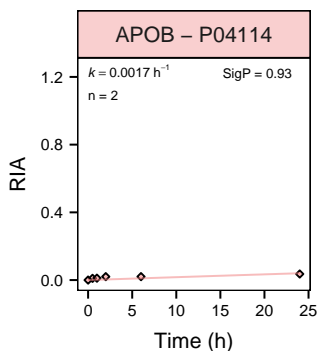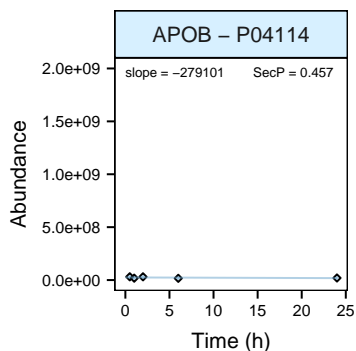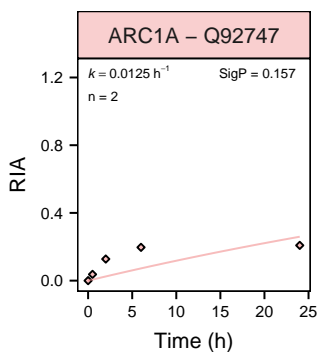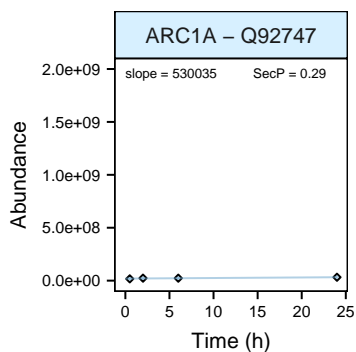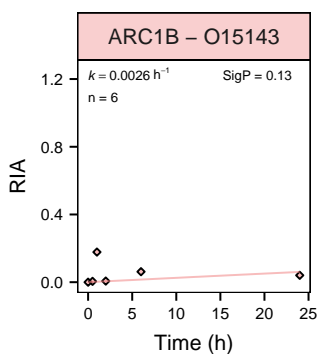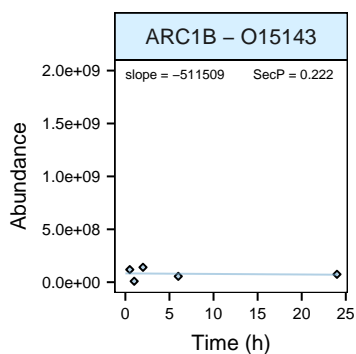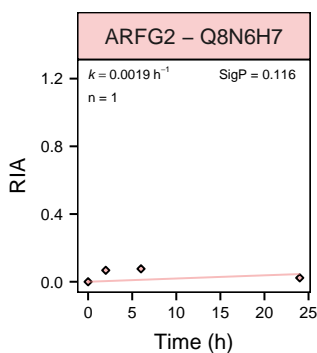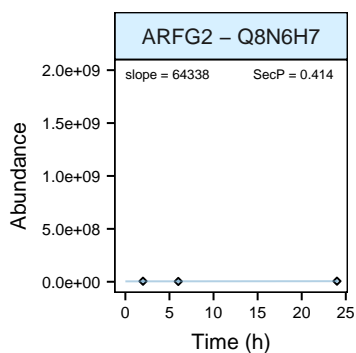

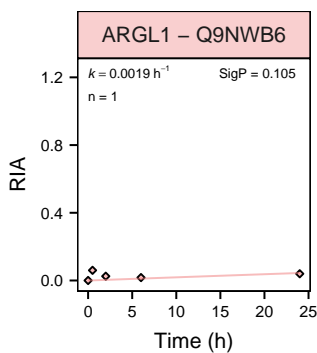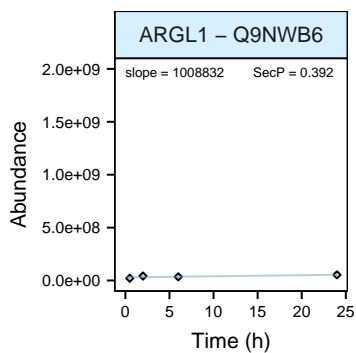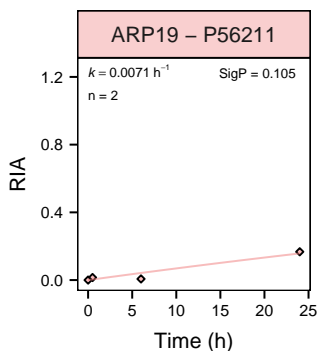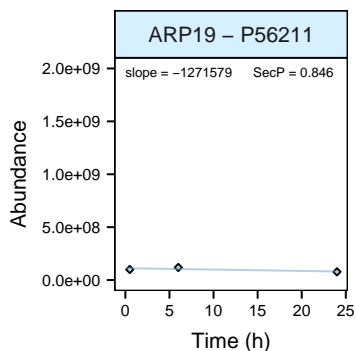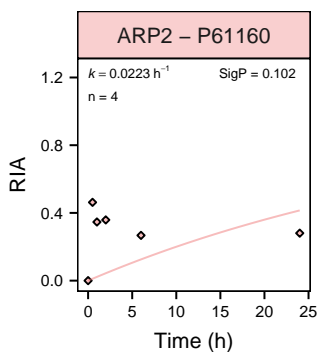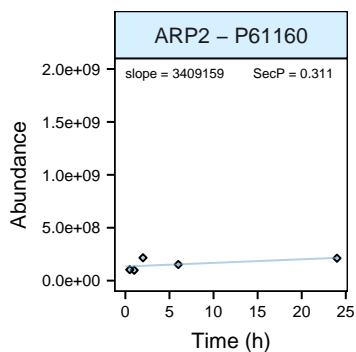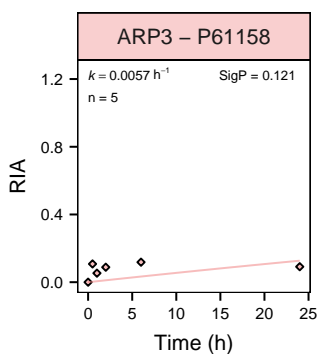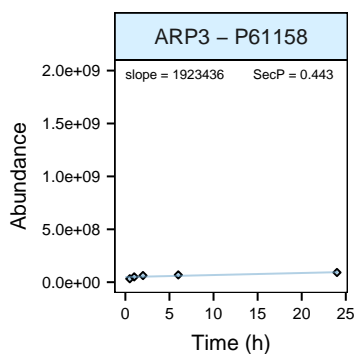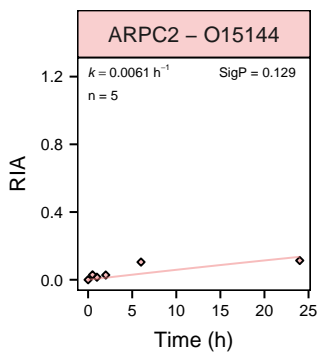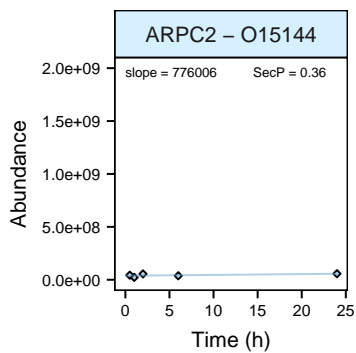

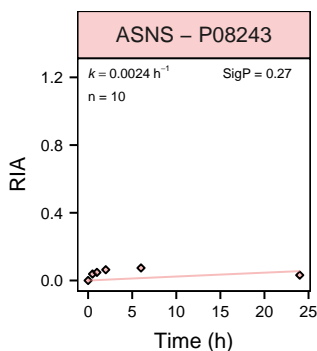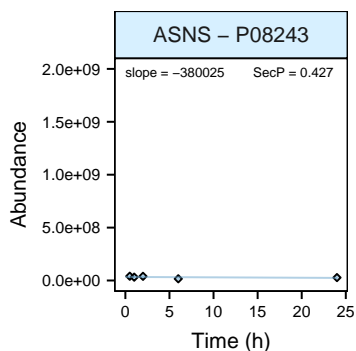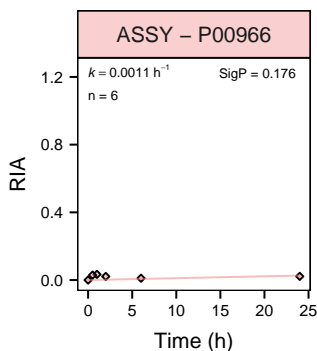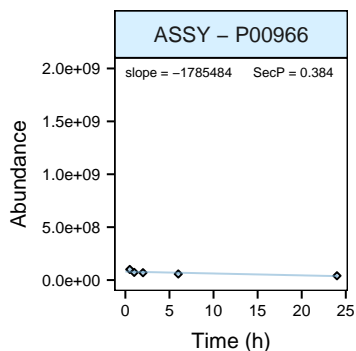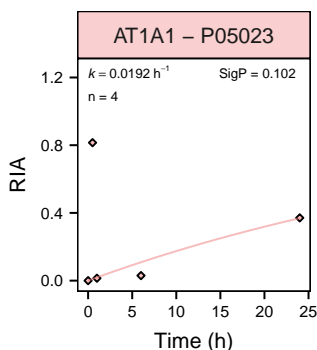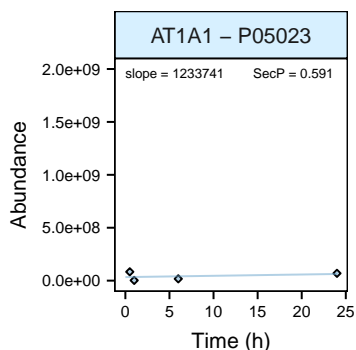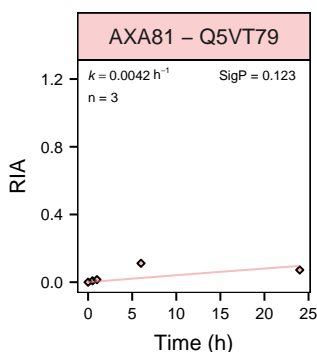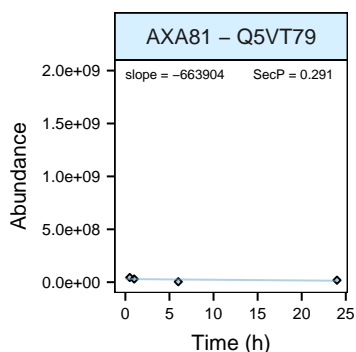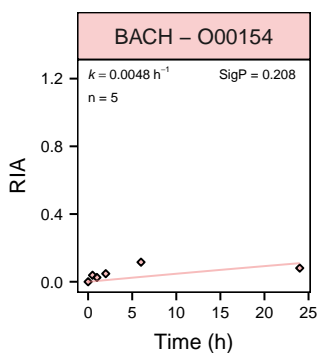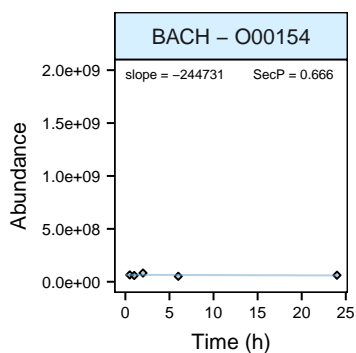

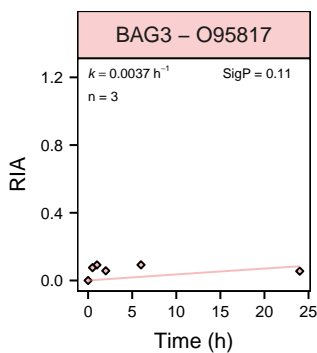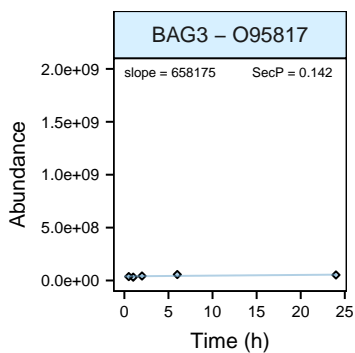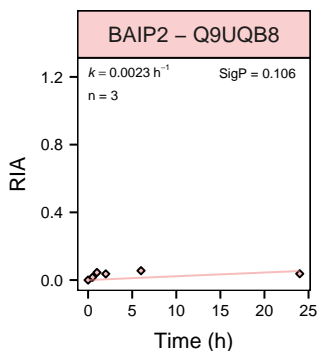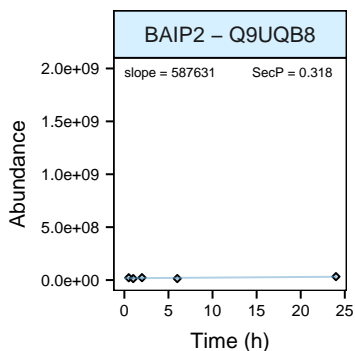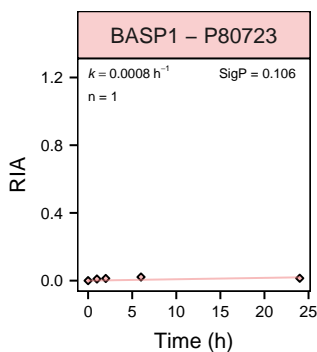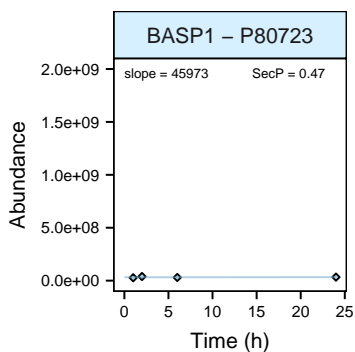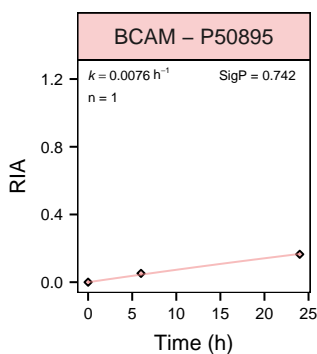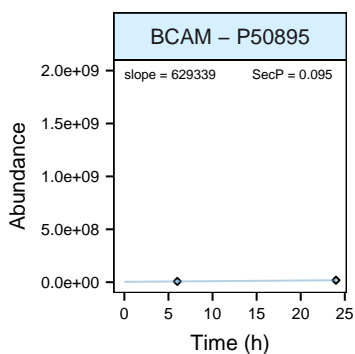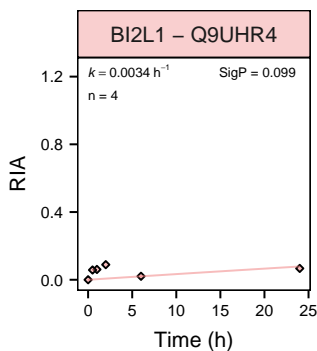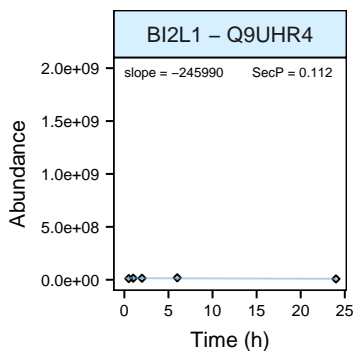

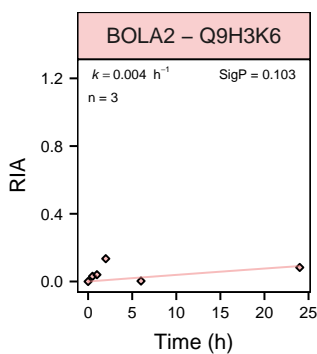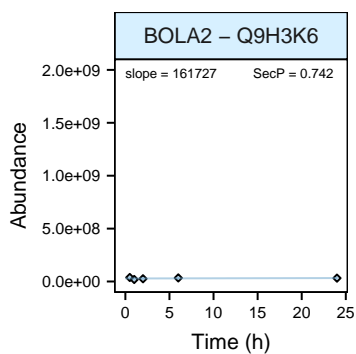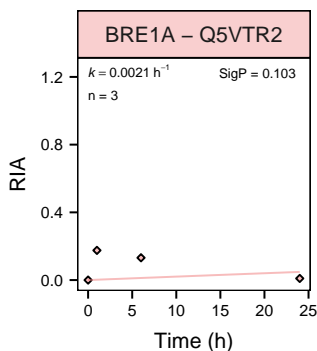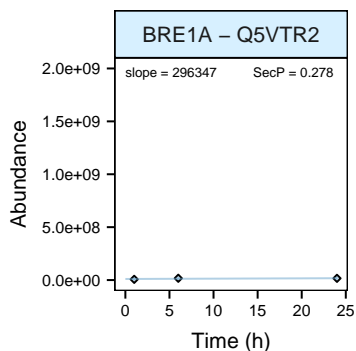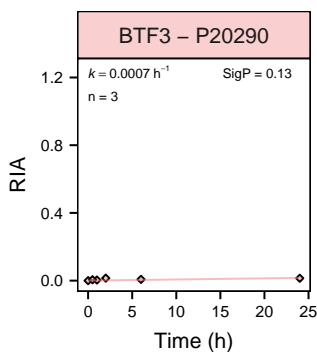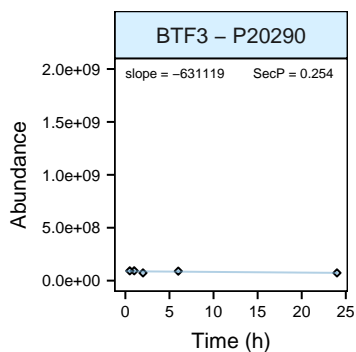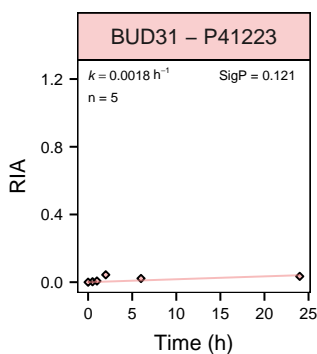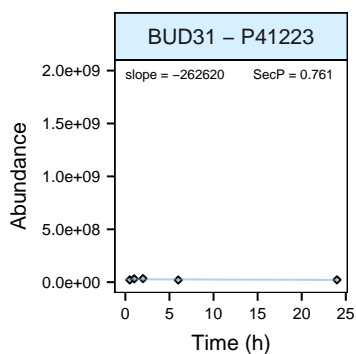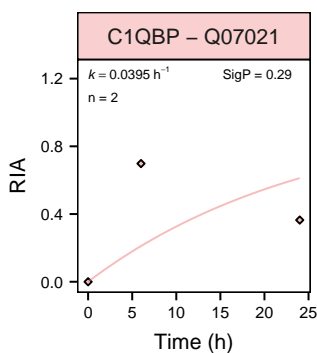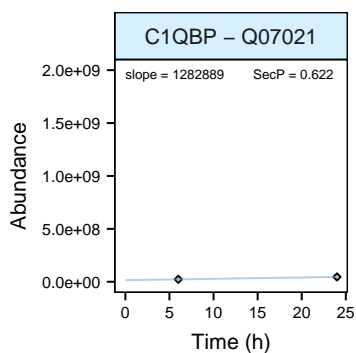

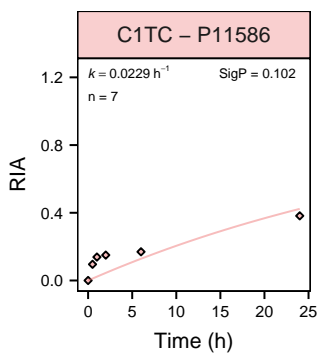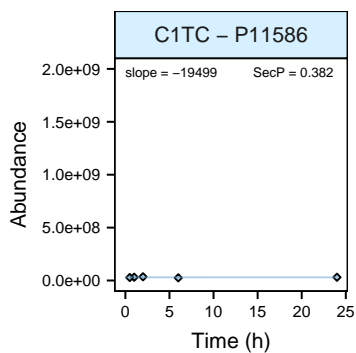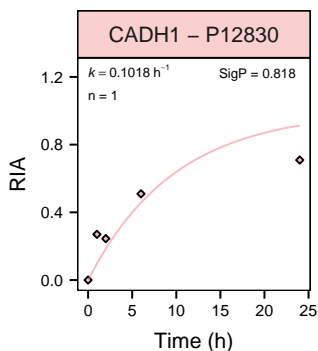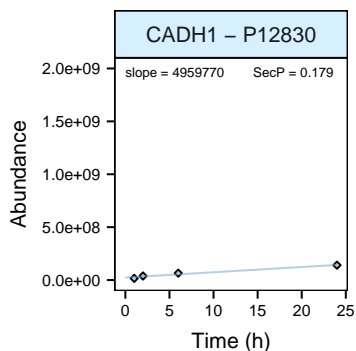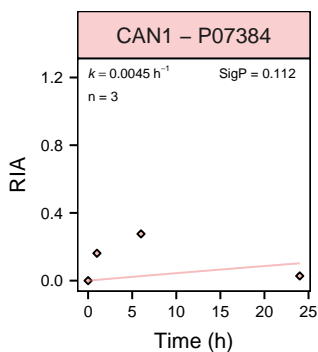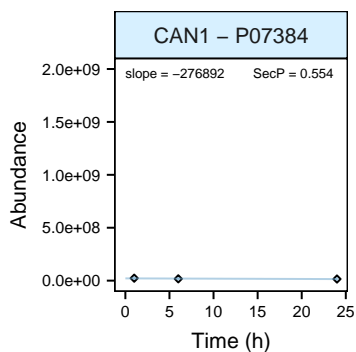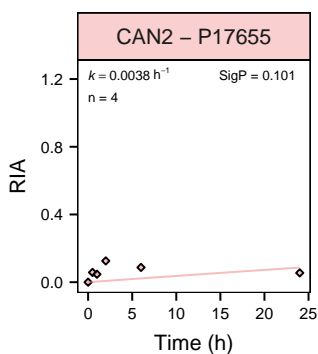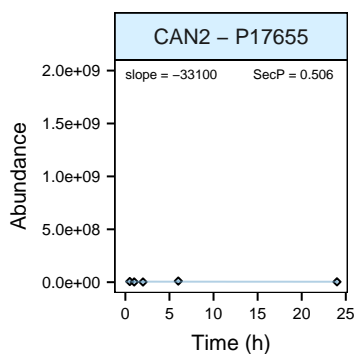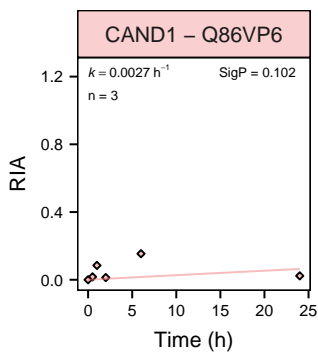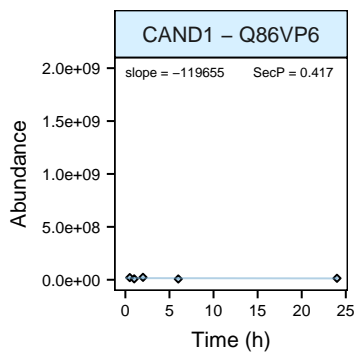

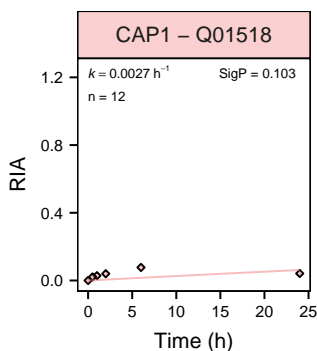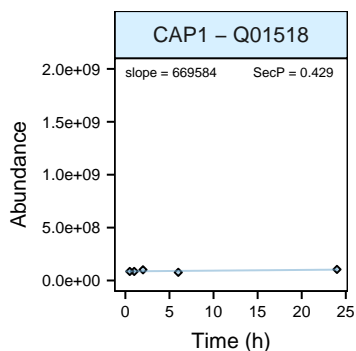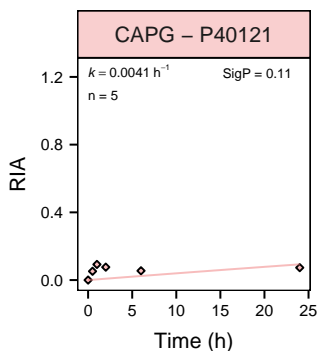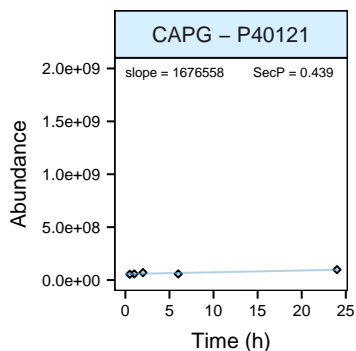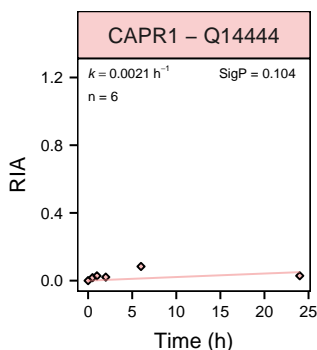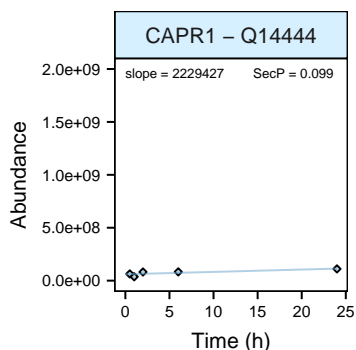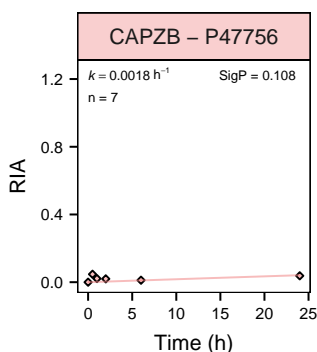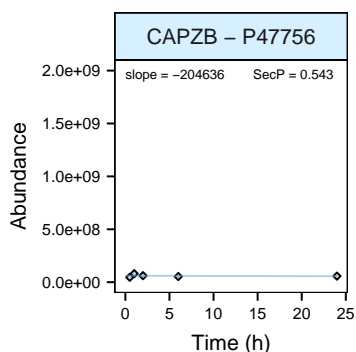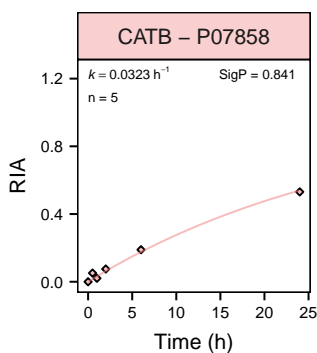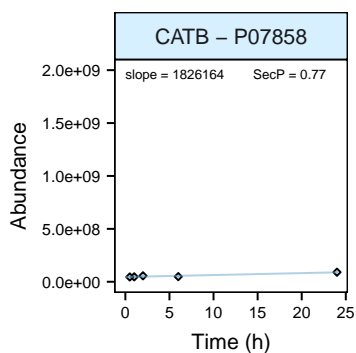

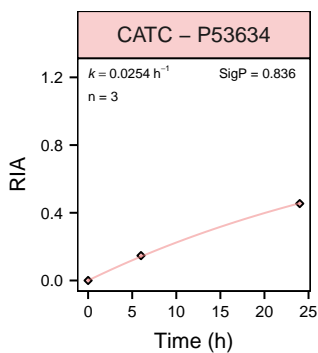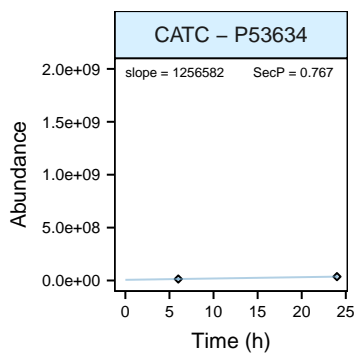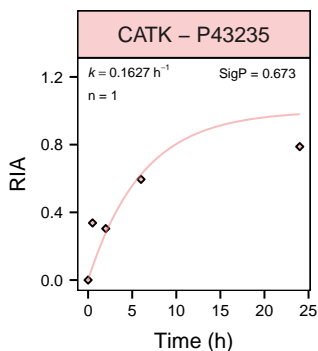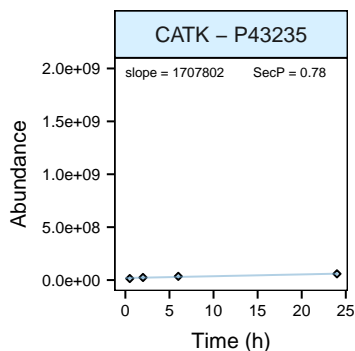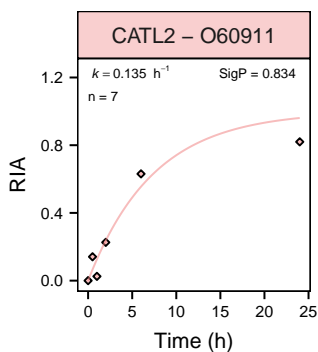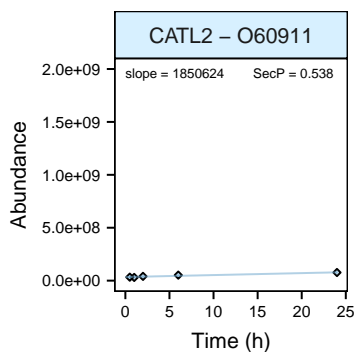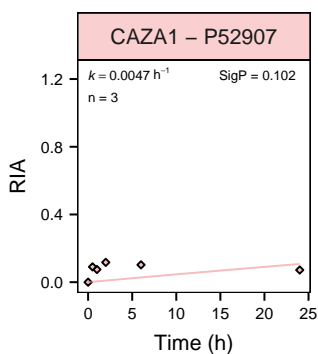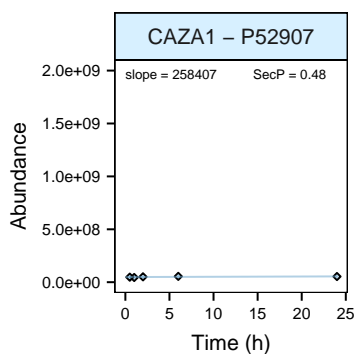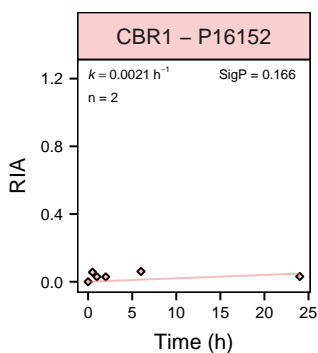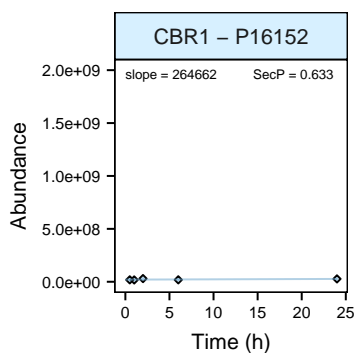

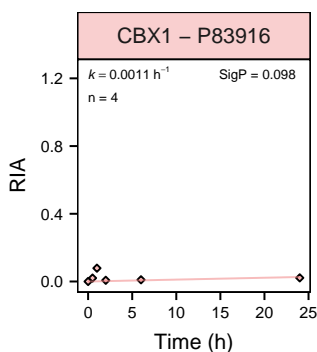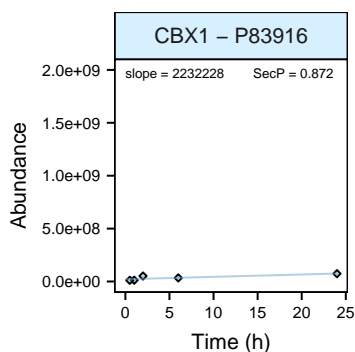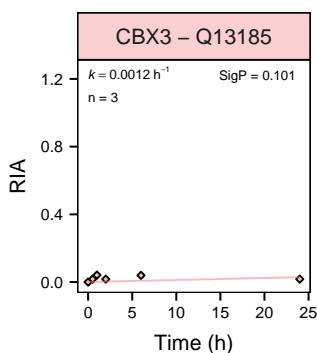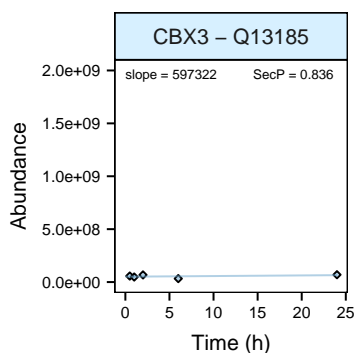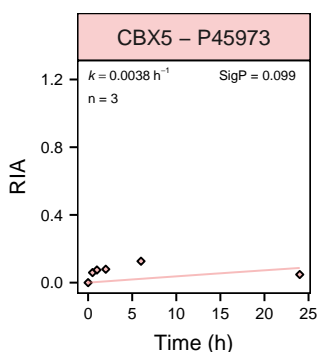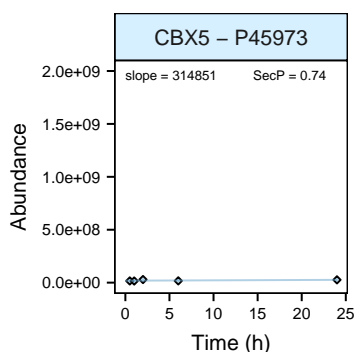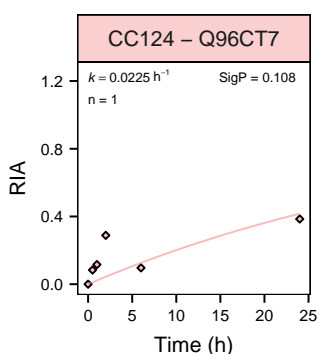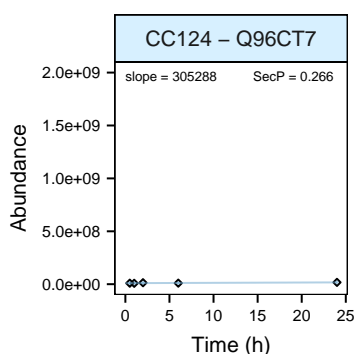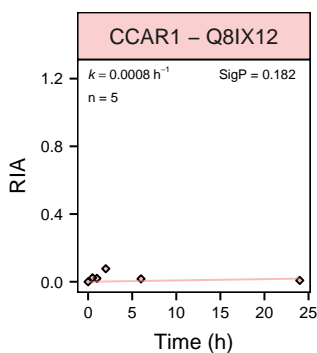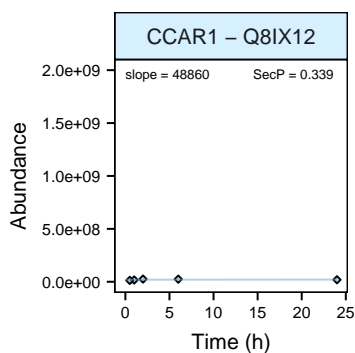

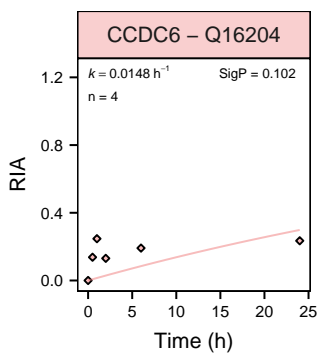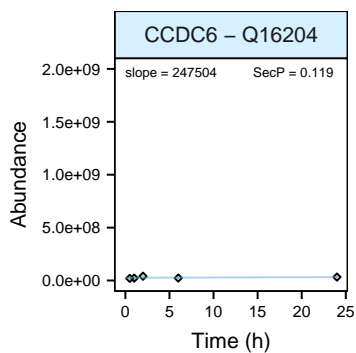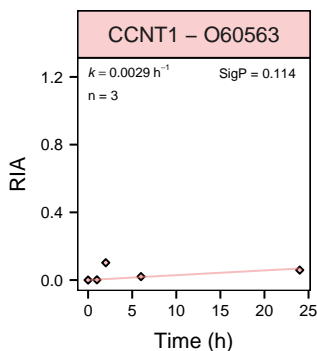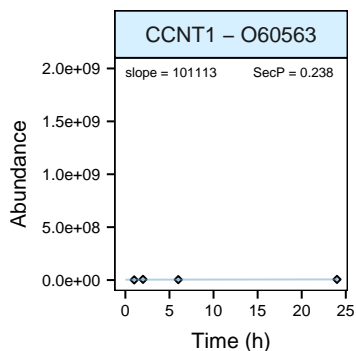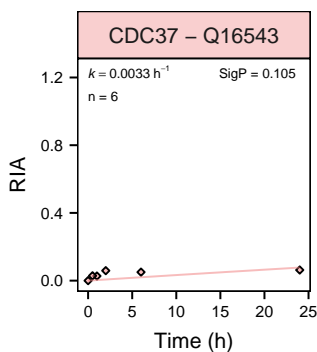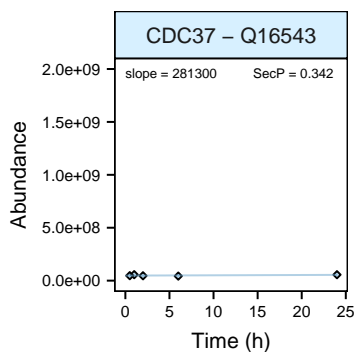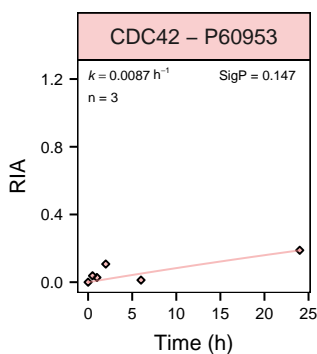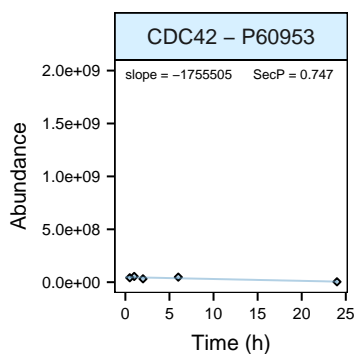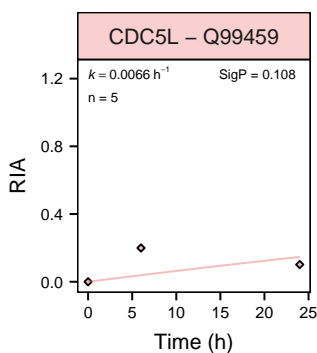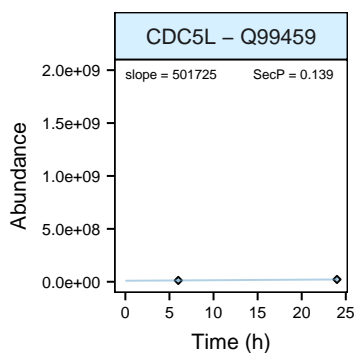

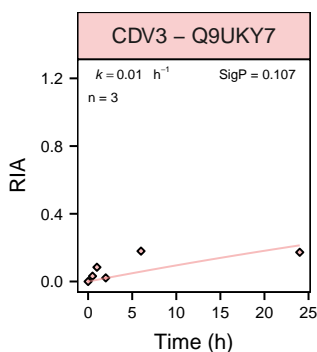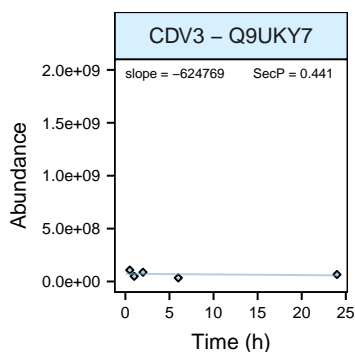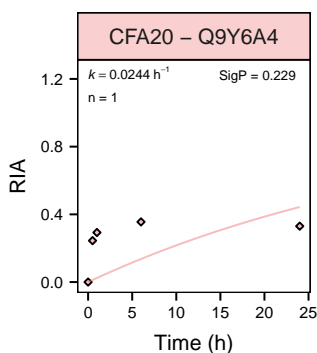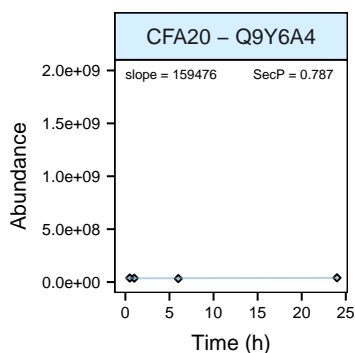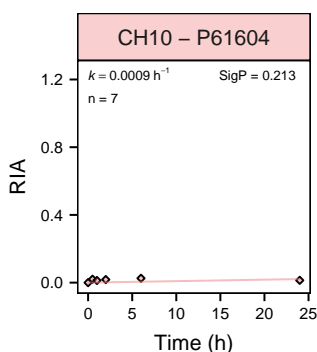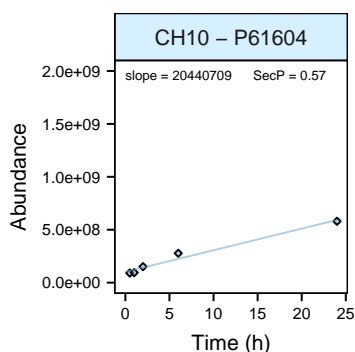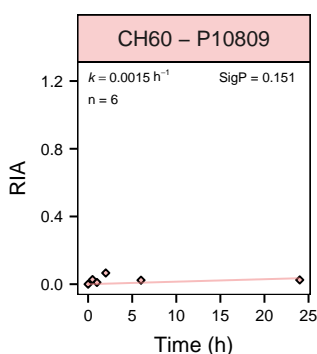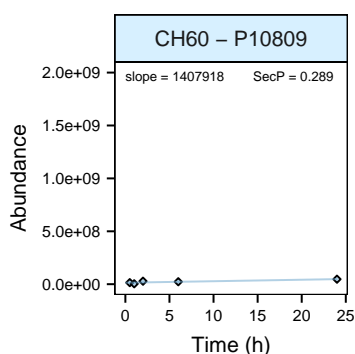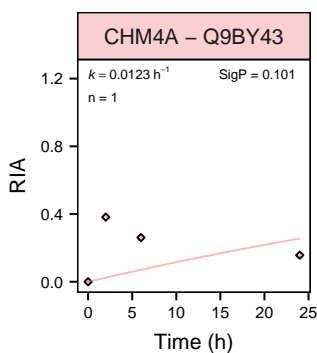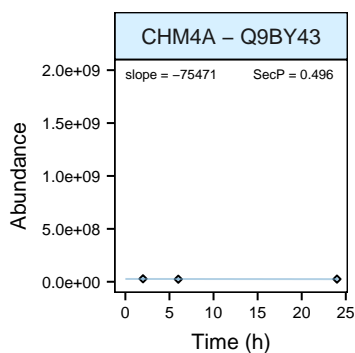

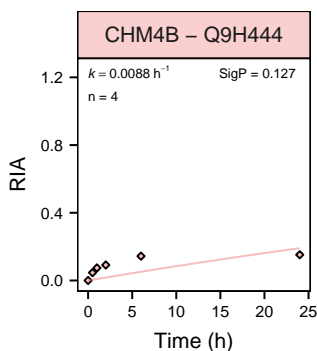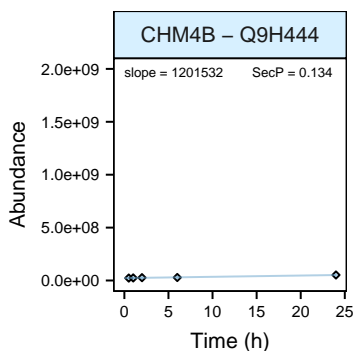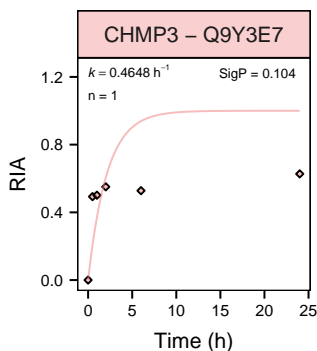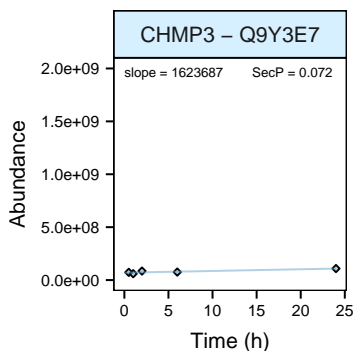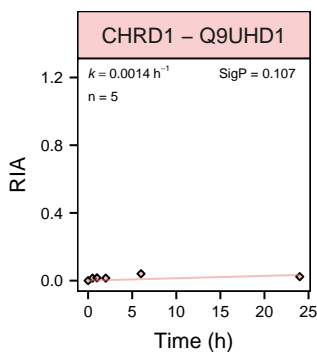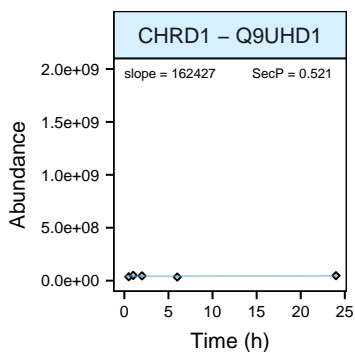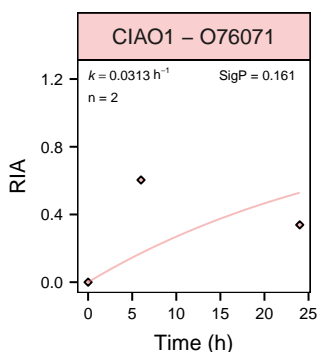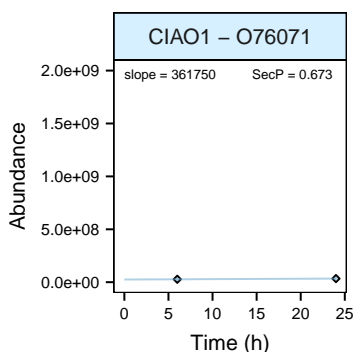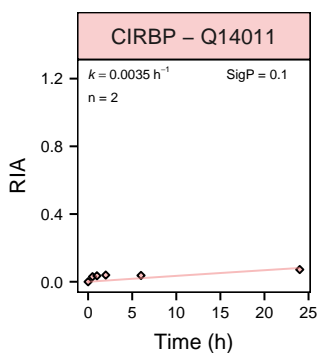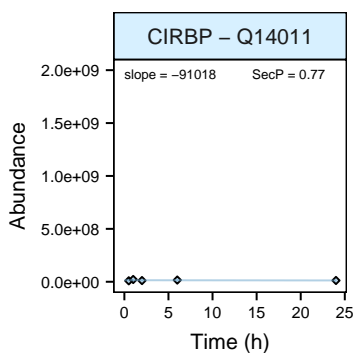

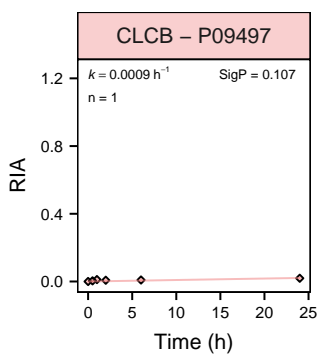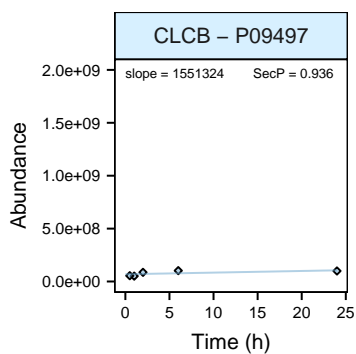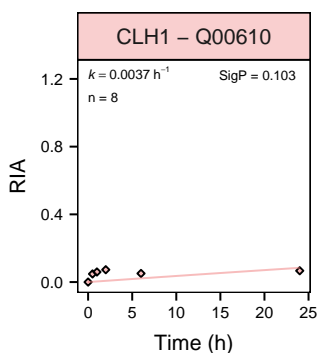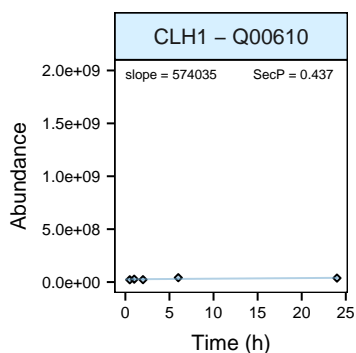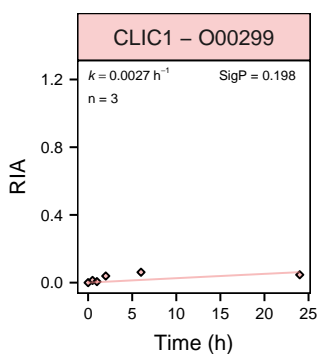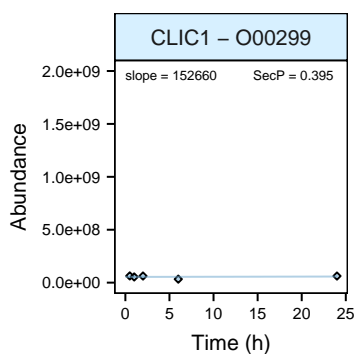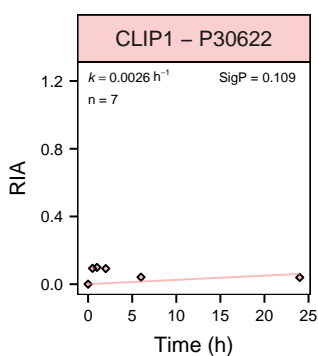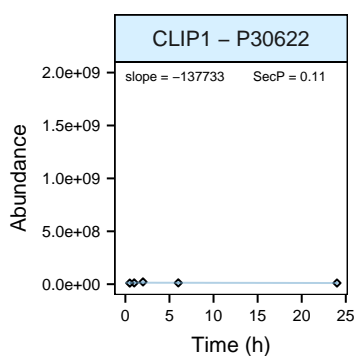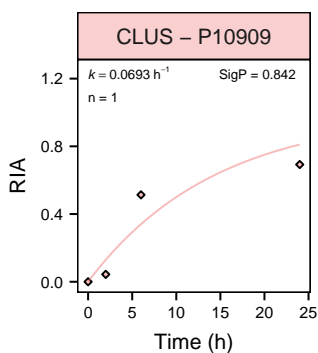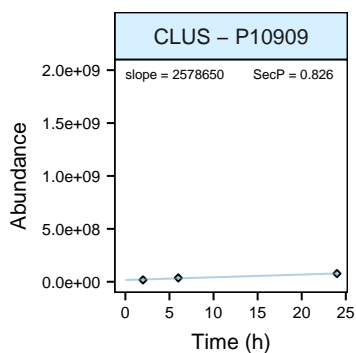

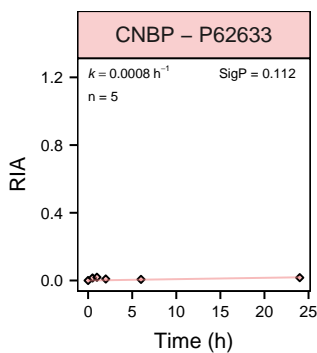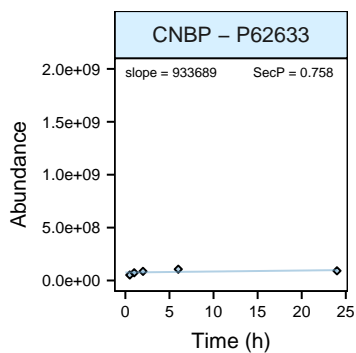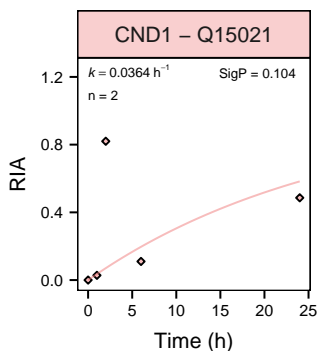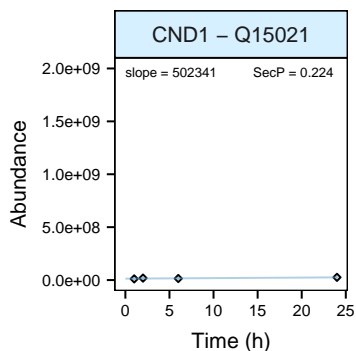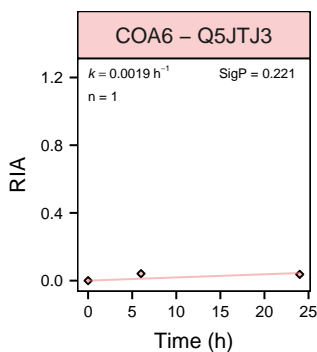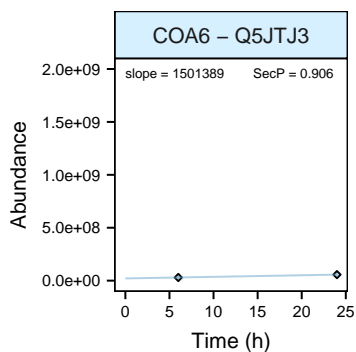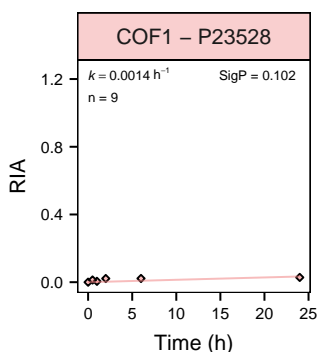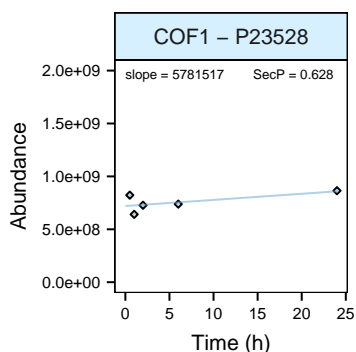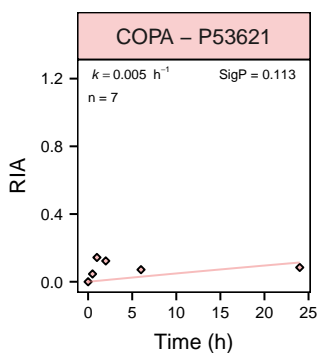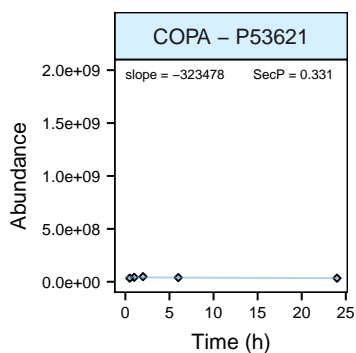

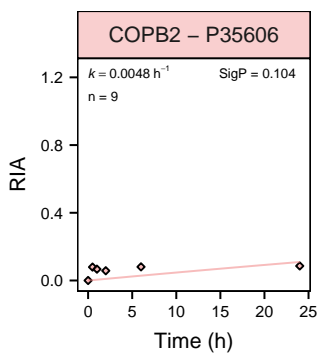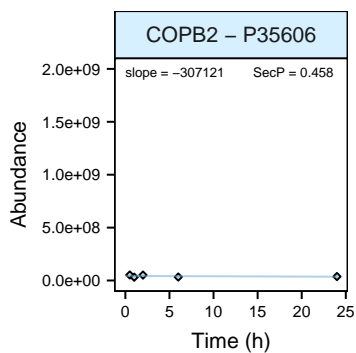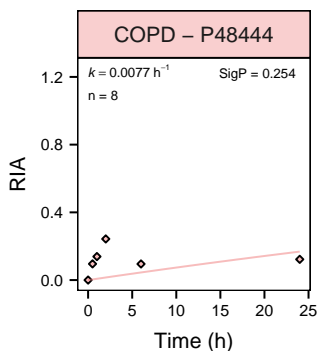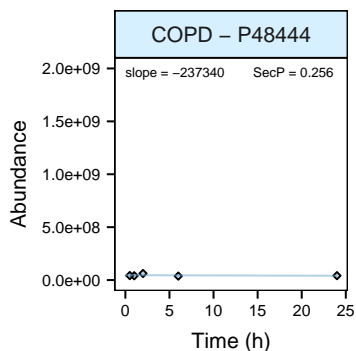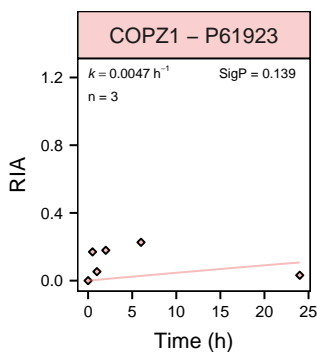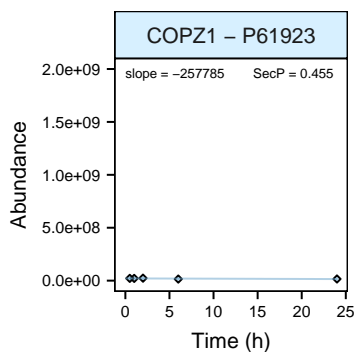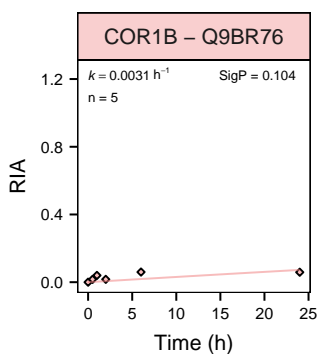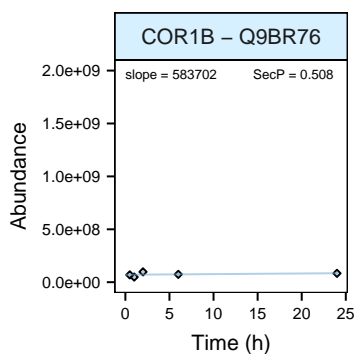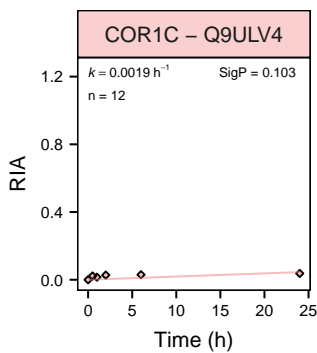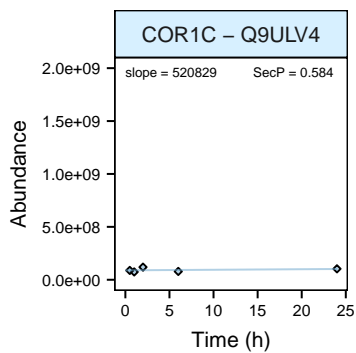

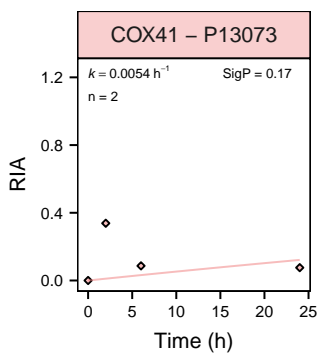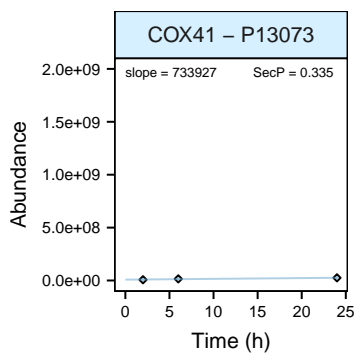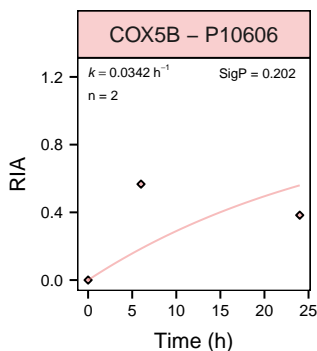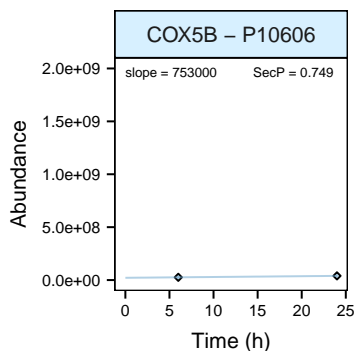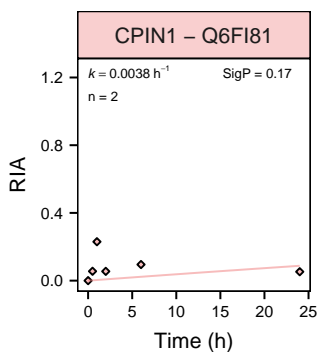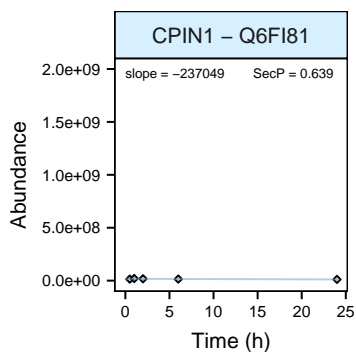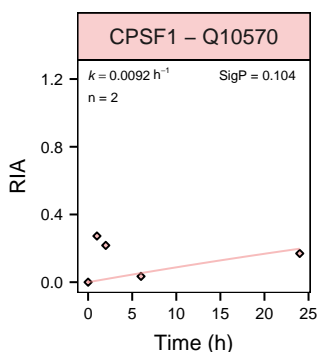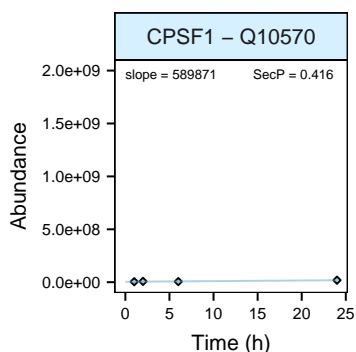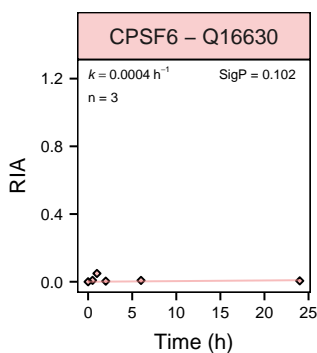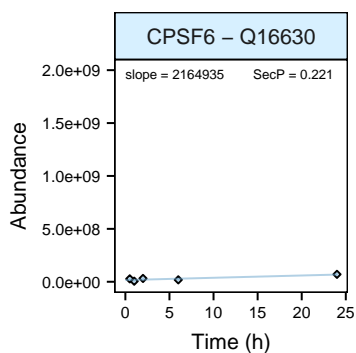

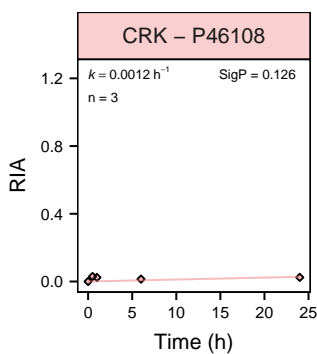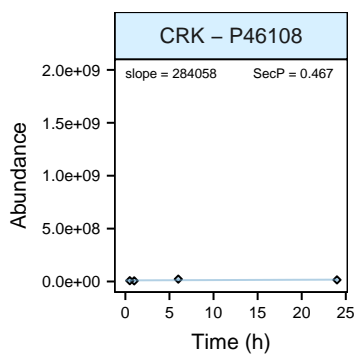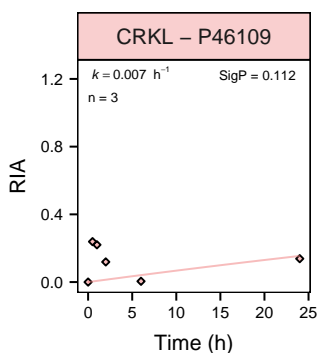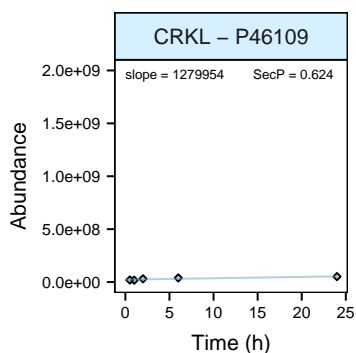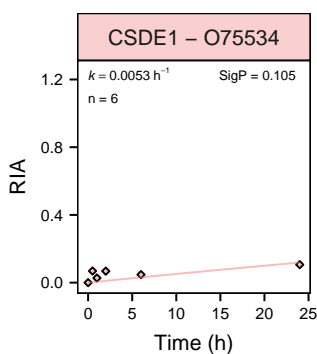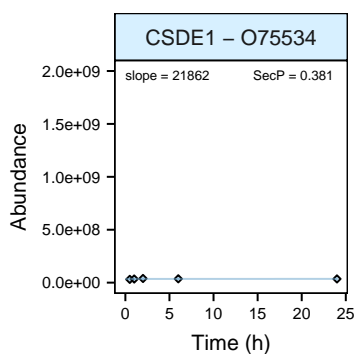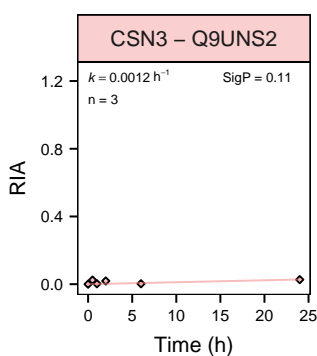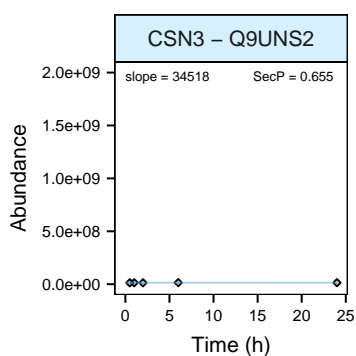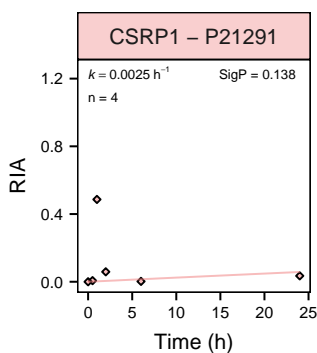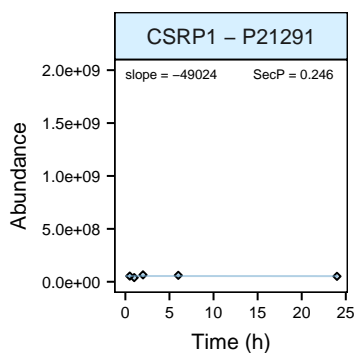

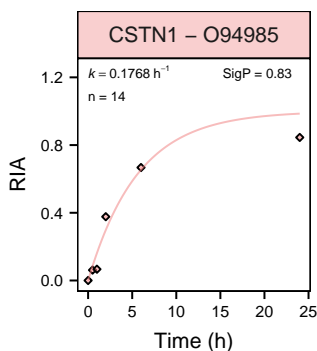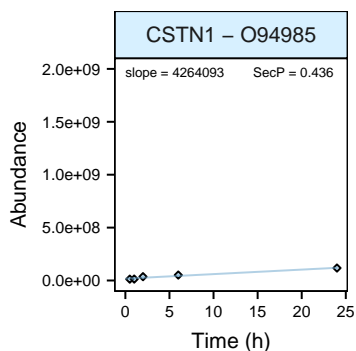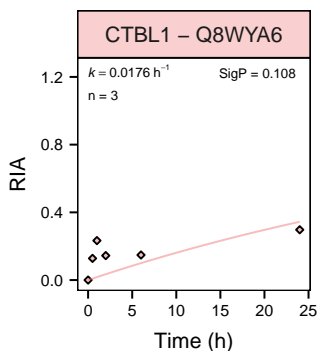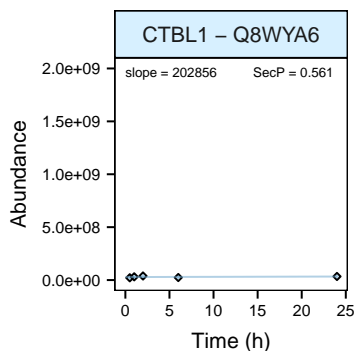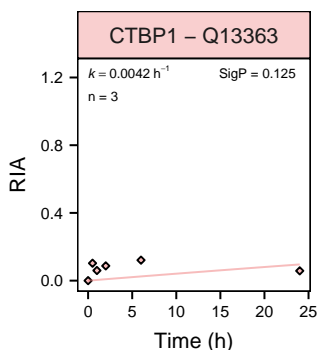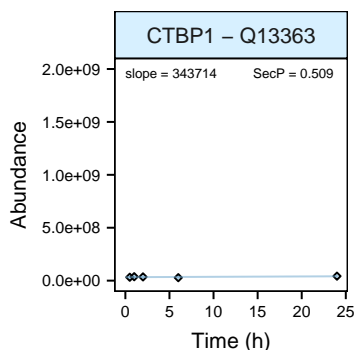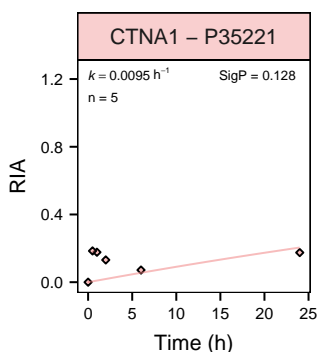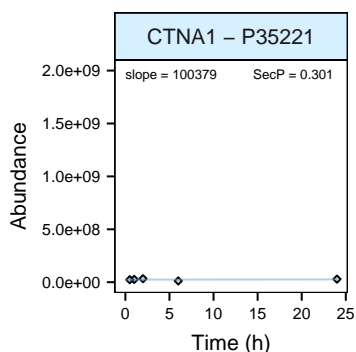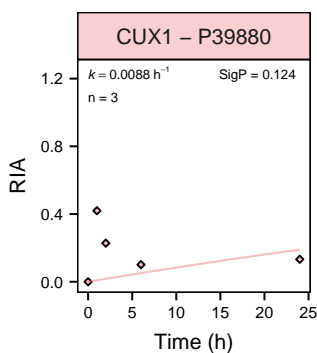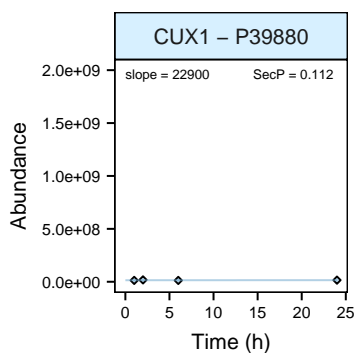

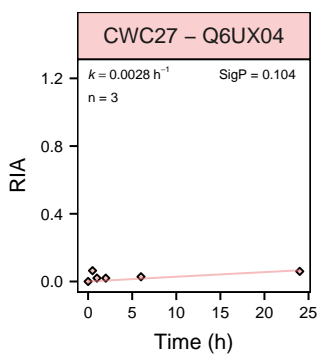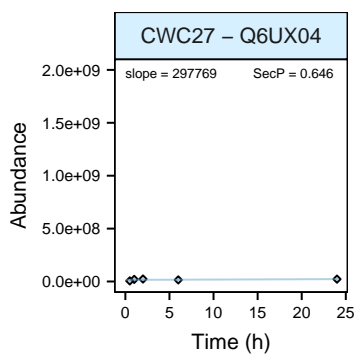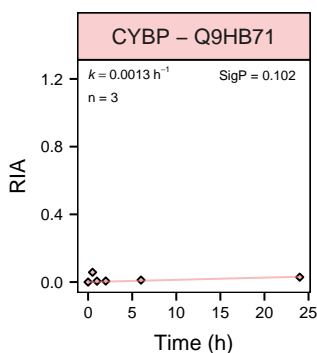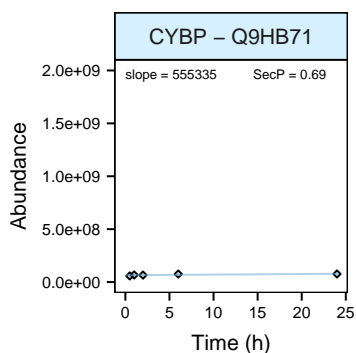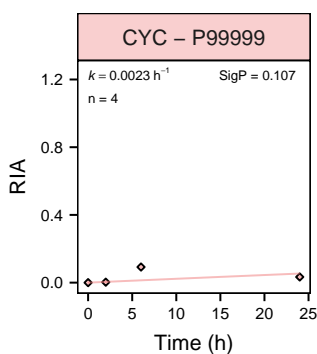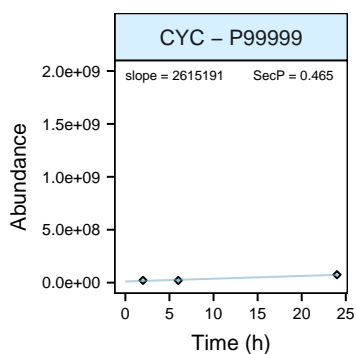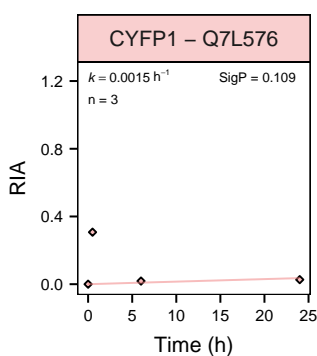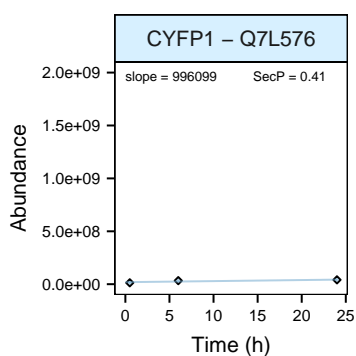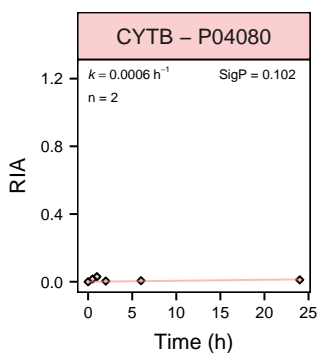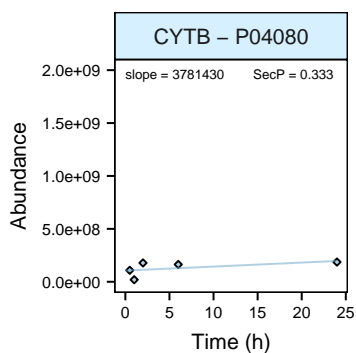

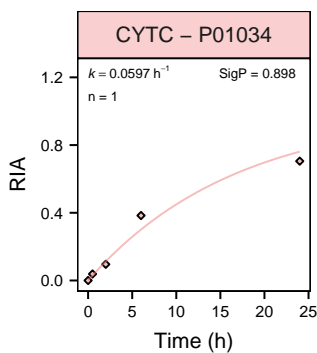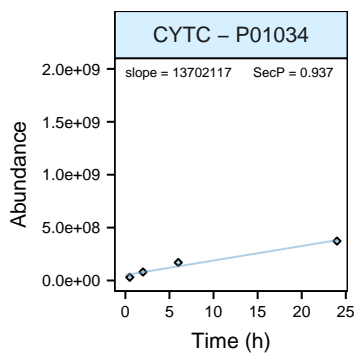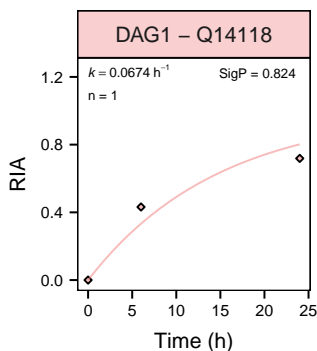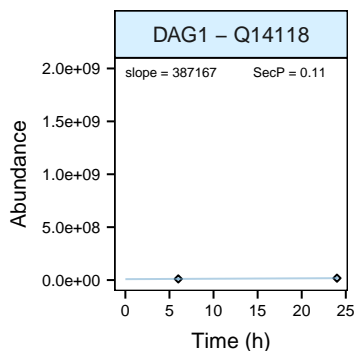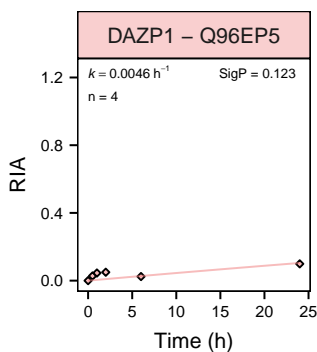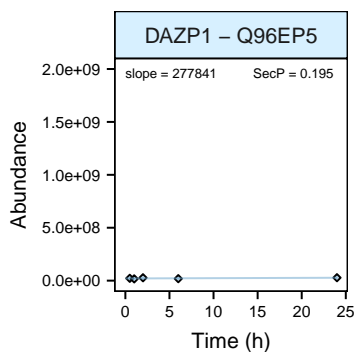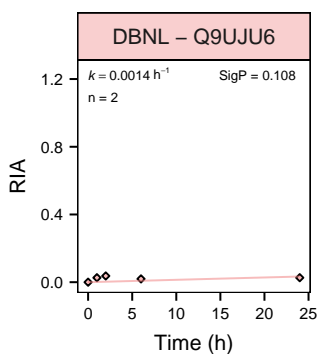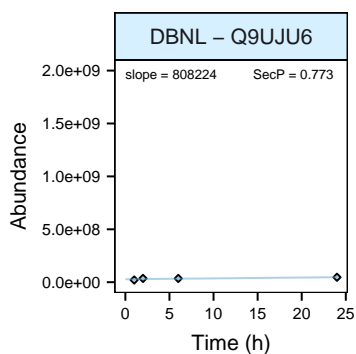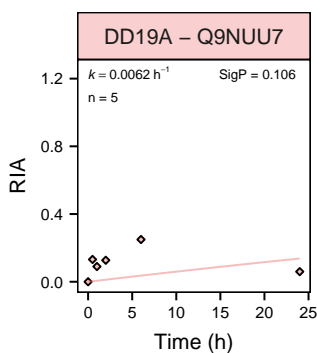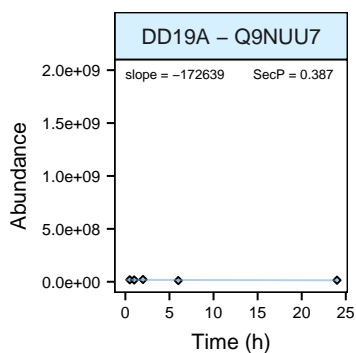

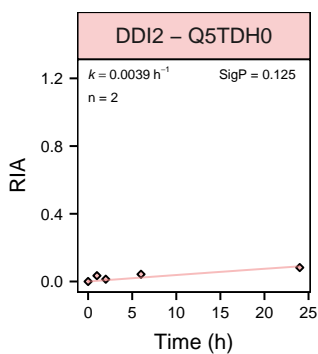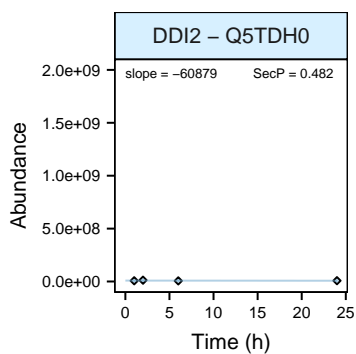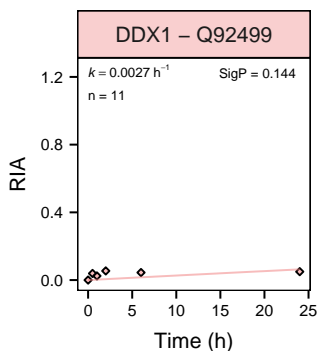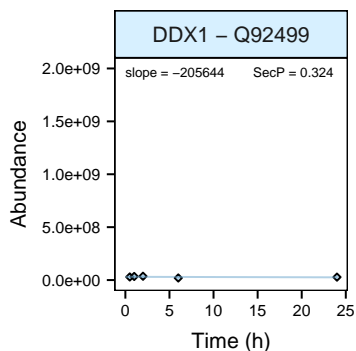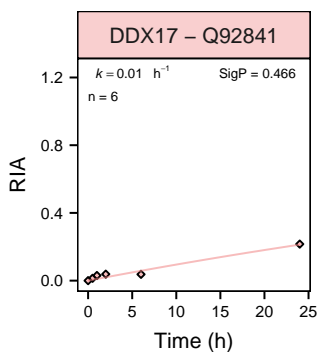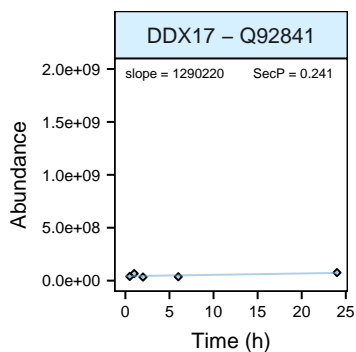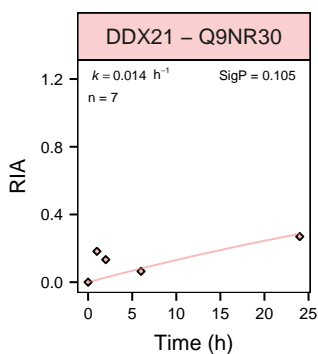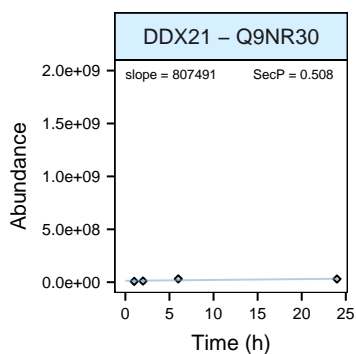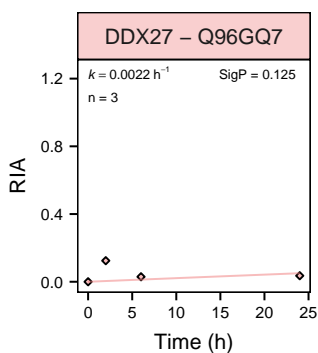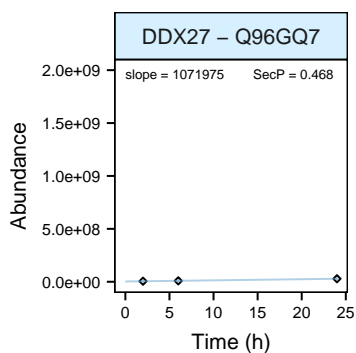

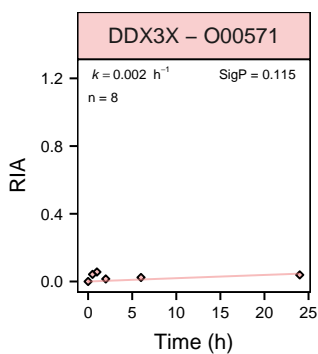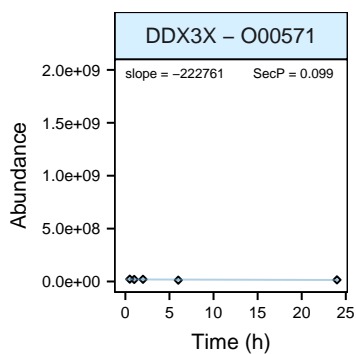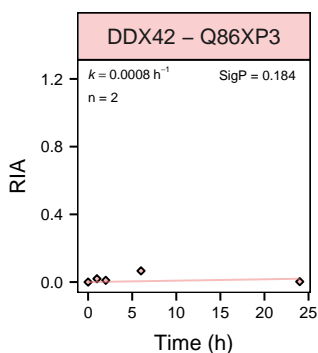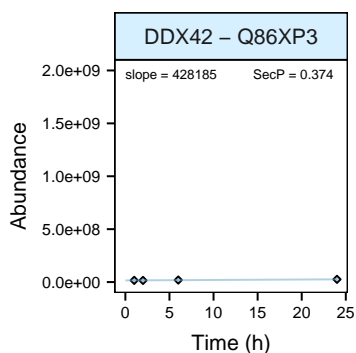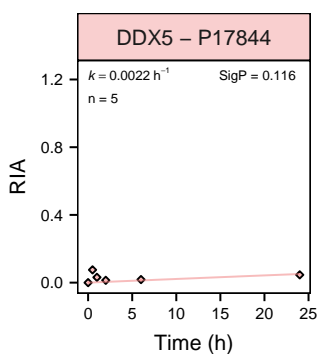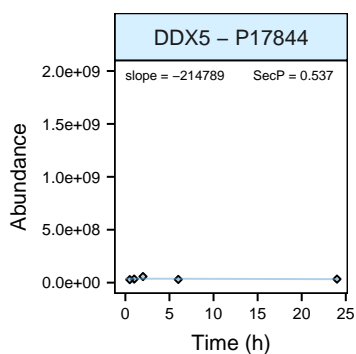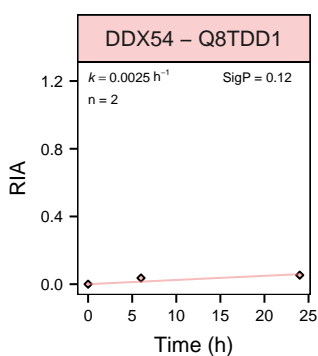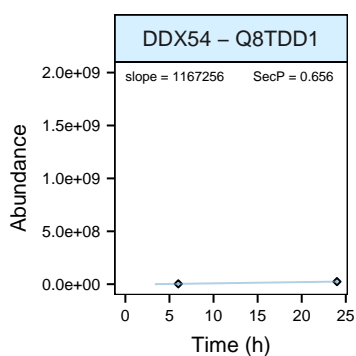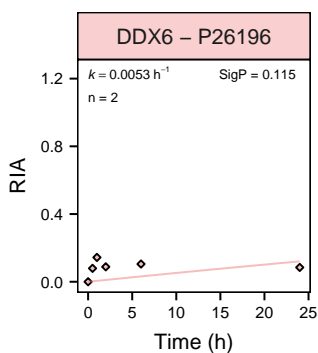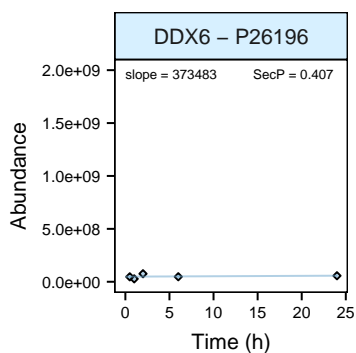

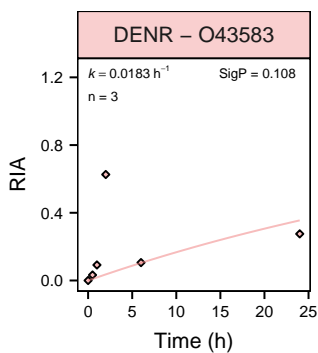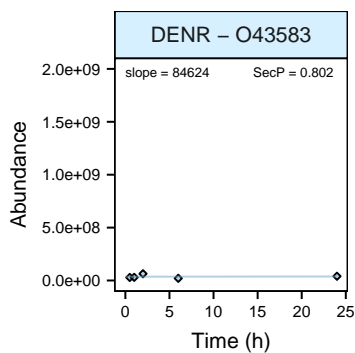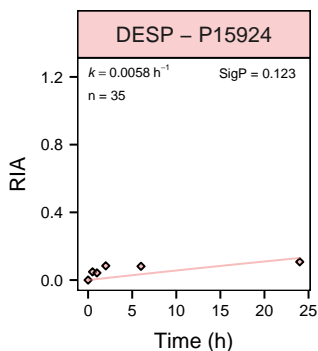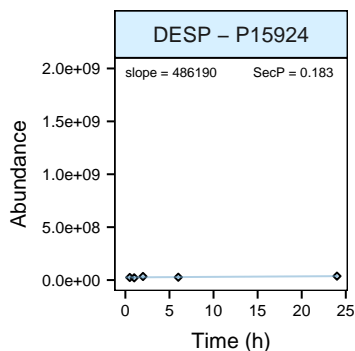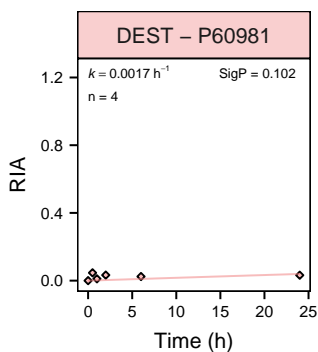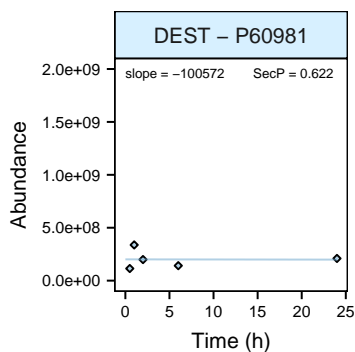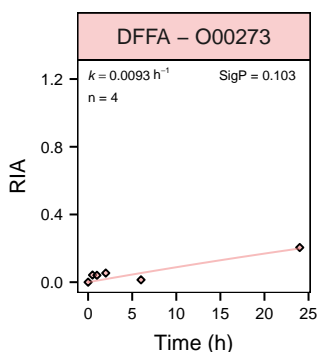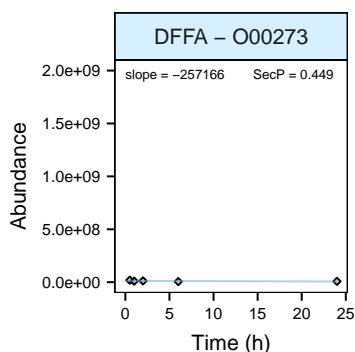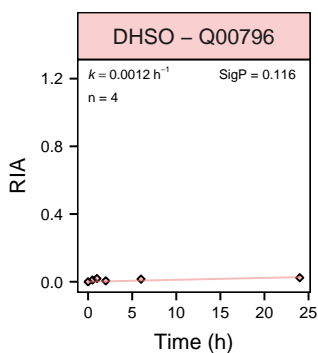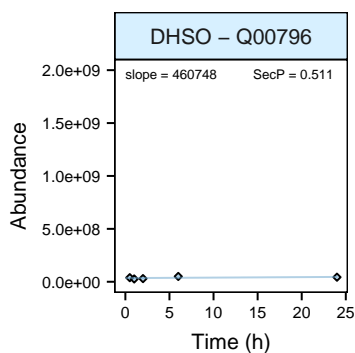

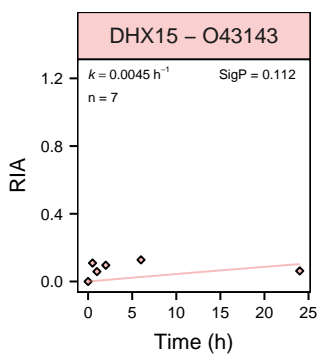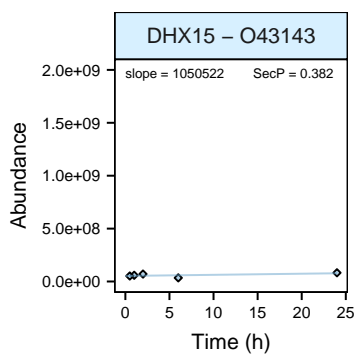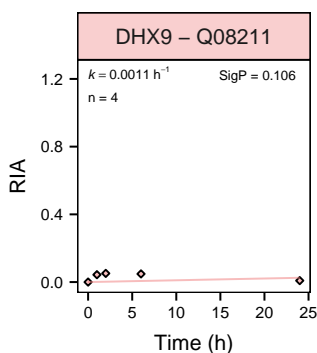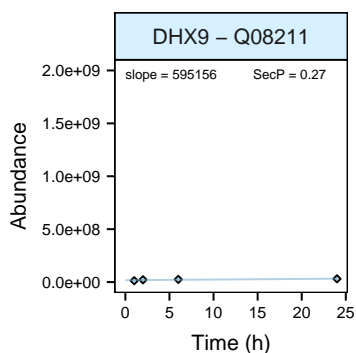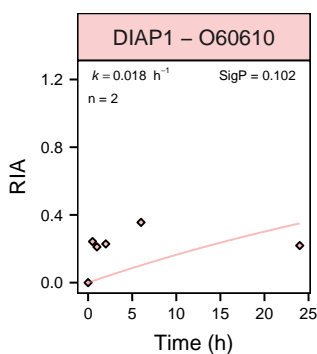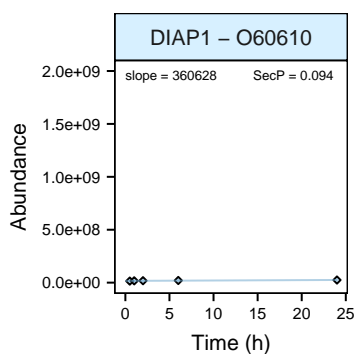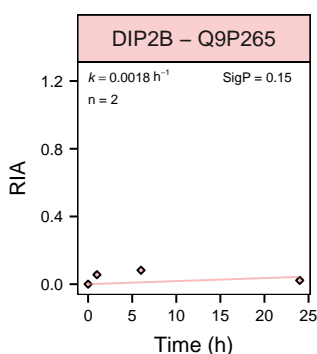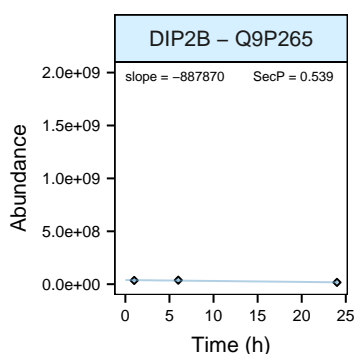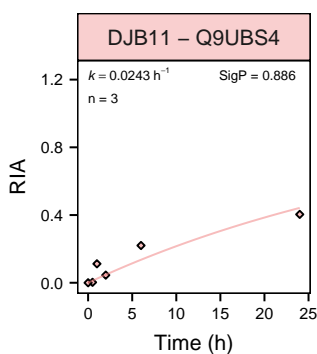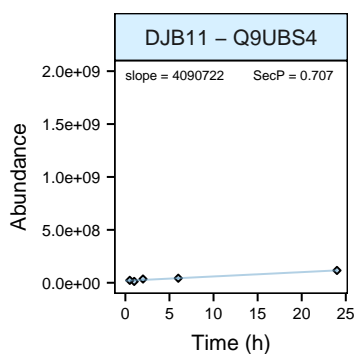

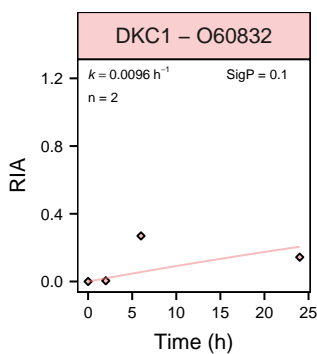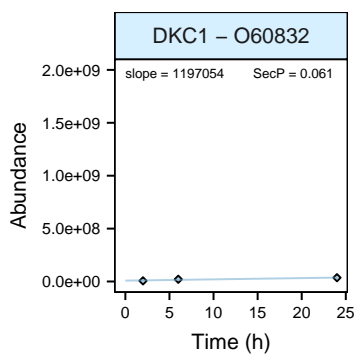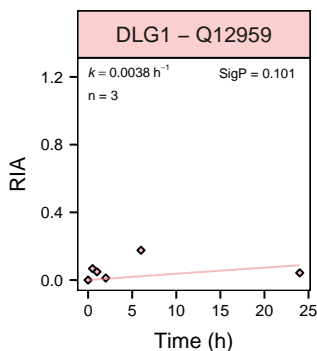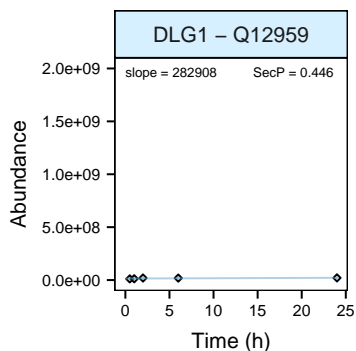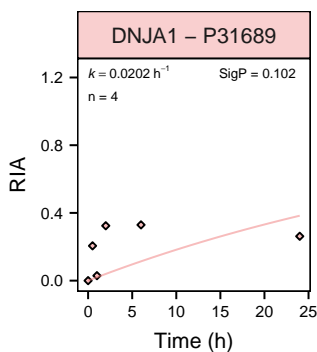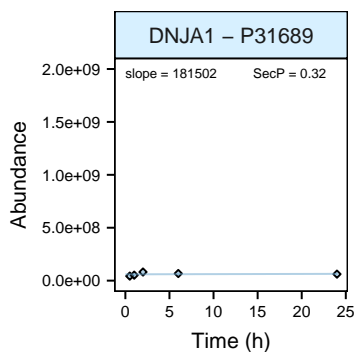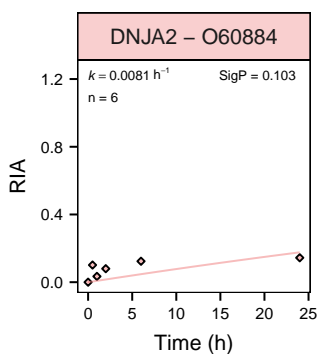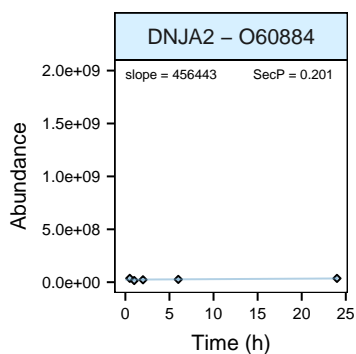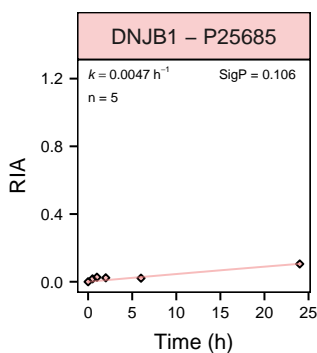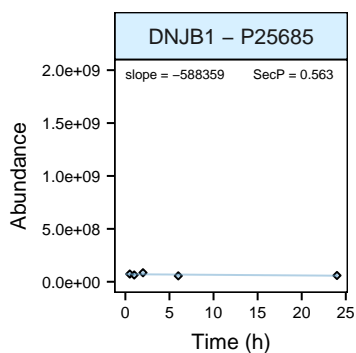

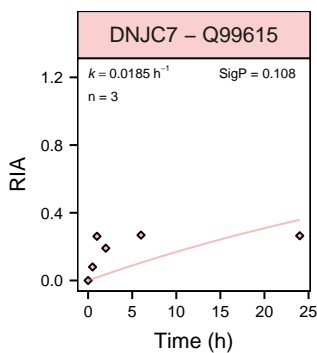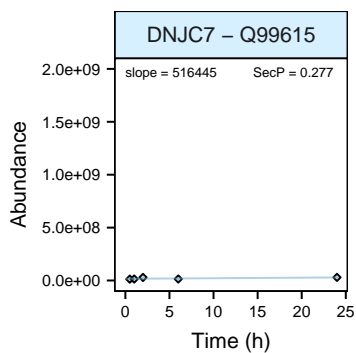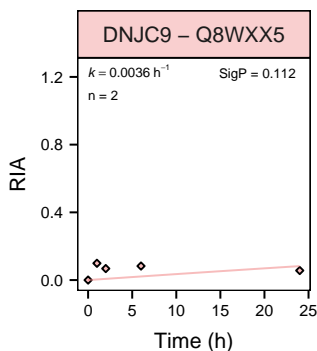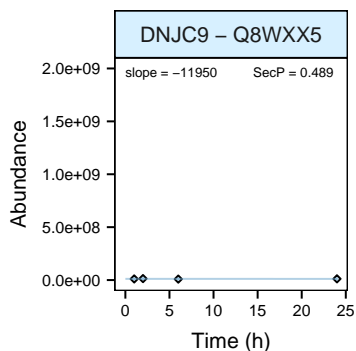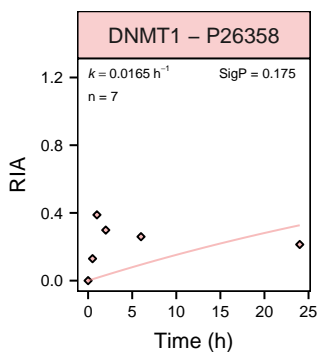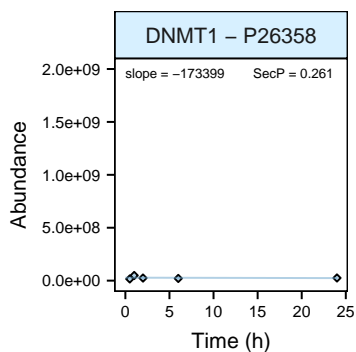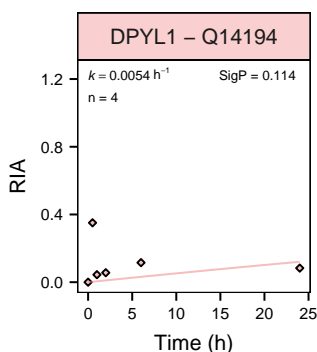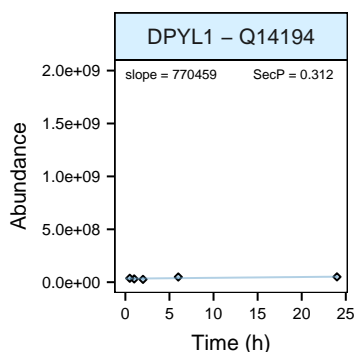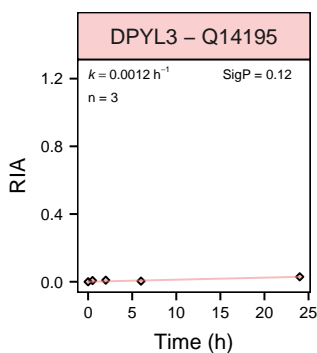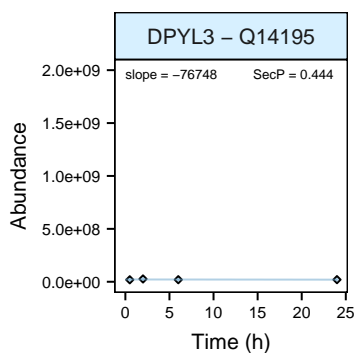

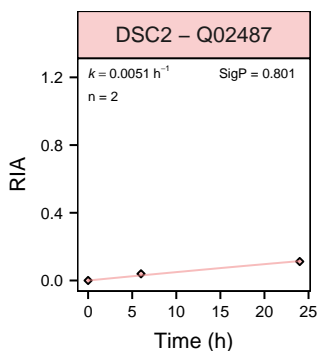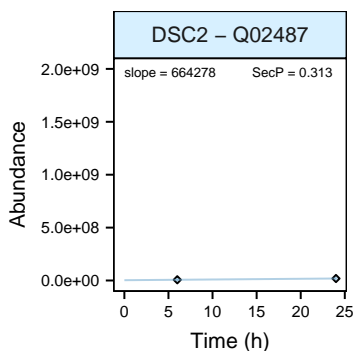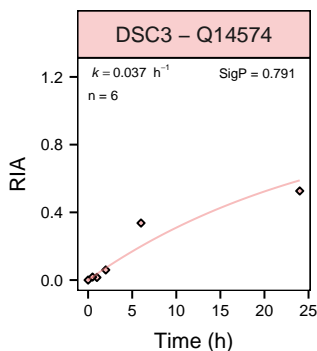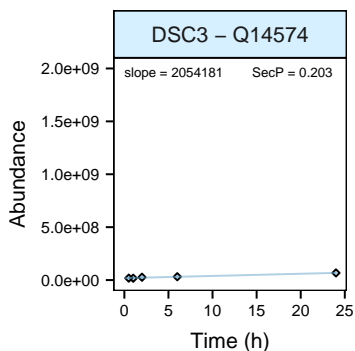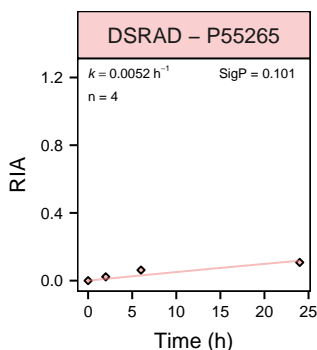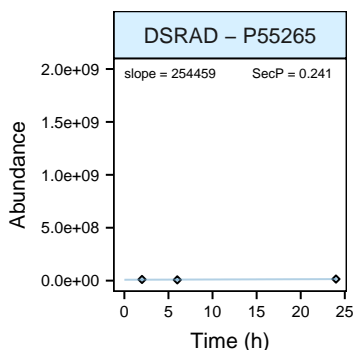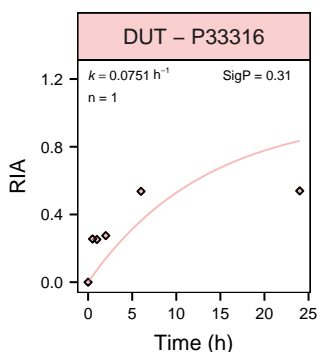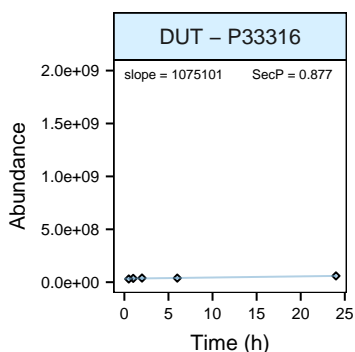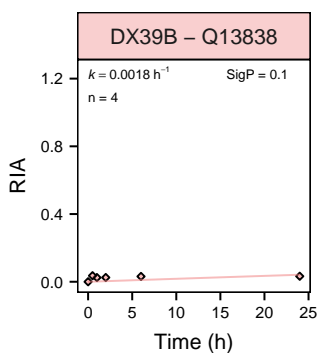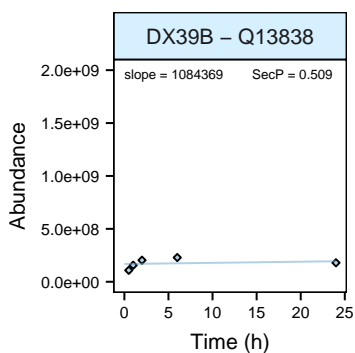

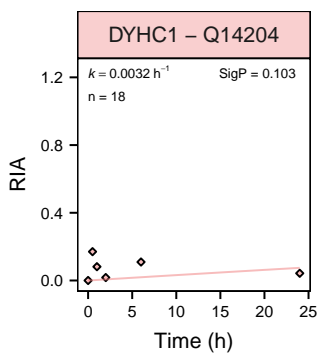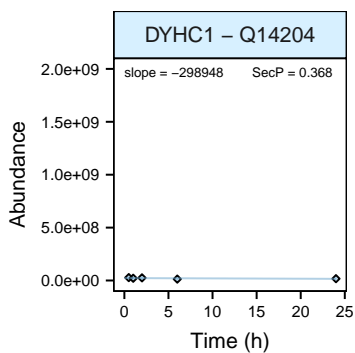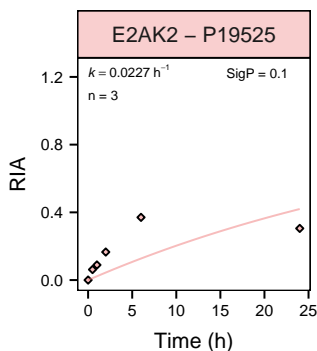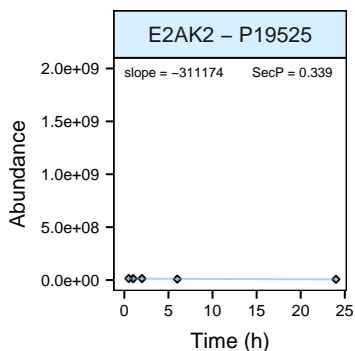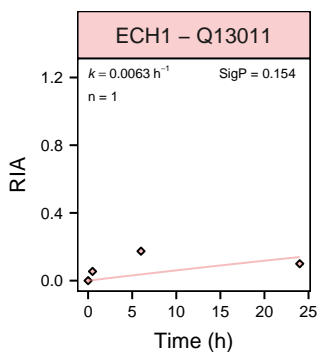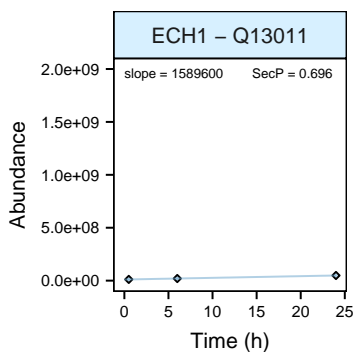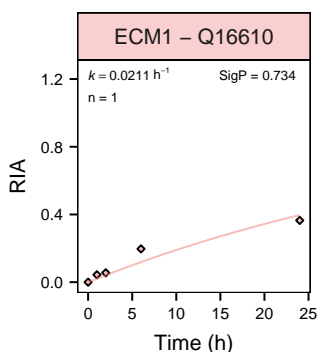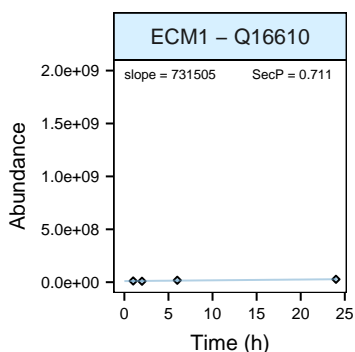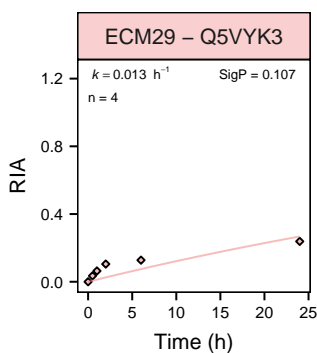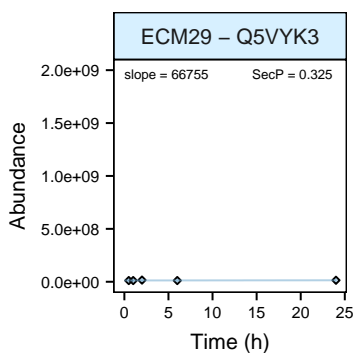

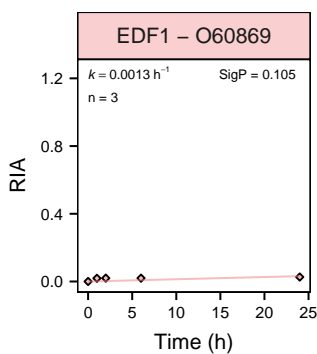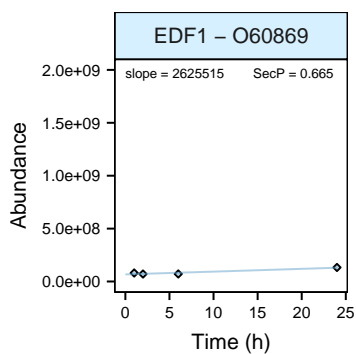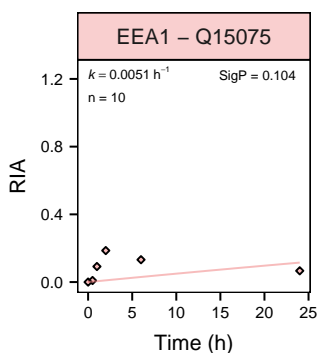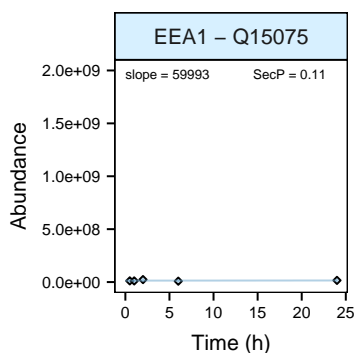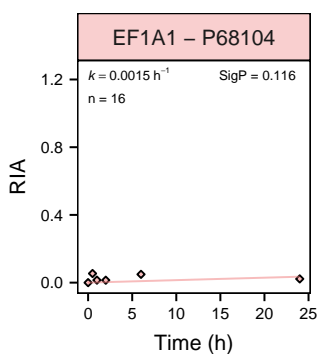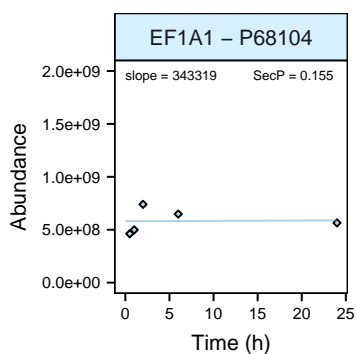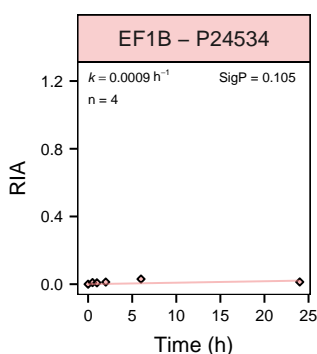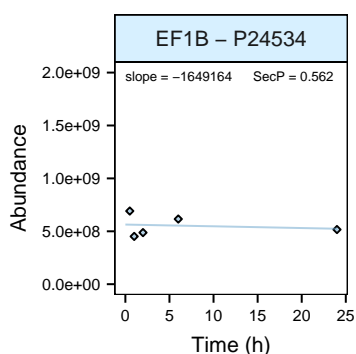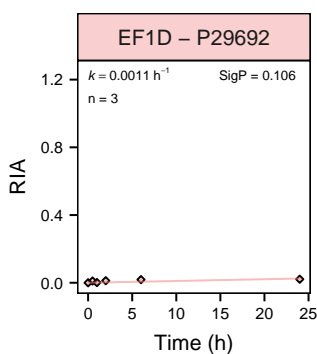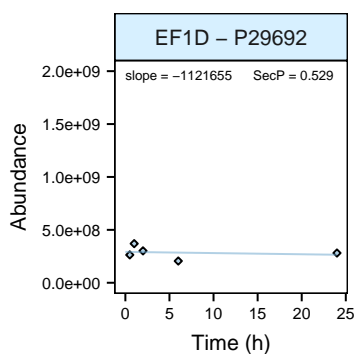

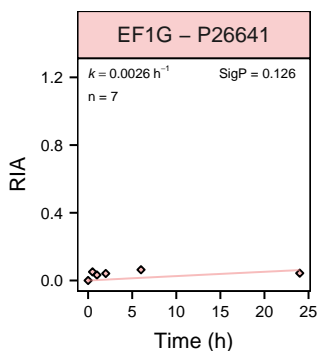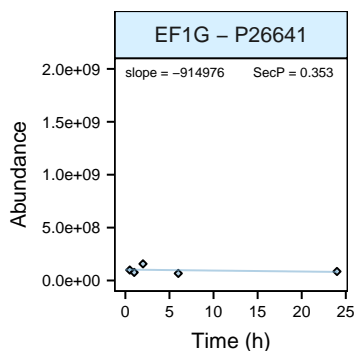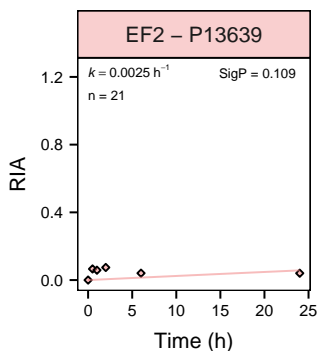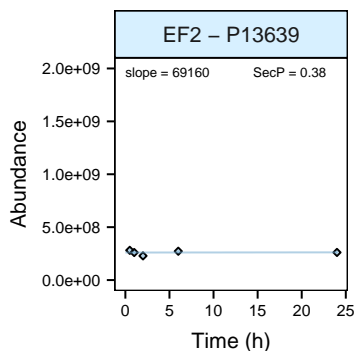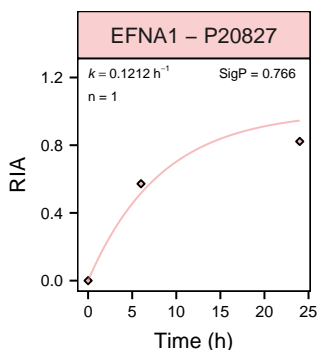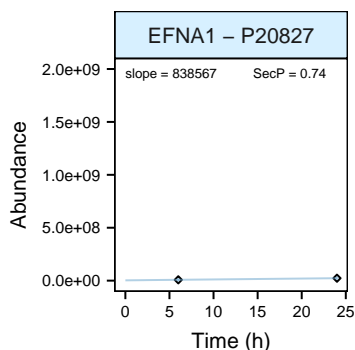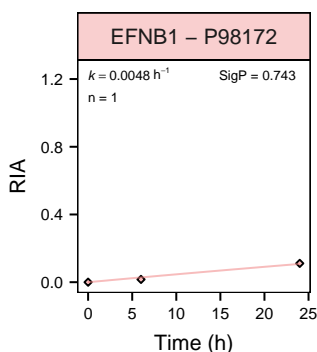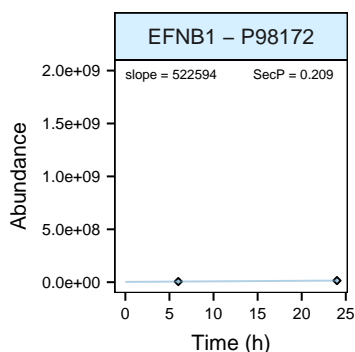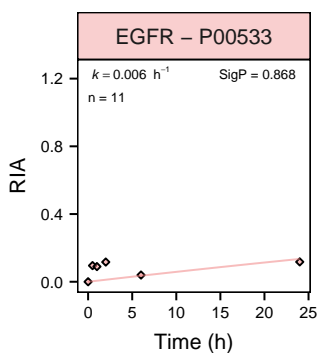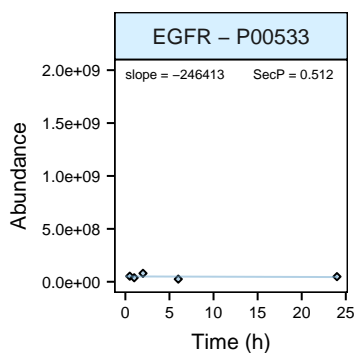

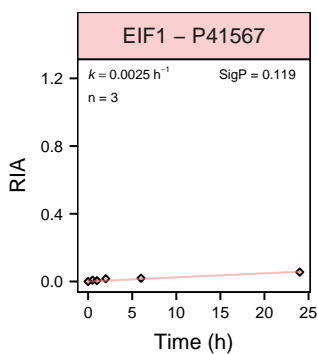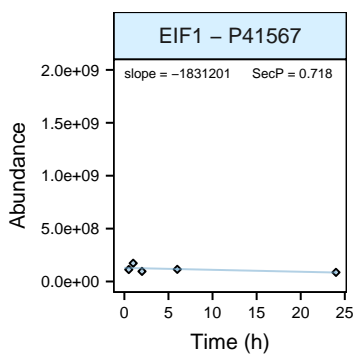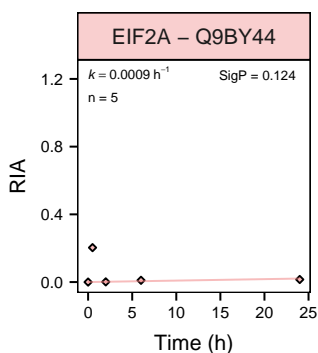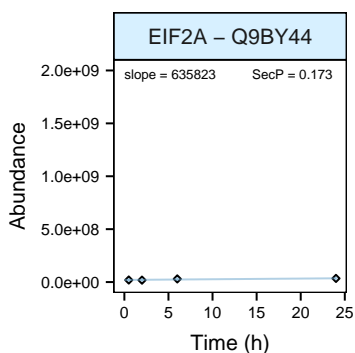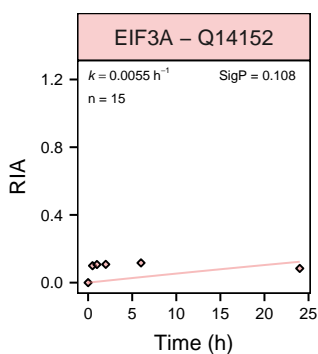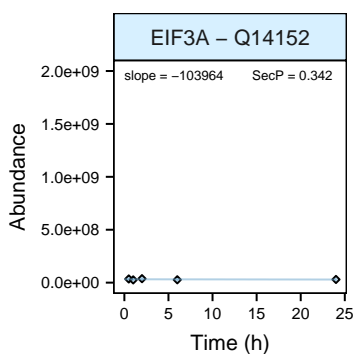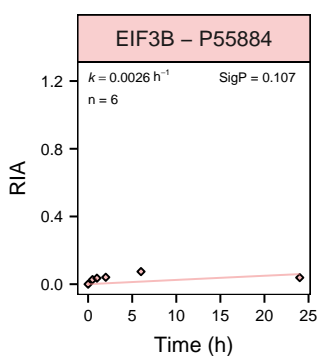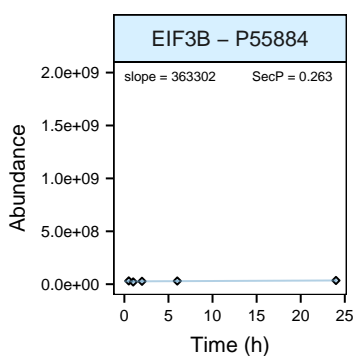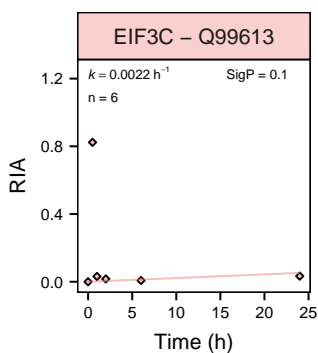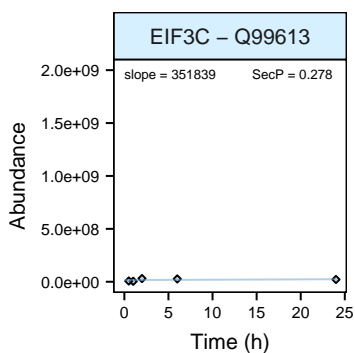

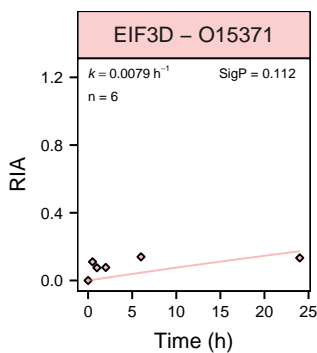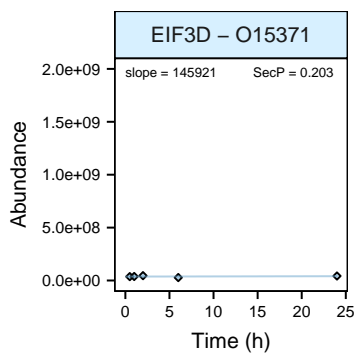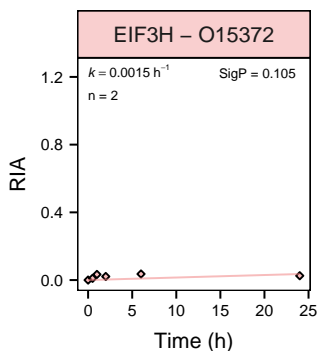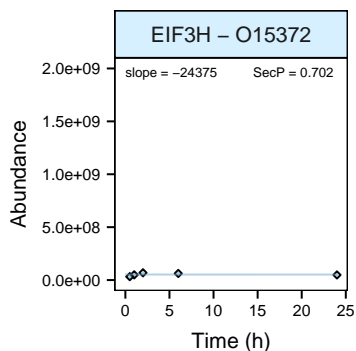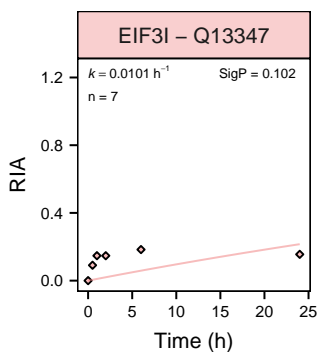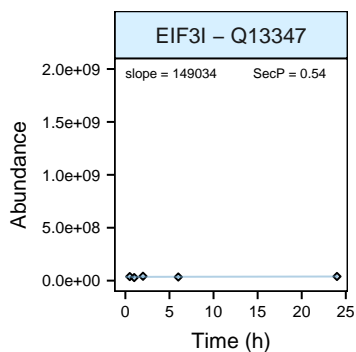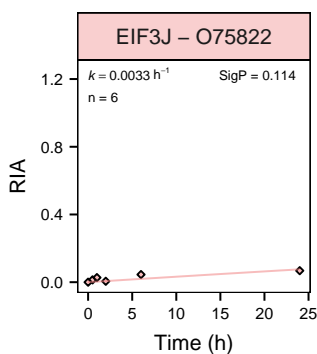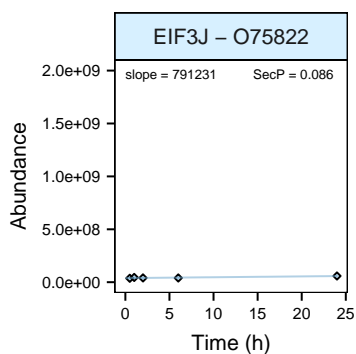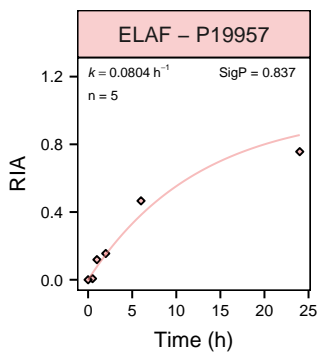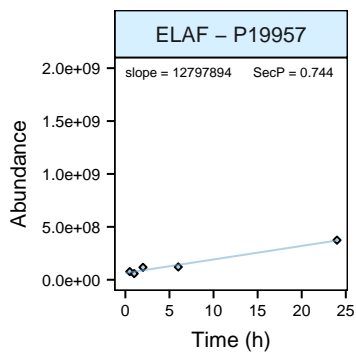

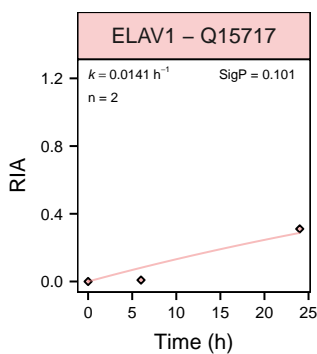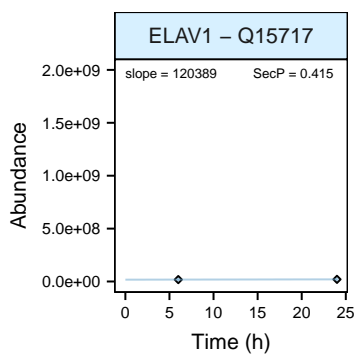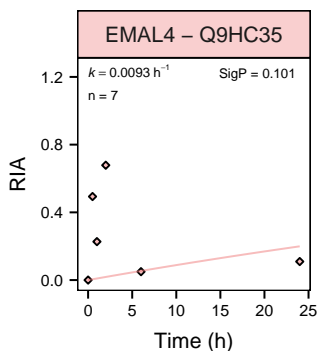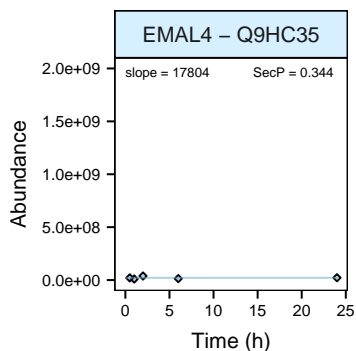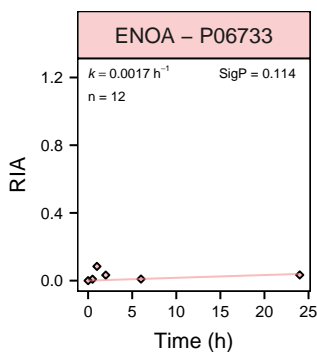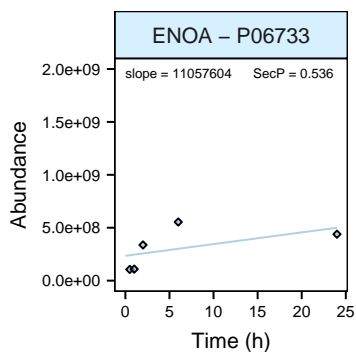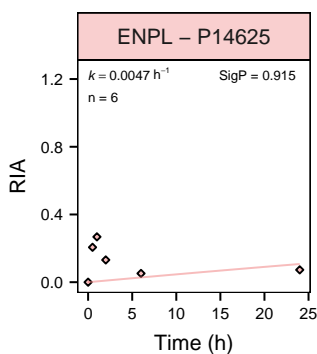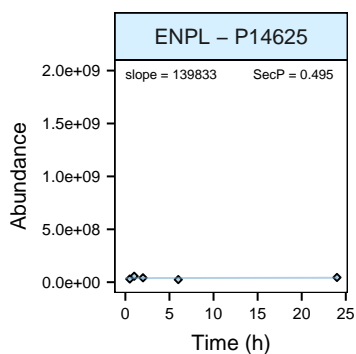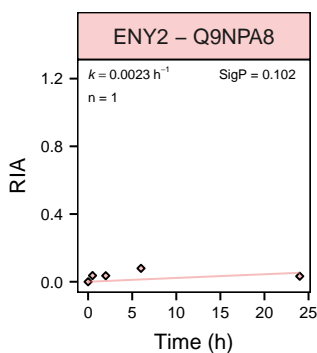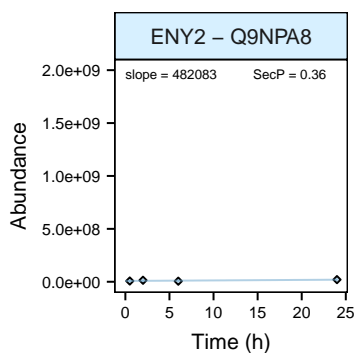

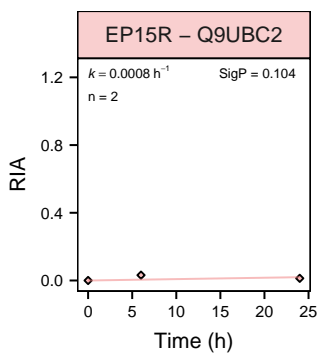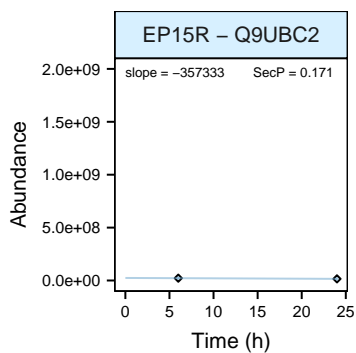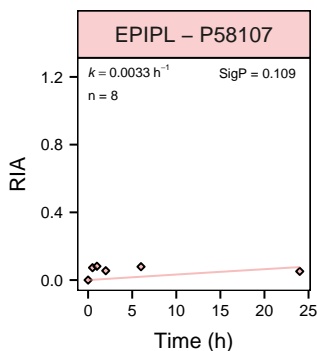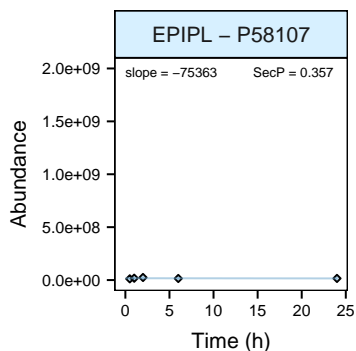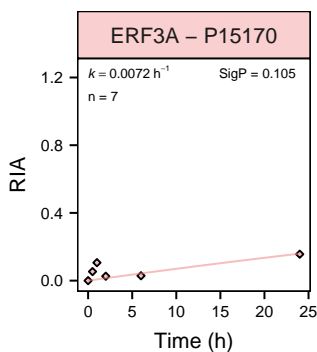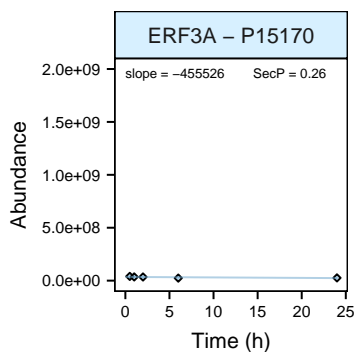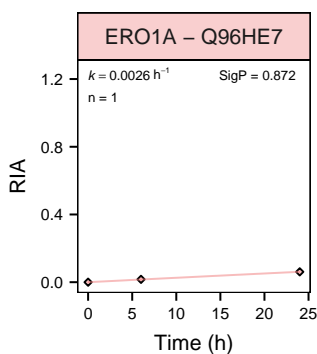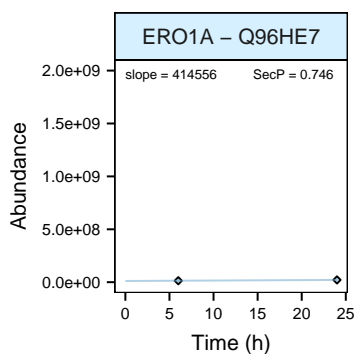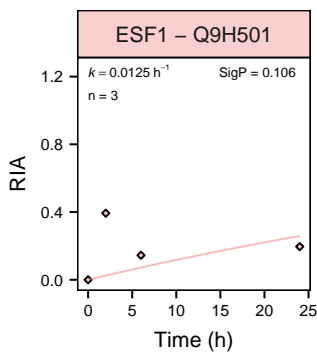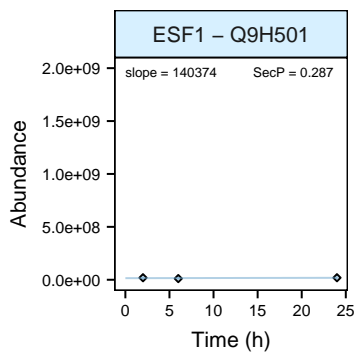

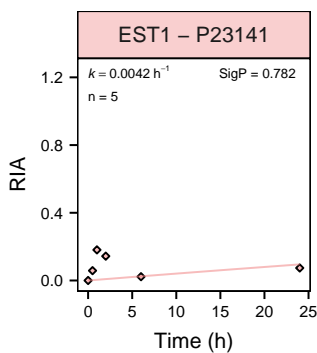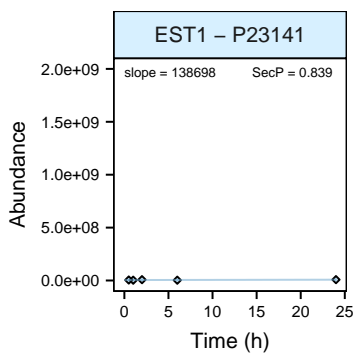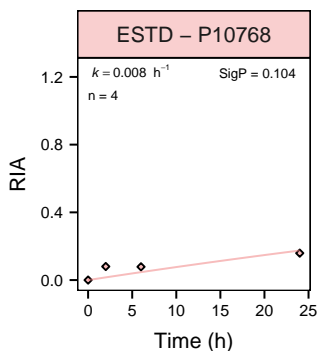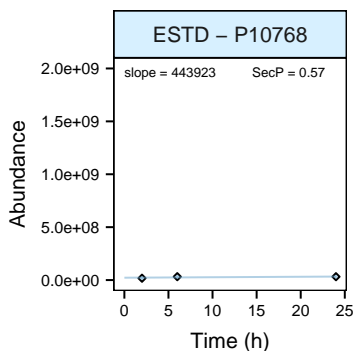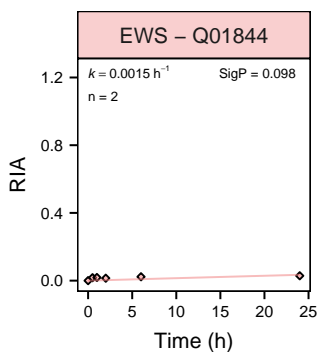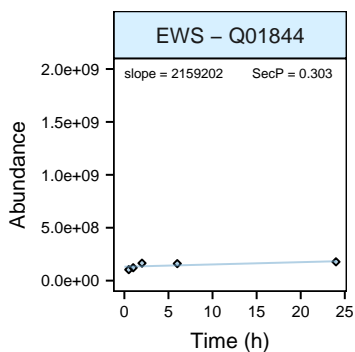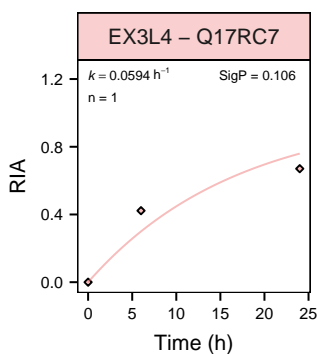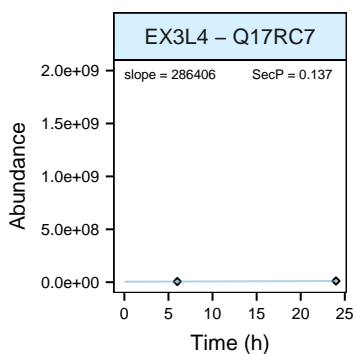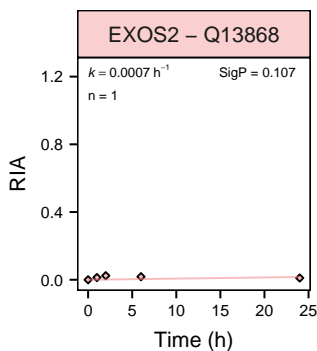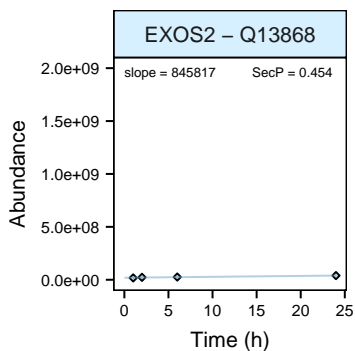

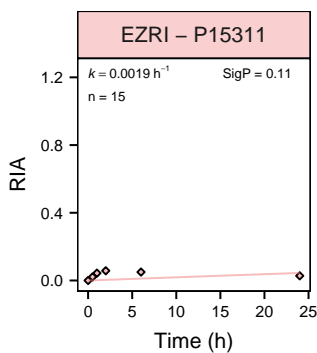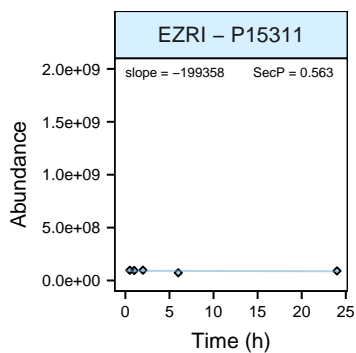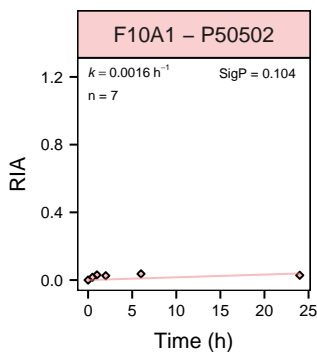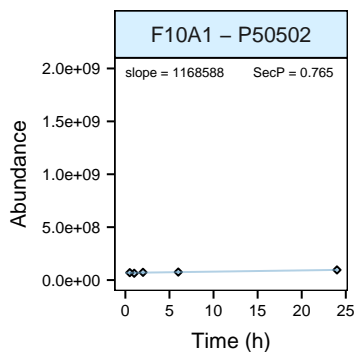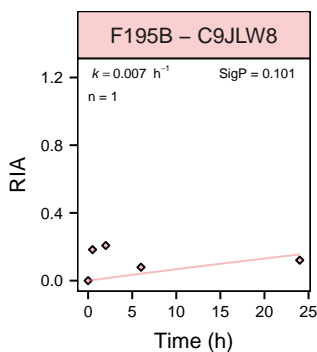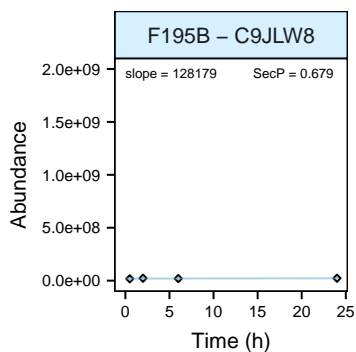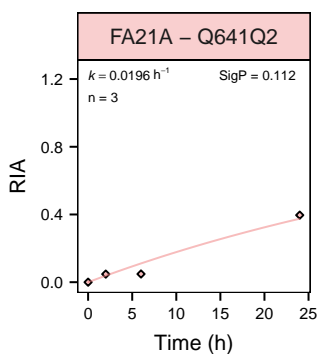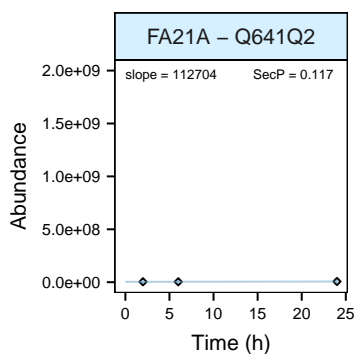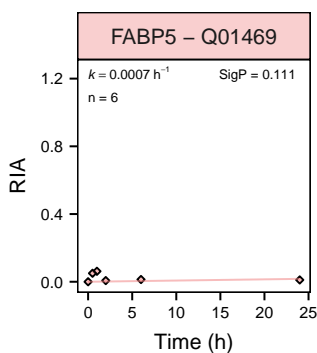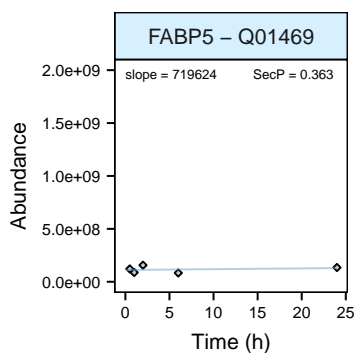

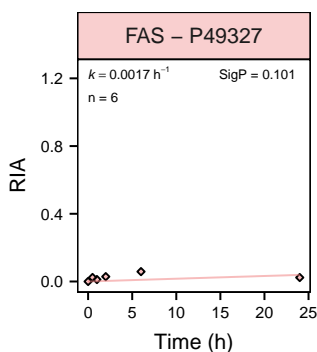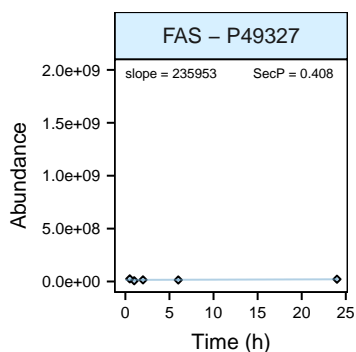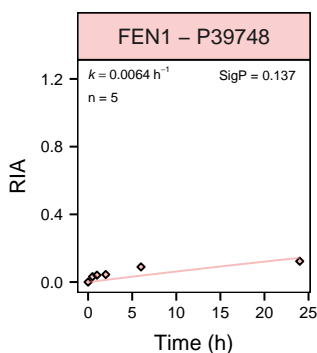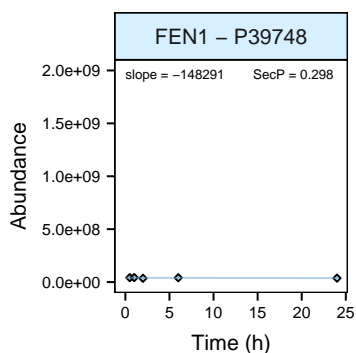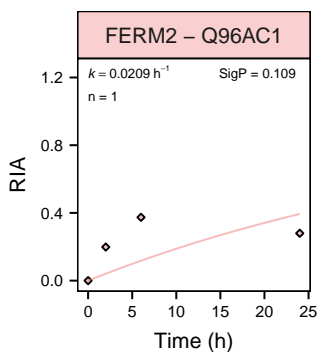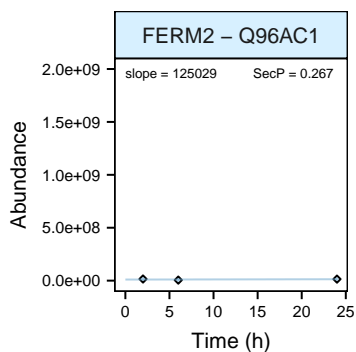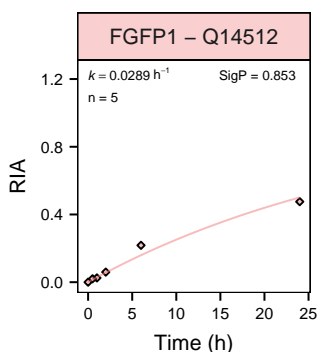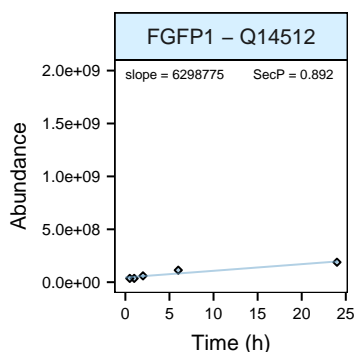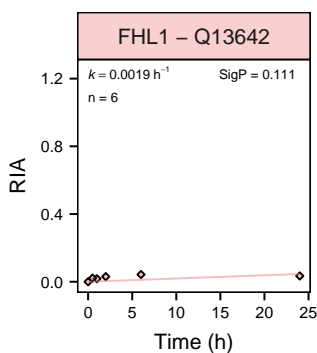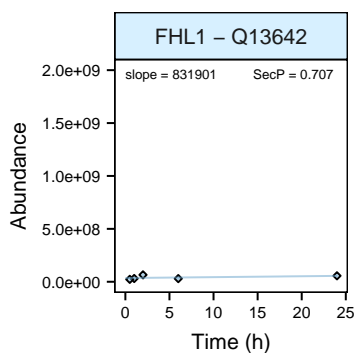

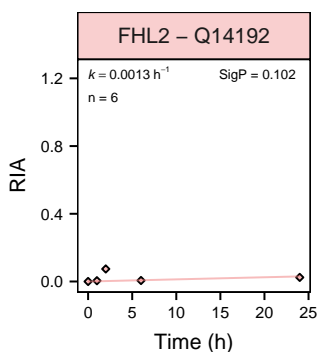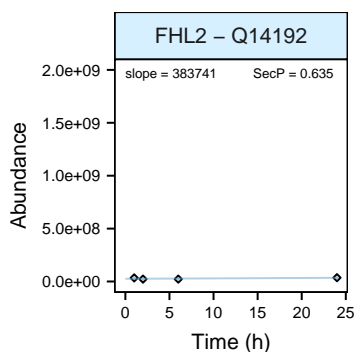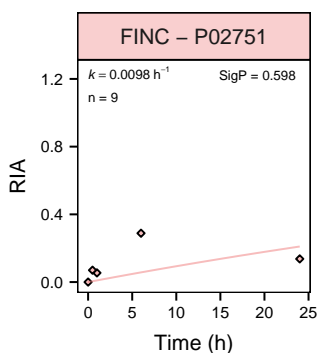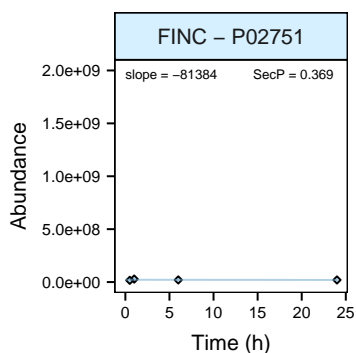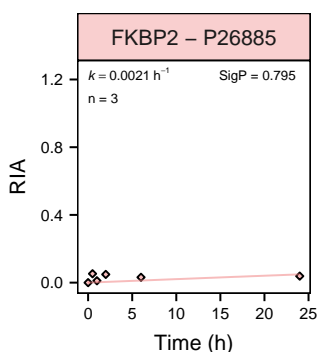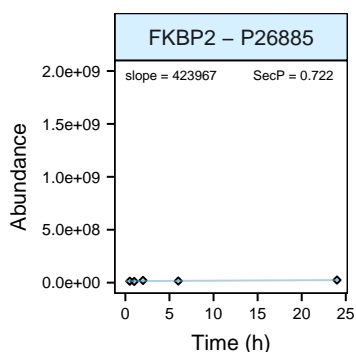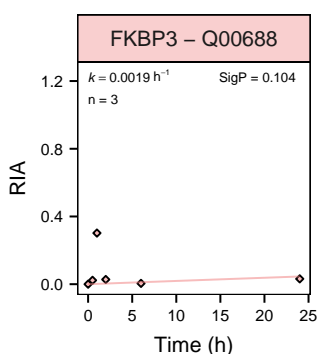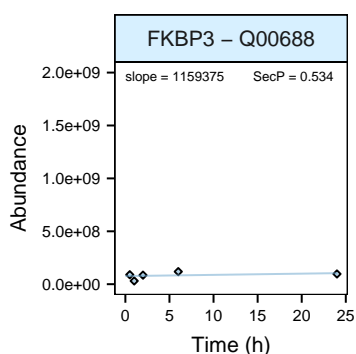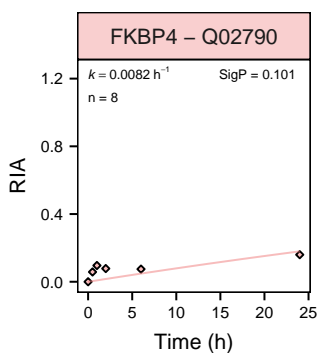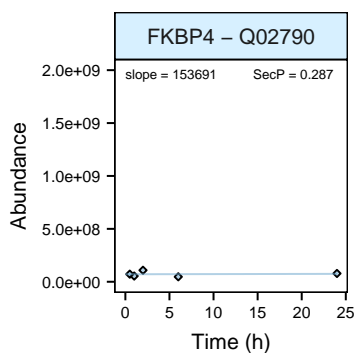

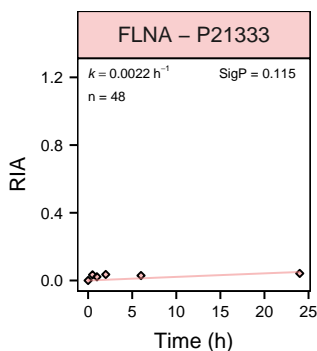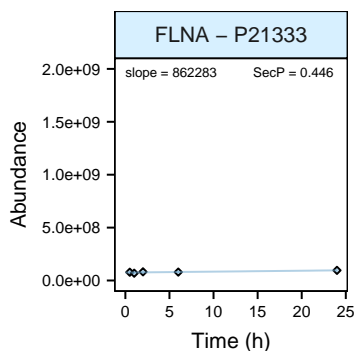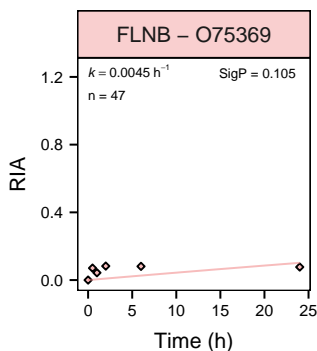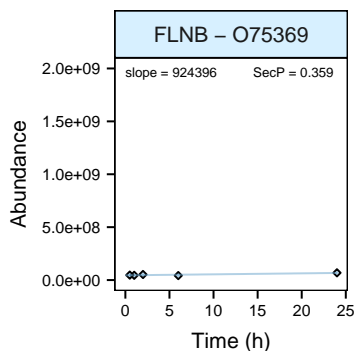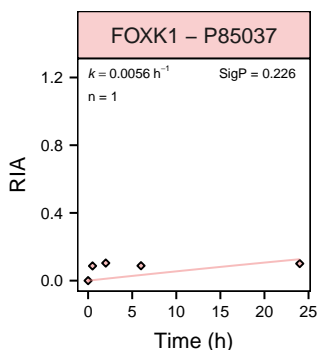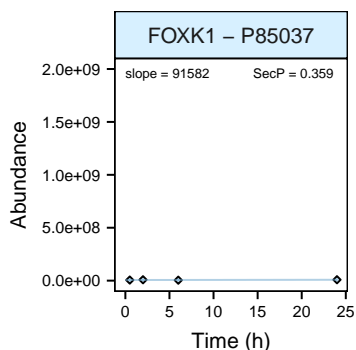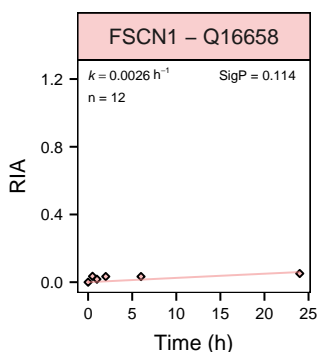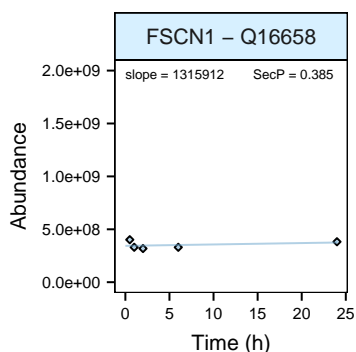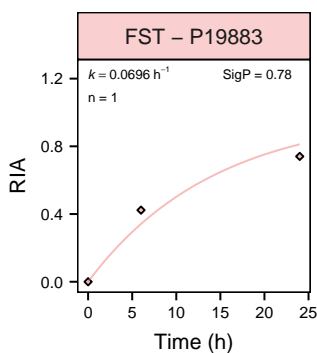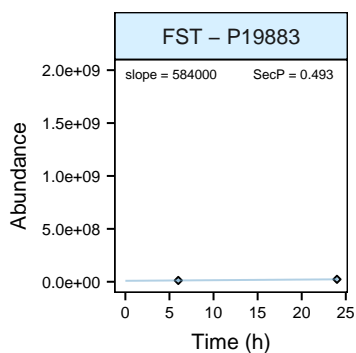

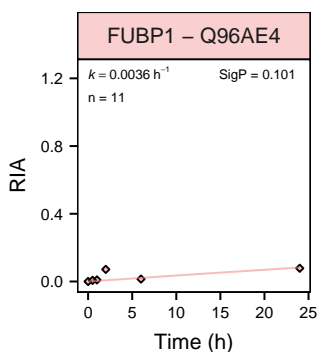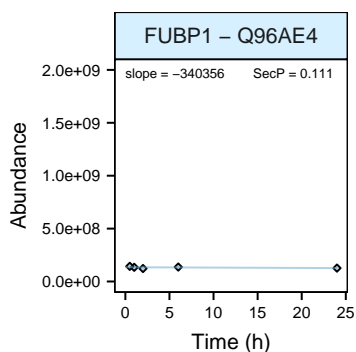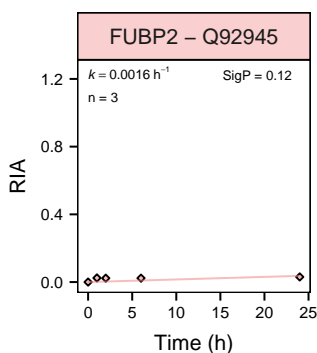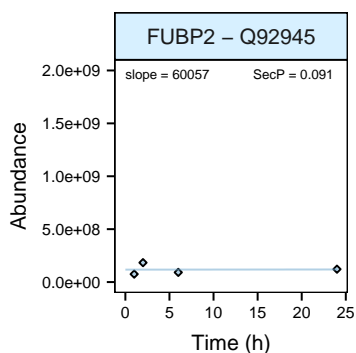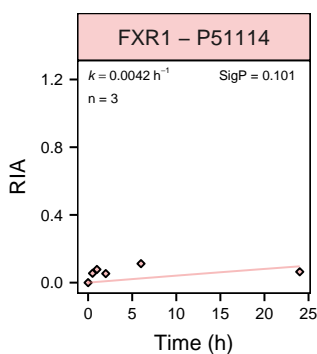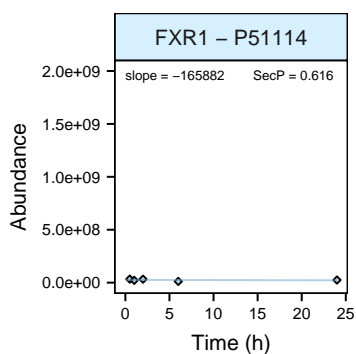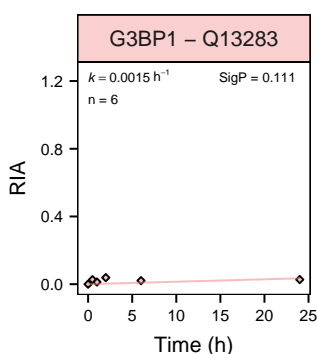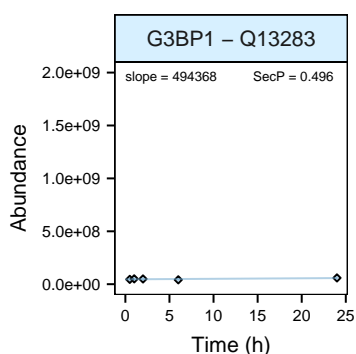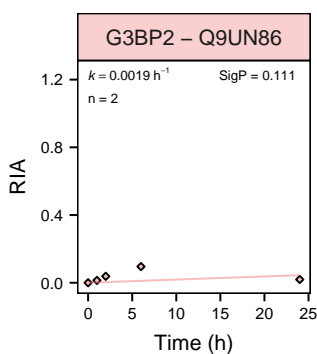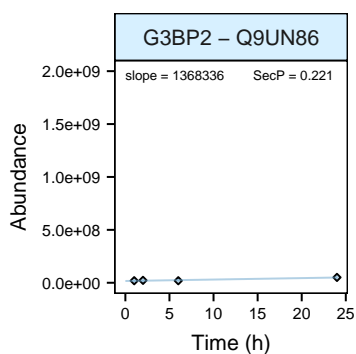

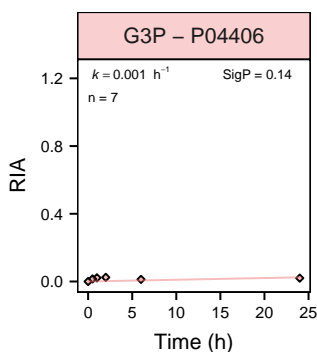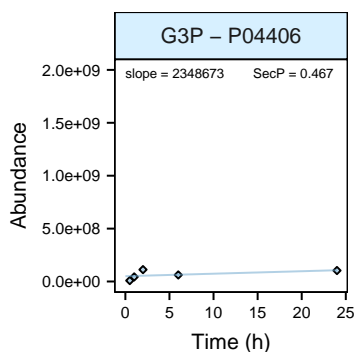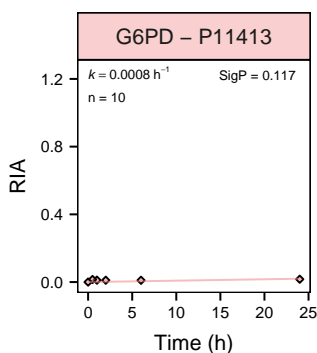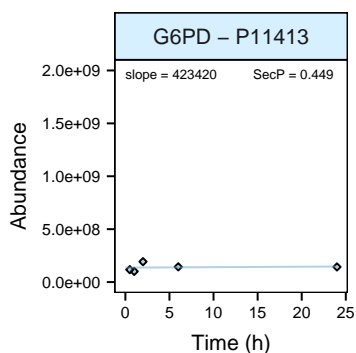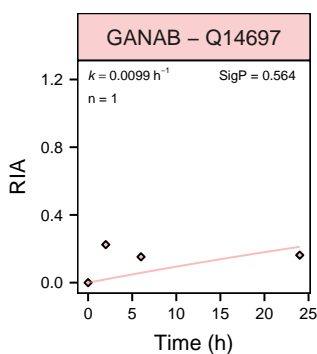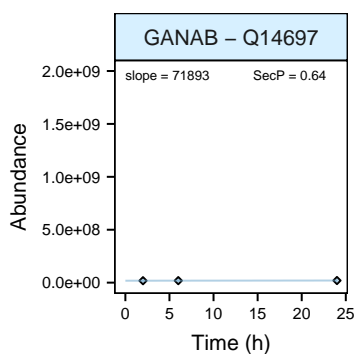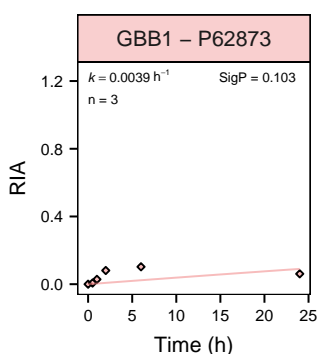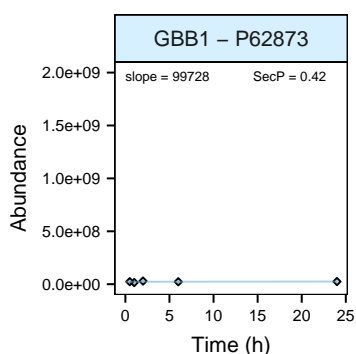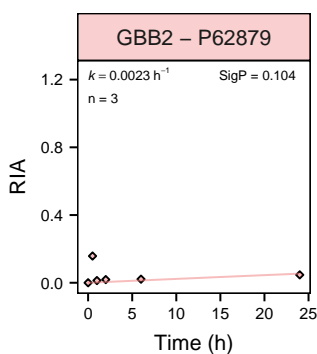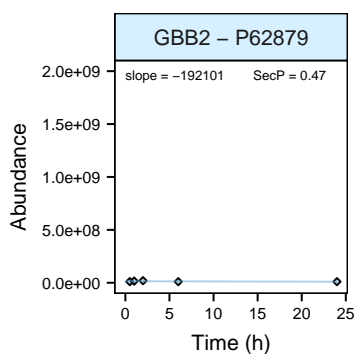

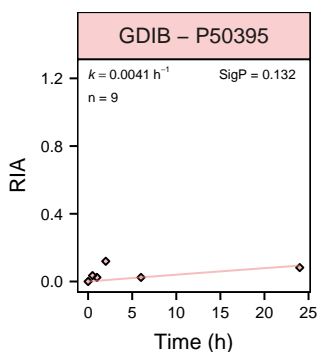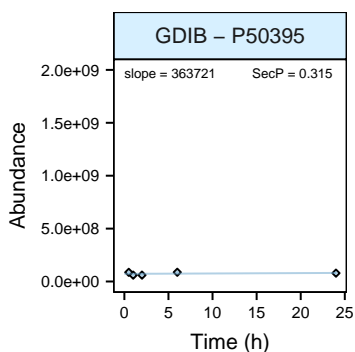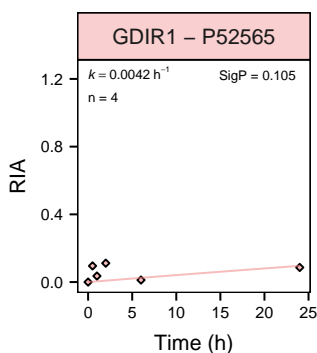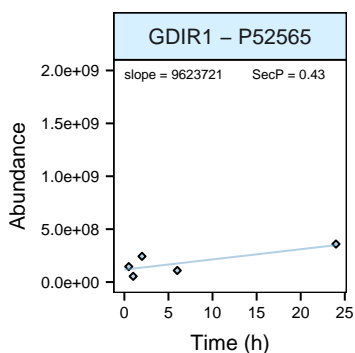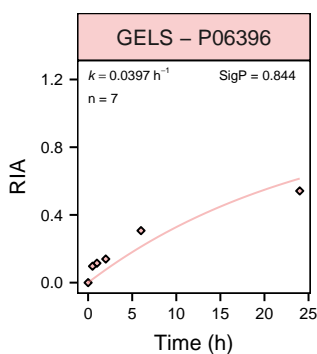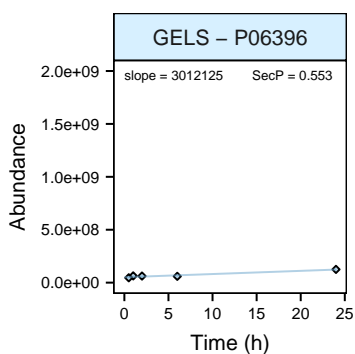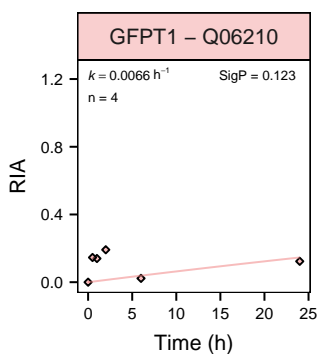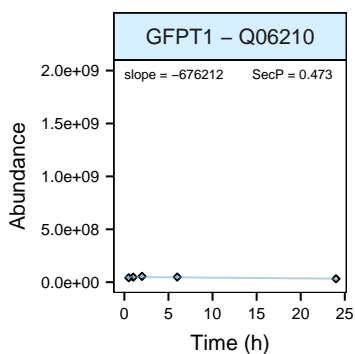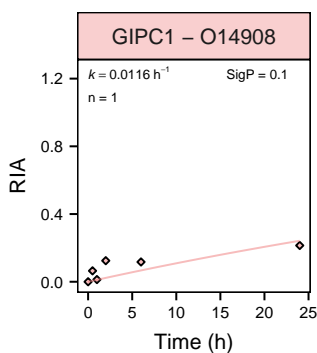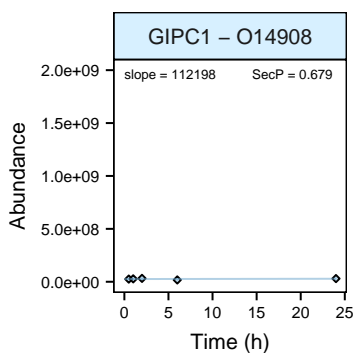

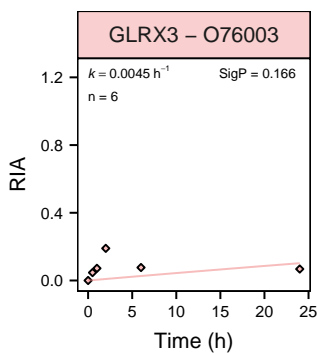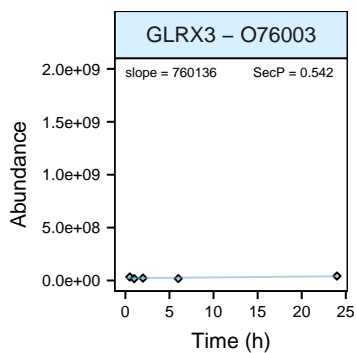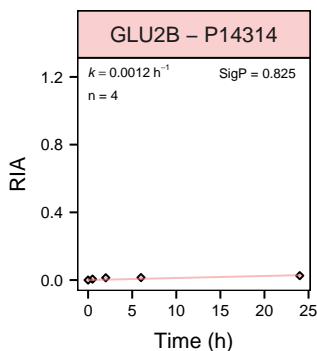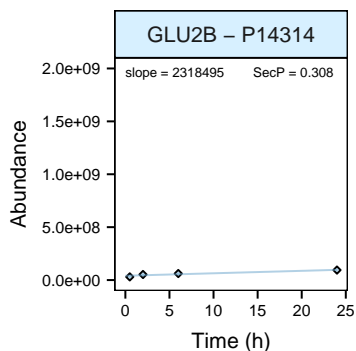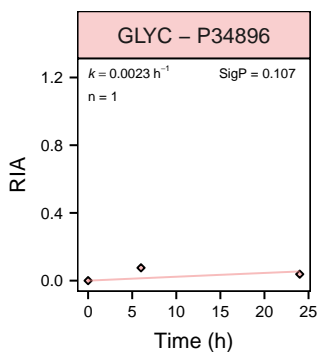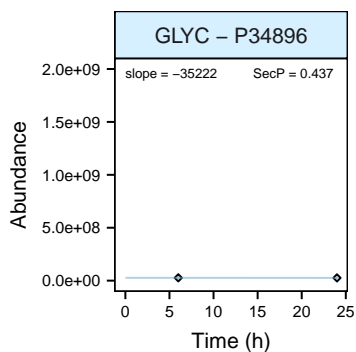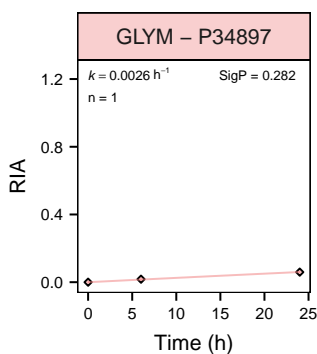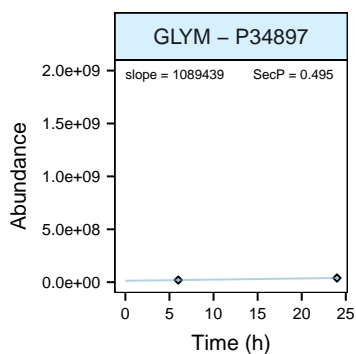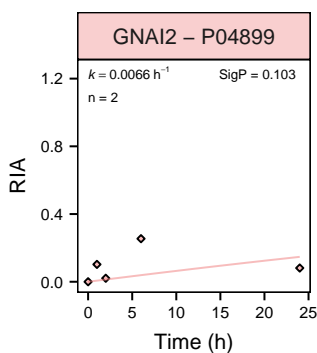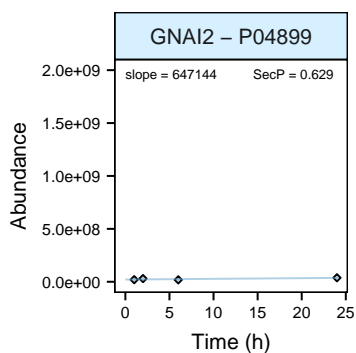

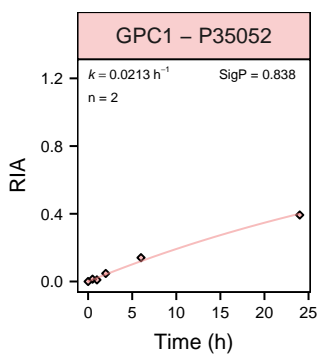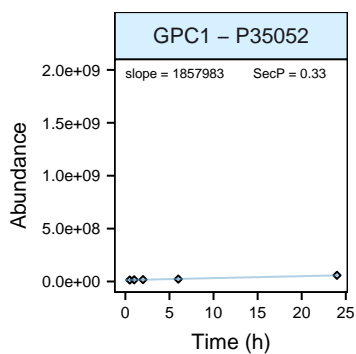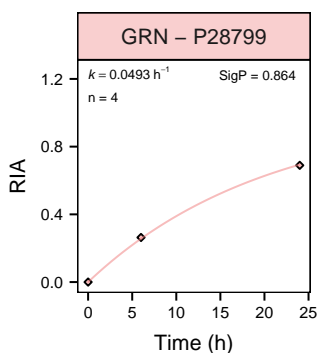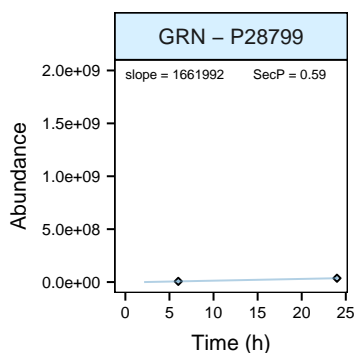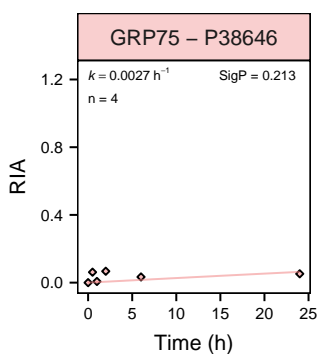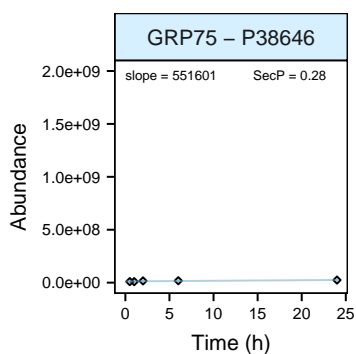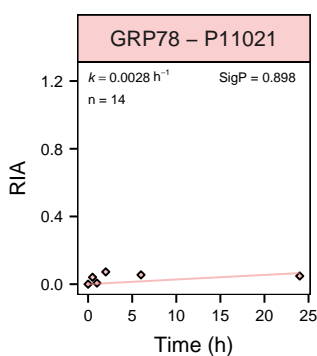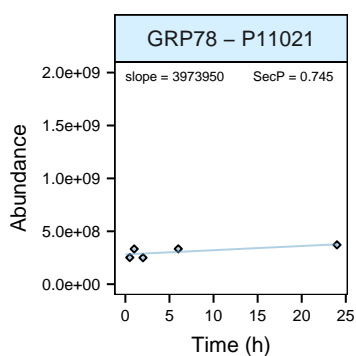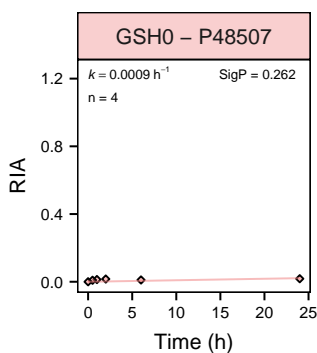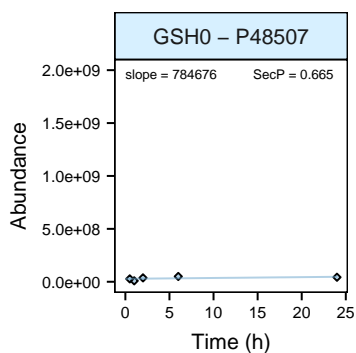

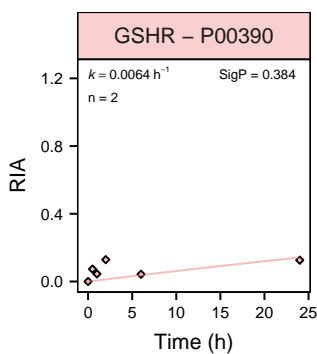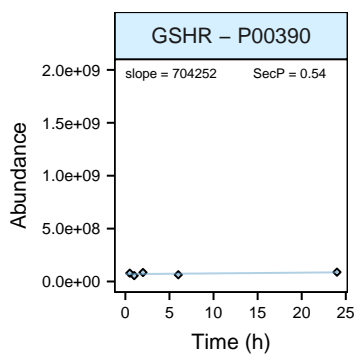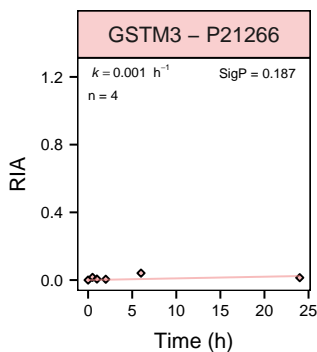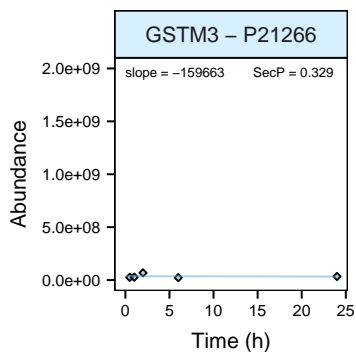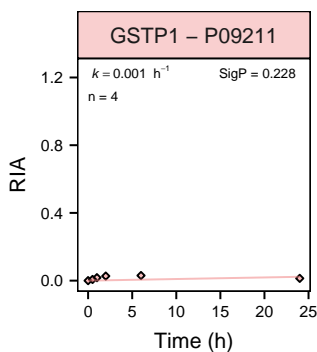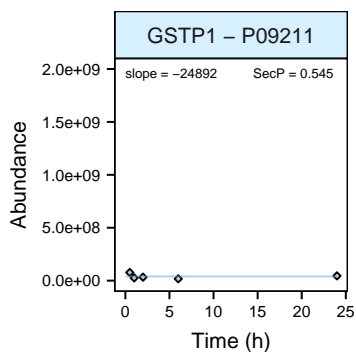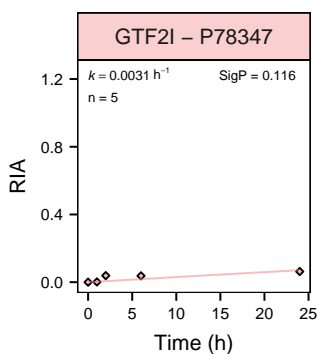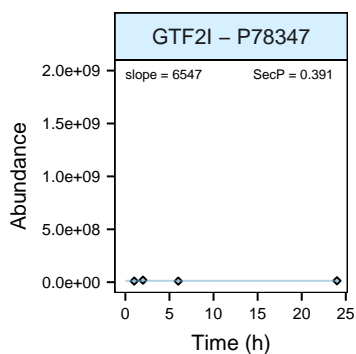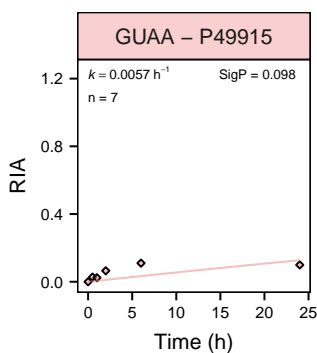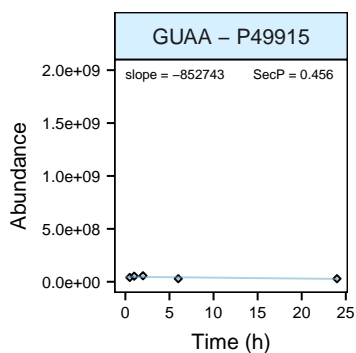

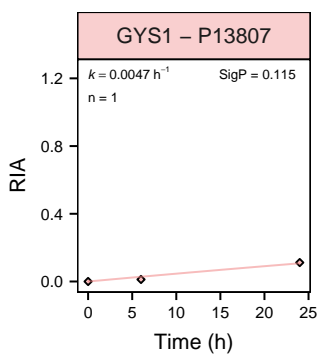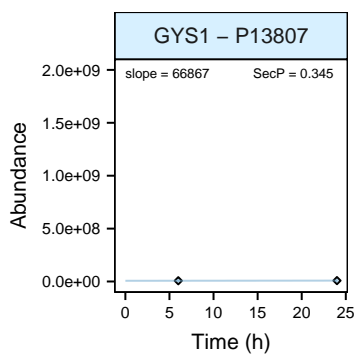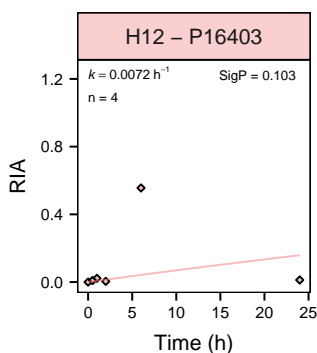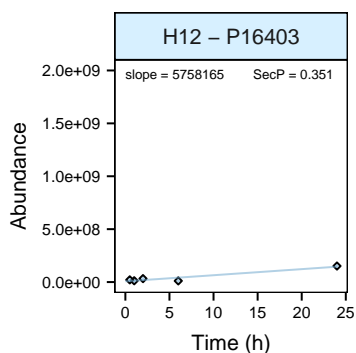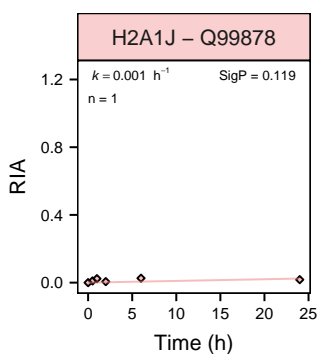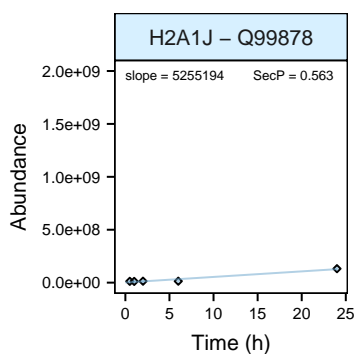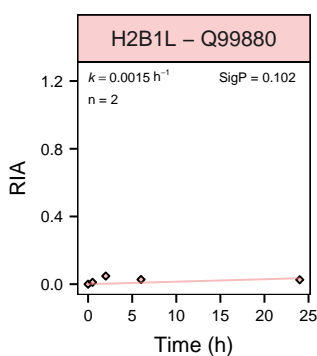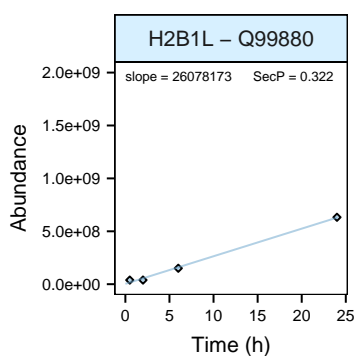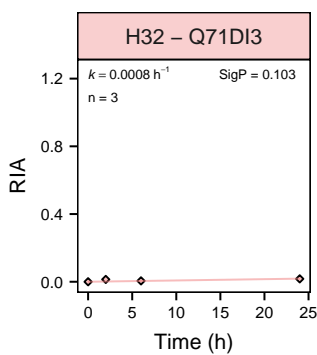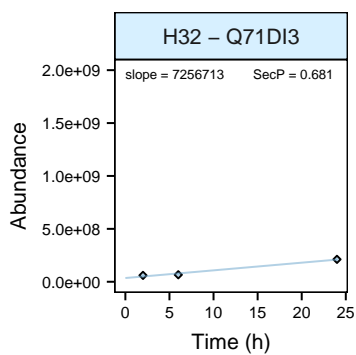

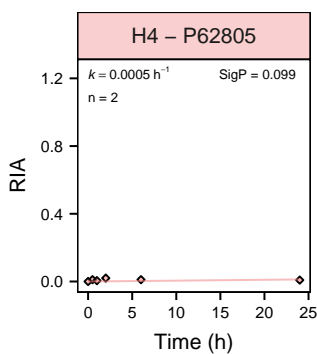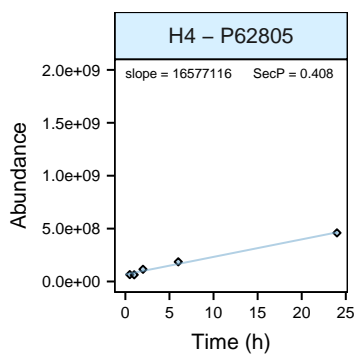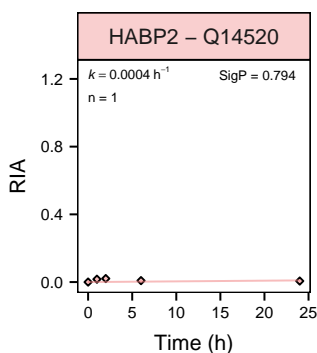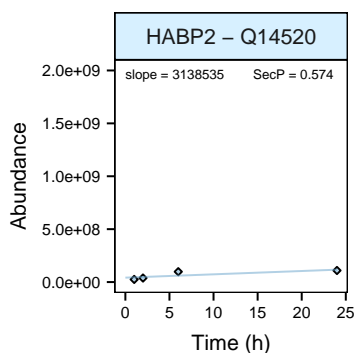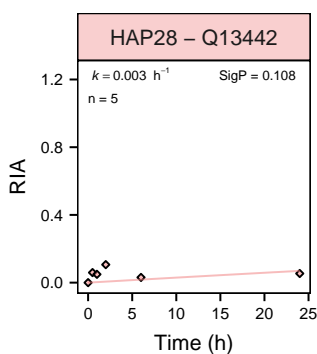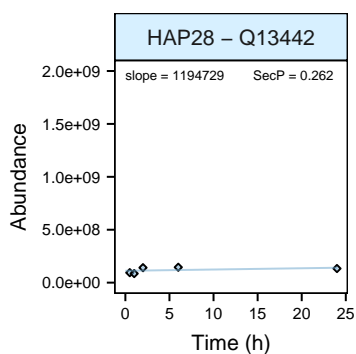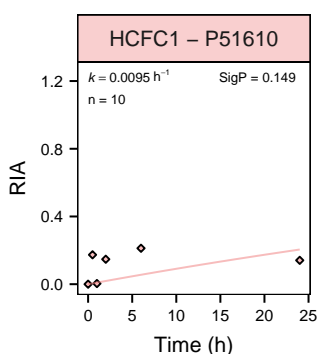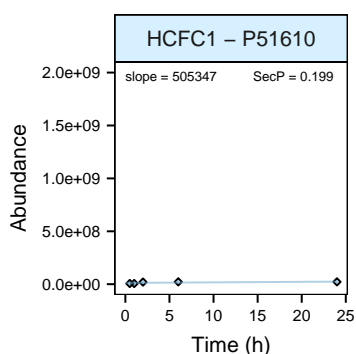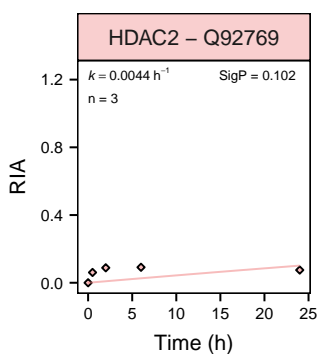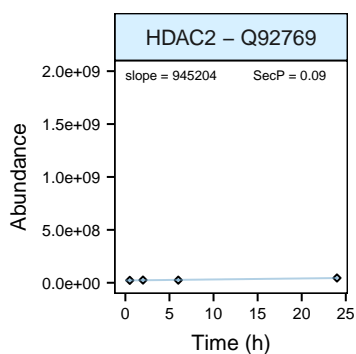

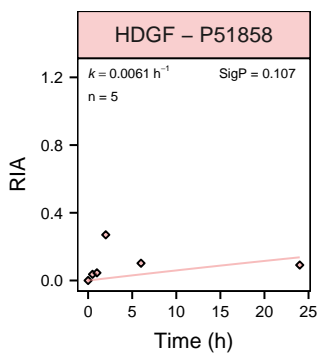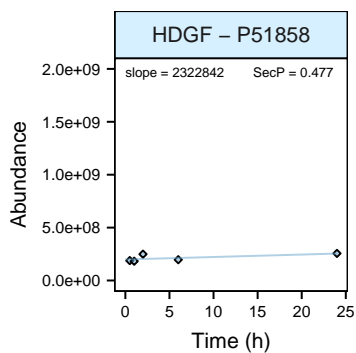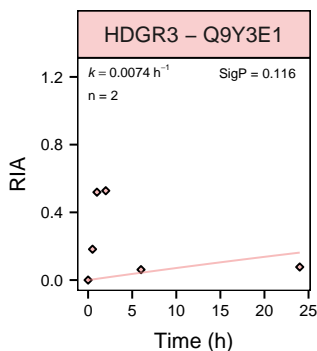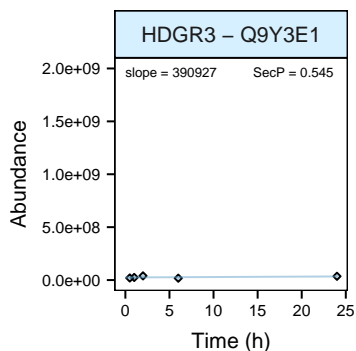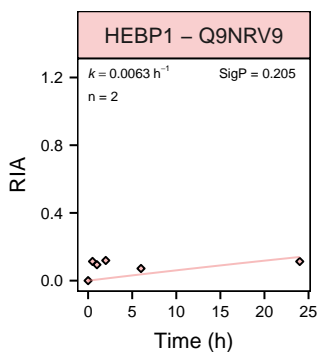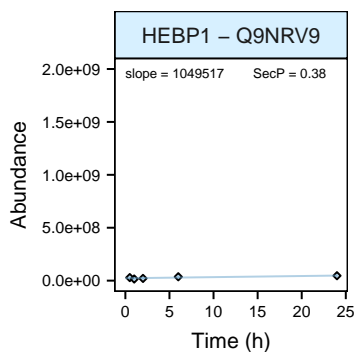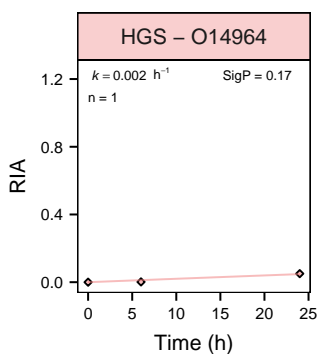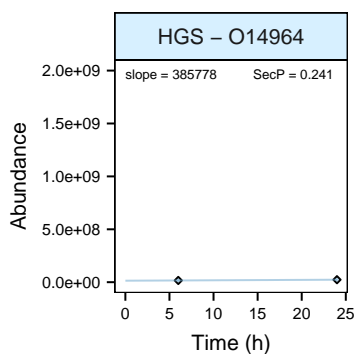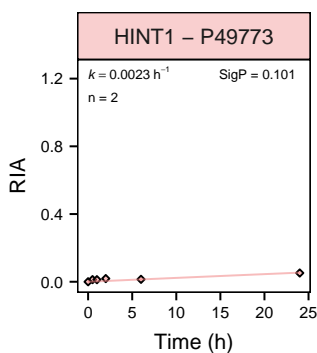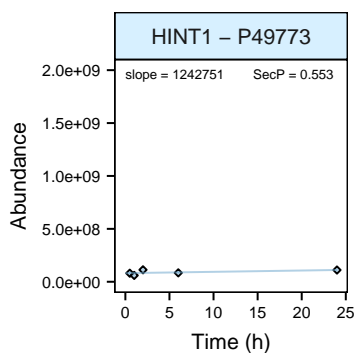

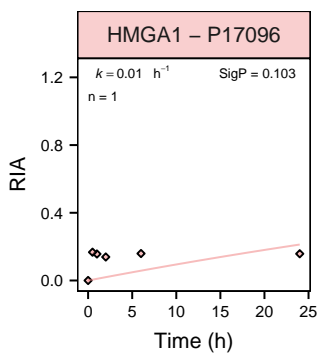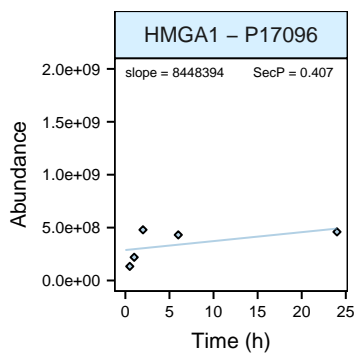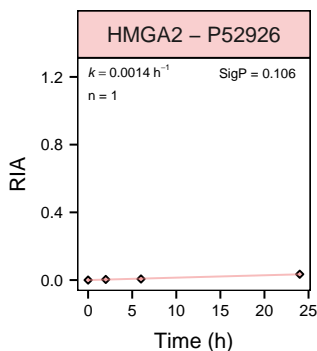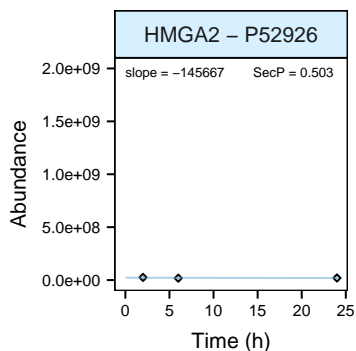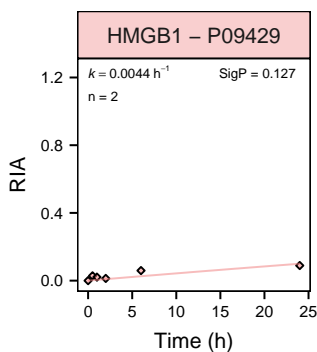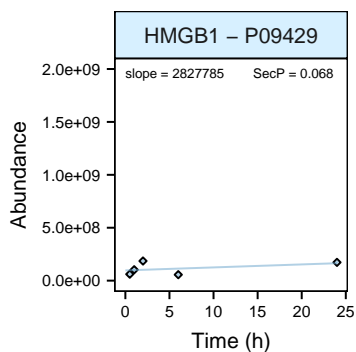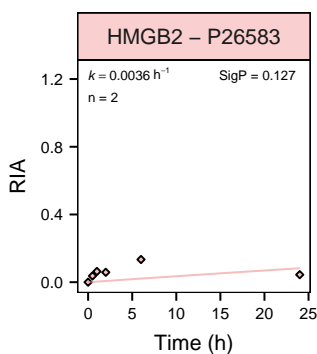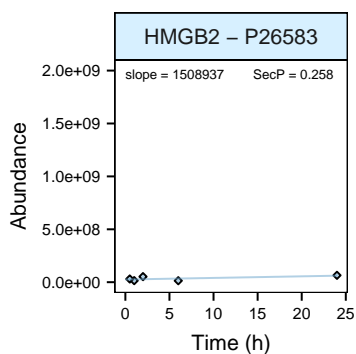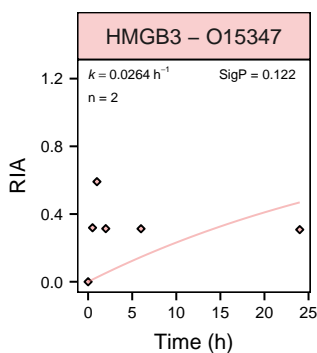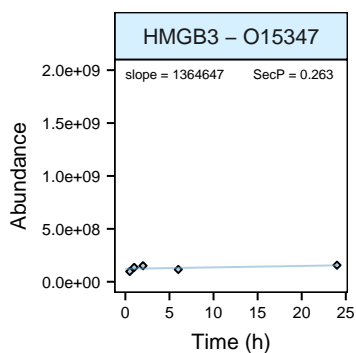

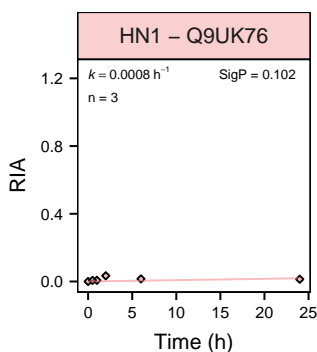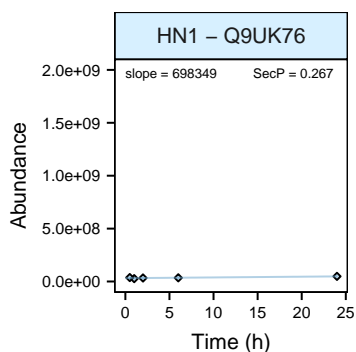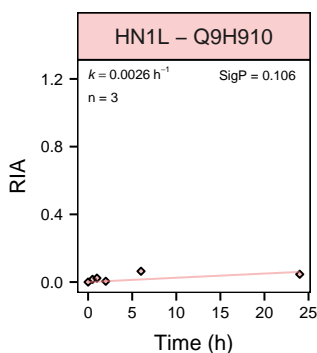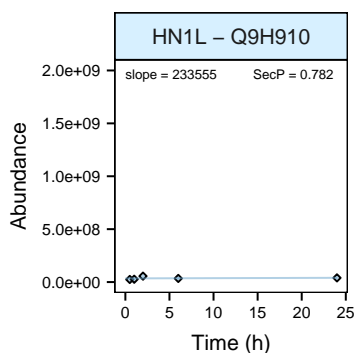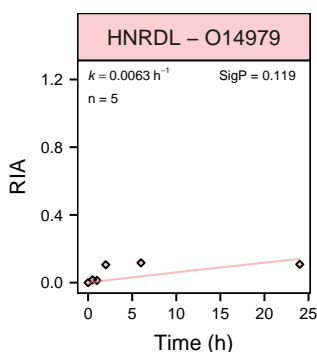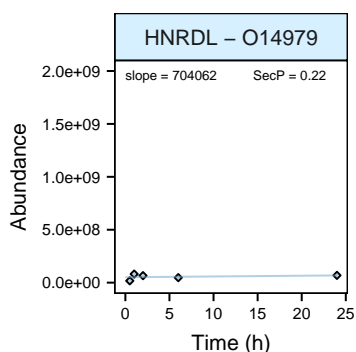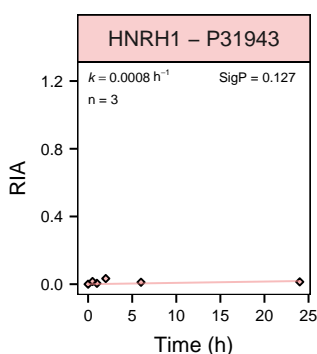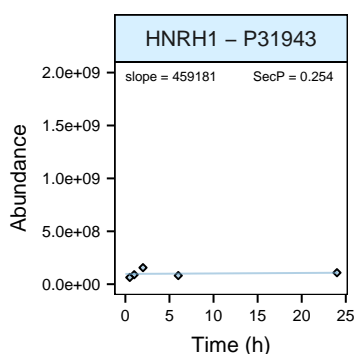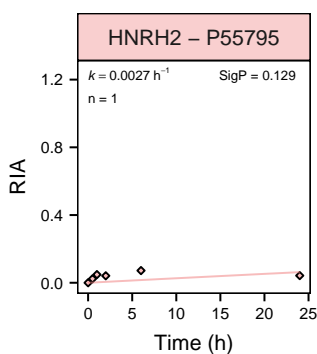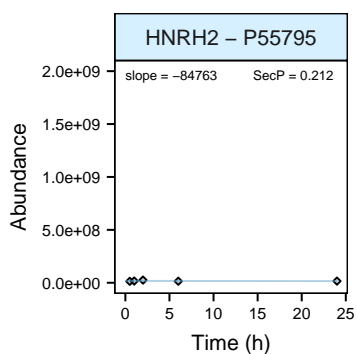

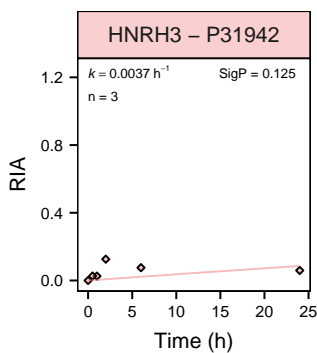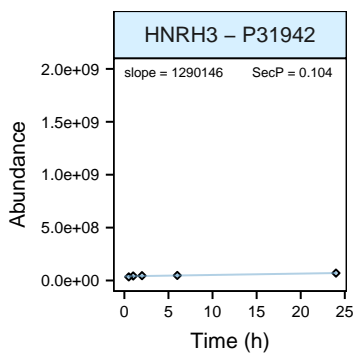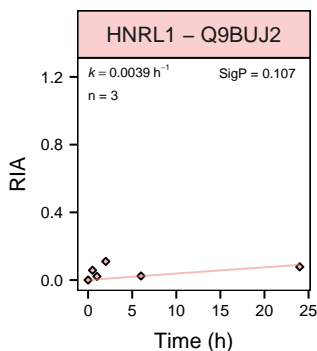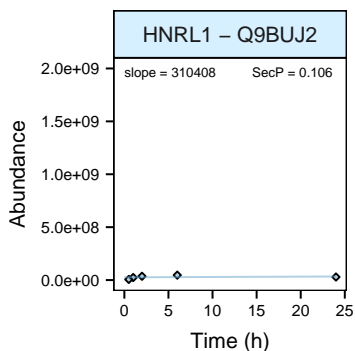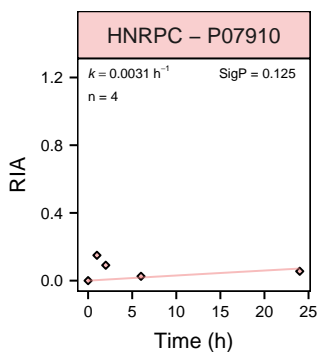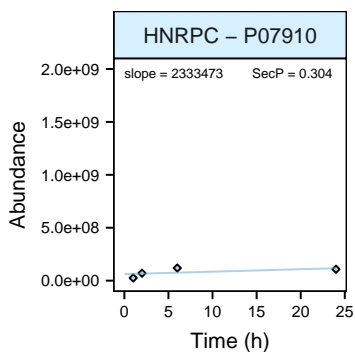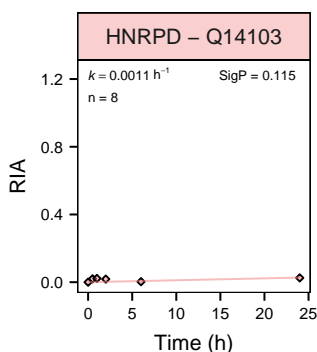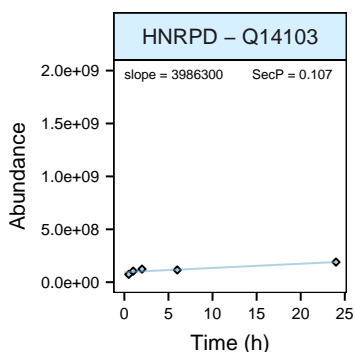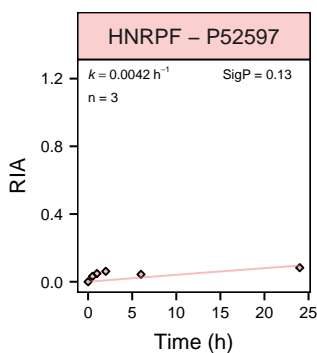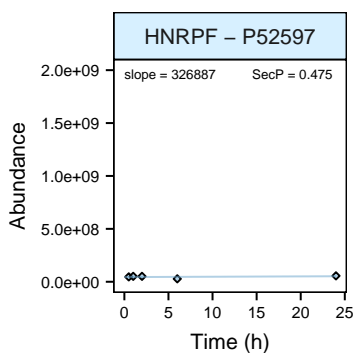

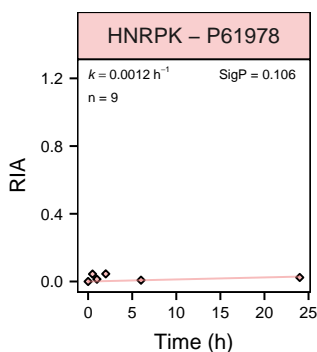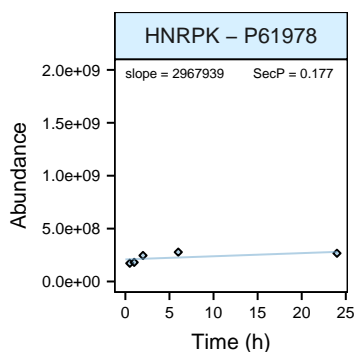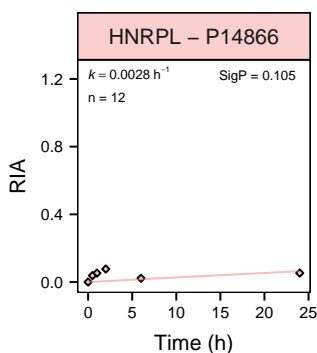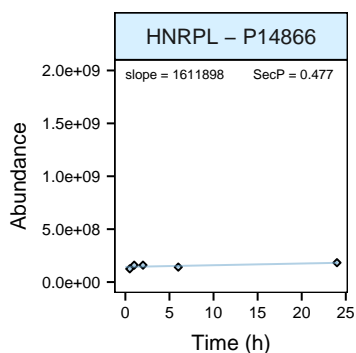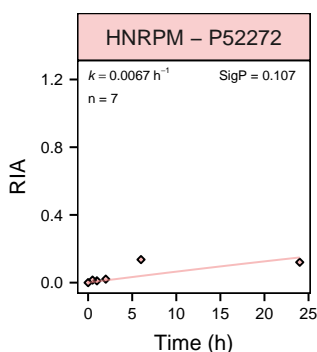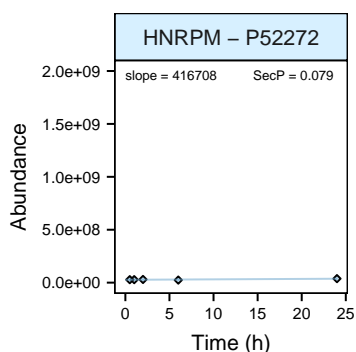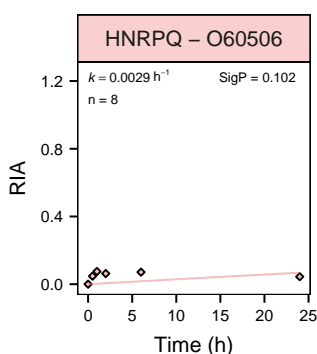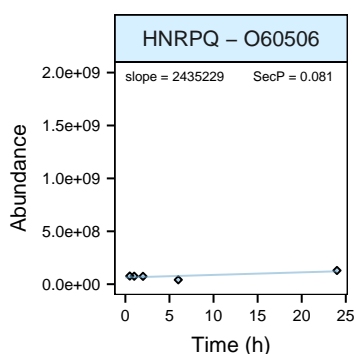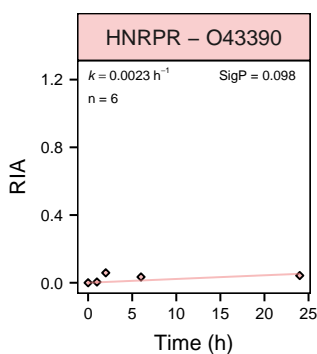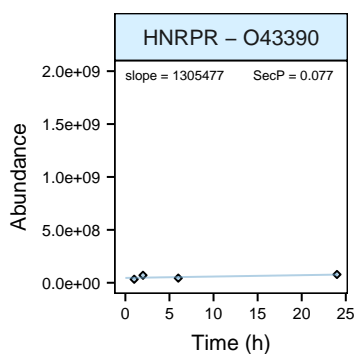

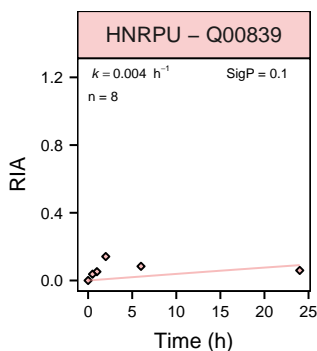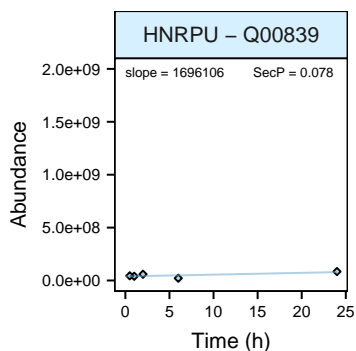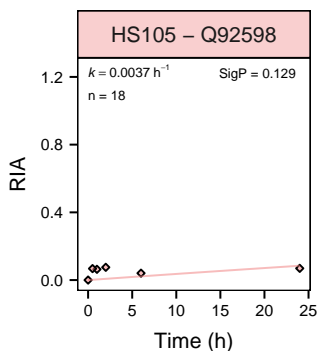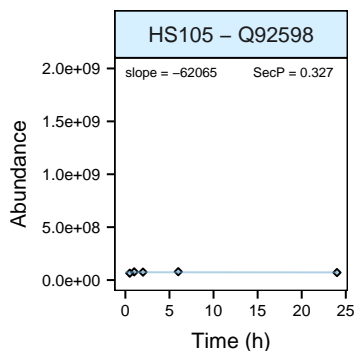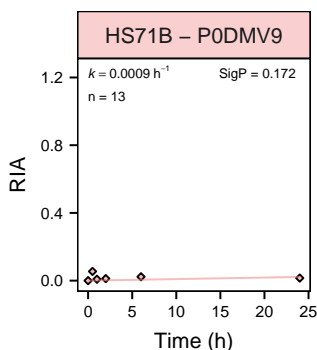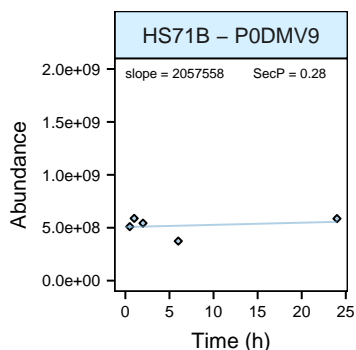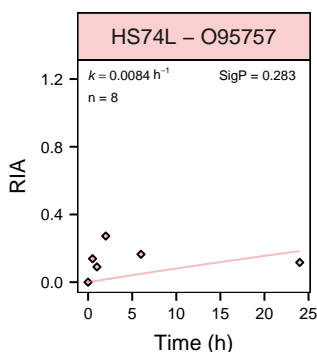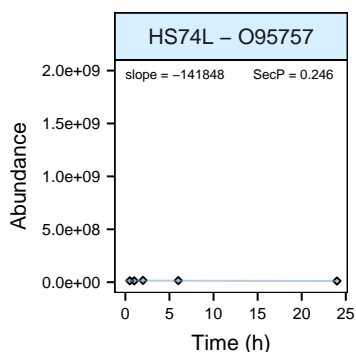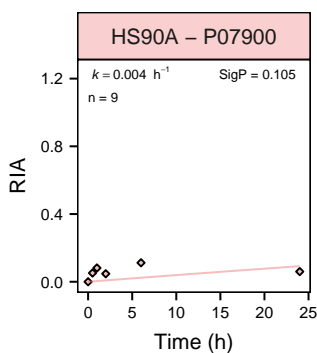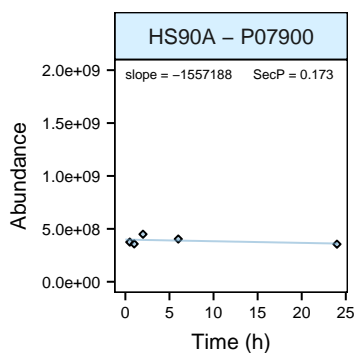

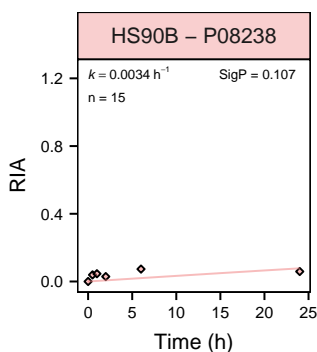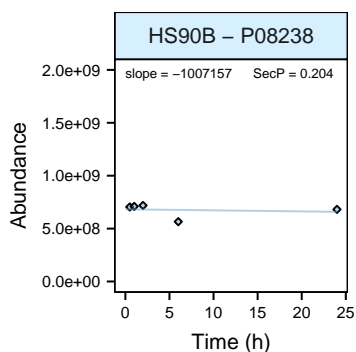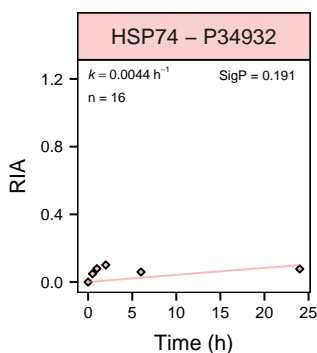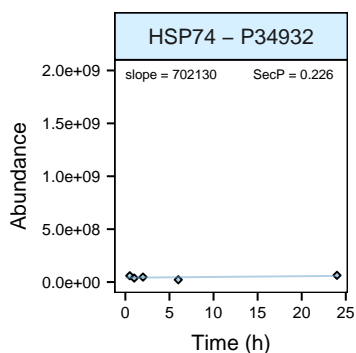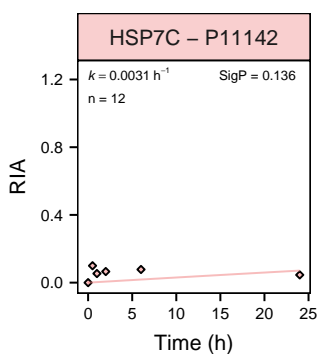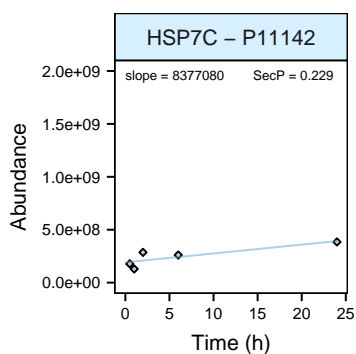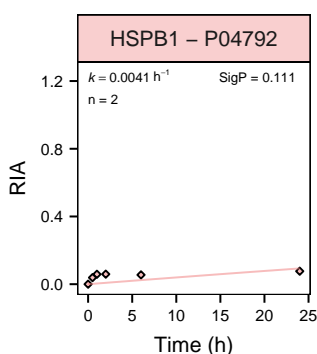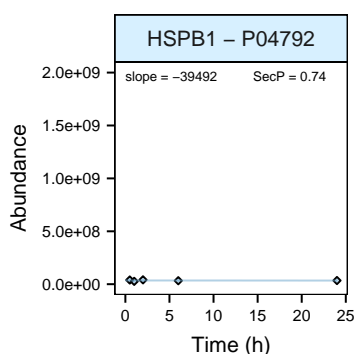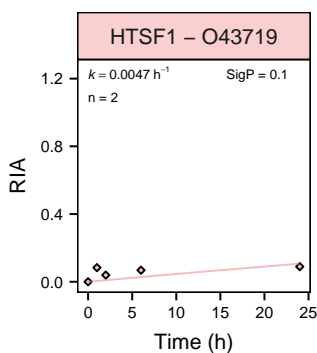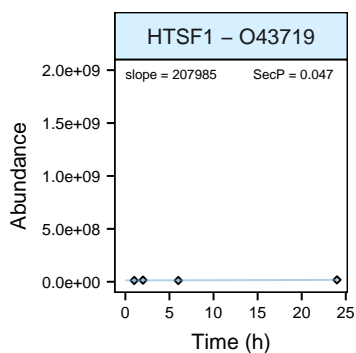

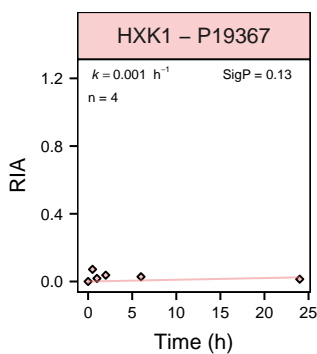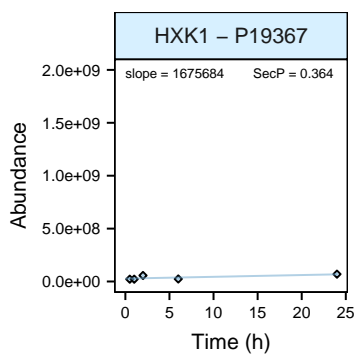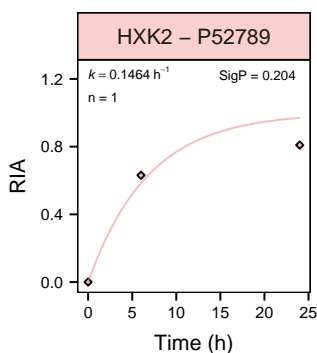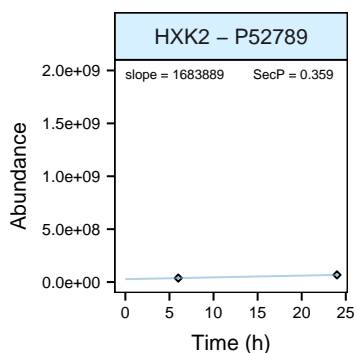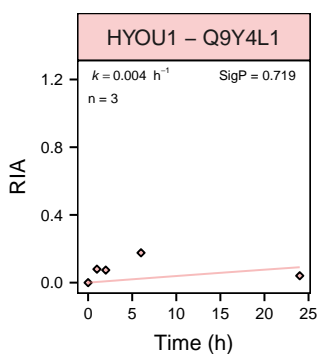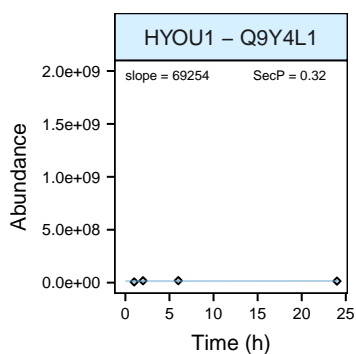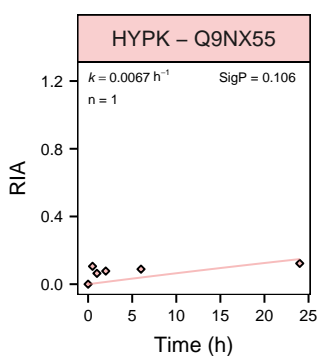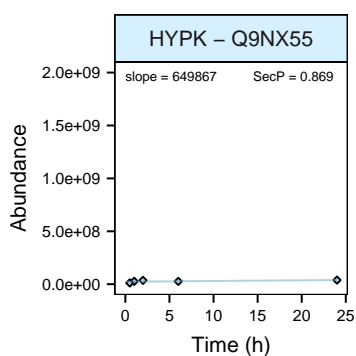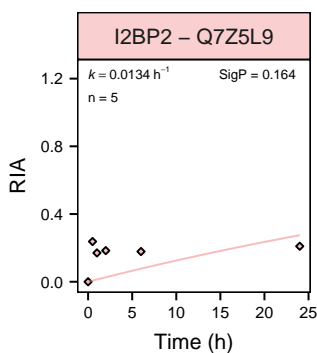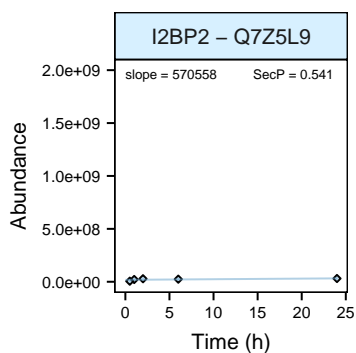

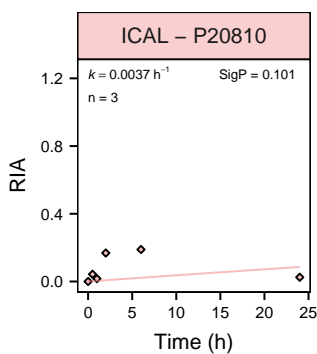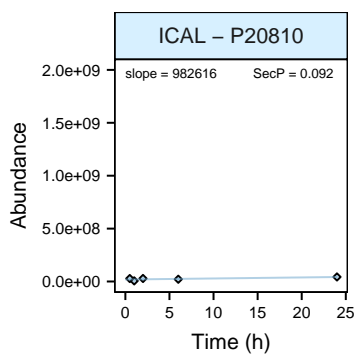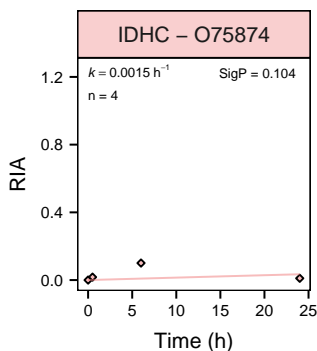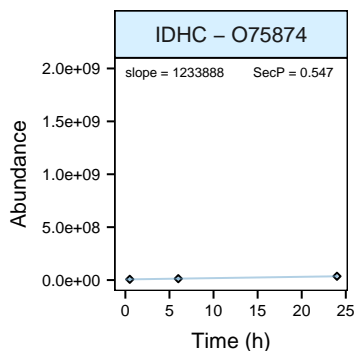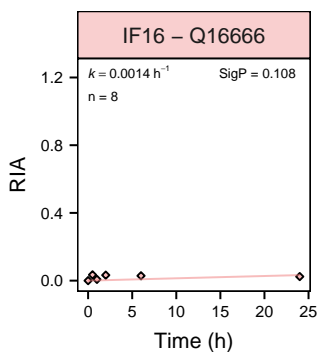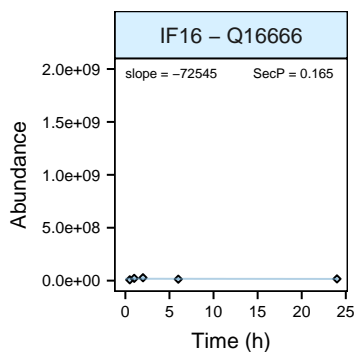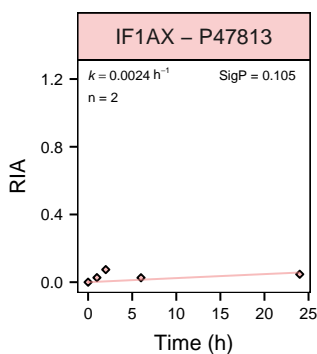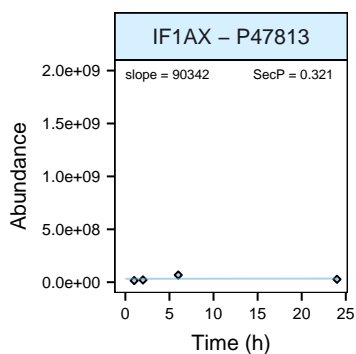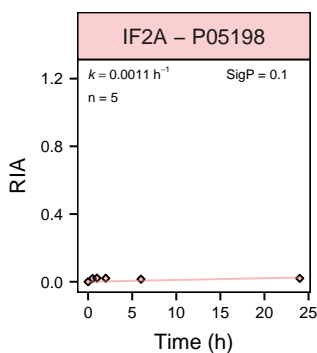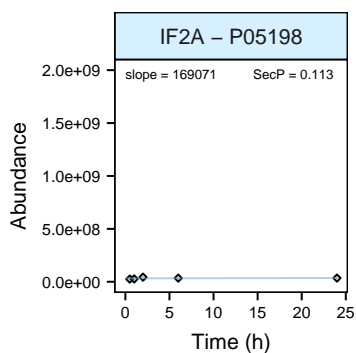

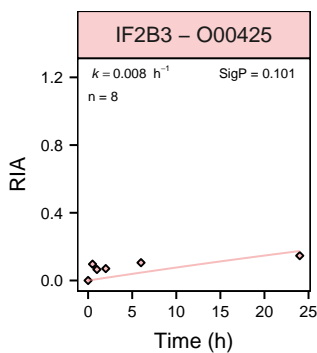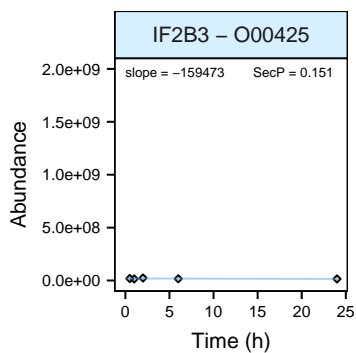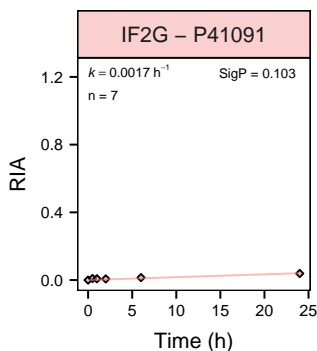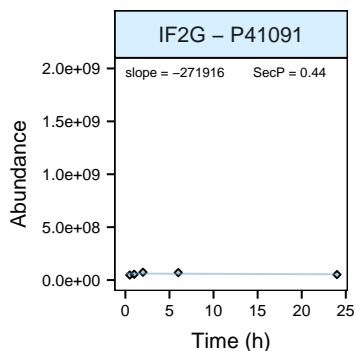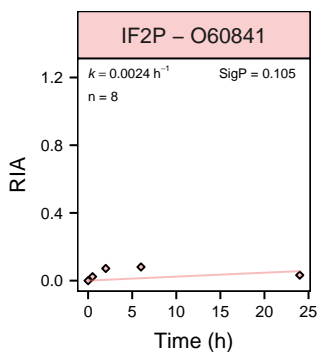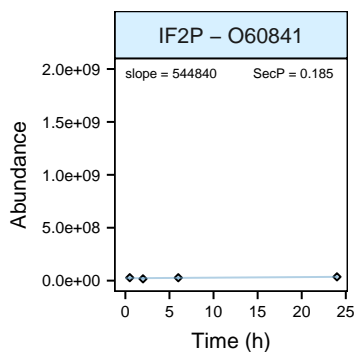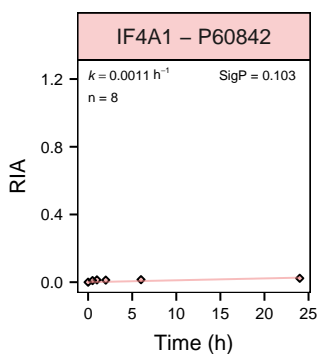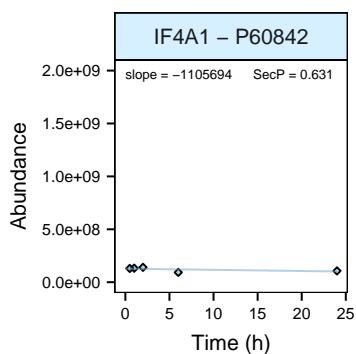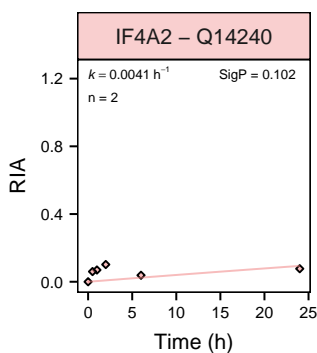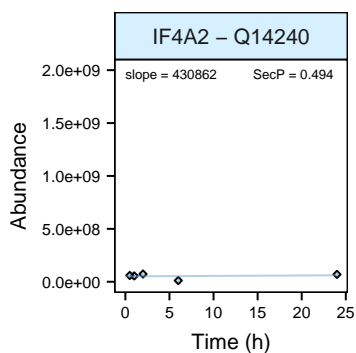

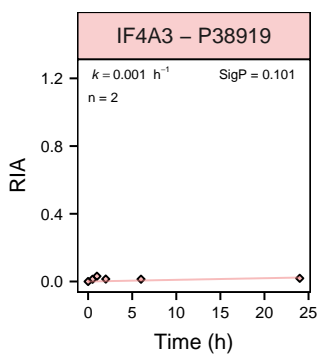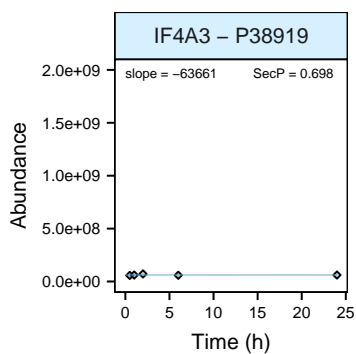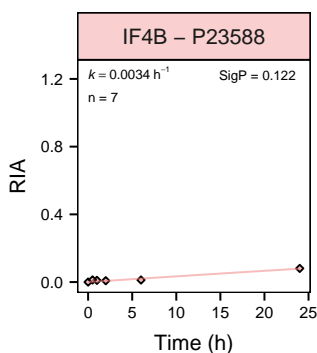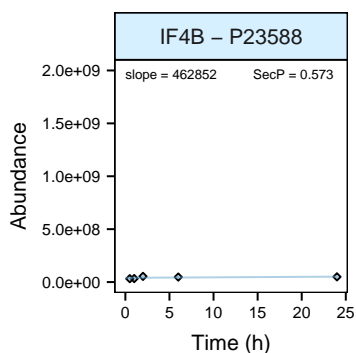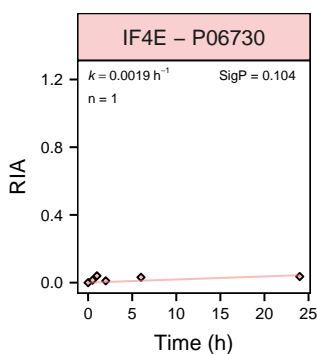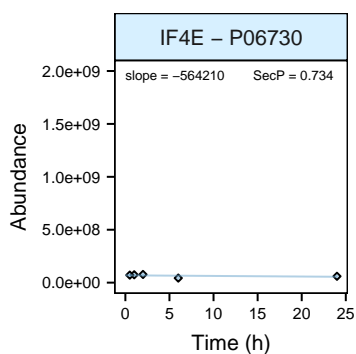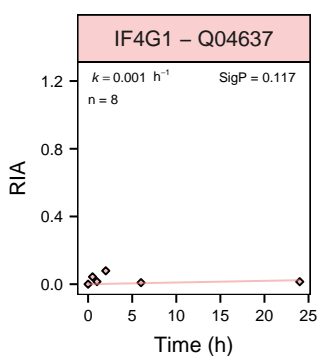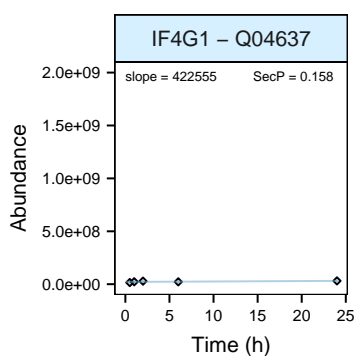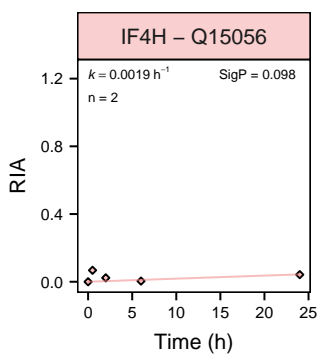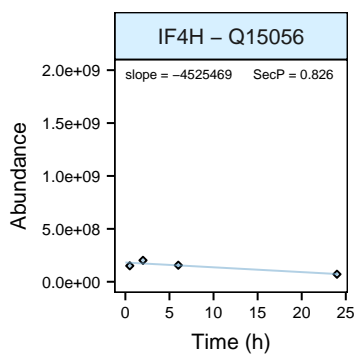

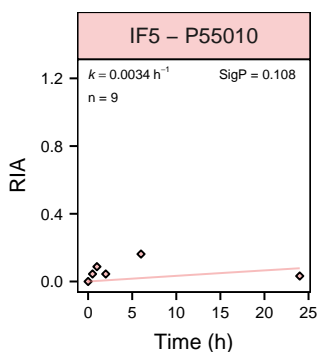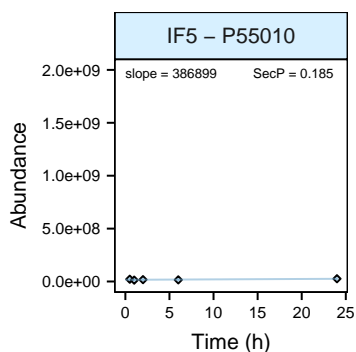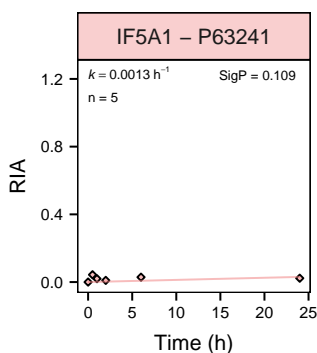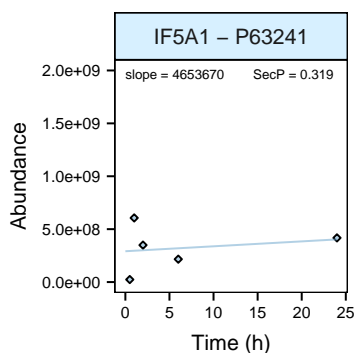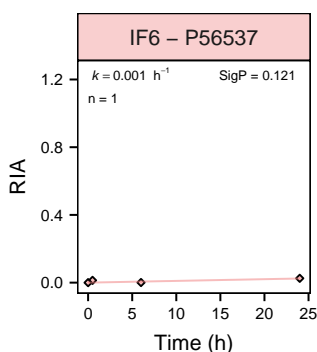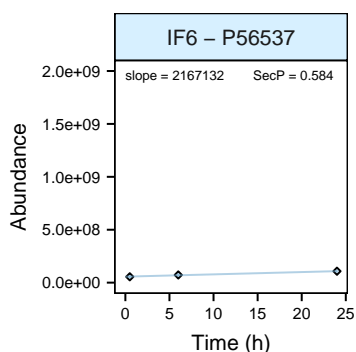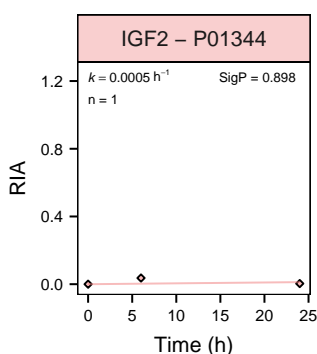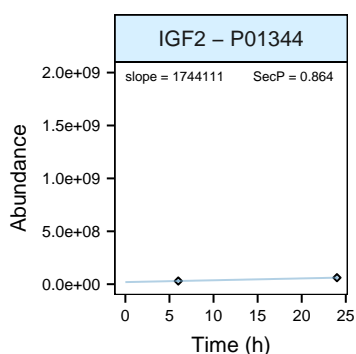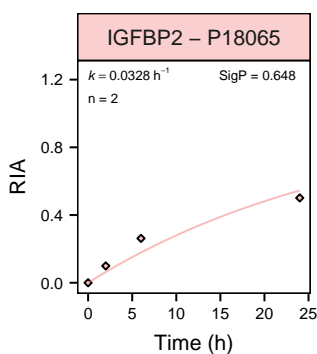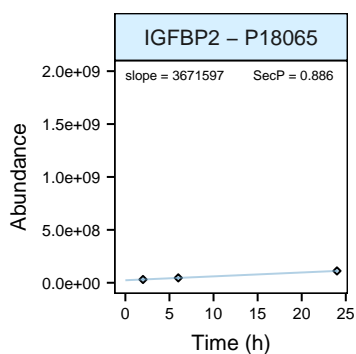

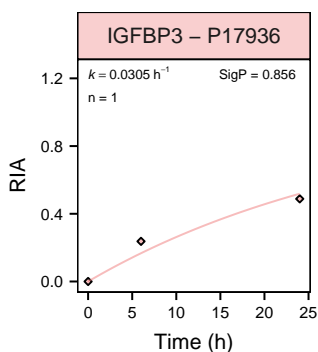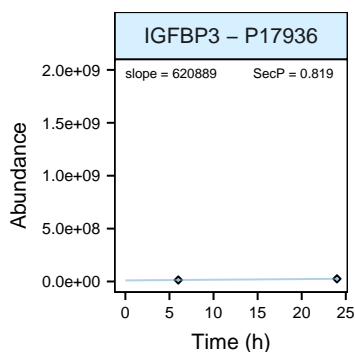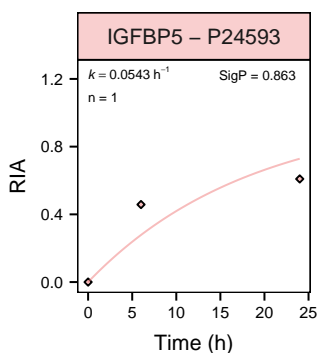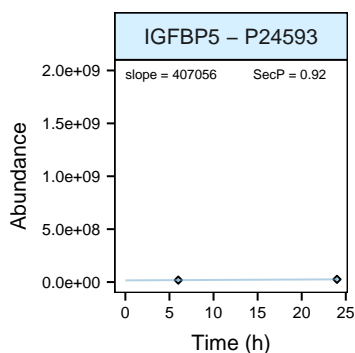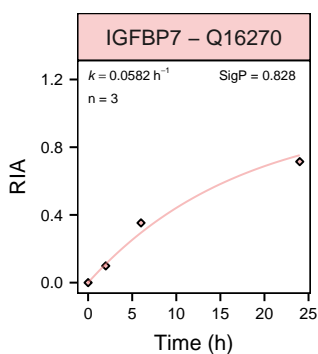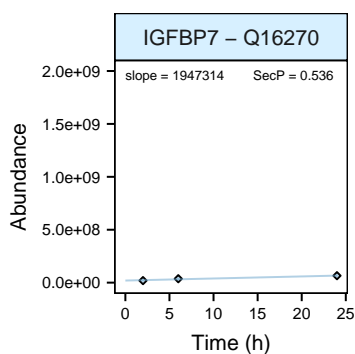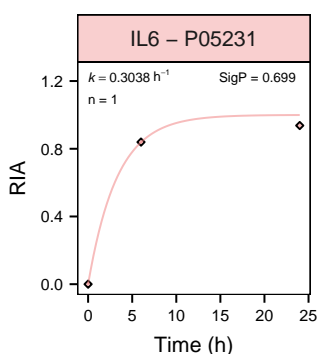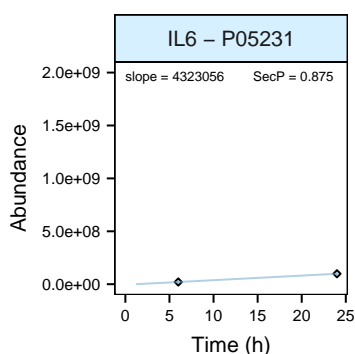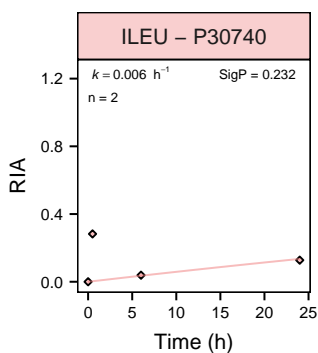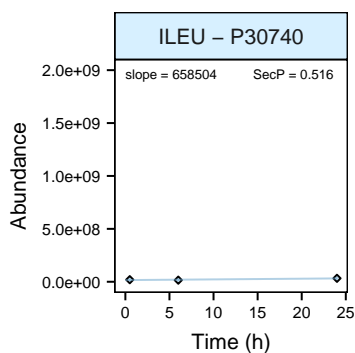

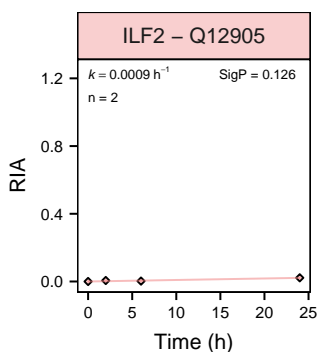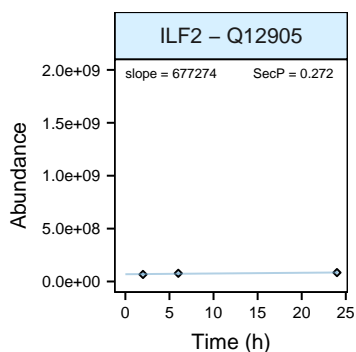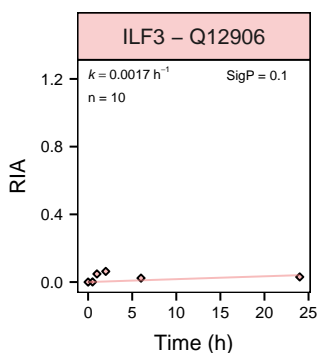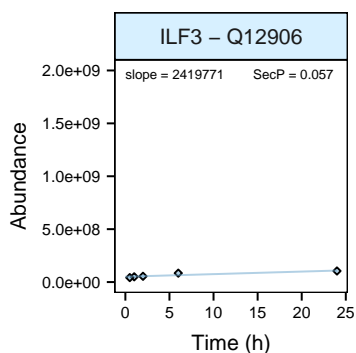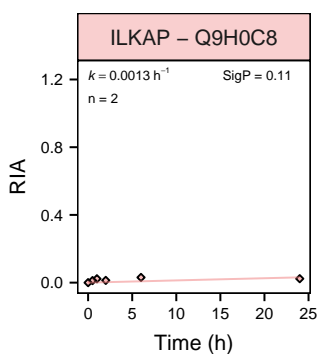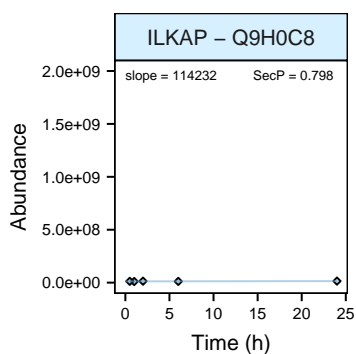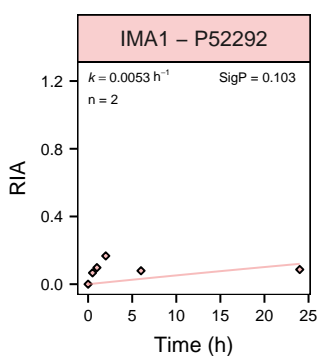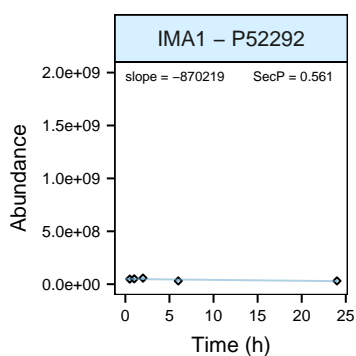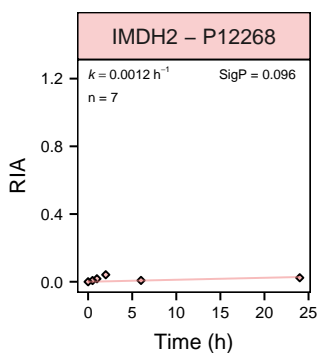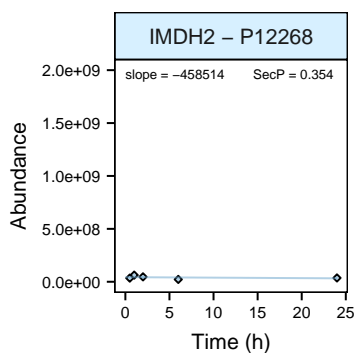

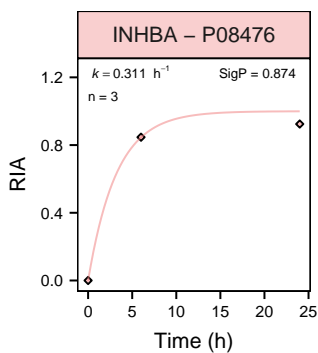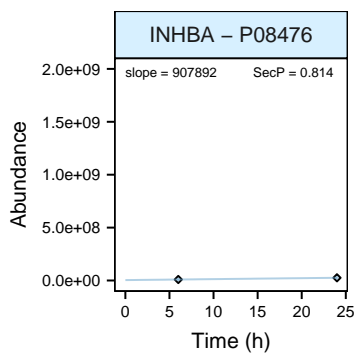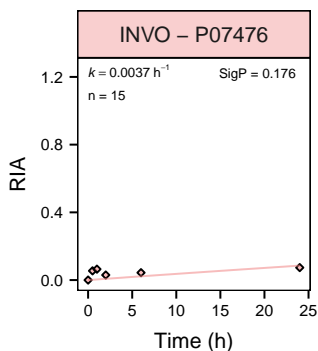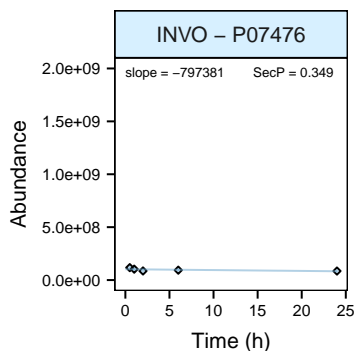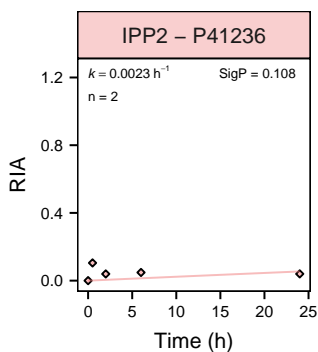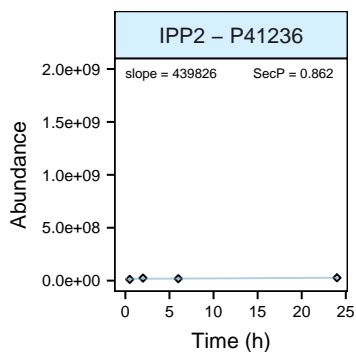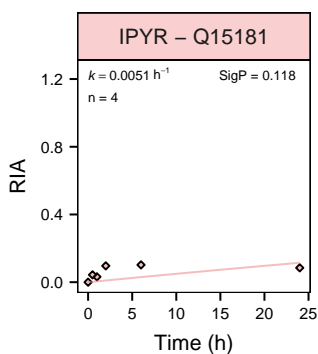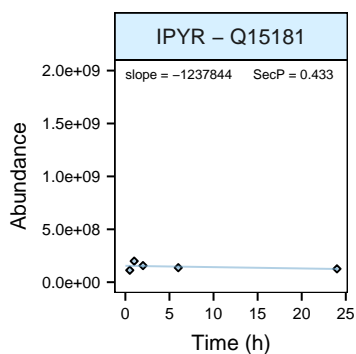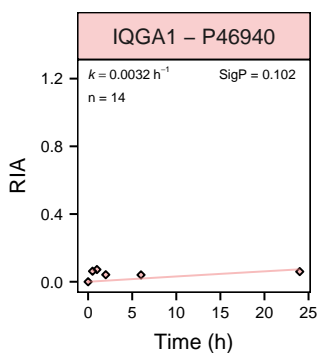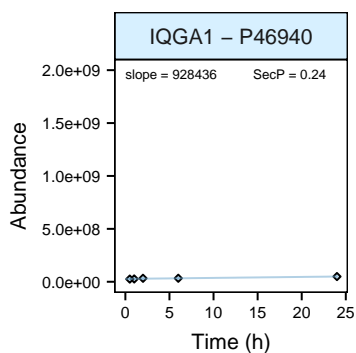

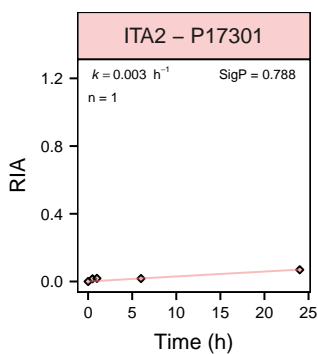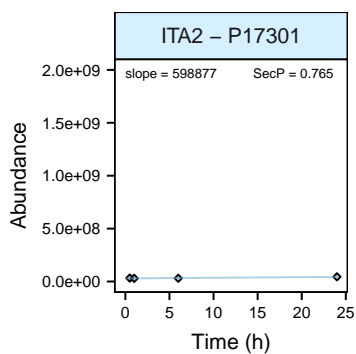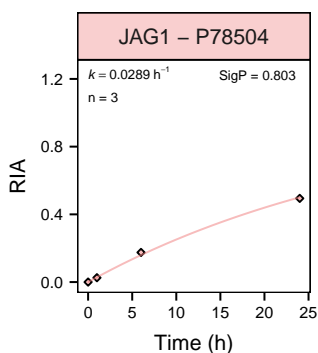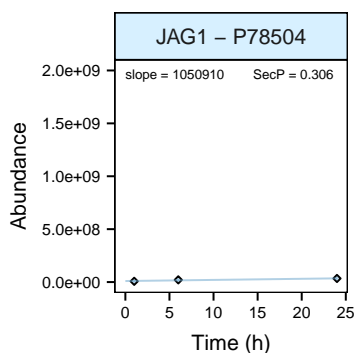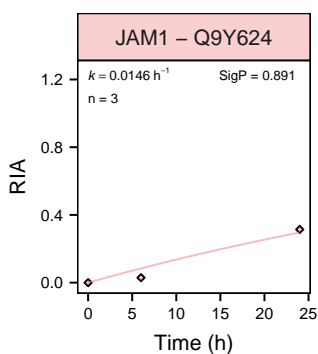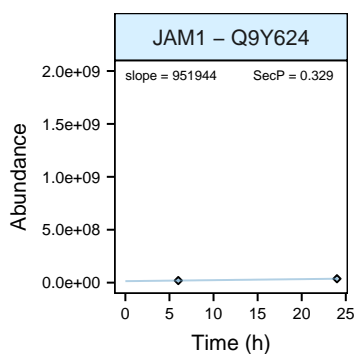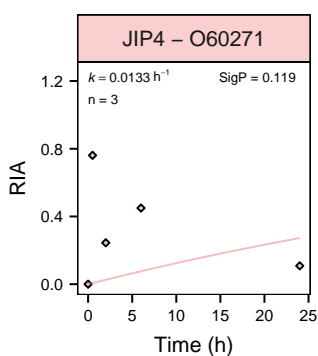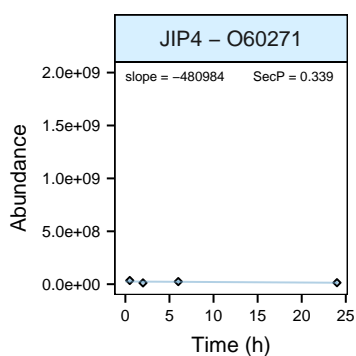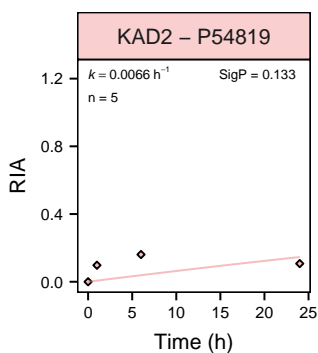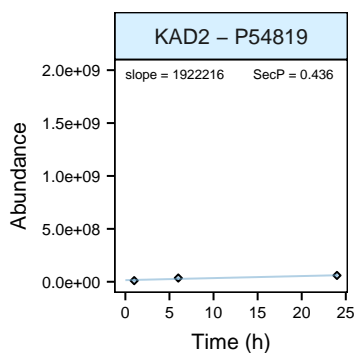

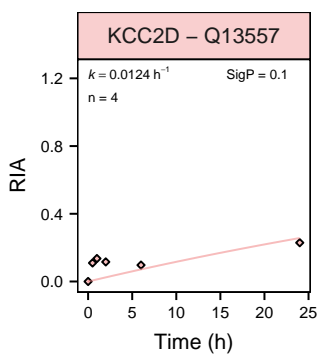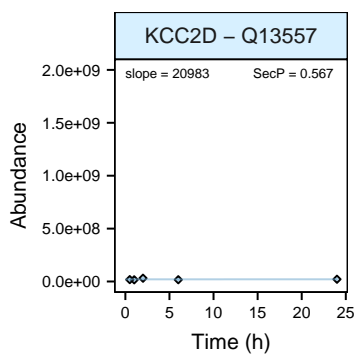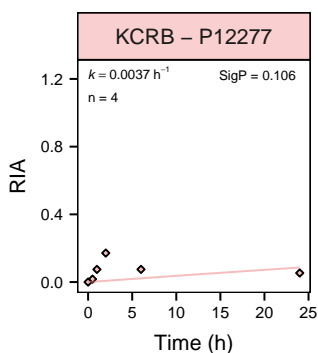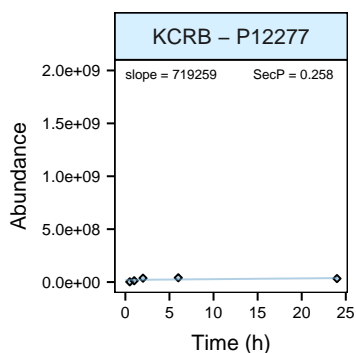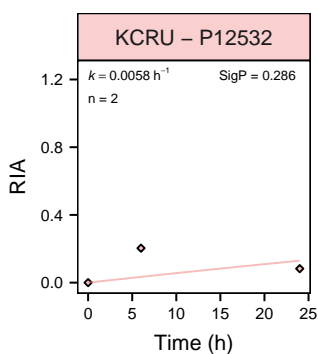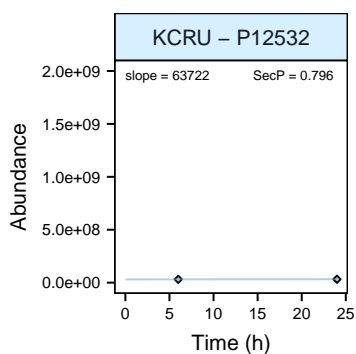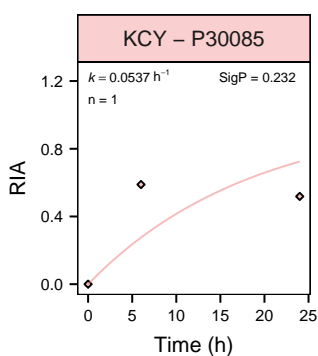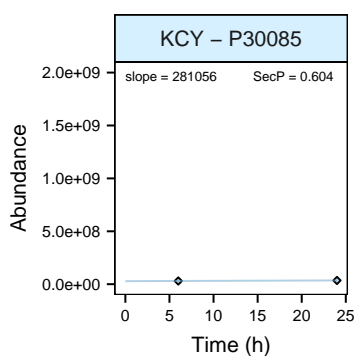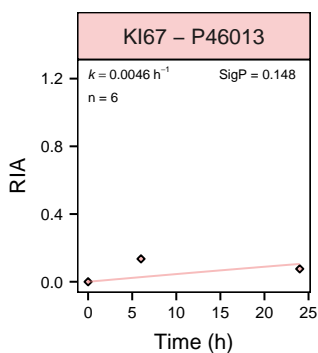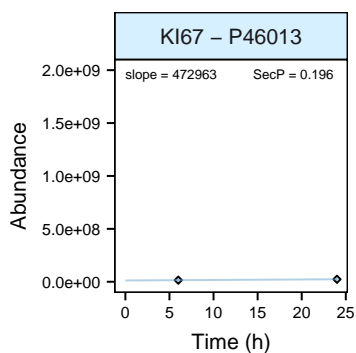

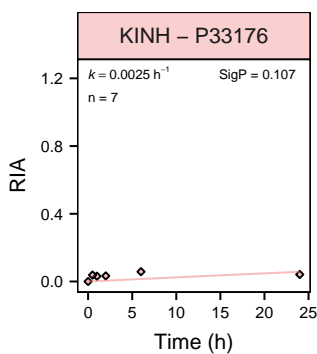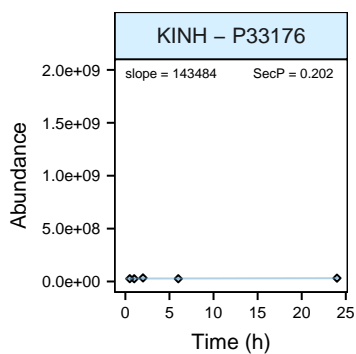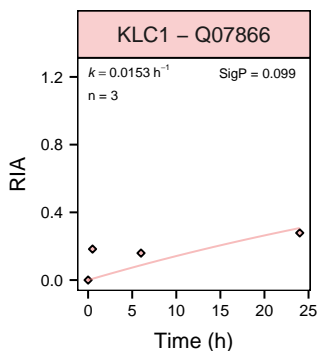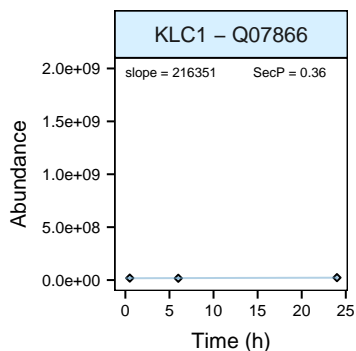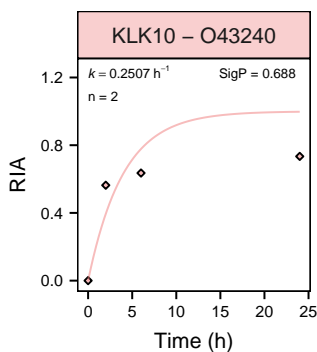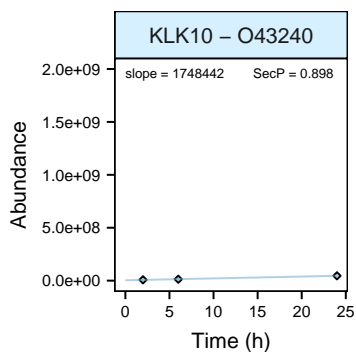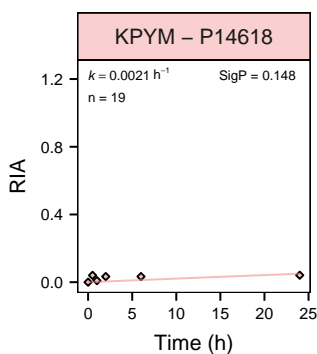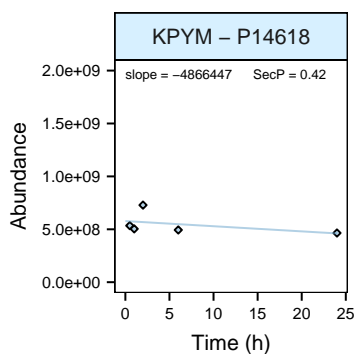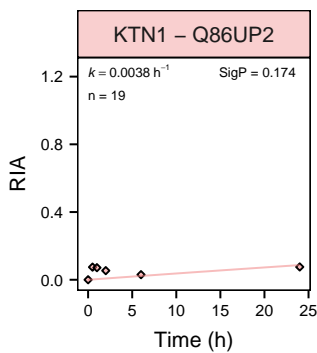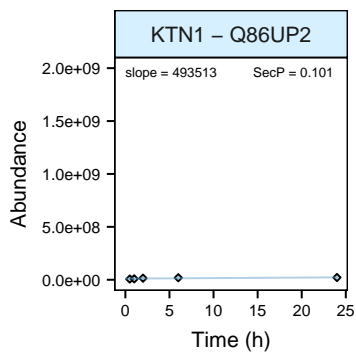

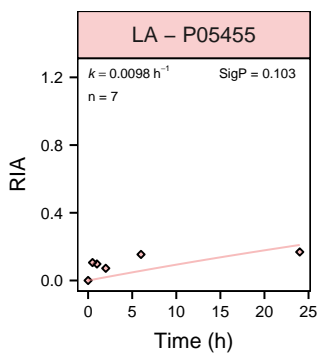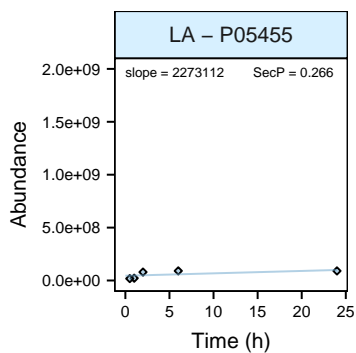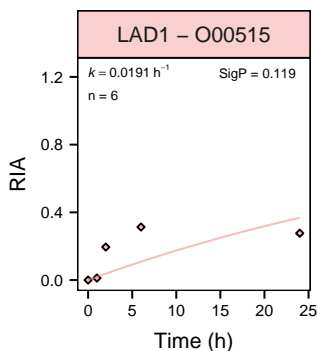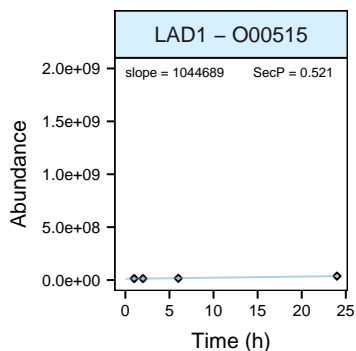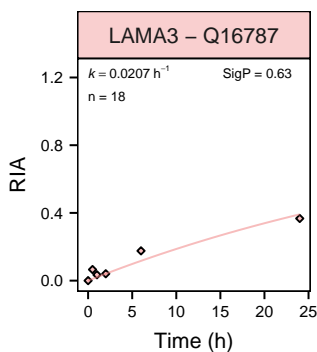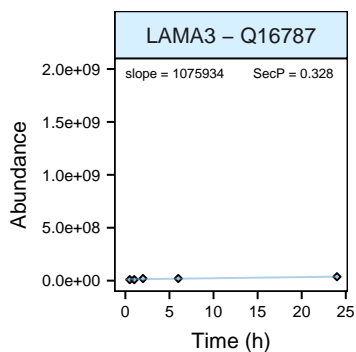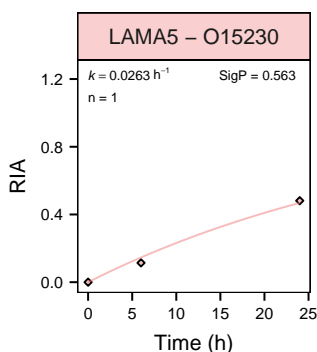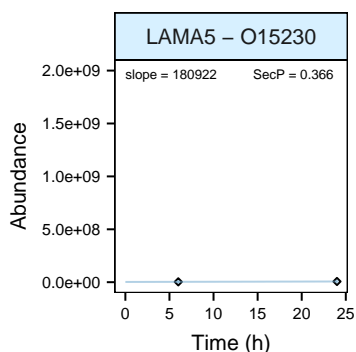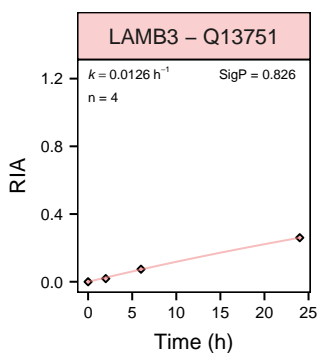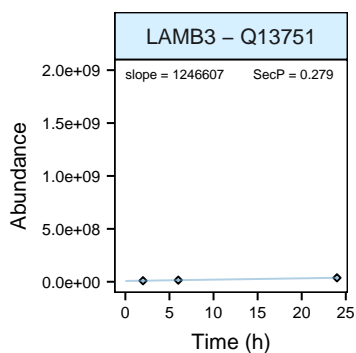

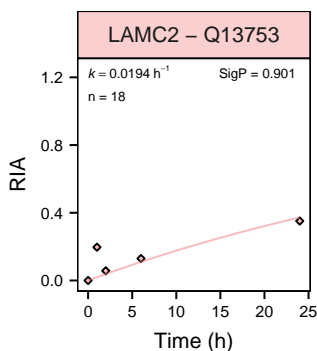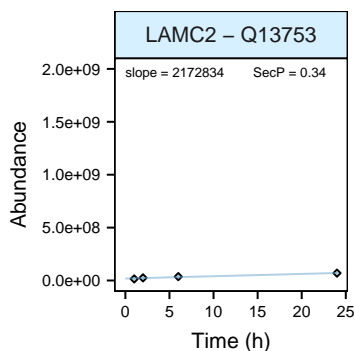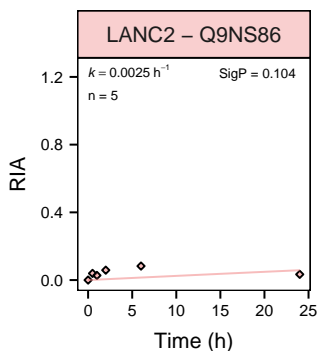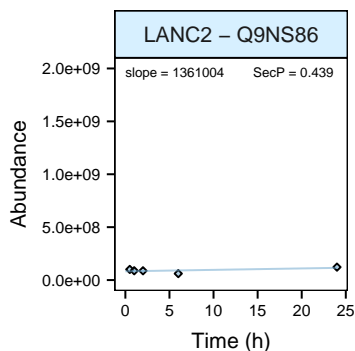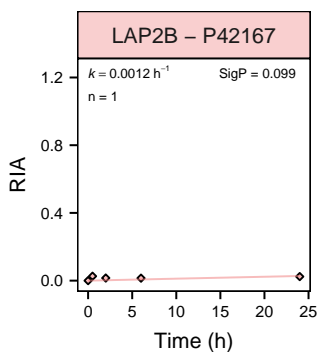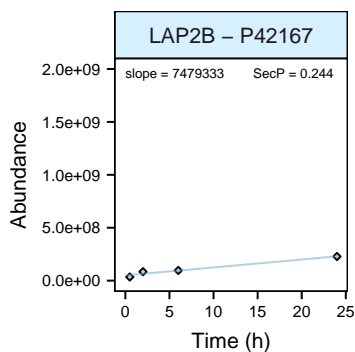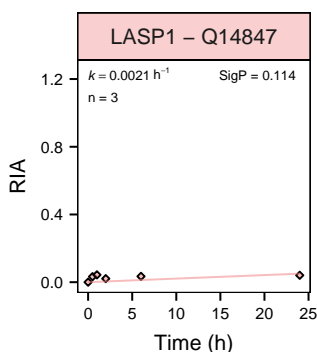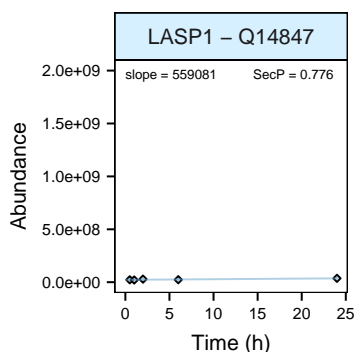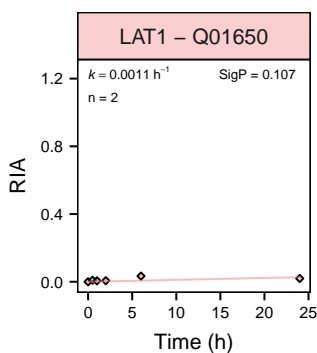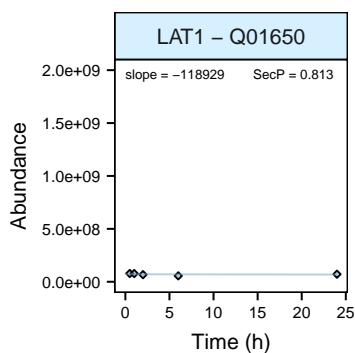

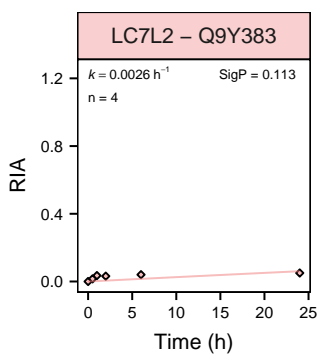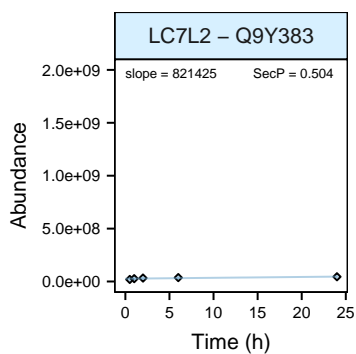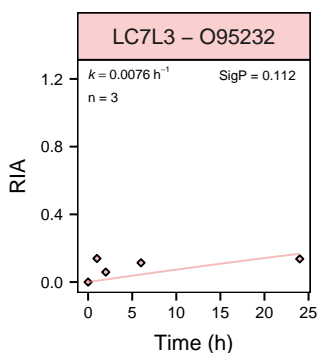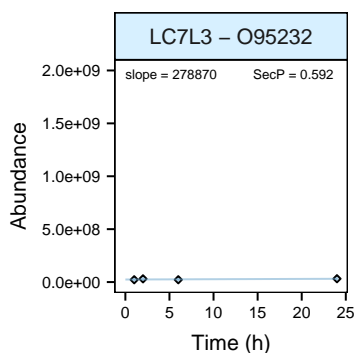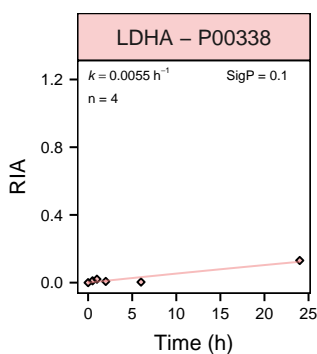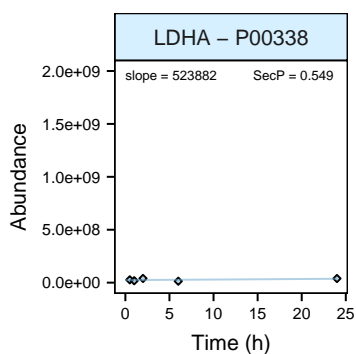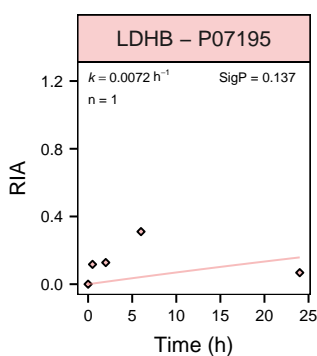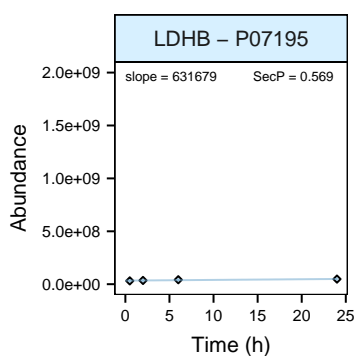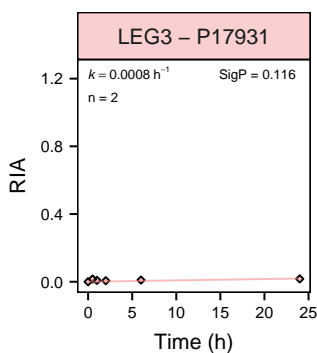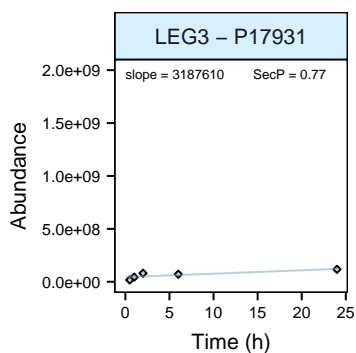

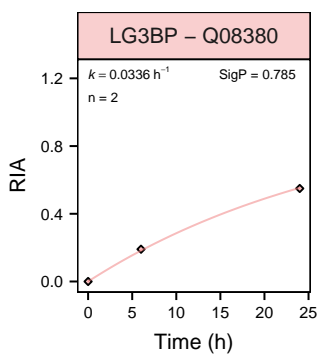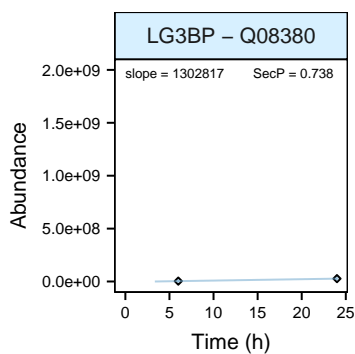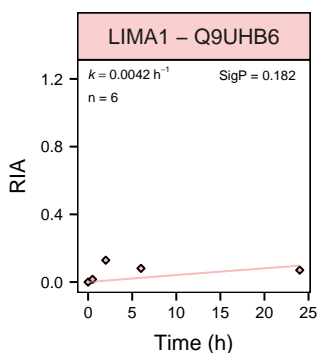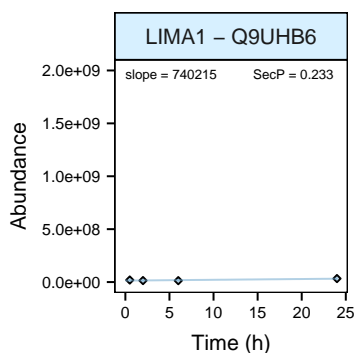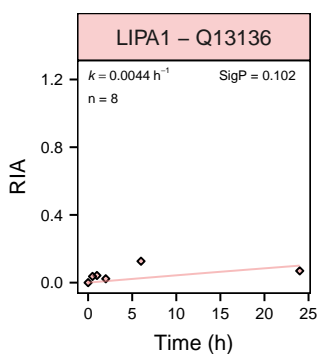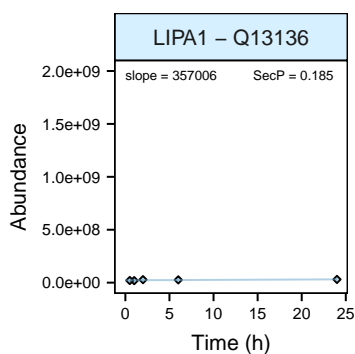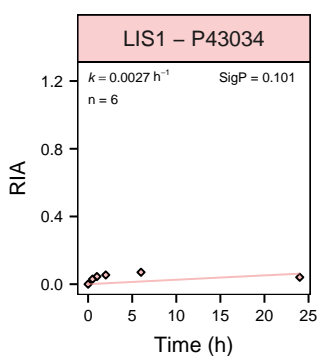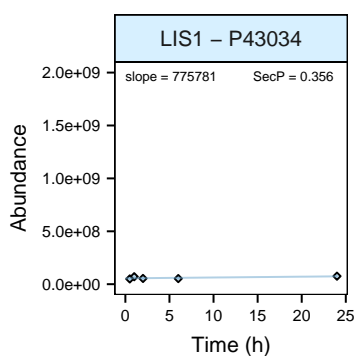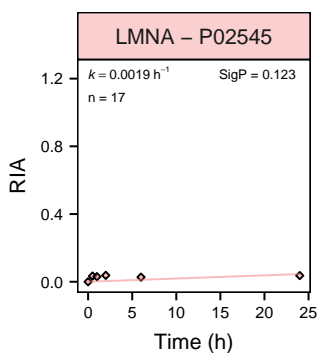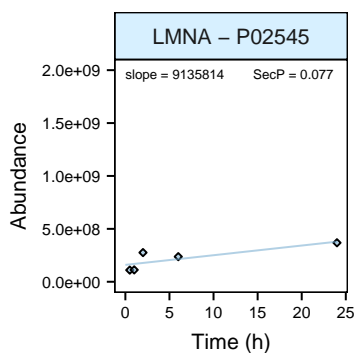

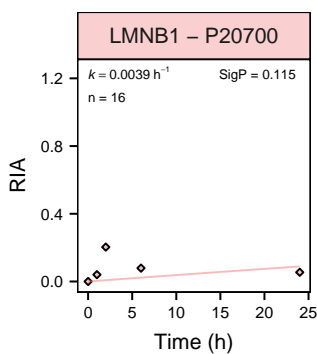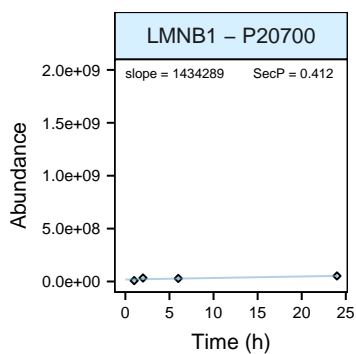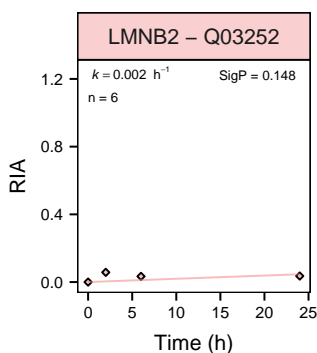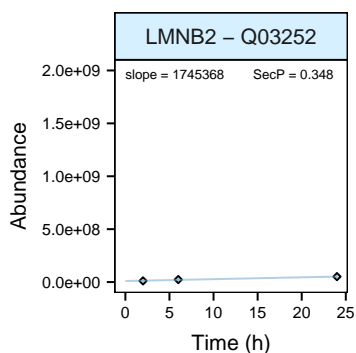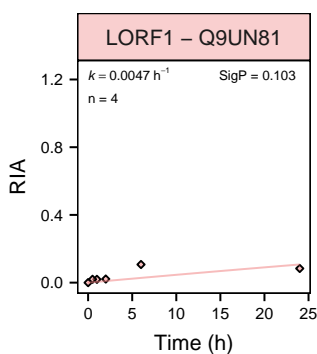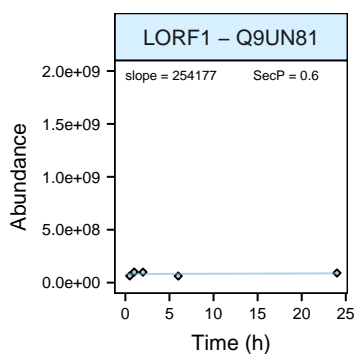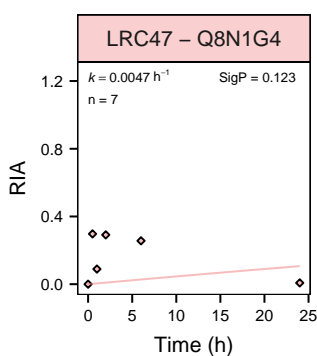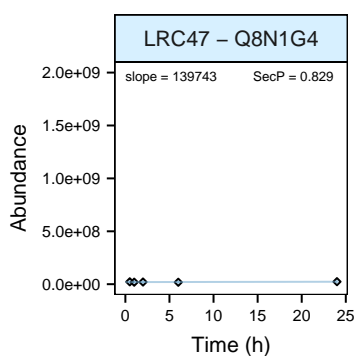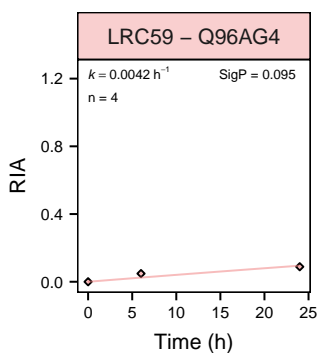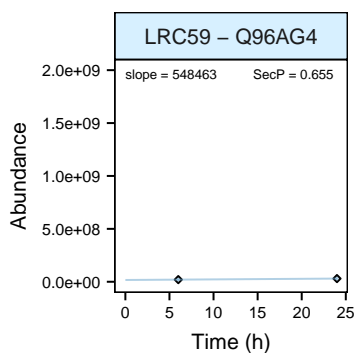

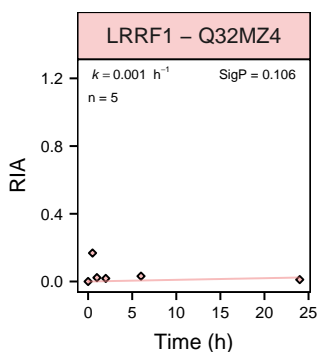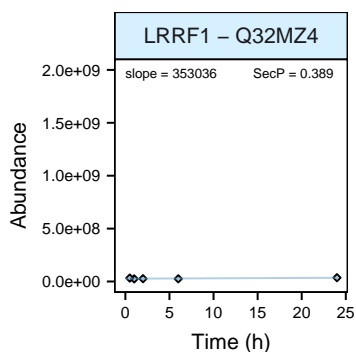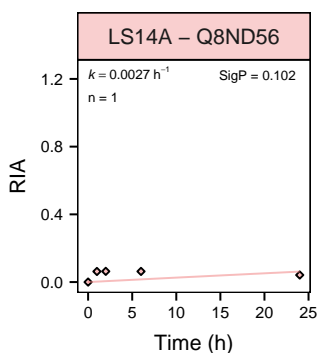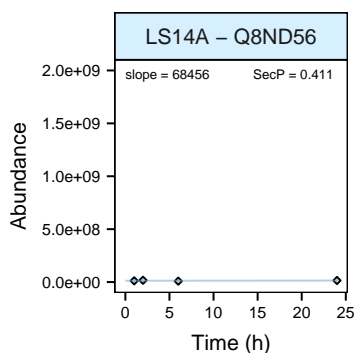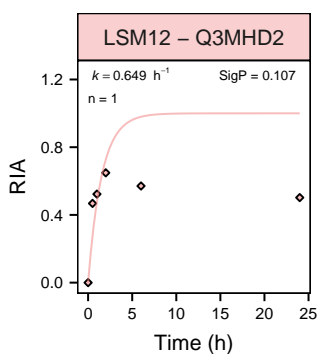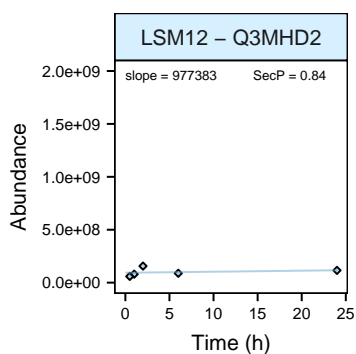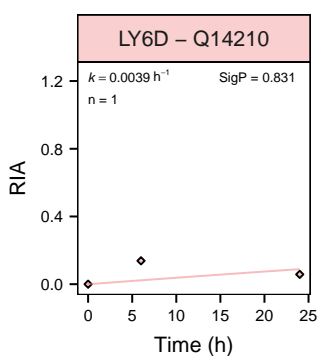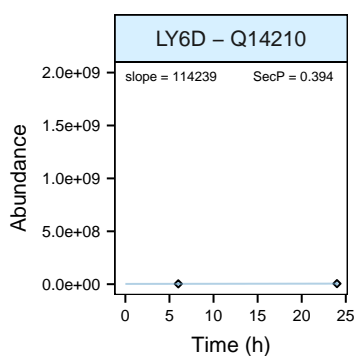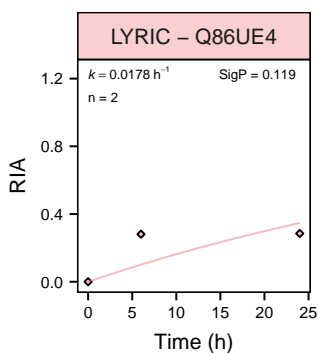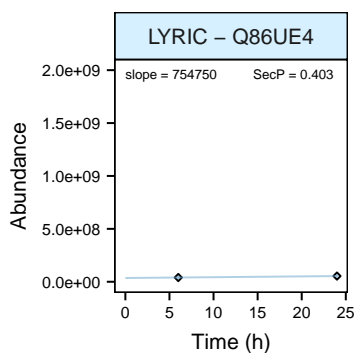

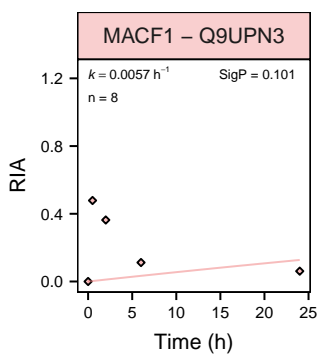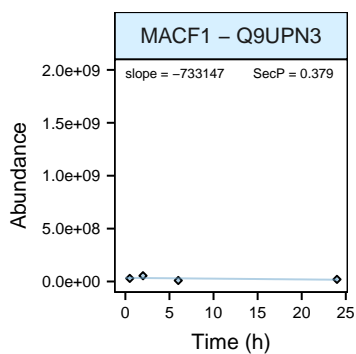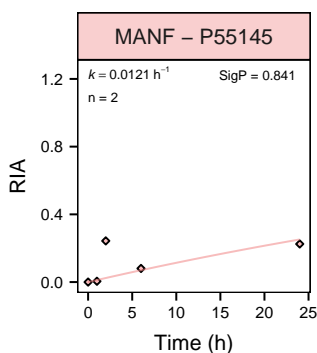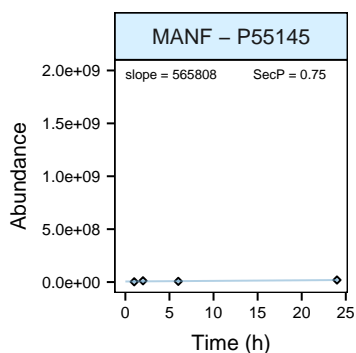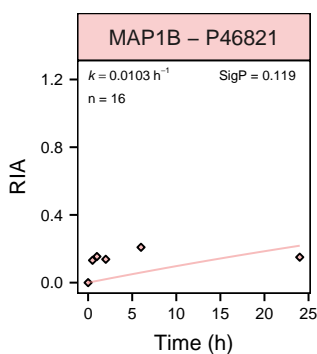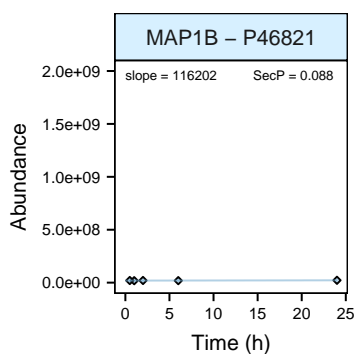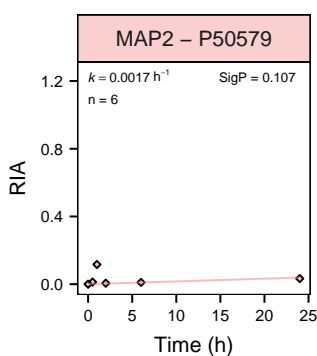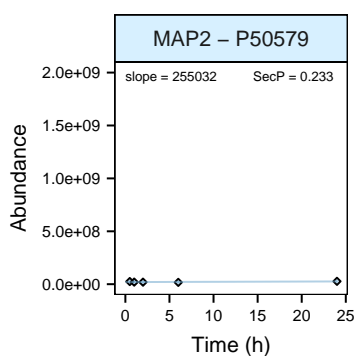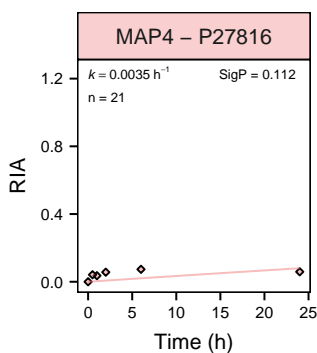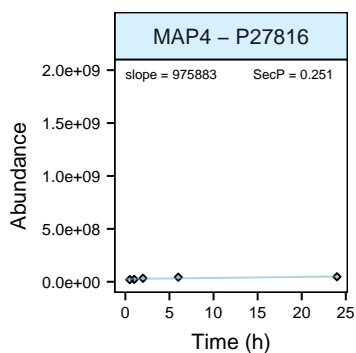

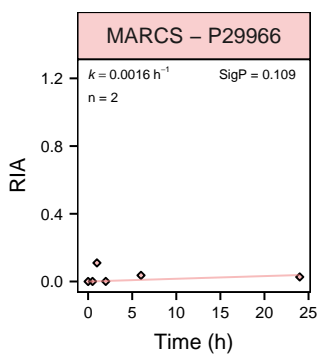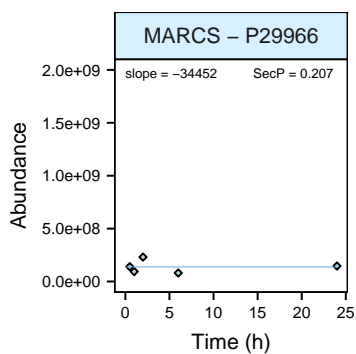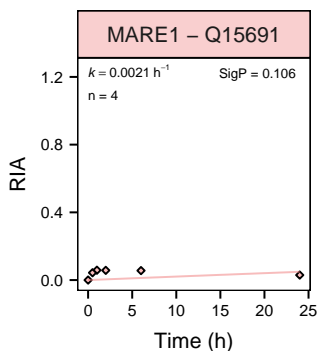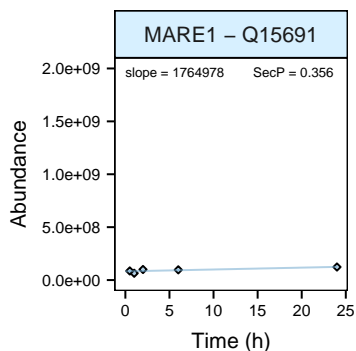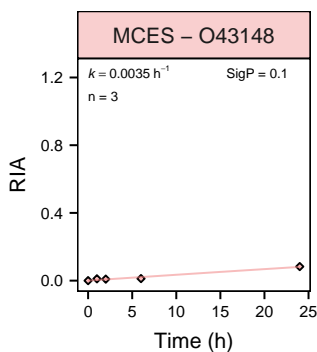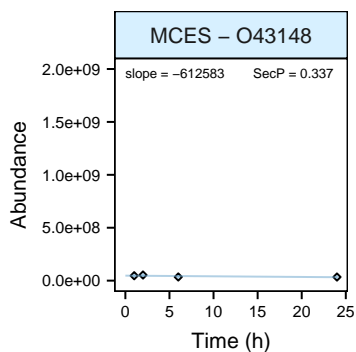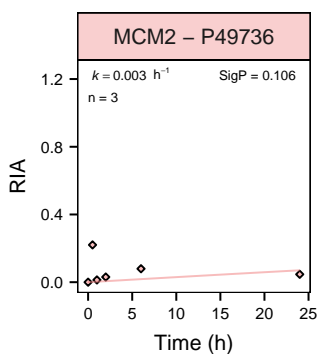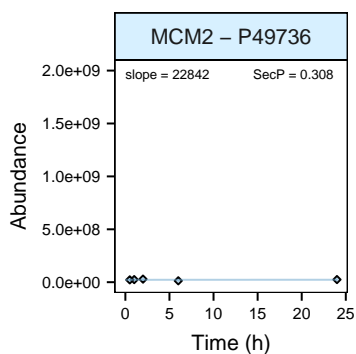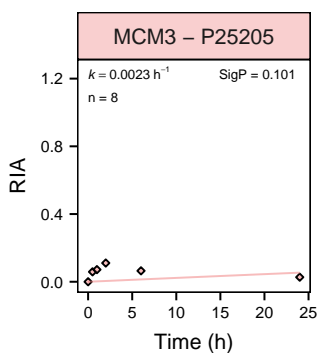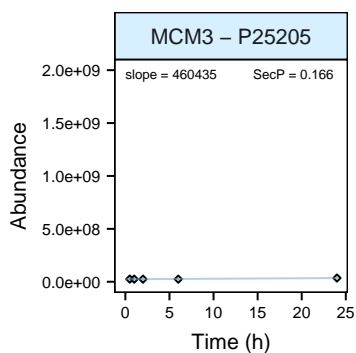

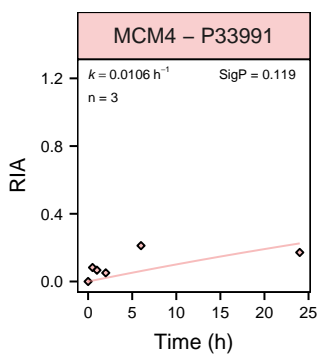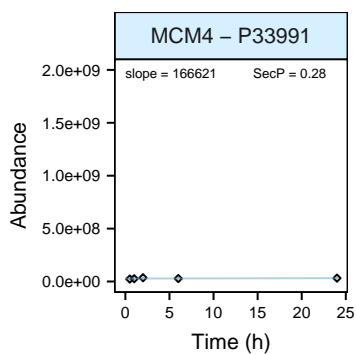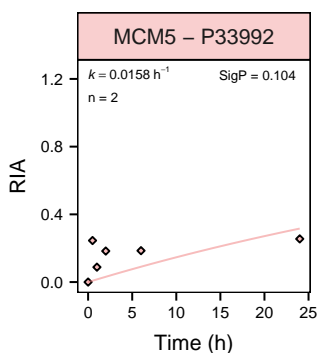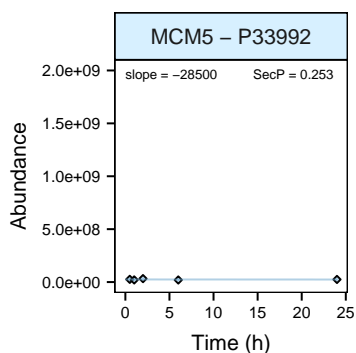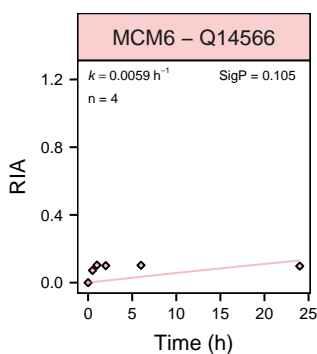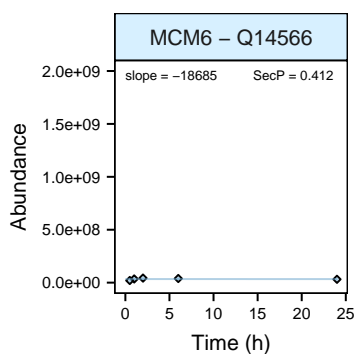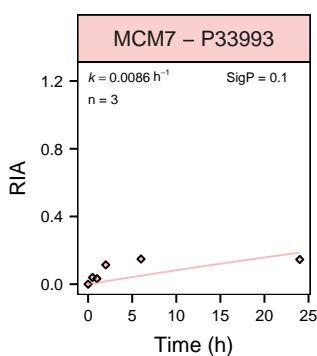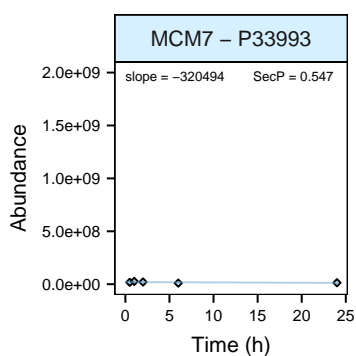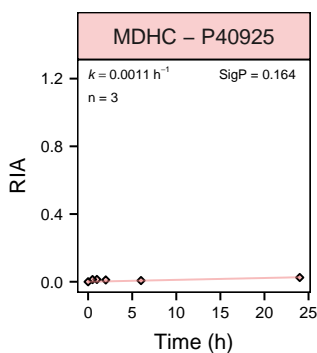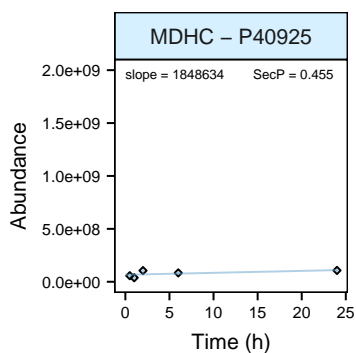

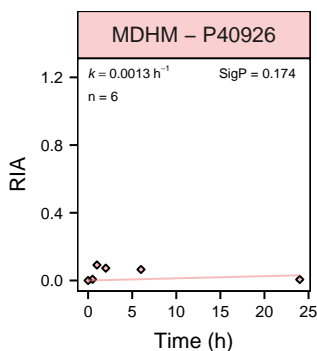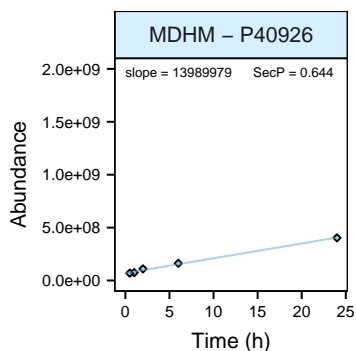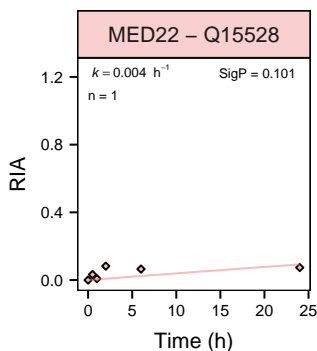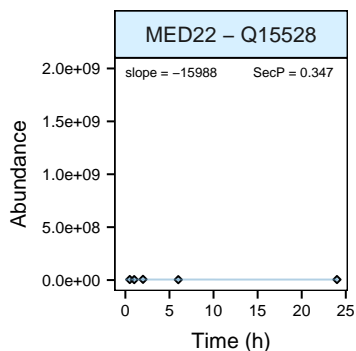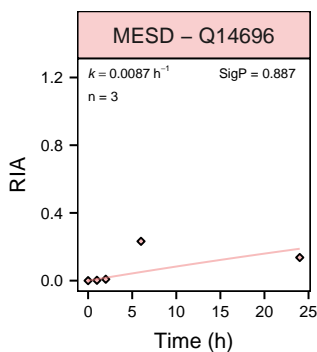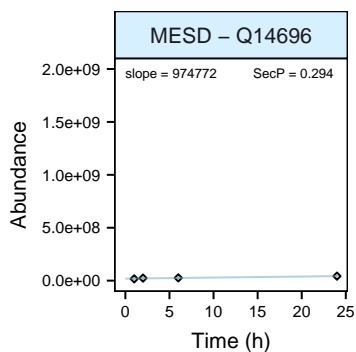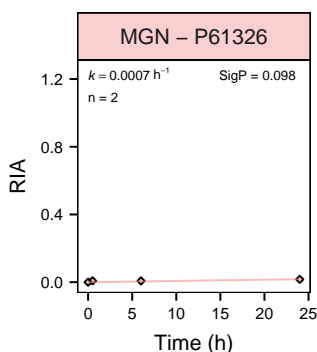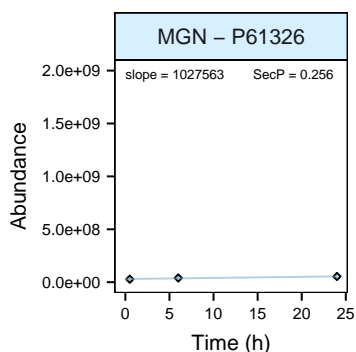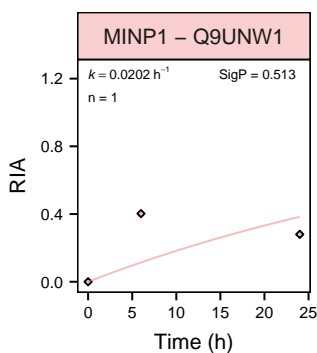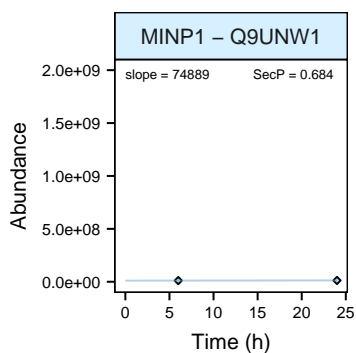

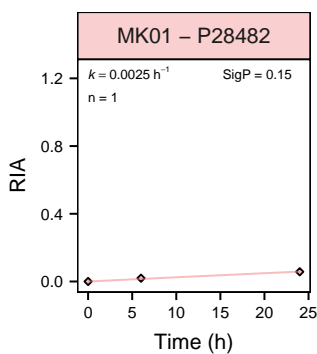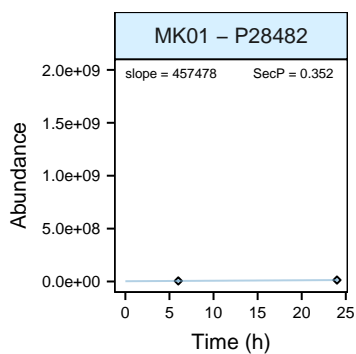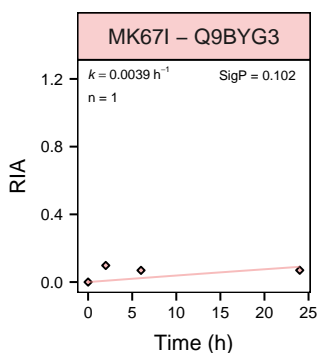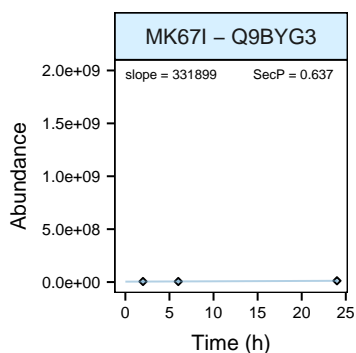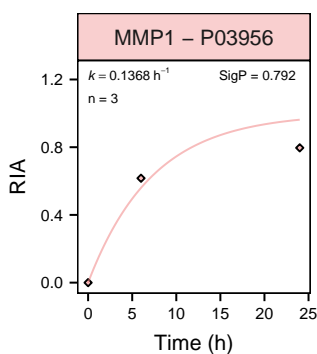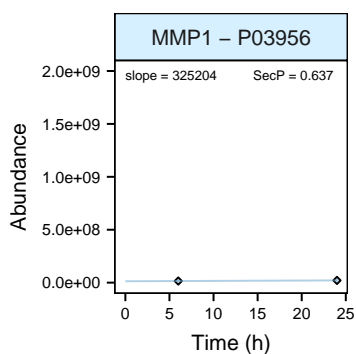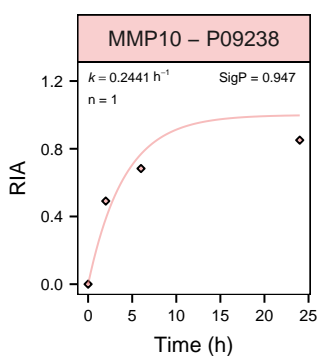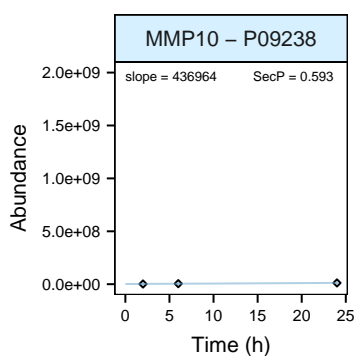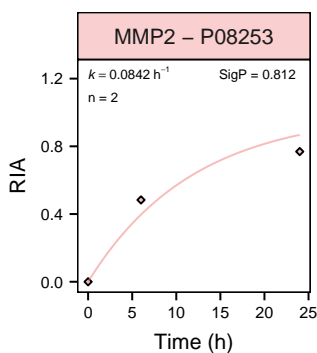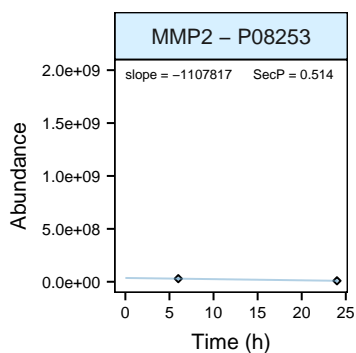

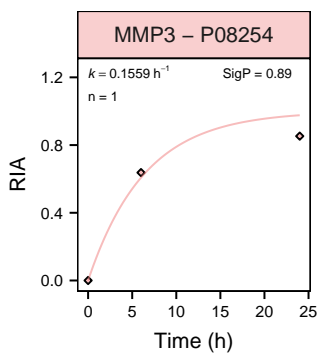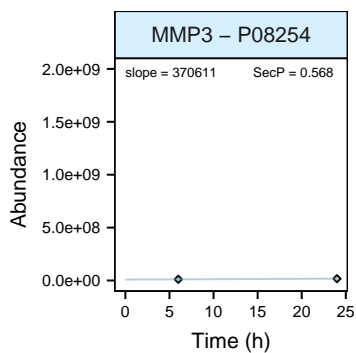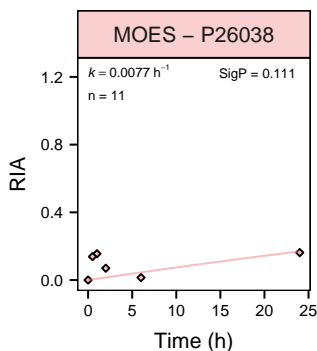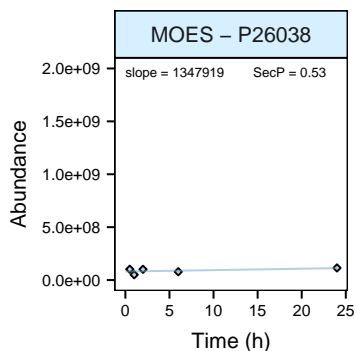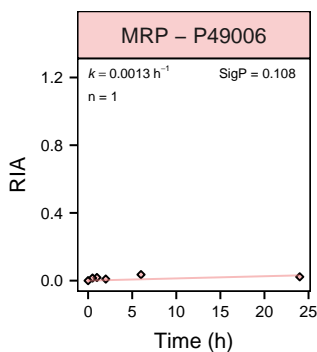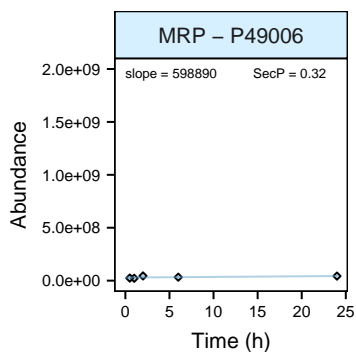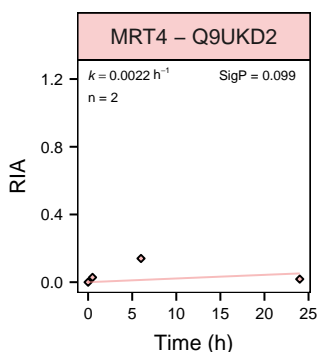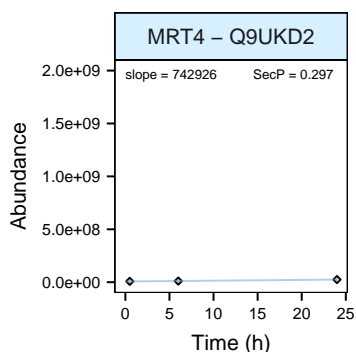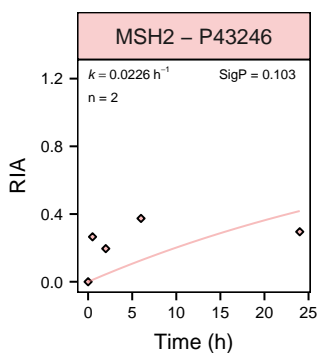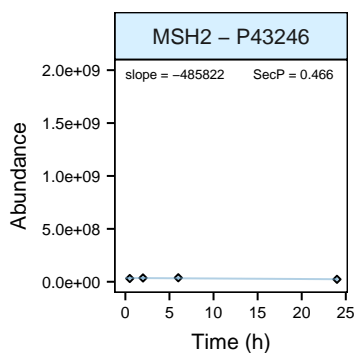

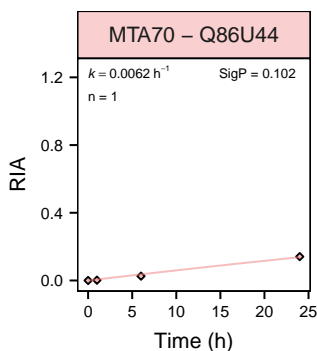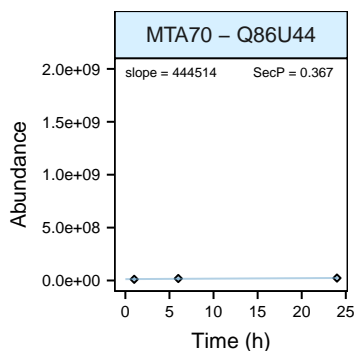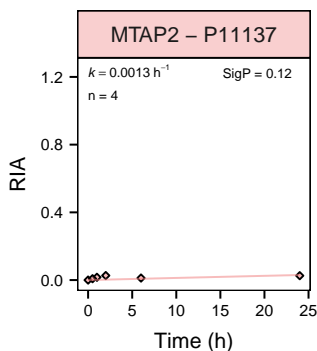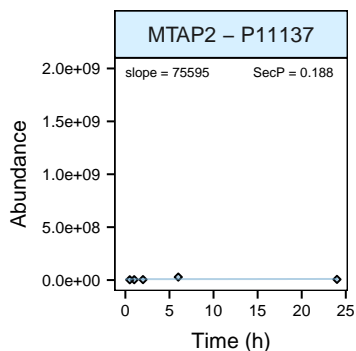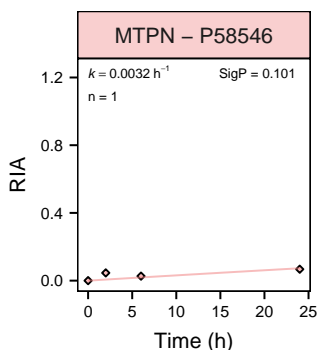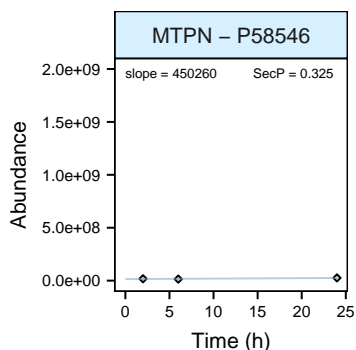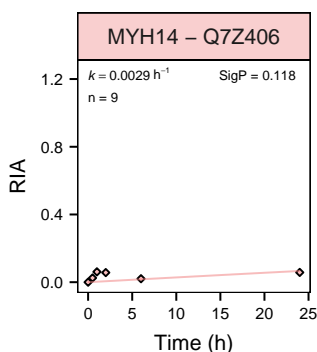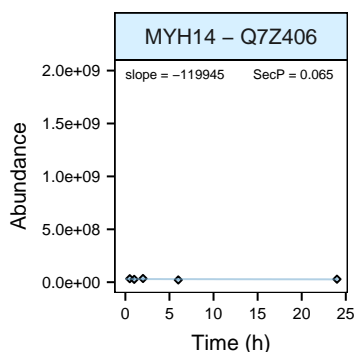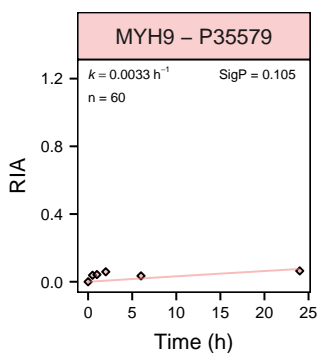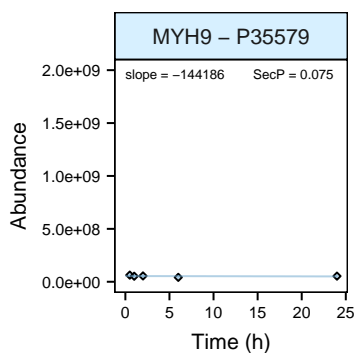

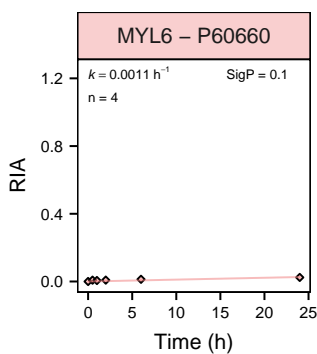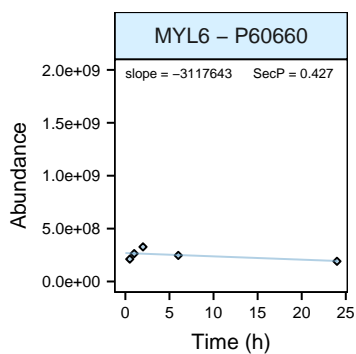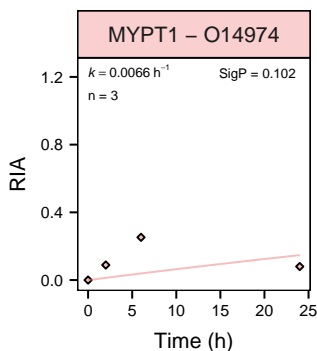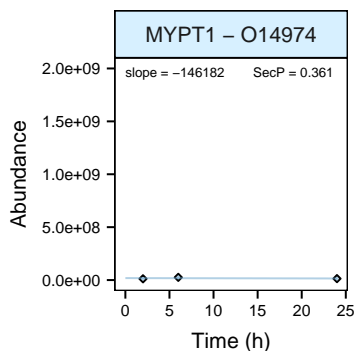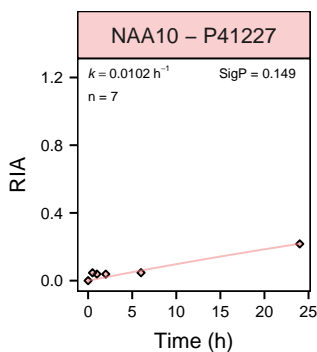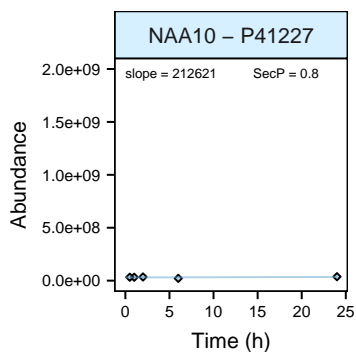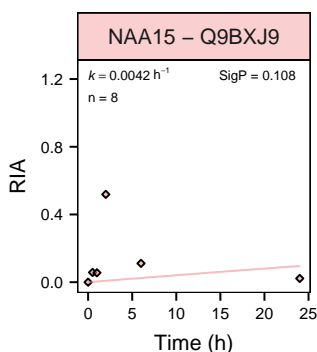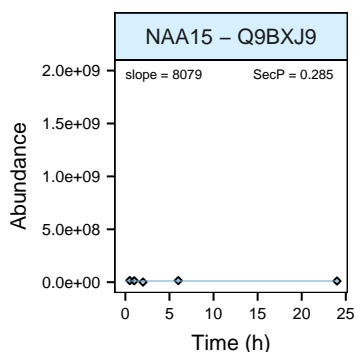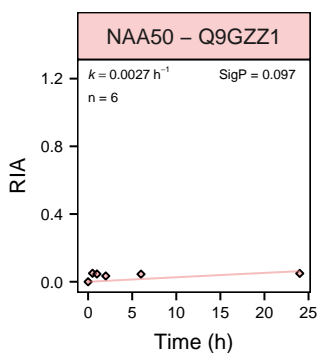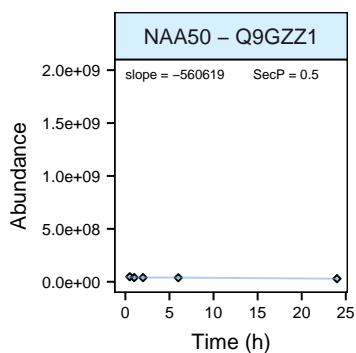

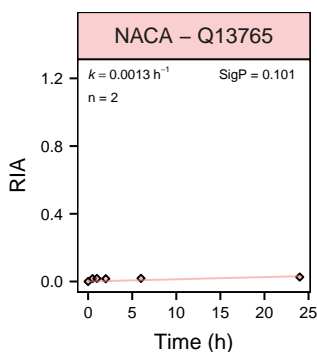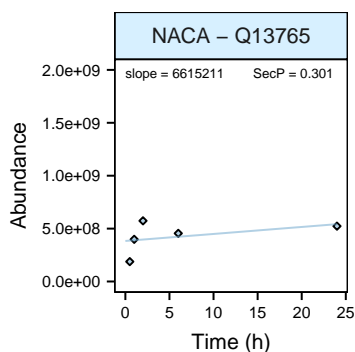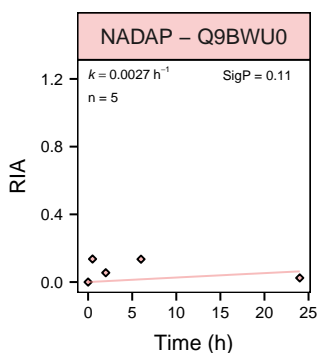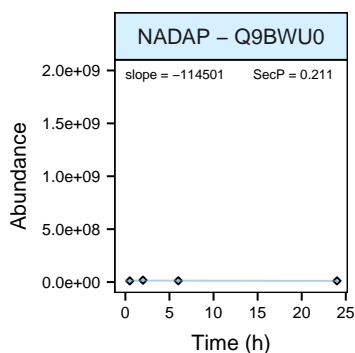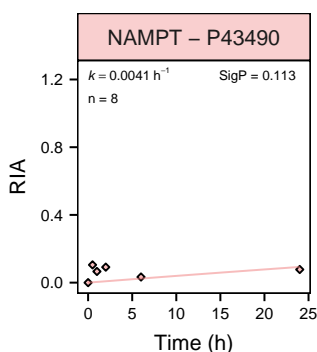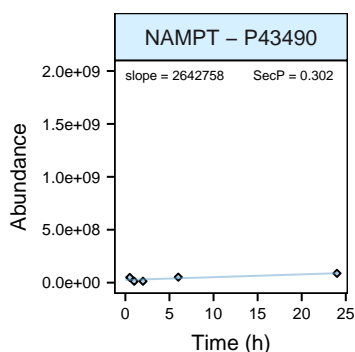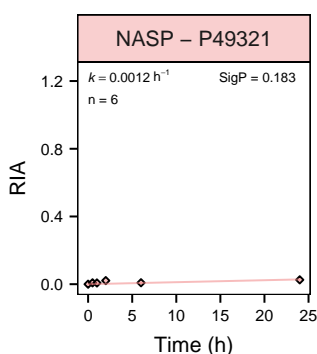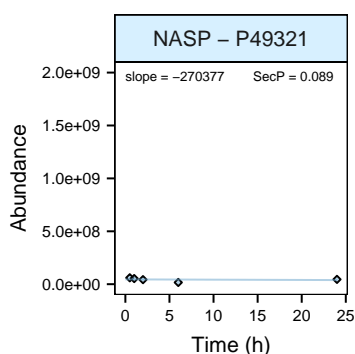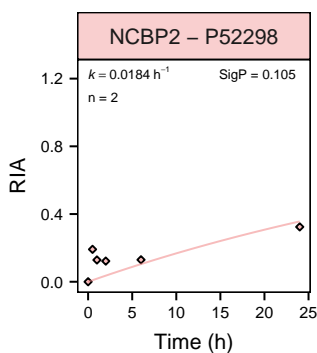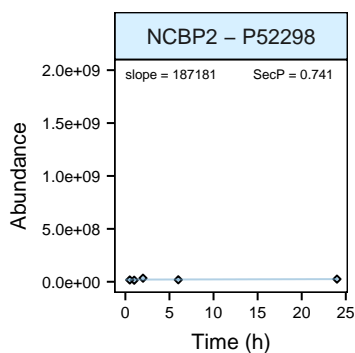

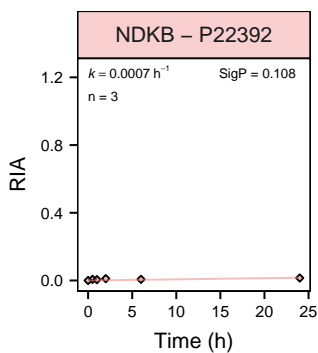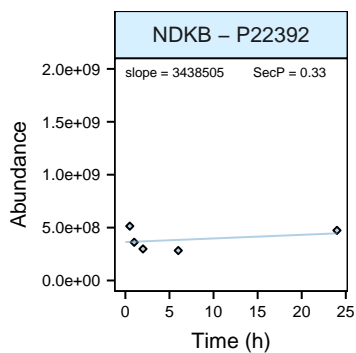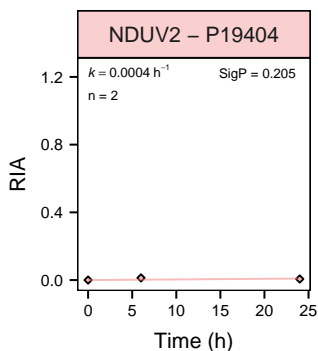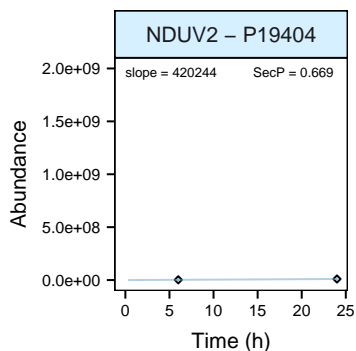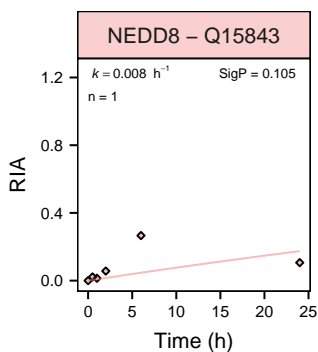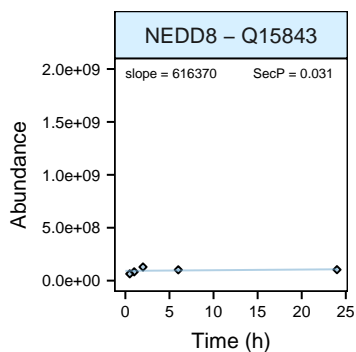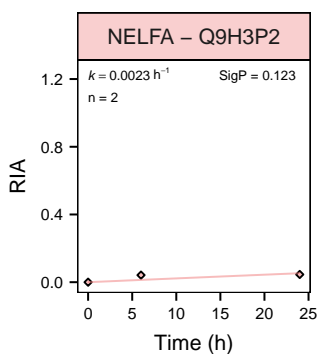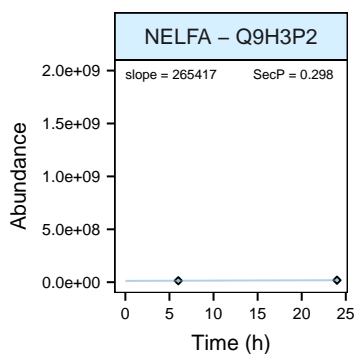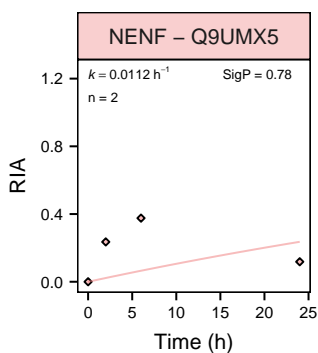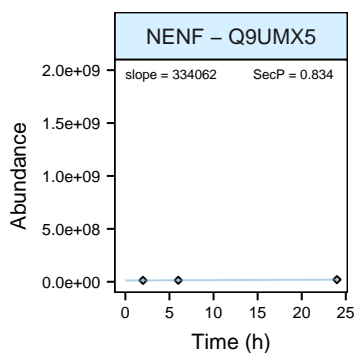

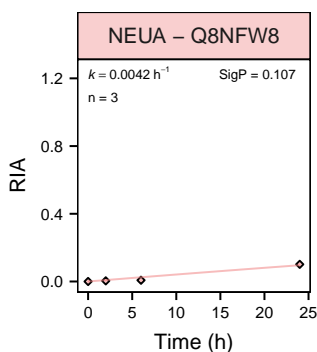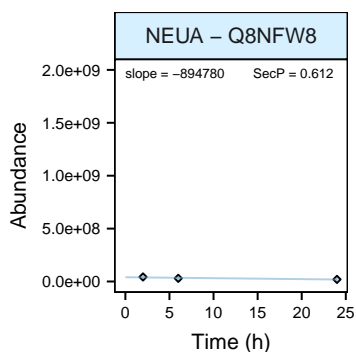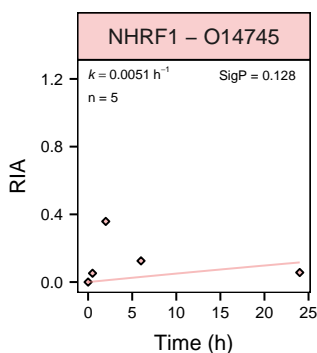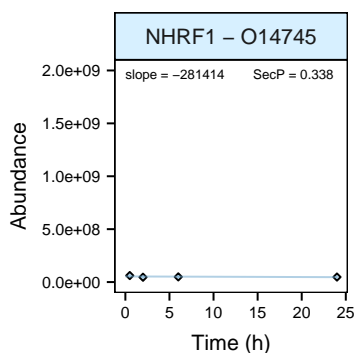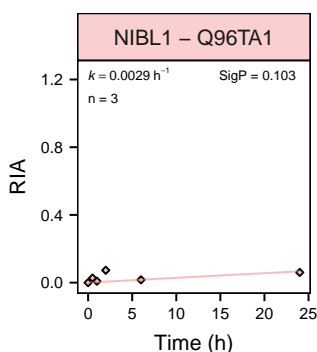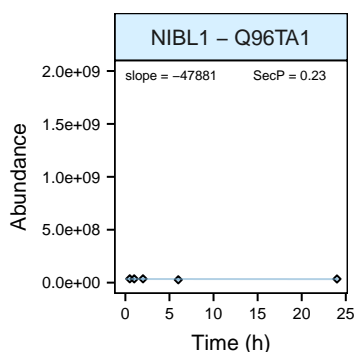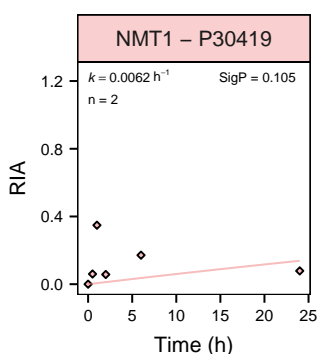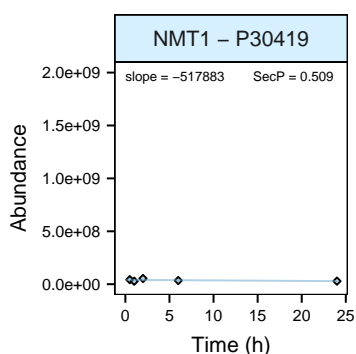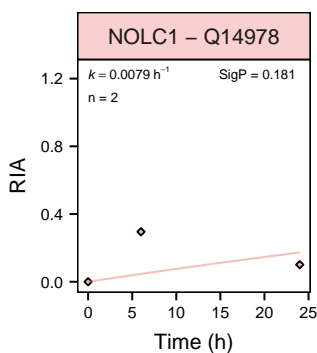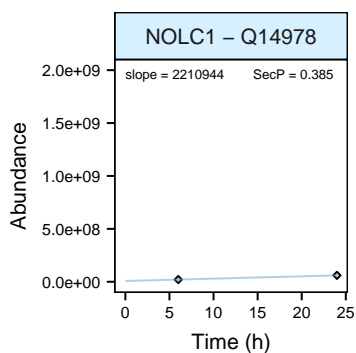

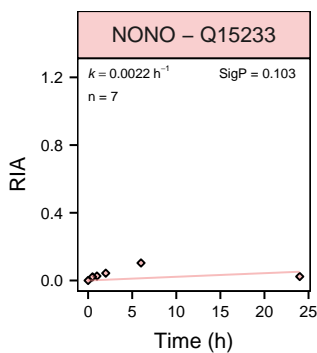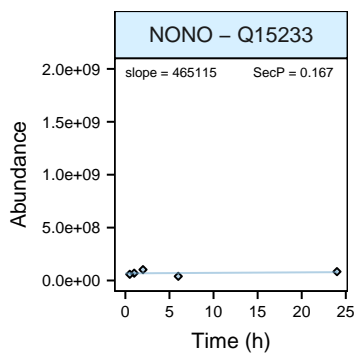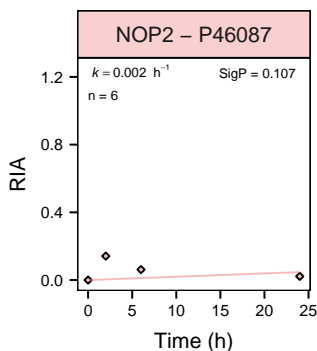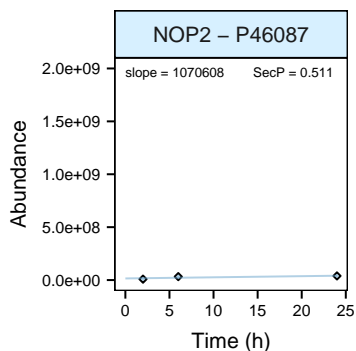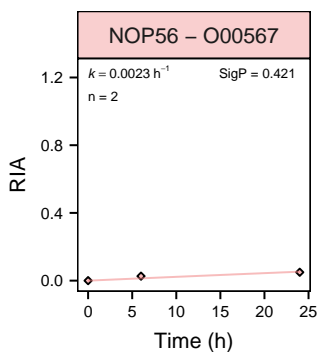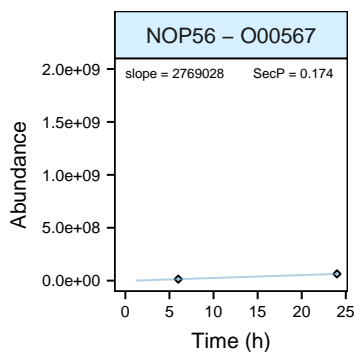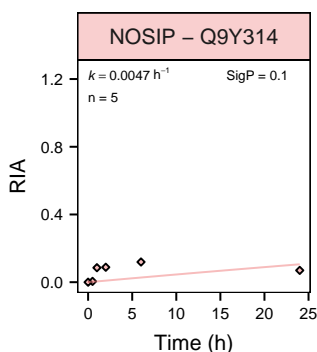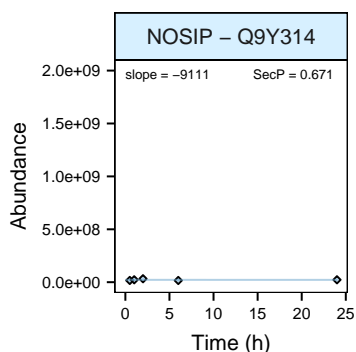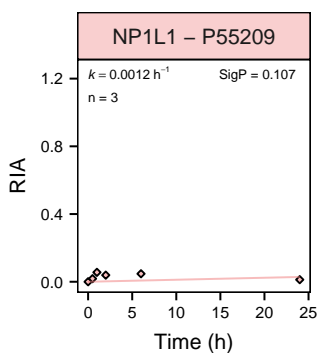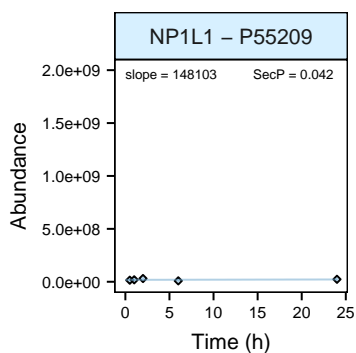

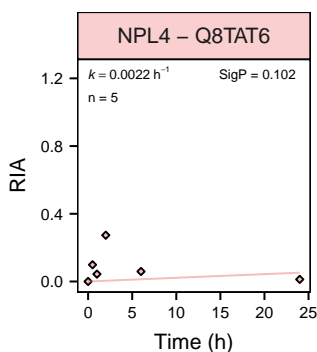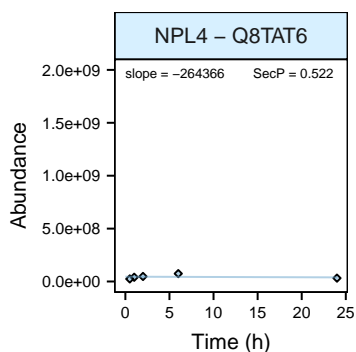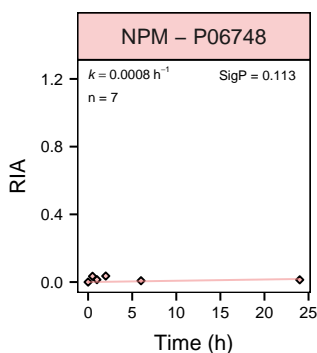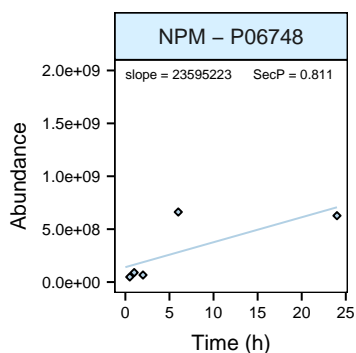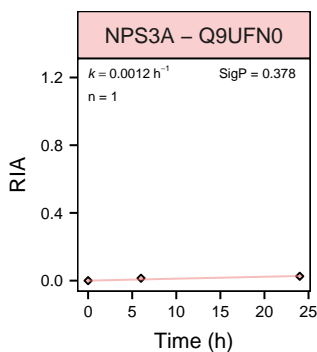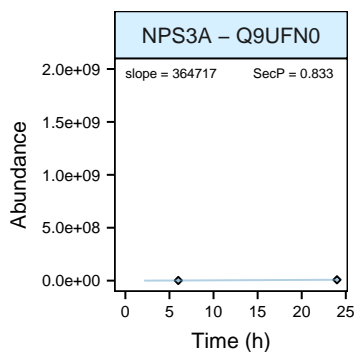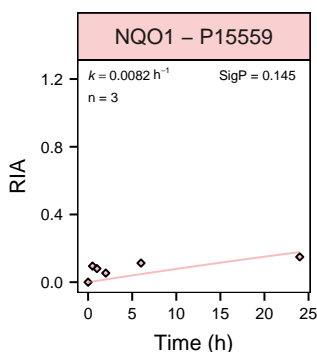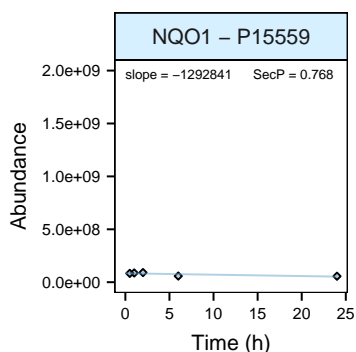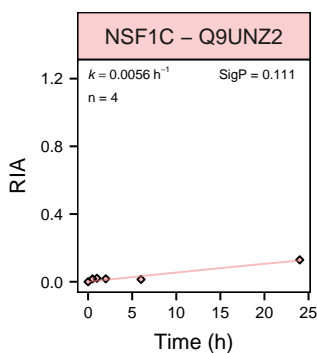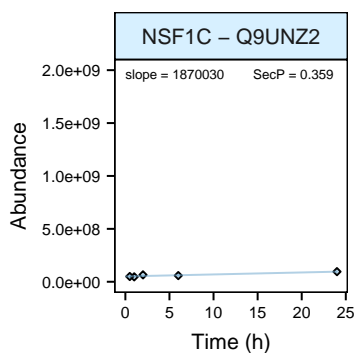

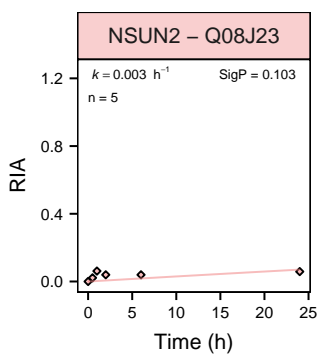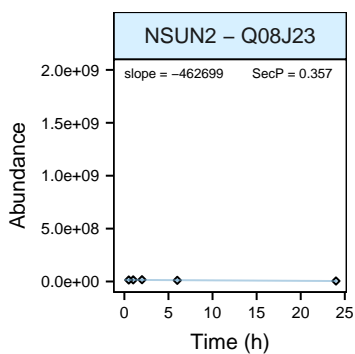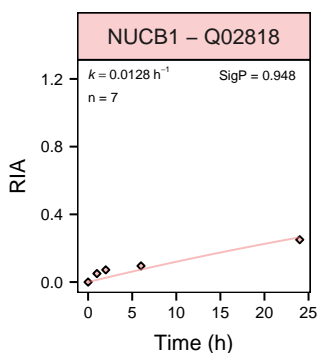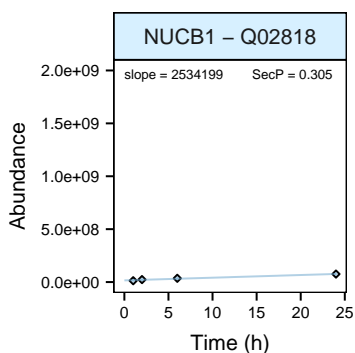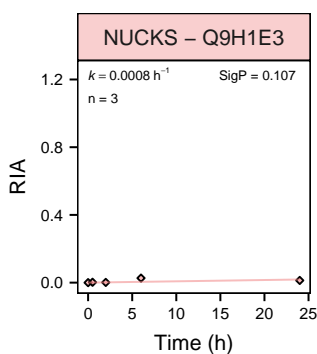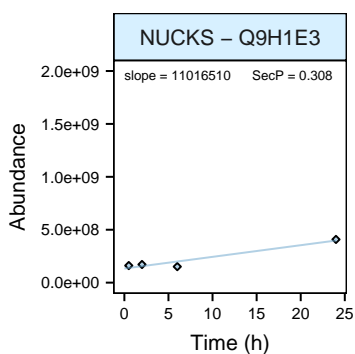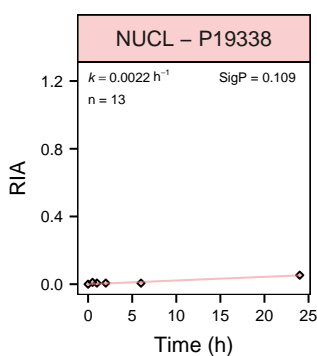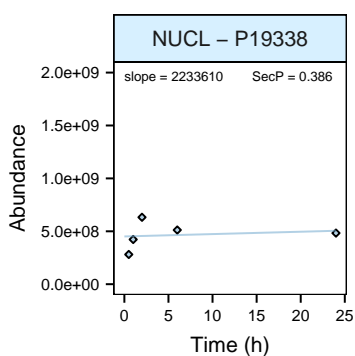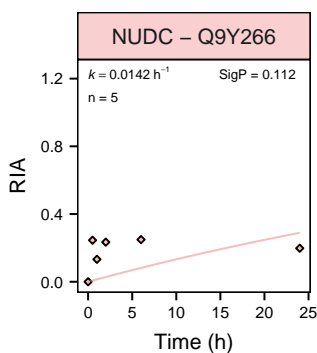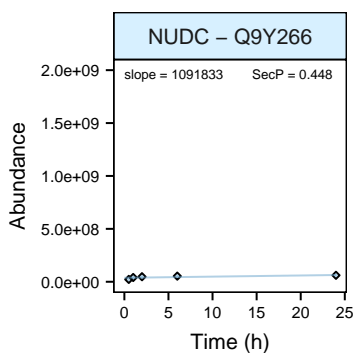

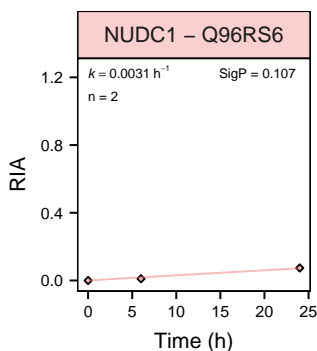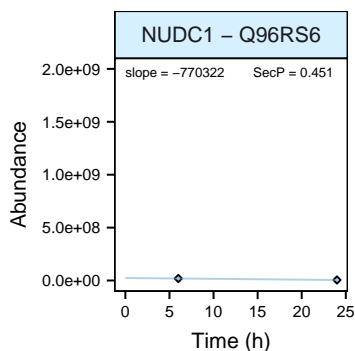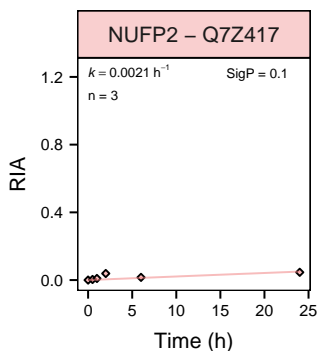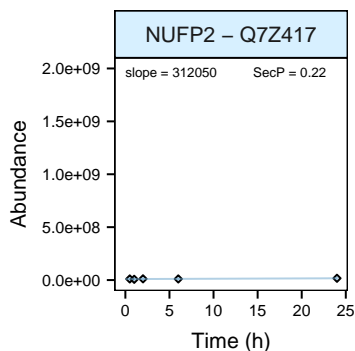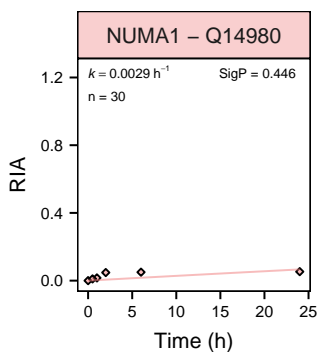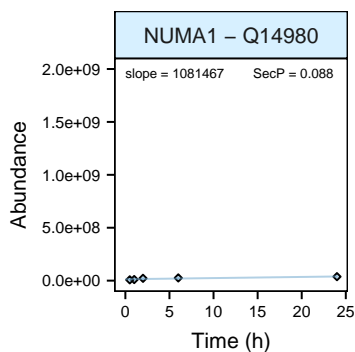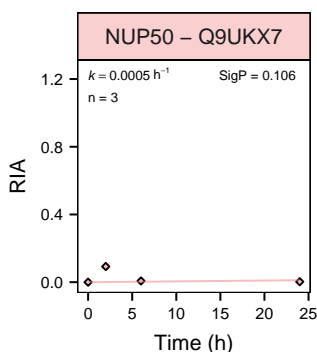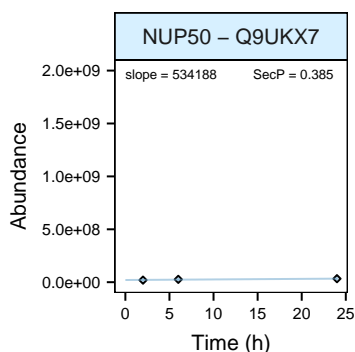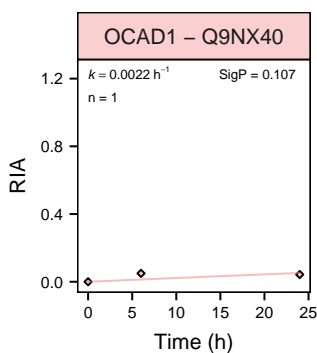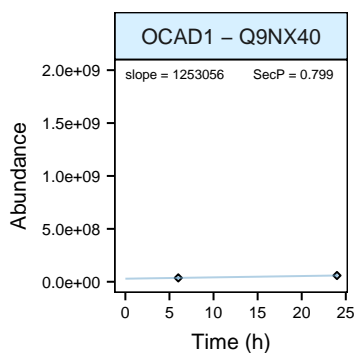

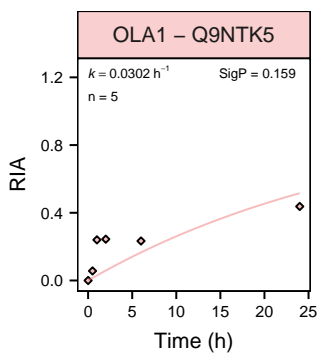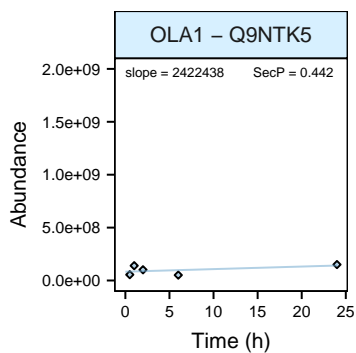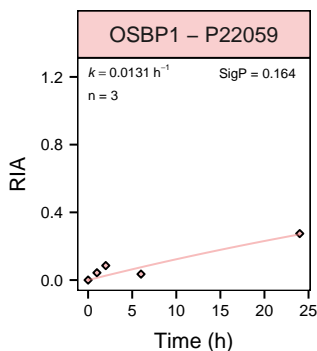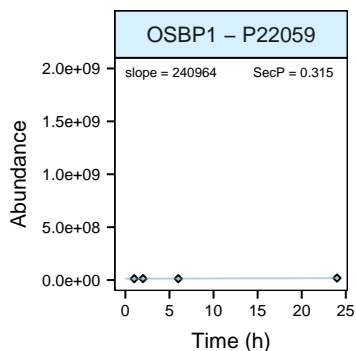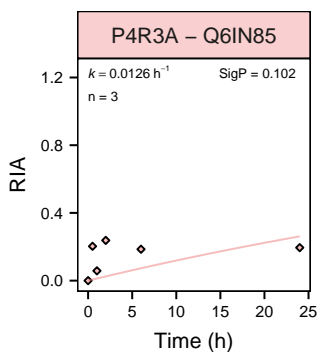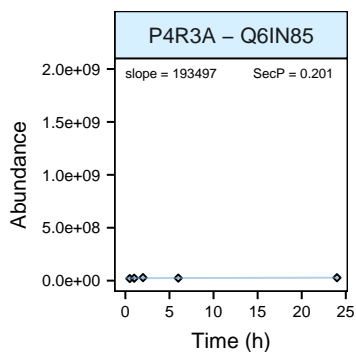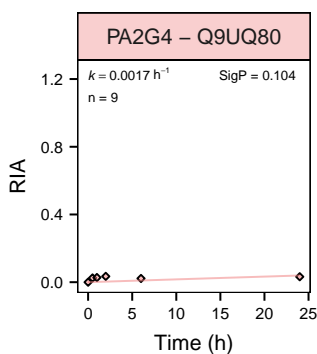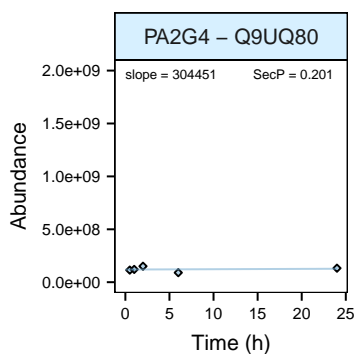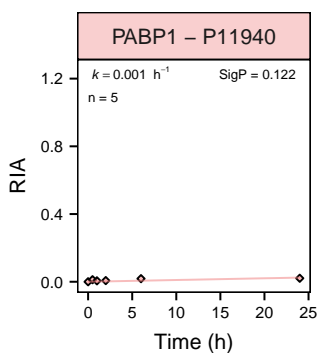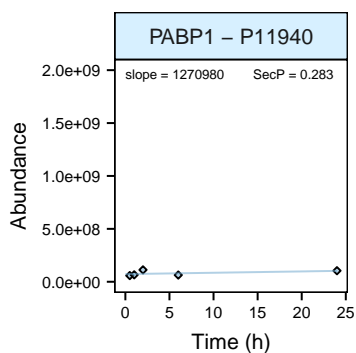

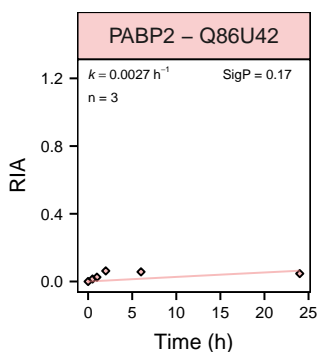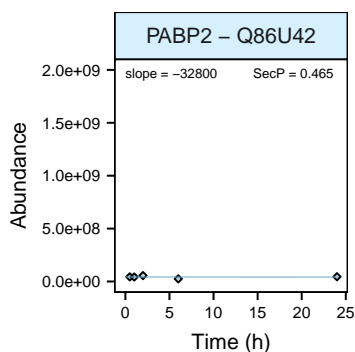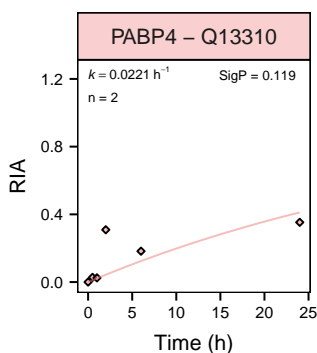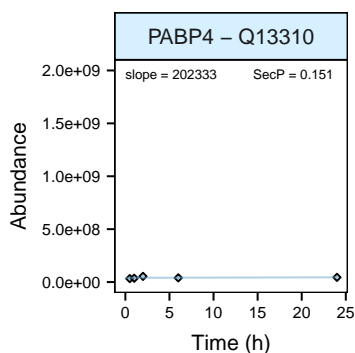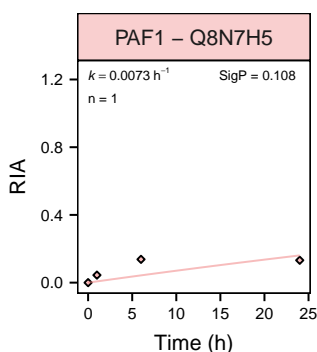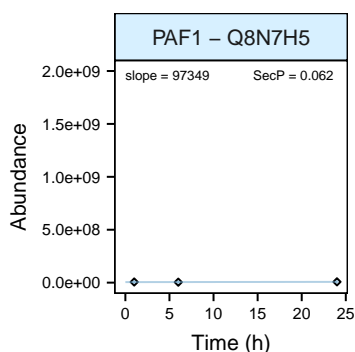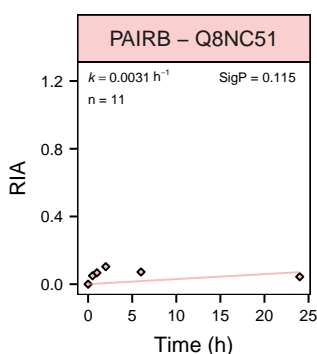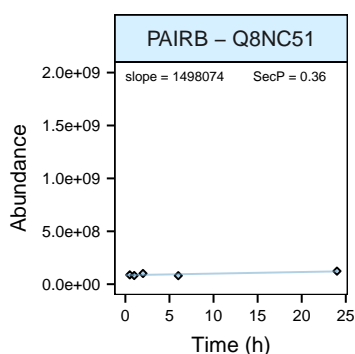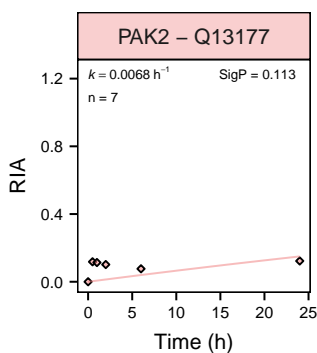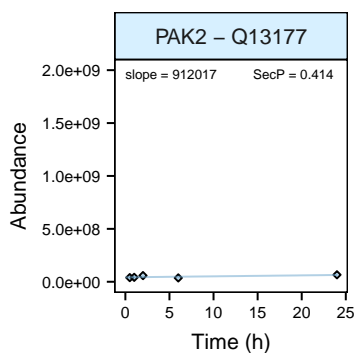

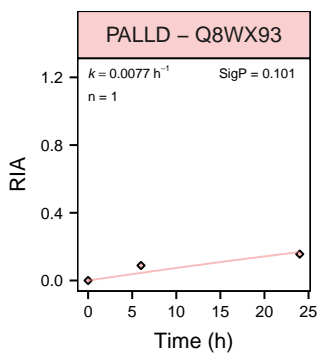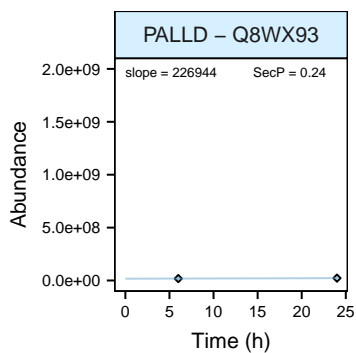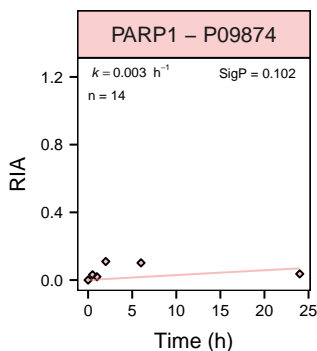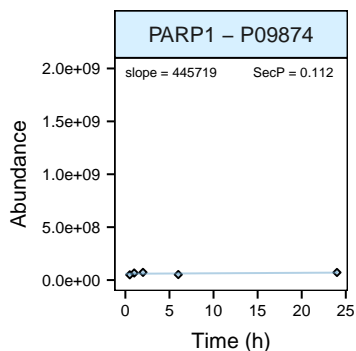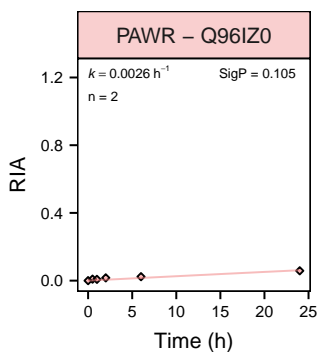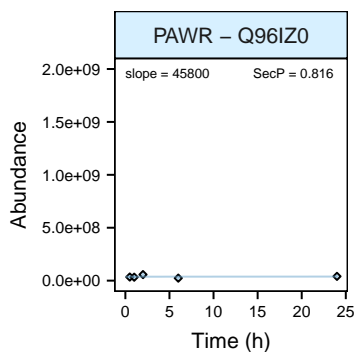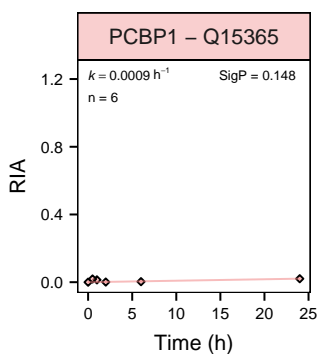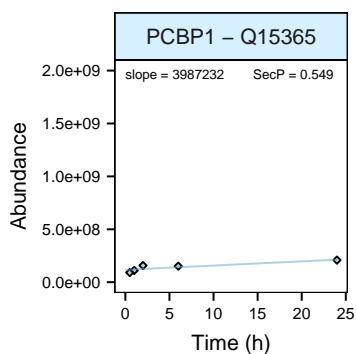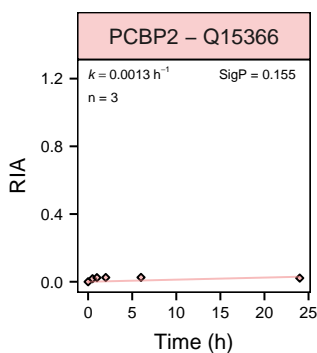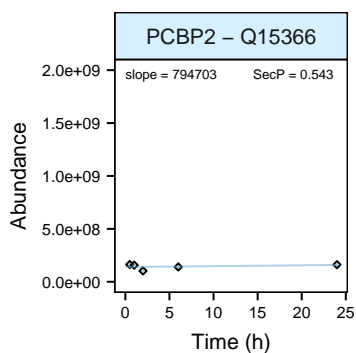

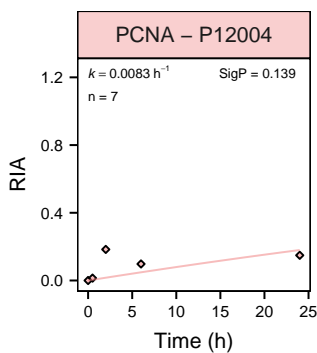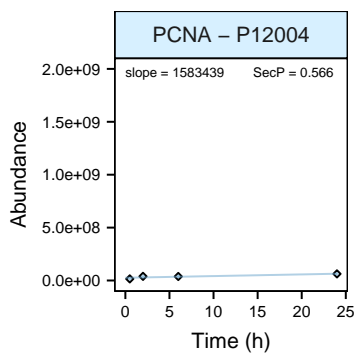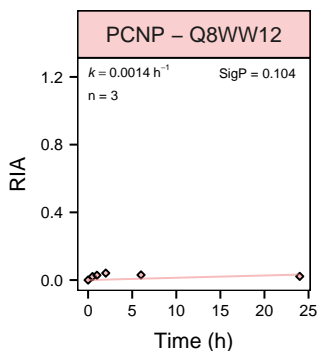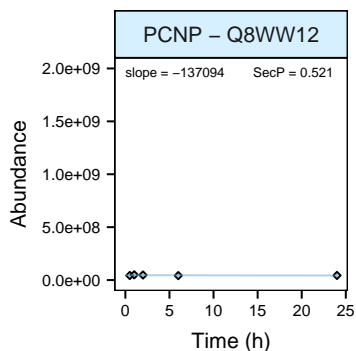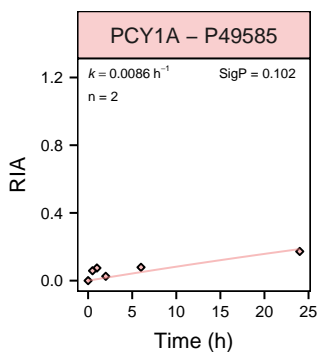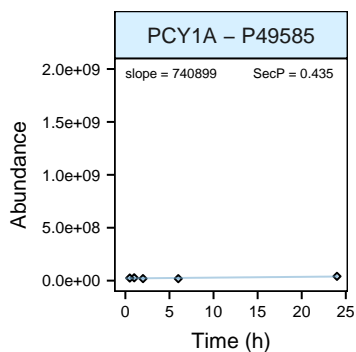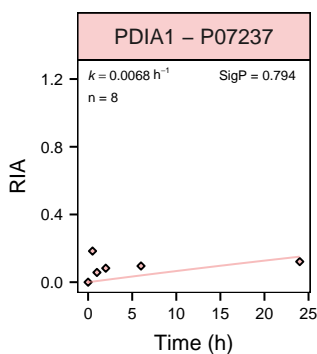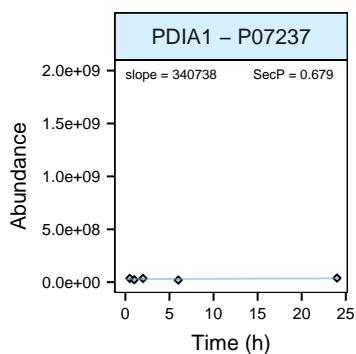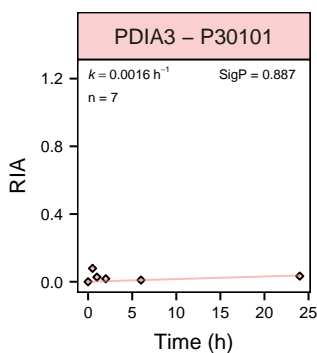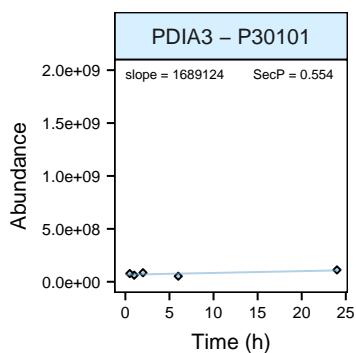

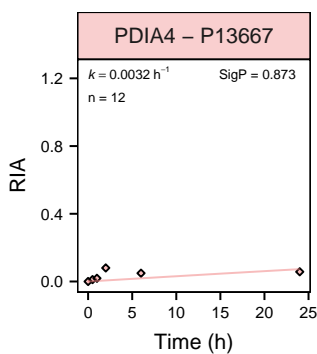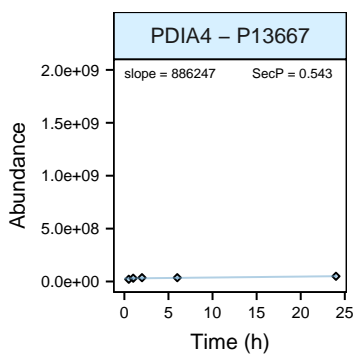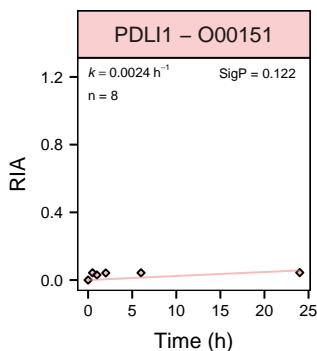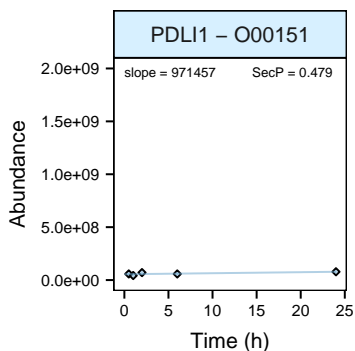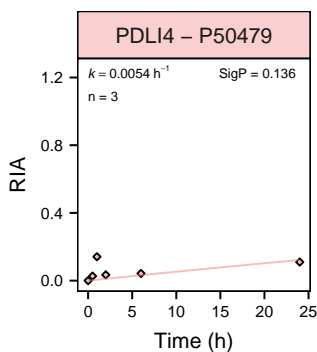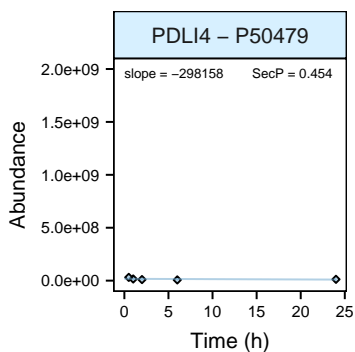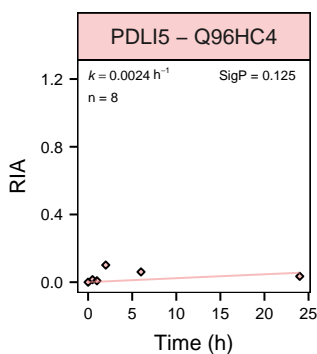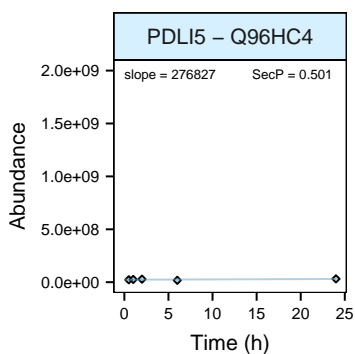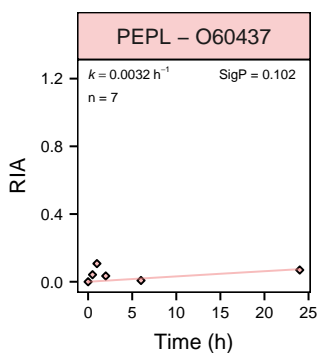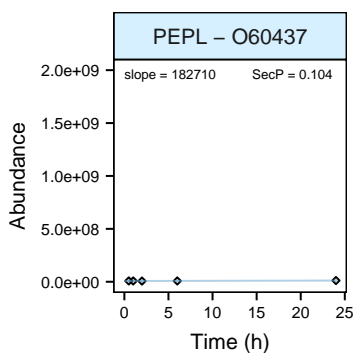

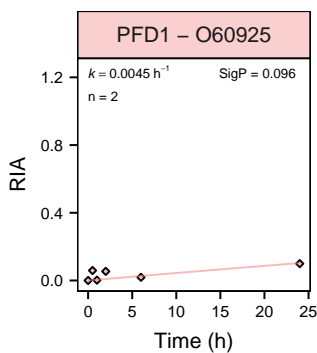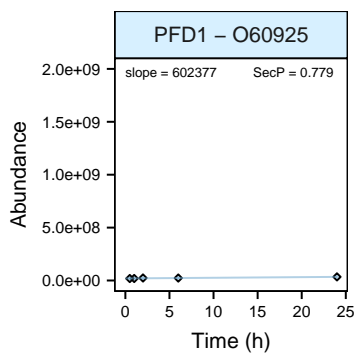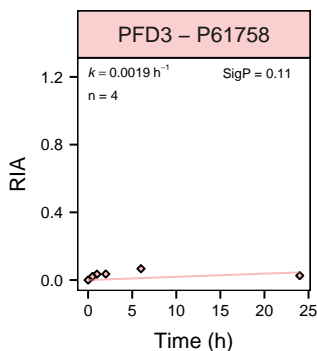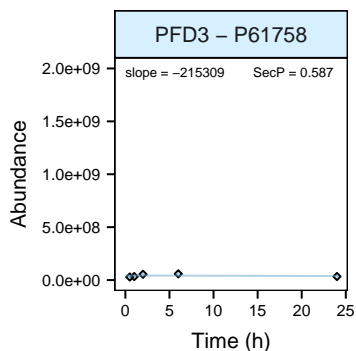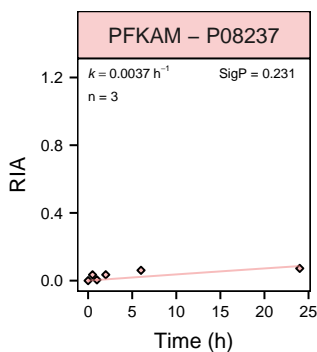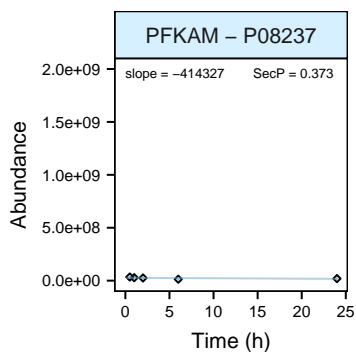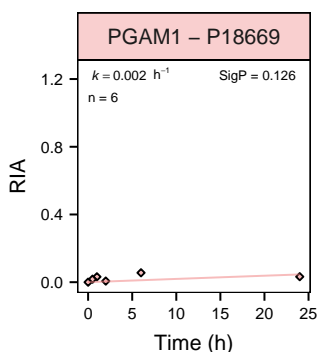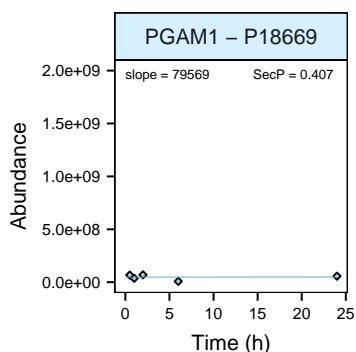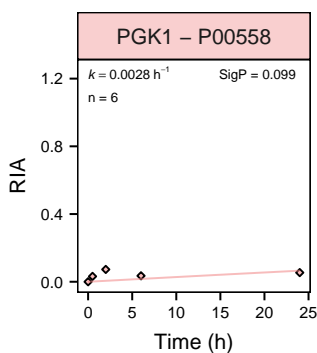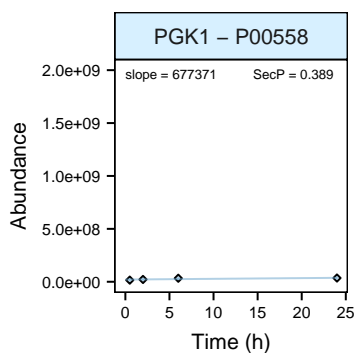

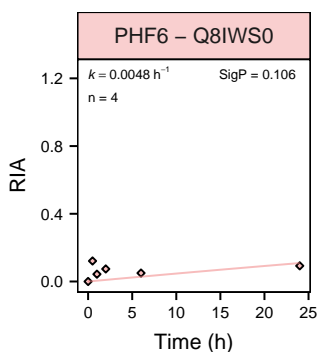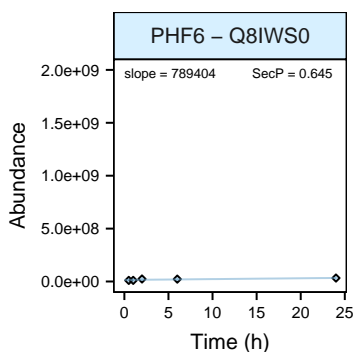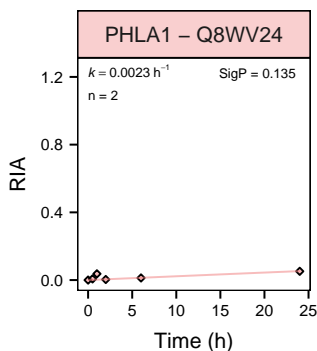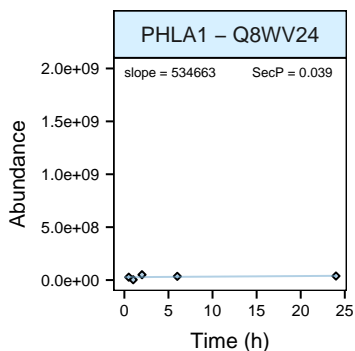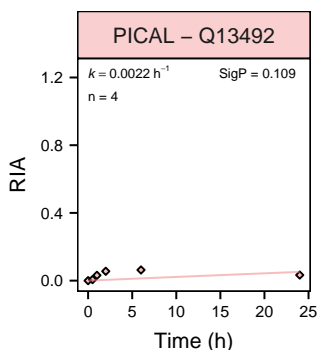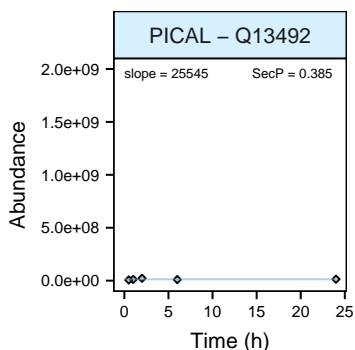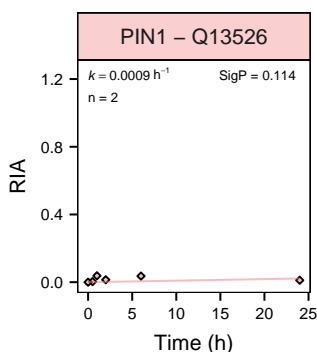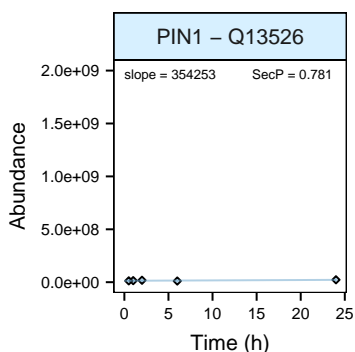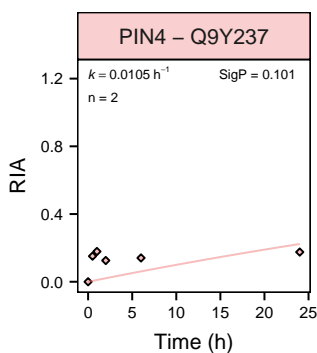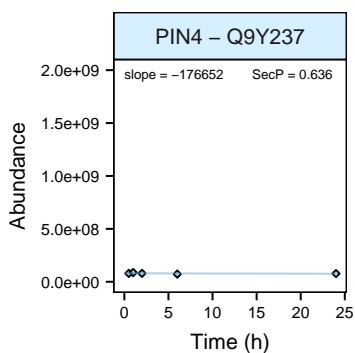

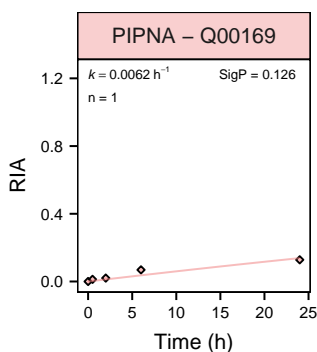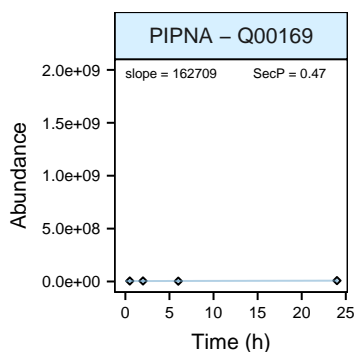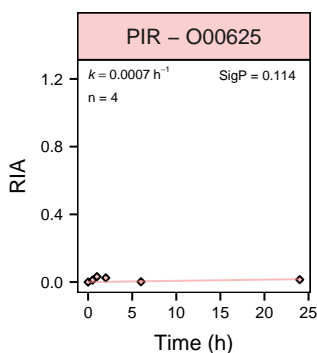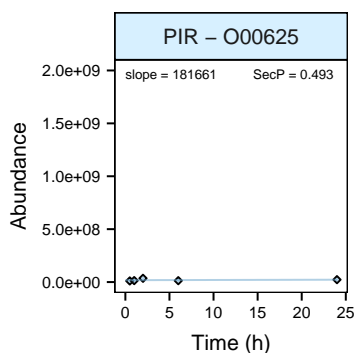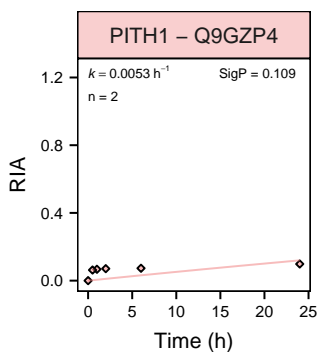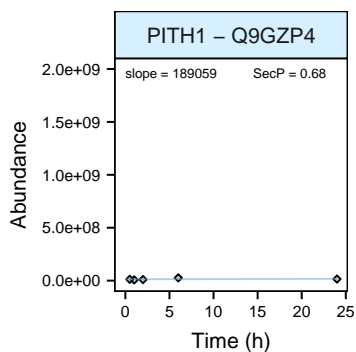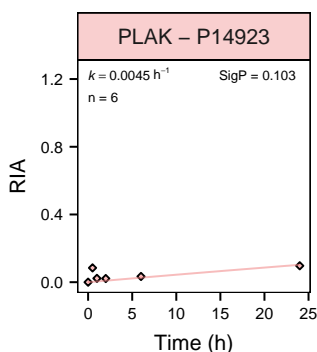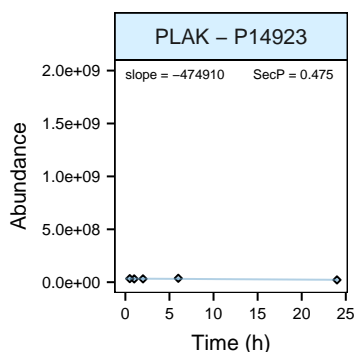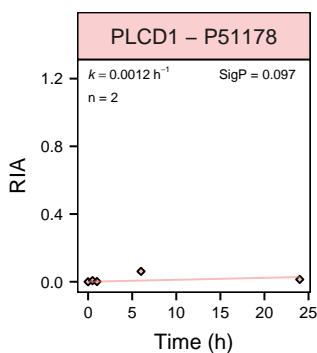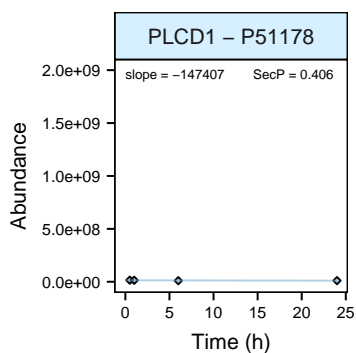

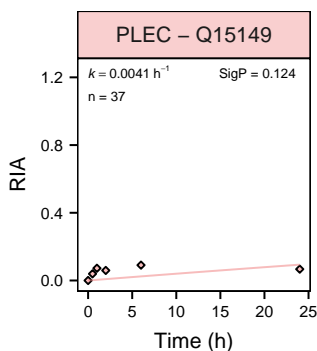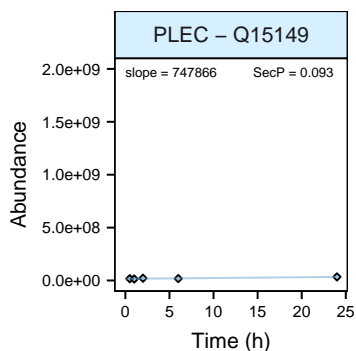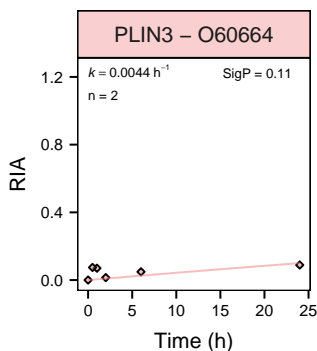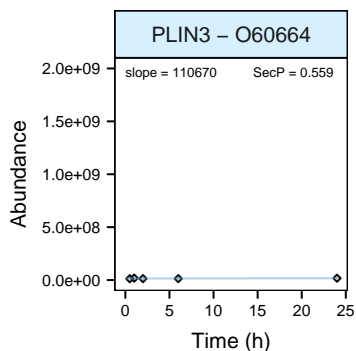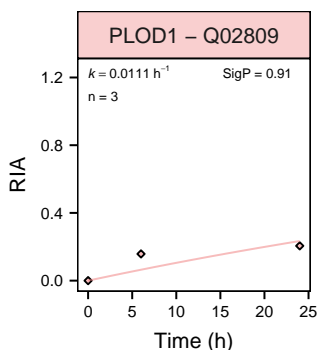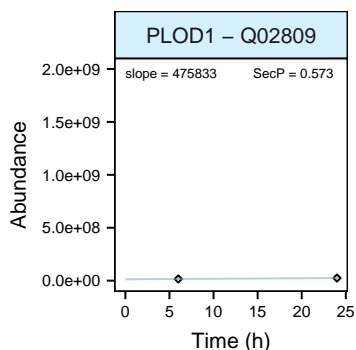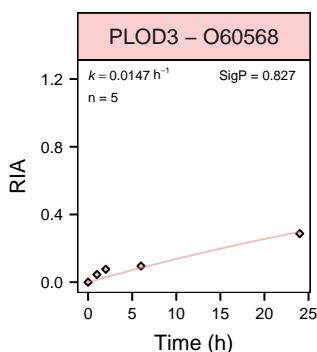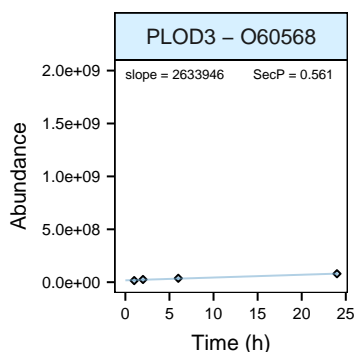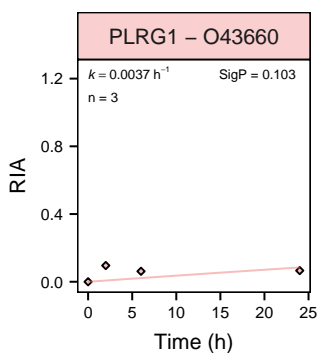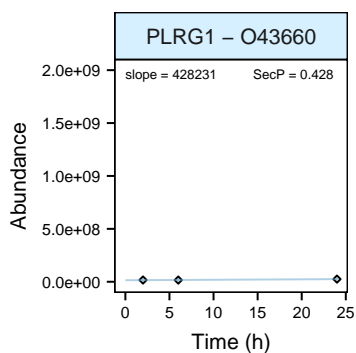

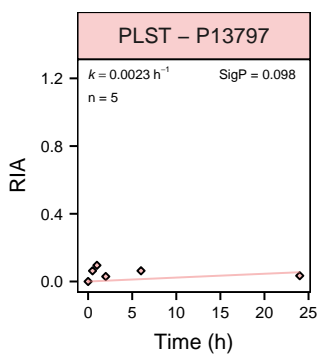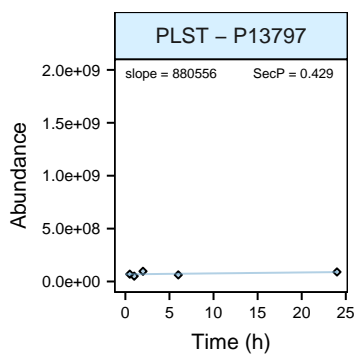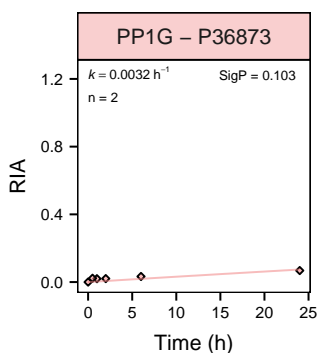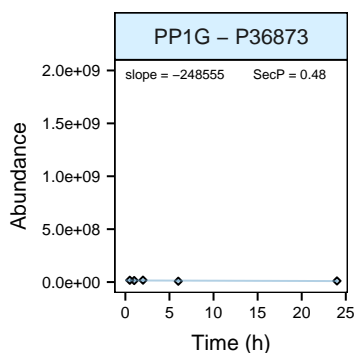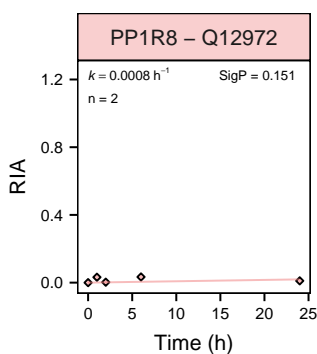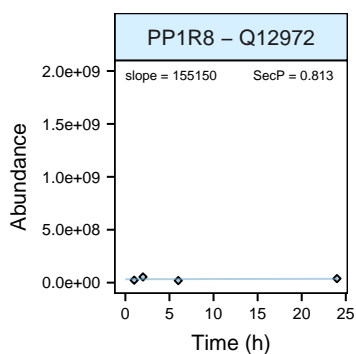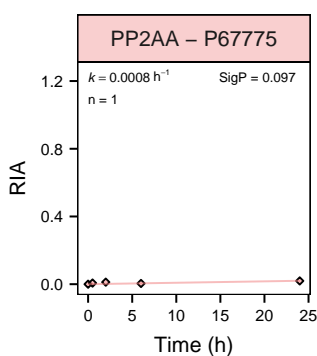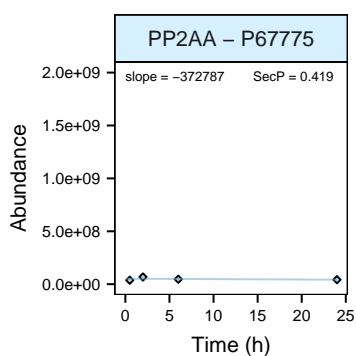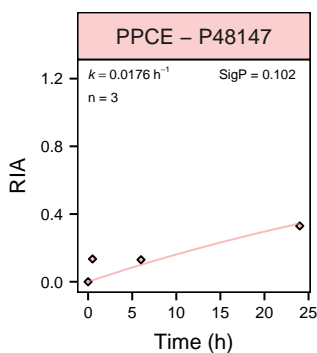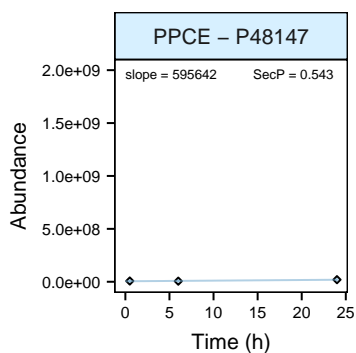

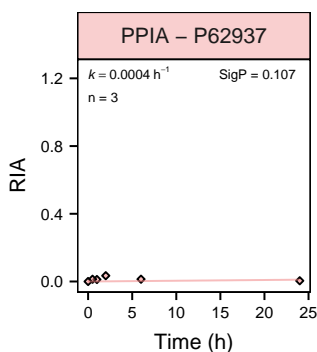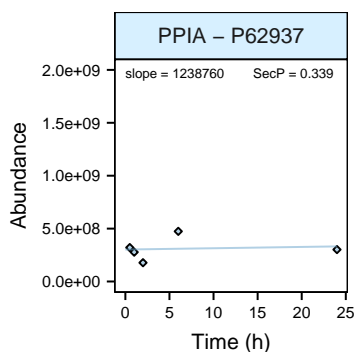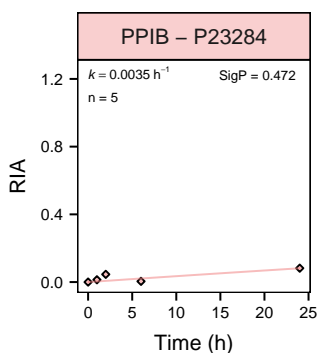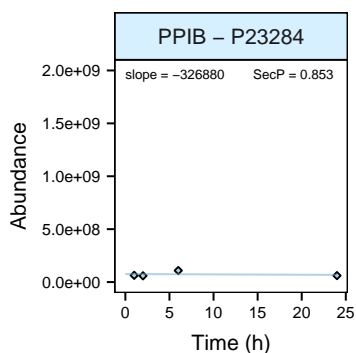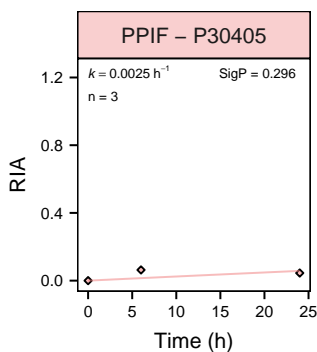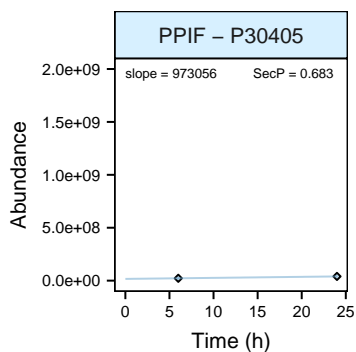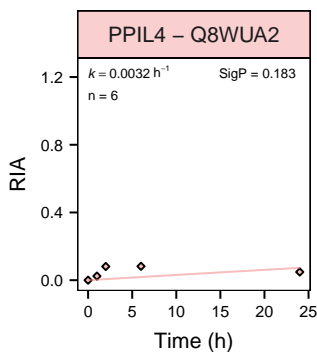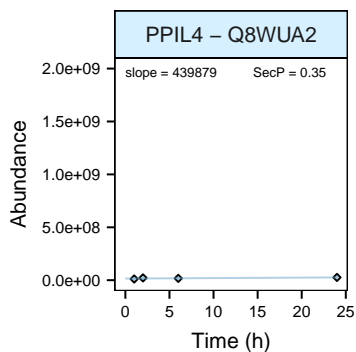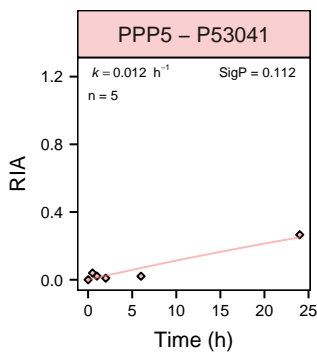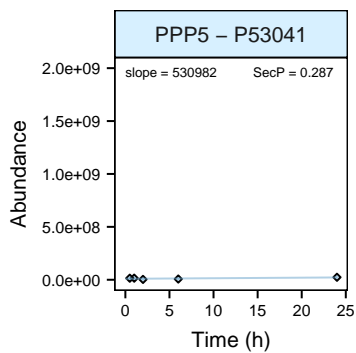

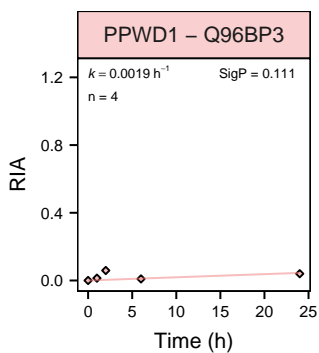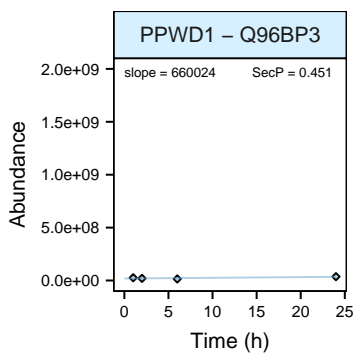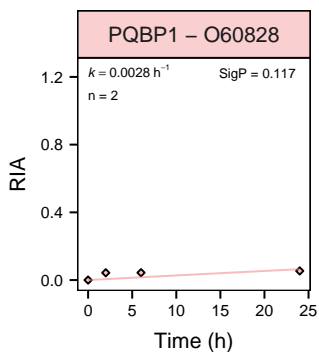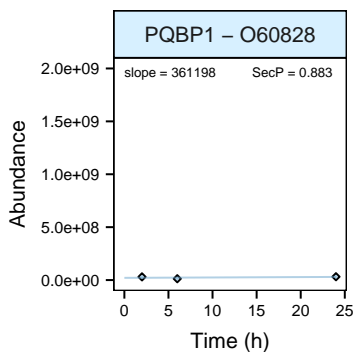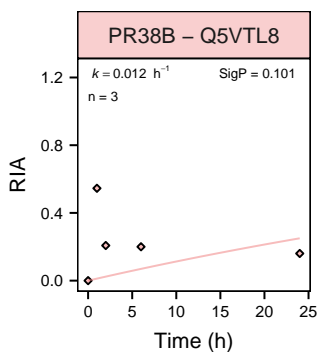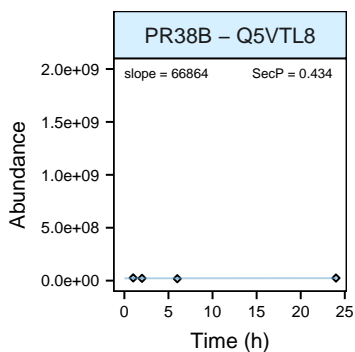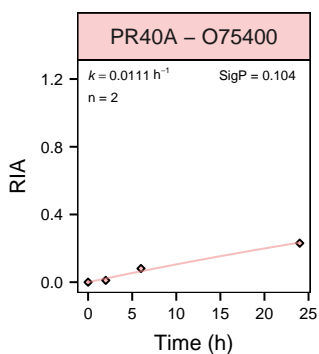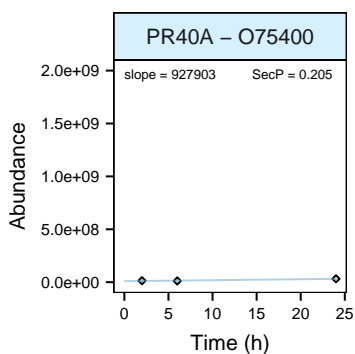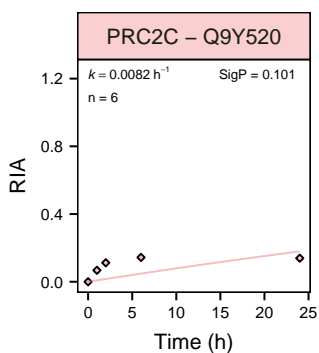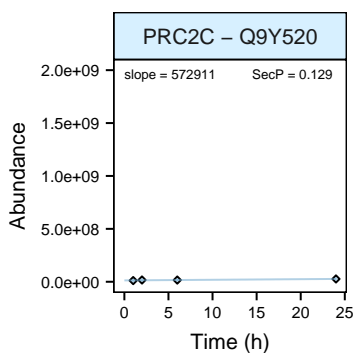

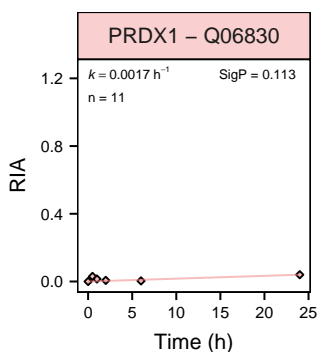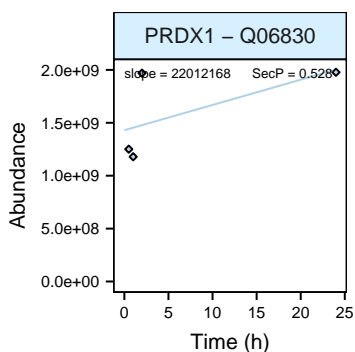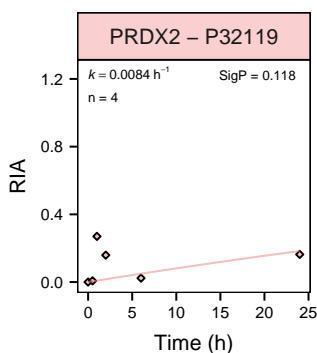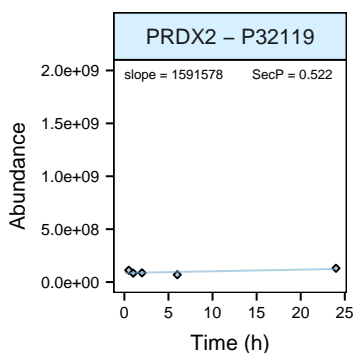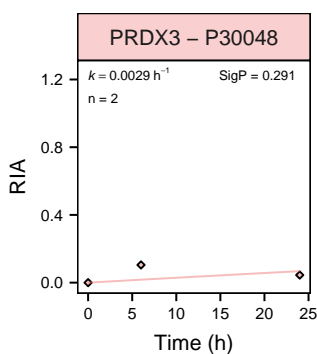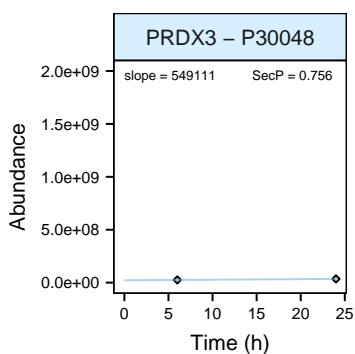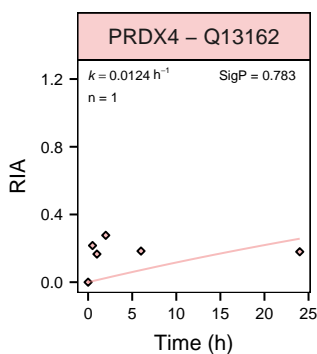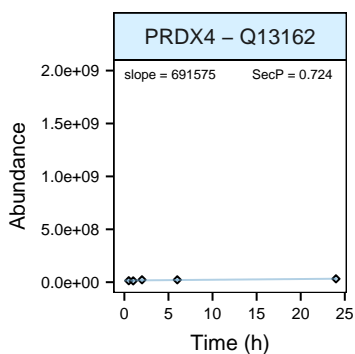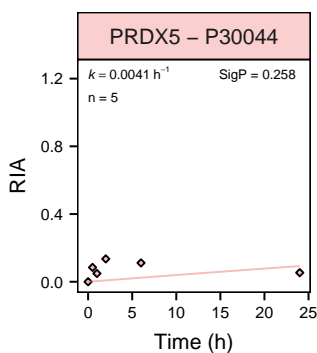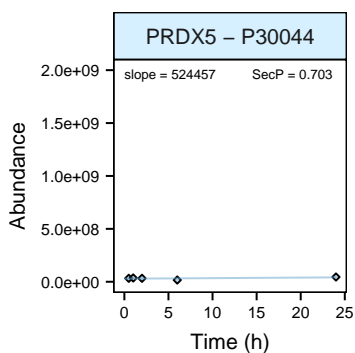

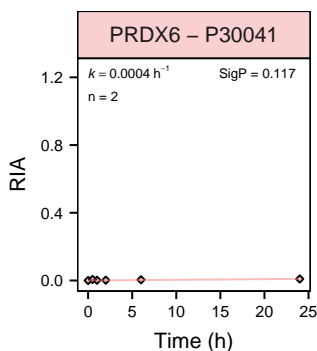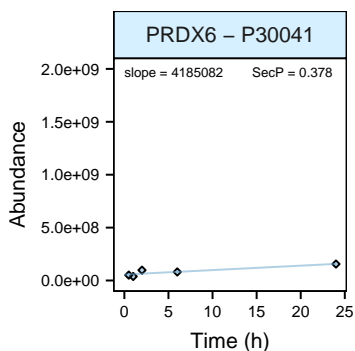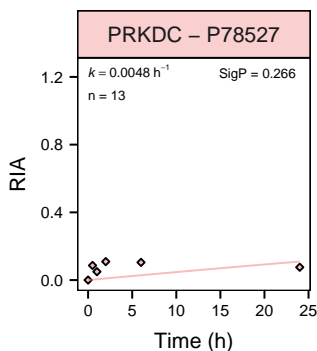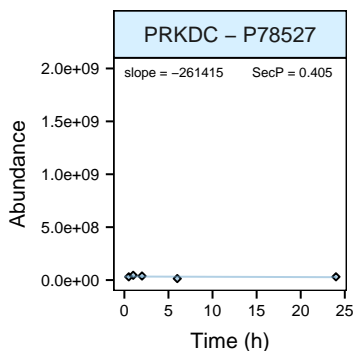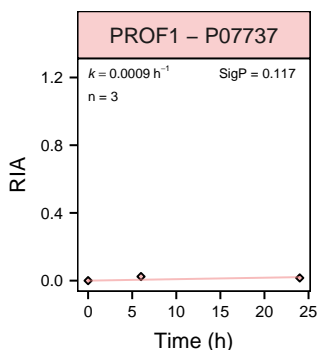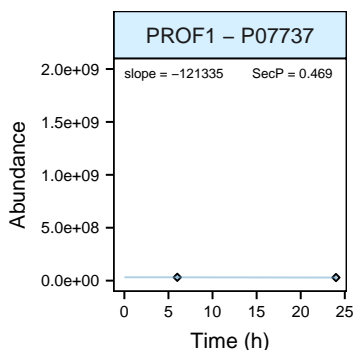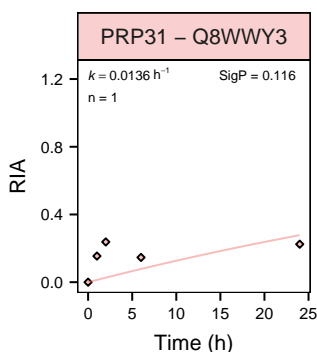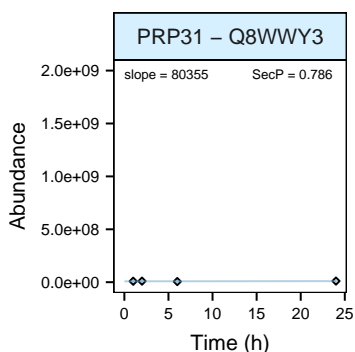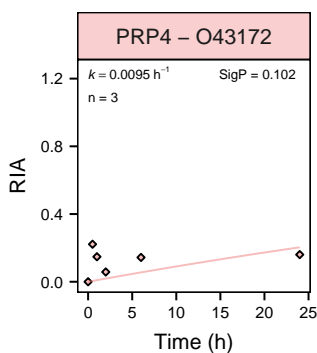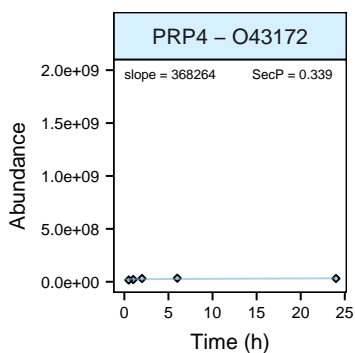

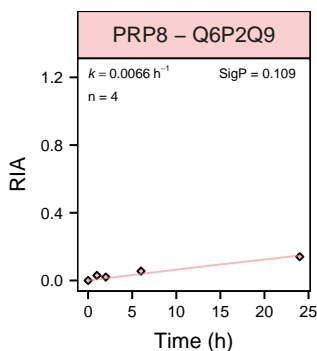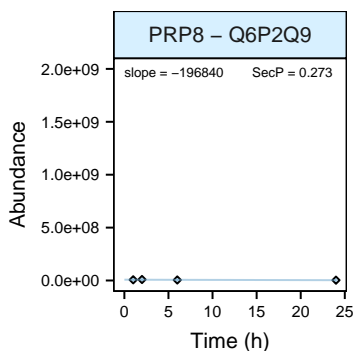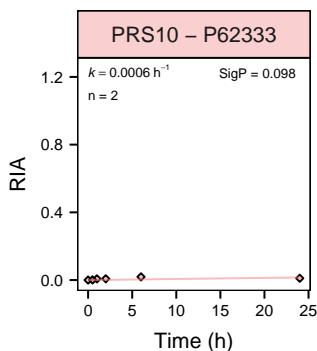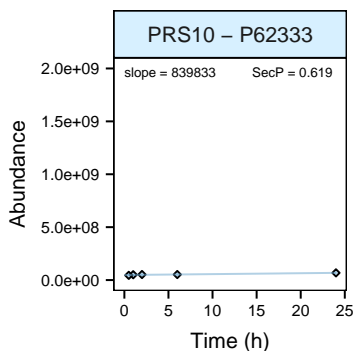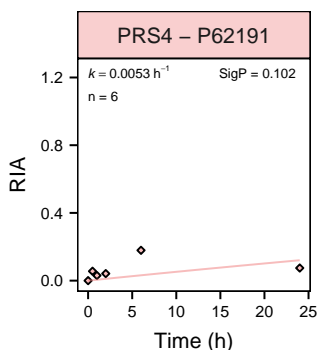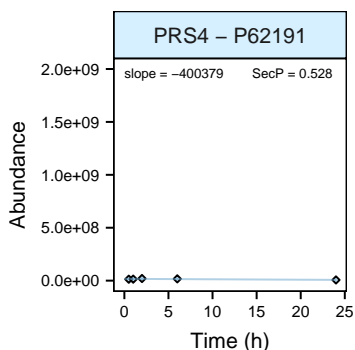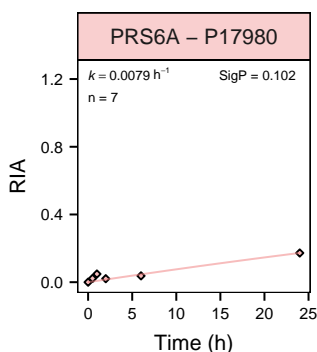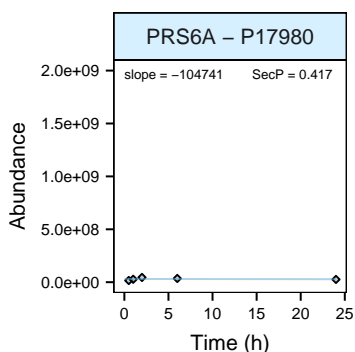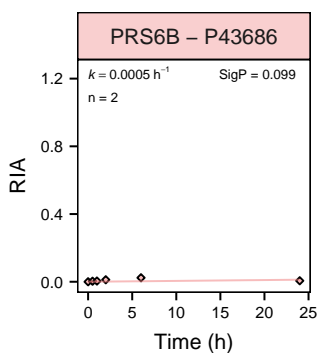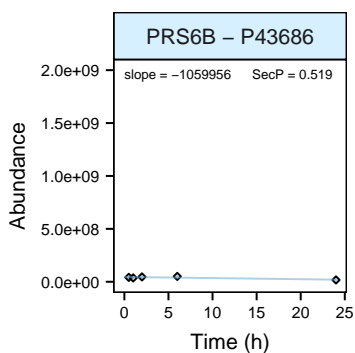

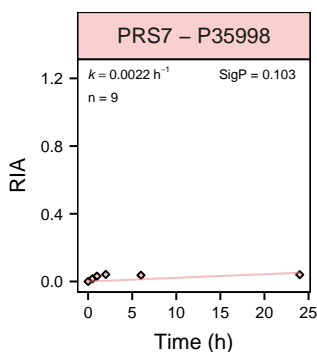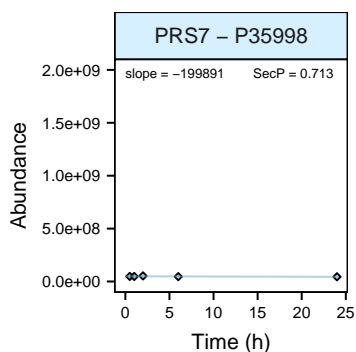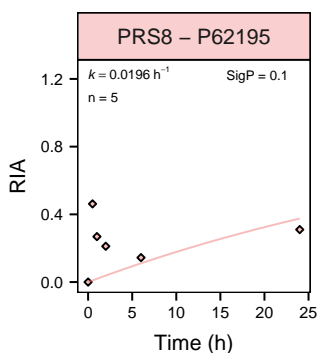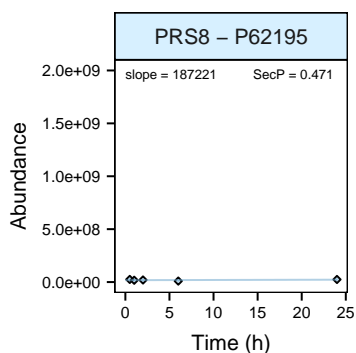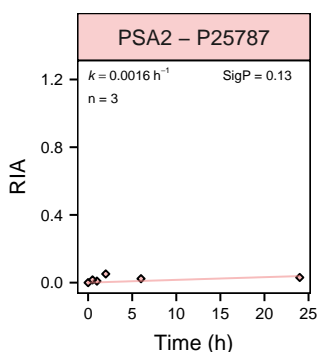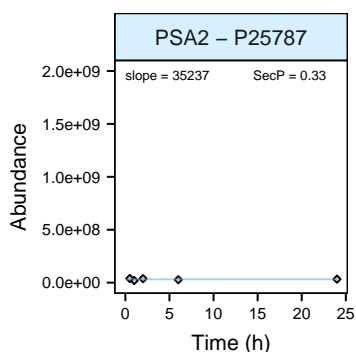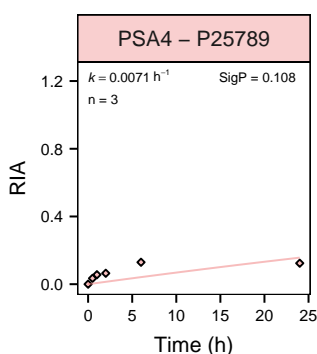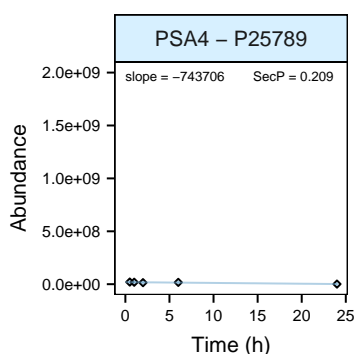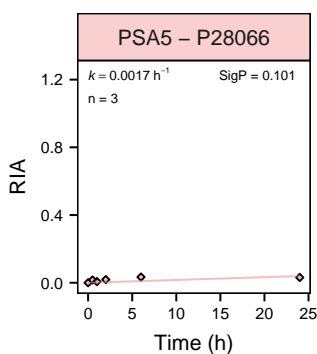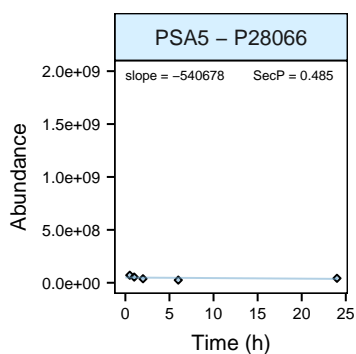

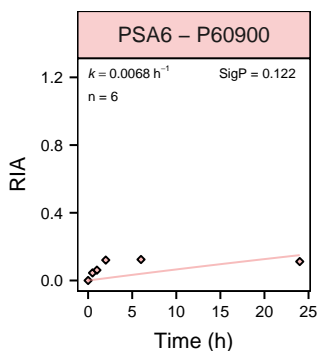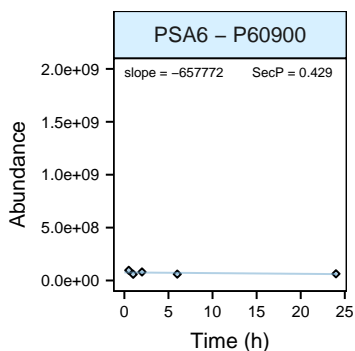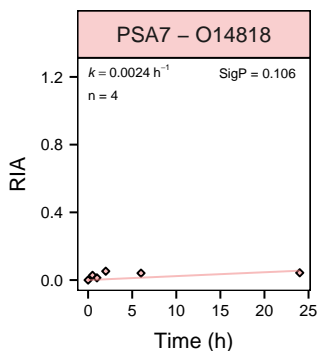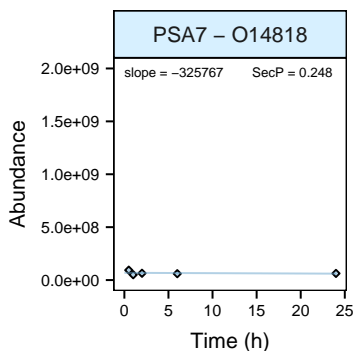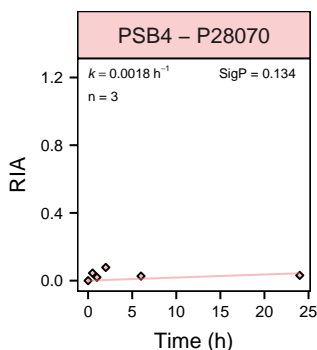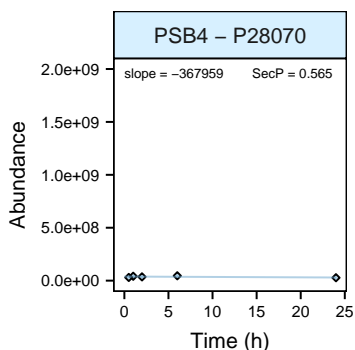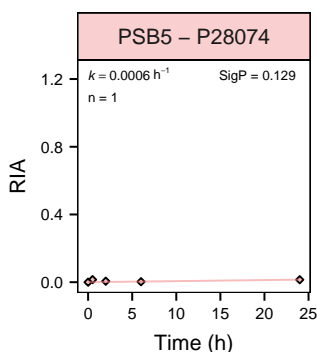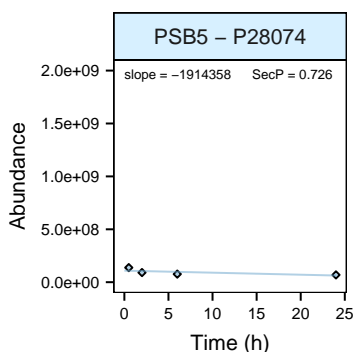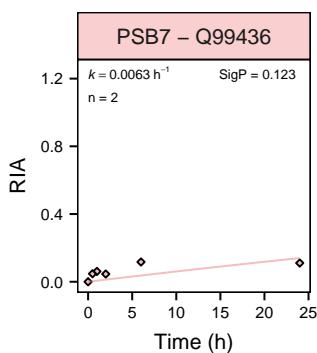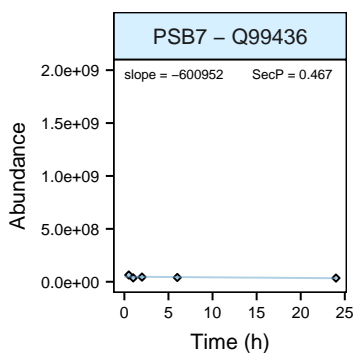

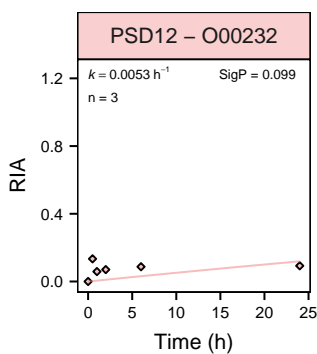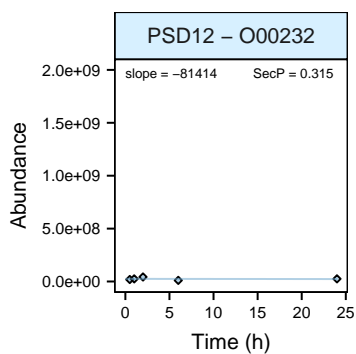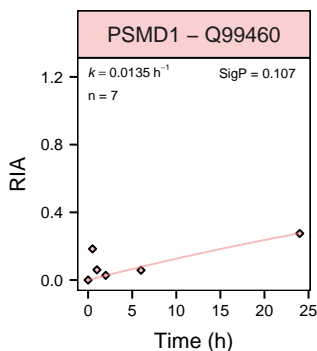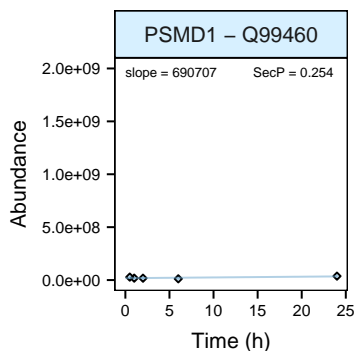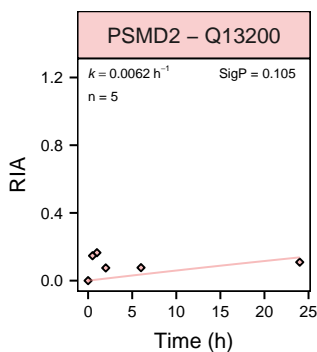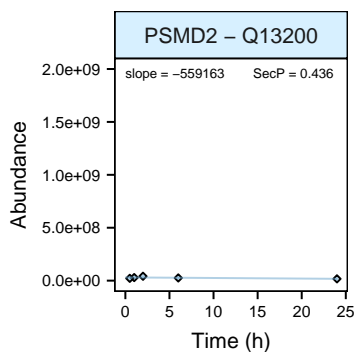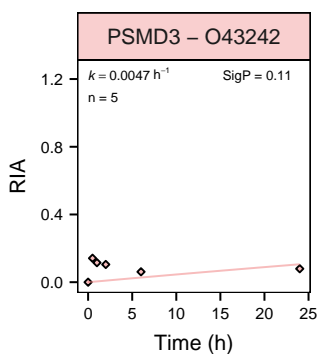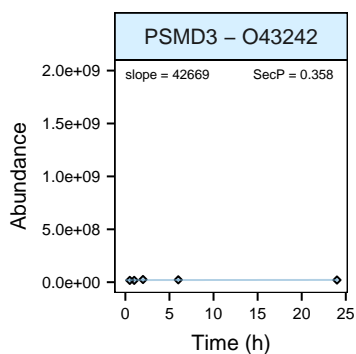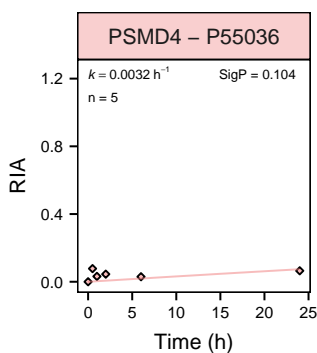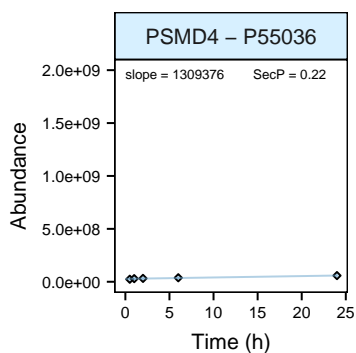

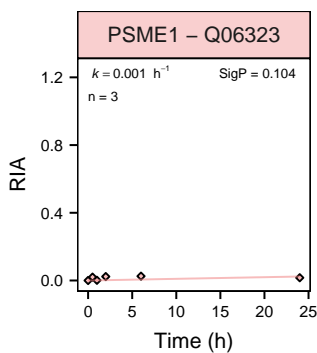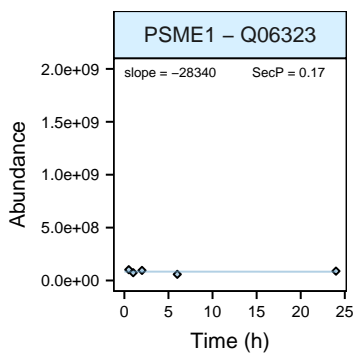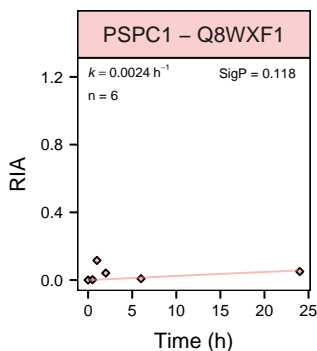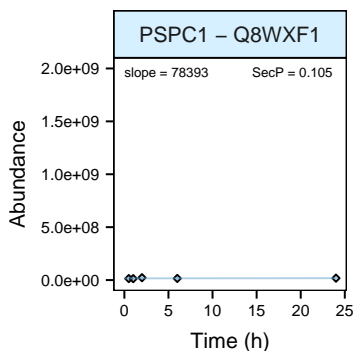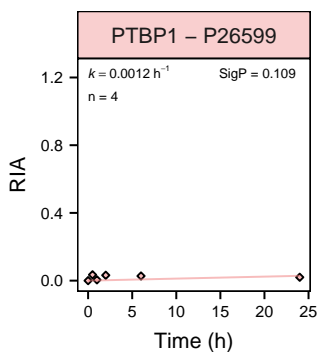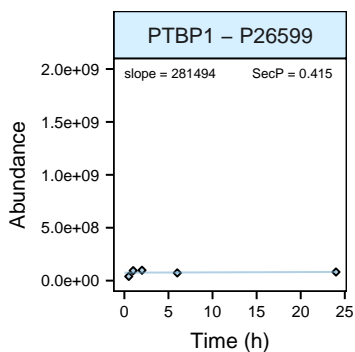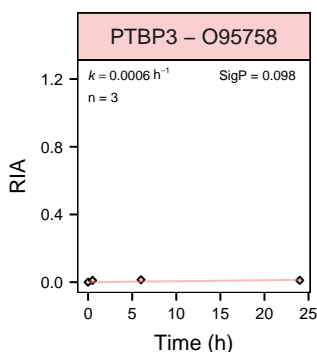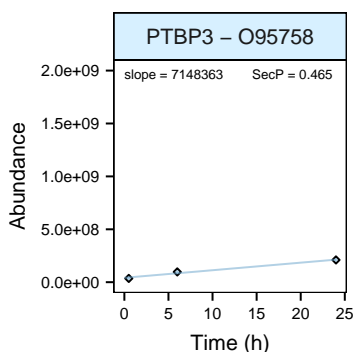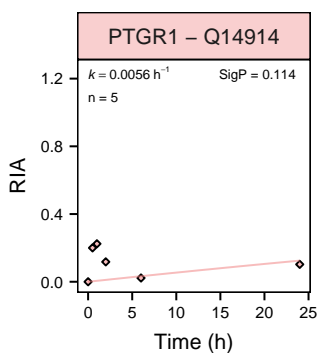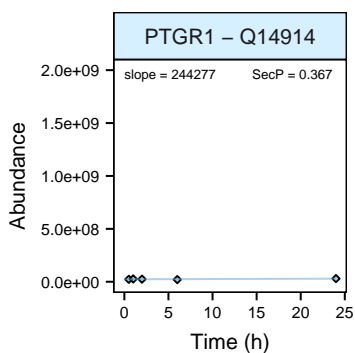

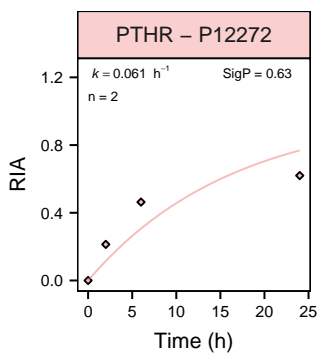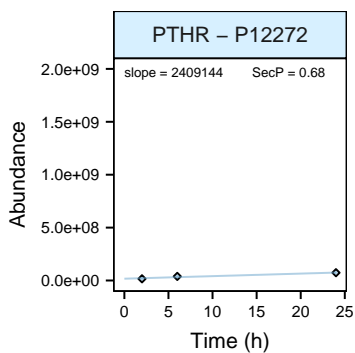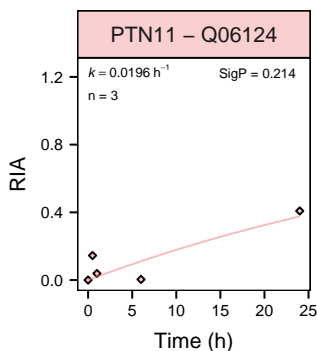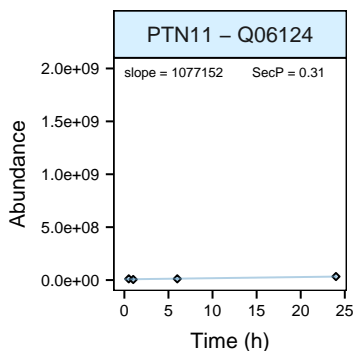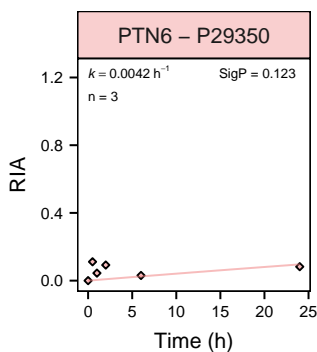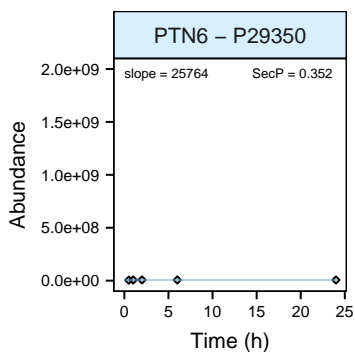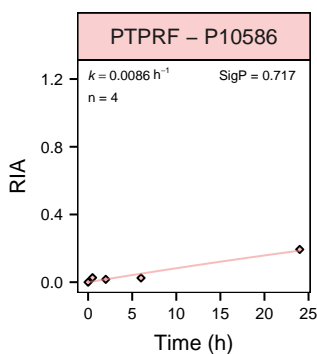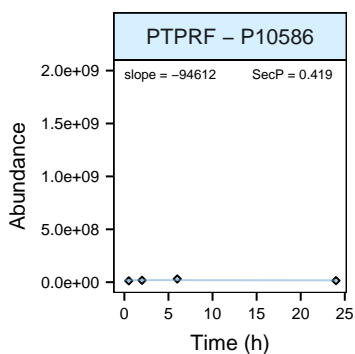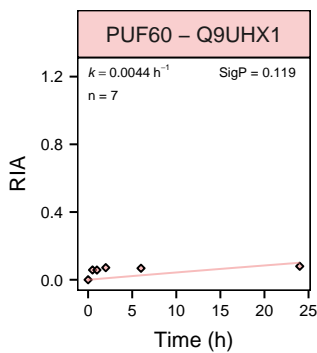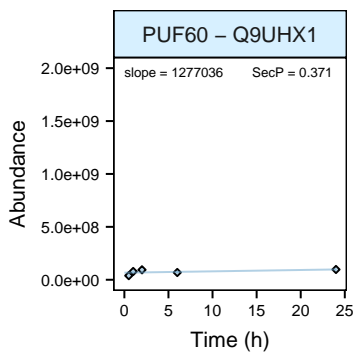

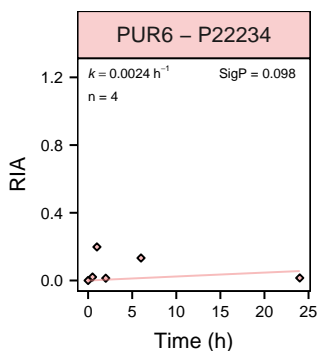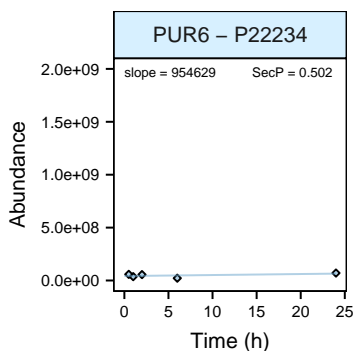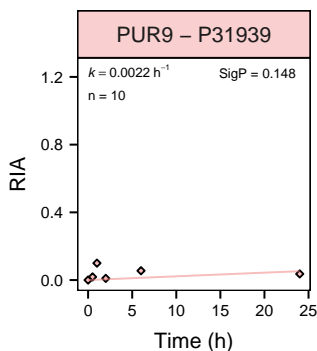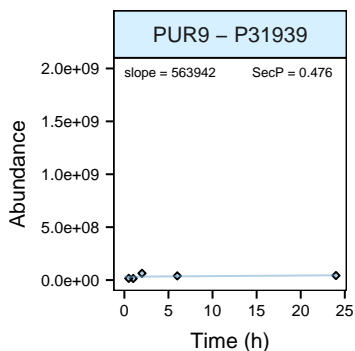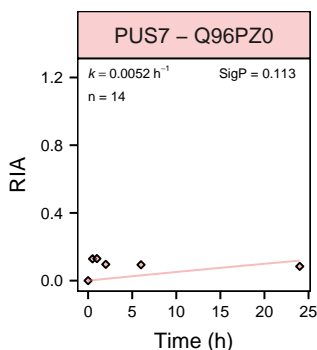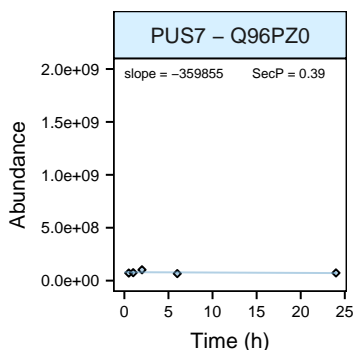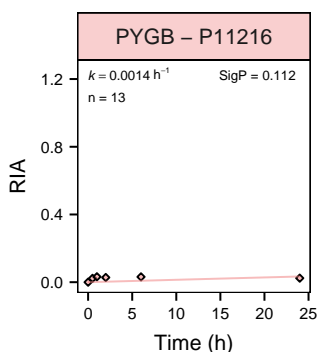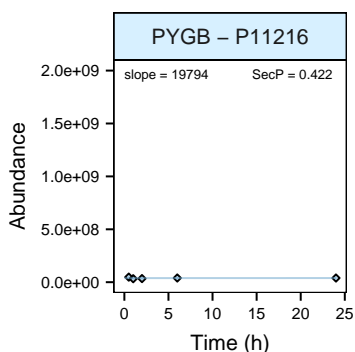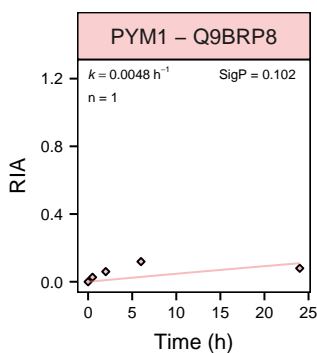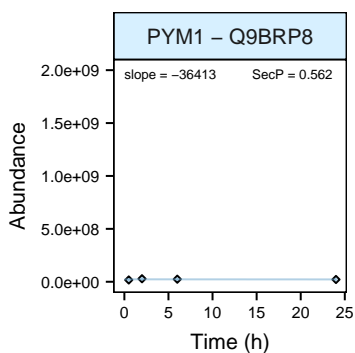

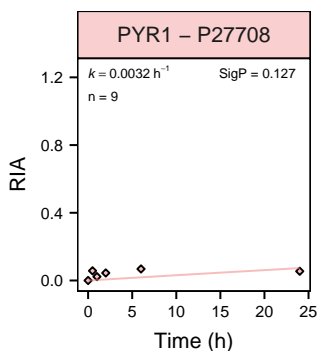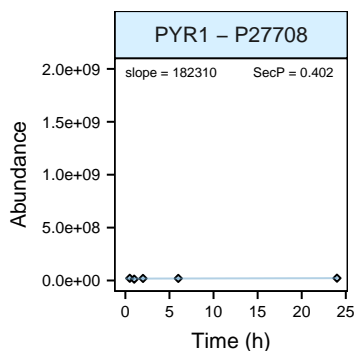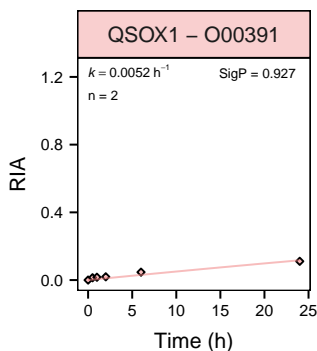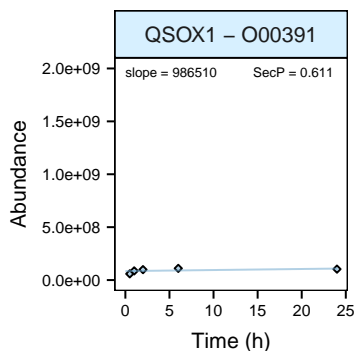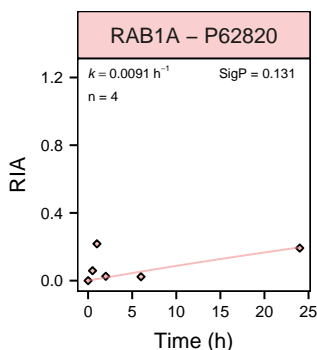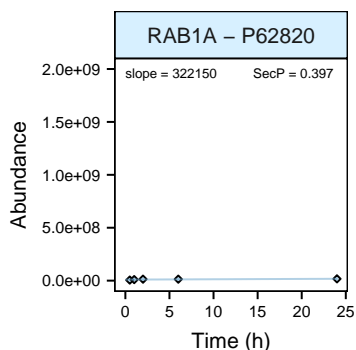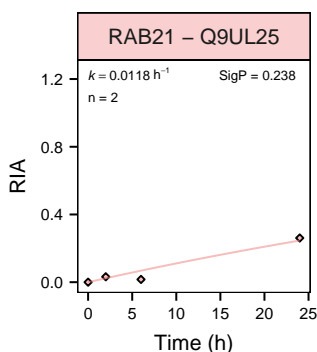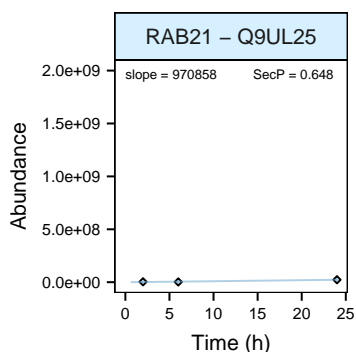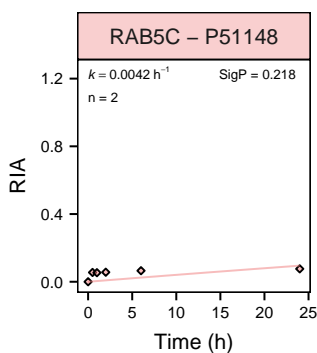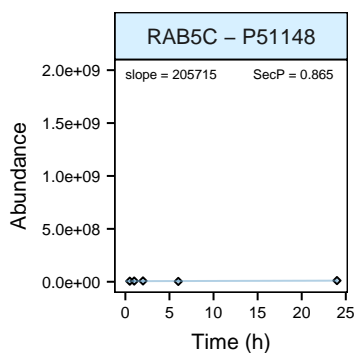

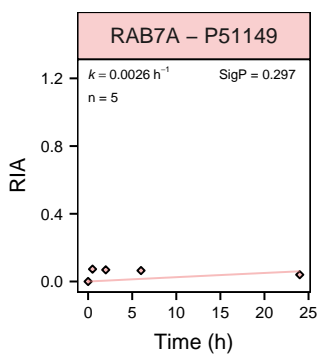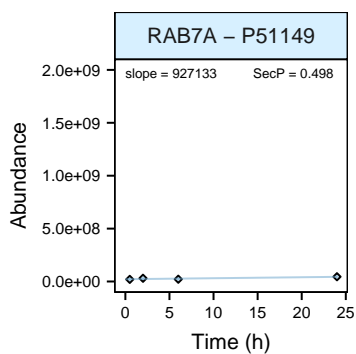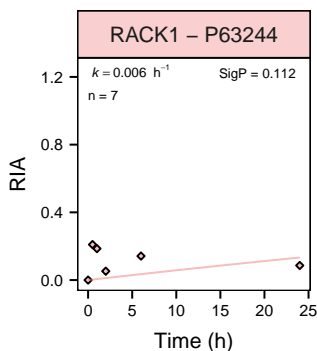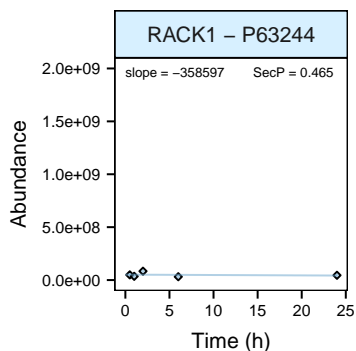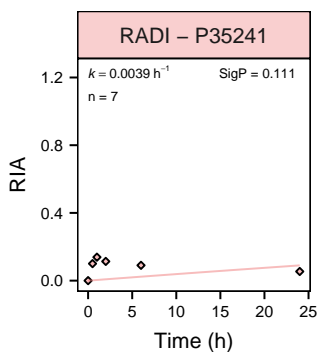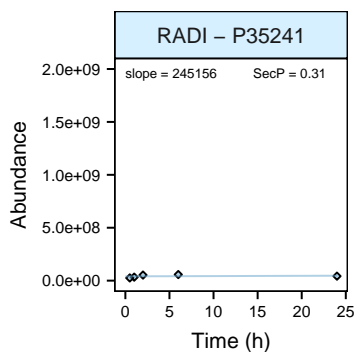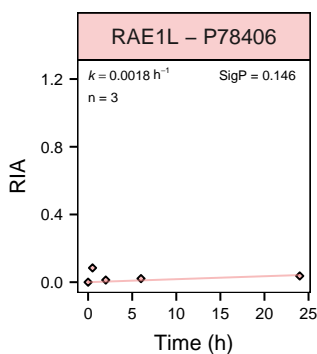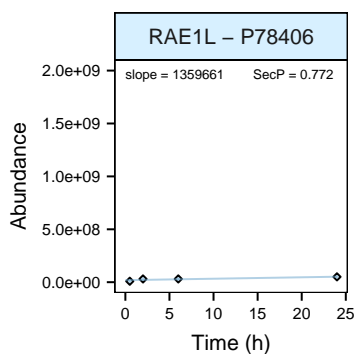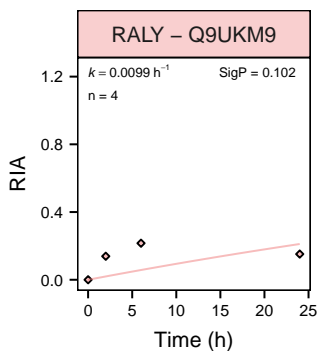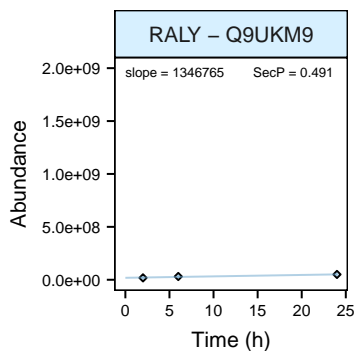

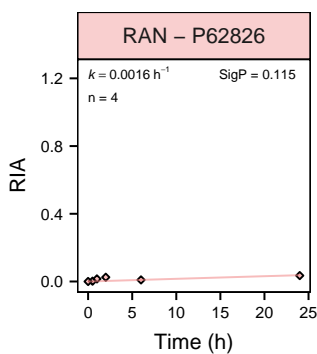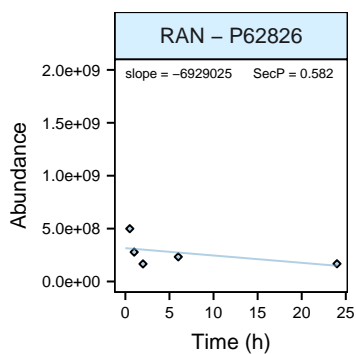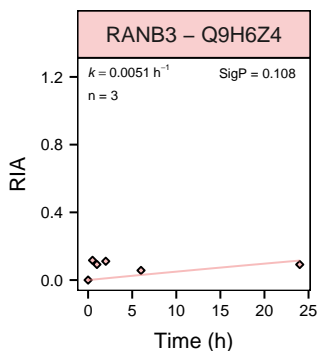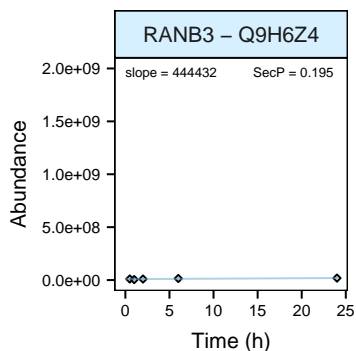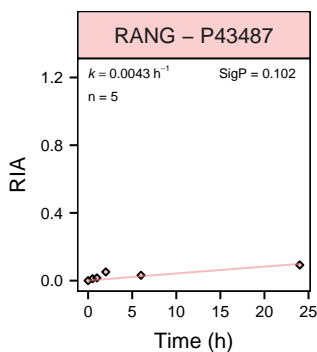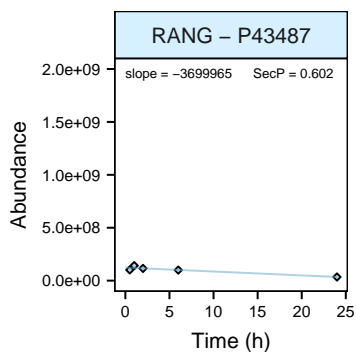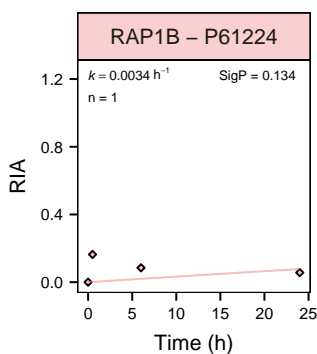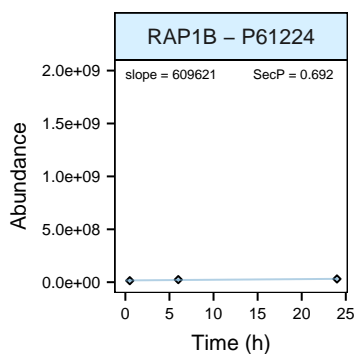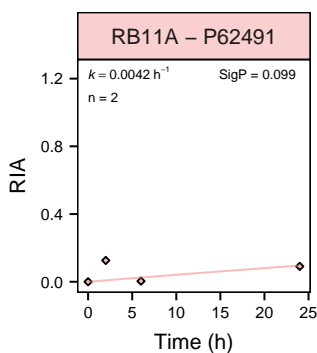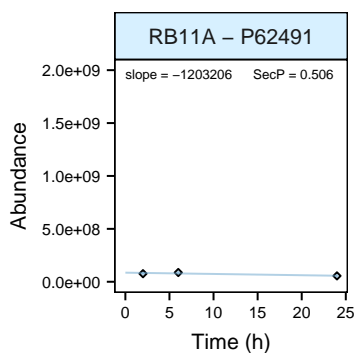

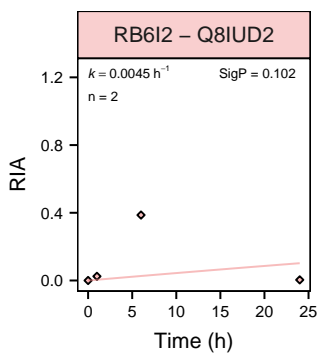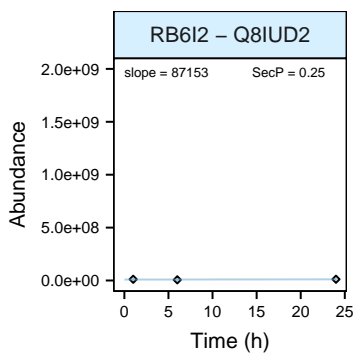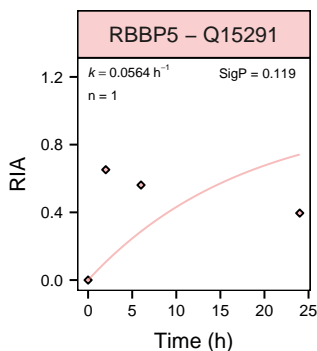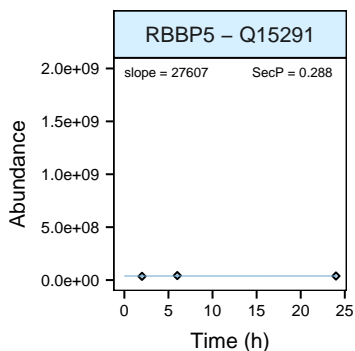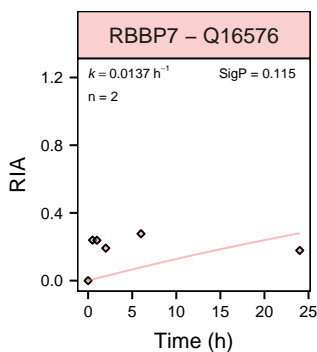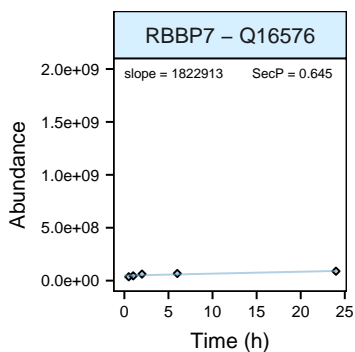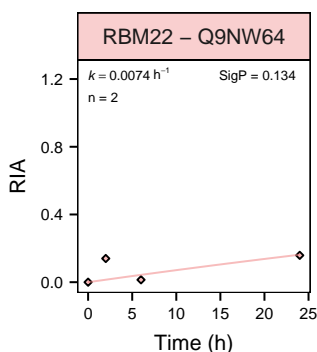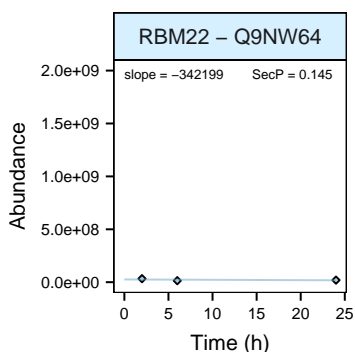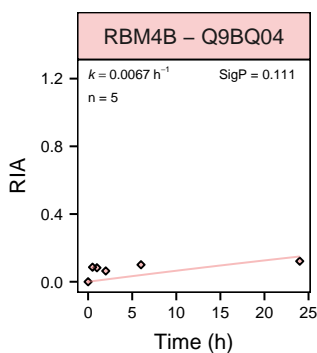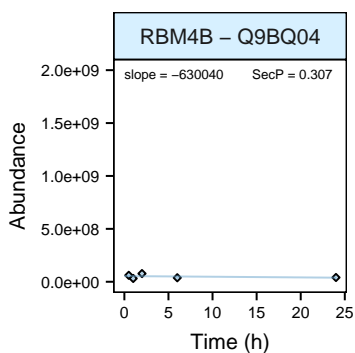

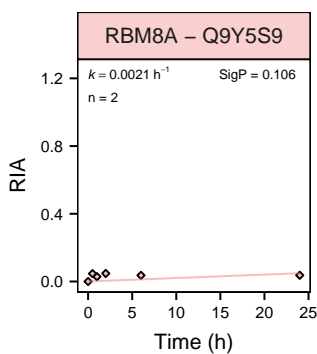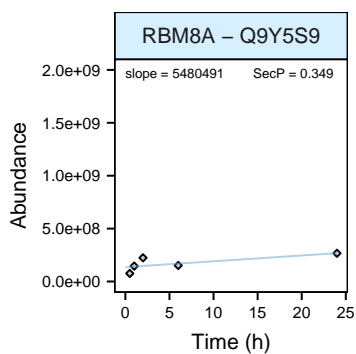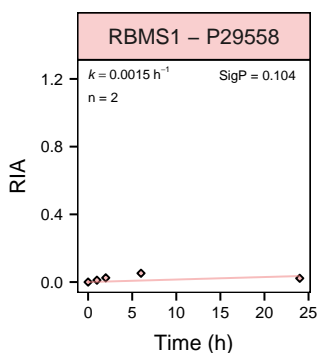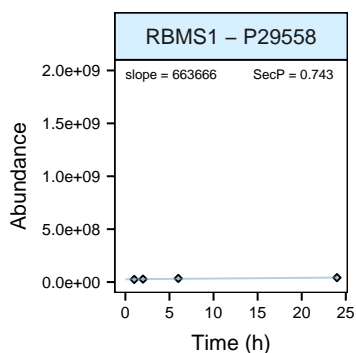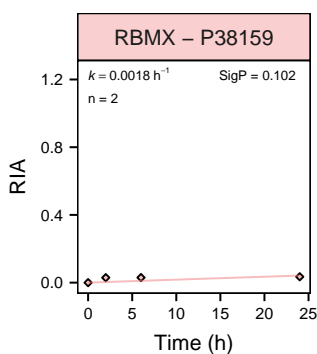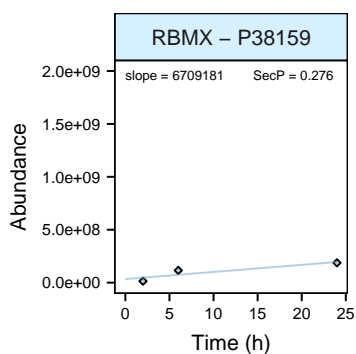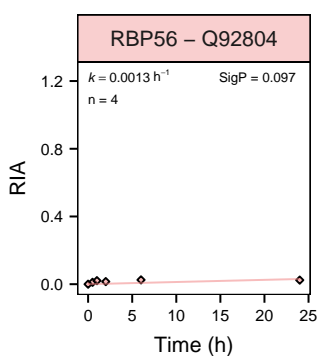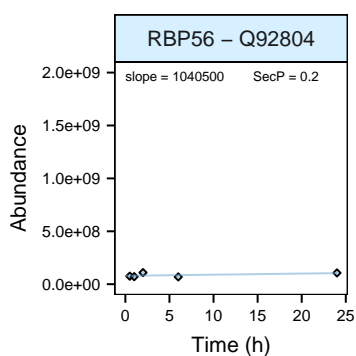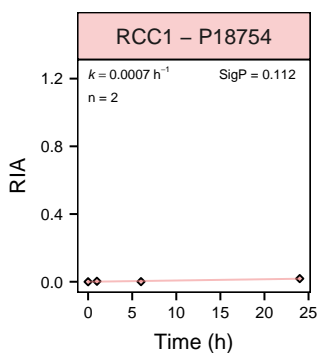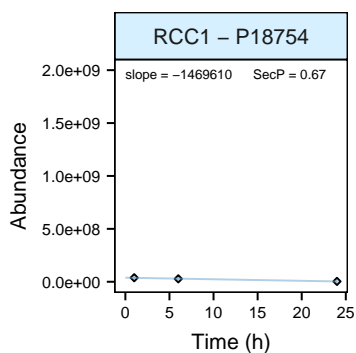

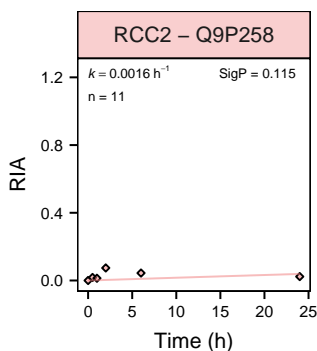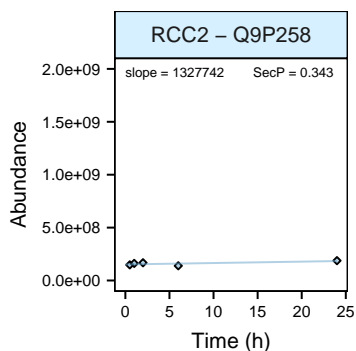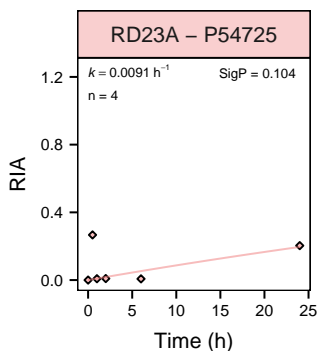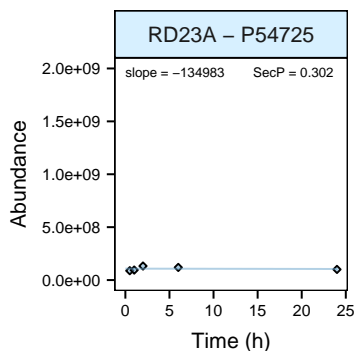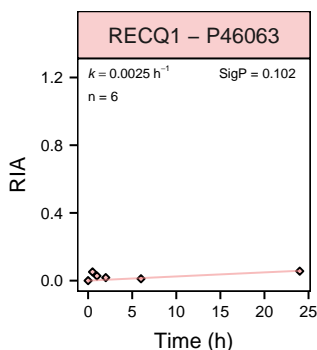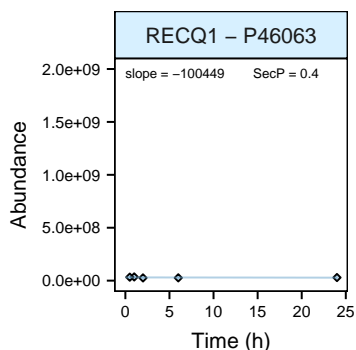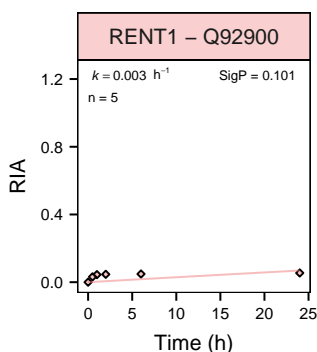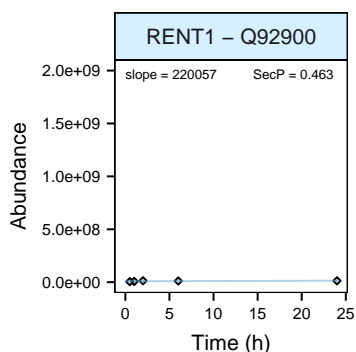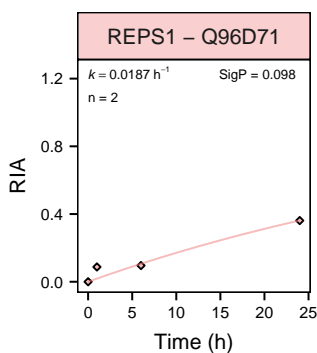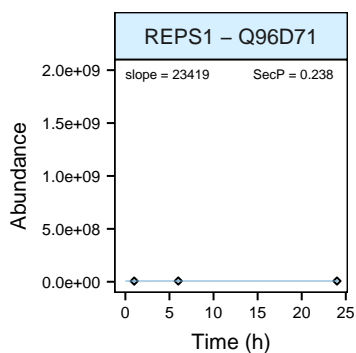

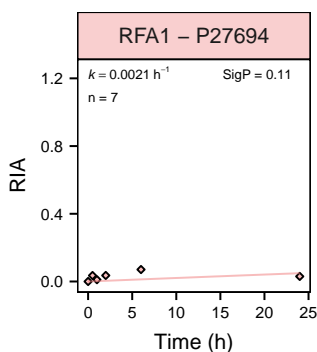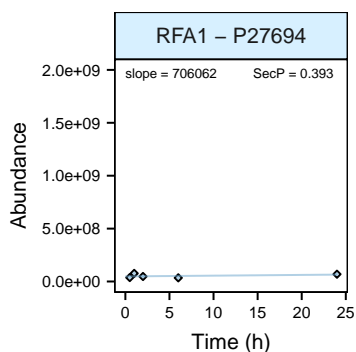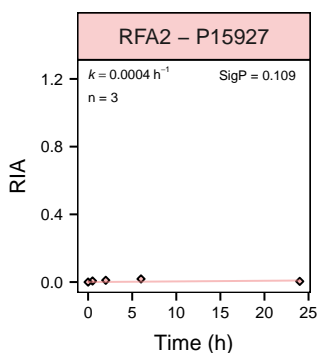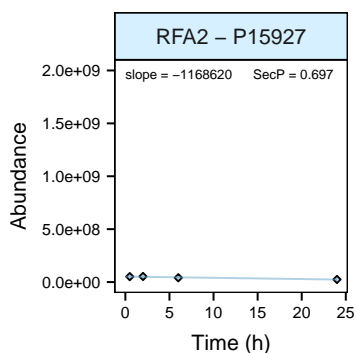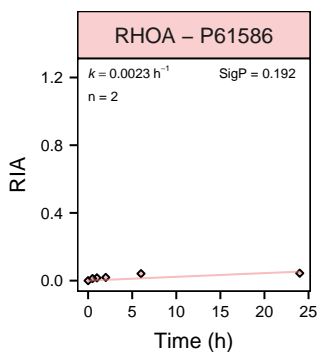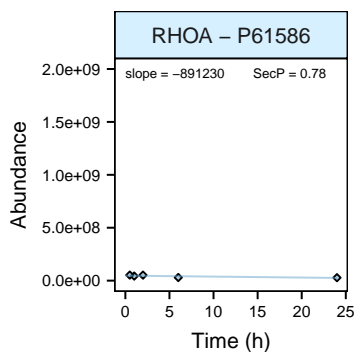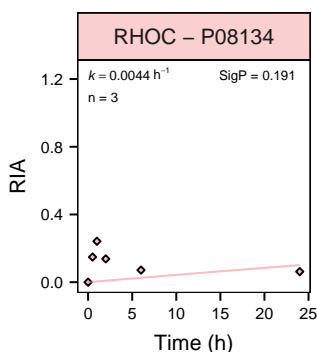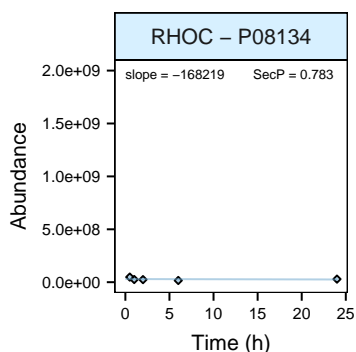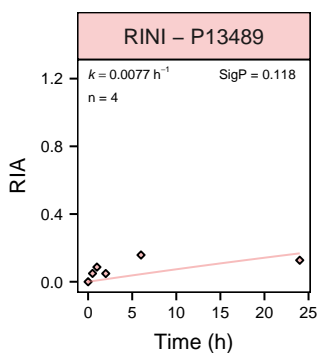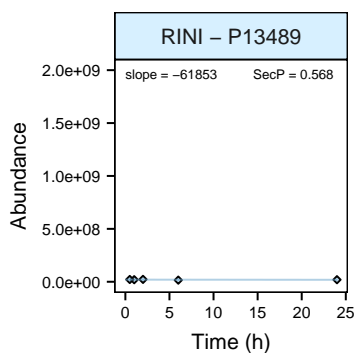

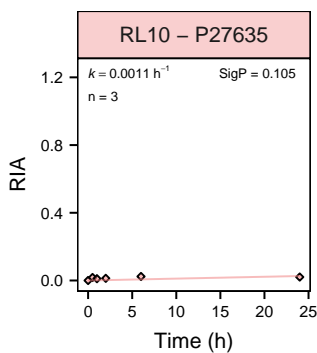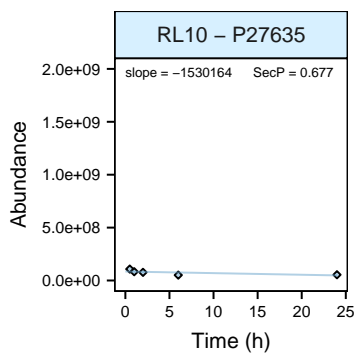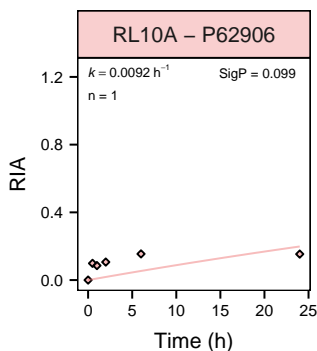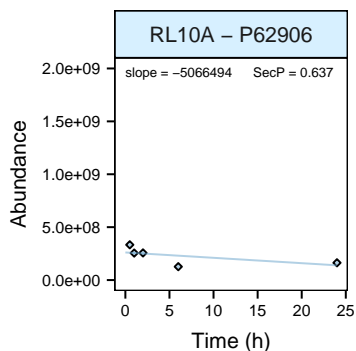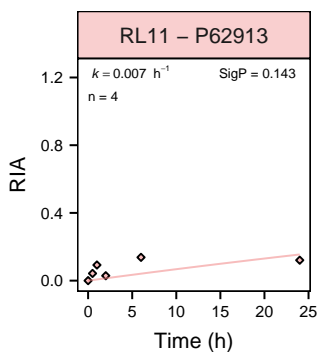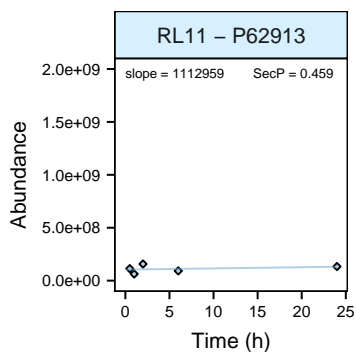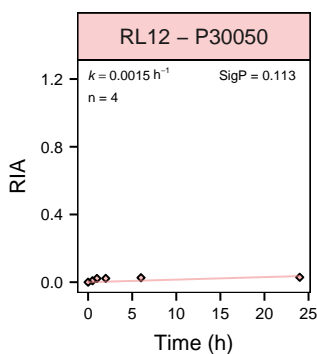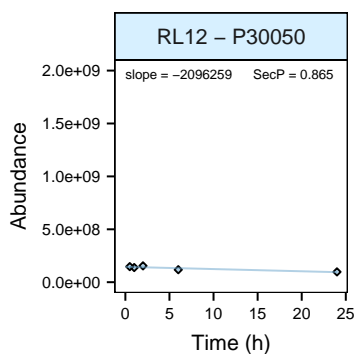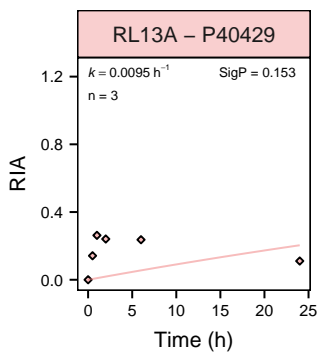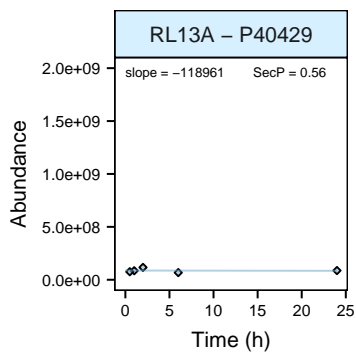

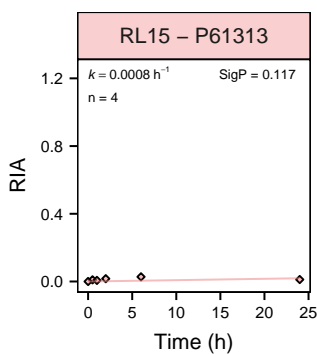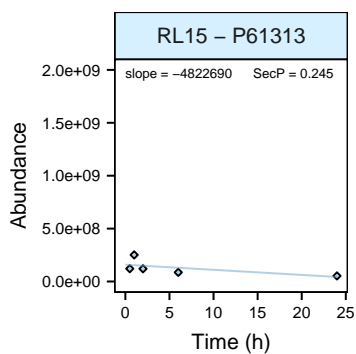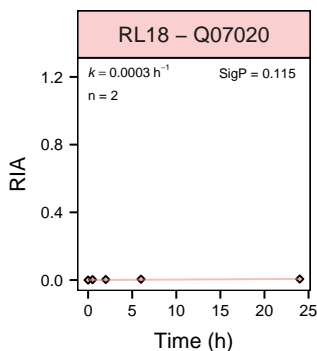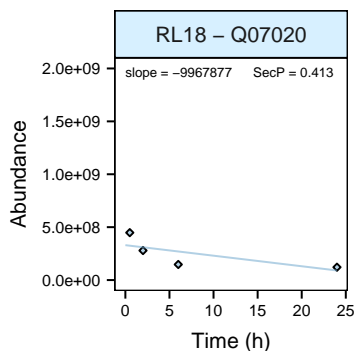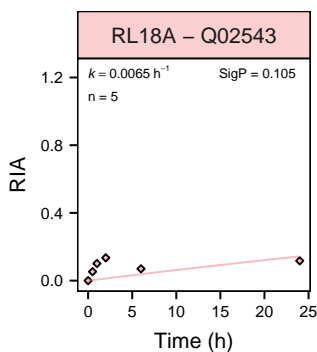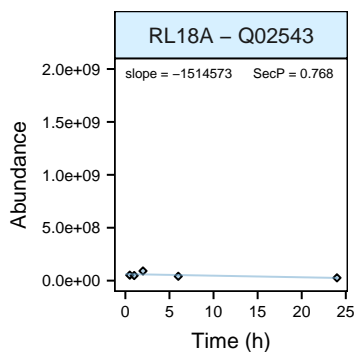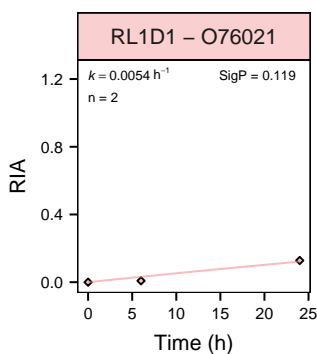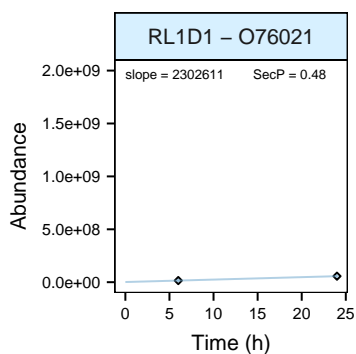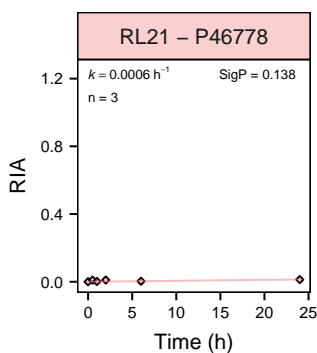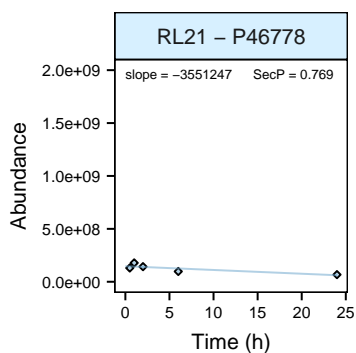

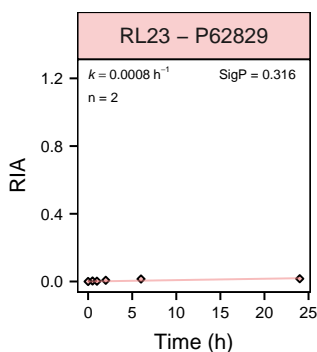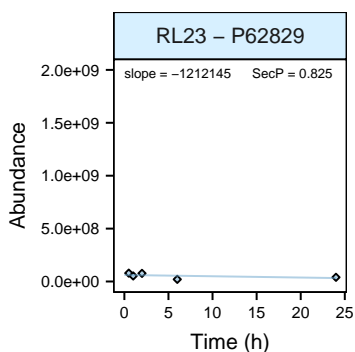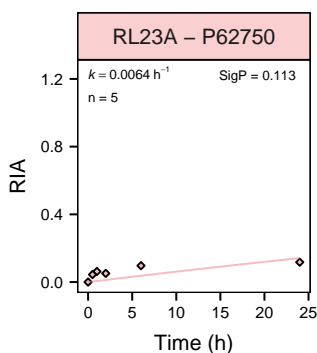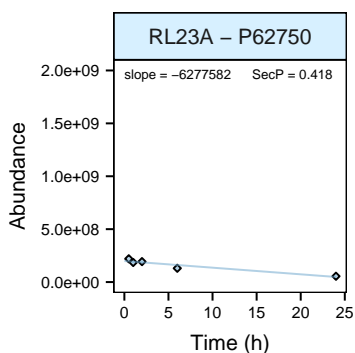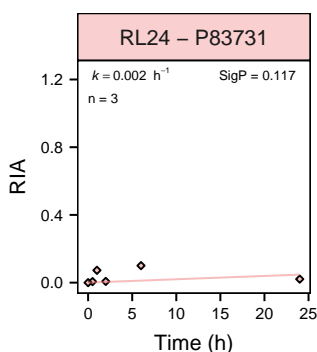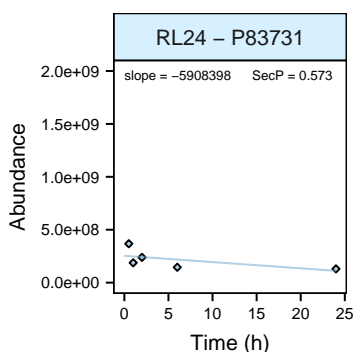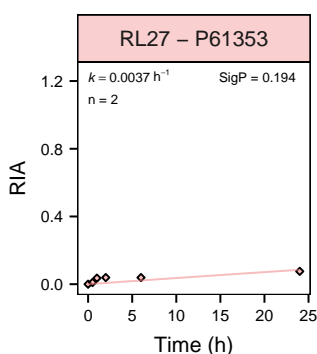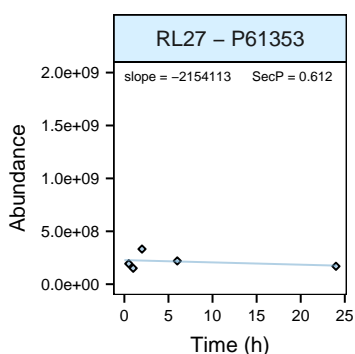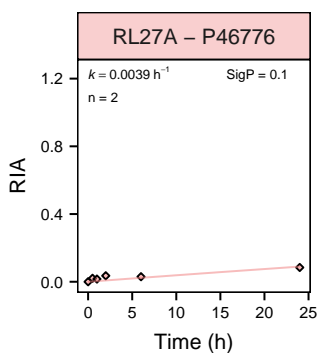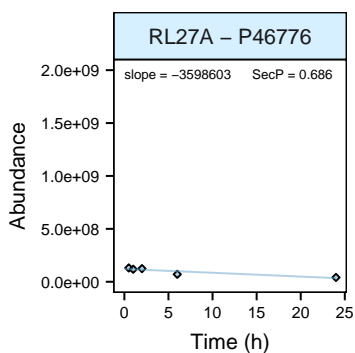

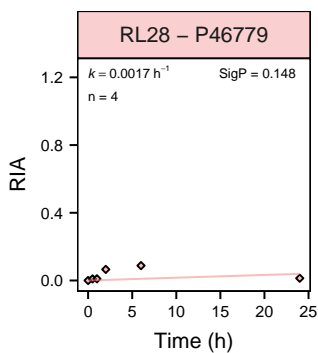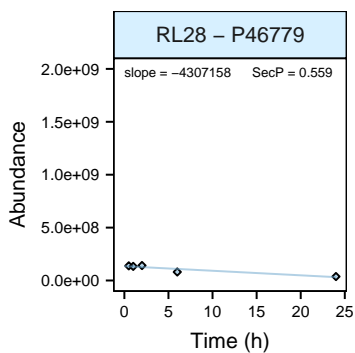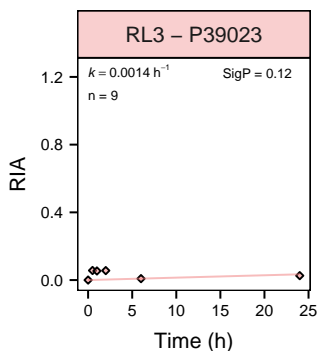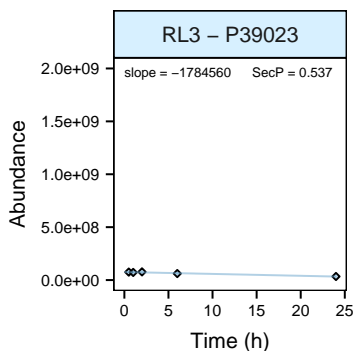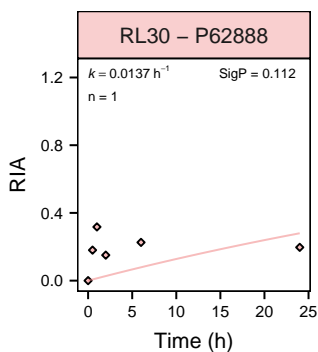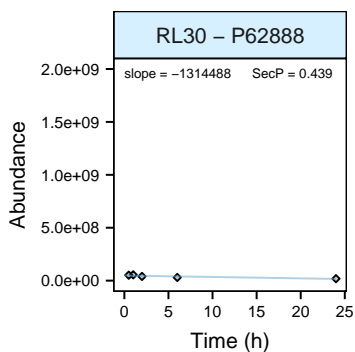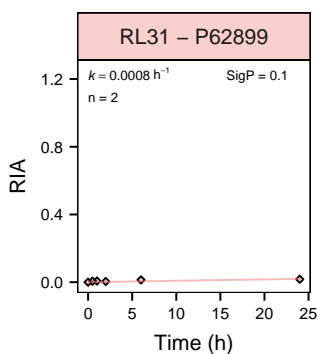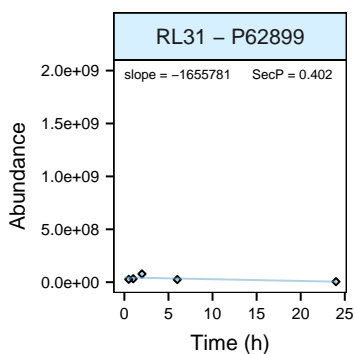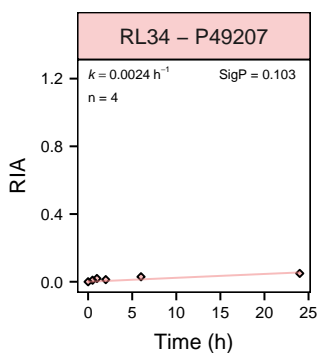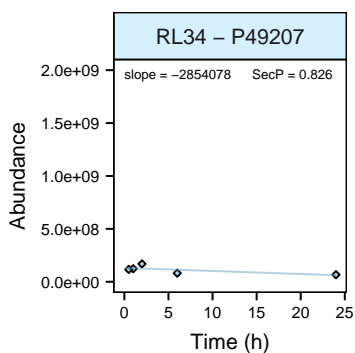

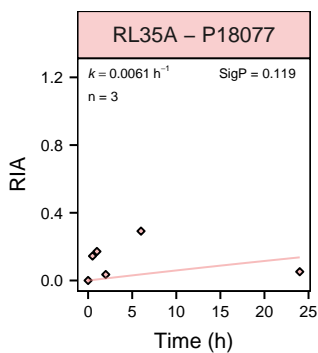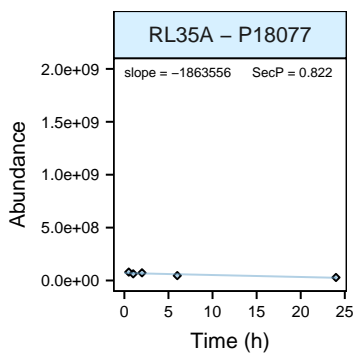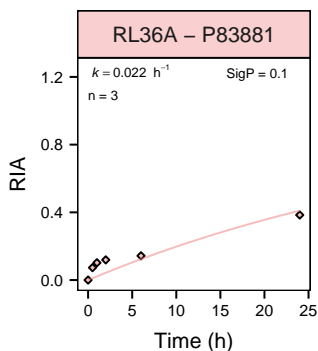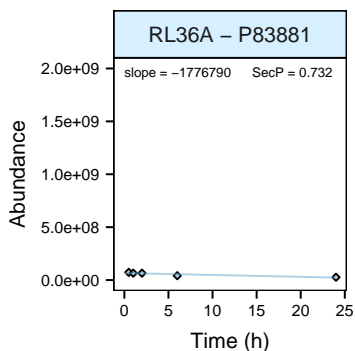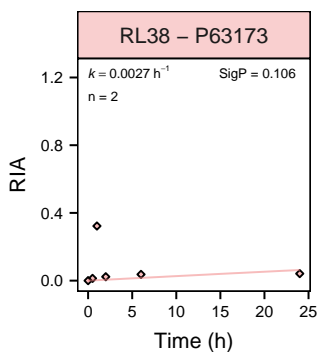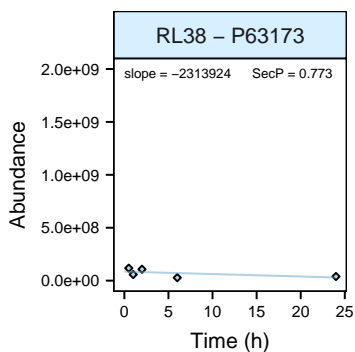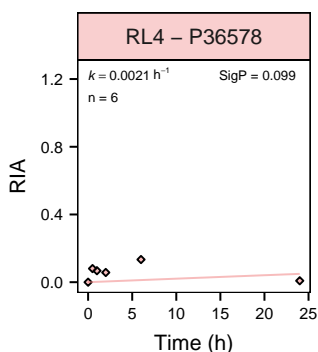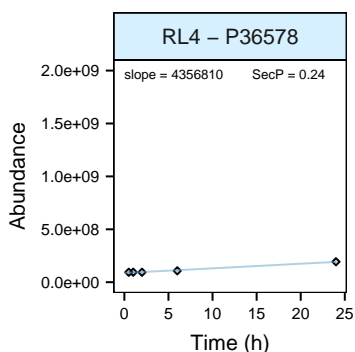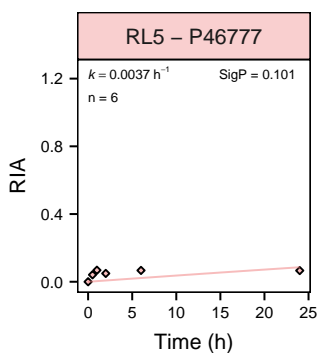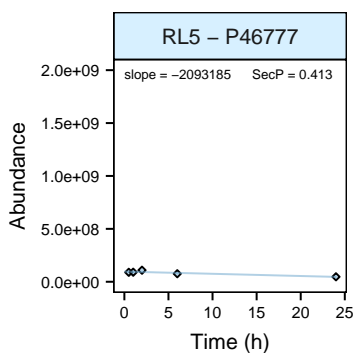

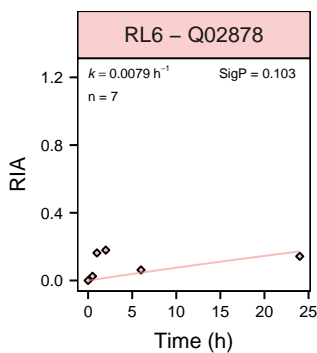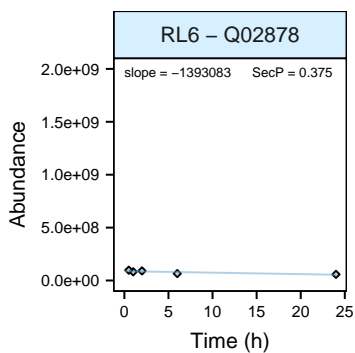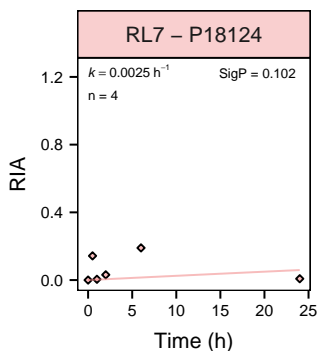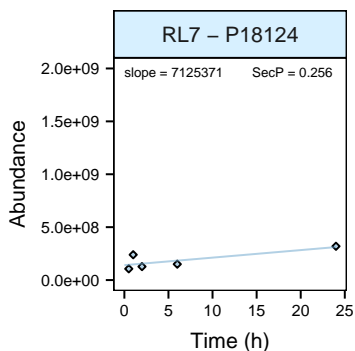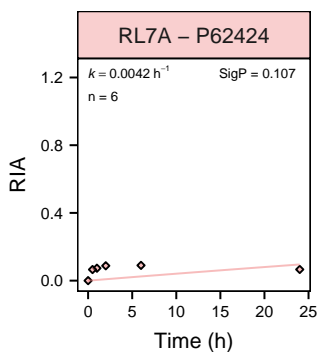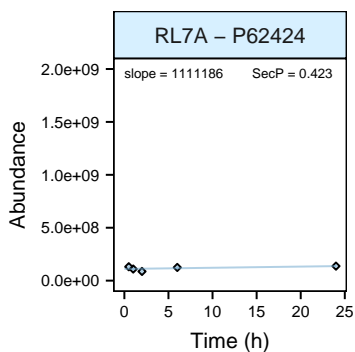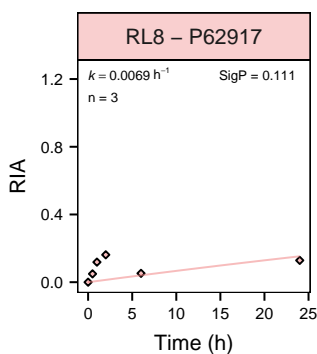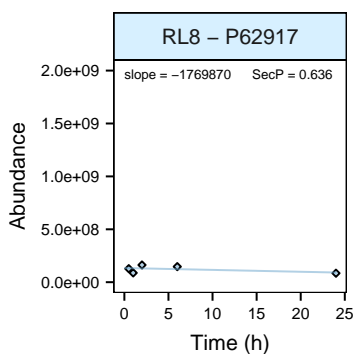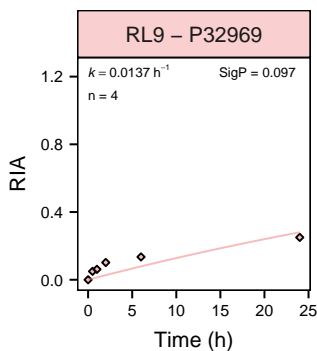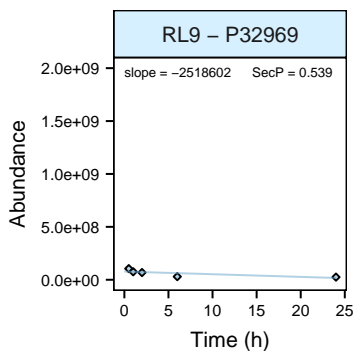

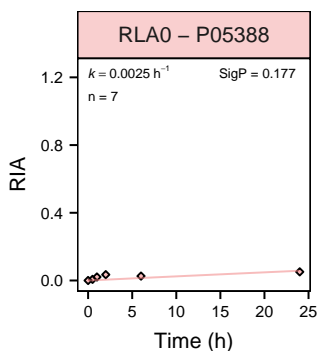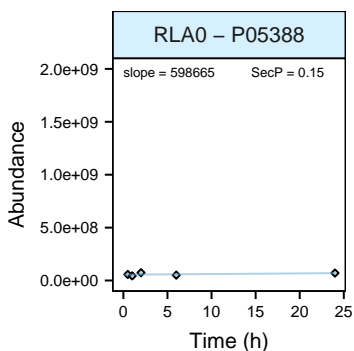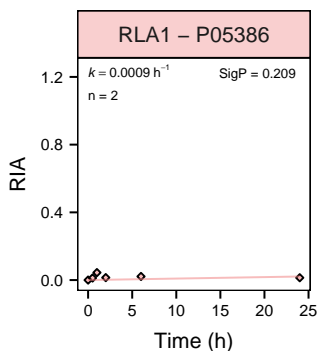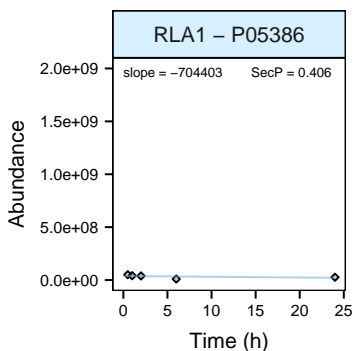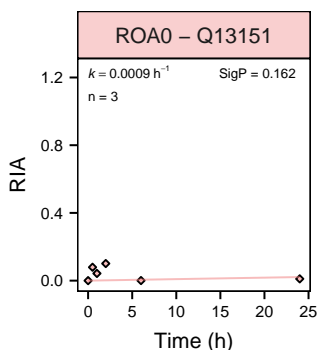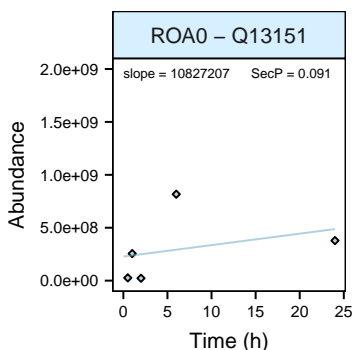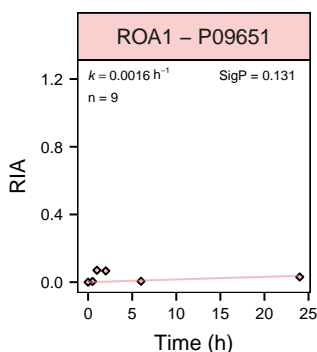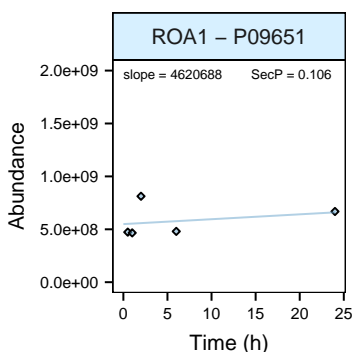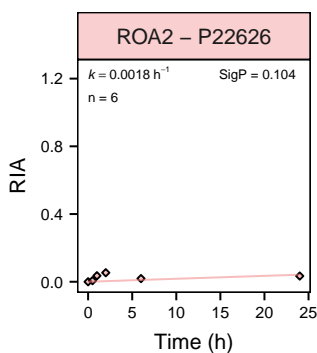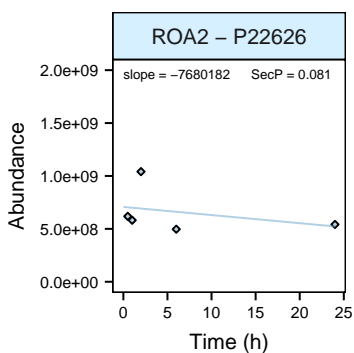

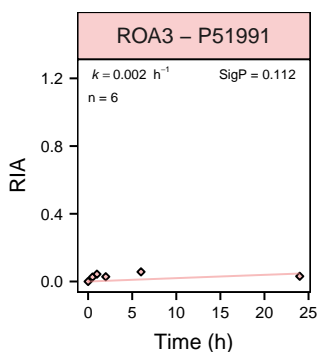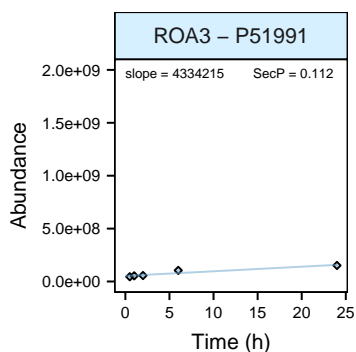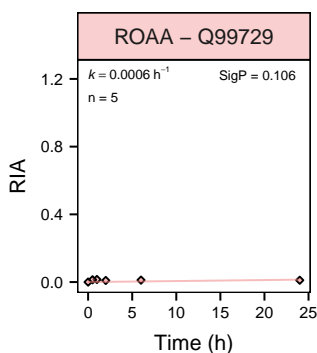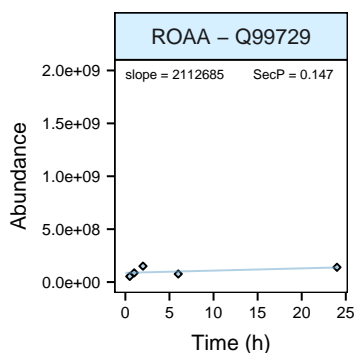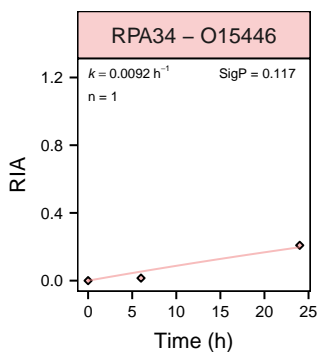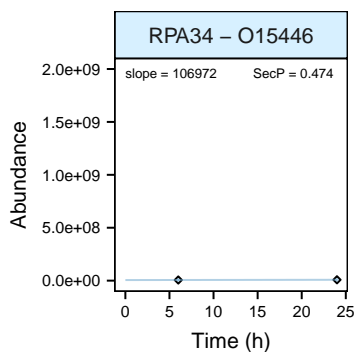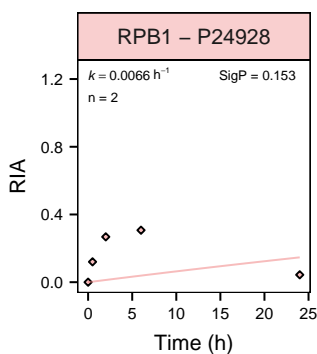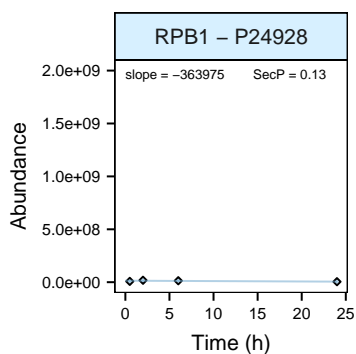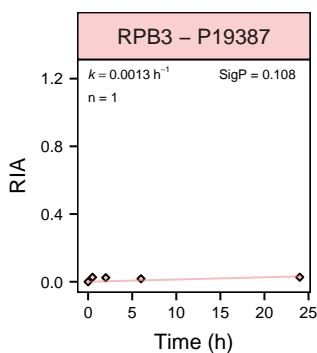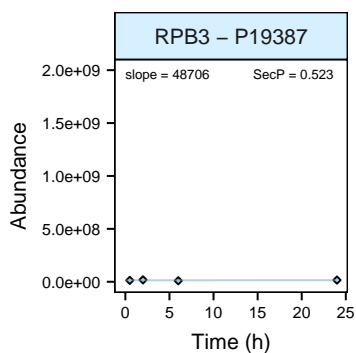

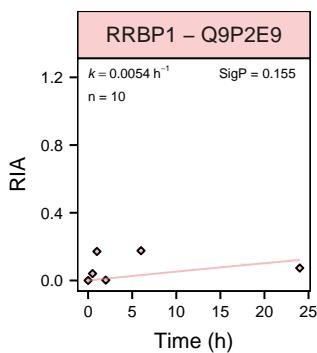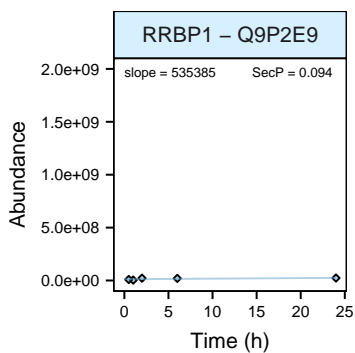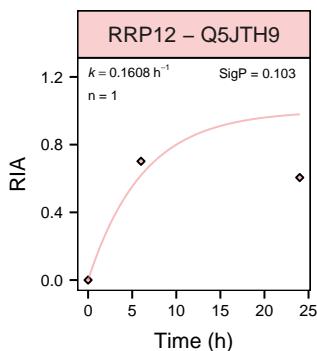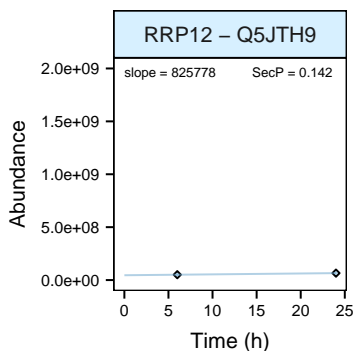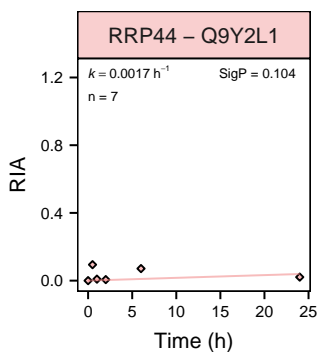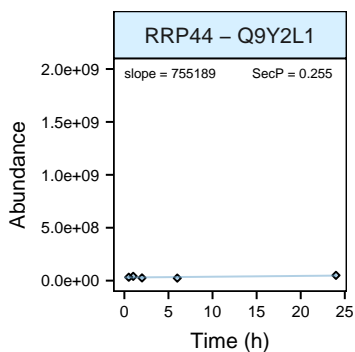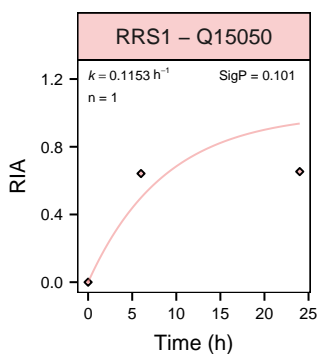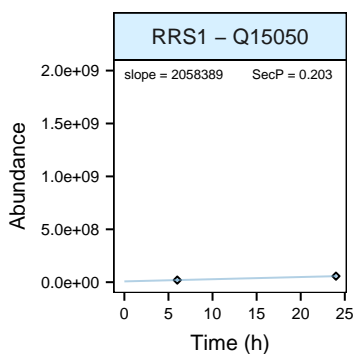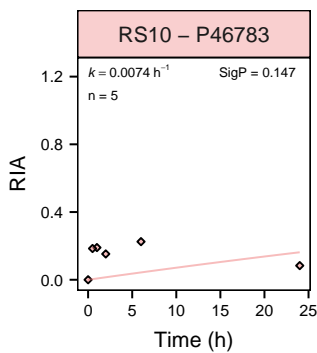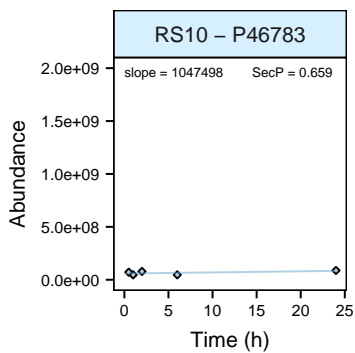

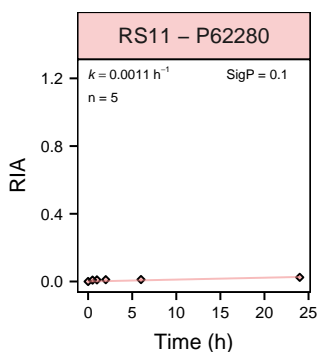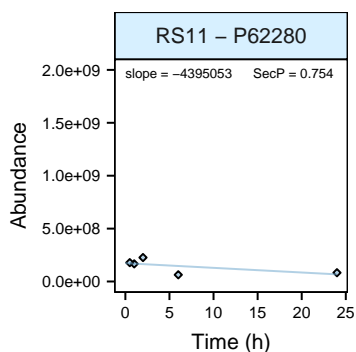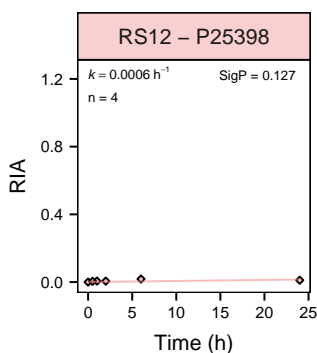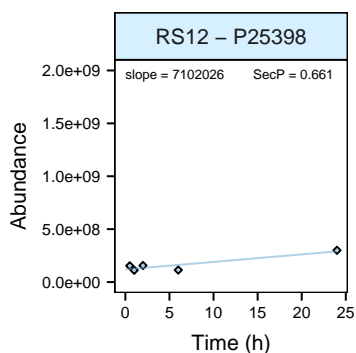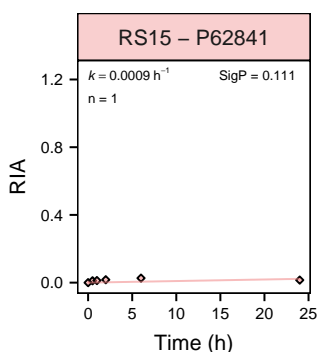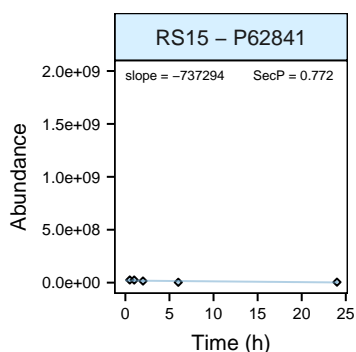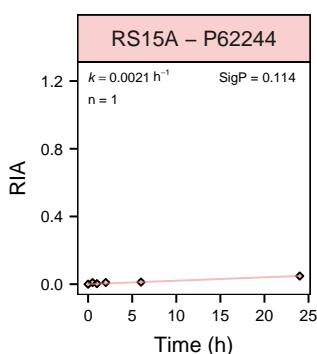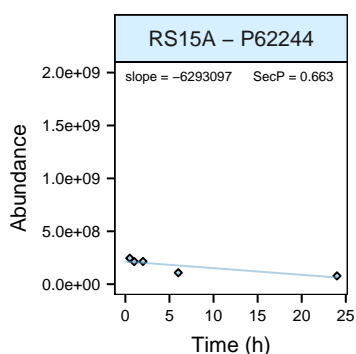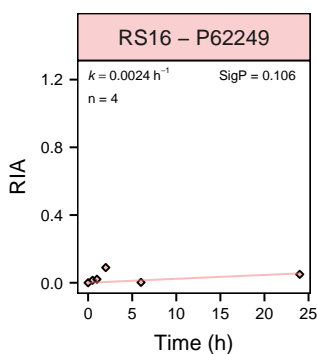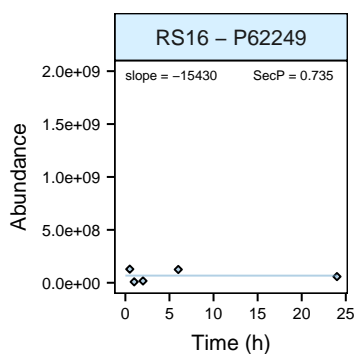

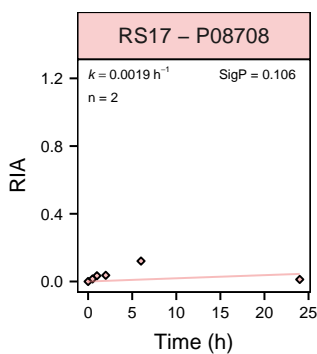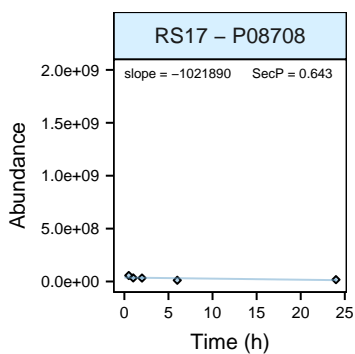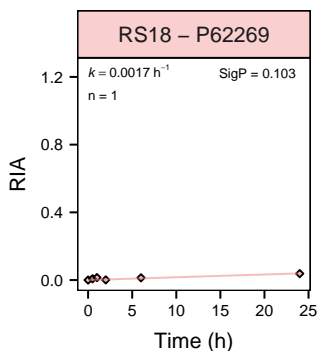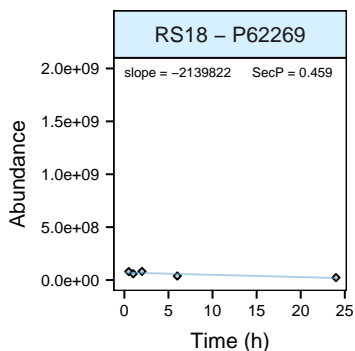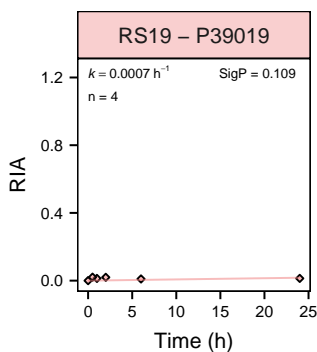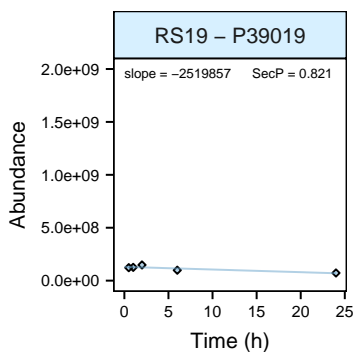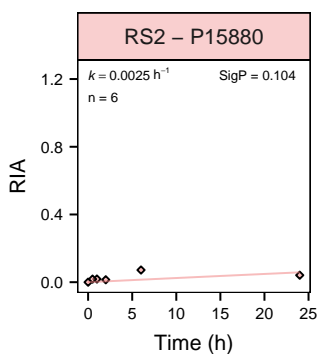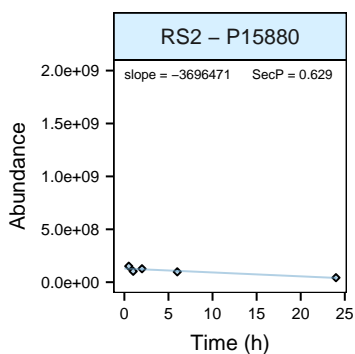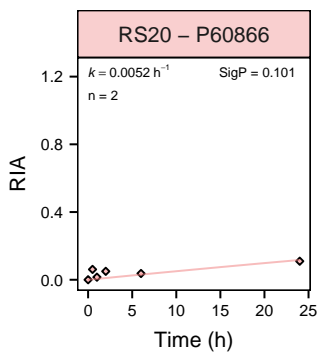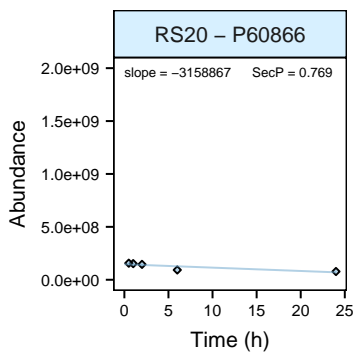

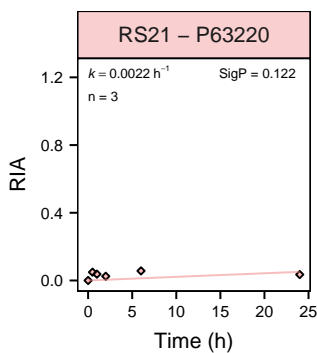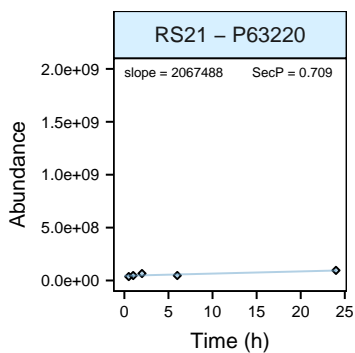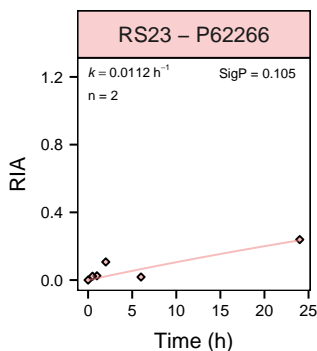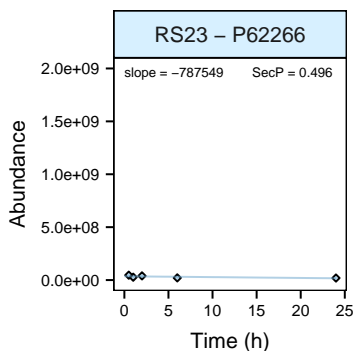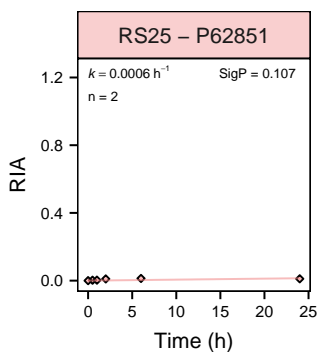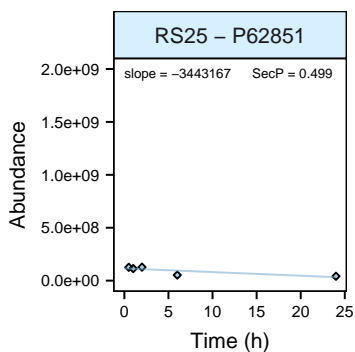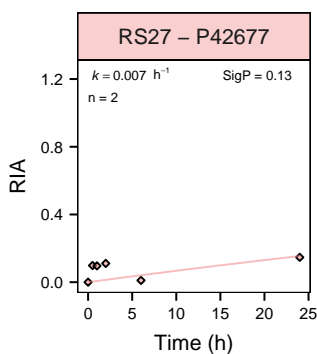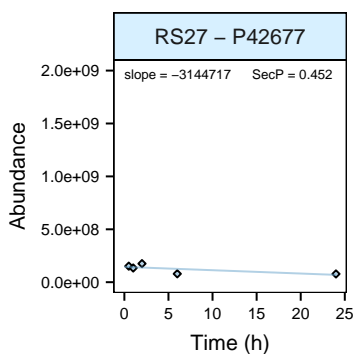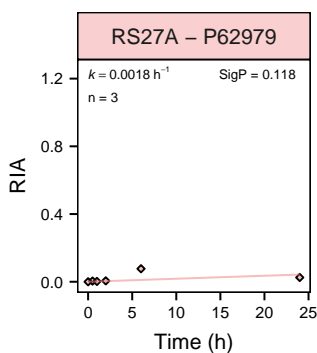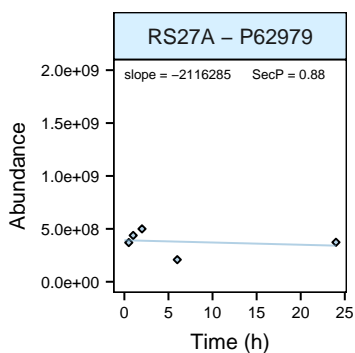

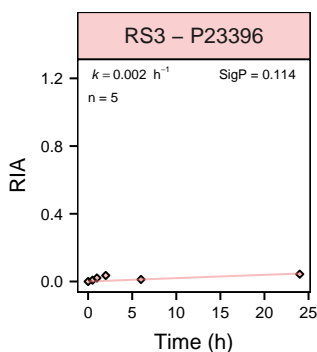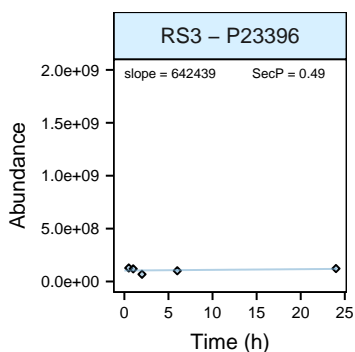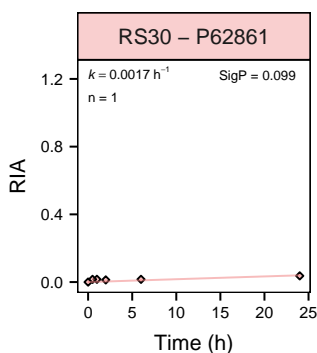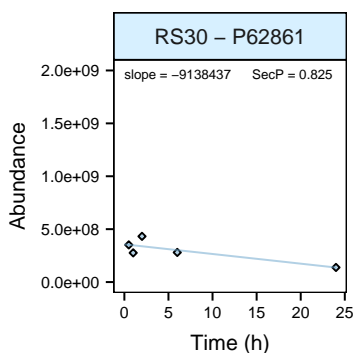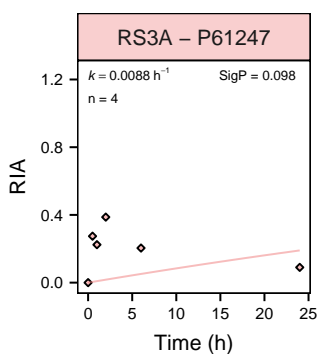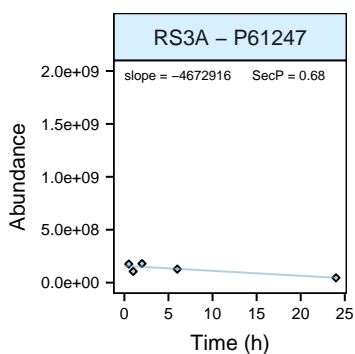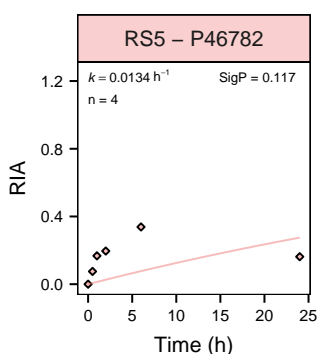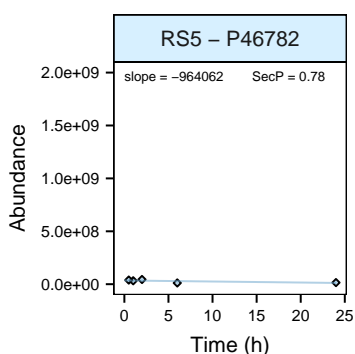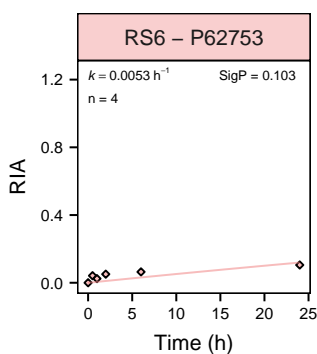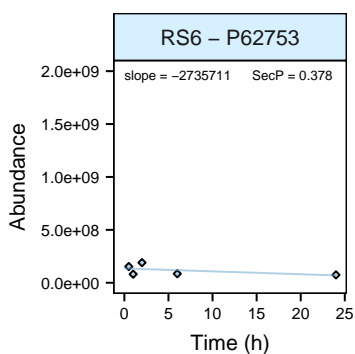

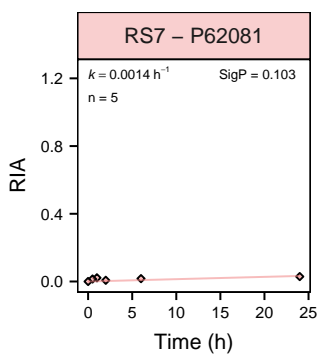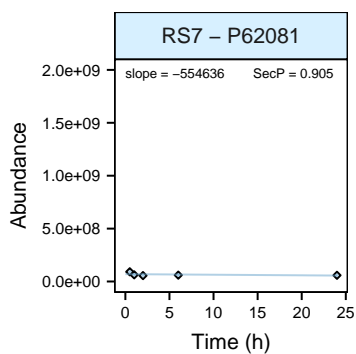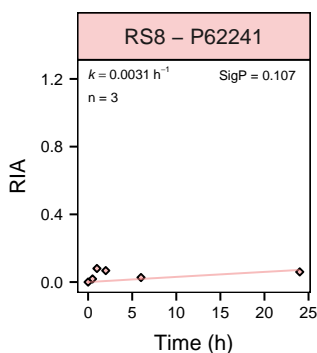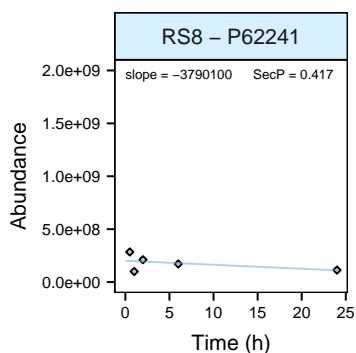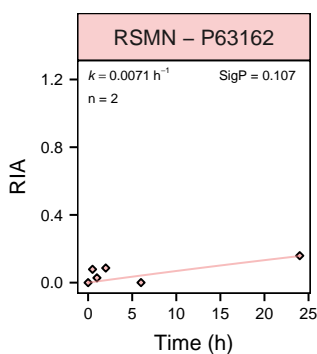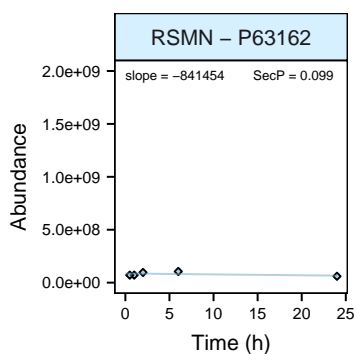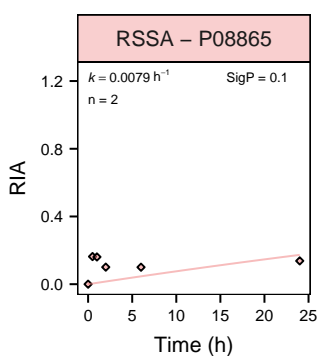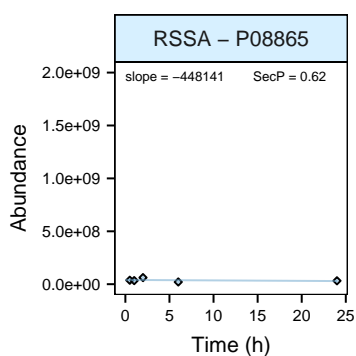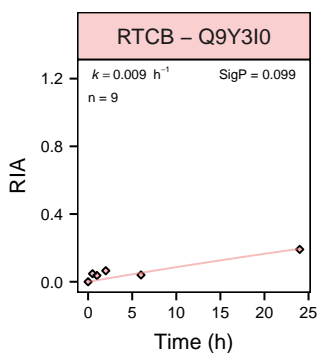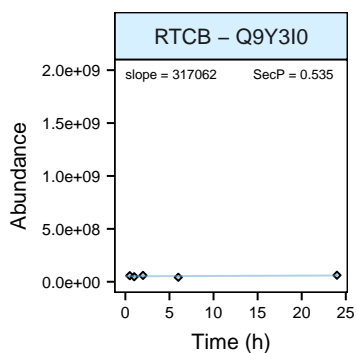

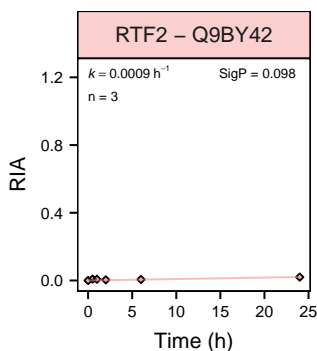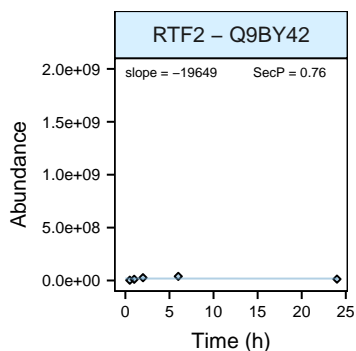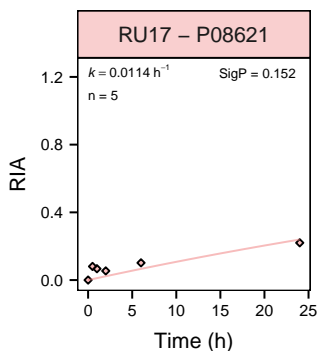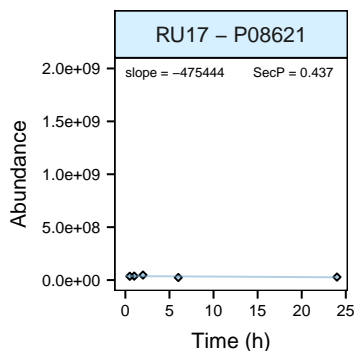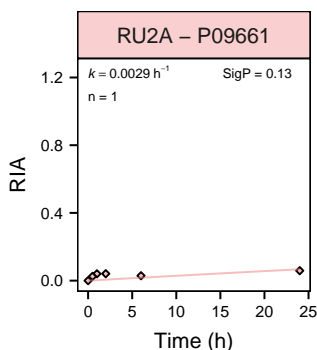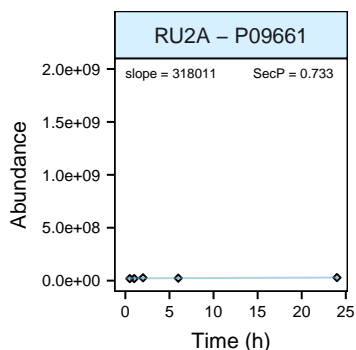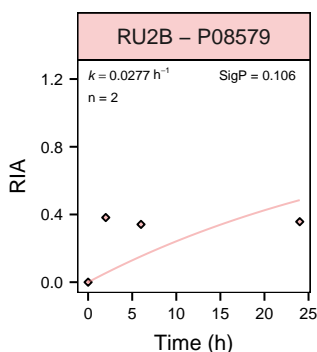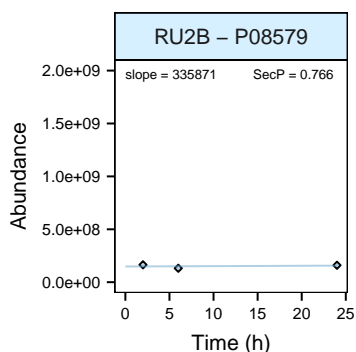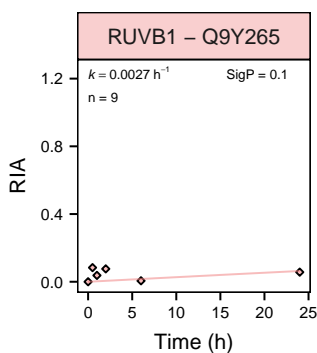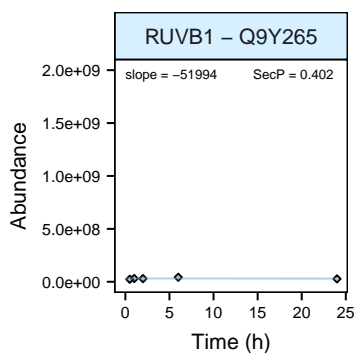

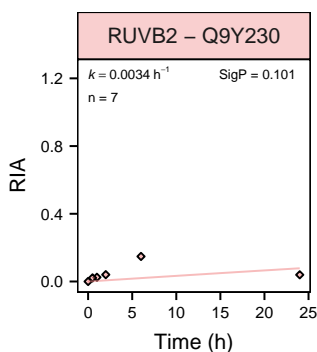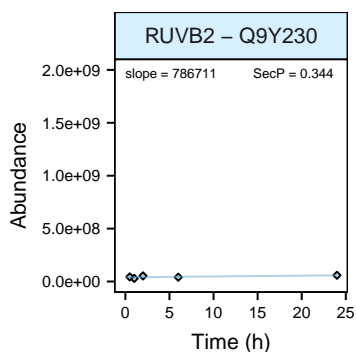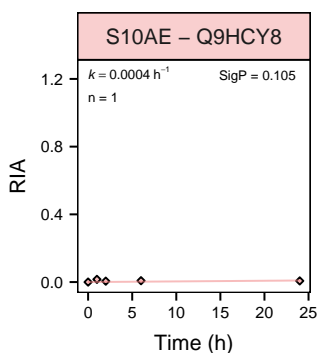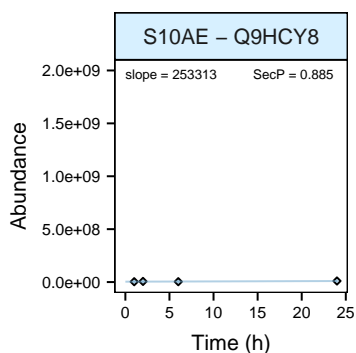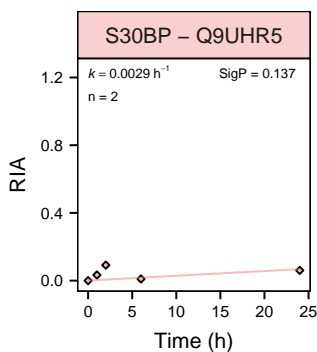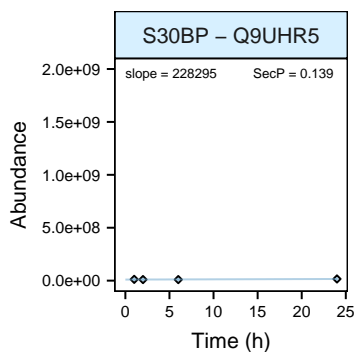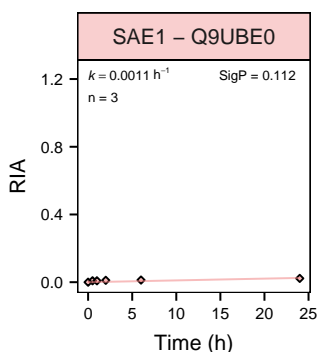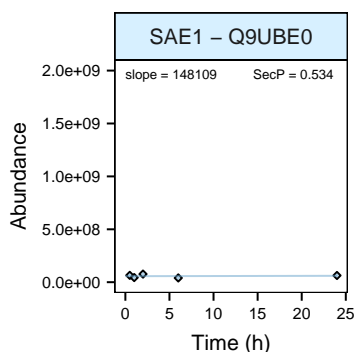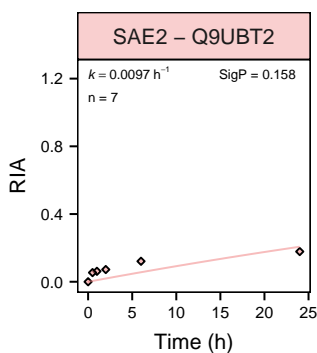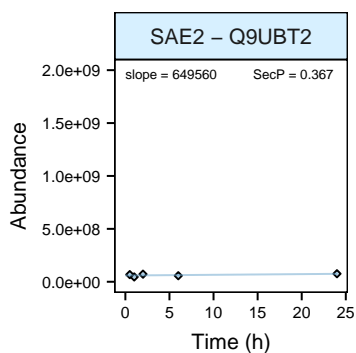

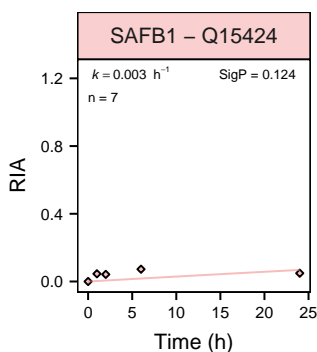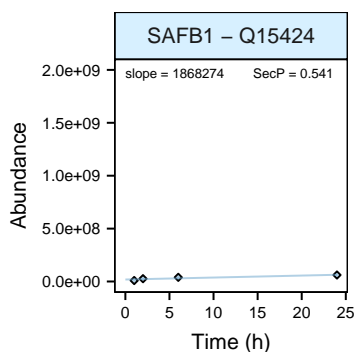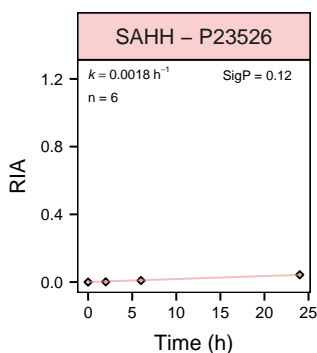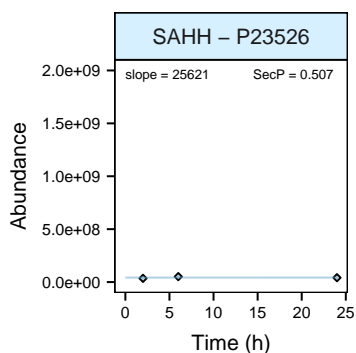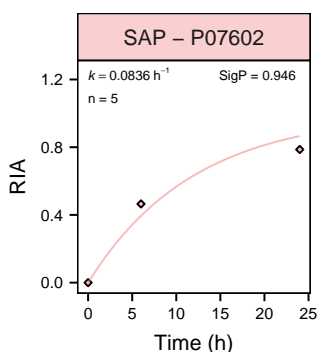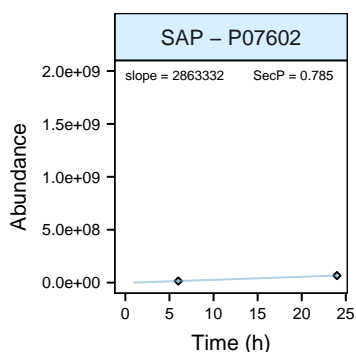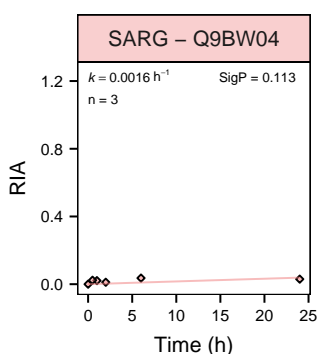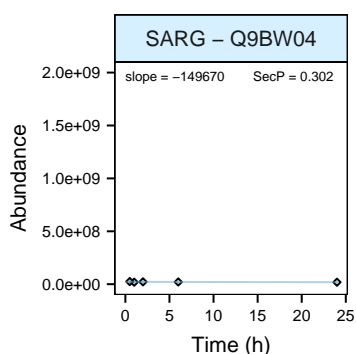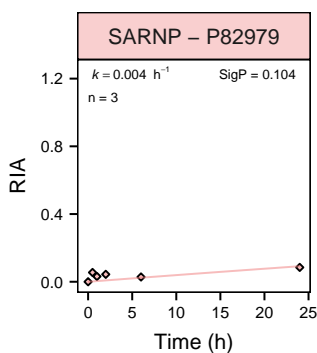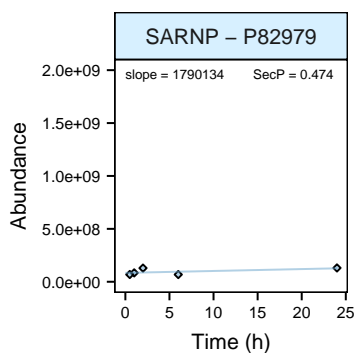

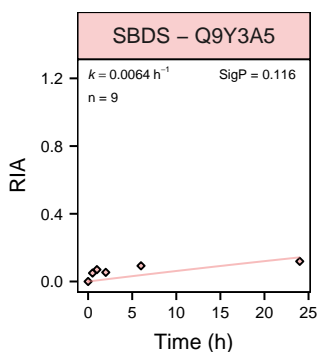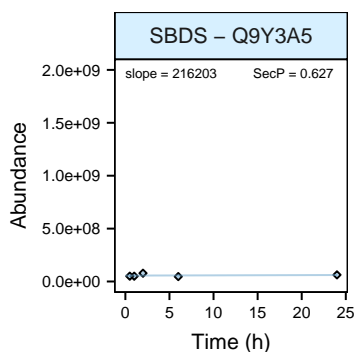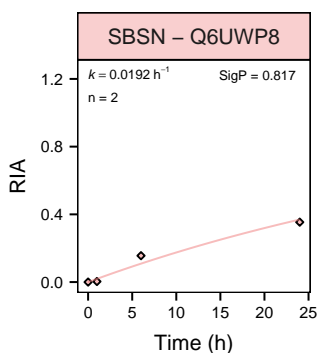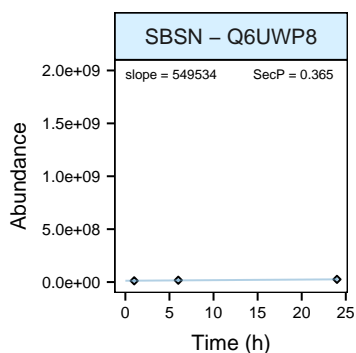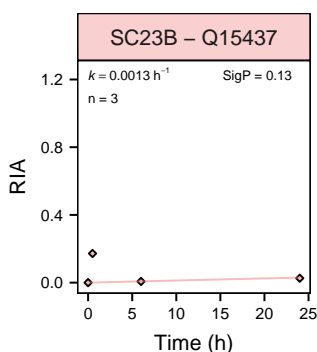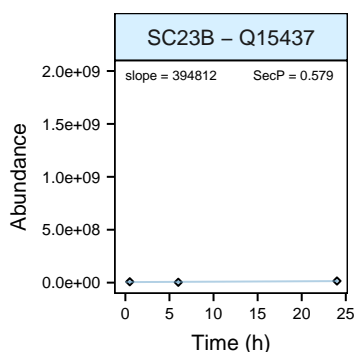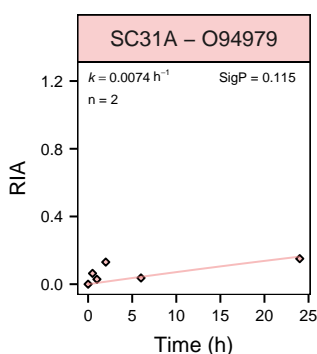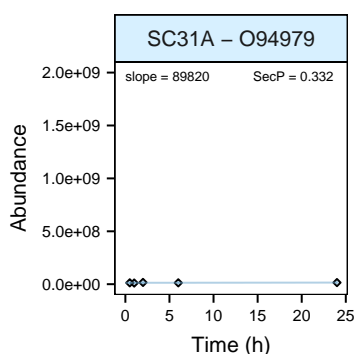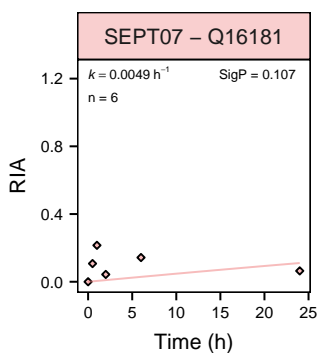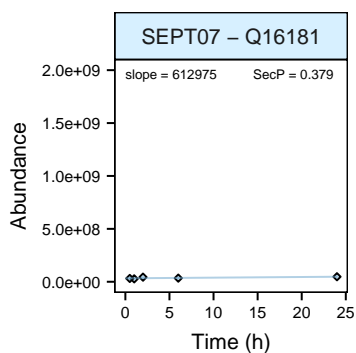

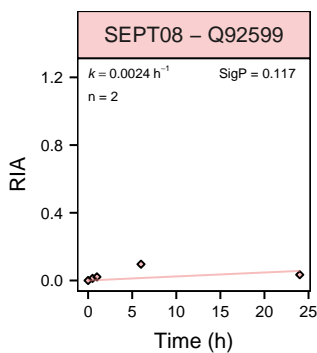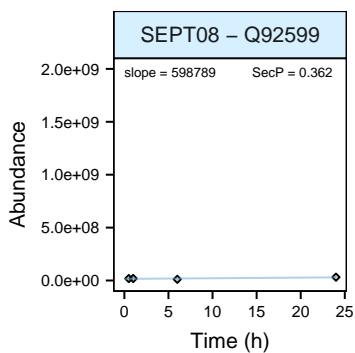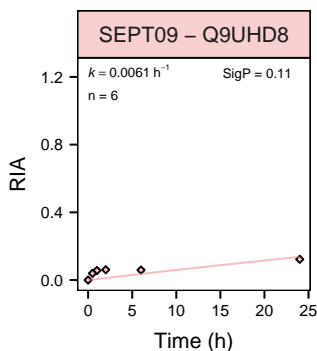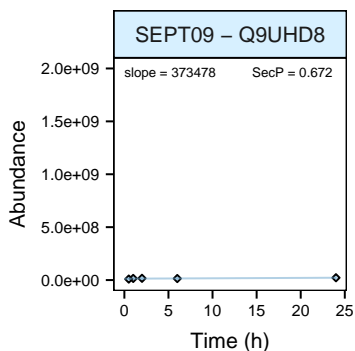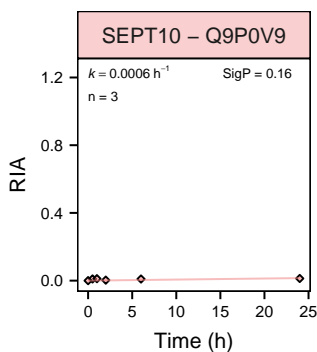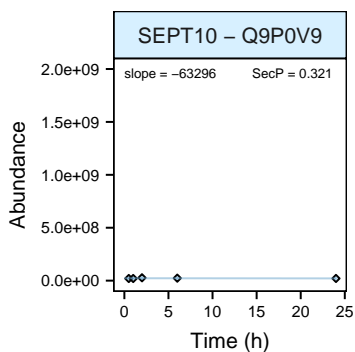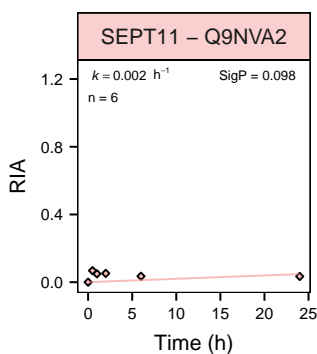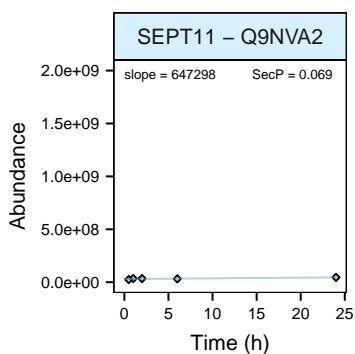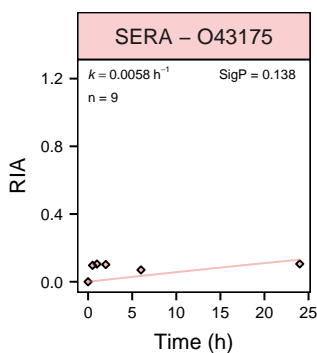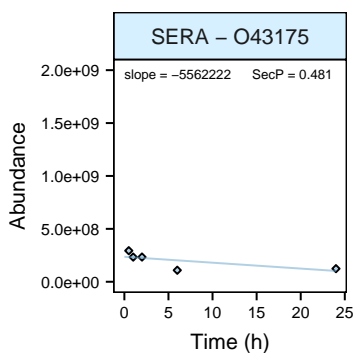

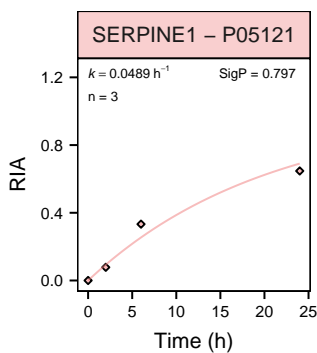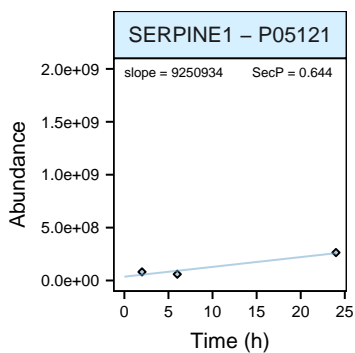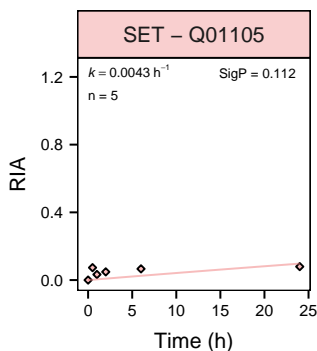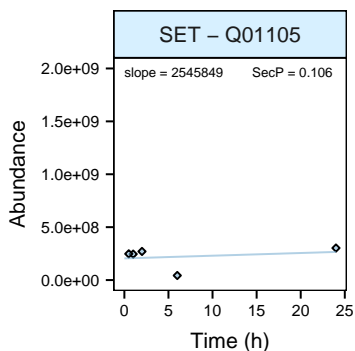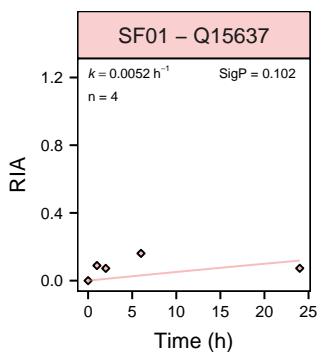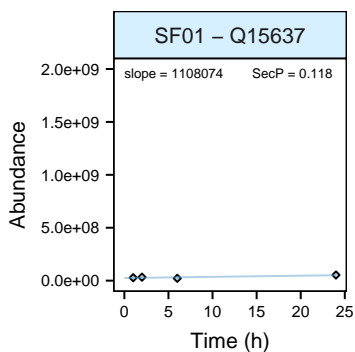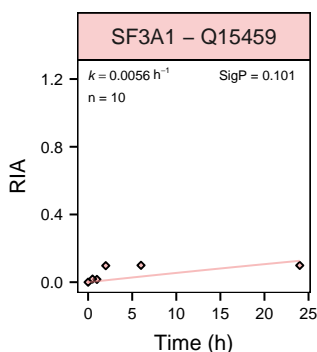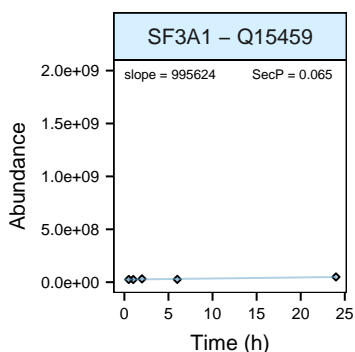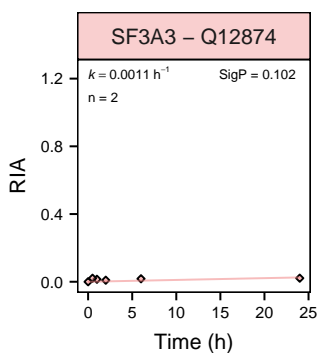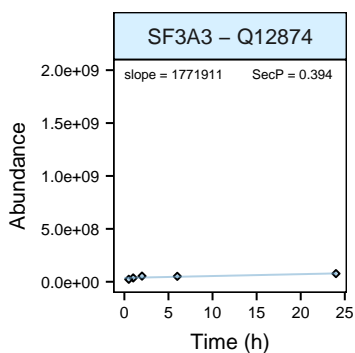

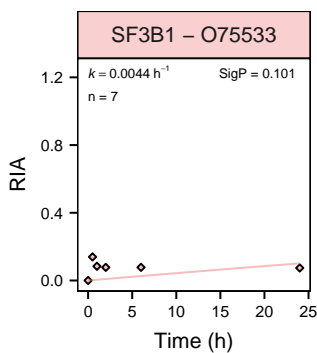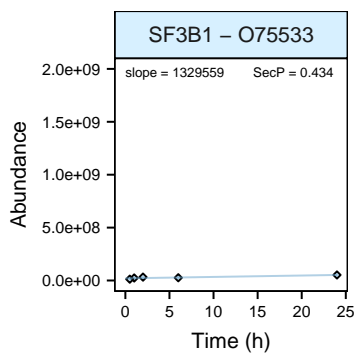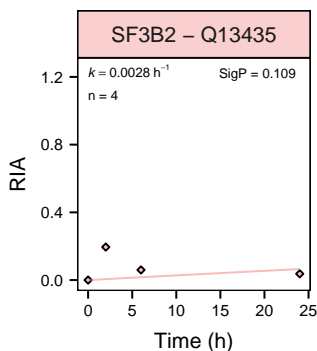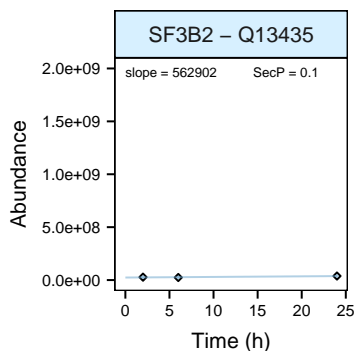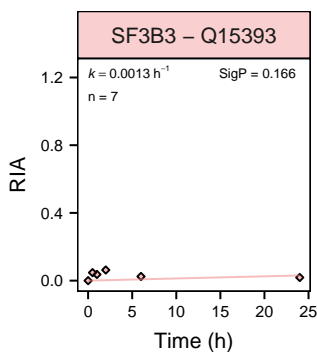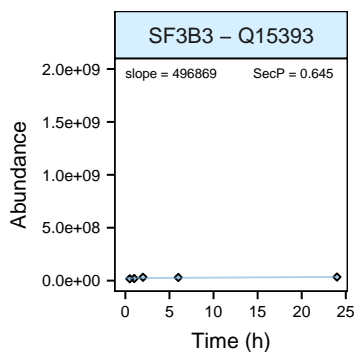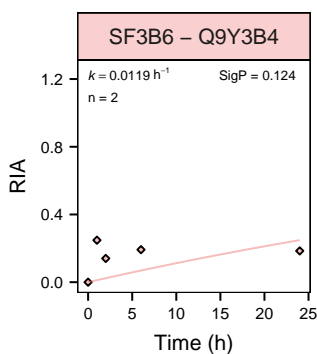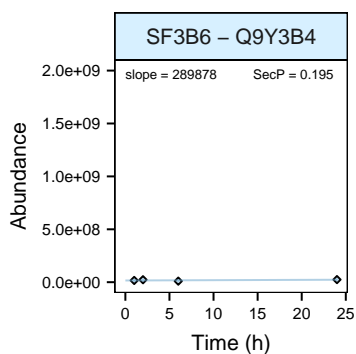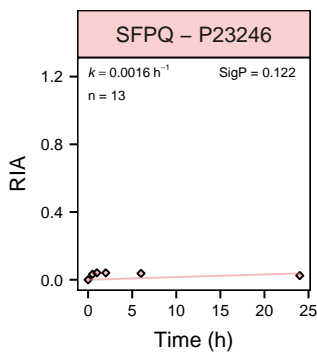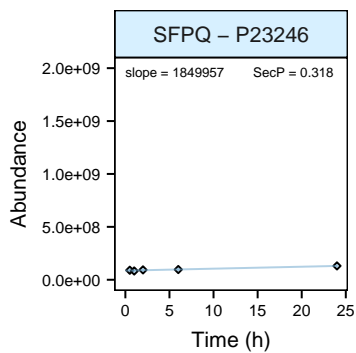

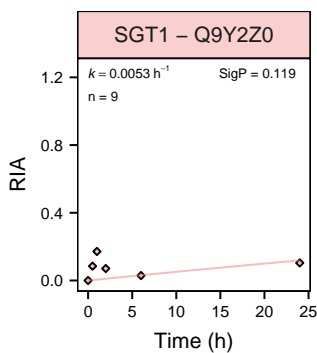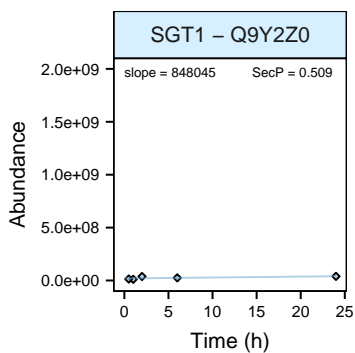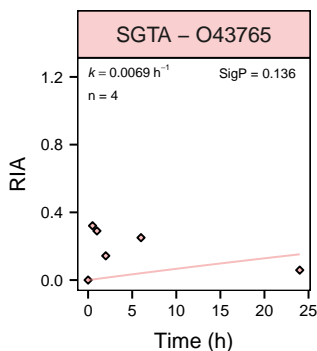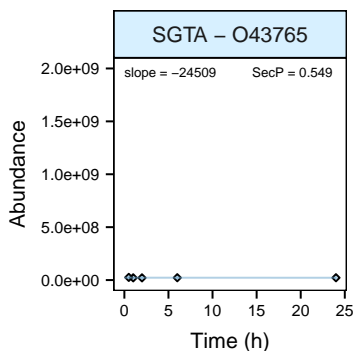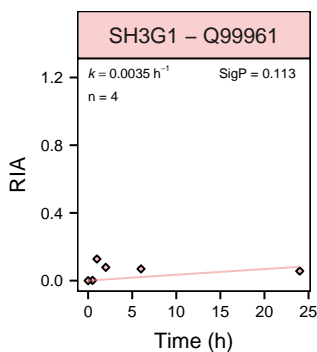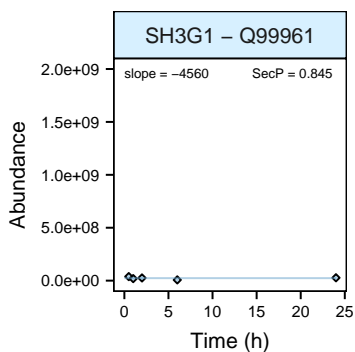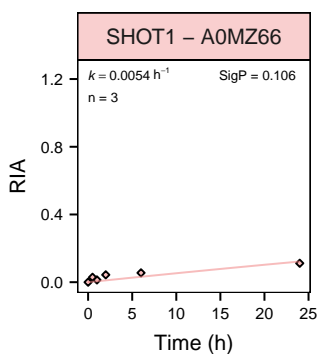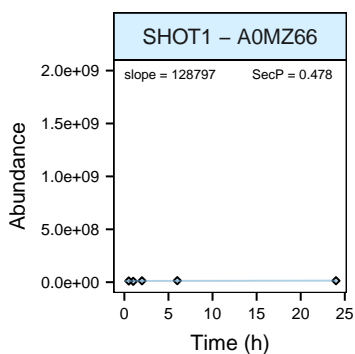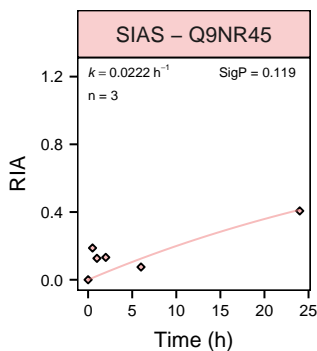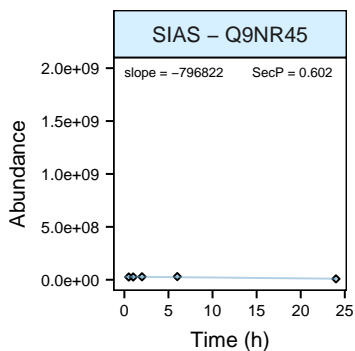

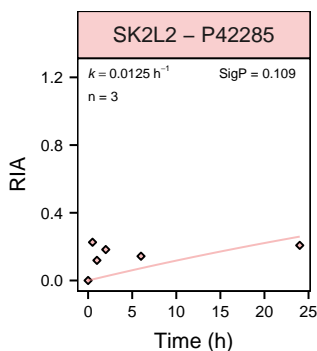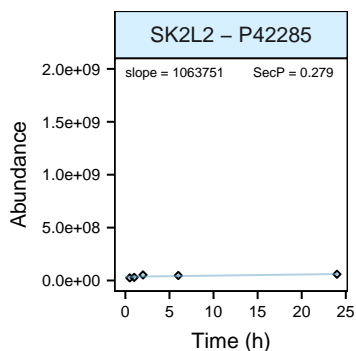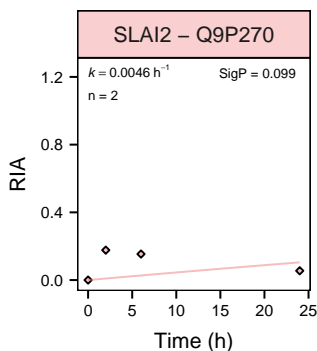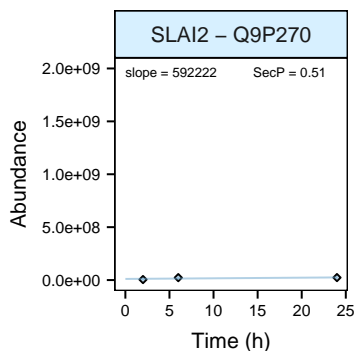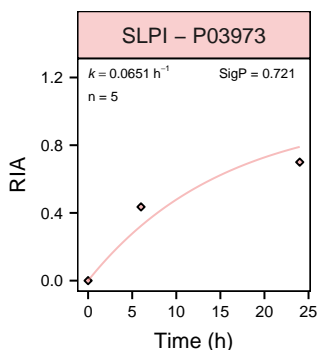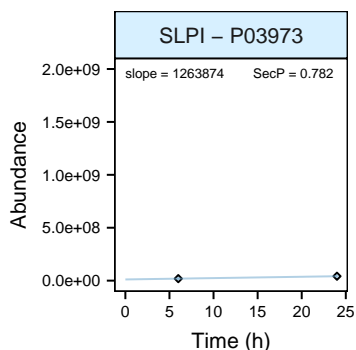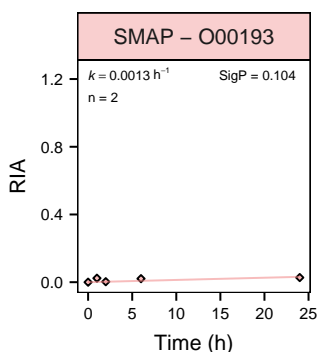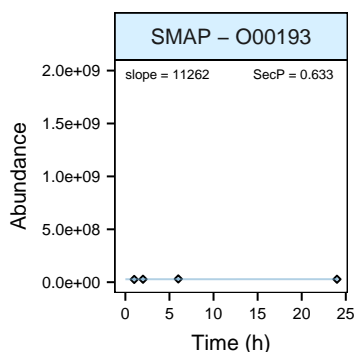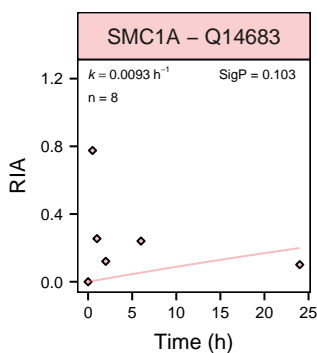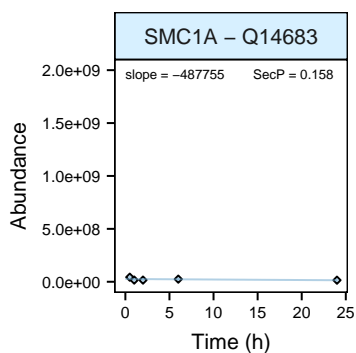

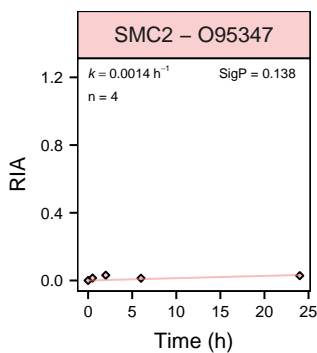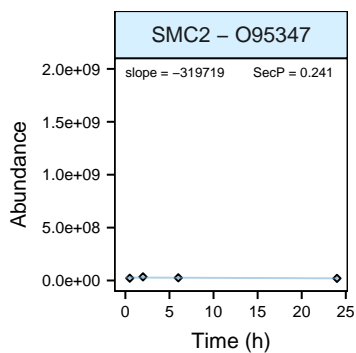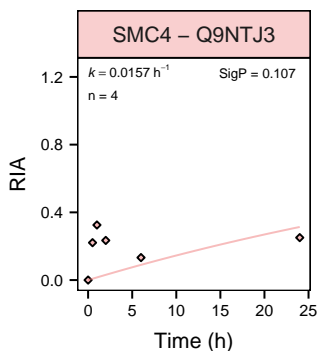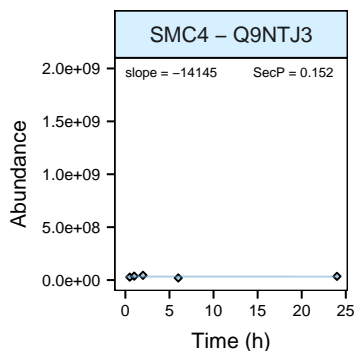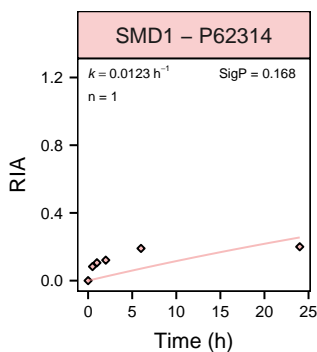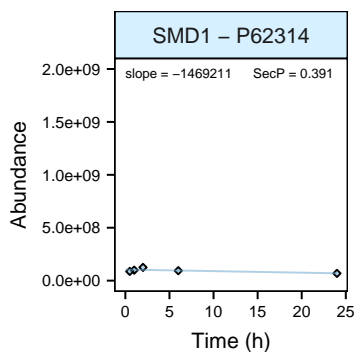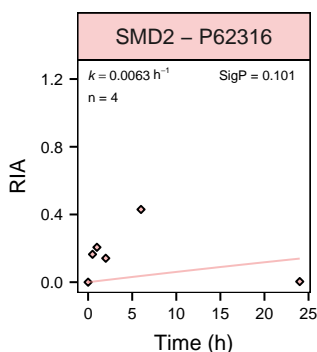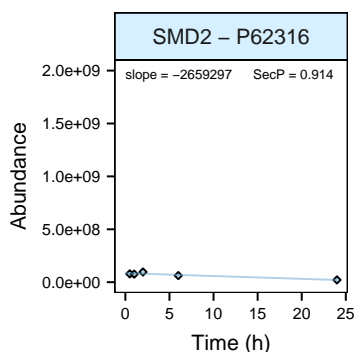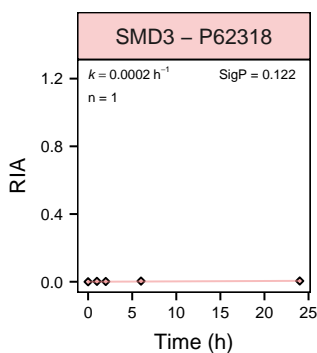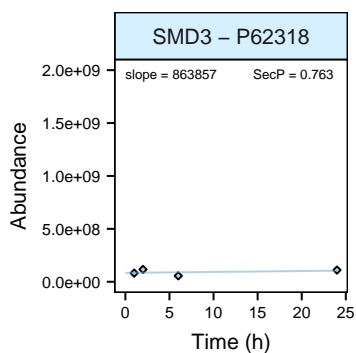

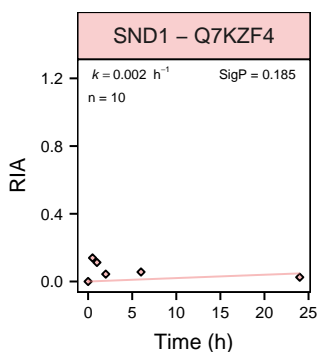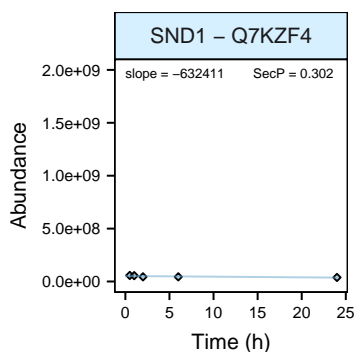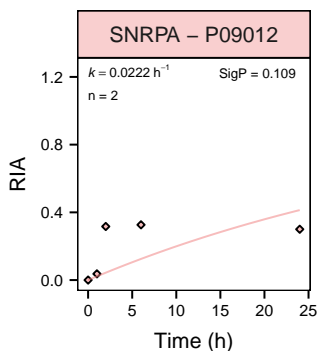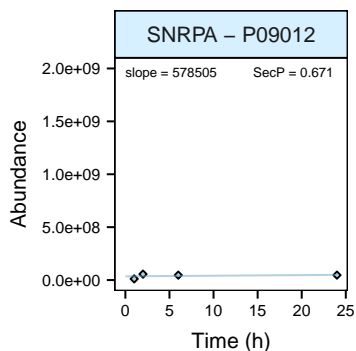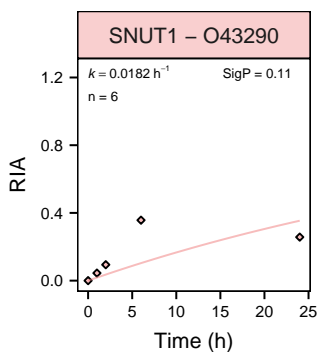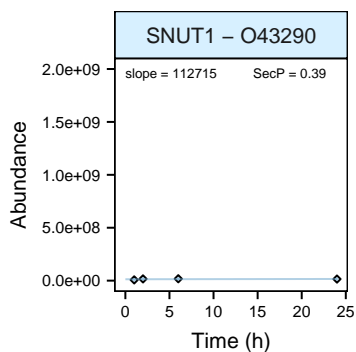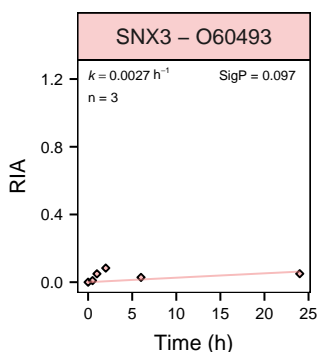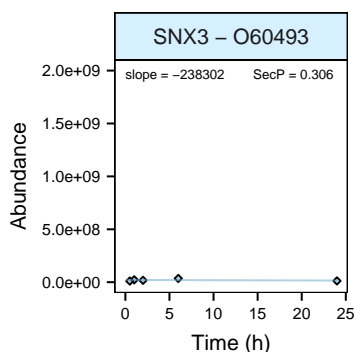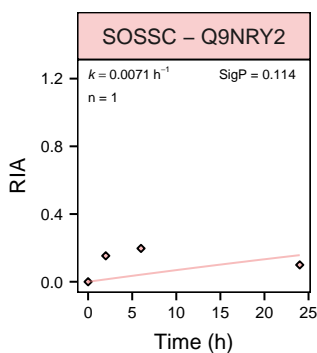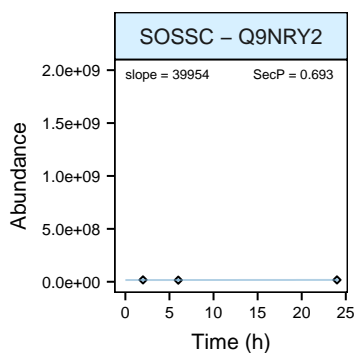

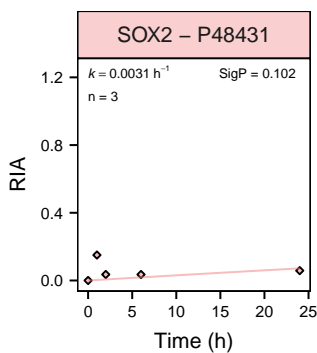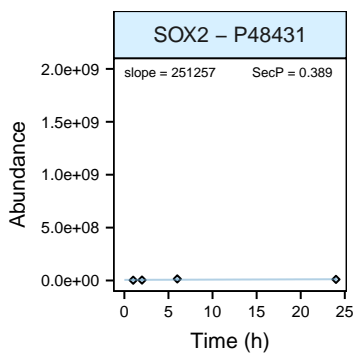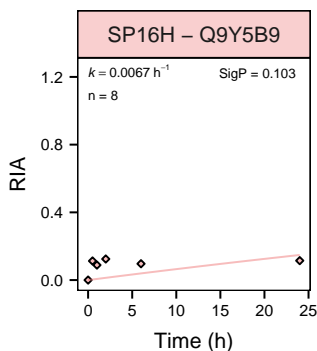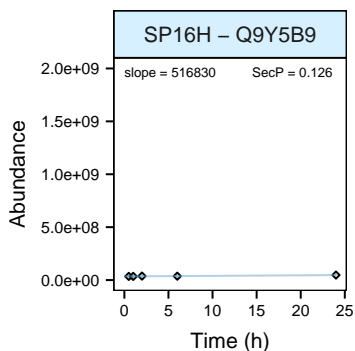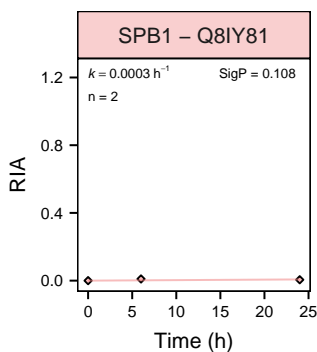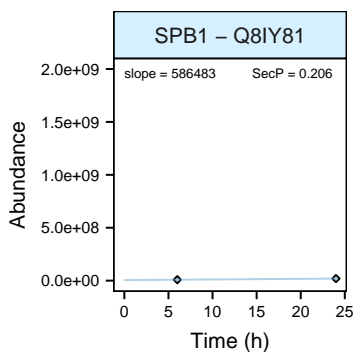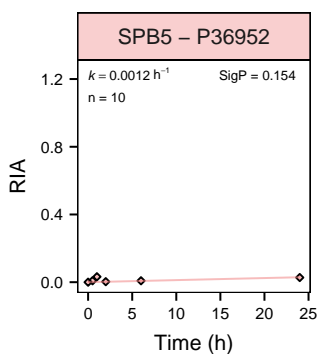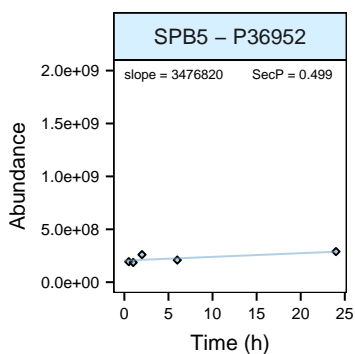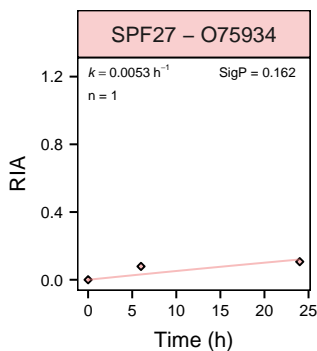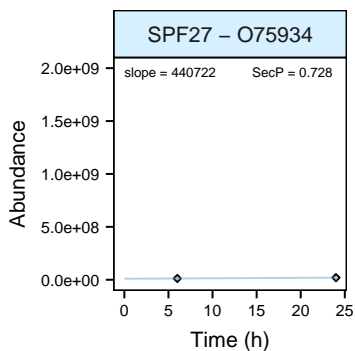

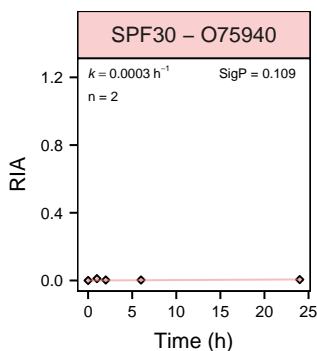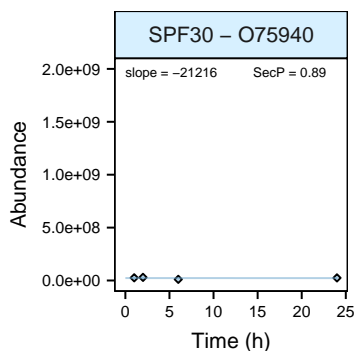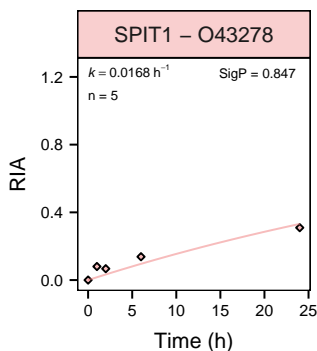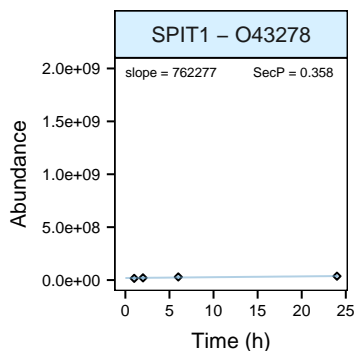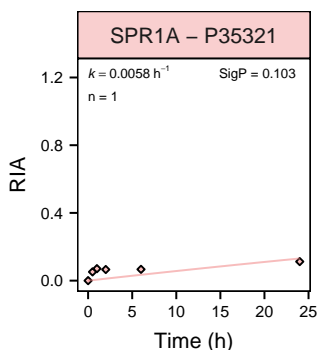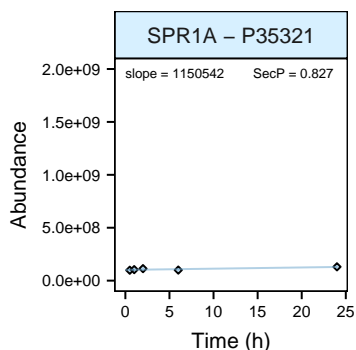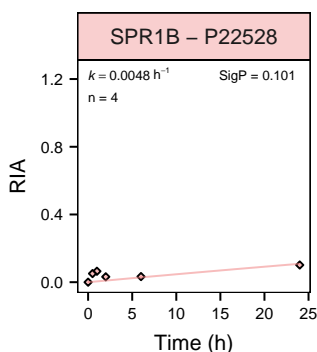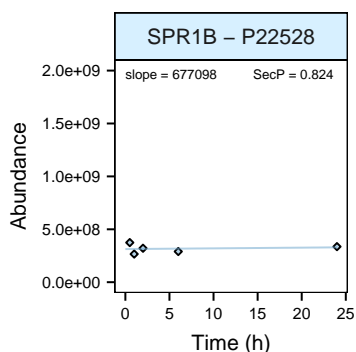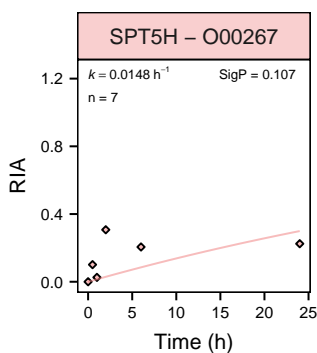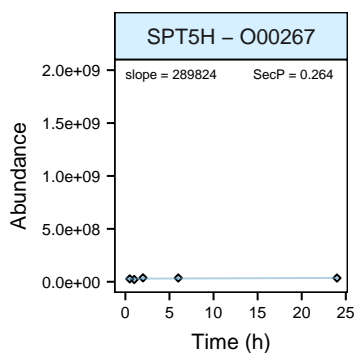

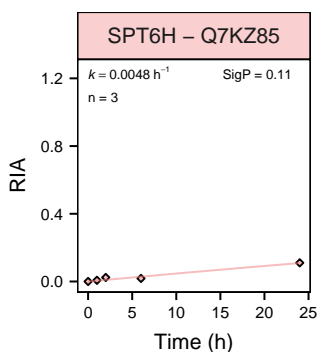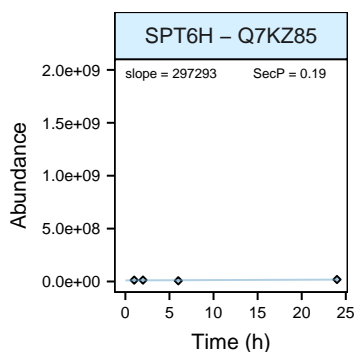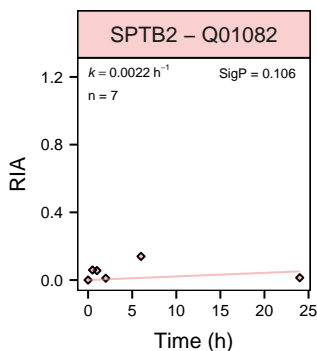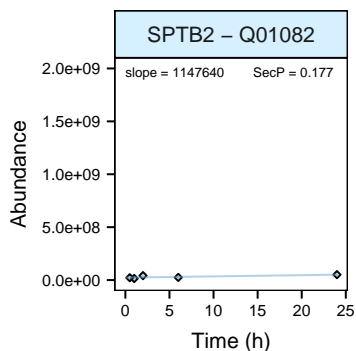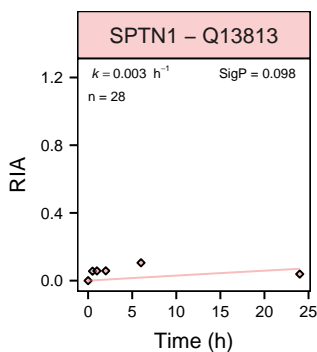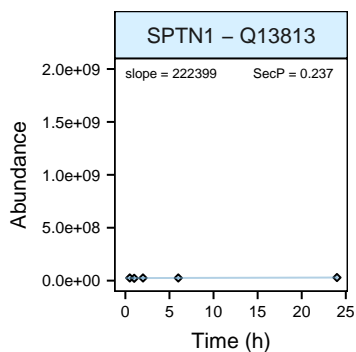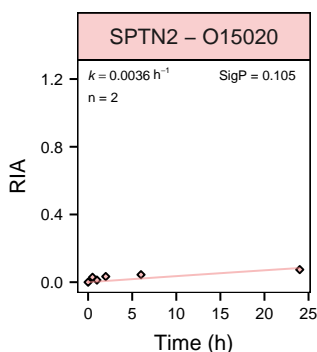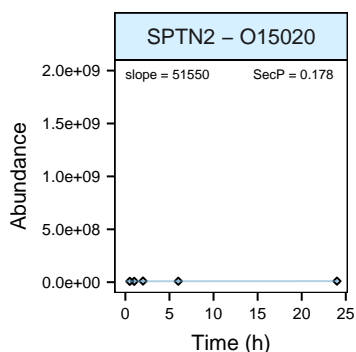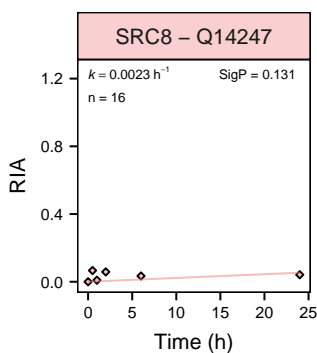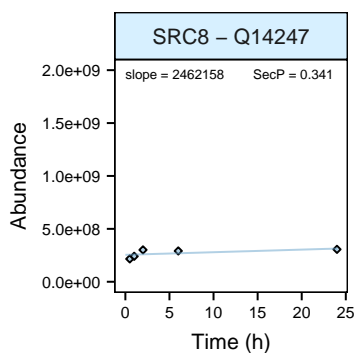

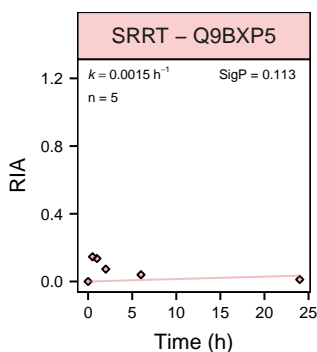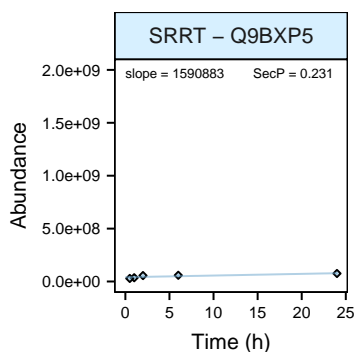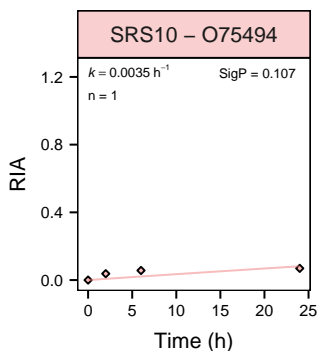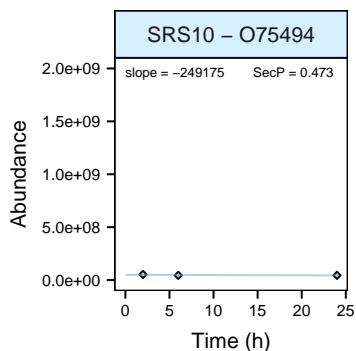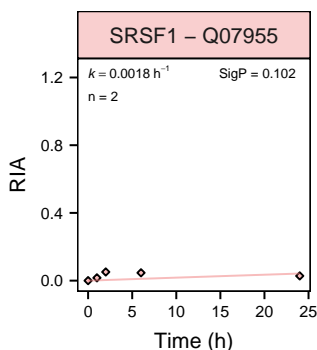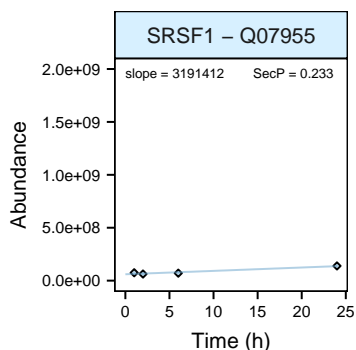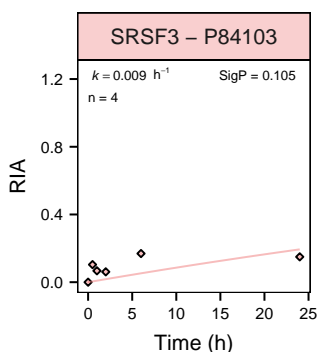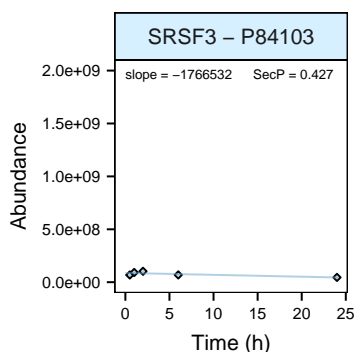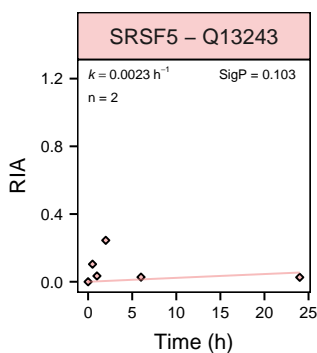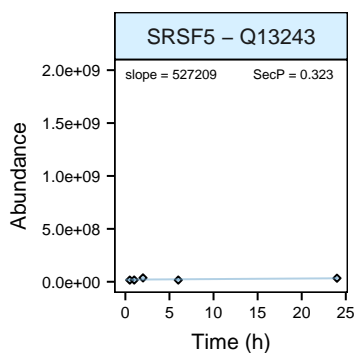

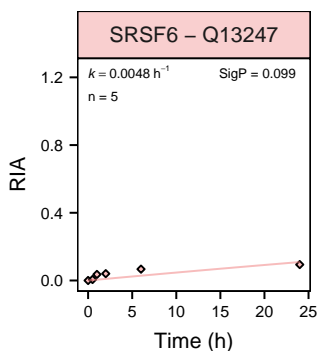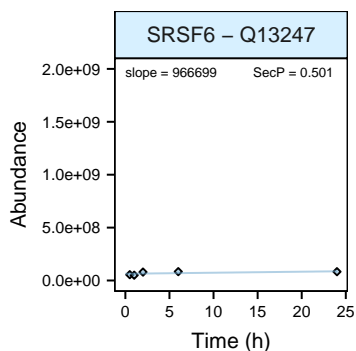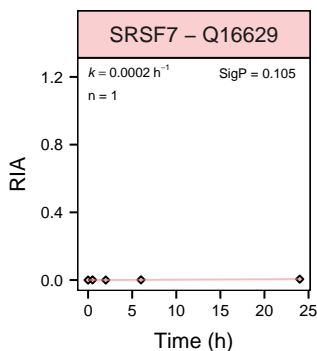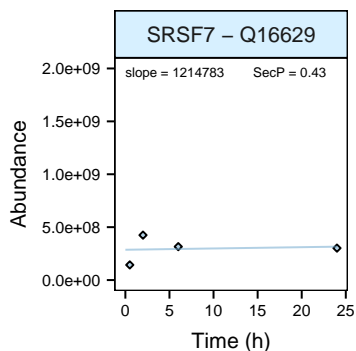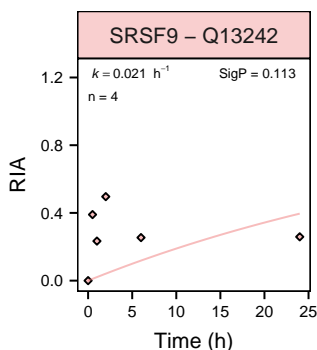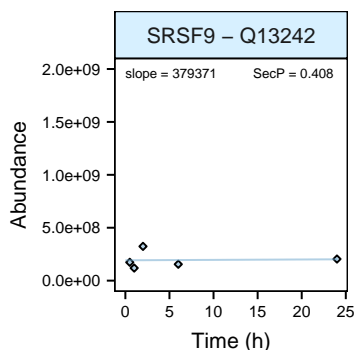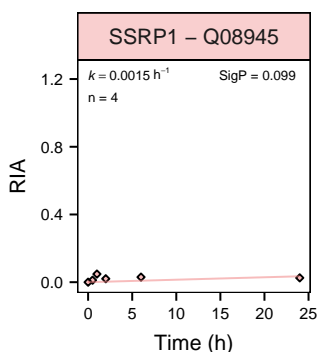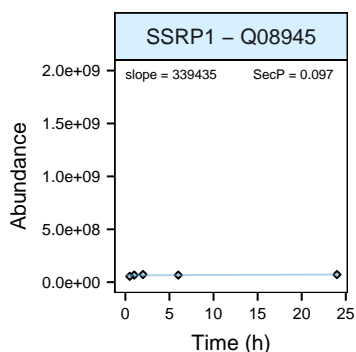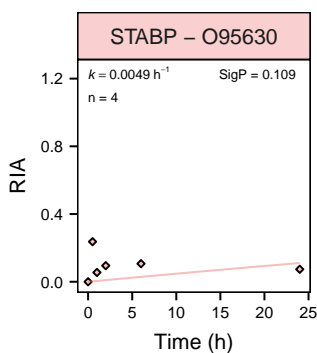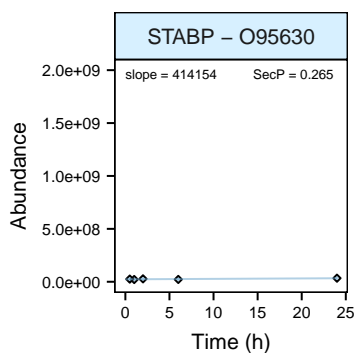

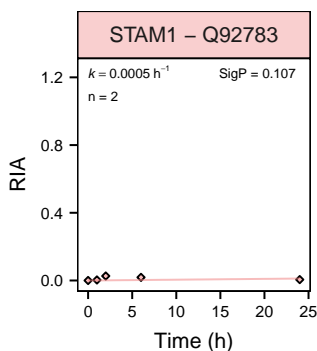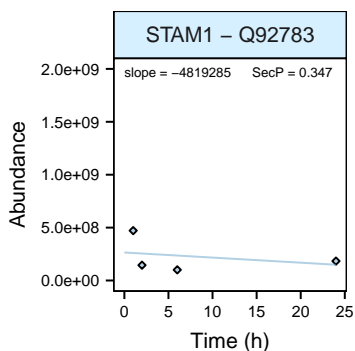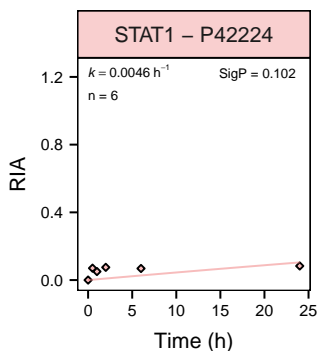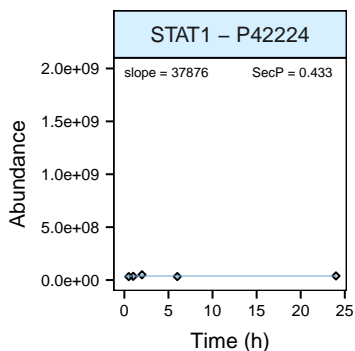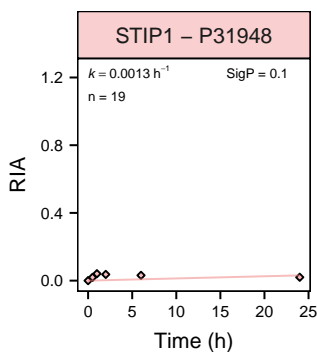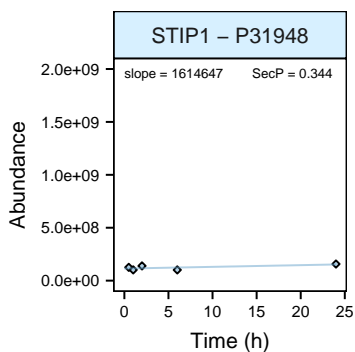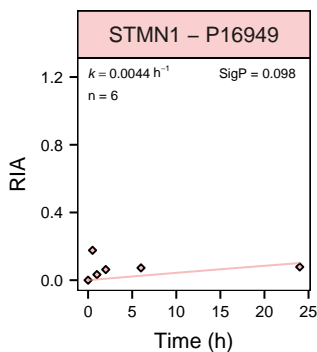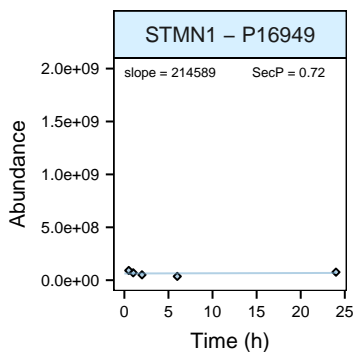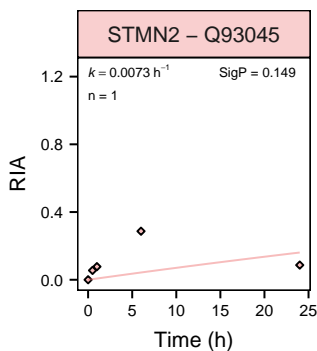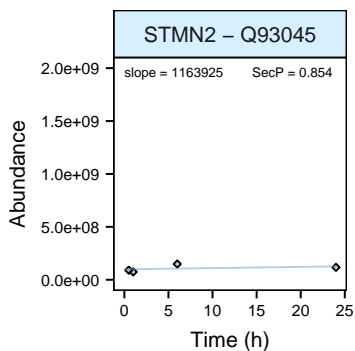

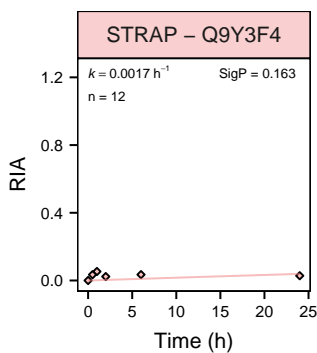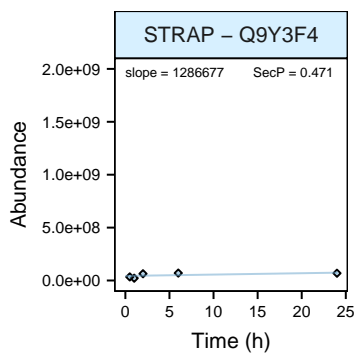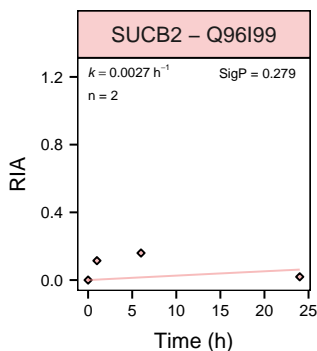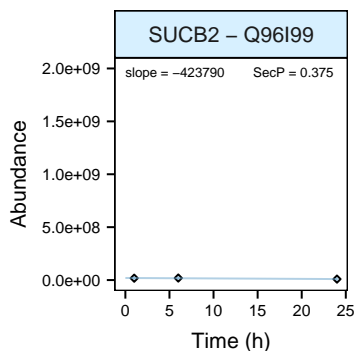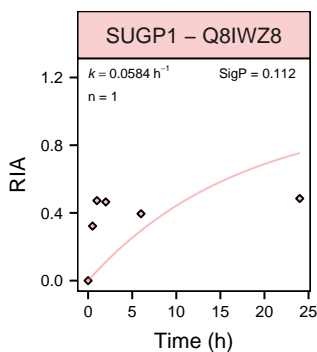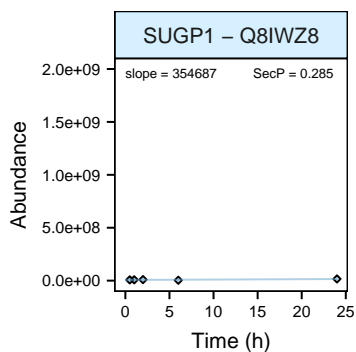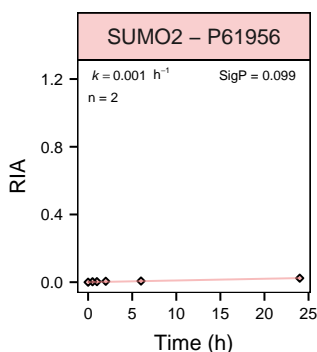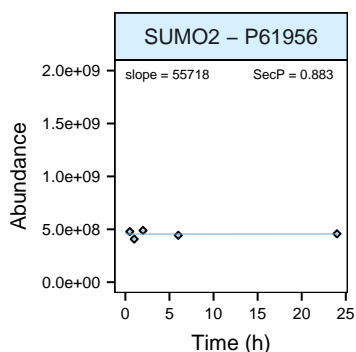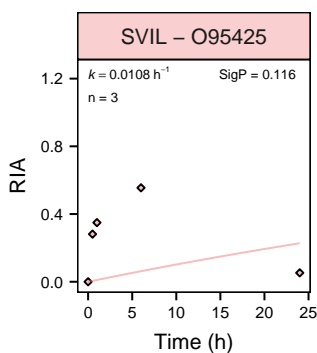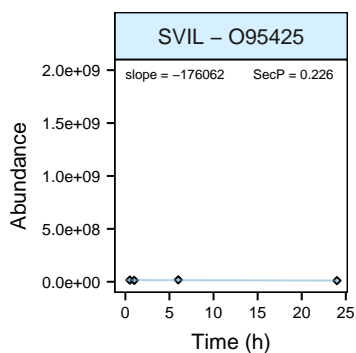

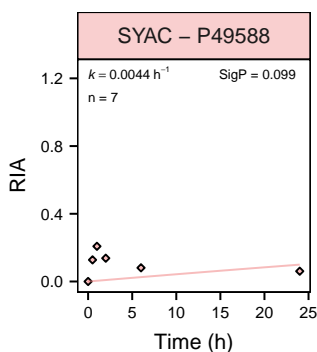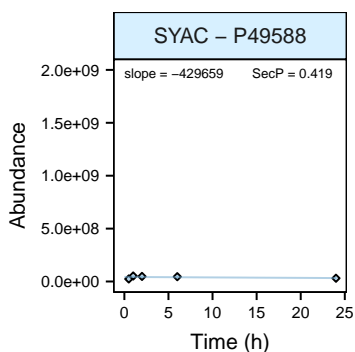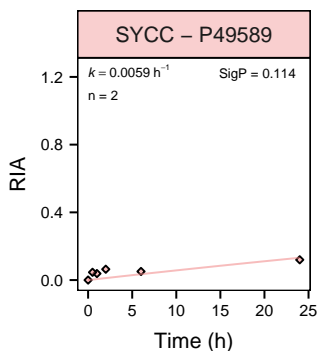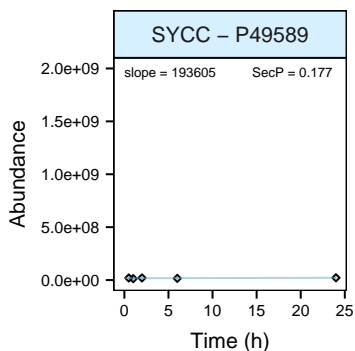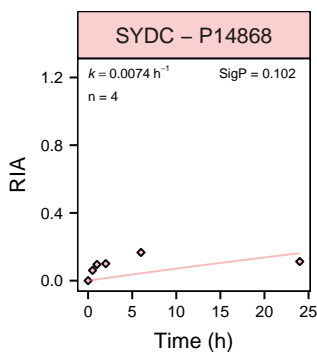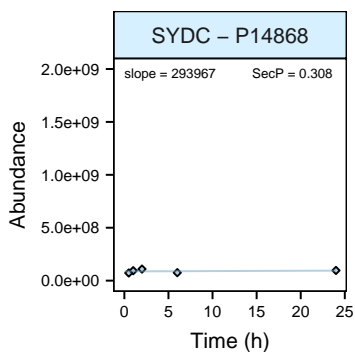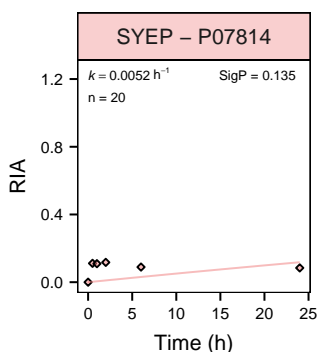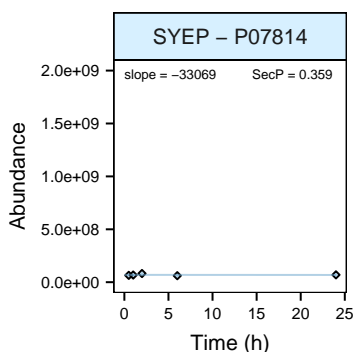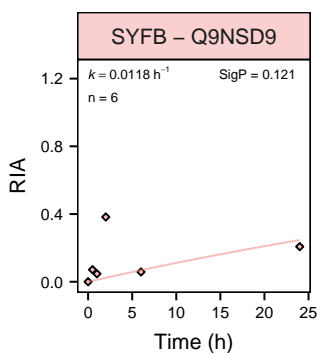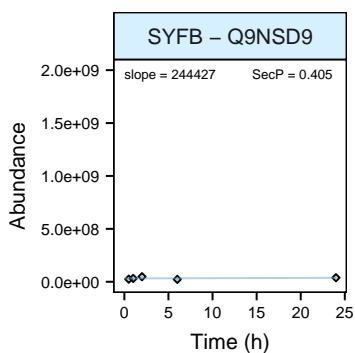

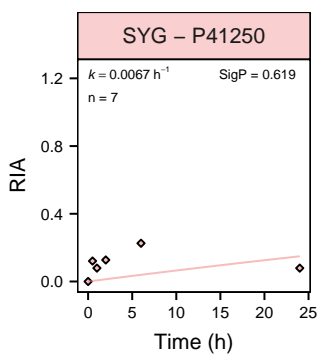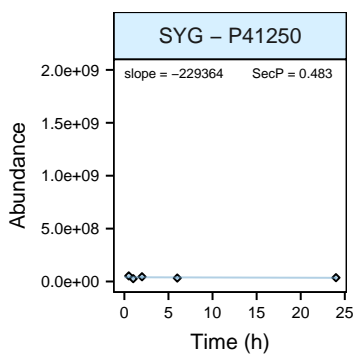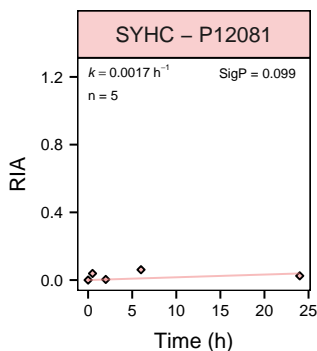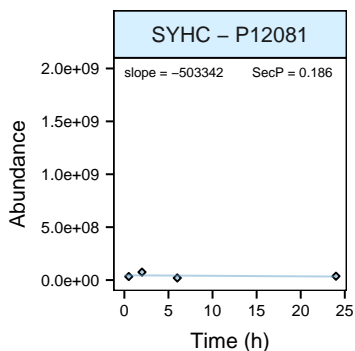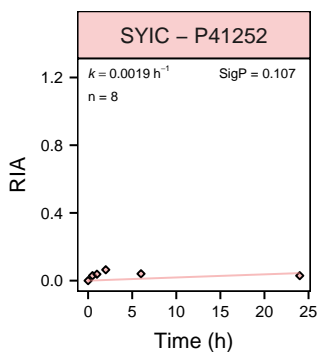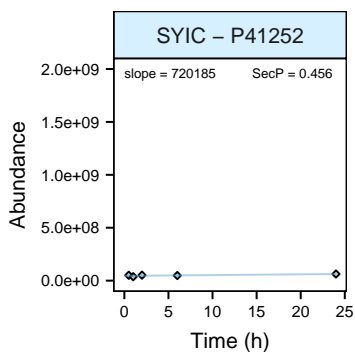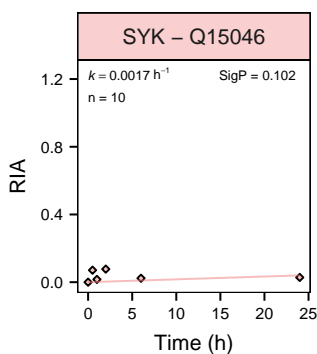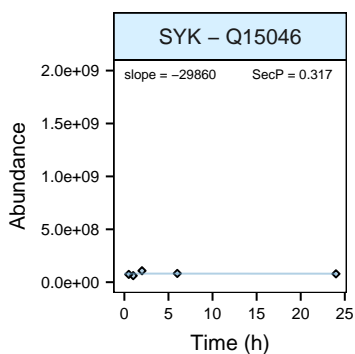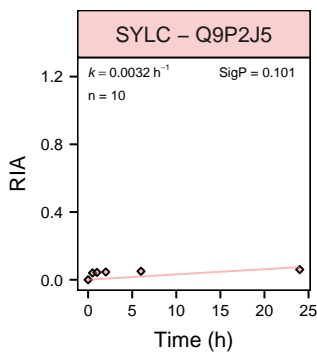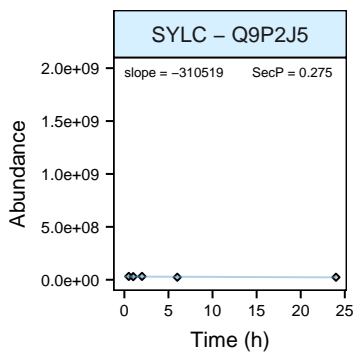

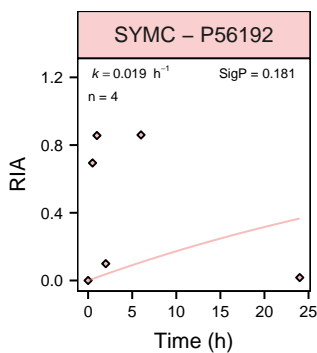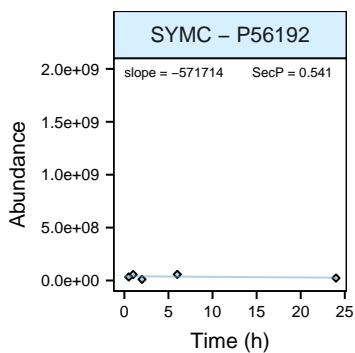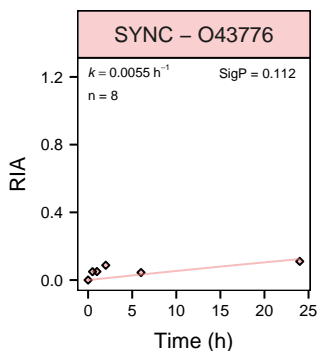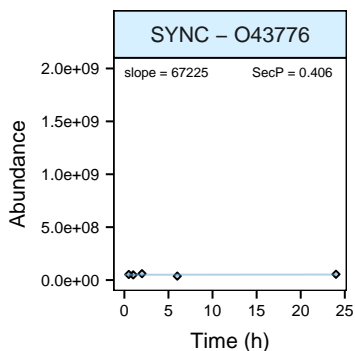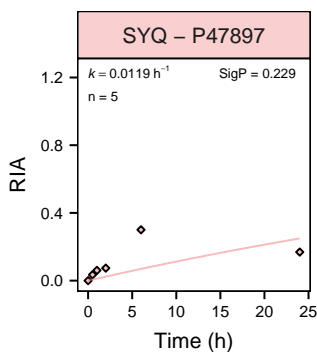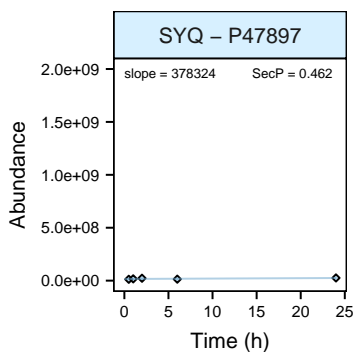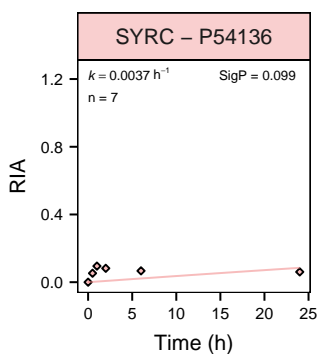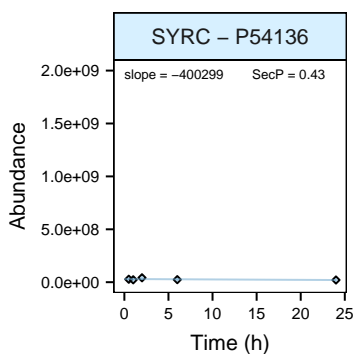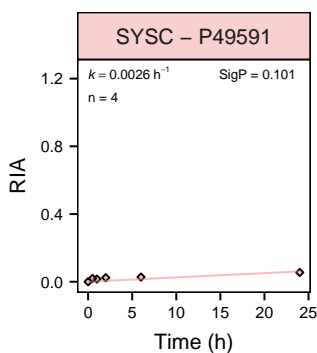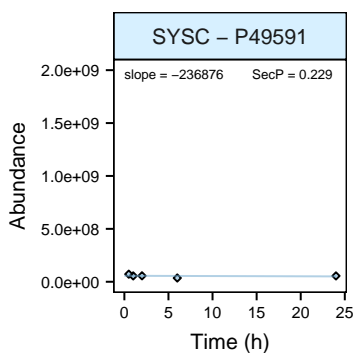

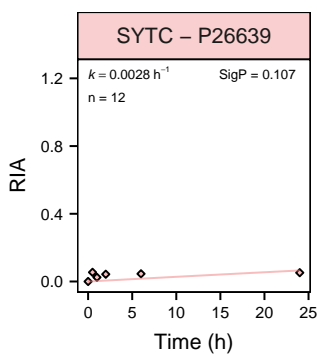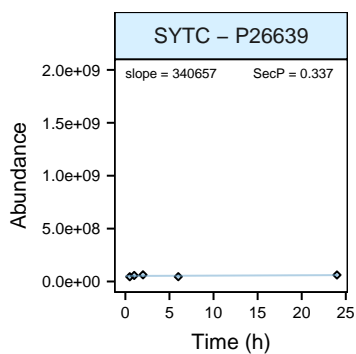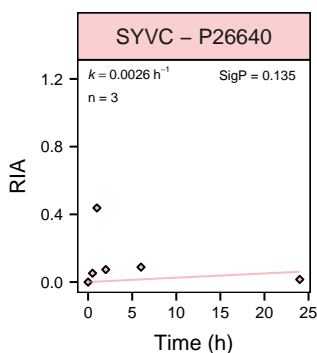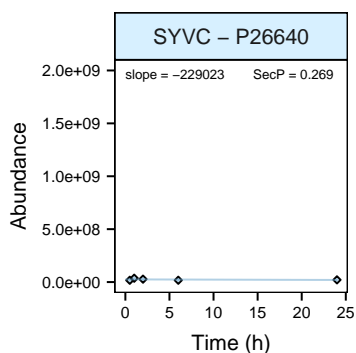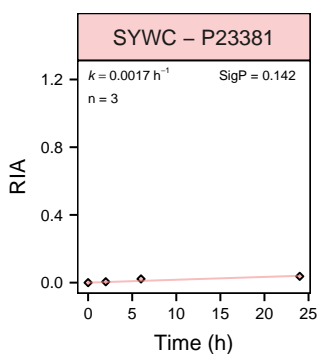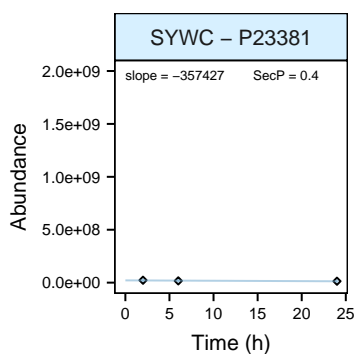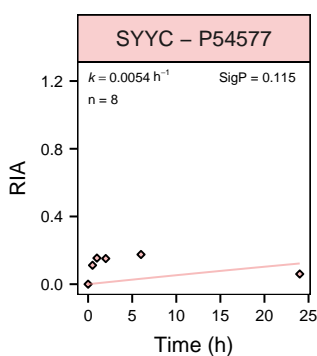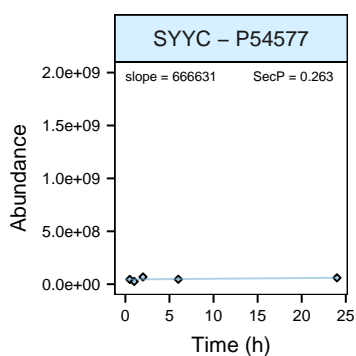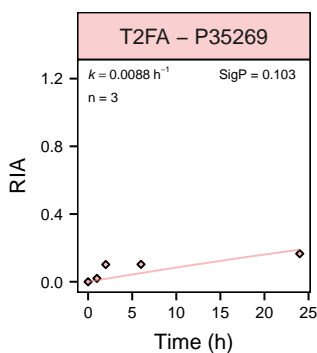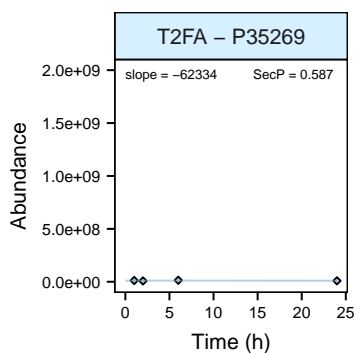

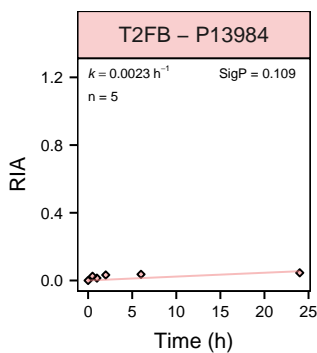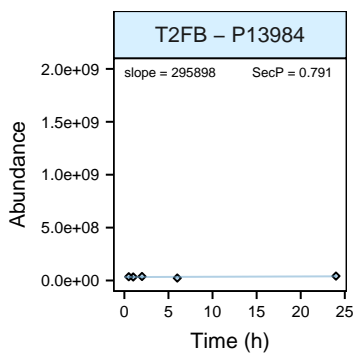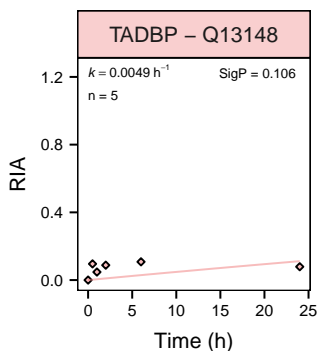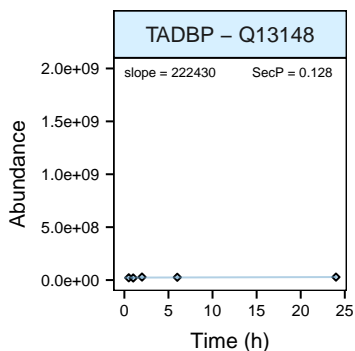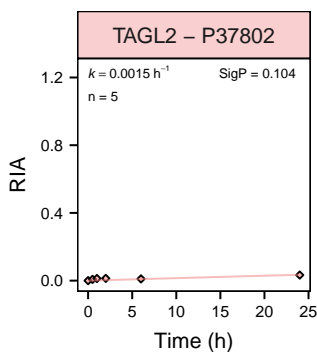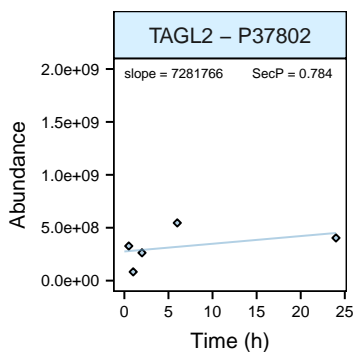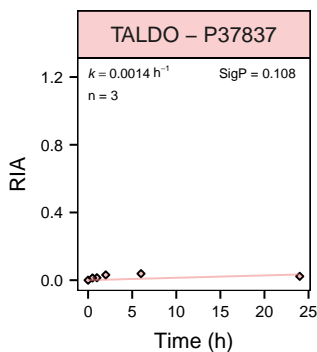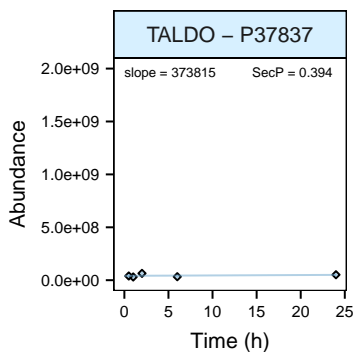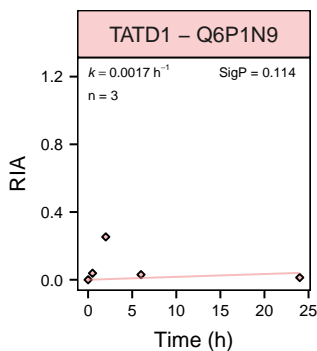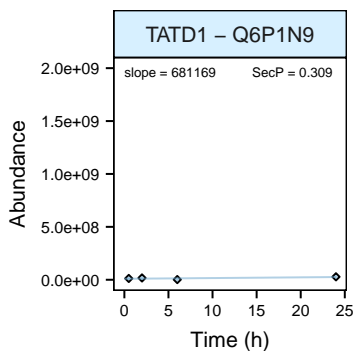

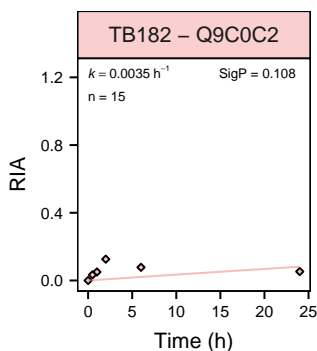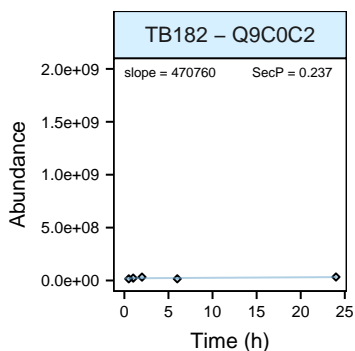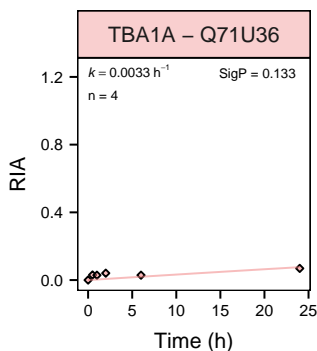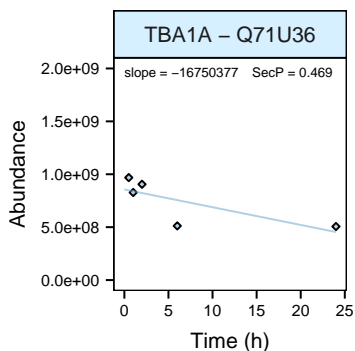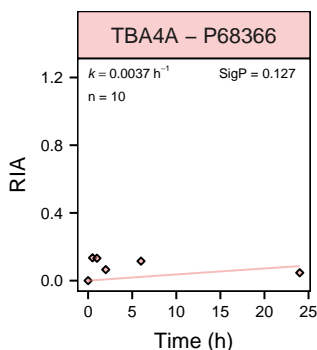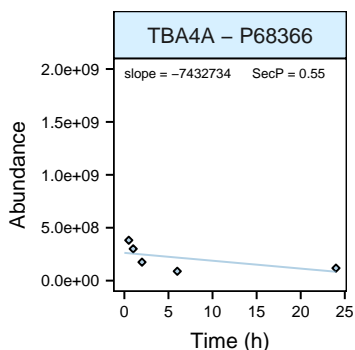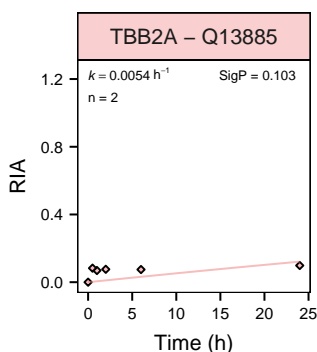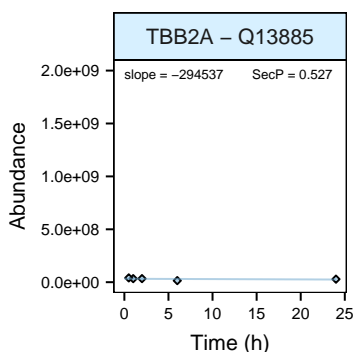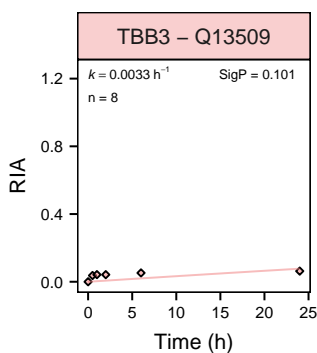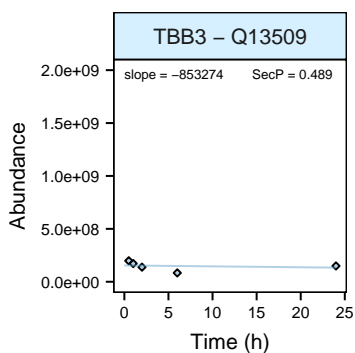

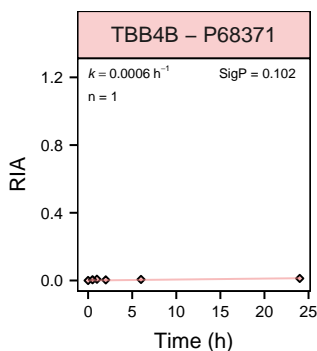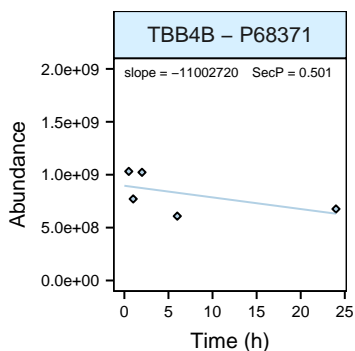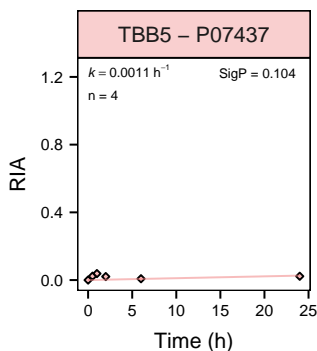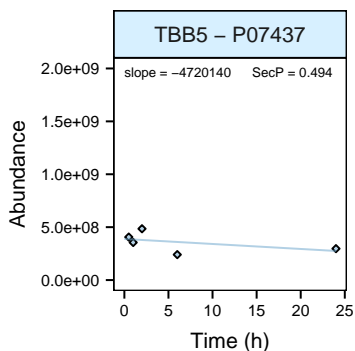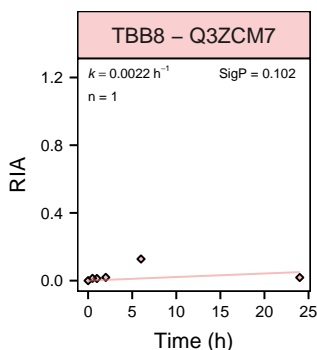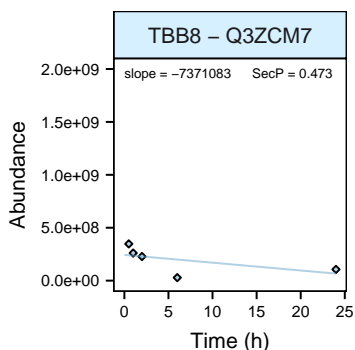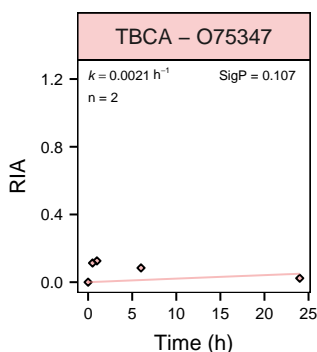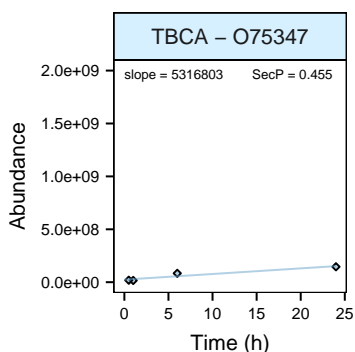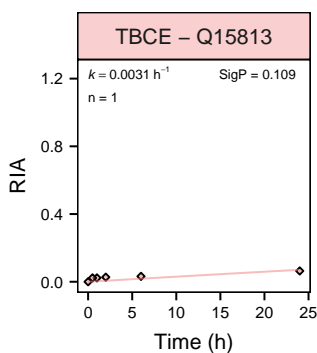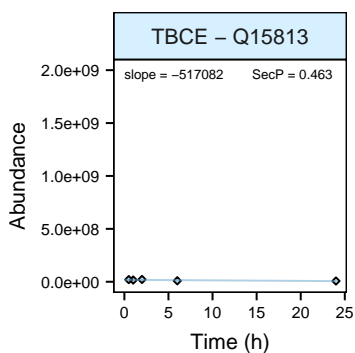

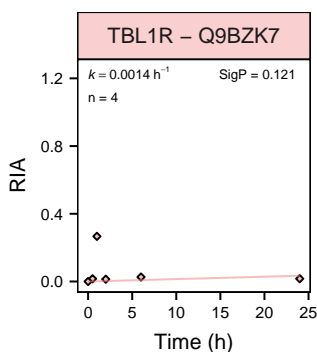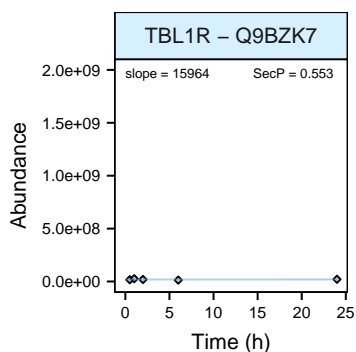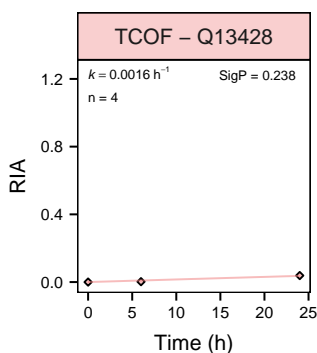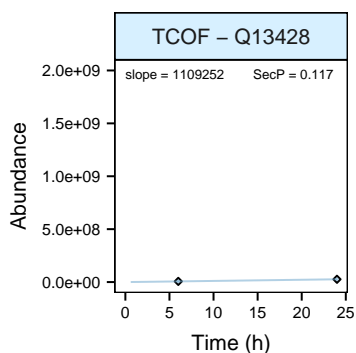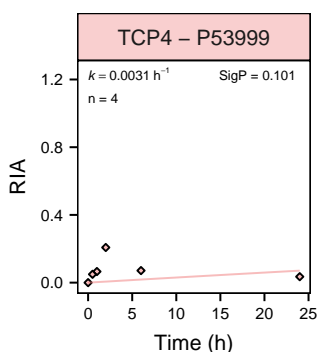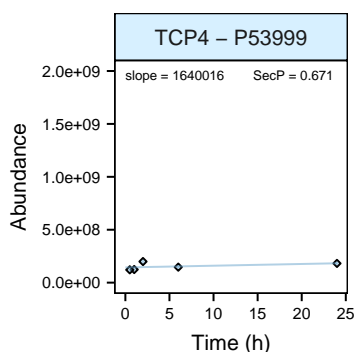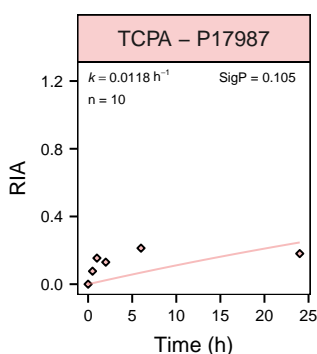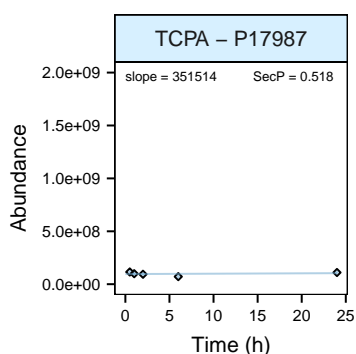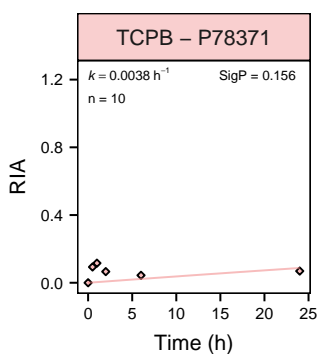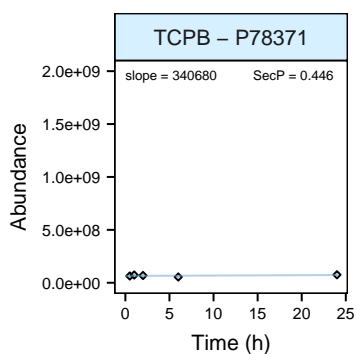

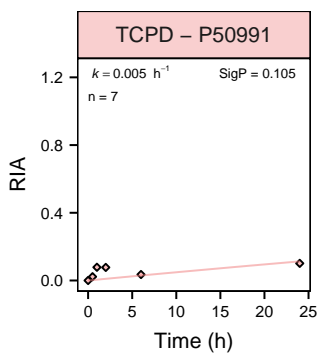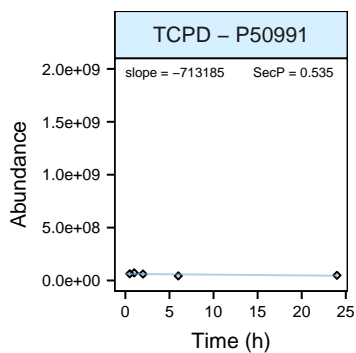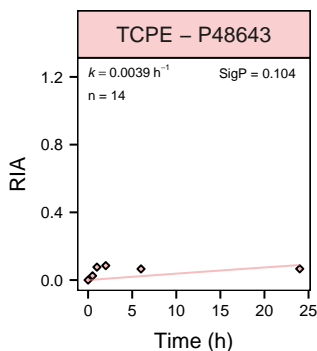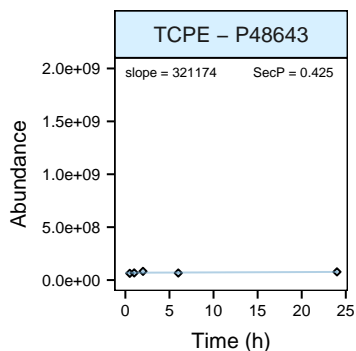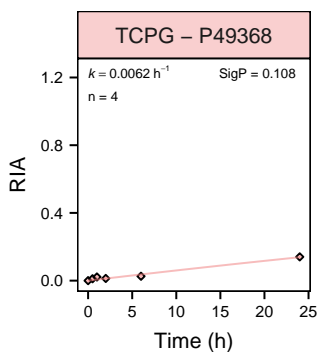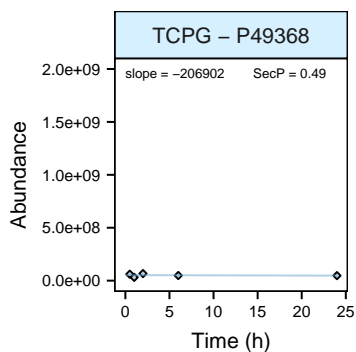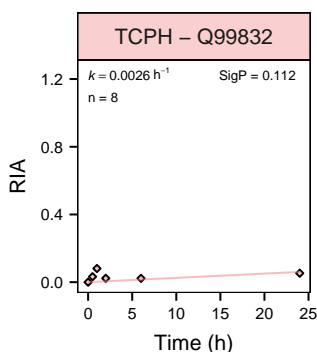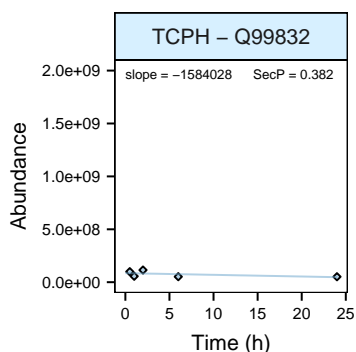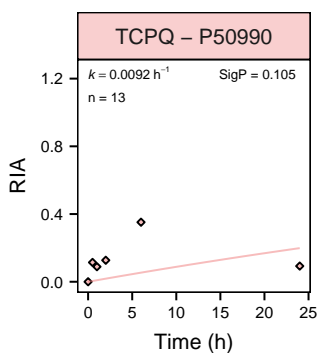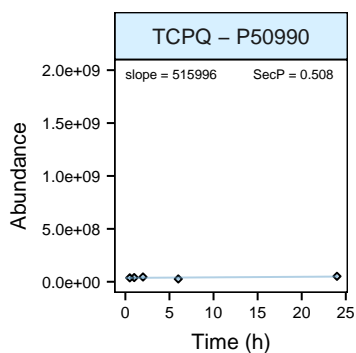

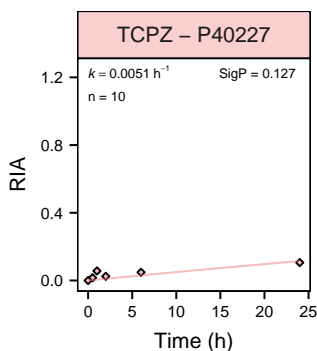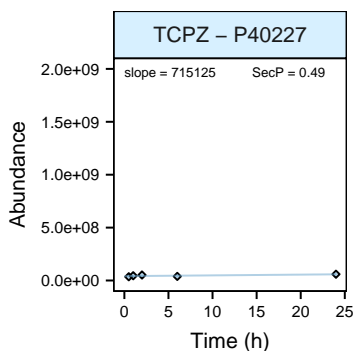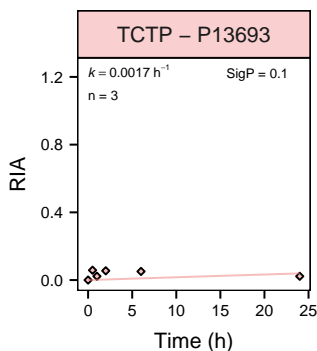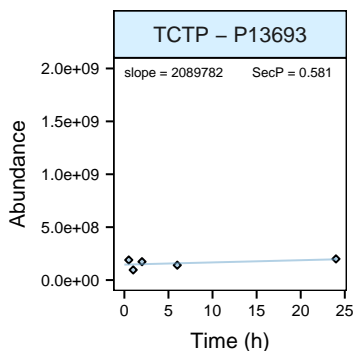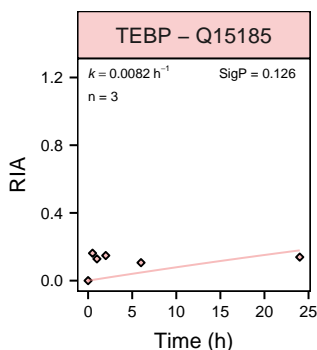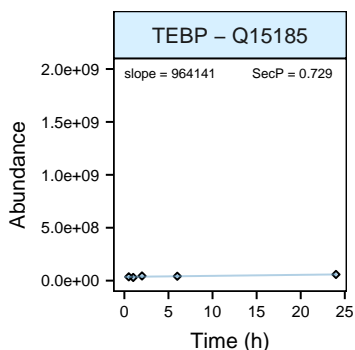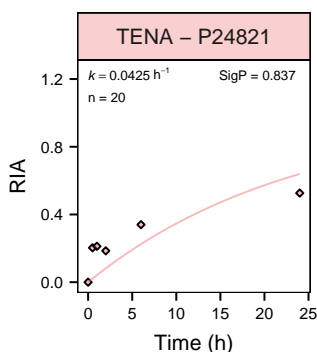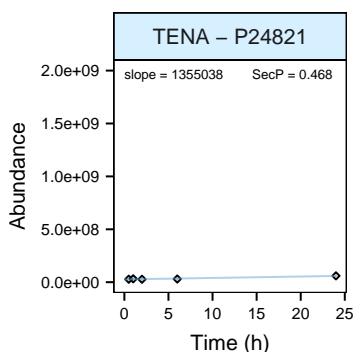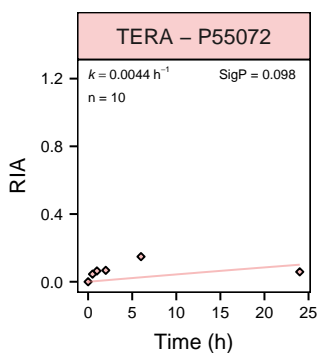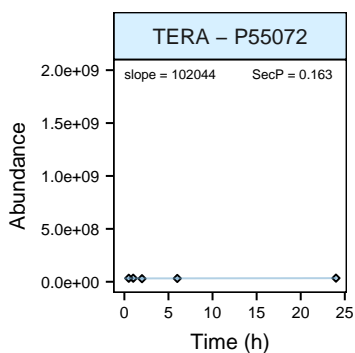

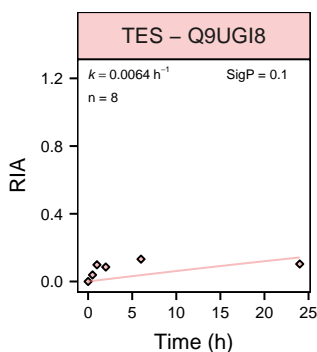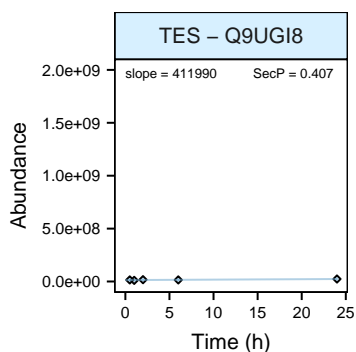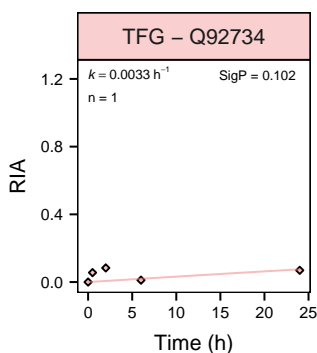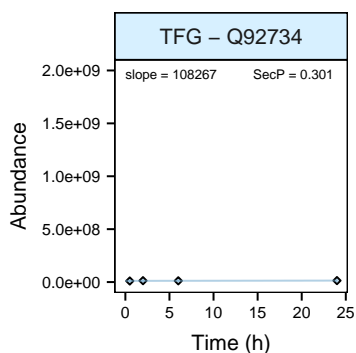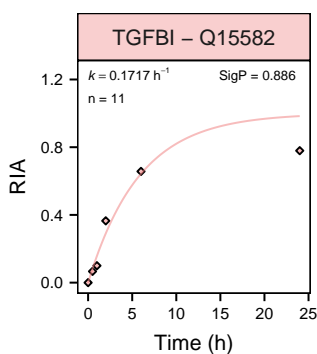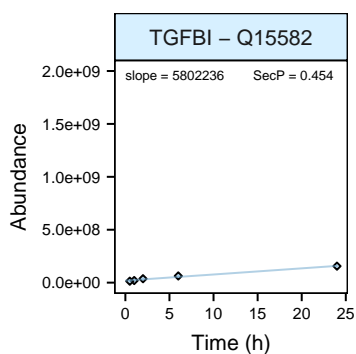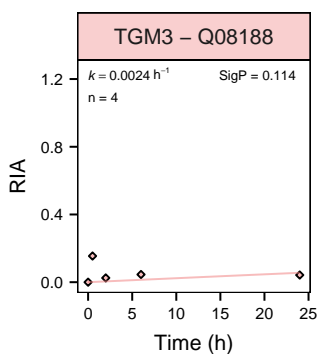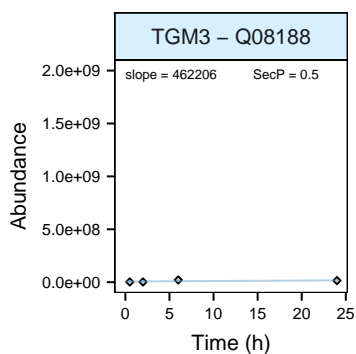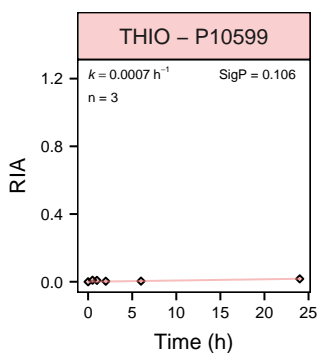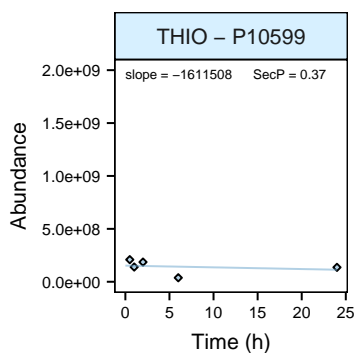

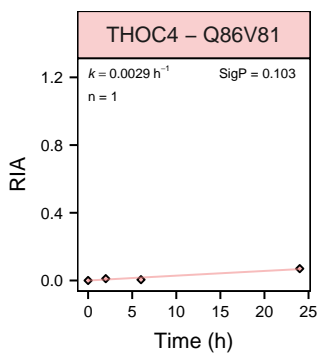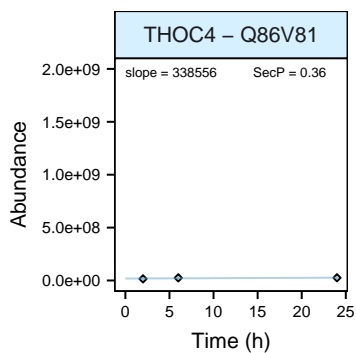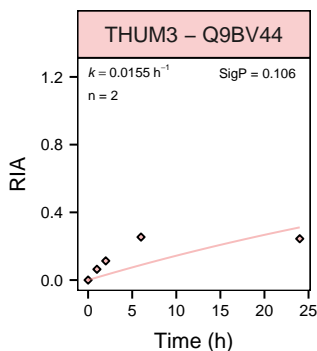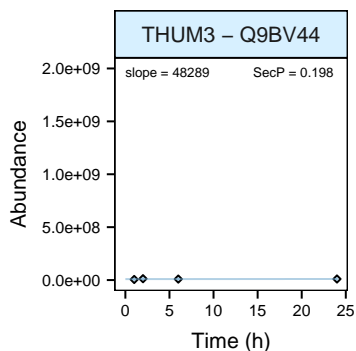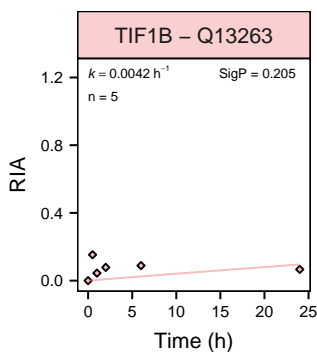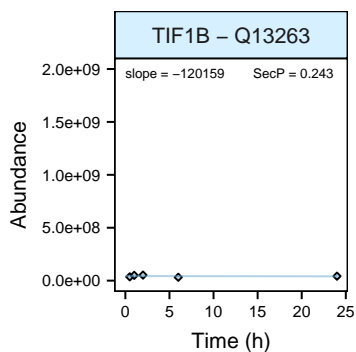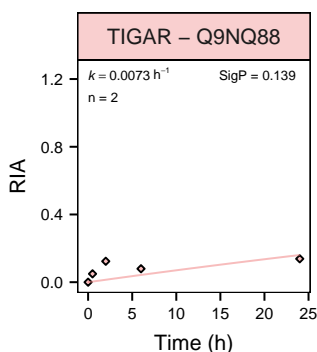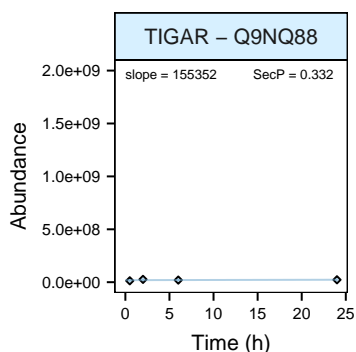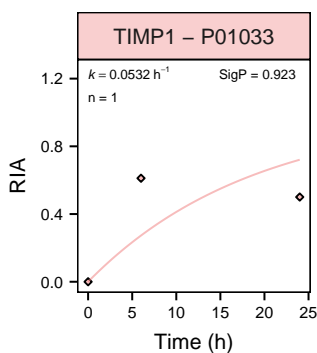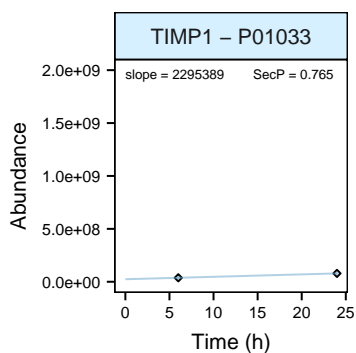

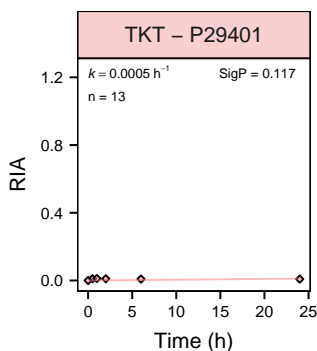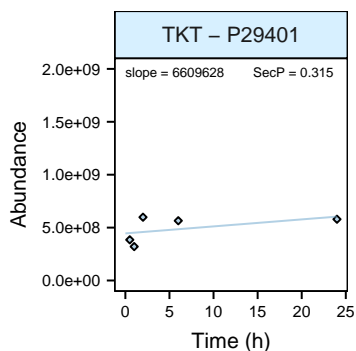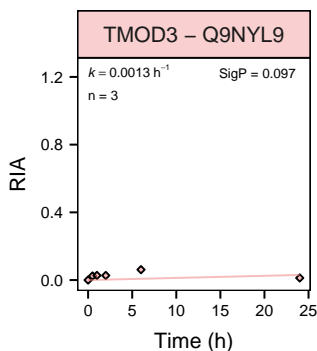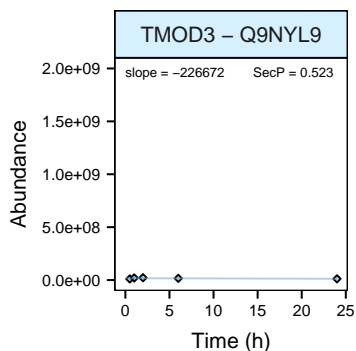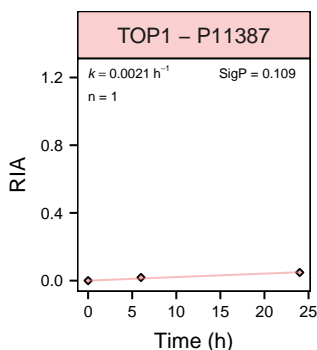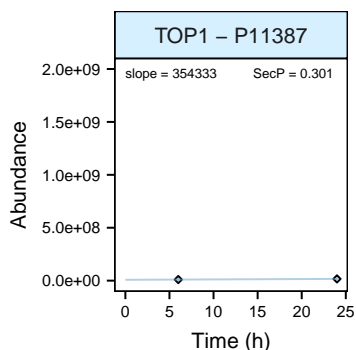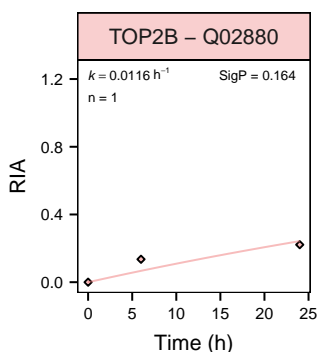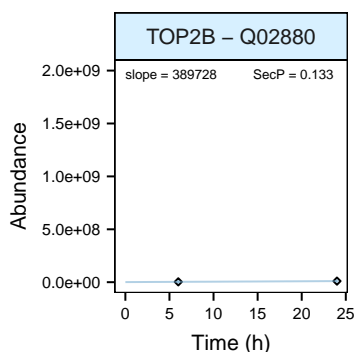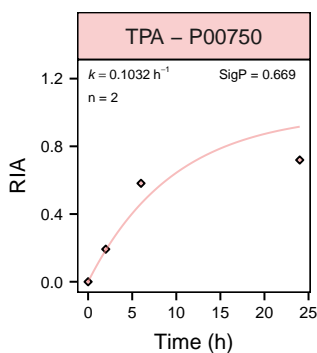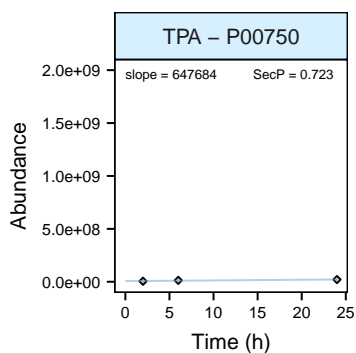

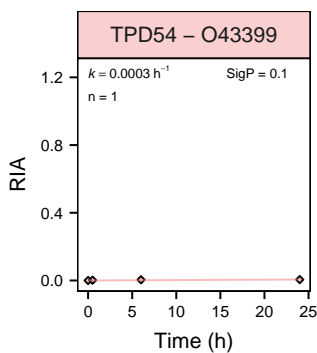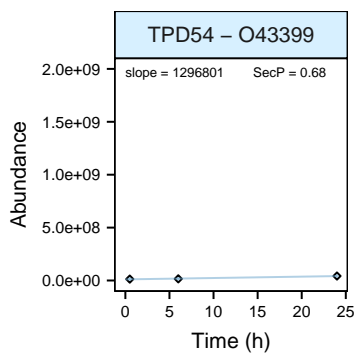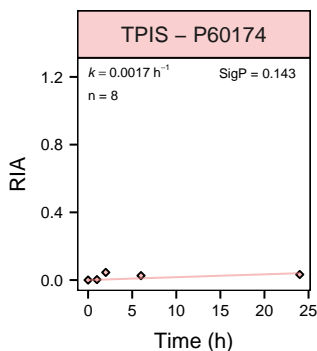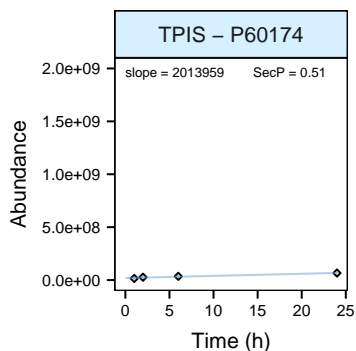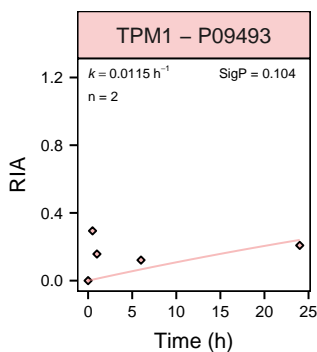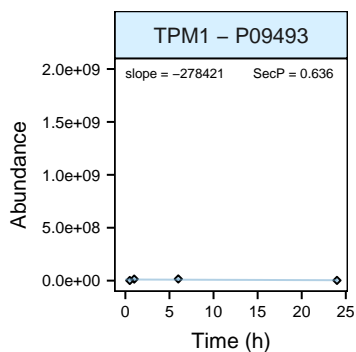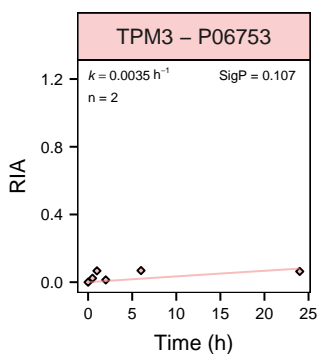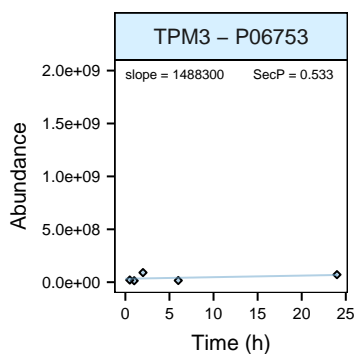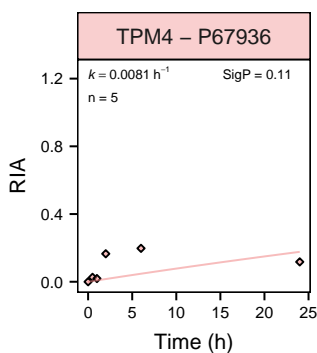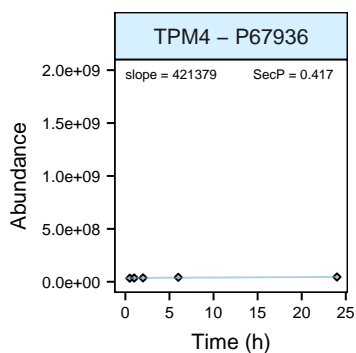

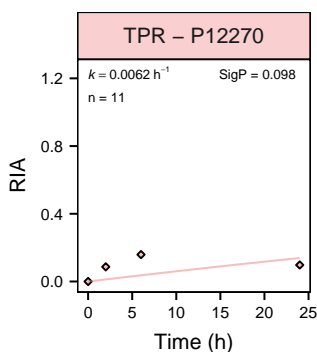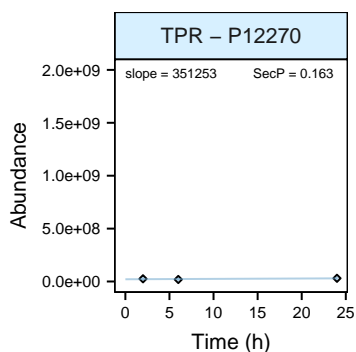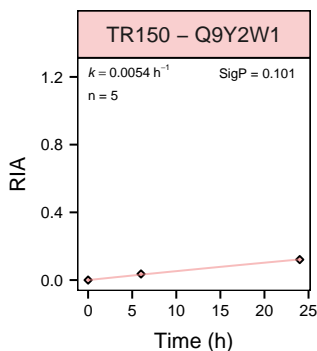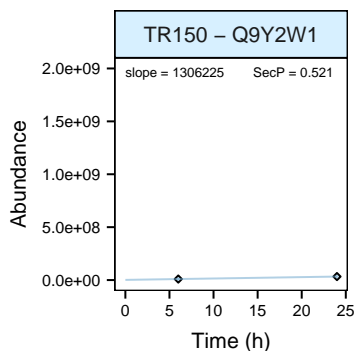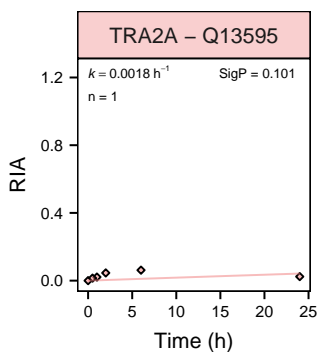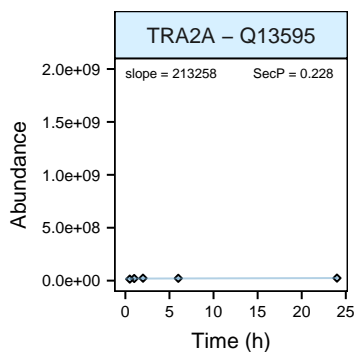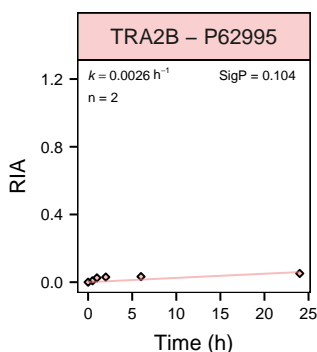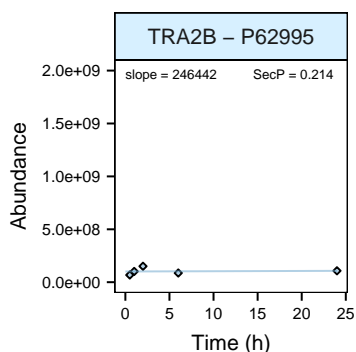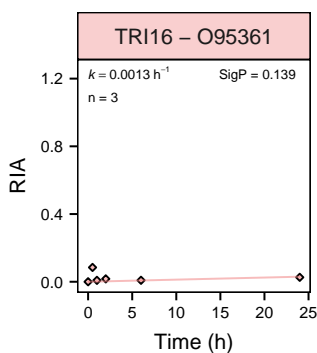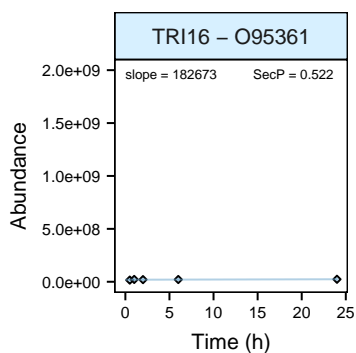

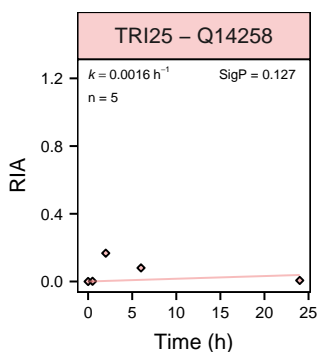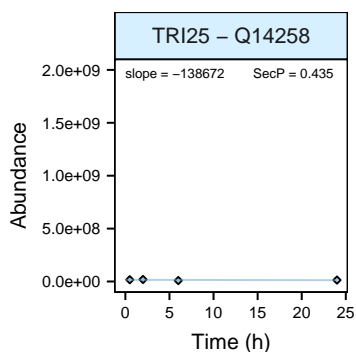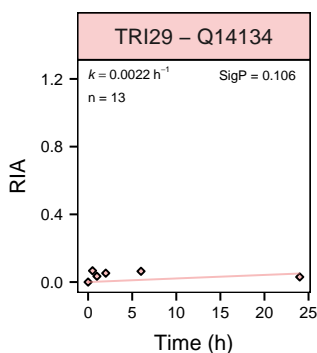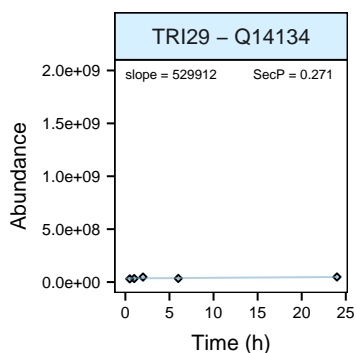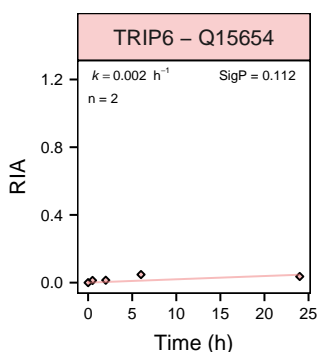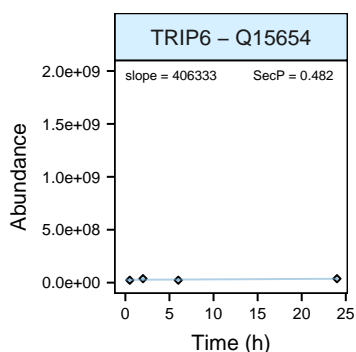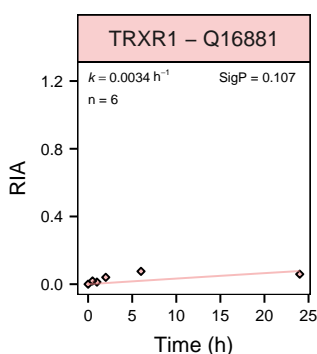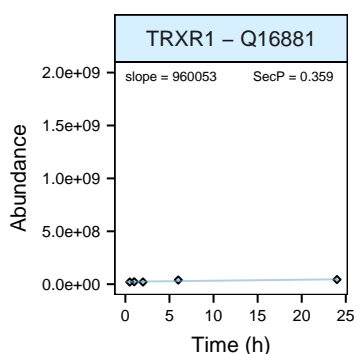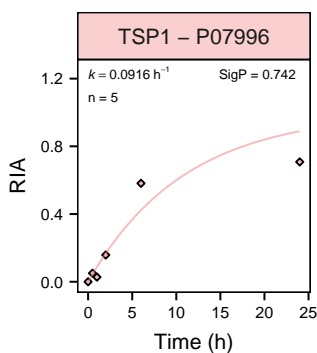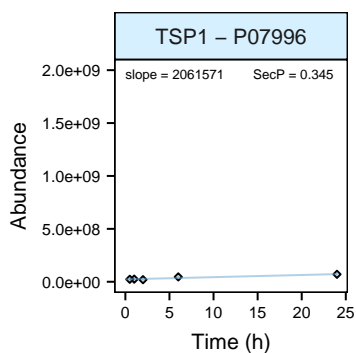

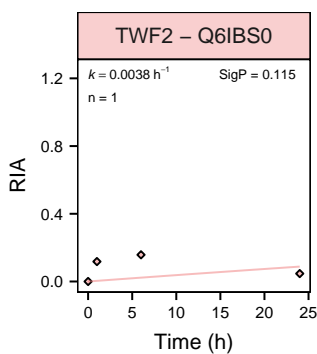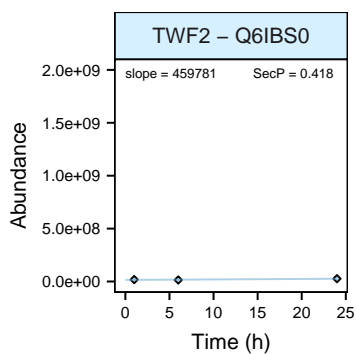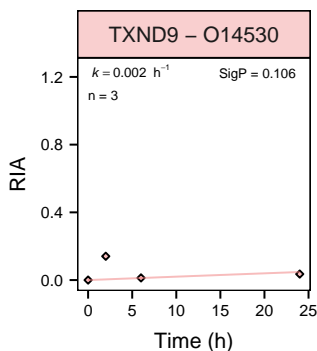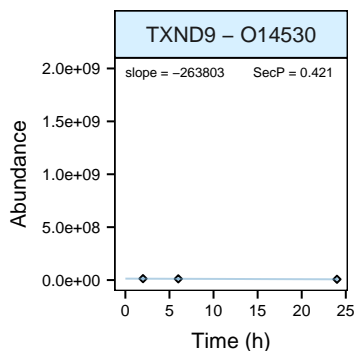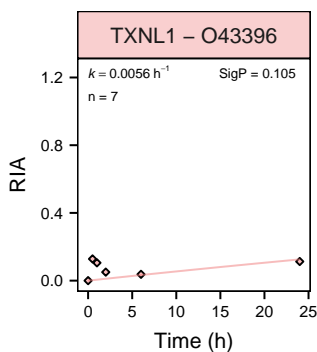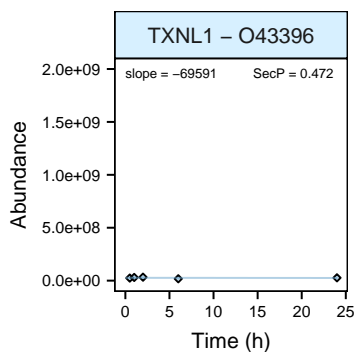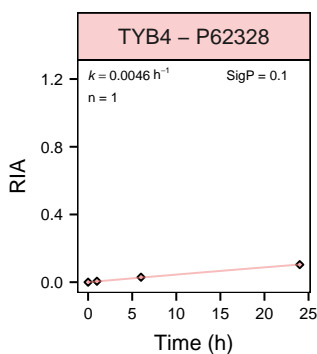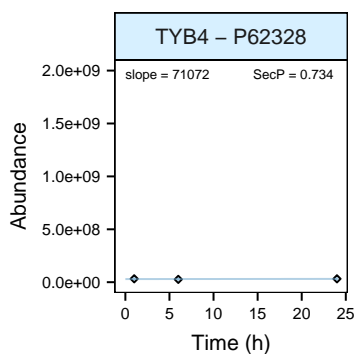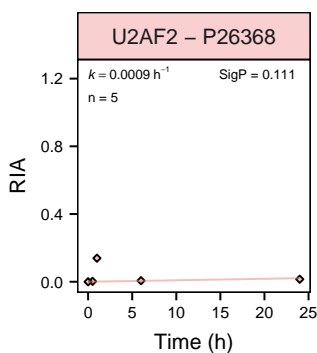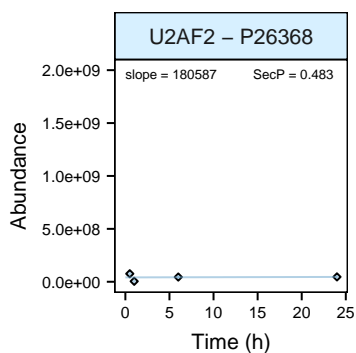

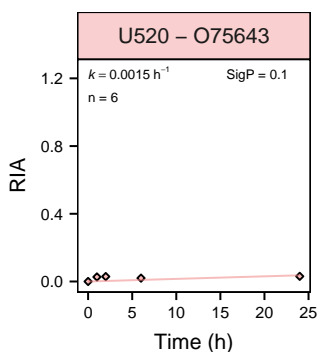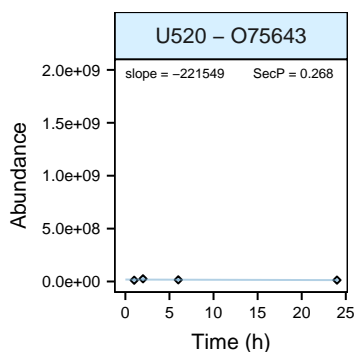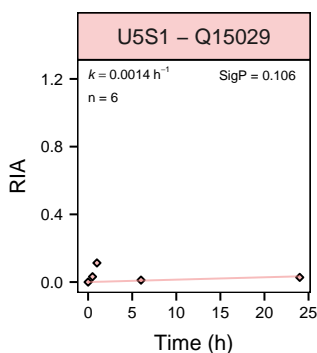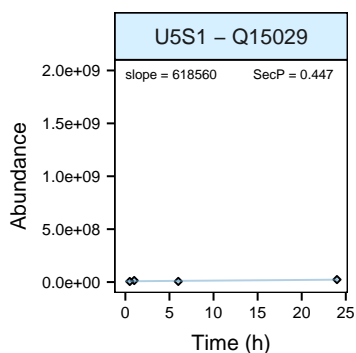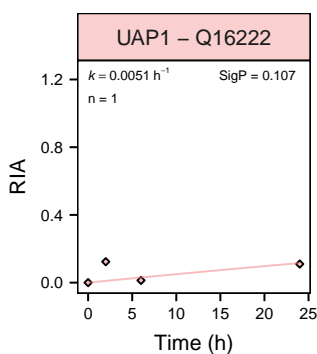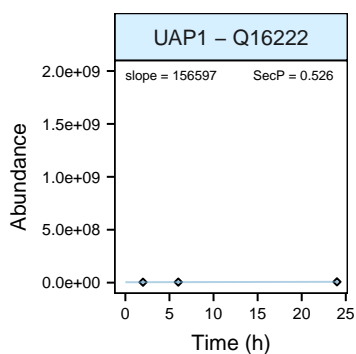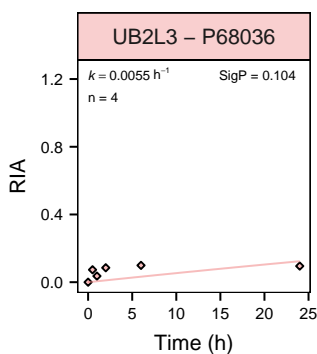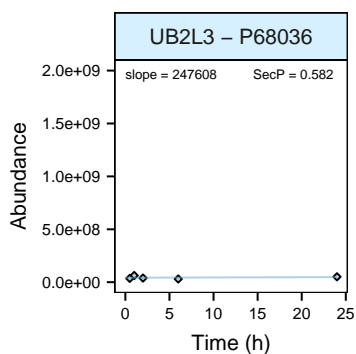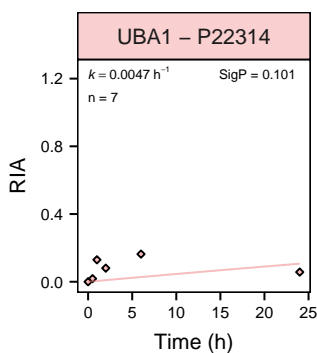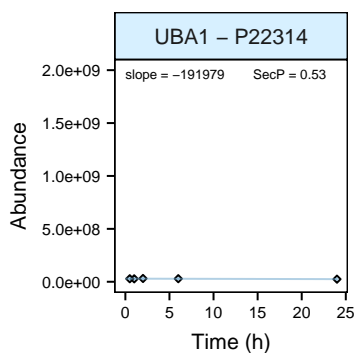

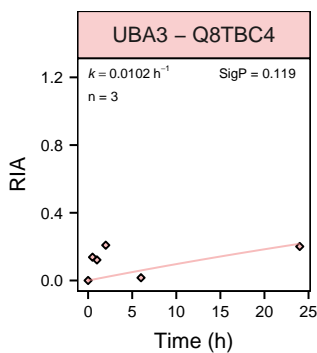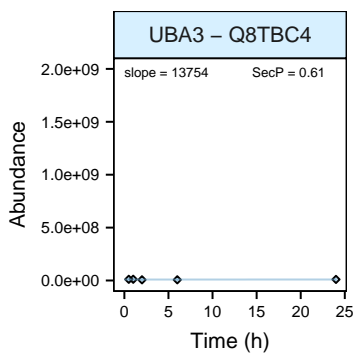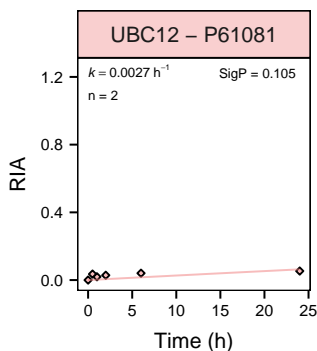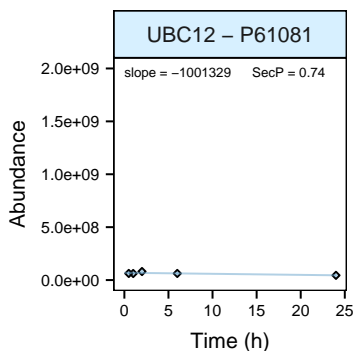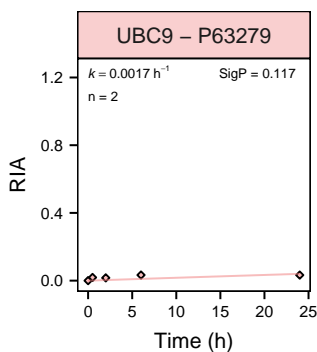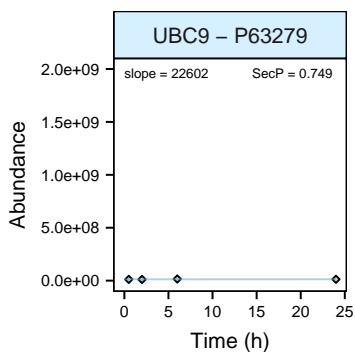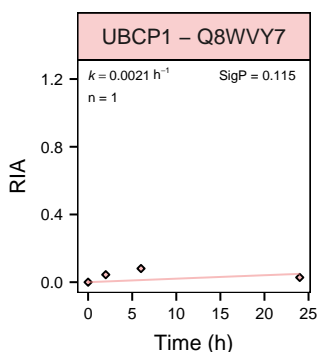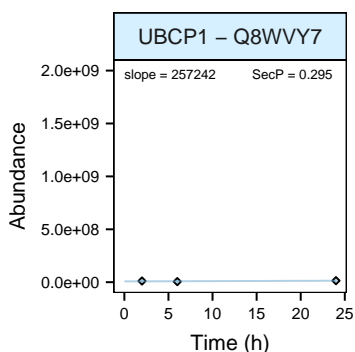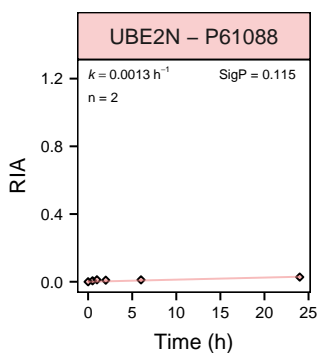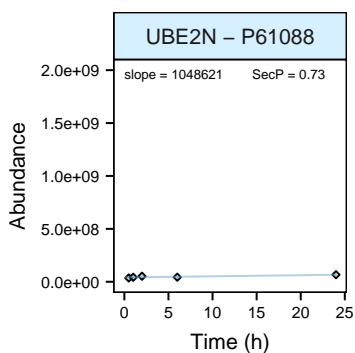

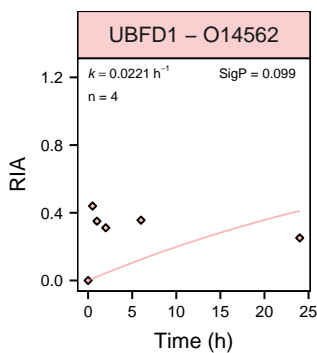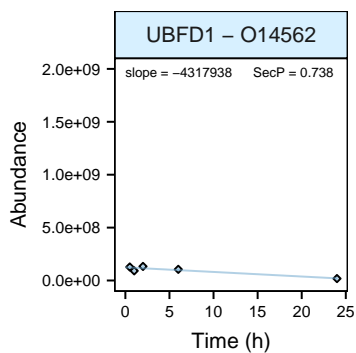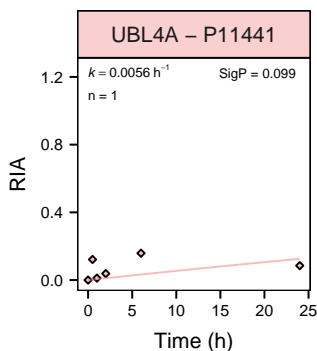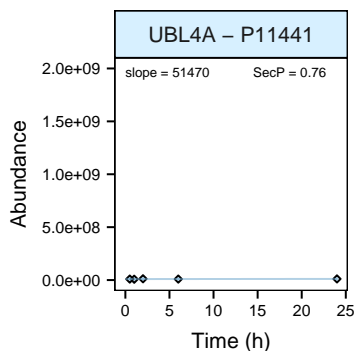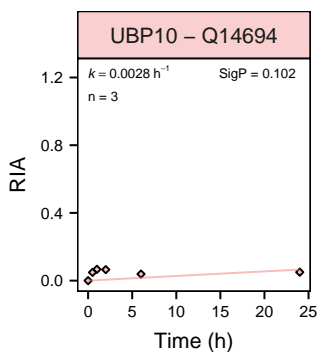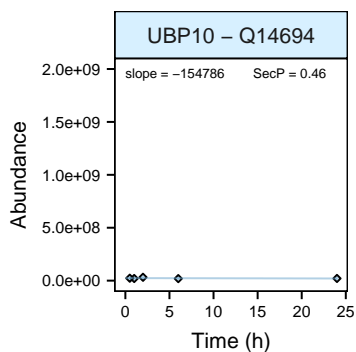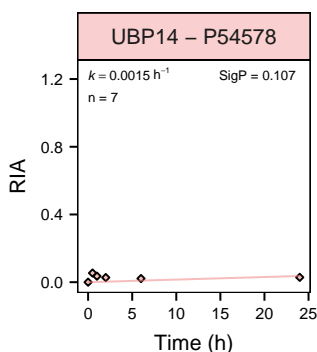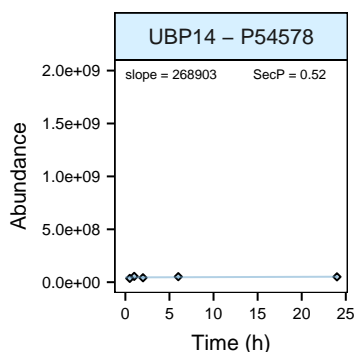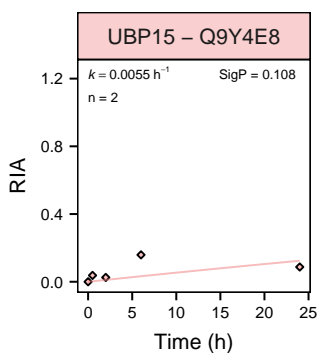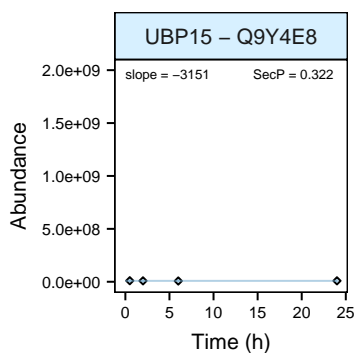

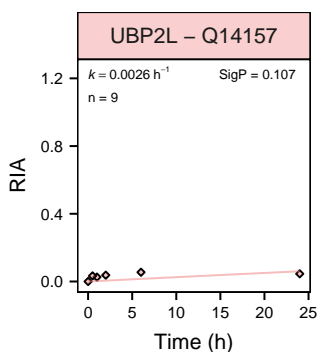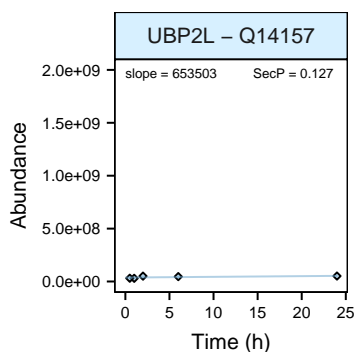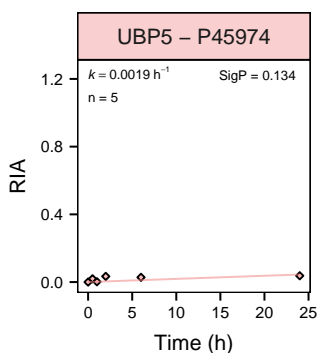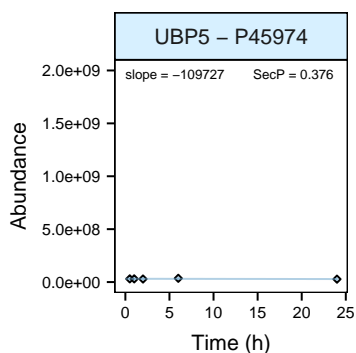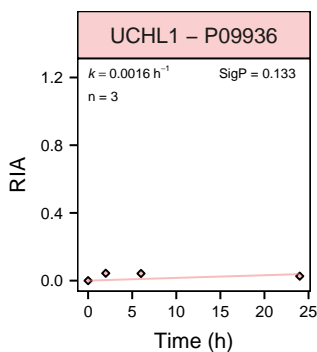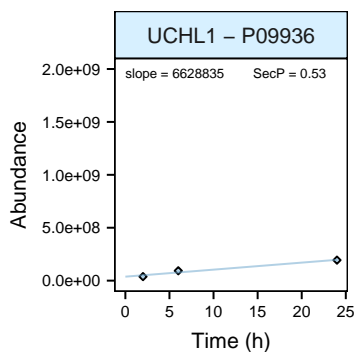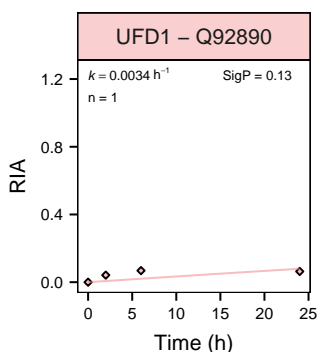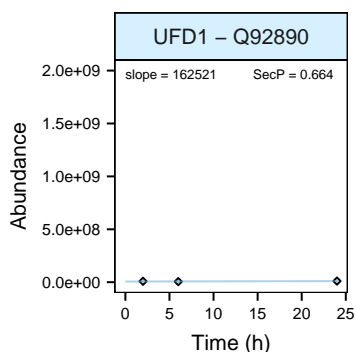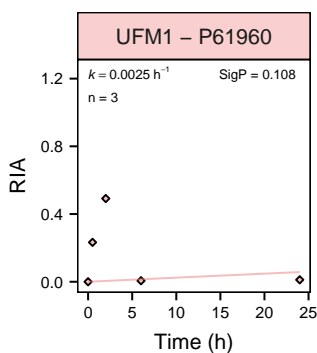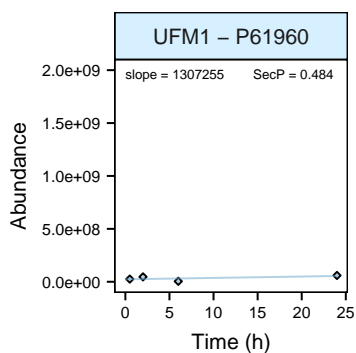

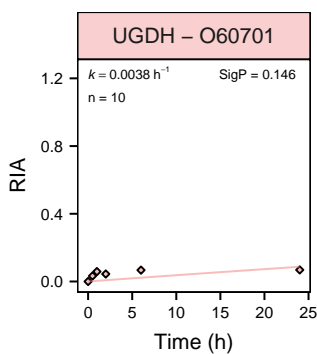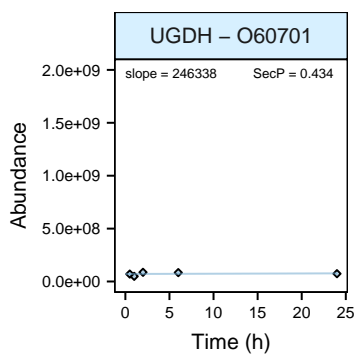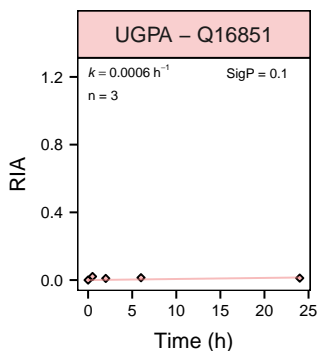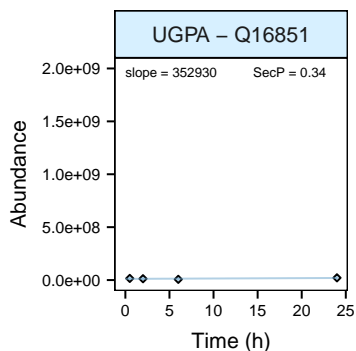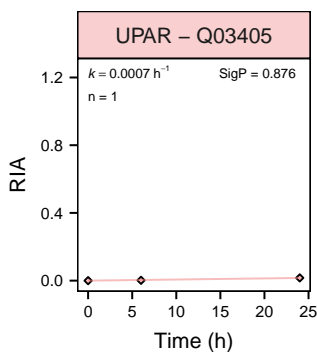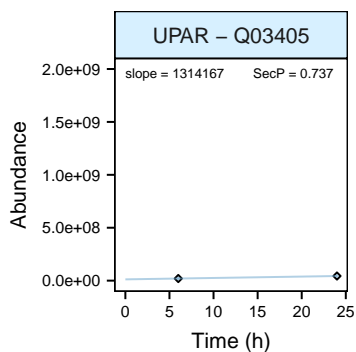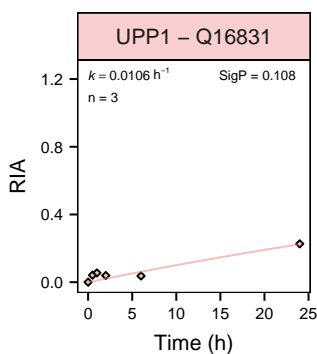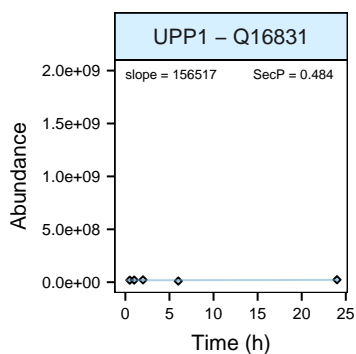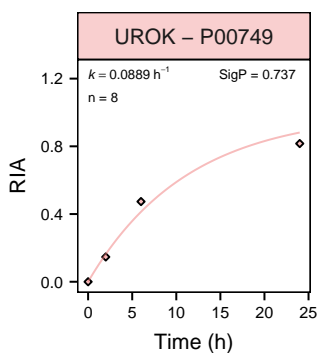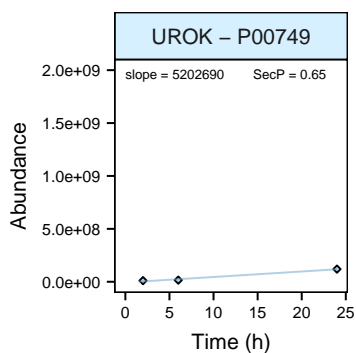

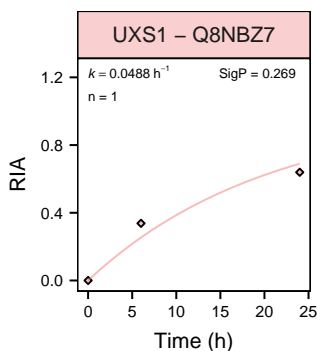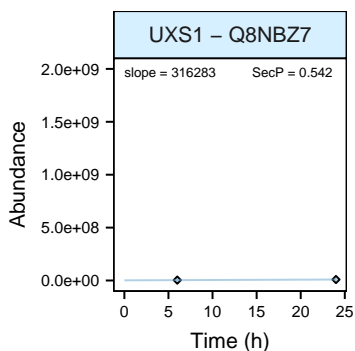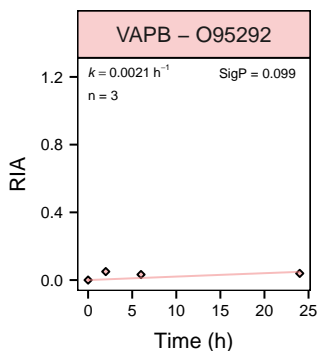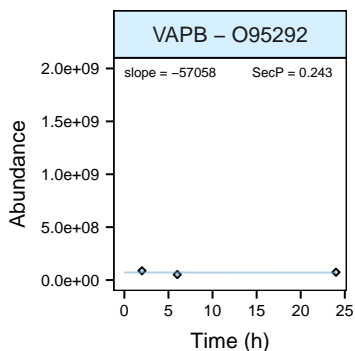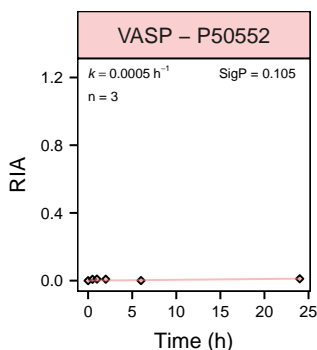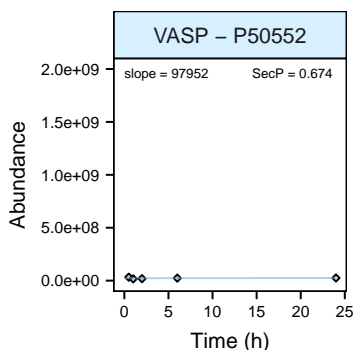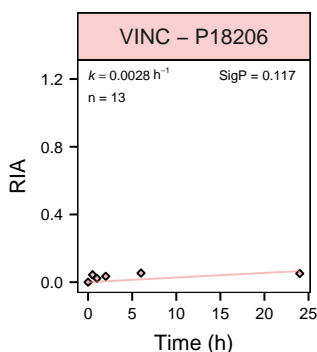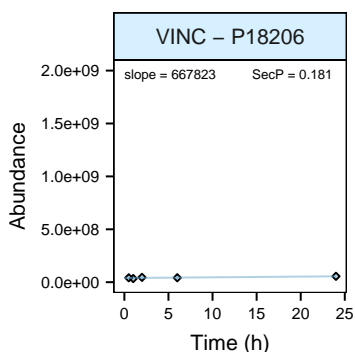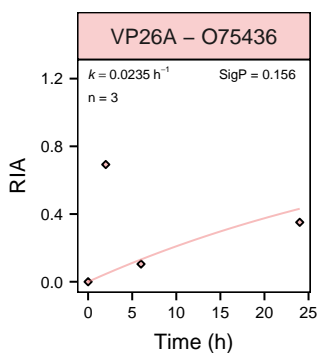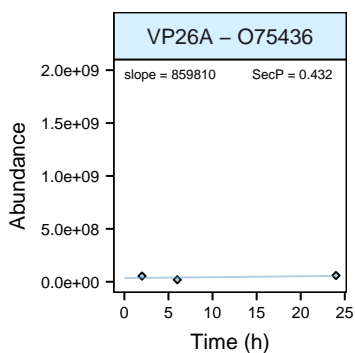

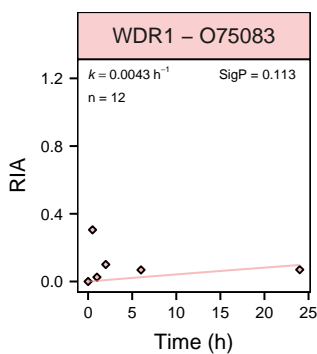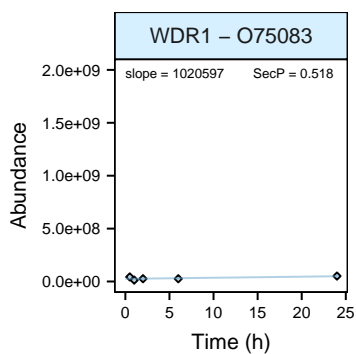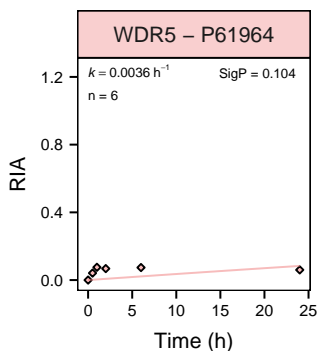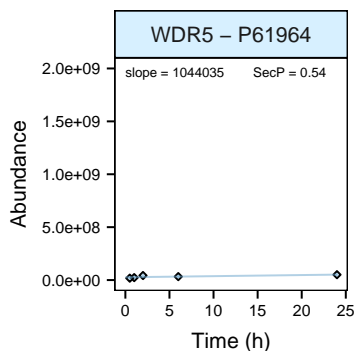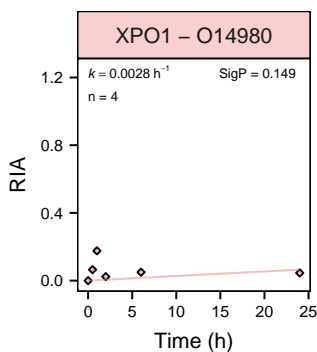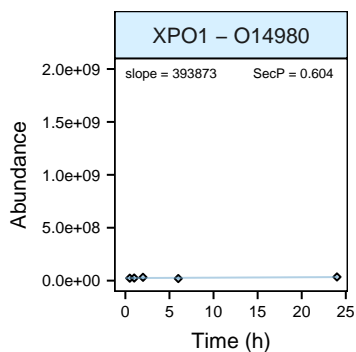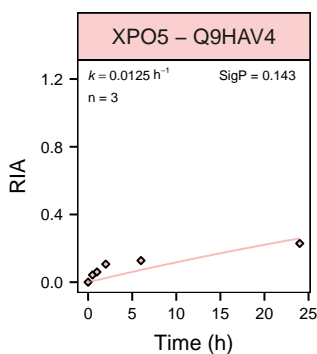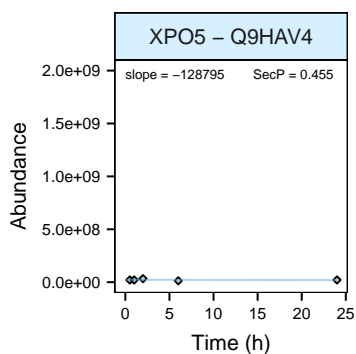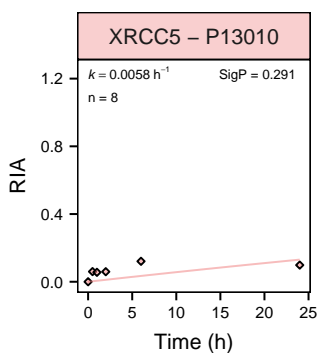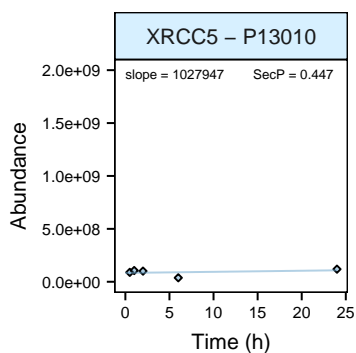

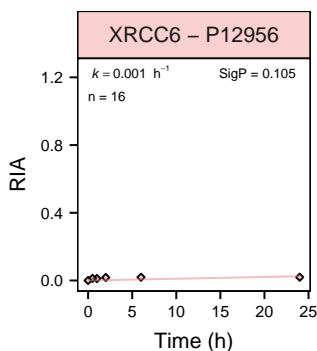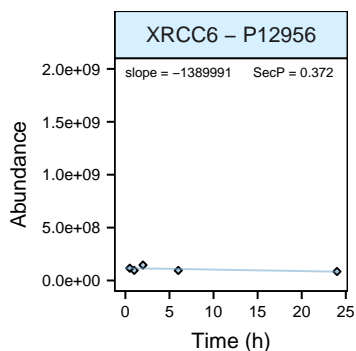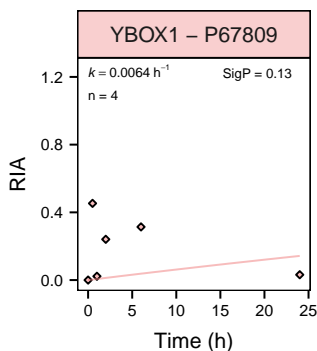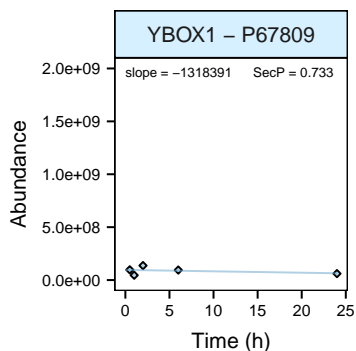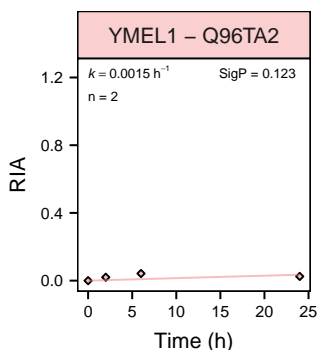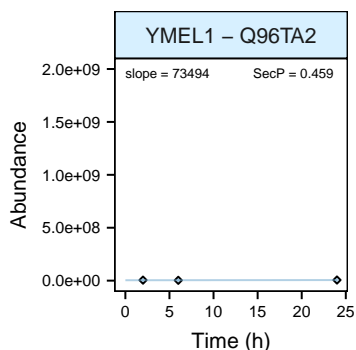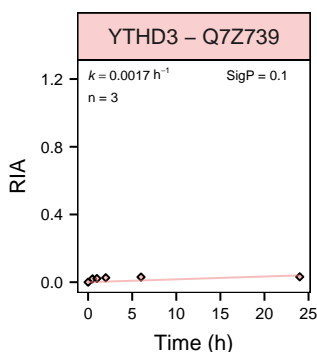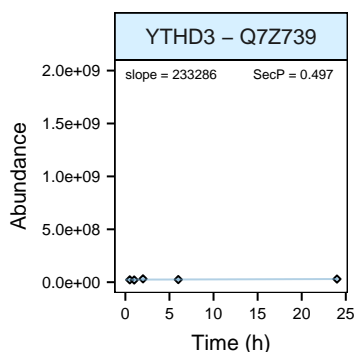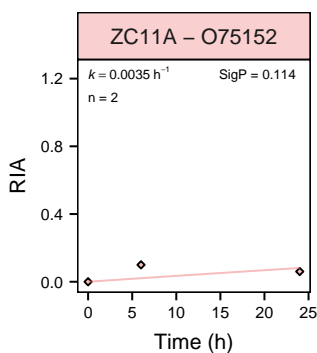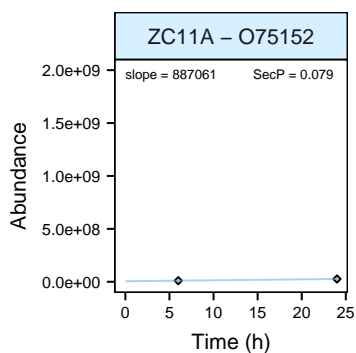

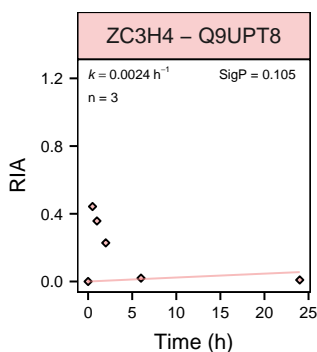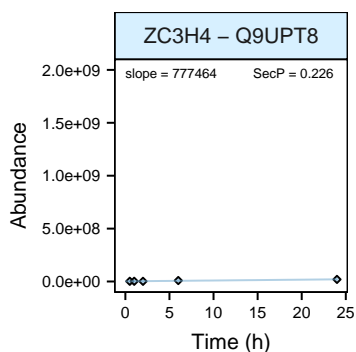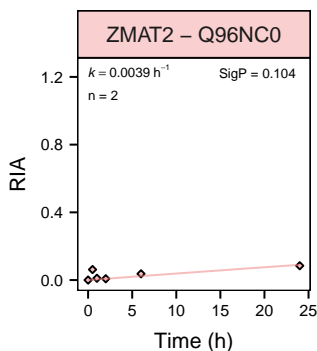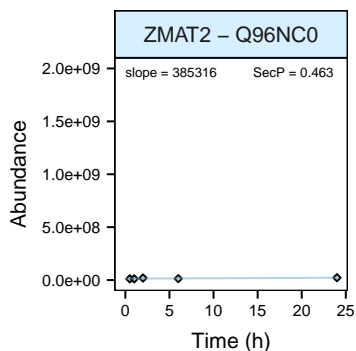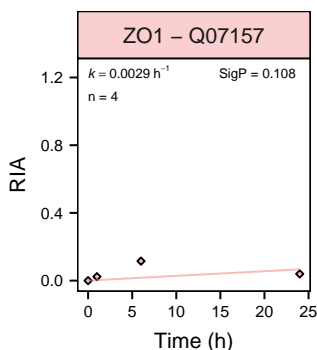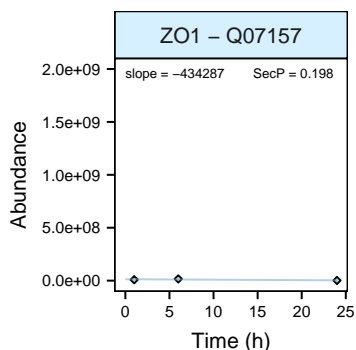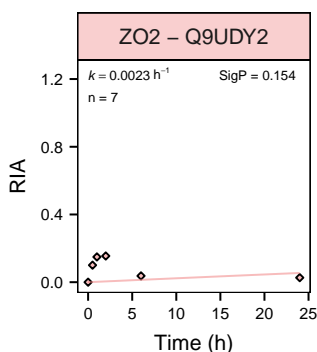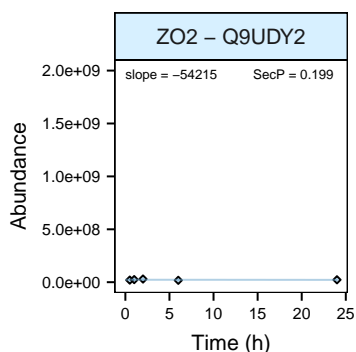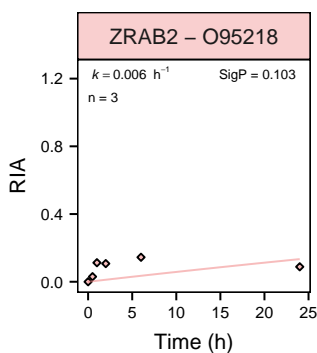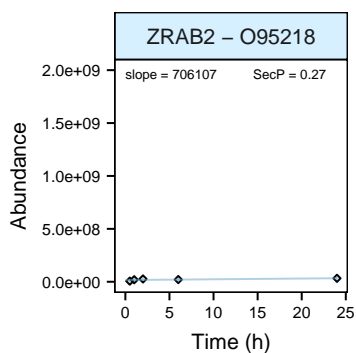

Supplement: supplemental material [file 134290_3_supp_155575_pbbbgs.pdf]

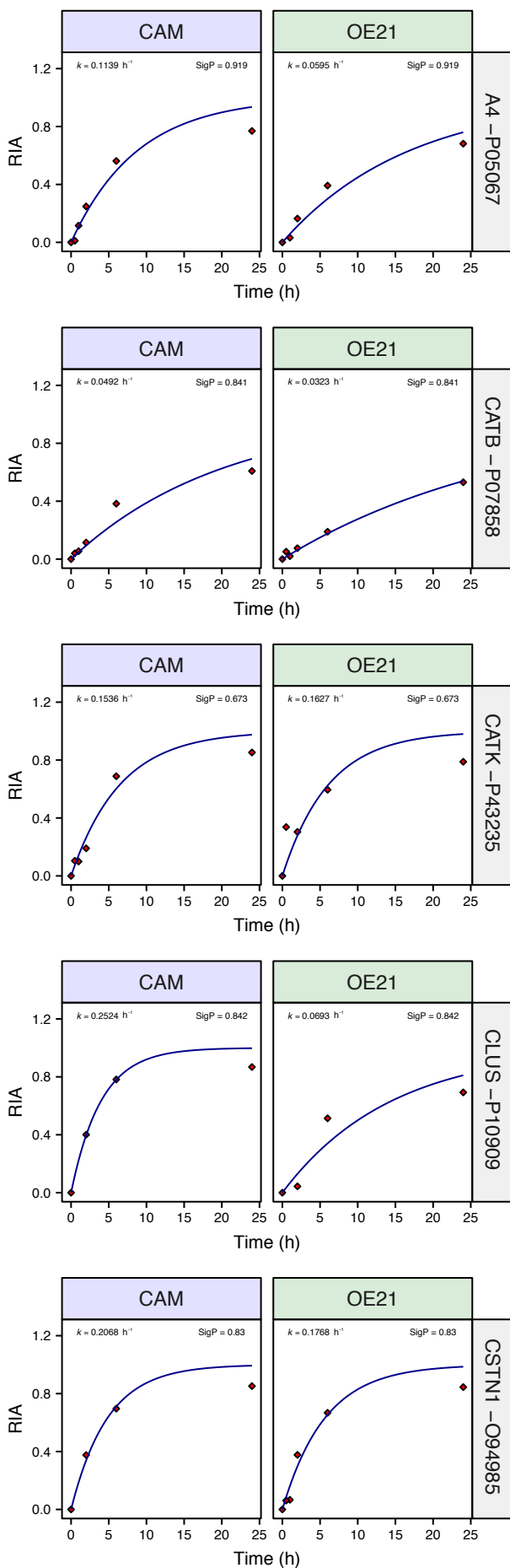

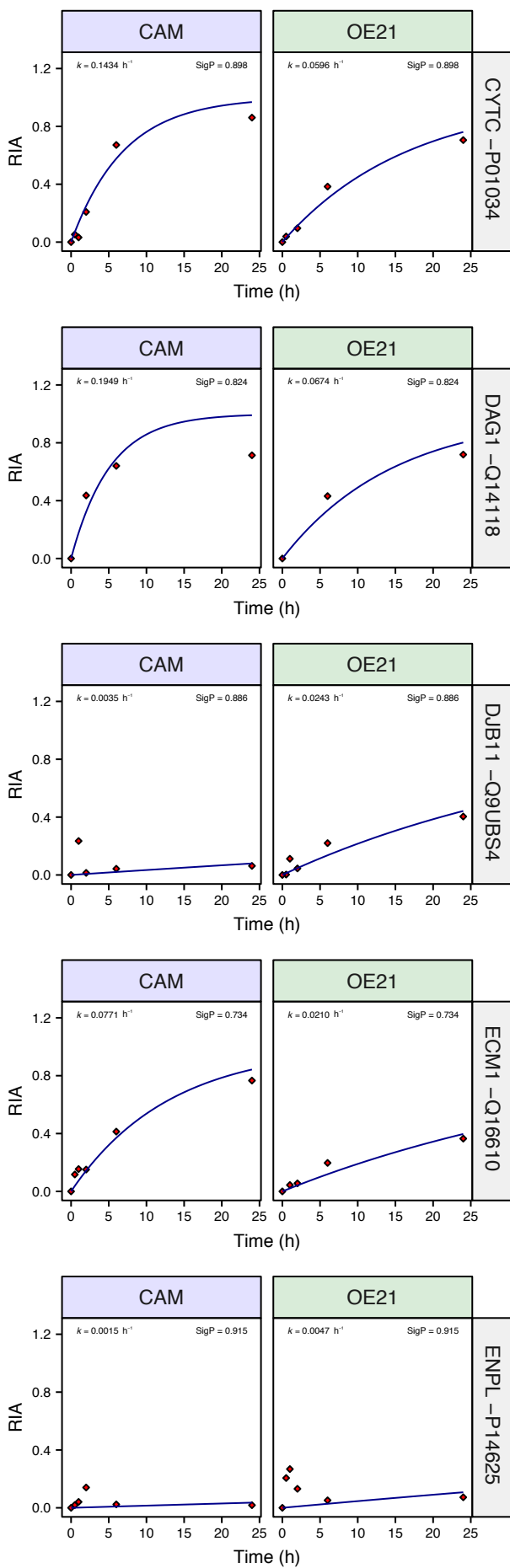

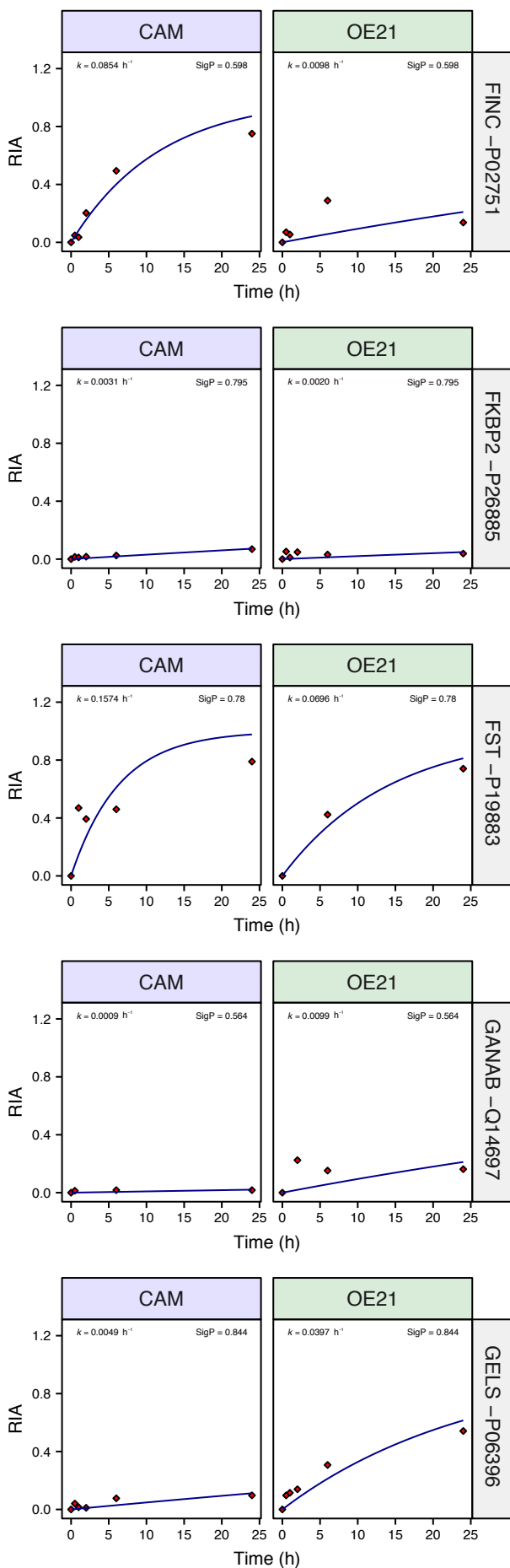

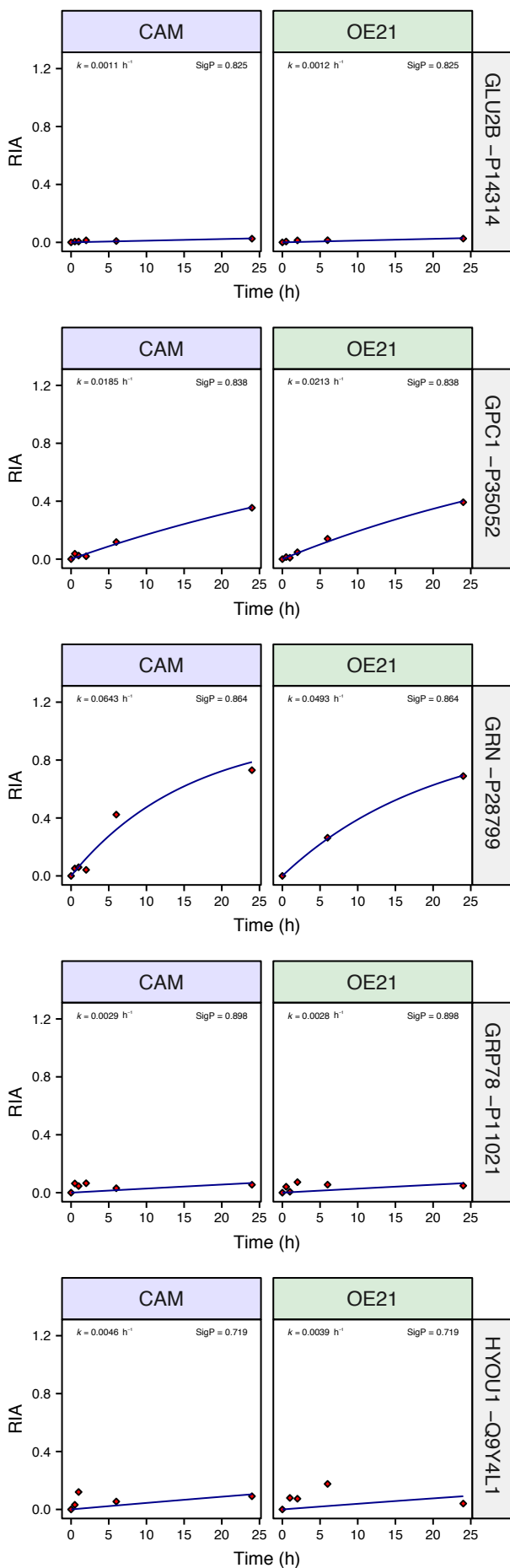

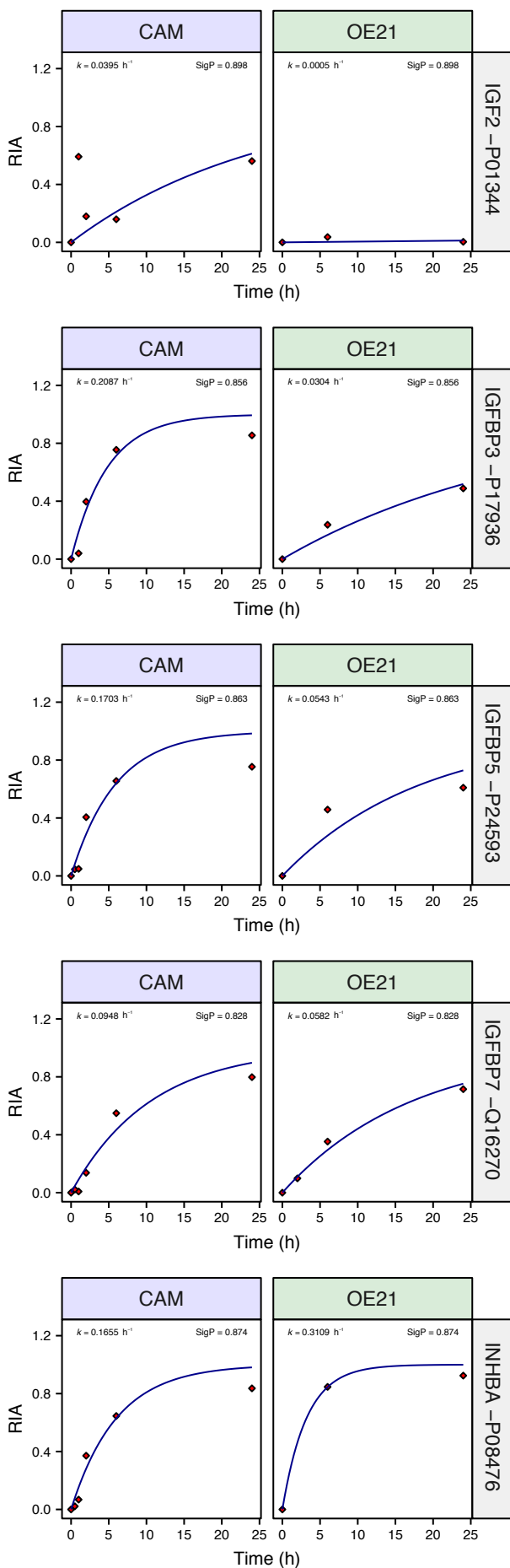

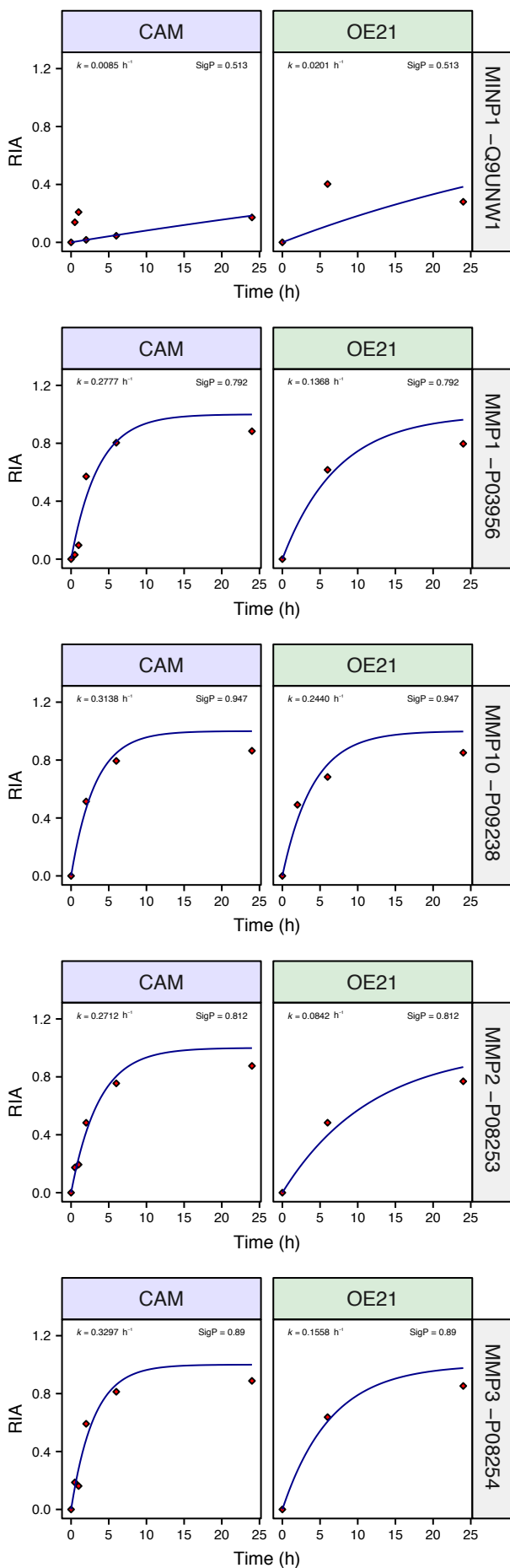

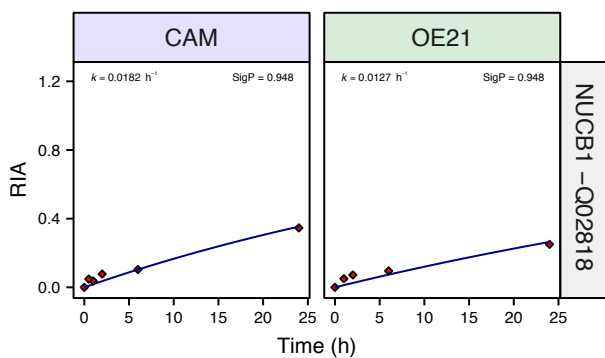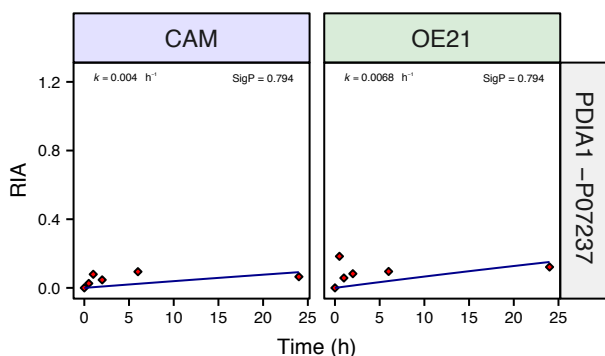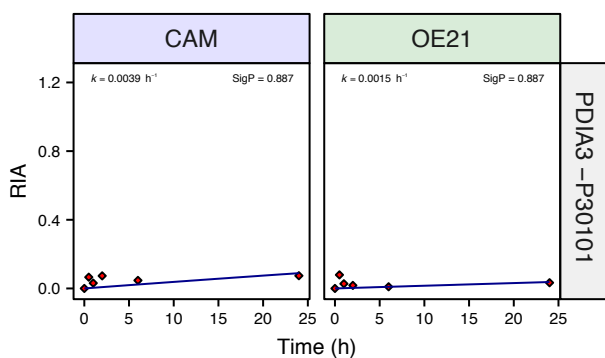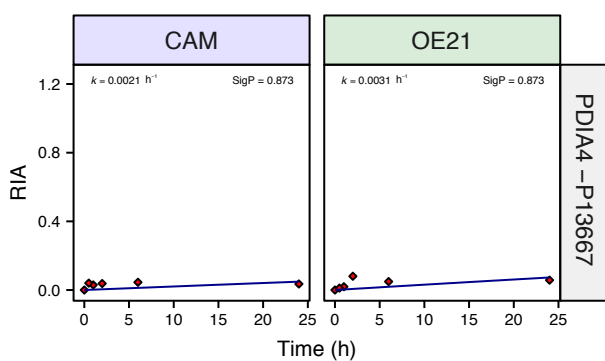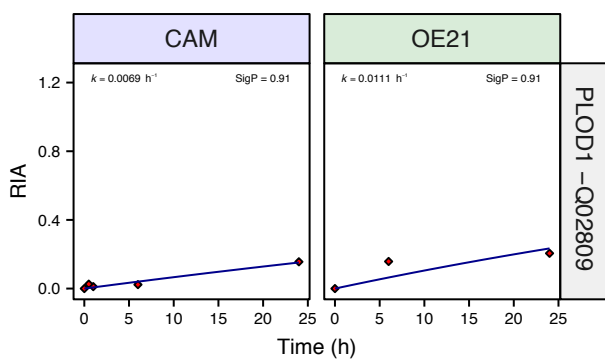

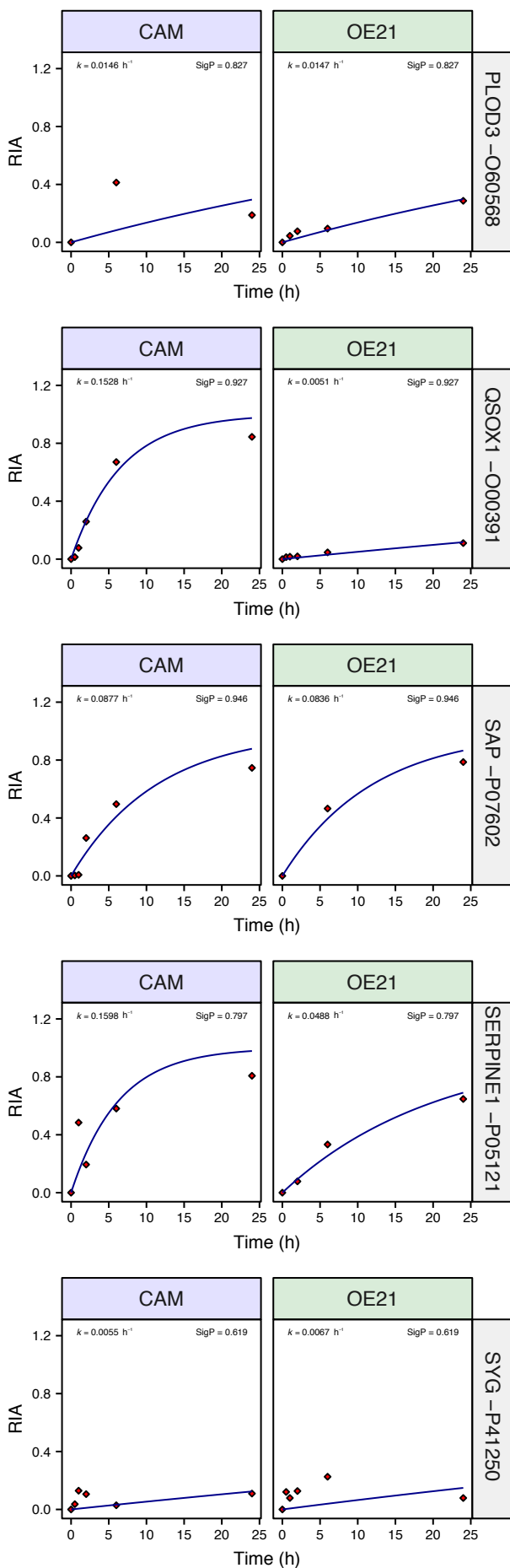

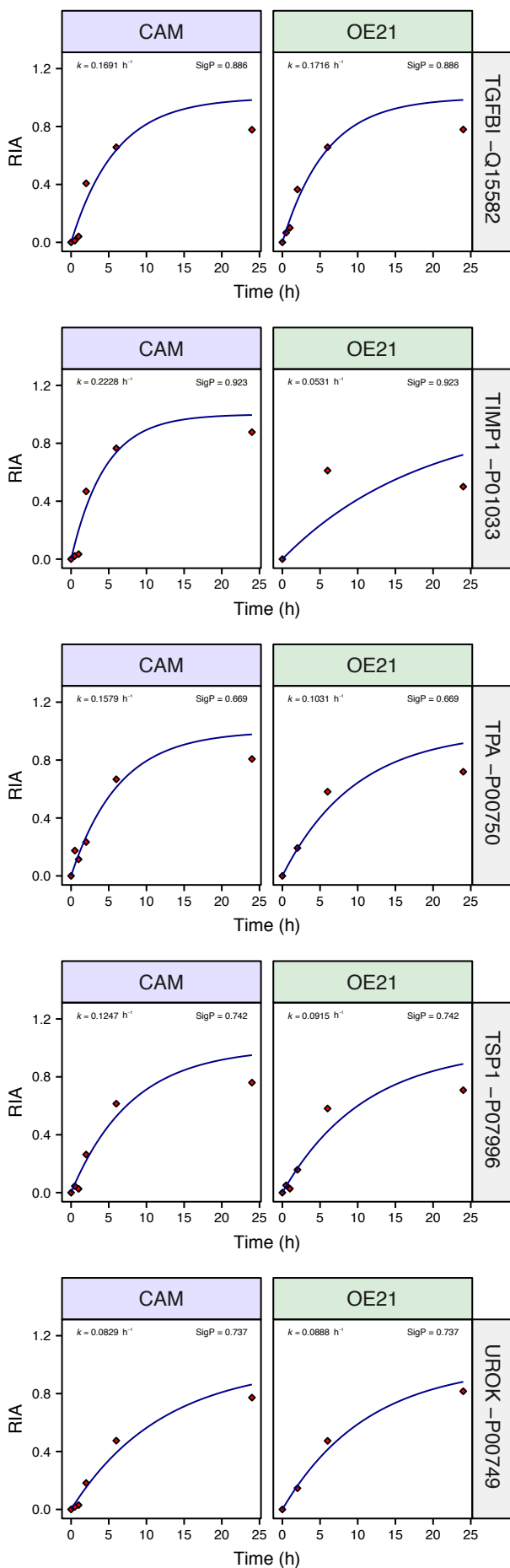

Supplement: supplemental material [file 134290_3_supp_155576_pqqqgs.pdf]
