# Supplementary material for: Stable Isotope Dynamic Labeling of Secretomes (SIDLS) Identifies Authentic Secretory Proteins Released by Cancer and Stromal Cells
Source: Mol Cell Proteomics. 2018 Jun 18;17(9):1837–49. doi: 10.1074/mcp.TIR117.000516 (PMC6126392; doi:10.1074/mcp.TIR117.000516)

Supplemental Figure 5. The mass spectrometry data was analyzed using MaxQuant in an identical manner to that described in the Methods section but with the ‘requant’ function switched off. Shown below are equivalent plots to Figures 3A (**A**), 3B (**B**), 6A (**C**), 6B (**D**), 8A (**E**) and 8B (**F**). Please refer to their corresponding legend in the main manuscript for further information.

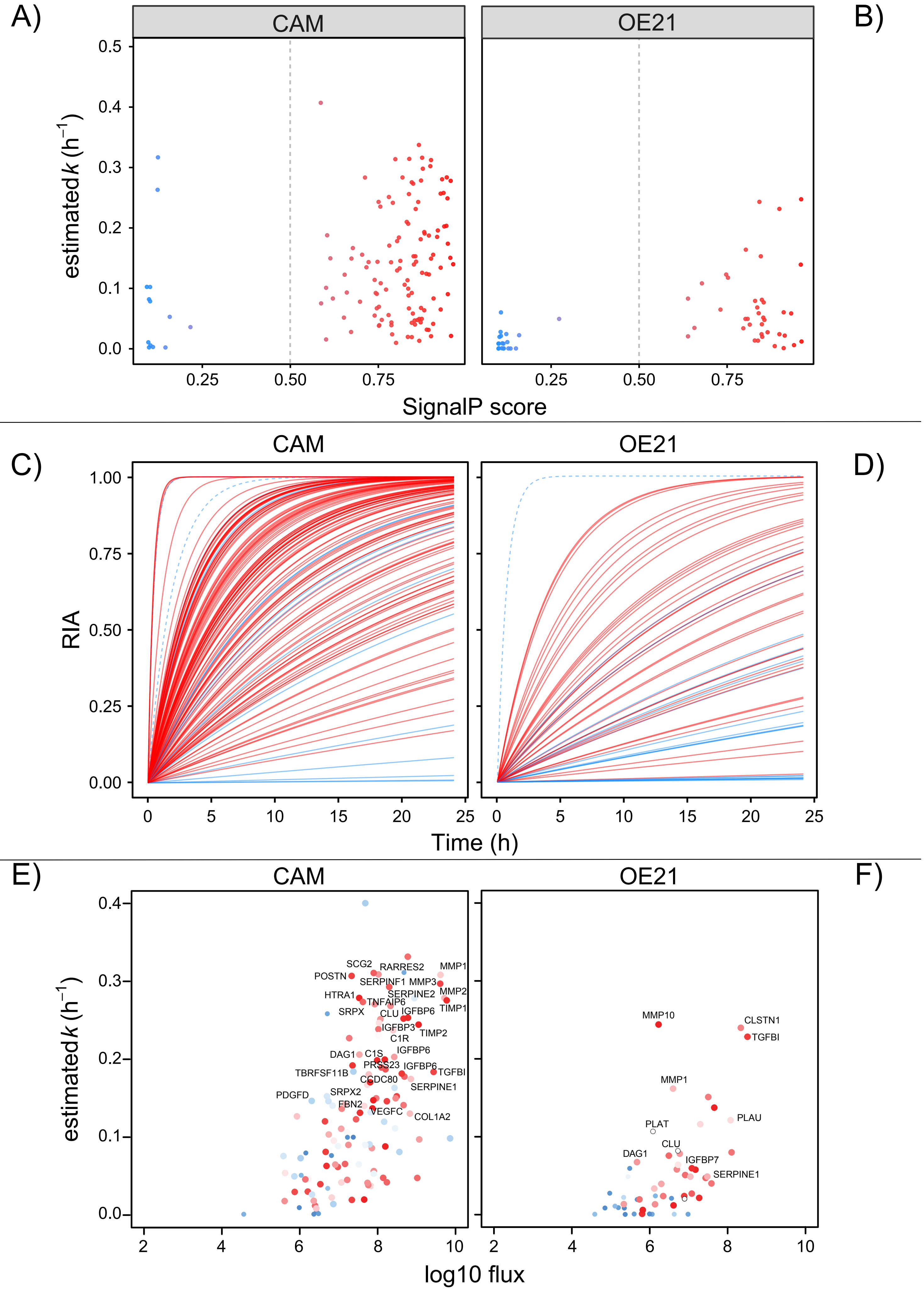

Supplement: supplemental material [file 134290_3_supp_155569_pjjjgp.pdf]
