# Supplementary material for: Stable Isotope Dynamic Labeling of Secretomes (SIDLS) Identifies Authentic Secretory Proteins Released by Cancer and Stromal Cells
Source: Mol Cell Proteomics. 2018 Jun 18;17(9):1837–49. doi: 10.1074/mcp.TIR117.000516 (PMC6126392; doi:10.1074/mcp.TIR117.000516)
Supplement: supplemental material [file 134290_3_supp_155568_pggggp.docx]

**Hammond et al.**

**Summary of Supplemental files.**

**Supplemental Figure 1**

Linearity of StrataClean capture method.

**Supplemental Figure 2**

Labelling trajectory for 1100+ secretome proteins from CAM cells.

**Supplemental Figure 3**

Labelling trajectory for 1800+ secretome proteins from OE21 cells.

**Supplemental Figure 4**

Comparison of labelling curves for the same secreted protein from CAM and OE21 cells.

**Supplemental Figure 5**

Selected equivalent Figures from the main manuscript, from data analysed using MaxQuant with the ‘requant’ function switched off.

**Supplementary Table 1**

MaxQuant output from the Linearity of StrataClean capture method; specifically, the evidence.txt file and proteinGroups.txt file.

**Supplementary Table 2**

Tables showing the pre- and post-filtered data-sets for both cell-lines. Refer to the main manuscript for the filtering applied. Each table has information on the peptide/protein identity (Unique_ID, UniProt accession), peptide sequence, number of peptides analysed per protein, charge state, m/z, retention time on chromatography column, search engine (Andromeda) score, posterior error probability (PEP) score, intensity/abundance of “light” SILAC feature, intensity/abundance of “heavy” SILAC feature, total abundance (H+L), time (h) in labelling trajectory, relative isotope abundance (RIA). Also included in the filtered data is information relating to SignalP and SecretomeP score.

**Supplementary Table 3**

MaxQuant output from the SIDLS method; specifically, the evidence.txt file and proteinGroups.txt file.
